# Supplementary material for: Synthetic hybrids of six yeast species
Source: Nat Commun. 2020 Apr 29;11:2085. doi: 10.1038/s41467-020-15559-4 (PMC7190663; doi:10.1038/s41467-020-15559-4)
Supplement: Supplementary file 1 — Supplementary Information [file 41467_2020_15559_MOESM1_ESM.pdf]

# Supplementary Information for

**Synthetic hybrids of six yeast species**

Peris *et al*.

Correspondence to: cthittinger@wisc.edu (CTH), david.perisnavarro@gmail.com (DP)

**This PDF file includes:**

**Supplementary text**
- Note 1
- Note 2

**Supplementary figures**
- Figure 1
- Figure 2
- Figure 3
- Figure 4
- Figure 5
- Figure 6
- Figure 7
- Figure 8
- Figure 9

**References**

## Supplementary text

Note 1: High instability of chromosome III

Industrial conditions offer a niche to interspecific hybrids due to the stressful conditions [1-7]. The existence of allotriploids and allotetraploids have generated speculation about rare-mating as one of the mechanisms for the generation of hybrids [8-10]. For rare-mating, one of the diploid cells must convert the heterozygous *MAT* locus into a homozygous state (*MAT***a**/*MAT***a** or *MAT*α/*MAT*α) or a hemizygous state (*MAT***a/-** or *MAT*α/-), such as after a double-strand break. Such mating-compatible diploids are then able to mate with a compatible cell of the opposite sex [11].

iHyPr exploits the rare-mating system by heterologously expressing the *HO* gene from differentially marked plasmids [12]. We expected that Ho would cut one copy of the heterozygous *MAT* locus of the diploid strain and use homology repair to convert the locus from heterozygous to homozygous, presumably over a small gene conversion patch using either a homologous chromosome or a silent mating cassette as a template. Although both this mechanism and larger breakage-induced replication events likely occur some of the time at this locus, 88.9% of the translocations involving chromosome III were unbalanced, suggesting other repair mechanisms are also leading to mating-type locus hemizygosity or homozygosity. For example, the high number of unbalanced translocations targeting chromosome III (40 % or 6/15 hybrids) might support the occurrence of imperfect non-homologous end joining (NHEJ) events, perhaps promoted by overexpression of the *HO* gene [11], or this chromosome might be inherently less stable [13]. Synthetic hybrids between *S. cerevisiae* and *S. kudriavzevii* have demonstrated how easily chromosome III of one of the parents can be lost, rendering the hybrid competent to mate again [14]. Recent studies of interspecific hybrids from the genus *Zygosaccharomyces* have also shown that inactivation of one of the *MAT* locus copies can also restore sexual competency [15,16]. Regardless of the precise mechanisms at work, the iHyPr method clearly facilitated the recovery of the sexual competency of higher-order hybrids by controlling and exploiting these naturally occurring mechanisms to generate interspecific hybrids.

Note 2: Heteroplasmic state

During interspecific hybridization, hybrids can inherit one of the two parental mitotypes or a recombinant version [17]. In general, one of the parental mitotypes was quickly fixed during the generation of our hybrids here, except for: the allotetraploid *S. kudriavzevii* x *S. mikatae* yHRWh4, the allotetraploid *S. cerevisiae* x *S. uvarum* yHRWh10, and the six-species hybrids yHRWh36, which were all heteroplasmic (Figure S4).

The loss of mitochondrial genomes in particular hybrid combinations, and the unusually high or low coverage (Figure S4) might suggest that interactions between nuclear-encoded mitochondrial proteins with the mtDNA are unbalanced. In such cases, one model proposes that an oligomeric circular mtDNA form precedes $\rho^-$ strain formation [18]. Although technical artifacts from Illumina sequencing cannot be excluded, the read coverages for some regions of the mtDNAs were surprisingly varied in some hybrids, such as yHRWh8, yHRWh13, and most of the hybrids in the yHRWh36 crossing scheme (Figure S4). Formation and subsequent mis-regulated of mtDNA concatemers by Din7p and Mhr1p [18] provide a possible model for how specific mitochondrial regions increase or decrease in copy numbers in hybrids, and this phenomenon merits further study.

## Supplementary figures

## Supplementary figure 1

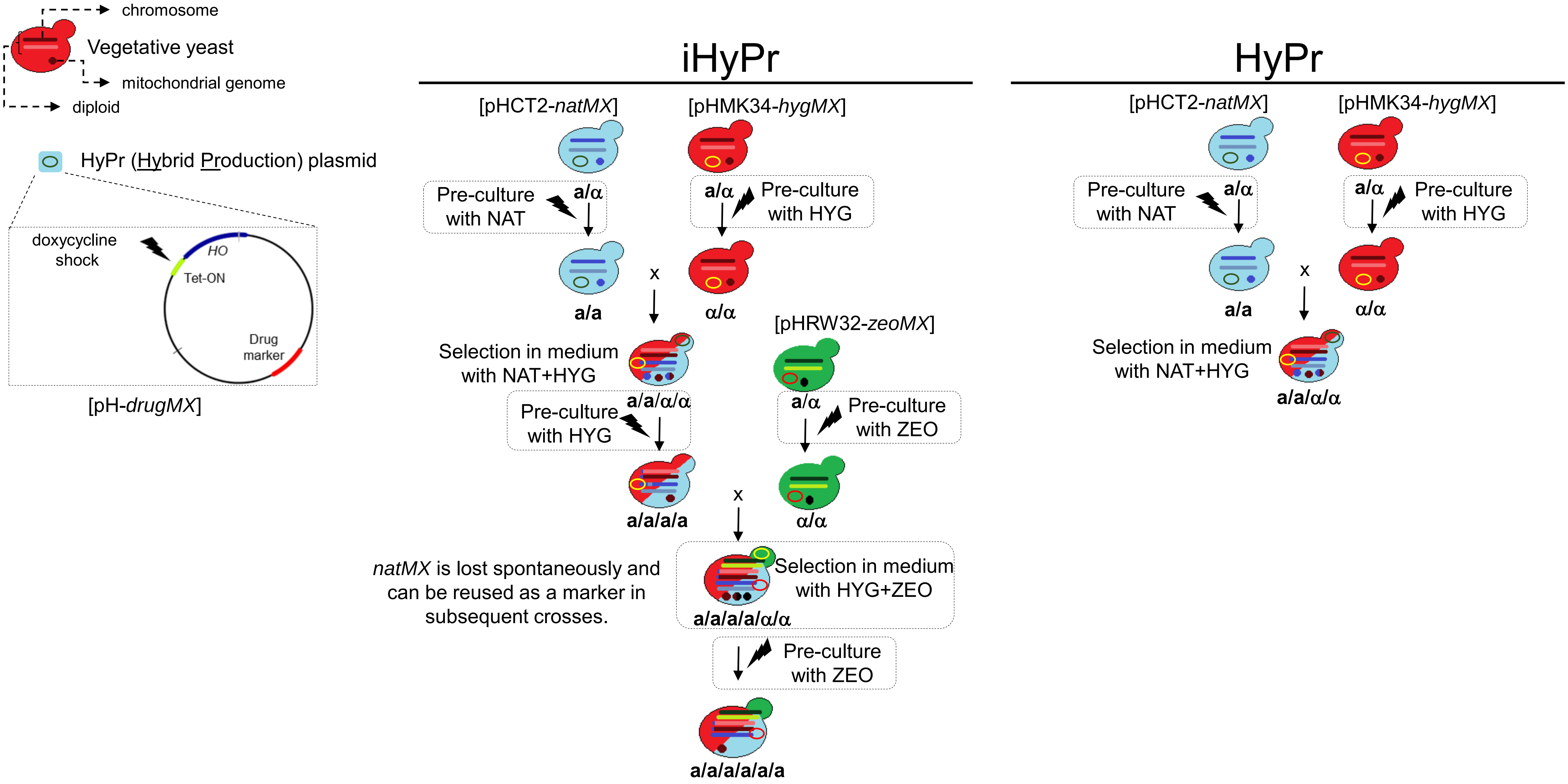

**Supplementary Figure 1 | The iHyPr method enabled the formation of higher-order synthetic hybrids using iterative crosses.** A simplified scheme comparing the protocol to generate an allohexaploid (6n) synthetic hybrid using iHyPr is displayed, in contrast with HyPr, which is not iterative. NAT, Nourseothricin; HYG, hygromycin; ZEO, zeocin. *MAT* idiomorphs examples are shortened to **a** and $\alpha$.

Supplementary figure 2

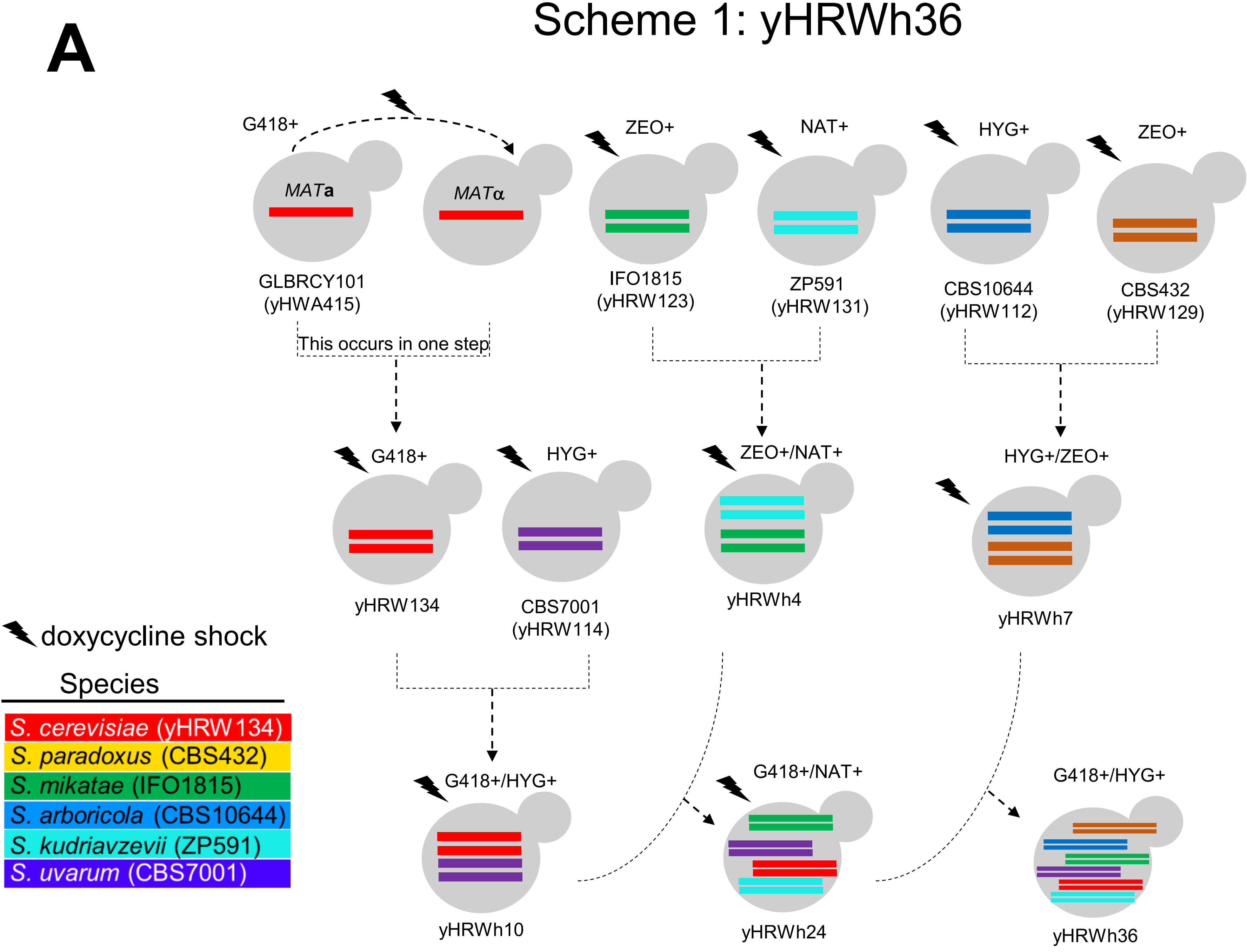

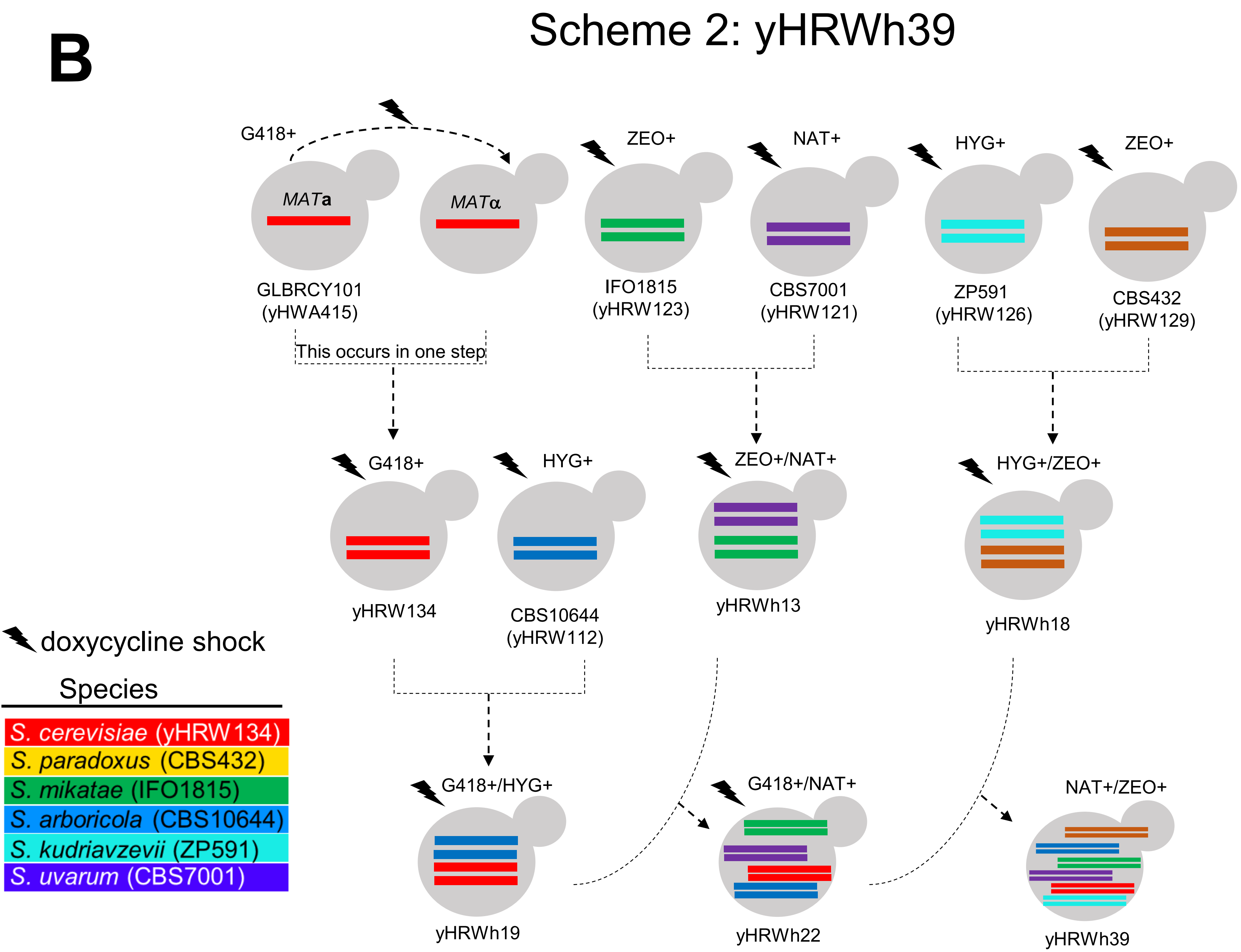

B
Scheme 2: yHRWh39
G418+
MATa
MATα
GLBRCY101
(yHWA415)
This occurs in one step
ZEO+
IFO1815
(yHRW123)
NAT+
CBS7001
(yHRW121)
HYG+
ZP591
(yHRW126)
ZEO+
CBS432
(yHRW129)
G418+
yHRW134
HYG+
CBS10644
(yHRW112)
ZEO+/NAT+
yHRWh13
HYG+/ZEO+
yHRWh18
doxycycline shock
Species
S. cerevisiae (yHRW134)
S. paradoxus (CBS432)
S. mikatae (IFO1815)
S. arboricola (CBS10644)
S. kudriavzevii (ZP591)
S. uvarum (CBS7001)
G418+/HYG+
yHRWh19
G418+/NAT+
yHRWh22
NAT+/ZEO+
yHRWh39

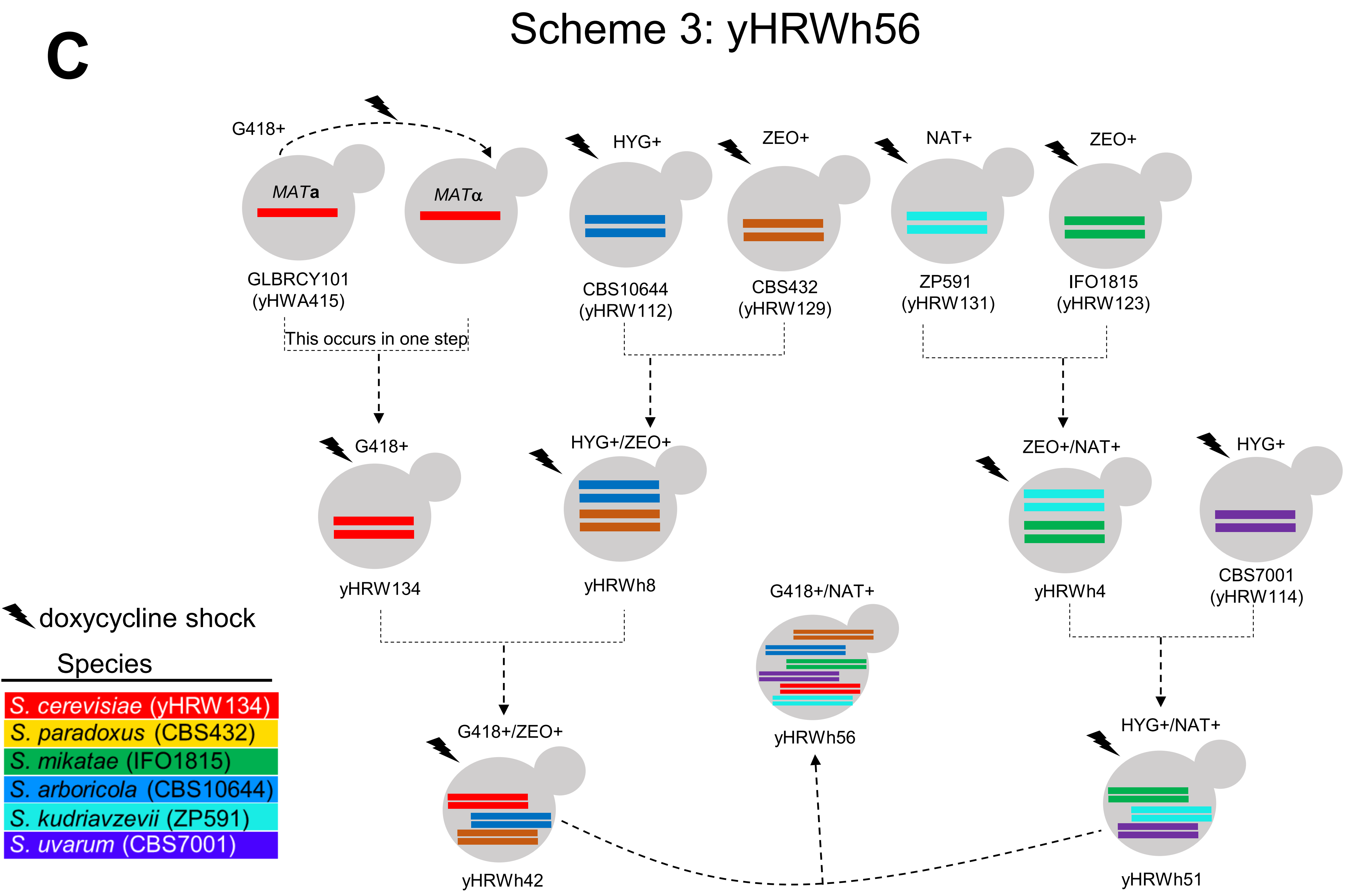

C
Scheme 3: yHRWh56
G418+
MATa
MATα
GLBRCY101
(yHWA415)
This occurs in one step
HYG+
CBS10644
(yHRW112)
ZEO+
CBS432
(yHRW129)
NAT+
ZP591
(yHRW131)
ZEO+
IFO1815
(yHRW123)
G418+
yHRW134
HYG+/ZEO+
yHRWh8
ZEO+/NAT+
yHRWh4
HYG+
CBS7001
(yHRW114)
G418+/NAT+
yHRWh56
G418+/ZEO+
yHRWh42
HYG+/NAT+
yHRWh51
doxycycline shock
Species
S. cerevisiae (yHRW134)
S. paradoxus (CBS432)
S. mikatae (IFO1815)
S. arboricola (CBS10644)
S. kudriavzevii (ZP591)
S. uvarum (CBS7001)

**Supplementary Figure 2 | Schematics for the generation of three six-species *Saccharomyces* hybrids.** The hybridization steps necessary to generate the six-species hybrids yHRWh36, yHRWh39, and yHRWh56 are represented in panels A), B) and C), respectively. Yeast cells are represented in gray, and chromosomes are colored according to the *Saccharomyces* species. The strain names of our lab's copy of some strains (Supplementary Data 1) are displayed in parentheses below the original culture collection strains. Drug resistance is indicated above yeast cells according to the abbreviations in Supplementary Figure 1. Systematic crosses are highlighted with arrows to form a pedigree. The black lightning bolt symbol represents the doxycycline shock to promote mating type switching or loss to facilitate hybridization.

**Supplementary figure 3**

**A**

yHRW134 (*Scer* x *Scer*)

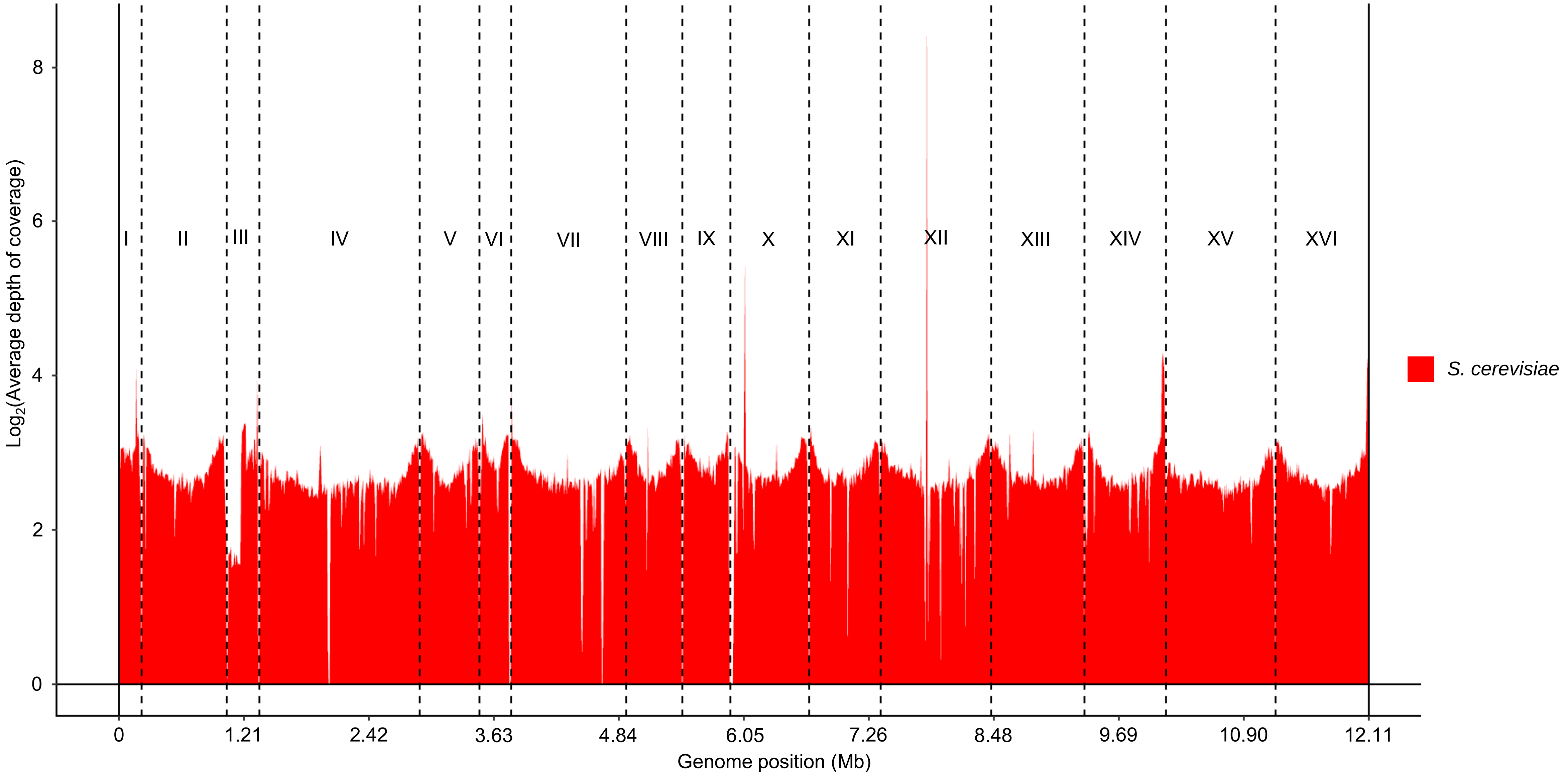

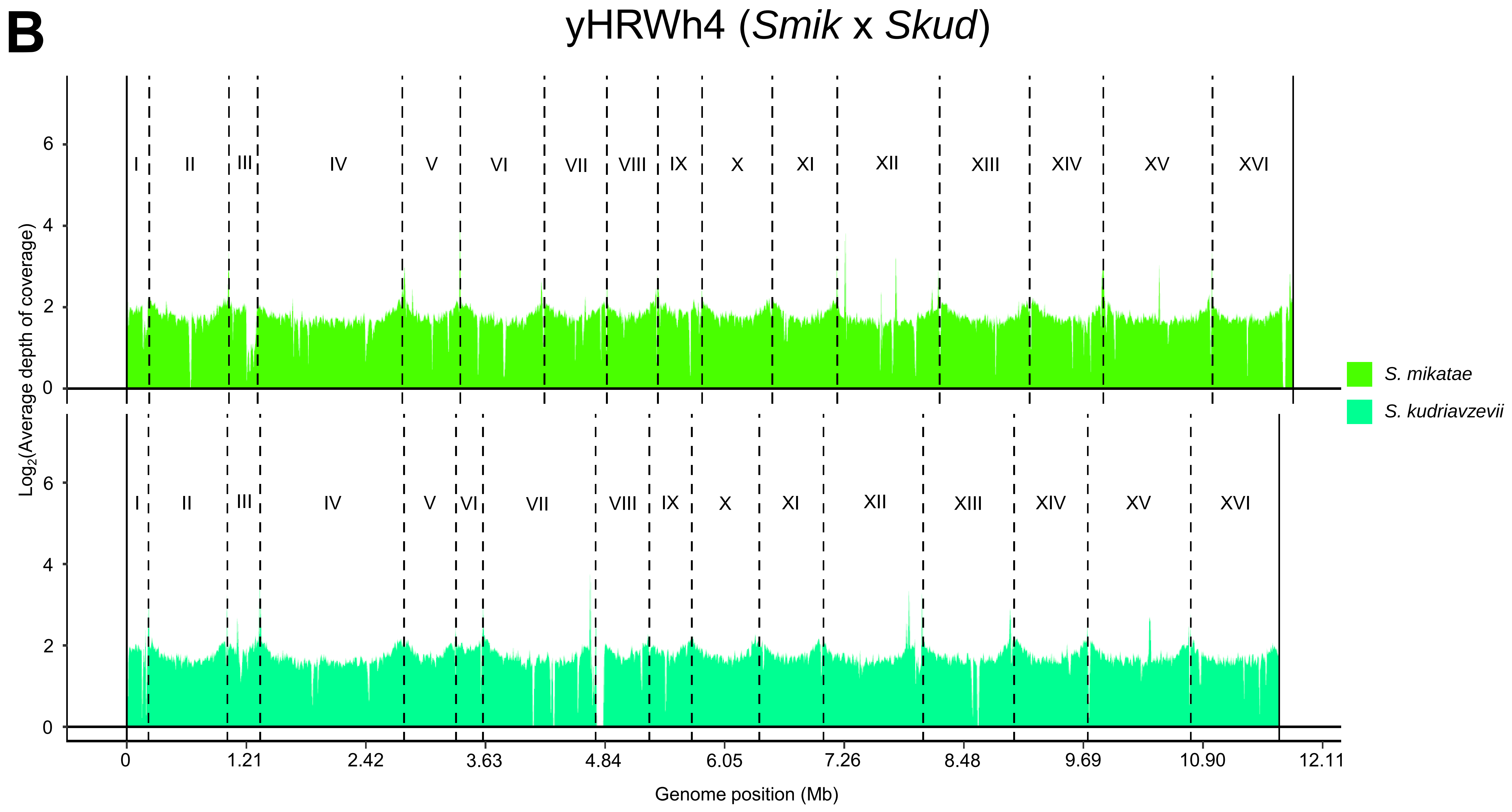
B
yHRWh4 (Smik x Skud)
Log2(Average depth of coverage)
0
2
4
6
I
II
III
IV
V
VI
VII
VIII
IX
X
XI
XII
XIII
XIV
XV
XVI
0
1.21
2.42
3.63
4.84
6.05
7.26
8.48
9.69
10.90
12.11
Genome position (Mb)
S. mikatae
S. kudriavzevii

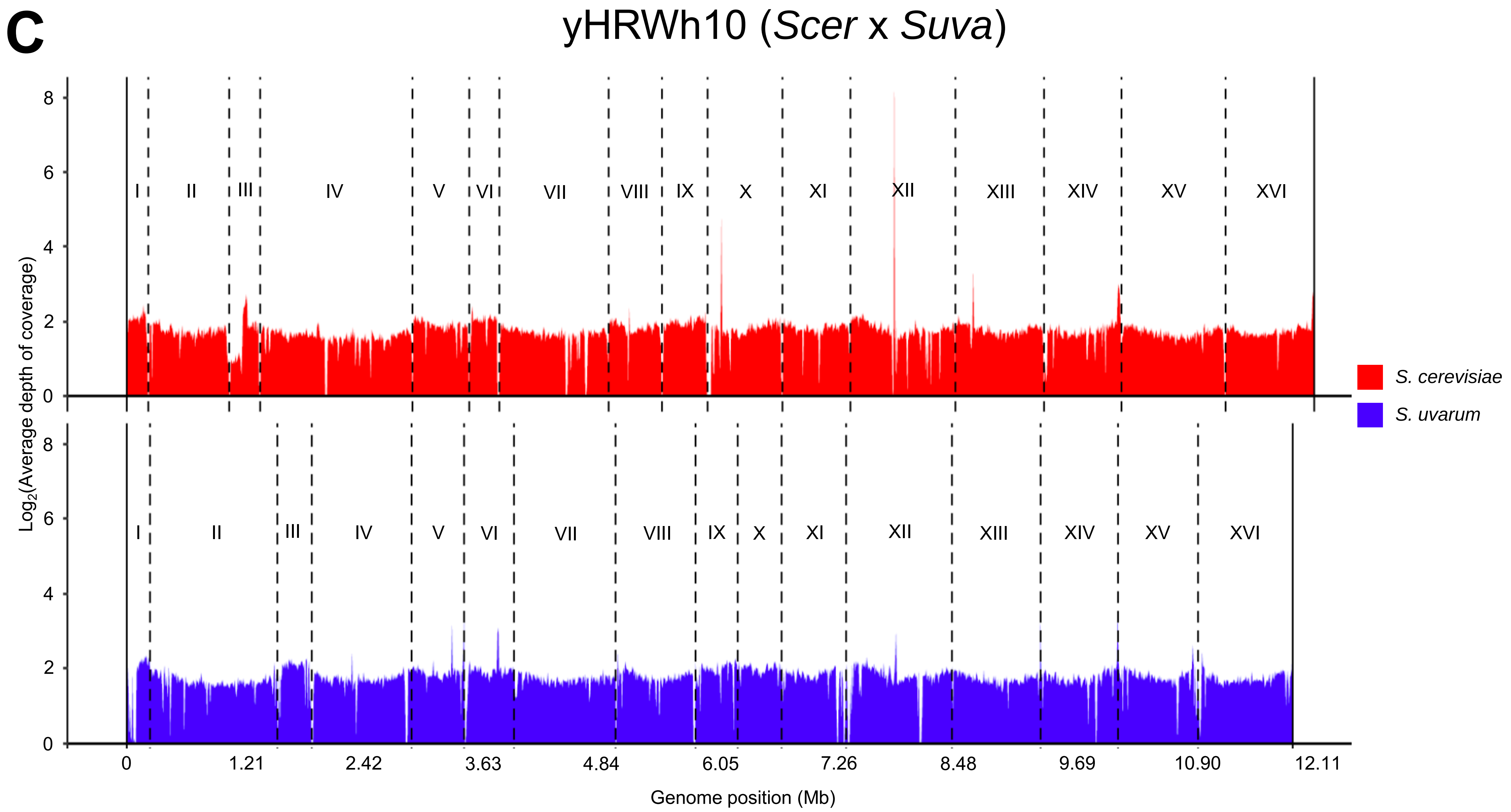
C
yHRWh10 (*Scer* x *Suva*)
Log2(Average depth of coverage)
8
6
4
2
0
I
II
III
IV
V
VI
VII
VIII
IX
X
XI
XII
XIII
XIV
XV
XVI
0
1.21
2.42
3.63
4.84
6.05
7.26
8.48
9.69
10.90
12.11
Genome position (Mb)
S. cerevisiae
S. uvarum

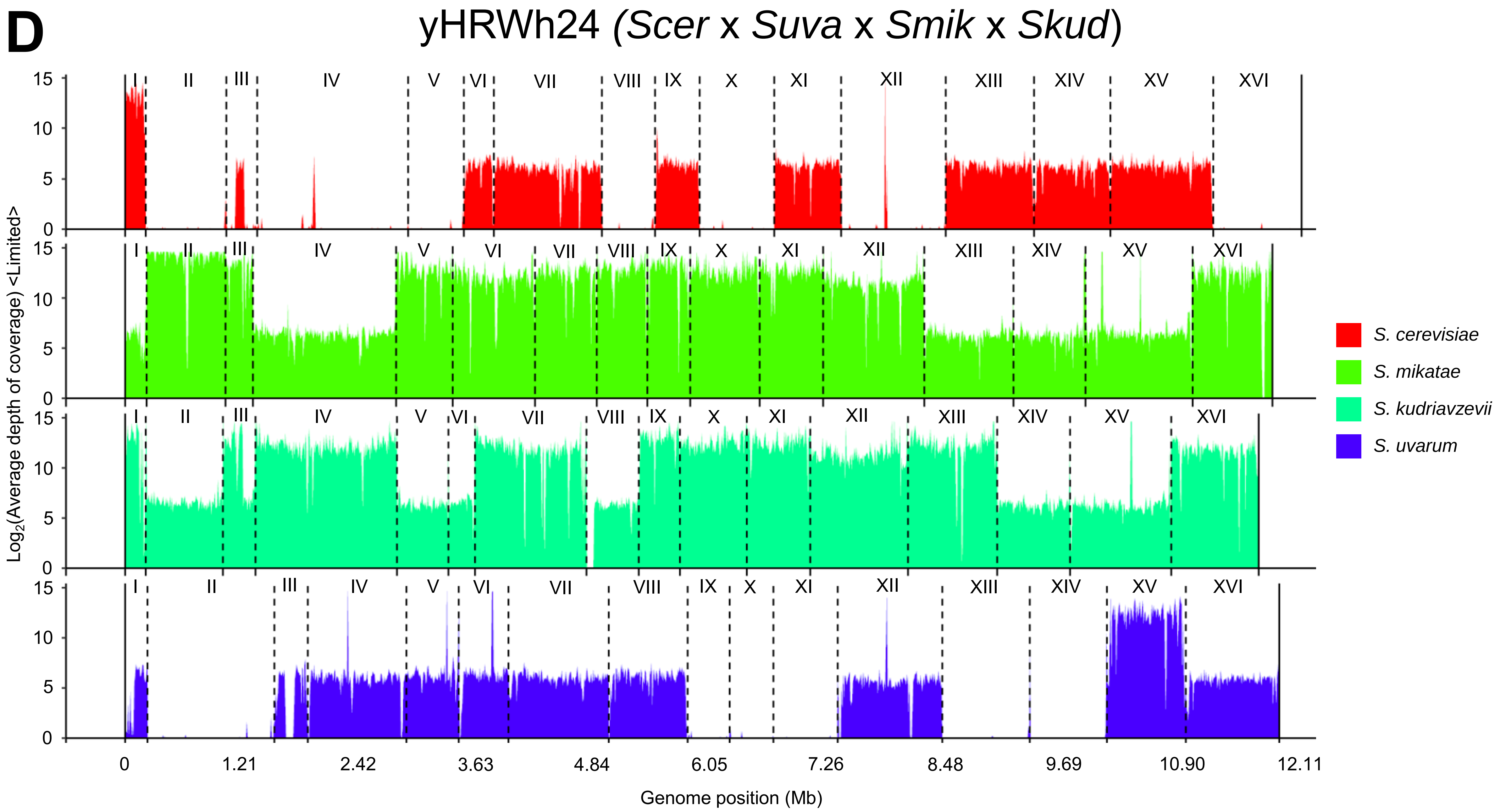

D
yHRWh24 (*Scer* x *Suva* x *Smik* x *Skud*)
I
II
III
IV
V
VI
VII
VIII
IX
X
XI
XII
XIII
XIV
XV
XVI
Log2(Average depth of coverage) <Limited>
0
5
10
15
0
1.21
2.42
3.63
4.84
6.05
7.26
8.48
9.69
10.90
12.11
Genome position (Mb)
S. cerevisiae
S. mikatae
S. kudriavzevii
S. uvarum

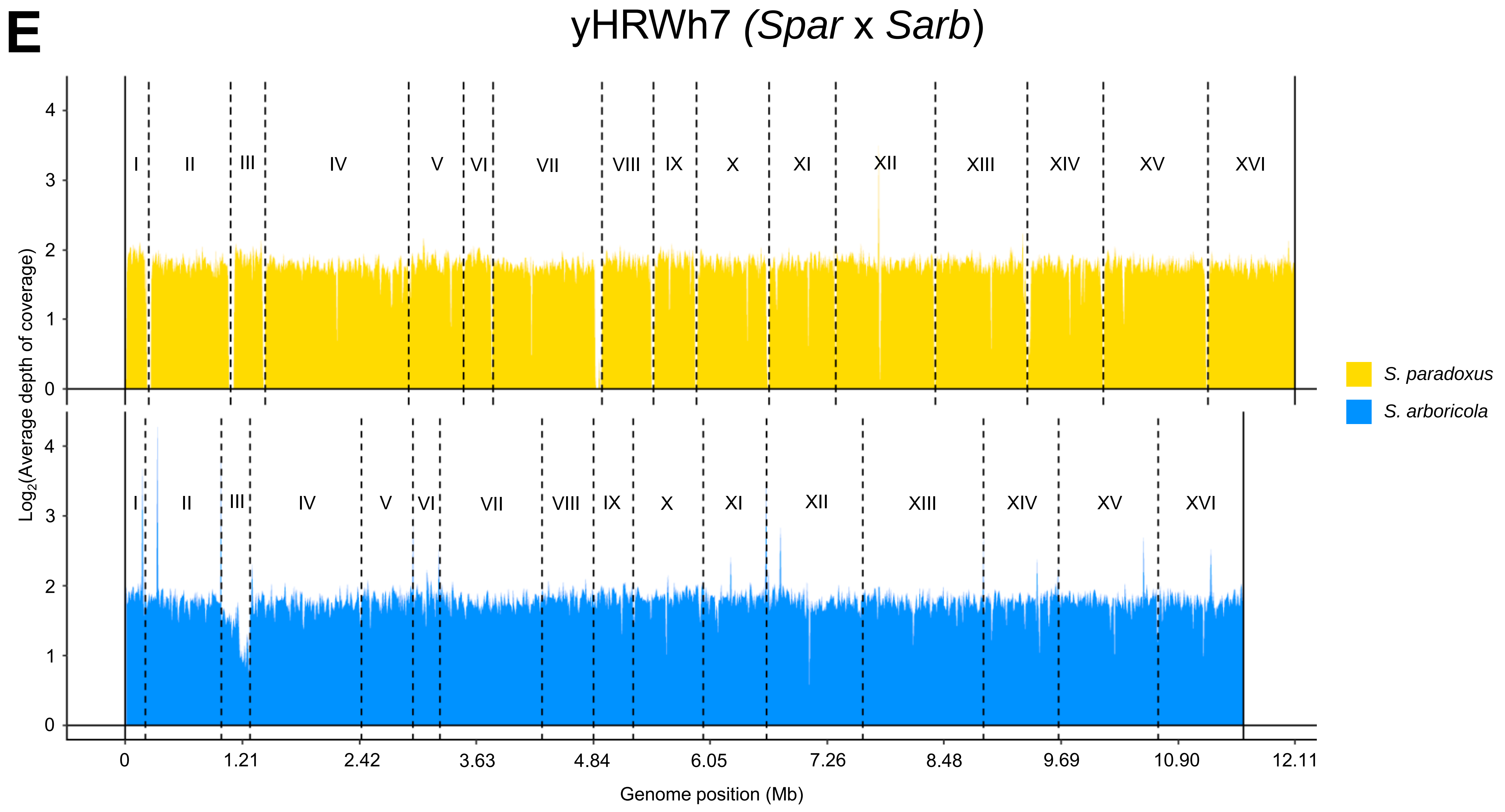
E
yHRWh7 (*Spar* x *Sarb*)
I
II
III
IV
V
VI
VII
VIII
IX
X
XI
XII
XIII
XIV
XV
XVI
Log2(Average depth of coverage)
0
1
2
3
4
0
1.21
2.42
3.63
4.84
6.05
7.26
8.48
9.69
10.90
12.11
Genome position (Mb)
S. paradoxus
S. arboricola

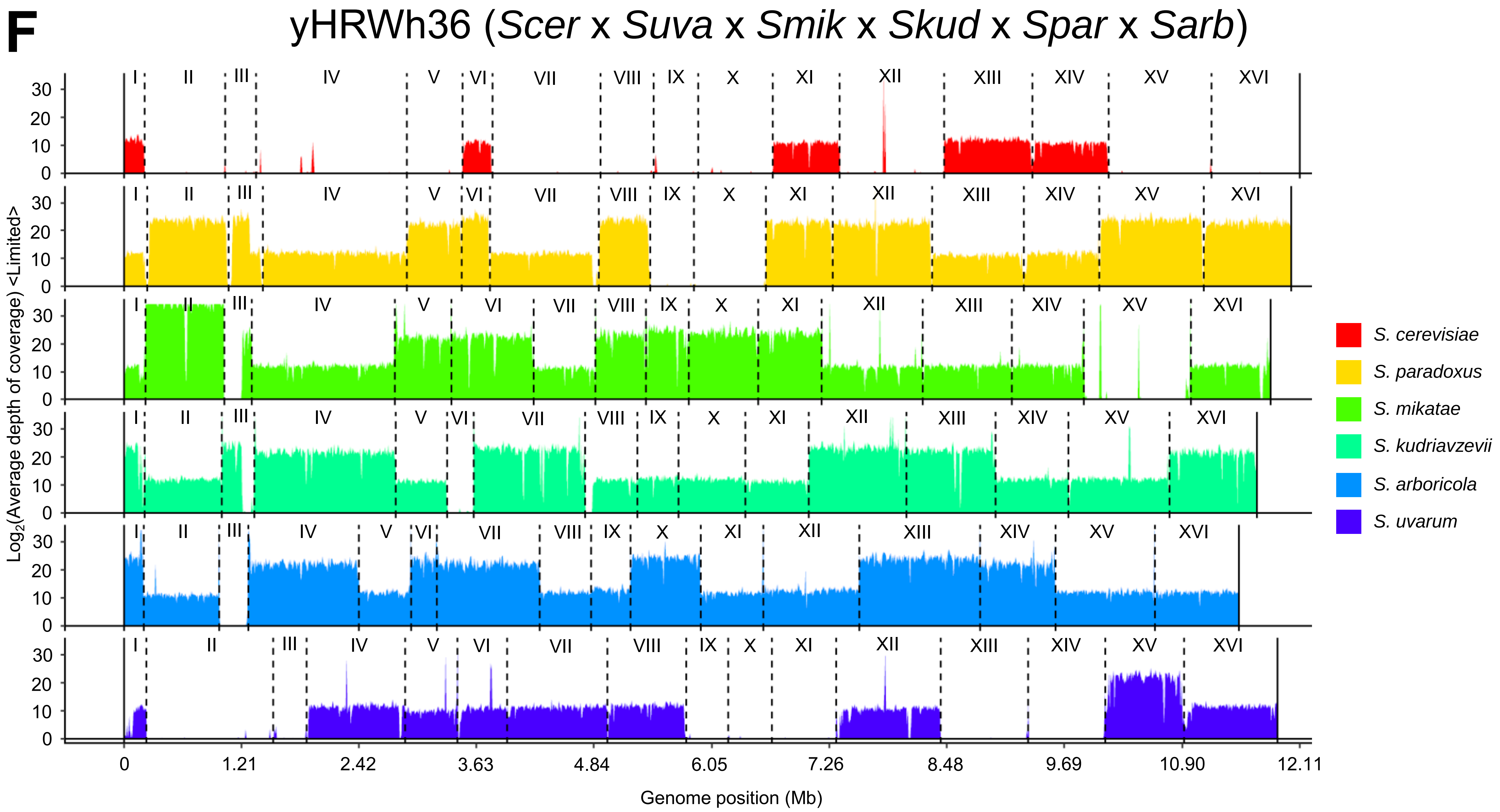
F
yHRWh36 (Scer x Suva x Smik x Skud x Spar x Sarb)
I II III IV V VI VII VIII IX X XI XII XIII XIV XV XVI
30
20
10
0
Log2(Average depth of coverage) <Limited>
0
1.21
2.42
3.63
4.84
6.05
7.26
8.48
9.69
10.90
12.11
Genome position (Mb)
S. cerevisiae
S. paradoxus
S. mikatae
S. kudriavzevii
S. arboricola
S. uvarum

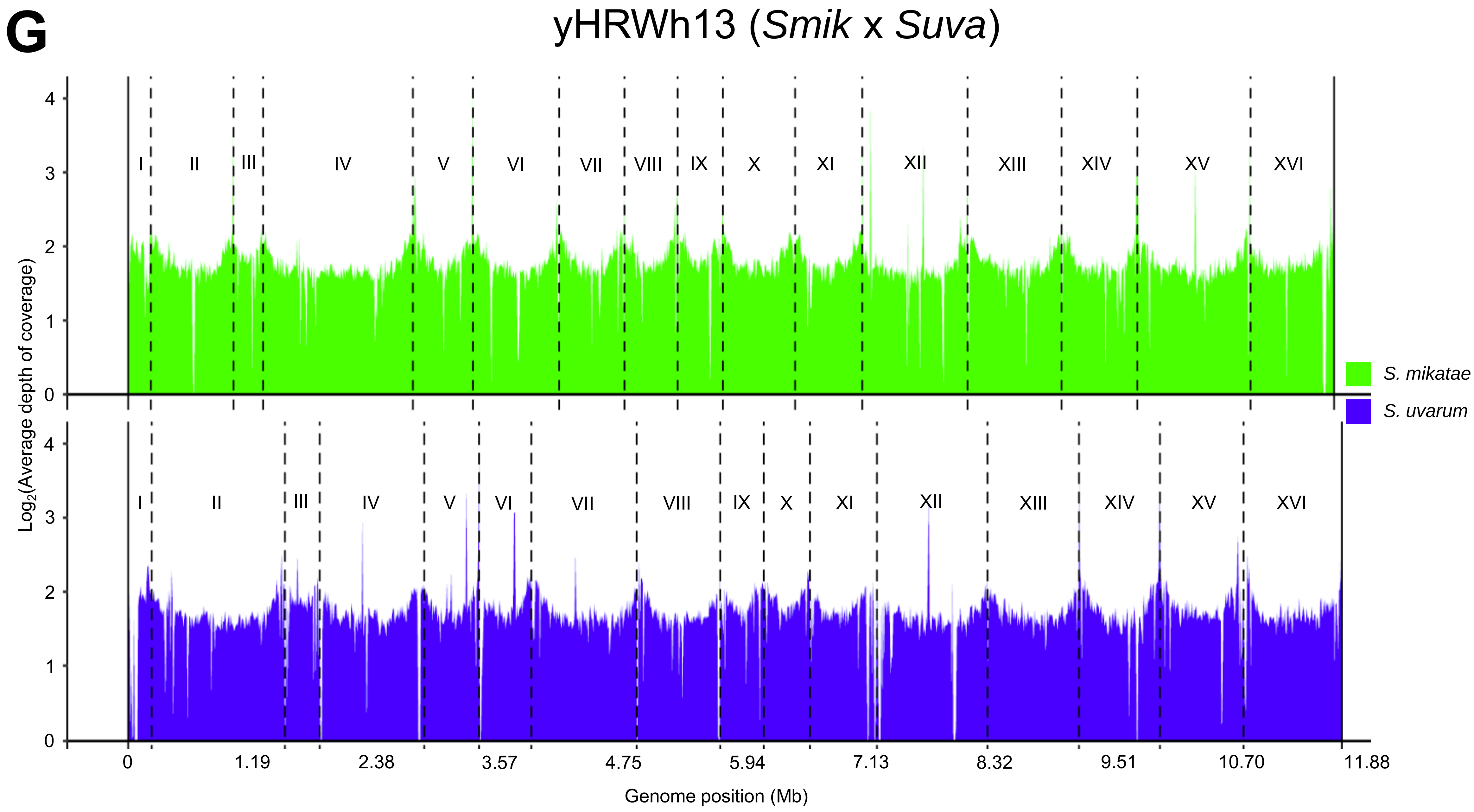

G
yHRWh13 (*Smik* x *Suva*)
I
II
III
IV
V
VI
VII
VIII
IX
X
XI
XII
XIII
XIV
XV
XVI
S. mikatae
S. uvarum
Log2(Average depth of coverage)
0
1
2
3
4
0
1.19
2.38
3.57
4.75
5.94
7.13
8.32
9.51
10.70
11.88
Genome position (Mb)

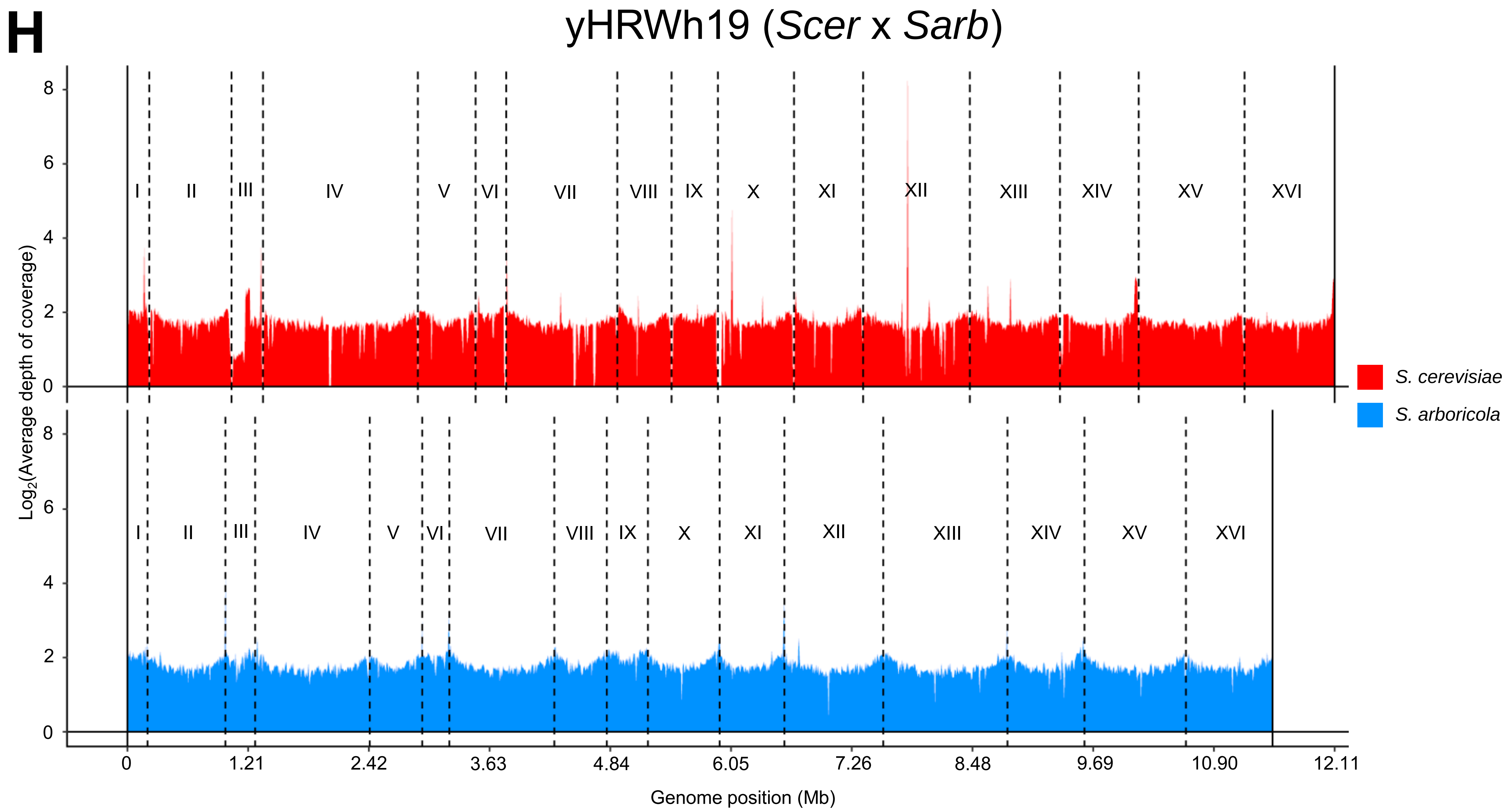
H
yHRWh19 (*Scer* x *Sarb*)
Log2(Average depth of coverage)
0
2
4
6
8
I
II
III
IV
V
VI
VII
VIII
IX
X
XI
XII
XIII
XIV
XV
XVI
0
1.21
2.42
3.63
4.84
6.05
7.26
8.48
9.69
10.90
12.11
Genome position (Mb)
*S. cerevisiae*
*S. arboricola*

**I**

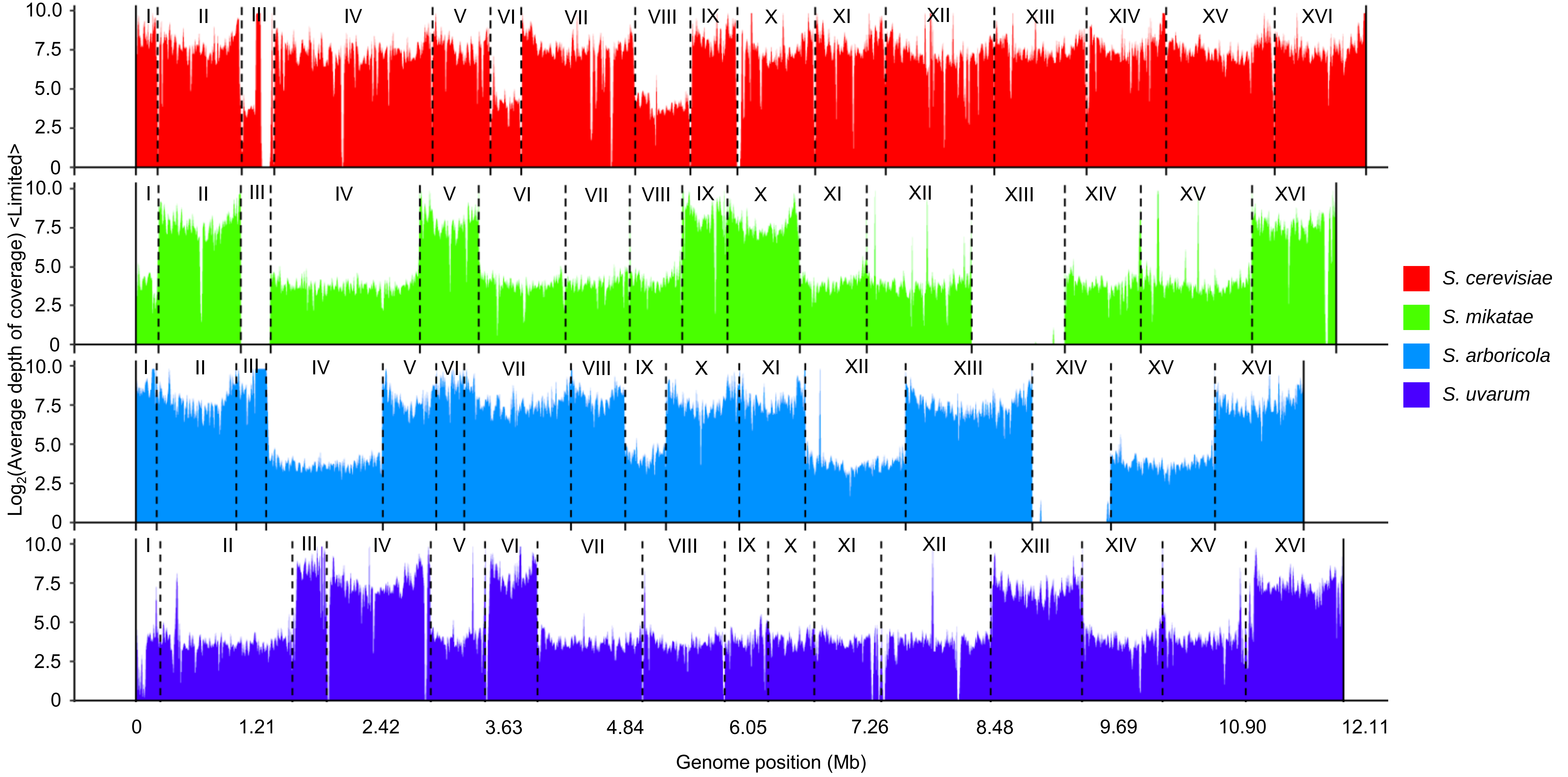

yHRWh22 (*Scer* x *Sarb* x *Smik* x *Suva*)
Log2(Average depth of coverage) <Limited>
Genome position (Mb)
0
1.21
2.42
3.63
4.84
6.05
7.26
8.48
9.69
10.90
12.11
10.0
7.5
5.0
2.5
0
I II III IV V VI VII VIII IX X XI XII XIII XIV XV XVI
*S. cerevisiae*
*S. mikatae*
*S. arboricola*
*S. uvarum*

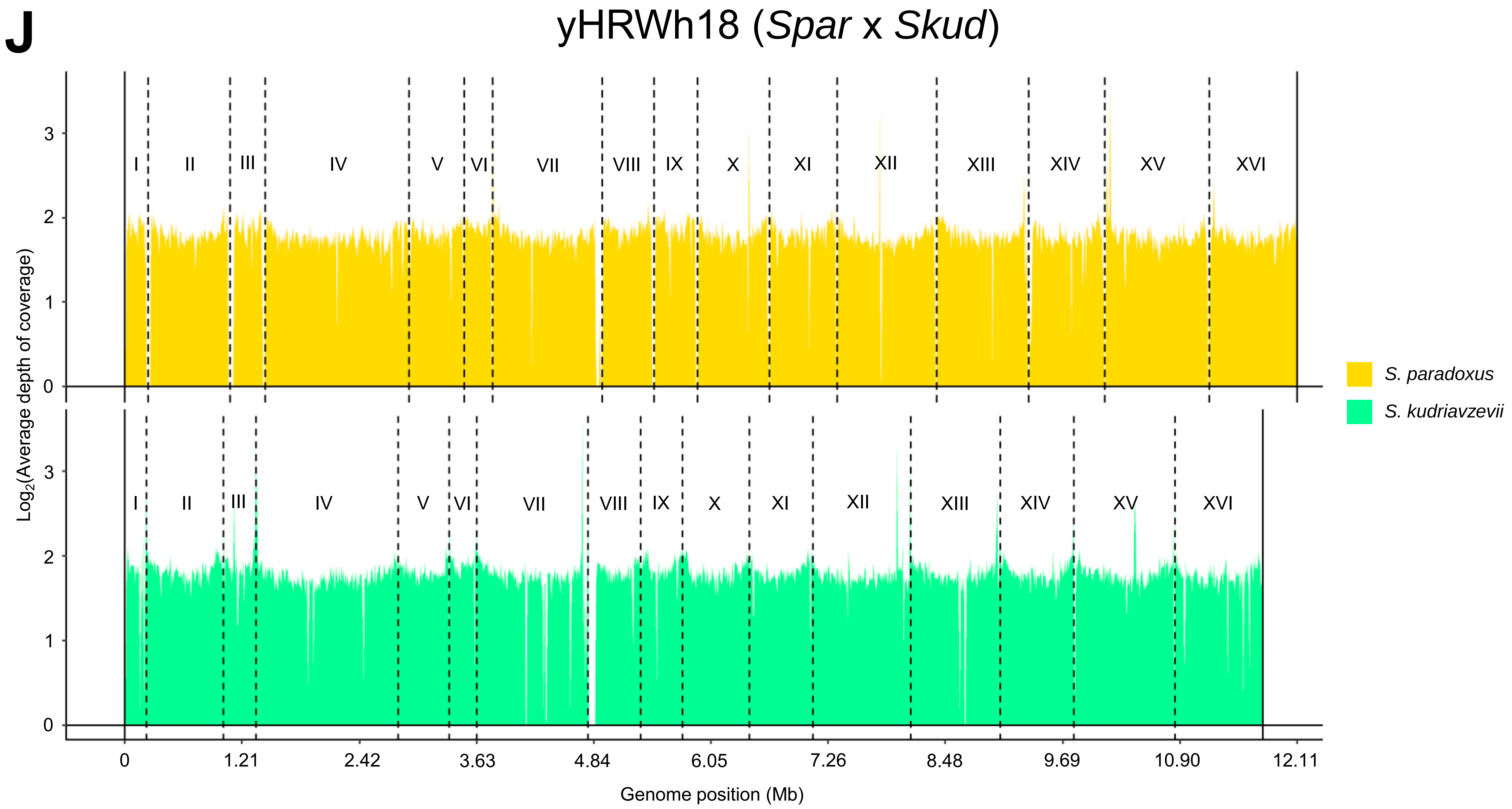

J
yHRWh18 (*Spar* x *Skud*)
Log2(Average depth of coverage)
0
1
2
3
I
II
III
IV
V
VI
VII
VIII
IX
X
XI
XII
XIII
XIV
XV
XVI
0
1.21
2.42
3.63
4.84
6.05
7.26
8.48
9.69
10.90
12.11
Genome position (Mb)
*S. paradoxus*
*S. kudriavzevii*

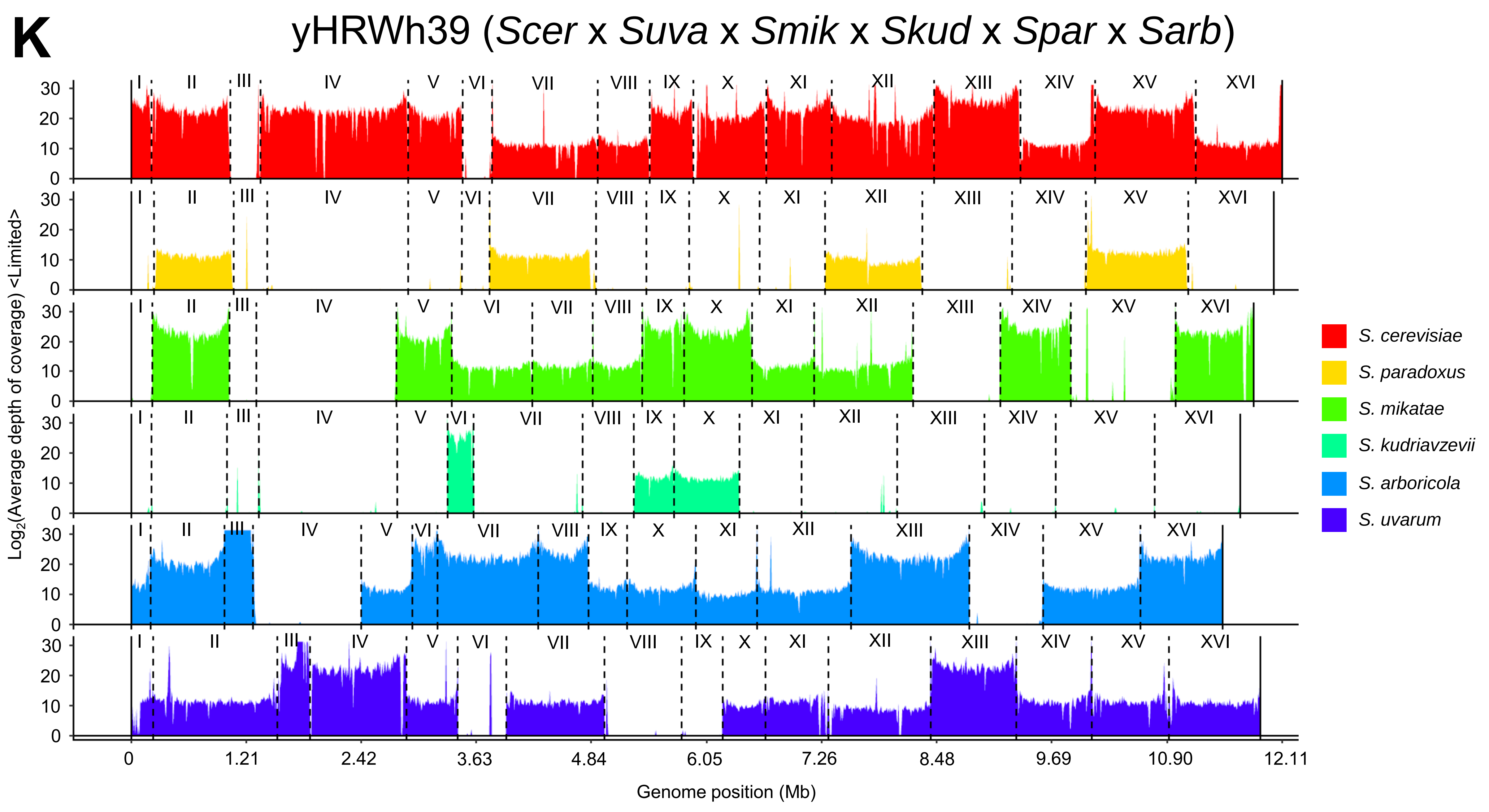

K
yHRWh39 (Scer x Suva x Smik x Skud x Spar x Sarb)
I II III IV V VI VII VIII IX X XI XII XIII XIV XV XVI
Log2(Average depth of coverage) <Limited>
0 10 20 30
S. cerevisiae
S. paradoxus
S. mikatae
S. kudriavzevii
S. arboricola
S. uvarum
0 1.21 2.42 3.63 4.84 6.05 7.26 8.48 9.69 10.90 12.11
Genome position (Mb)

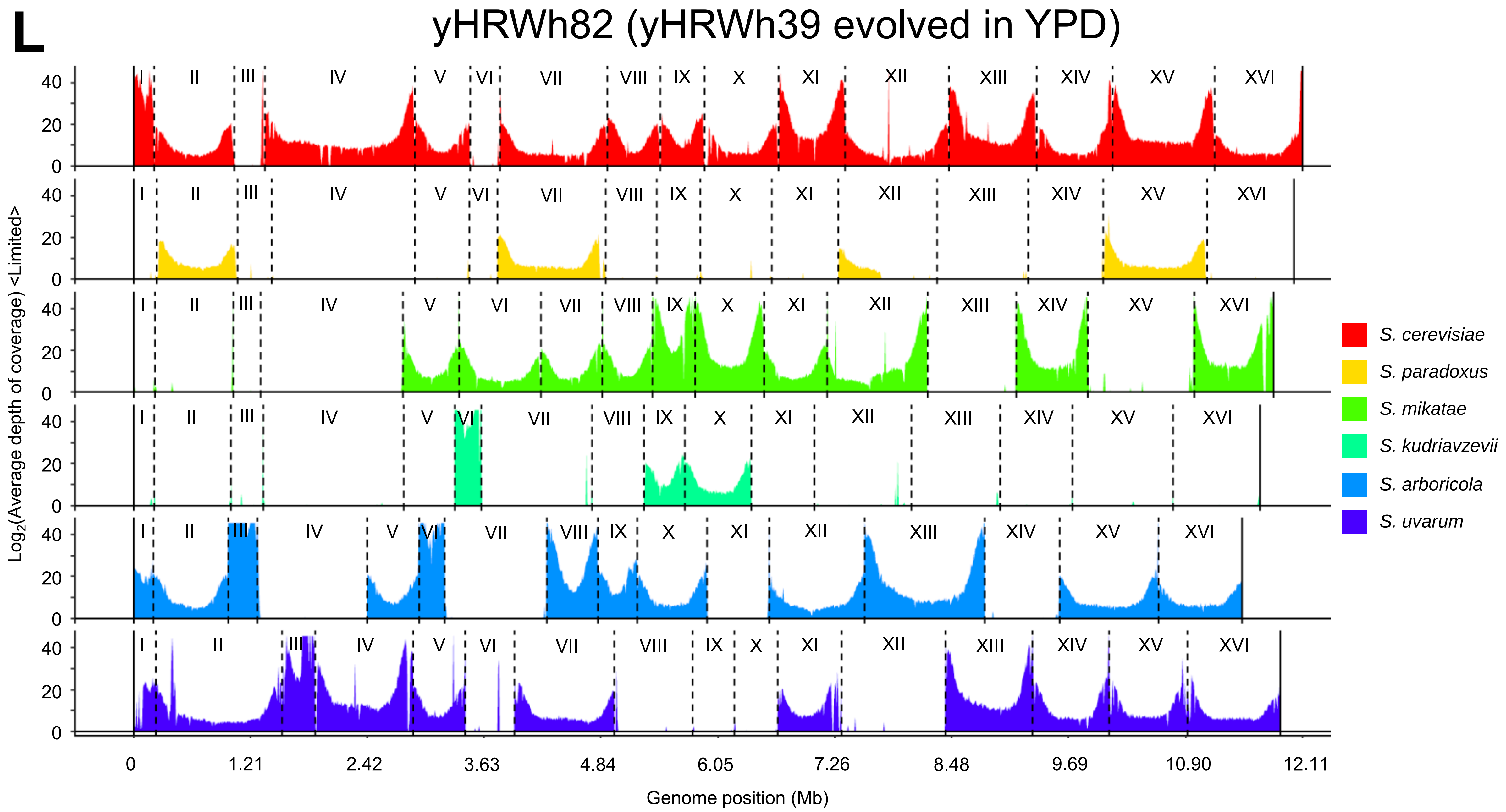
L
yHRWh82 (yHRWh39 evolved in YPD)
I
II
III
IV
V
VI
VII
VIII
IX
X
XI
XII
XIII
XIV
XV
XVI
0
20
40
Log2(Average depth of coverage) <Limited>
0
1.21
2.42
3.63
4.84
6.05
7.26
8.48
9.69
10.90
12.11
Genome position (Mb)
S. cerevisiae
S. paradoxus
S. mikatae
S. kudriavzevii
S. arboricola
S. uvarum

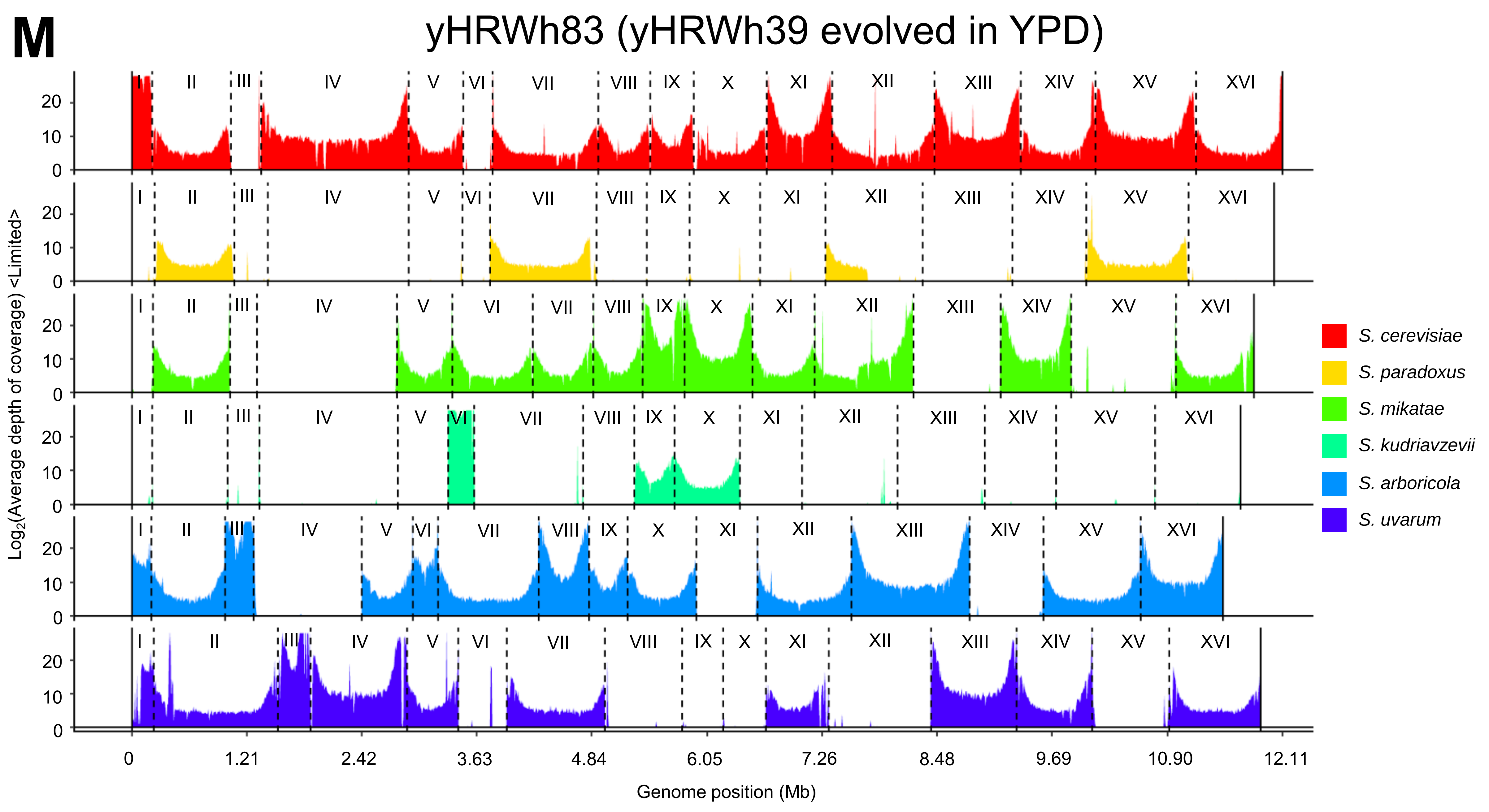
M
yHRWh83 (yHRWh39 evolved in YPD)
Log2(Average depth of coverage) <Limited>
Genome position (Mb)
0
1.21
2.42
3.63
4.84
6.05
7.26
8.48
9.69
10.90
12.11
I II III IV V VI VII VIII IX X XI XII XIII XIV XV XVI
S. cerevisiae
S. paradoxus
S. mikatae
S. kudriavzevii
S. arboricola
S. uvarum

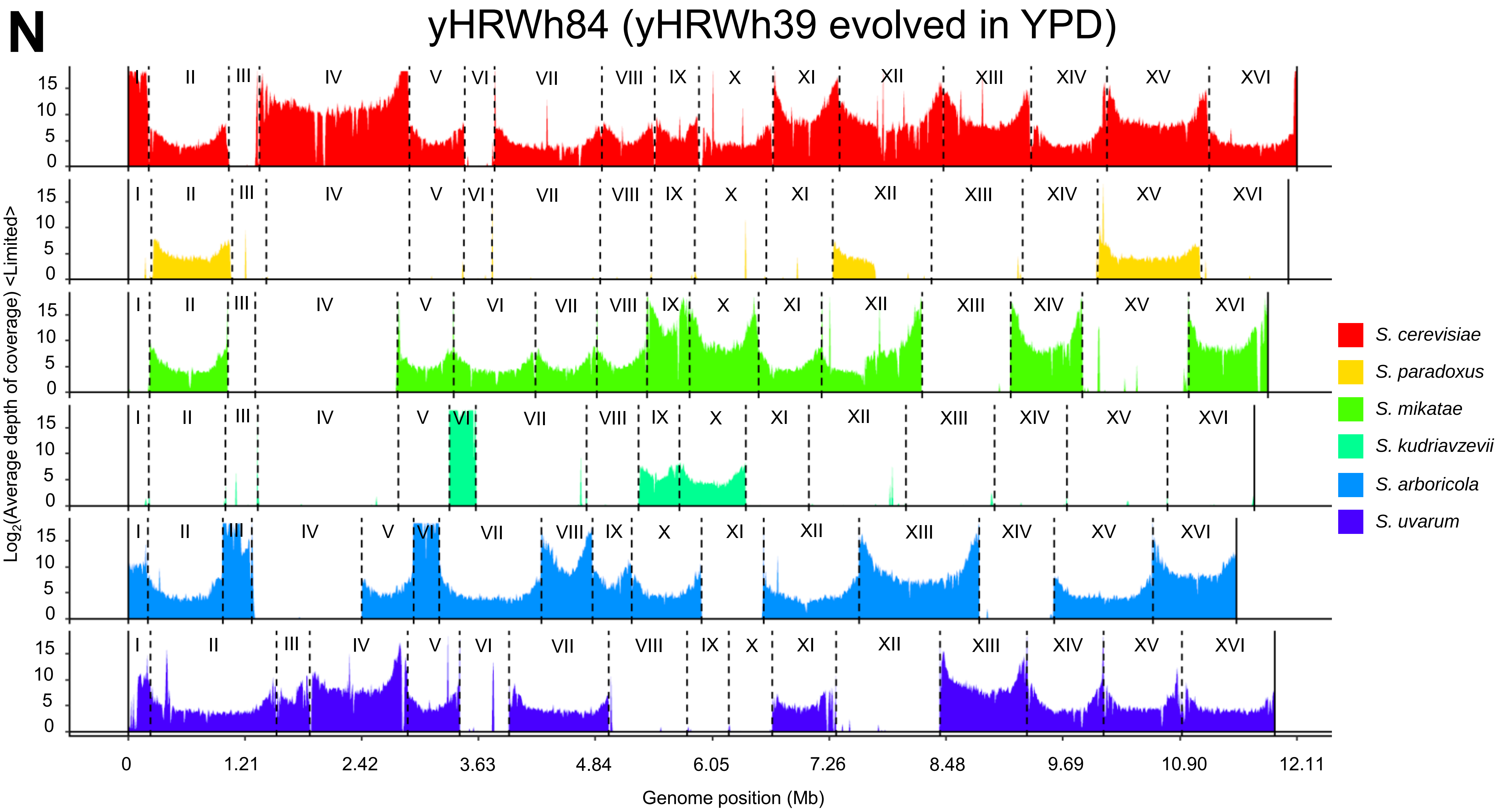
N
yHRWh84 (yHRWh39 evolved in YPD)
Log2(Average depth of coverage) <Limited>
I II III IV V VI VII VIII IX X XI XII XIII XIV XV XVI
15 10 5 0
S. cerevisiae
S. paradoxus
S. mikatae
S. kudriavzevii
S. arboricola
S. uvarum
0 1.21 2.42 3.63 4.84 6.05 7.26 8.48 9.69 10.90 12.11
Genome position (Mb)

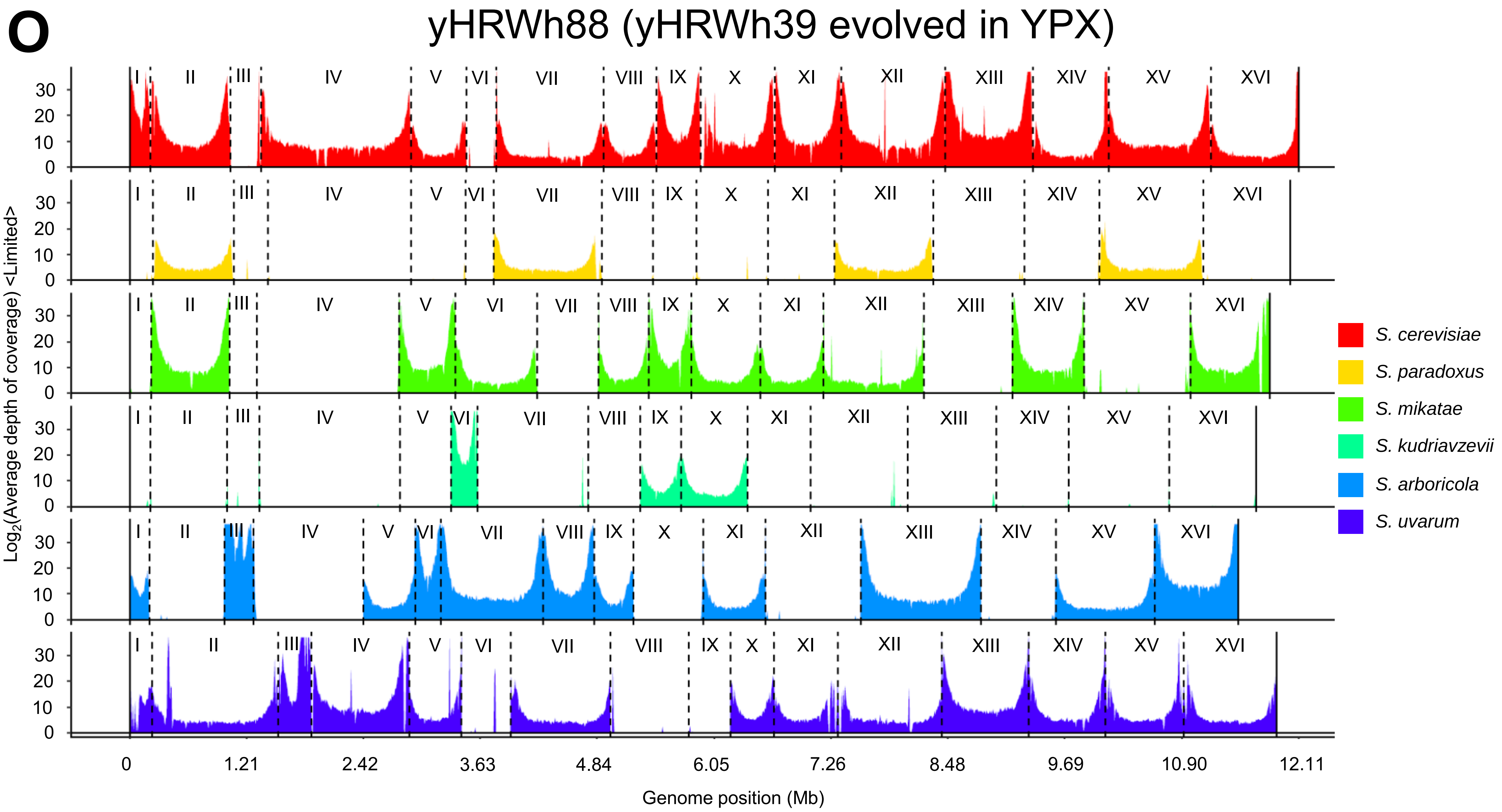
O
yHRWh88 (yHRWh39 evolved in YPX)
I
II
III
IV
V
VI
VII
VIII
IX
X
XI
XII
XIII
XIV
XV
XVI
30
20
10
0
Log2(Average depth of coverage) <Limited>
S. cerevisiae
S. paradoxus
S. mikatae
S. kudriavzevii
S. arboricola
S. uvarum
0
1.21
2.42
3.63
4.84
6.05
7.26
8.48
9.69
10.90
12.11
Genome position (Mb)

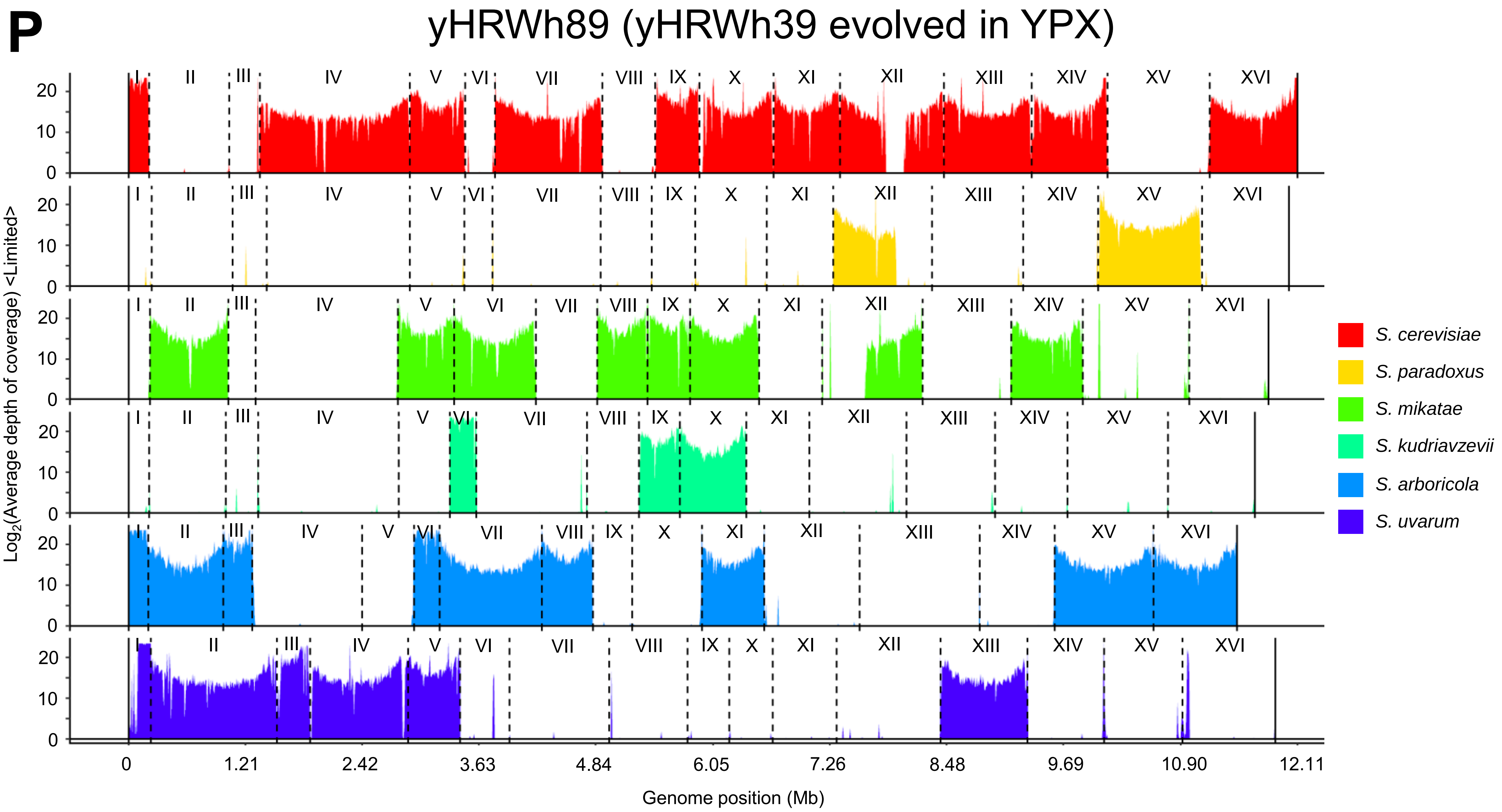
P
yHRWh89 (yHRWh39 evolved in YPX)
Log2(Average depth of coverage) <Limited>
S. cerevisiae
S. paradoxus
S. mikatae
S. kudriavzevii
S. arboricola
S. uvarum
0
1.21
2.42
3.63
4.84
6.05
7.26
8.48
9.69
10.90
12.11
Genome position (Mb)

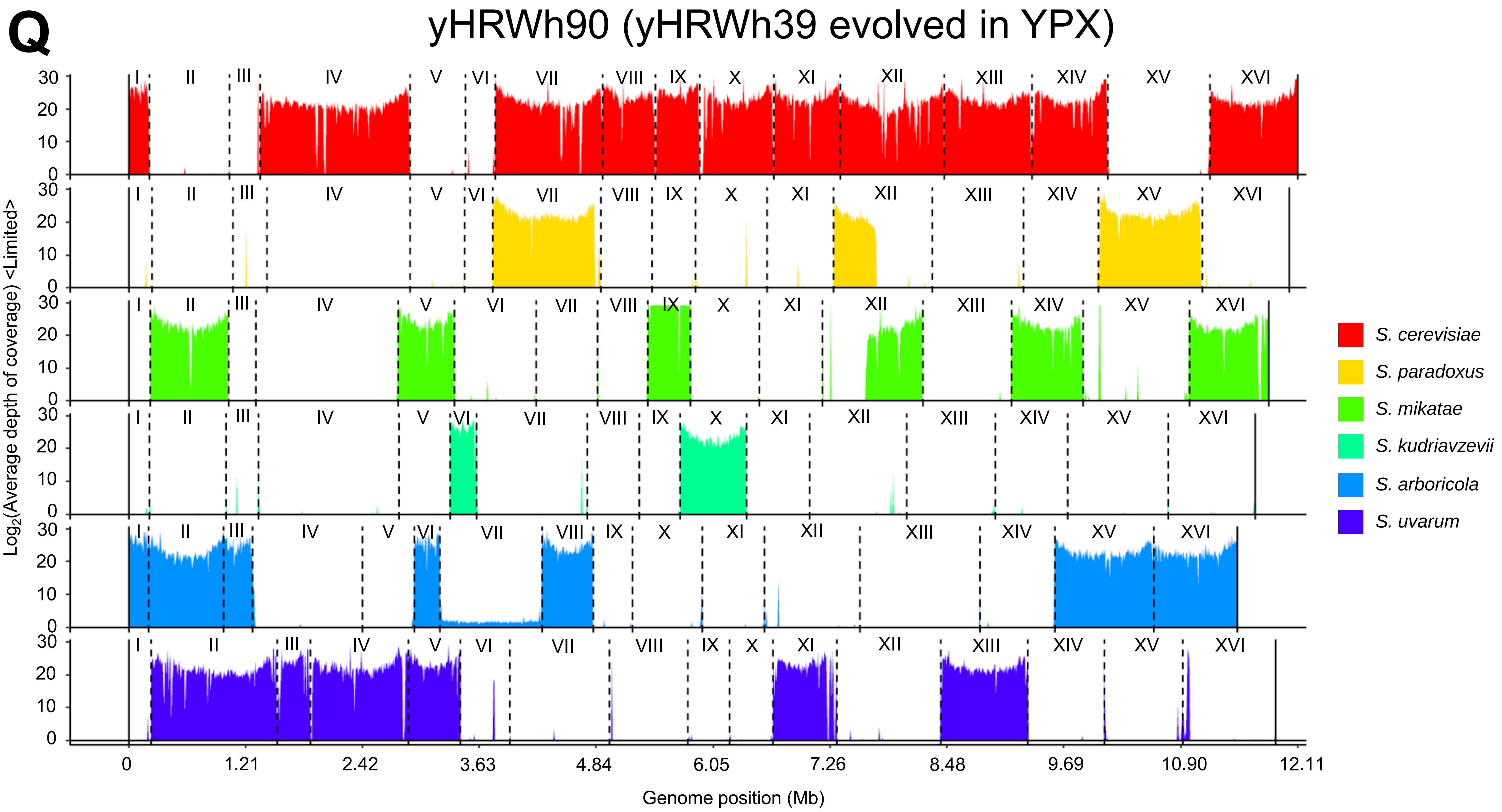
Q
yHRWh90 (yHRWh39 evolved in YPX)
Log$_2$(Average depth of coverage) <Limited>
Genome position (Mb)
0
1.21
2.42
3.63
4.84
6.05
7.26
8.48
9.69
10.90
12.11
I II III IV V VI VII VIII IX X XI XII XIII XIV XV XVI
S. cerevisiae
S. paradoxus
S. mikatae
S. kudriavzevii
S. arboricola
S. uvarum

R

yHRWh51 (*Smik* x *Skud* x *Suva*)

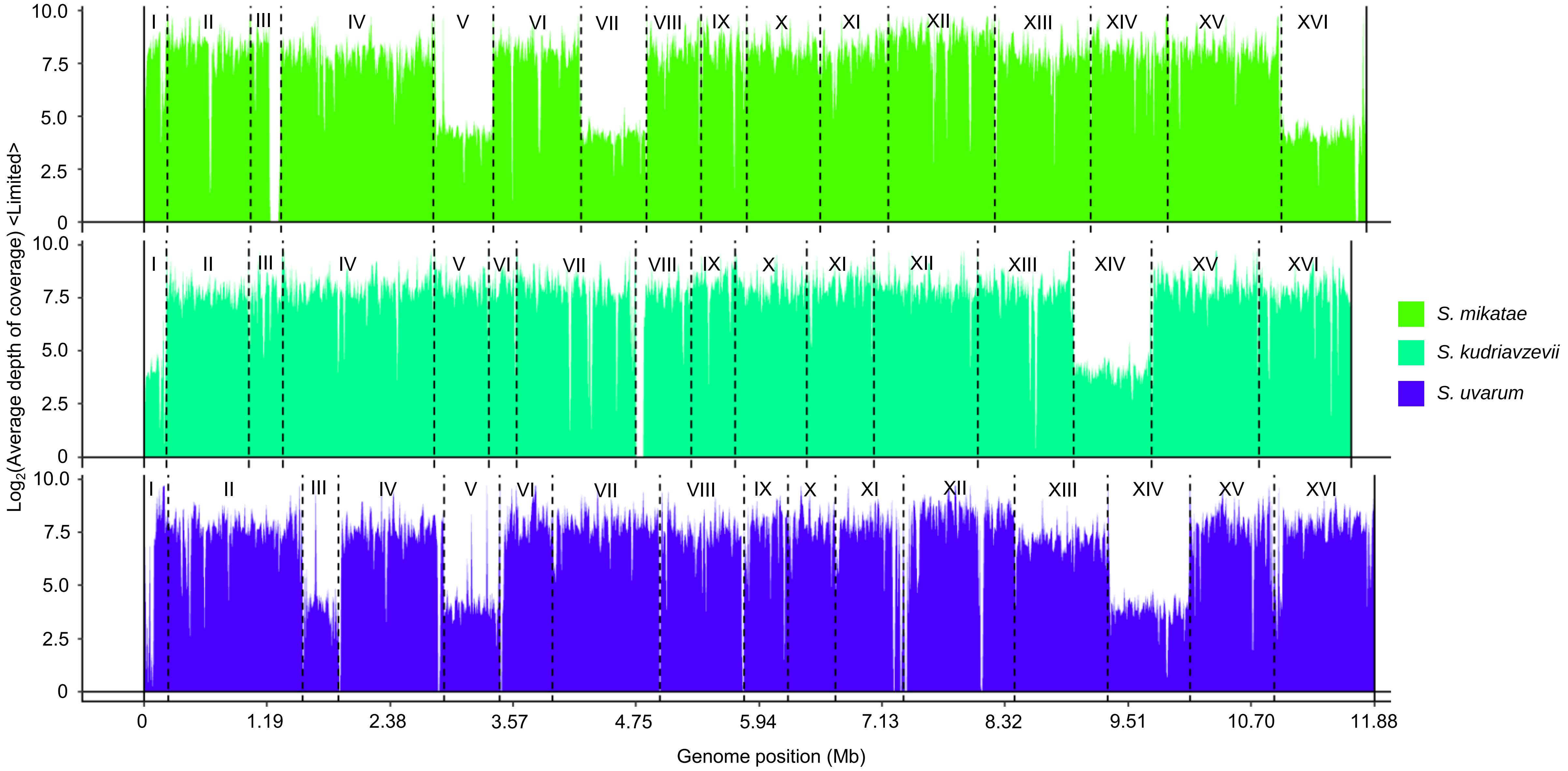

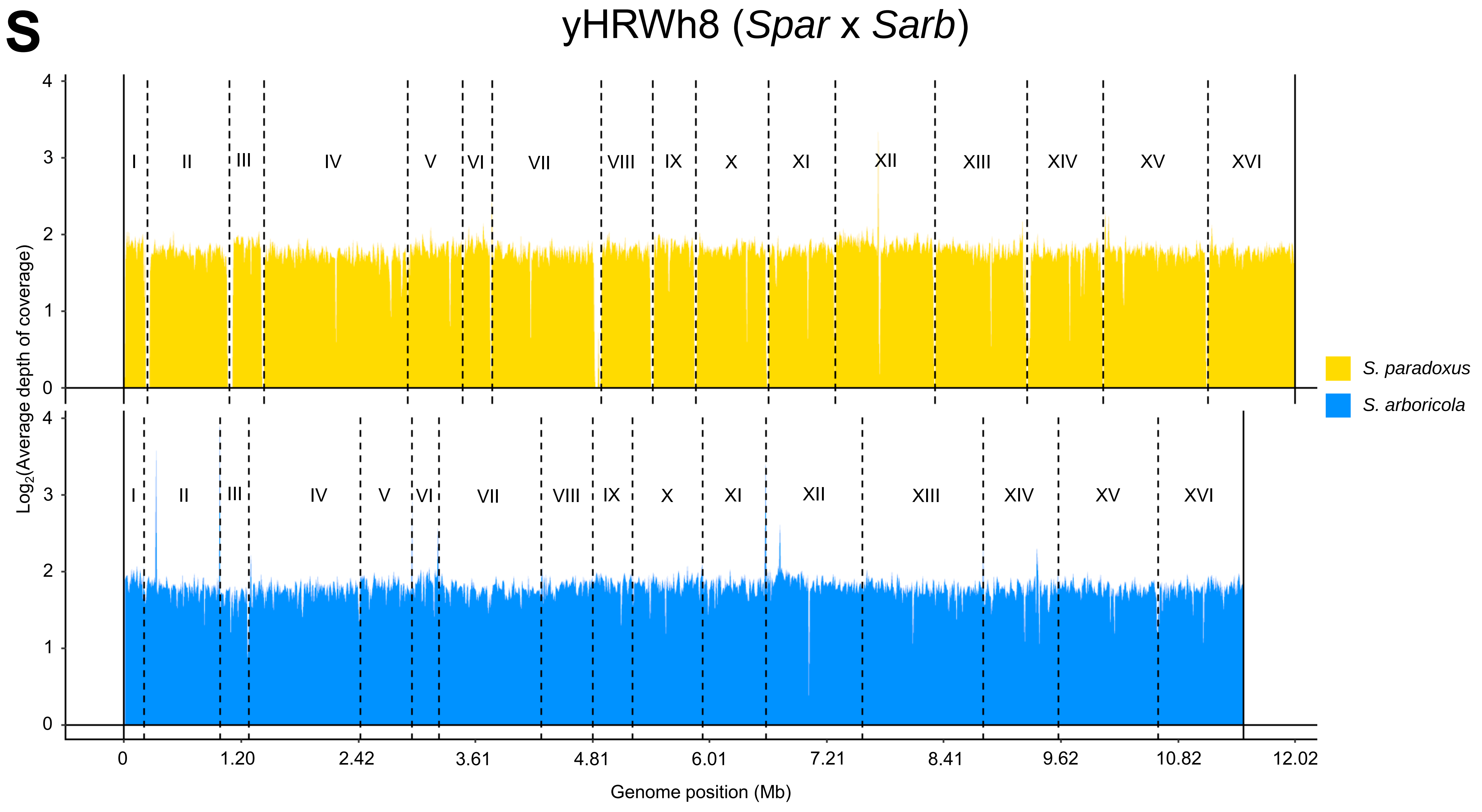
S
yHRWh8 (*Spar* x *Sarb*)
I
II
III
IV
V
VI
VII
VIII
IX
X
XI
XII
XIII
XIV
XV
XVI
Log2(Average depth of coverage)
0
1
2
3
4
0
1.20
2.42
3.61
4.81
6.01
7.21
8.41
9.62
10.82
12.02
Genome position (Mb)
*S. paradoxus*
*S. arboricola*

**T**

yHRWh42 (*Spar* x *Sarb* x *Scer*)

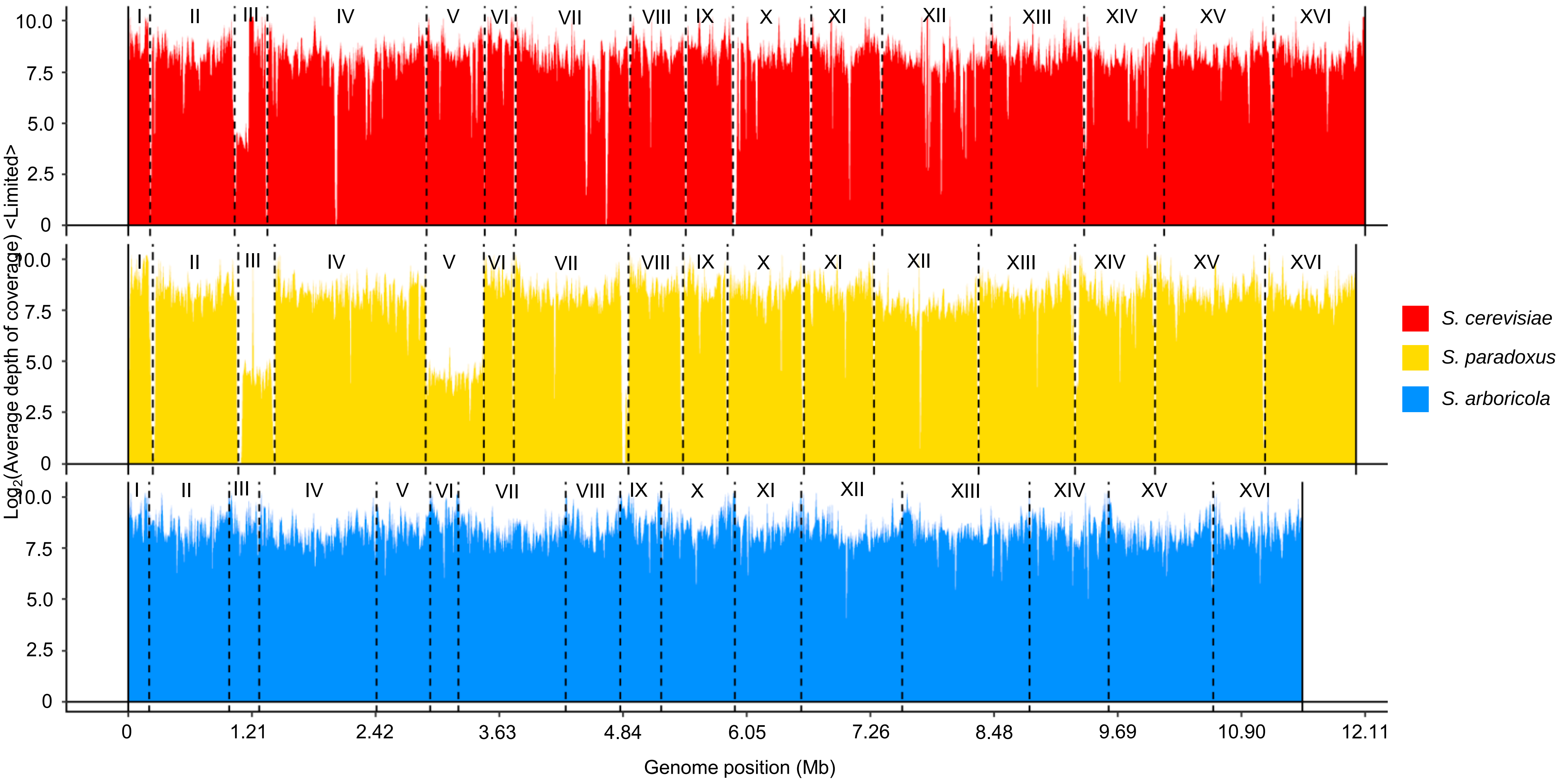

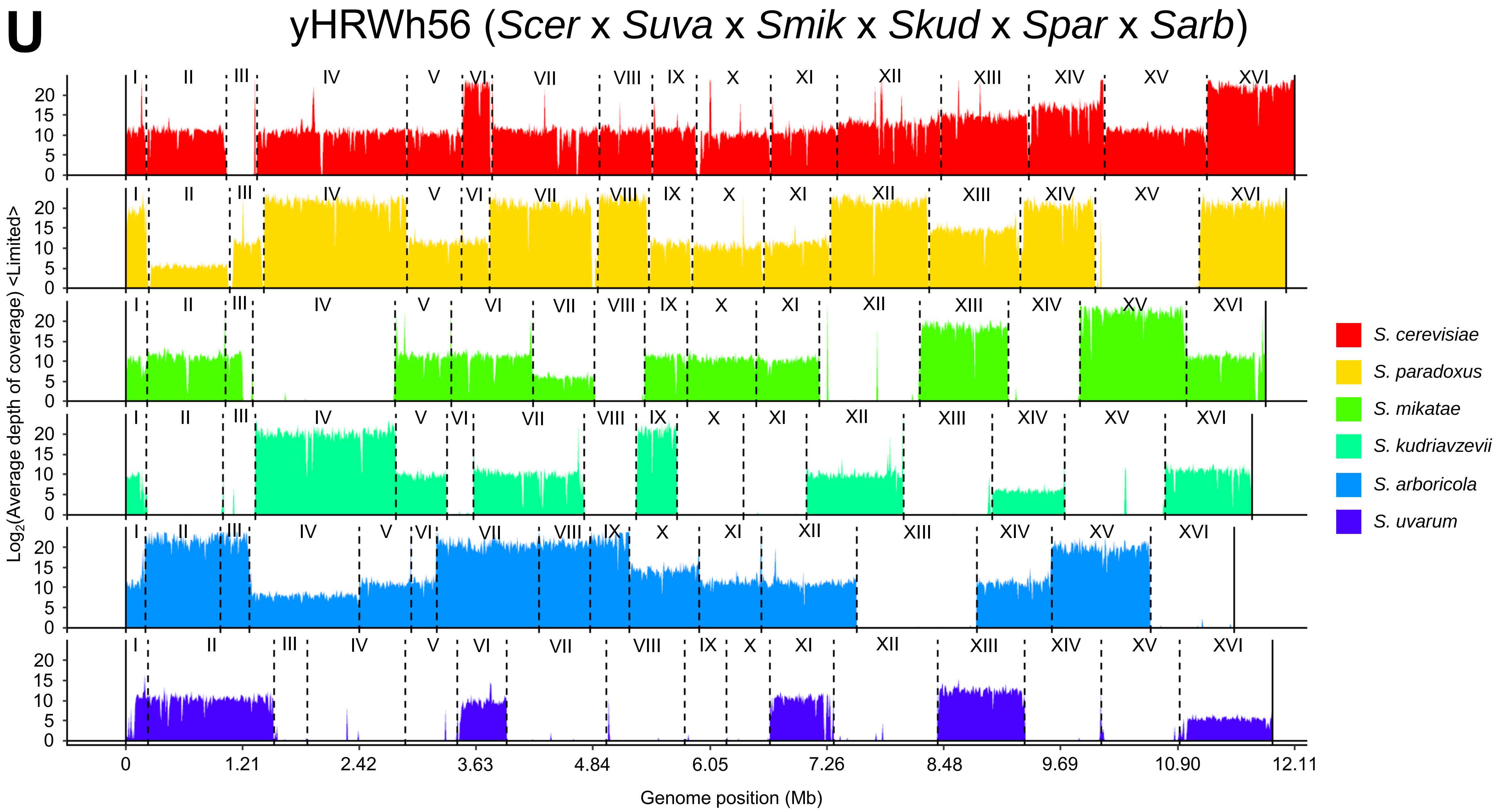

U
yHRWh56 (Scer x Suva x Smik x Skud x Spar x Sarb)
Log2(Average depth of coverage) <Limited>
Genome position (Mb)
0
1.21
2.42
3.63
4.84
6.05
7.26
8.48
9.69
10.90
12.11
I
II
III
IV
V
VI
VII
VIII
IX
X
XI
XII
XIII
XIV
XV
XVI
S. cerevisiae
S. paradoxus
S. mikatae
S. kudriavzevii
S. arboricola
S. uvarum

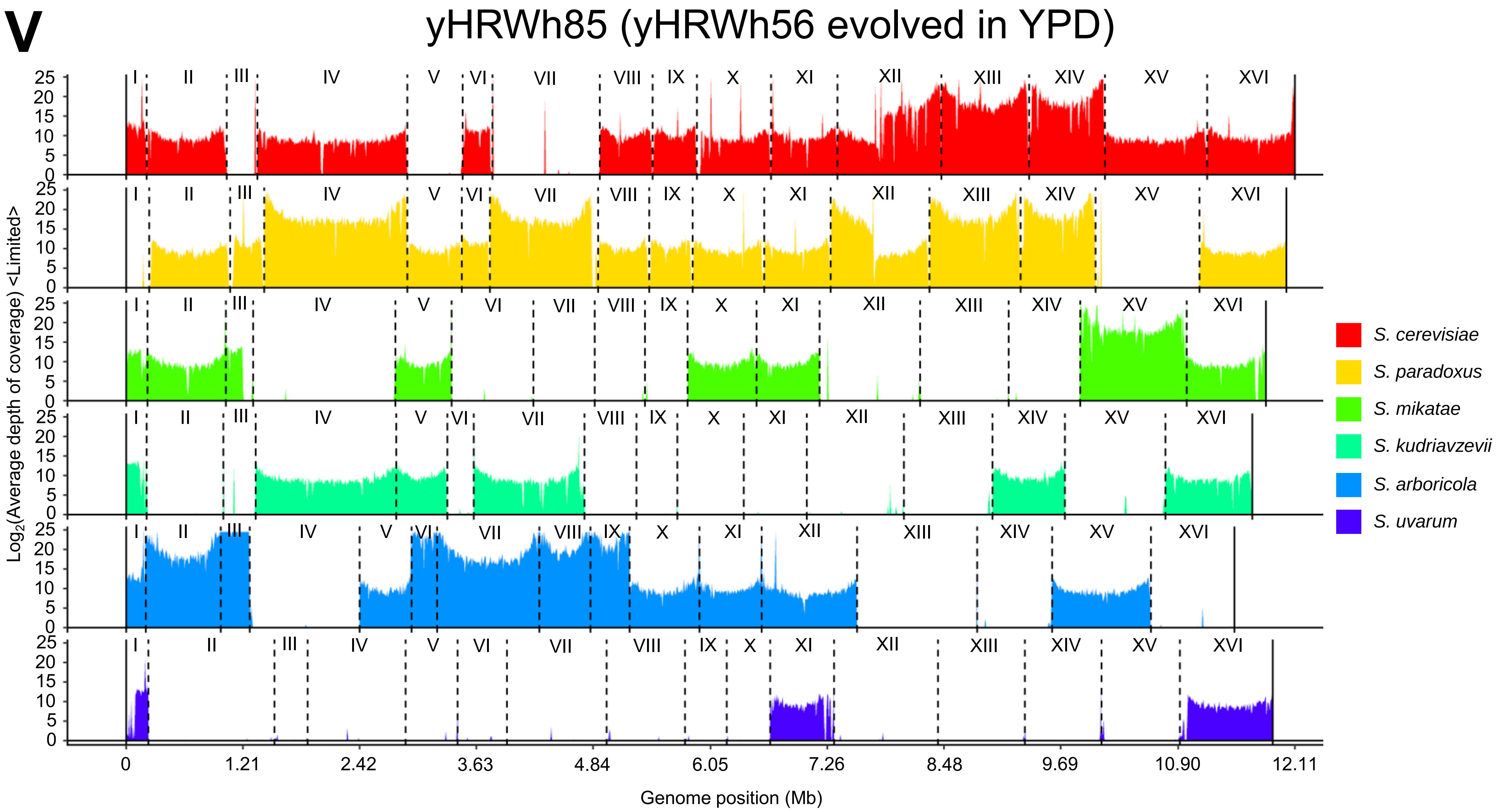

V
yHRWh85 (yHRWh56 evolved in YPD)
Log2(Average depth of coverage) <Limited>
Genome position (Mb)
0
1.21
2.42
3.63
4.84
6.05
7.26
8.48
9.69
10.90
12.11
I
II
III
IV
V
VI
VII
VIII
IX
X
XI
XII
XIII
XIV
XV
XVI
S. cerevisiae
S. paradoxus
S. mikatae
S. kudriavzevii
S. arboricola
S. uvarum

W

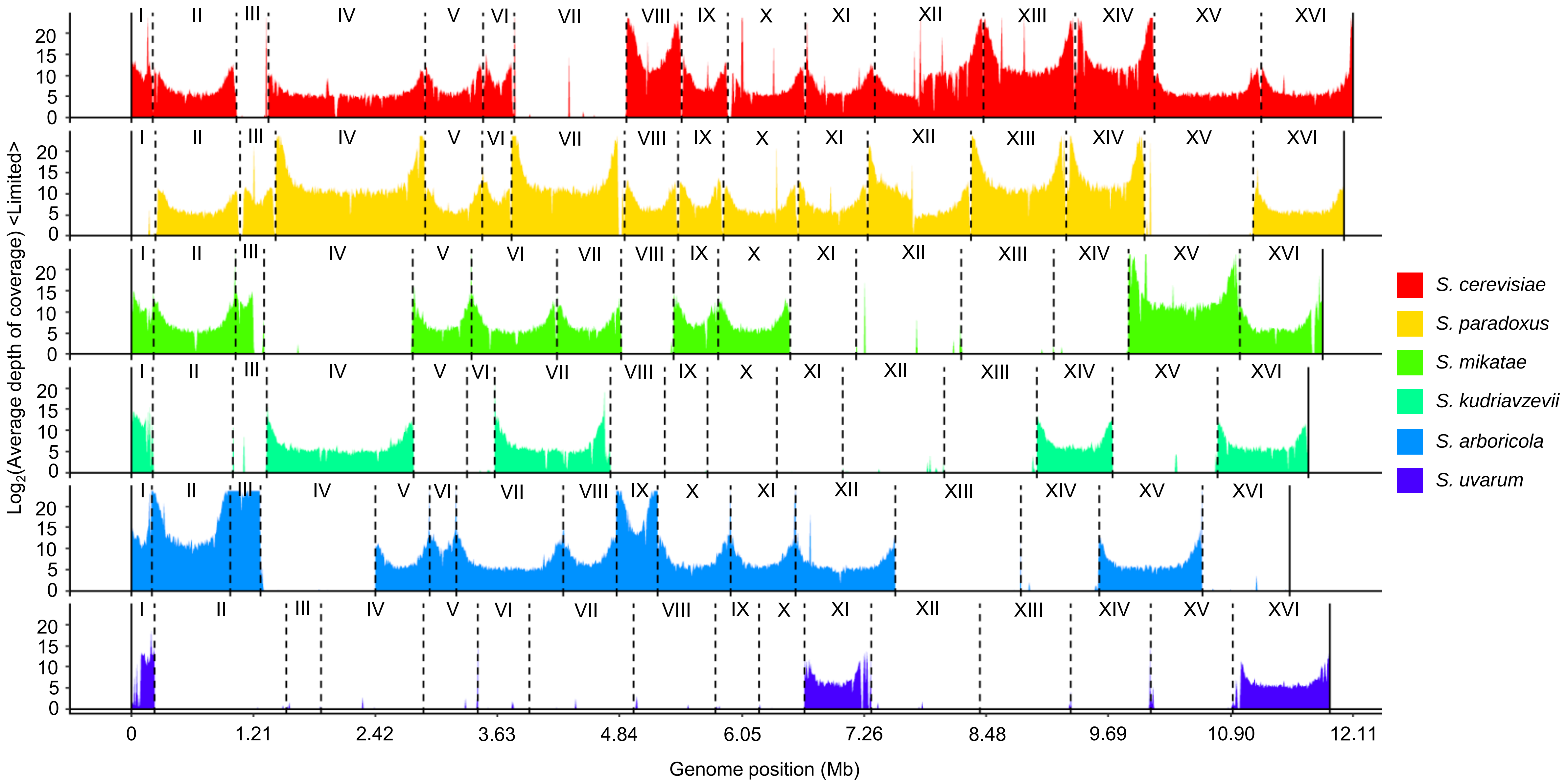

yHRWh86 (yHRWh56 evolved in YPD)
I
II
III
IV
V
VI
VII
VIII
IX
X
XI
XII
XIII
XIV
XV
XVI
Log2(Average depth of coverage) <Limited>
0
5
10
15
20
S. cerevisiae
S. paradoxus
S. mikatae
S. kudriavzevii
S. arboricola
S. uvarum
0
1.21
2.42
3.63
4.84
6.05
7.26
8.48
9.69
10.90
12.11
Genome position (Mb)

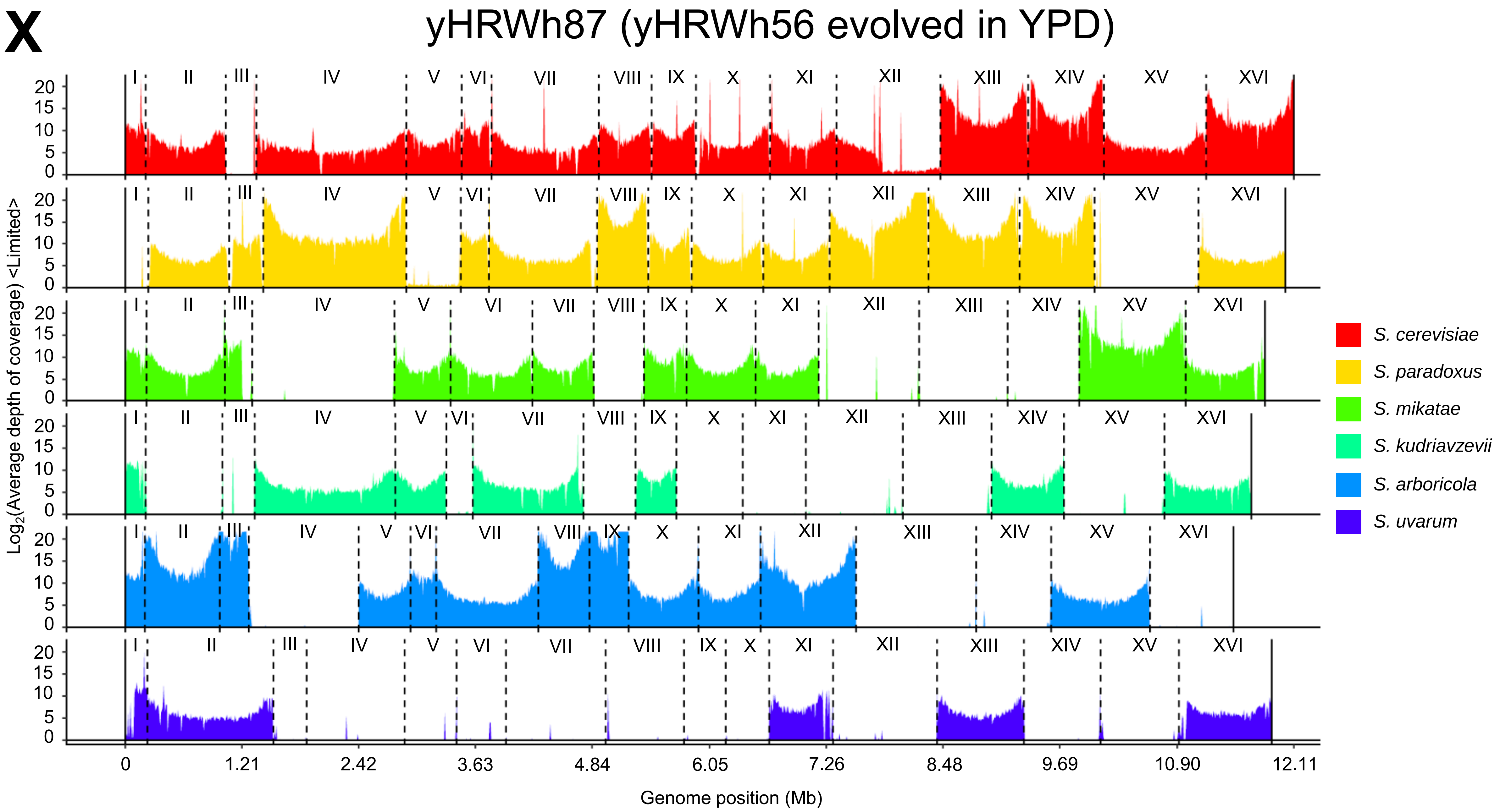

X
yHRWh87 (yHRWh56 evolved in YPD)
Log$_2$(Average depth of coverage) <Limited>
I II III IV V VI VII VIII IX X XI XII XIII XIV XV XVI
0 5 10 15 20
0 1.21 2.42 3.63 4.84 6.05 7.26 8.48 9.69 10.90 12.11
Genome position (Mb)
S. cerevisiae
S. paradoxus
S. mikatae
S. kudriavzevii
S. arboricola
S. uvarum

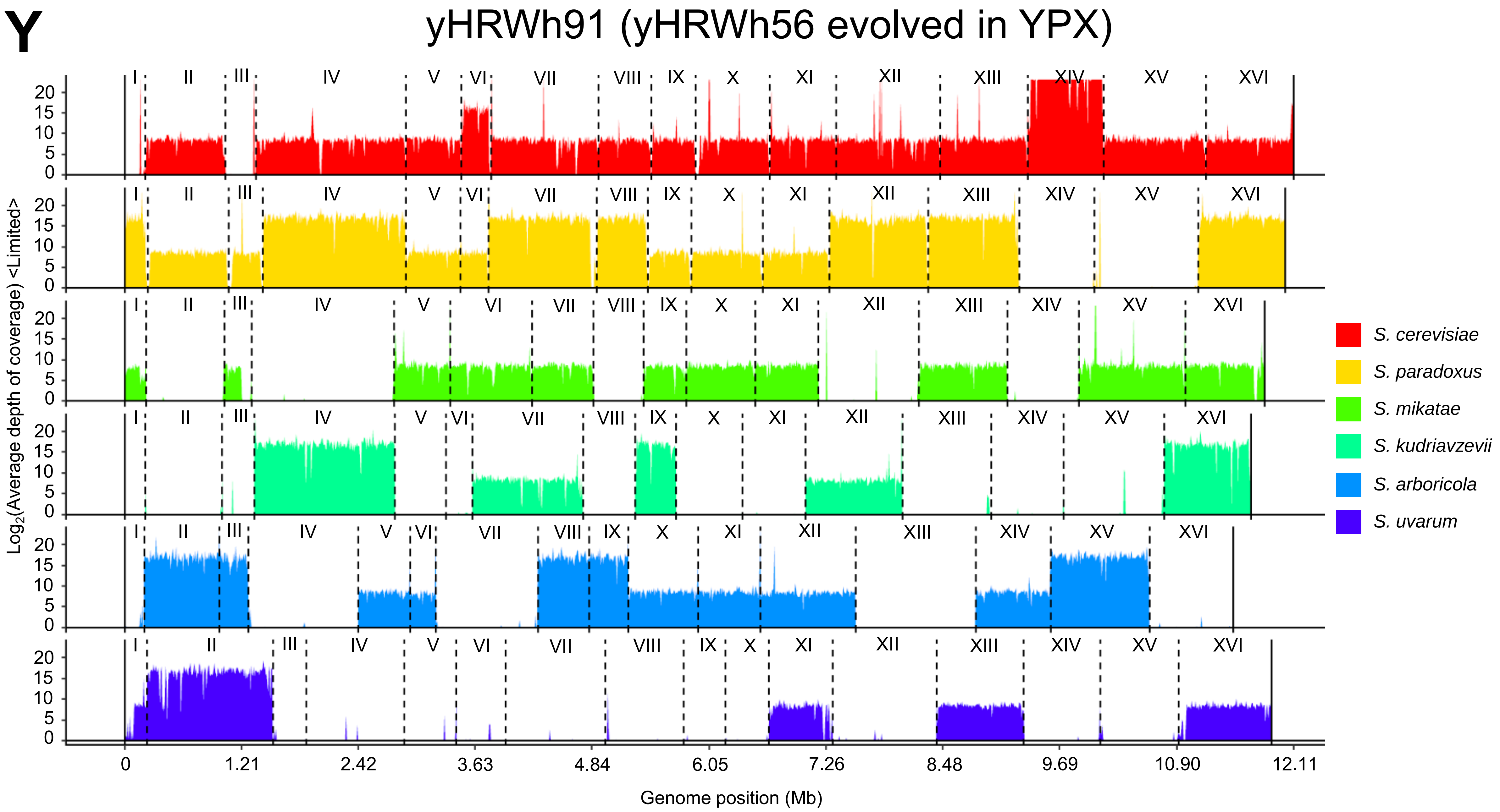
Y
yHRWh91 (yHRWh56 evolved in YPX)
Log2(Average depth of coverage) <Limited>
I
II
III
IV
V
VI
VII
VIII
IX
X
XI
XII
XIII
XIV
XV
XVI
0
5
10
15
20
0
1.21
2.42
3.63
4.84
6.05
7.26
8.48
9.69
10.90
12.11
Genome position (Mb)
S. cerevisiae
S. paradoxus
S. mikatae
S. kudriavzevii
S. arboricola
S. uvarum

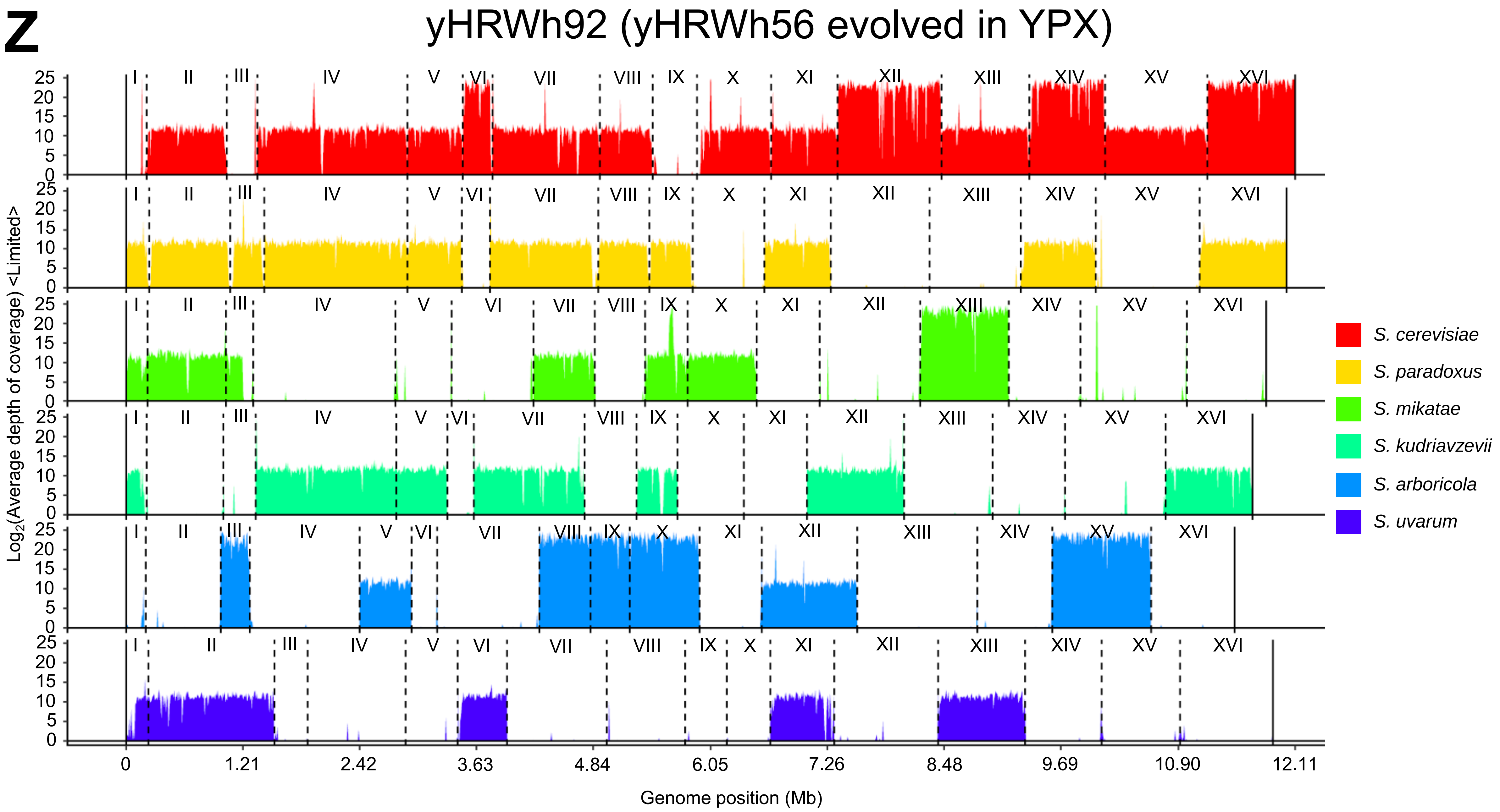
Z
yHRWh92 (yHRWh56 evolved in YPX)
Log2(Average depth of coverage) <Limited>
Genome position (Mb)
0
1.21
2.42
3.63
4.84
6.05
7.26
8.48
9.69
10.90
12.11
I II III IV V VI VII VIII IX X XI XII XIII XIV XV XVI
S. cerevisiae
S. paradoxus
S. mikatae
S. kudriavzevii
S. arboricola
S. uvarum

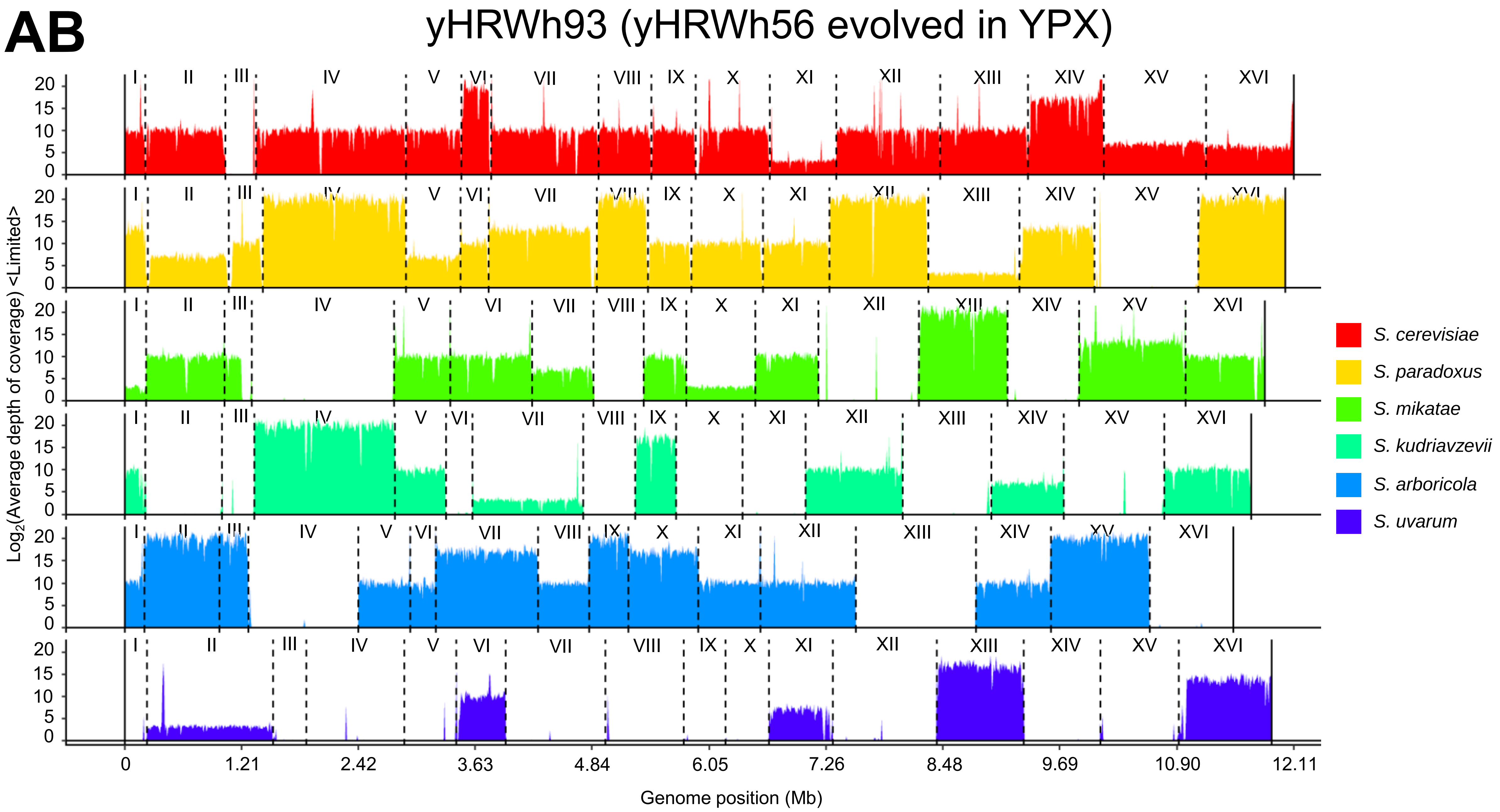

AB
yHRWh93 (yHRWh56 evolved in YPX)
Log2(Average depth of coverage) <Limited>
Genome position (Mb)
0
1.21
2.42
3.63
4.84
6.05
7.26
8.48
9.69
10.90
12.11
I II III IV V VI VII VIII IX X XI XII XIII XIV XV XVI
S. cerevisiae
S. paradoxus
S. mikatae
S. kudriavzevii
S. arboricola
S. uvarum

**Supplementary Figure 3 | Nuclear genome composition of the diploidized *S. cerevisiae* reference strain and the synthetic and evolved hybrids.** Panels A-Z,AB are the `sppIDer` outputs for the diploidized reference strain of *S. cerevisiae* (GLBRCY101) and the synthetic and evolved hybrids. Sequencing coverage values are colored according to each *Saccharomyces* species' contribution in that portion of the genome. Panels were ordered to represent synthetic hybrid data based on the order they were used to generate the next hybrid (Supplementary Fig. 2). `sppIDer` produces multiple plots [19], but here we show the $\log_2$ of the average coverage of ~8 Kbp-windows normalized to the genome-wide average coverage. To improve the resolution of the three-, four-, and six-species hybrid plots, window coverage values were normalized to the genome-wide average coverage, and values were limited to the 99% percentile and below. Source data are provided at http://bit.ly/2v1rq1T.

# Supplementary figure 4

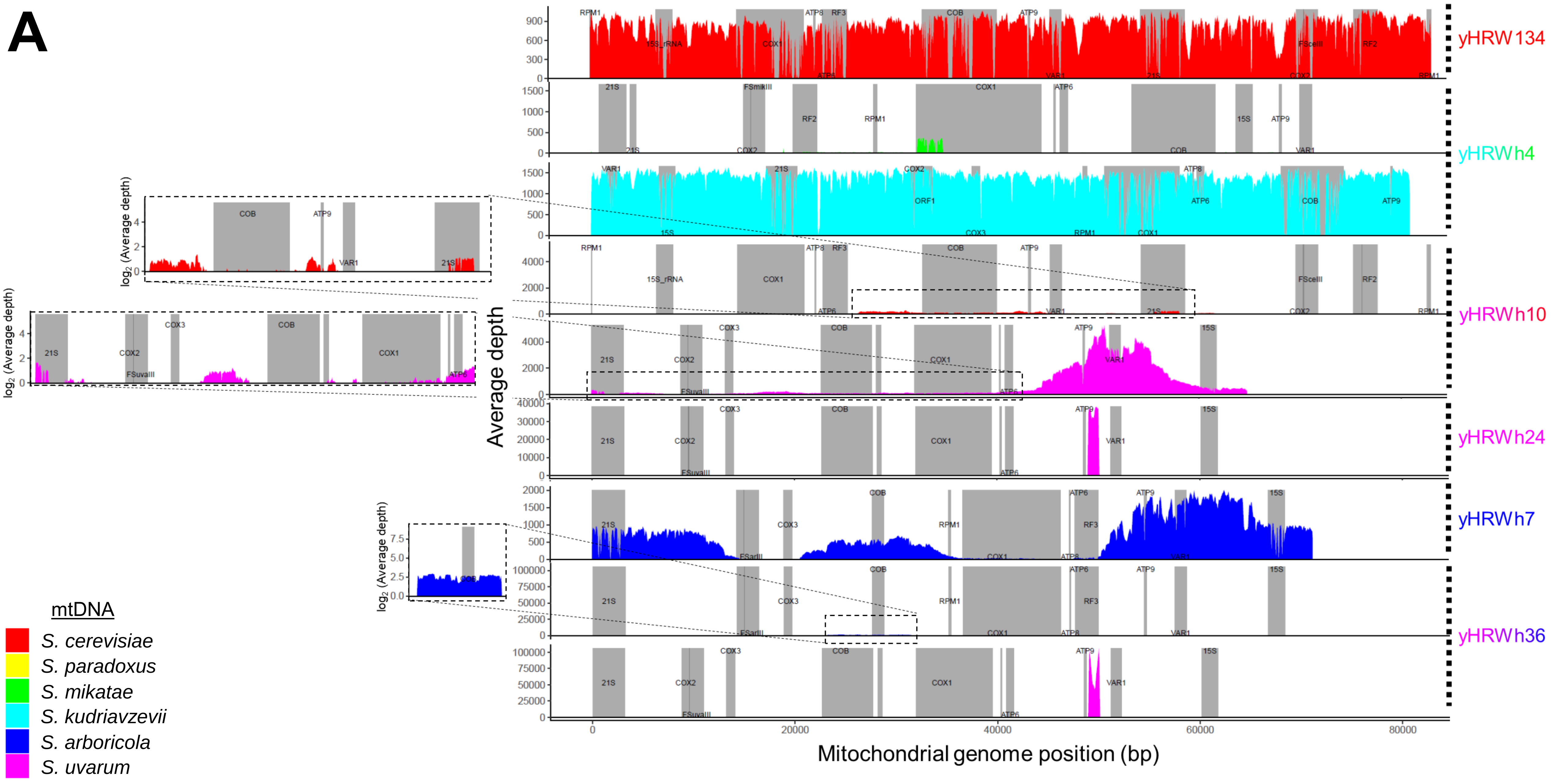

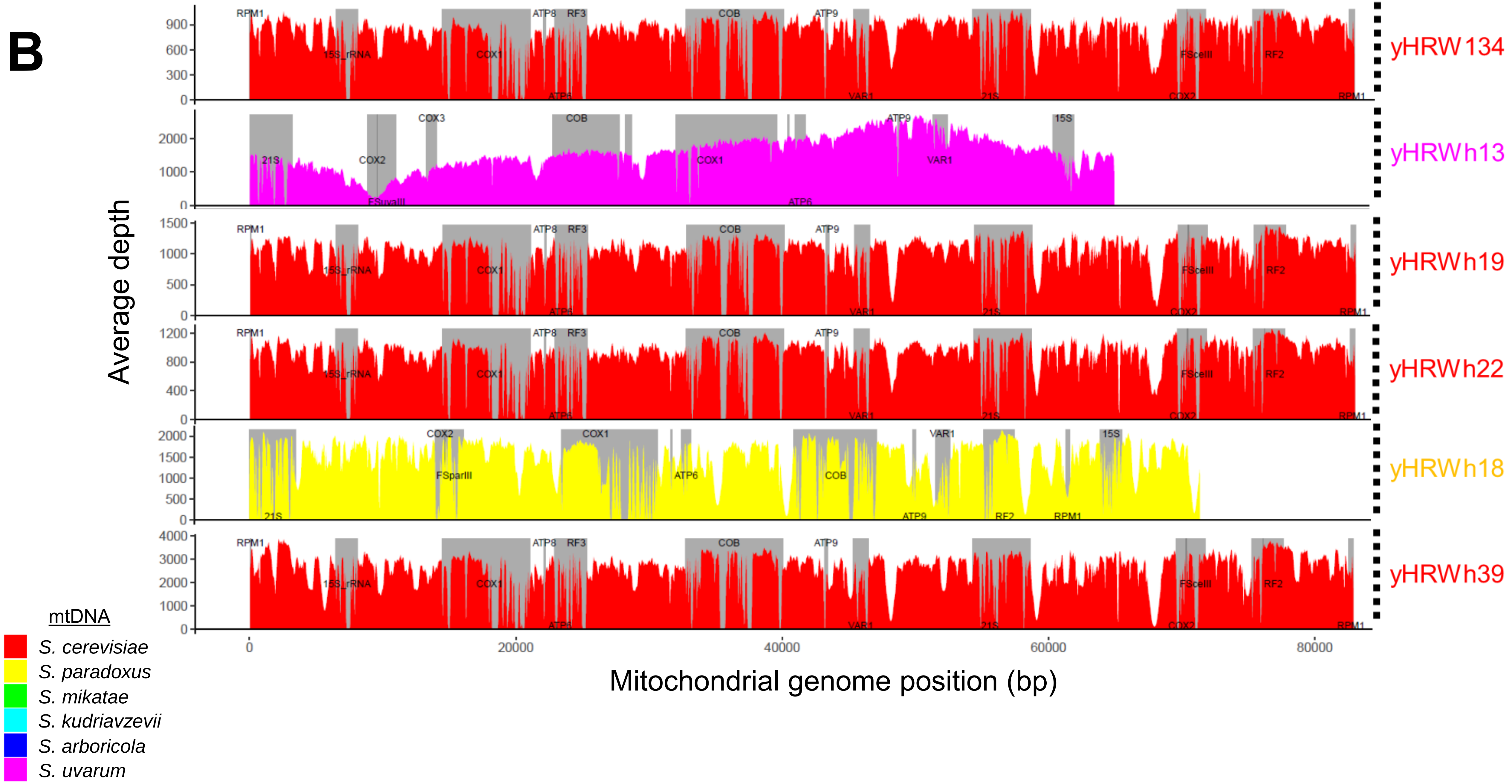
B
Average depth
Mitochondrial genome position (bp)
yHRW134
yHRWh13
yHRWh19
yHRWh22
yHRWh18
yHRWh39
mtDNA
S. cerevisiae
S. paradoxus
S. mikatae
S. kudriavzevii
S. arboricola
S. uvarum

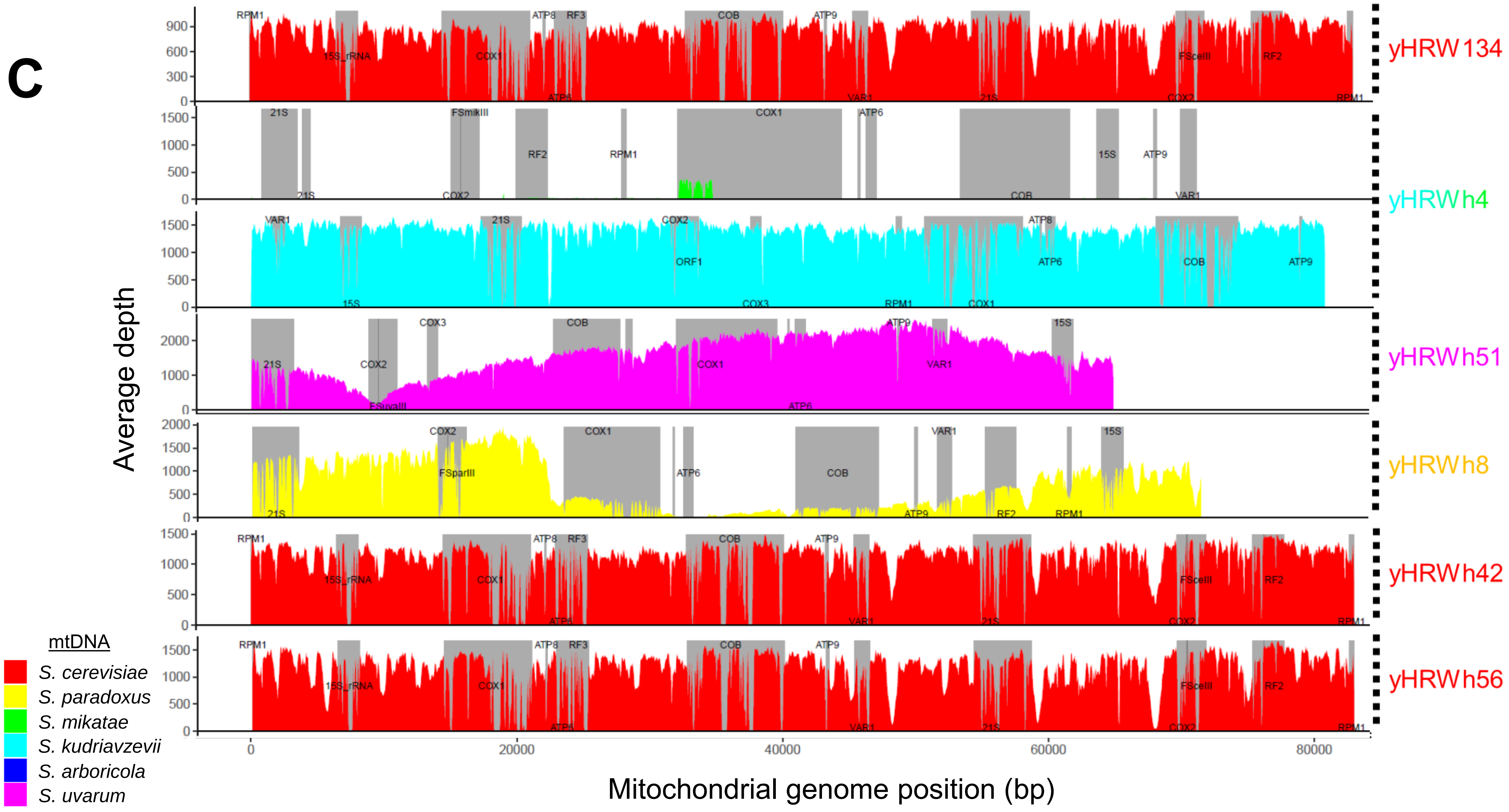
C
yHRW134
yHRWh4
yHRWh51
yHRWh8
yHRWh42
yHRWh56
Average depth
Mitochondrial genome position (bp)
mtDNA
S. cerevisiae
S. paradoxus
S. mikatae
S. kudriavzevii
S. arboricola
S. uvarum

**Supplementary Figure 4 | Mitochondrial genome inheritance of the diploidized *S. cerevisiae* reference strain and the synthetic and evolved hybrids.** Panels A-C are the `sppIDer` outputs for the diploidized reference strain of *S. cerevisiae* (GLBRCY101) and the synthetic and evolved hybrids. Sequencing coverage values are colored according to each *Saccharomyces* species' contribution in that portion of the mtDNA. Each panel contains the mtDNA inheritance for the synthetic hybrid used to generate that particular six-species hybrids (Supplementary Fig. 2). `sppIDer` produces multiple plots [19], but here we show the $\log_2$ of the average coverage of 44-bp windows normalized to the mtDNA-wide average coverage. When a synthetic hybrid is formed between parent strains that both contain mtDNA, a heteroplasmic state can be maintained for several generations, but eventually, a parent or recombinant mtDNA is generally fixed [17]. In some hybrids, this heteroplasmic state persisted, and the names of hybrids are colored in a gradient according to the detected mtDNAs; these colors are also displayed in Figure 4A. Due to the unusually high coverage of *ATP9* or *ATP9-VAR1-15S rRNA* of *S. uvarum* in panel A), additional inset plots with limited y-axes are shown. Source data are provided at http://bit.ly/2v1rq1T.

**Supplementary figure 5**

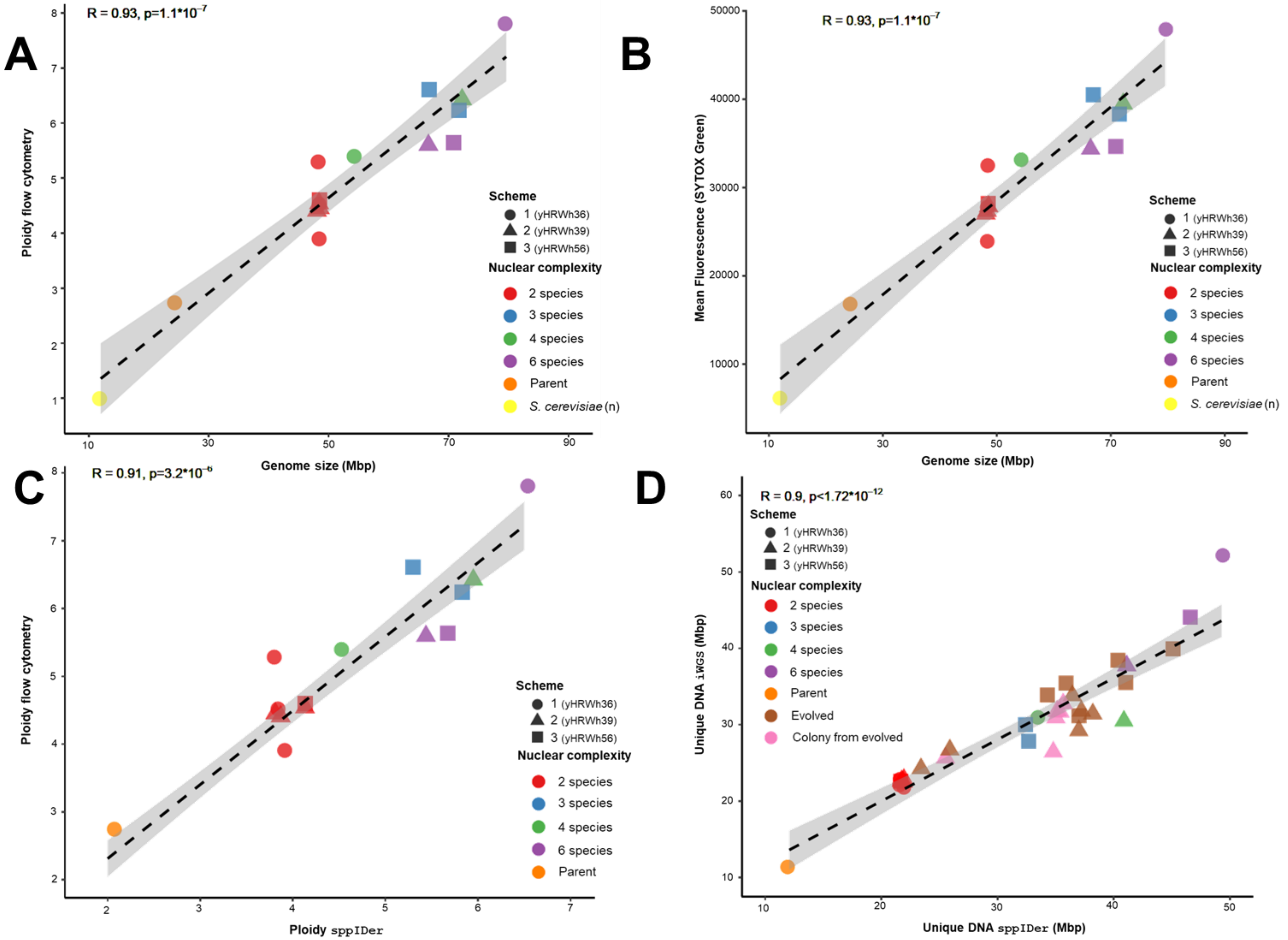

**Supplementary Figure 5 | Ploidy and genome size estimations were well-correlated among different methods.** A) Genome size (Supplementary Data 2) was correlated with the ploidy estimates from flow cytometry. B) Genome size was correlated with the mean fluorescence (n=10000, counts per strain) of SYTOX Green. C) Ploidy estimated from `iWGS` (Supplementary Data 2) was correlated with the ploidy estimated using flow cytometry. D) The estimates of the amount of unique DNA present were correlated between `iWGS` and `sppIDer`. `sppIDer` values were corrected for copy number to generate the genome size estimates in the other panels. Points are colored according to the number of species (nuclear complexity) genomes contributing to the strain. Synthetic hybrids generated from each independent scheme are represented with different shapes. The Spearman rank sum test R and *p*-value are displayed. Linear regression lines and their 95% confidence intervals of the fit are represented with a black line and gray shadow, respectively. Source data are provided at http://bit.ly/2v1rq1T.

**Supplementary figure 6**

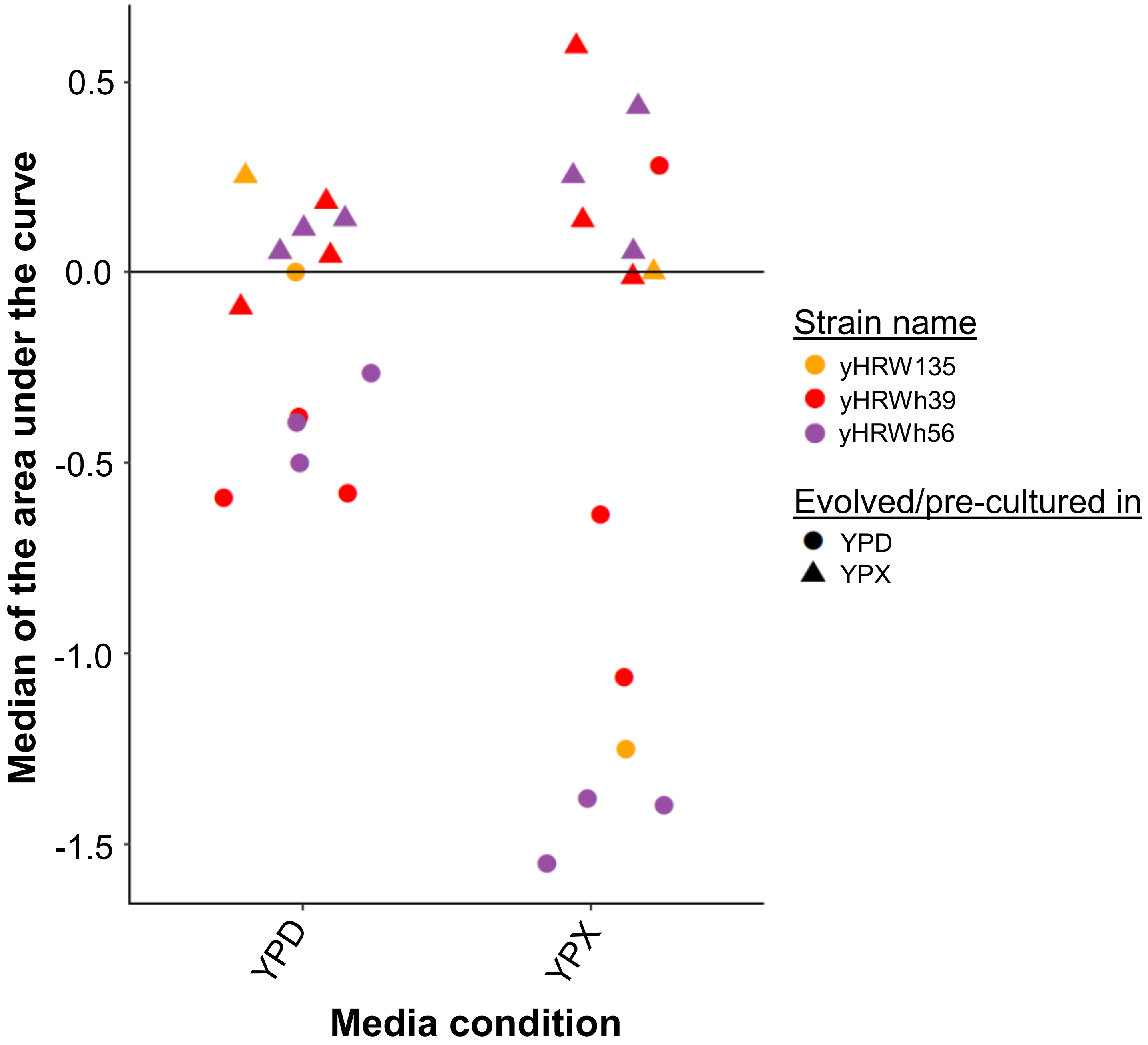

**Supplementary Figure 6 | Growth kinetics for ancestor and evolved six-species populations in a microtiter plate.** Growth measured as area under the curve (AUC) for the reference *S. cerevisiae* strain and evolved six-species hybrids (n = 6 independent biological replicates), following normalization to the *S. cerevisiae* reference strain yHRW135 (full microtiter plate kinetic parameters are reported in Supplementary Data 4). Different shapes indicate the media in which the synthetic six-species hybrids were evolved. Colors differentiate the reference strain (orange), and the ancestor of evolved hybrids (red for hybrids evolved from yHRWh39 and purple for hybrids evolved from yHRWh56), while each data point represents an evolved replicate population. Source data are provided at http://bit.ly/2v1rq1T.

## Supplementary figure 7

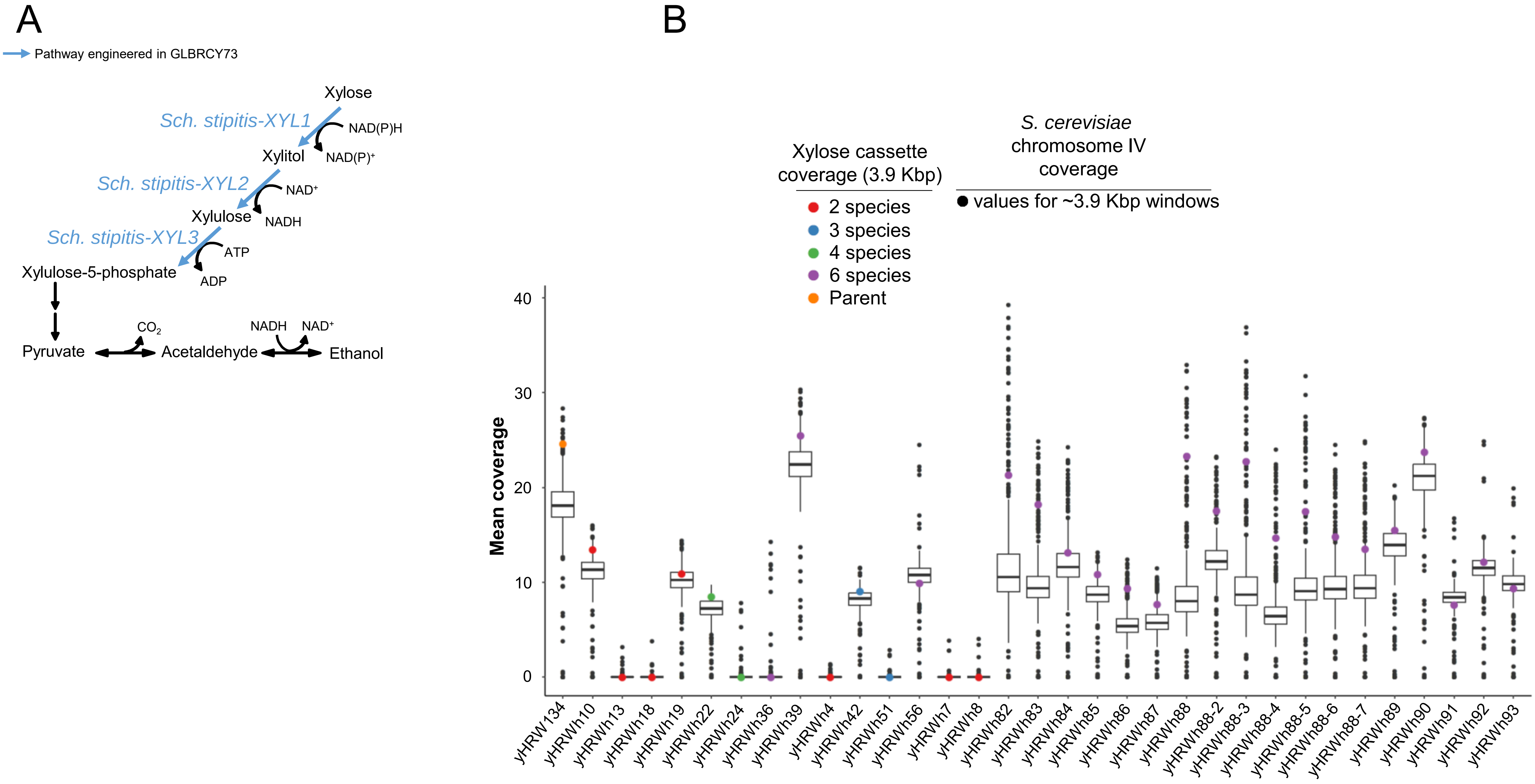

**Supplementary Figure 7 | The fitness improvement during adaptive laboratory evolution was not due to an increase in the number of copies of xylose utilization genes.** A) Schematic representation of the metabolic pathway for xylose utilization. The engineered xylose utilization genes are highlighted in blue. B) Boxplots of coverage levels for 3.9 Kbp windows (n = 396 windows) of chromosome IV are displayed for each strain. Median values for the strains are represented by a horizontal line inside the box, and the upper and lower whiskers represent the highest and lowest values of the 1.5 * IQR (inter-quartile range), respectively. Color dots show the coverage values for the coding sequences of the engineered xylose utilization genes. Points are colored according to the number of species (nuclear complexity) contributing to the strain. The coverage values of the xylose utilization genes were not significantly higher than the values for the chromosome IV (Supplementary Data 7). Source data are provided at http://bit.ly/2v1rq1T.

**Supplementary figure 8**

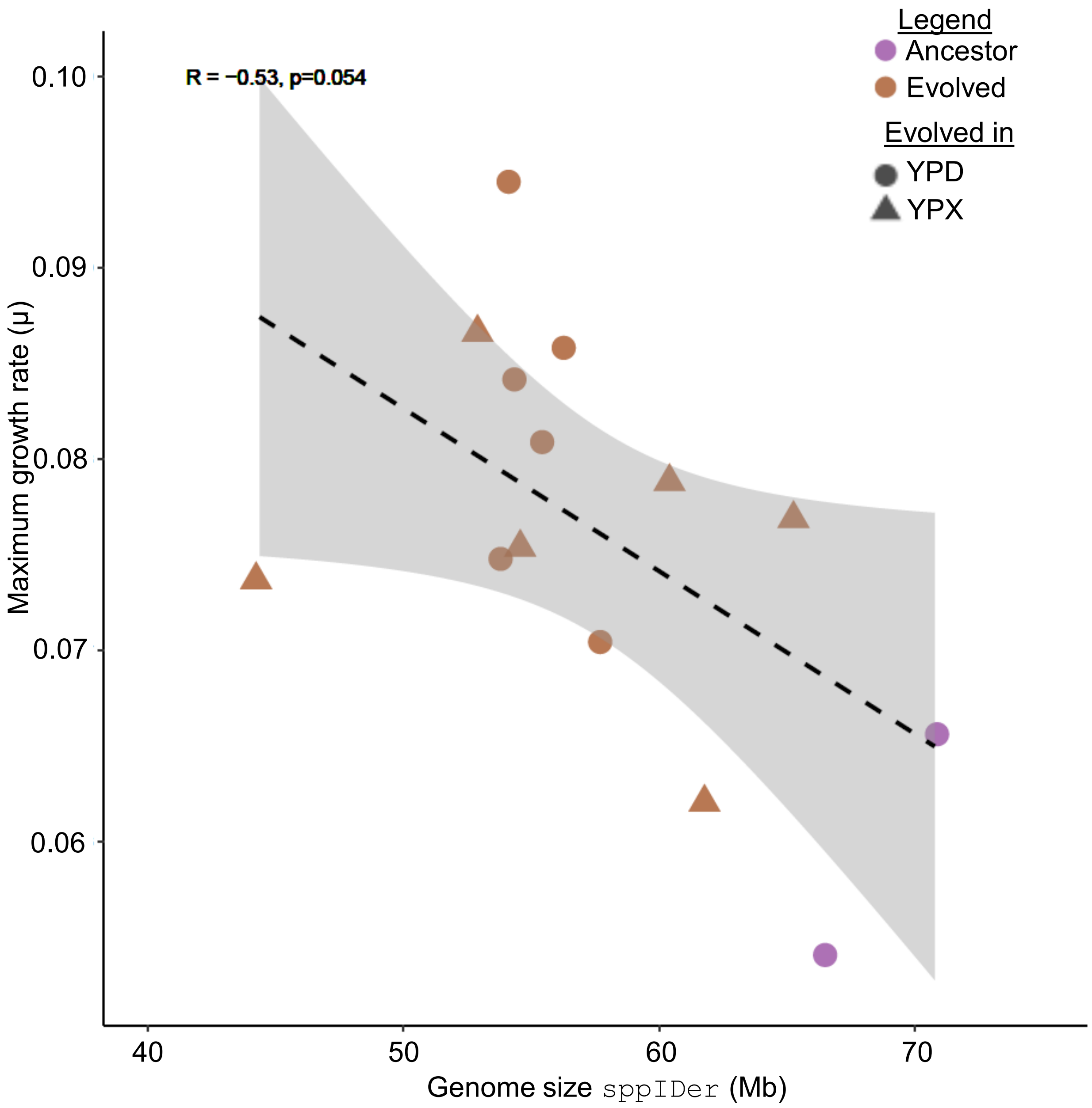

**Supplementary Figure 8 | The fitness improvement during adaptive laboratory evolution was not simply due to genome reduction.** Genome size (Supplementary Data 2) was not significantly correlated (Spearman rank sum test) with the maximum specific growth rate (μ, defined as (ln(OD2)-ln(OD1))/(T2-T1))) (Supplementary Data 5) (n = 6 independent biological replicates). Ancestor (purple dots) and evolved six-species strains (brown dots) are shown. Different shapes indicate the media in which the synthetic six-species hybrids were evolved. Color points differentiate the ancestor from the evolved hybrids. Source data are provided at http://bit.ly/2v1rq1T.

# Supplementary figure 9

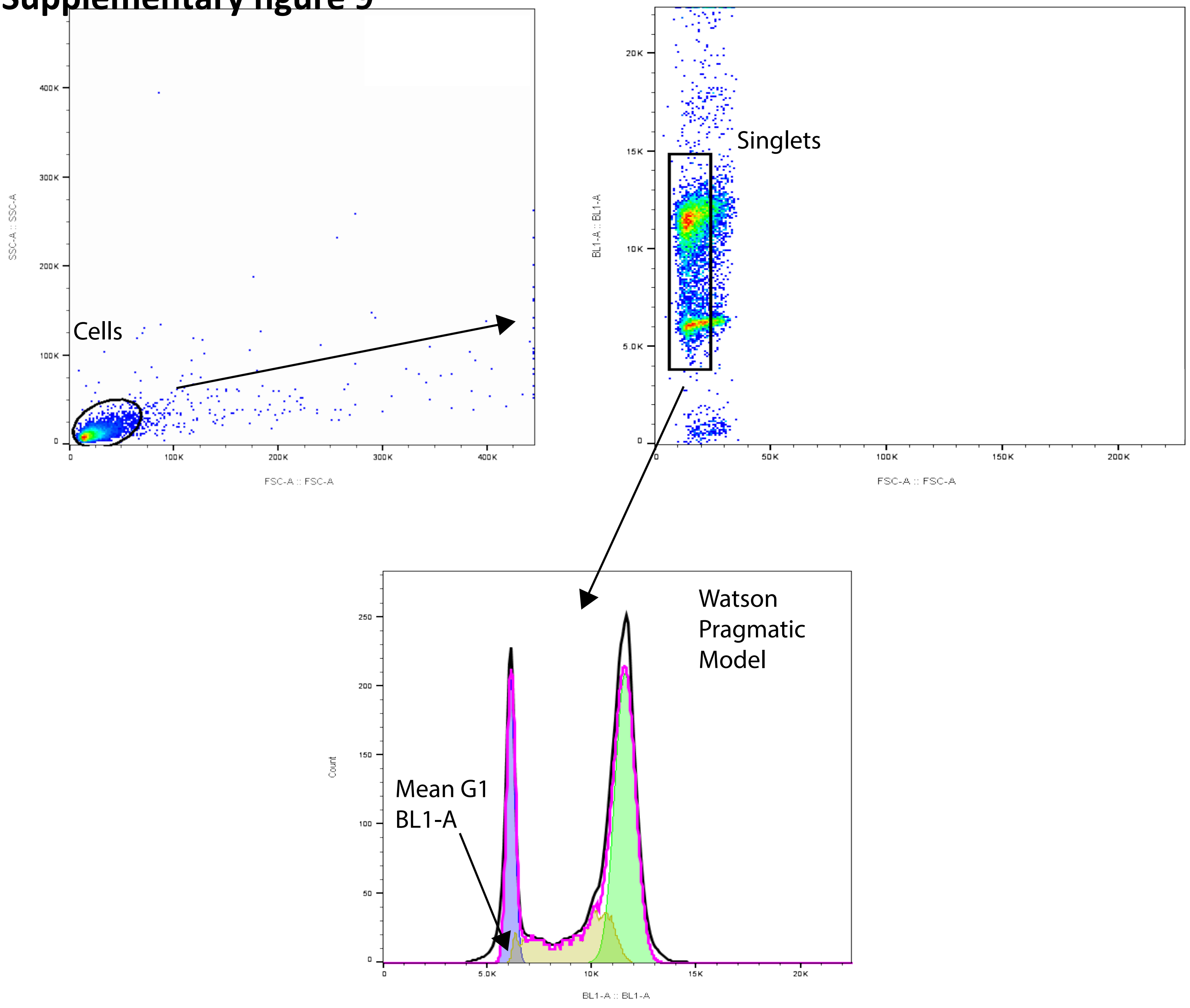

**Supplementary Figure 9 | Flow cytometry gating schematic.** Files were processed in FlowJo. Samples were first gated on SSC and FSC to remove debris (upper left panel). Doublets were then removed by gating on BL1-A and FSC-A (upper right panel). A histogram of BL1-A values were then generated for remaining cells (lower panel). G1 and G2 peaks were identified by applying a Watson Pragmatic Cell Cycle model to extrapolate G1 and G2 means. Mean G1 BL1-A values for each strain were used in genome size analyses in Supplementary Figure 5 and Supplementary Data 2.

## References


1 Ivorra, C., Pérez-Ortín, J. E., and del Olmo, M. l., "An inverse correlation between stress resistance and stuck fermentations in wine yeasts. A molecular study," *Biotechnol. Bioeng.* **64**, 698-708 (1999).

2 Carrasco, P., Querol, A., and del Olmo, M. l., "Analysis of the stress resistance of commercial wine yeast strains," *Arch Microbiol* **175**, 450-457 (2001).

3 Erasmus, D. J., van der Merwe, G. K., and van Vuuren, H. J. J., "Genome-wide expression analyses: Metabolic adaptation of *Saccharomyces cerevisiae* to high sugar stress," *FEMS Yeast Res.* **3**, 375-399 (2003).

4 Briggs*, et al.*, *brewing: science and practice* (Woodhead, Cambridge, UK, 2004).

5 Dunn, B. and Sherlock, G., "Reconstruction of the genome origins and evolution of the hybrid lager yeast *Saccharomyces pastorianus*," *Genome Res.* **18**, 1610-1623 (2008).

6 Peris, D.*, et al.*, "Reconstruction of the evolutionary history of *Saccharomyces cerevisiae* x *S. kudriavzevii* hybrids based on multilocus sequence analysis," *PLoS ONE* **7**, e45527 (2012).

7 Pérez-Través, L.*, et al.*, "On the complexity of the *Saccharomyces bayanus* taxon: hybridization and potential hybrid speciation," *PLoS ONE* **9**, e93729 (2014).

8 Belloch, C.*, et al.*, "The chimerical genomes of natural hybrids between *Saccharomyces cerevisiae* and *Saccharomyces kudriavzevii*," *Appl Environ Microbiol* **75**, 2534-2544 (2009).

9 Sipiczki, M., "Interspecies hybridization and recombination in *Saccharomyces* wine yeasts," *FEMS Yeast Res.* **8**, 996-1007 (2008).

10 Peris, D.*, et al.*, "Comparative genomics among *Saccharomyces cerevisiae* x *Saccharomyces kudriavzevii* natural hybrid strains isolated from wine and beer reveals different origins," *BMC Genomics* **13**, 407 (2012).

11 Haber, J. E., "Mating-type genes and *MAT* switching in *Saccharomyces cerevisiae*," *Genetics* **191**, 33-64 (2012).

12 Alexander, W. G.*, et al.*, "Efficient engineering of marker-free synthetic allotetraploids of *Saccharomyces*," *Fungal Genet Biol* **89**, 10-17 (2016).

13 Kumaran, R., Yang, S. Y., and Leu, J. Y., "Characterization of chromosome stability in diploid, polyploid and hybrid yeast cells," *PLoS ONE* **8**, e68094 (2013).

14 Karanyicz, E., *et al.*, "Non-introgressive genome chimerisation by malsegregation in autodiploidised allotetraploids during meiosis of *Saccharomyces kudriavzevii* x *Saccharomyces uvarum* hybrids," *Appl Microbiol Biot* **101**, 1-17 (2017).

15 Braun-Galleani, S., *et al.*, "*Zygosaccharomyces pseudobailii*, another yeast interspecies hybrid that regained fertility by damaging one of its MAT loci," *FEMS Yeast Res.* **18**, foy079 (2018).

16 Ortiz-Merino, R. A., *et al.*, "Evolutionary restoration of fertility in an interspecies hybrid yeast, by whole-genome duplication after a failed mating-type switch," **15**, e2002128 (2017).

17 Berger, K. H. and Yaffe, M. P., "Mitochondrial DNA inheritance in *Saccharomyces cerevisiae*," *Trends Microbiol* **8**, 508-513 (2000).

18 Ling, F., *et al.*, "Din7 and Mhr1 expression levels regulate double-strand-break-induced replication and recombination of mtDNA at ori5 in yeast," *Nucl. Acids Res.* **41**, 5799-5816 (2013).

19 Langdon, Q. K., *et al.*, "sppIDer: a species identification tool to investigate hybrid genomes with high-throughput sequencing," *Mol Biol Evol* **35**, 2835-2849 (2018).
