# Supplementary material for: Synthetic hybrids of six yeast species
Source: Nat Commun. 2020 Apr 29;11:2085. doi: 10.1038/s41467-020-15559-4 (PMC7190663; doi:10.1038/s41467-020-15559-4)
Supplement: Supplementary file 13 — Reporting Summary [file 41467_2020_15559_MOESM13_ESM.pdf]

nature research

Corresponding author(s): David Peris & Chris Todd Hittinger
NCOMMS-19-16771A

Last updated by author(s): Jan, 29,2020

Reporting Summary

Nature Research wishes to improve the reproducibility of the work that we publish. This form provides structure for consistency and transparency in reporting. For further information on Nature Research policies, see Authors & Referees and the Editorial Policy Checklist .

Statistics

For all statistical analyses, confirm that the following items are present in the figure legend, table legend, main text, or Methods section.

n/a	Confirmed	
☐	☒	The exact sample size (*n*) for each experimental group/condition, given as a discrete number and unit of measurement
☐	☒	A statement on whether measurements were taken from distinct samples or whether the same sample was measured repeatedly
☐	☒	The statistical test(s) used AND whether they are one- or two-sided<br>*Only common tests should be described solely by name; describe more complex techniques in the Methods section.*
☒	☐	A description of all covariates tested
☒	☐	A description of any assumptions or corrections, such as tests of normality and adjustment for multiple comparisons
☐	☒	A full description of the statistical parameters including central tendency (e.g. means) or other basic estimates (e.g. regression coefficient) AND variation (e.g. standard deviation) or associated estimates of uncertainty (e.g. confidence intervals)
☐	☒	For null hypothesis testing, the test statistic (e.g. *F*, *t*, *r*) with confidence intervals, effect sizes, degrees of freedom and *P* value noted<br>*Give P values as exact values whenever suitable.*
☒	☐	For Bayesian analysis, information on the choice of priors and Markov chain Monte Carlo settings
☒	☐	For hierarchical and complex designs, identification of the appropriate level for tests and full reporting of outcomes
☒	☐	Estimates of effect sizes (e.g. Cohen's *d*, Pearson's *r*), indicating how they were calculated

Our web collection on statistics for biologists contains articles on many of the points above.

Software and code

Policy information about availability of computer code

Data collection	Attune NxT<br>BMG FLUOstar Omega<br>Evos<br>Illumina MiSeq
Data analysis	bedtools v2.2.27<br>FIJI<br>FlowJo v 10.4.2<br>GCAT v6.3<br>HybPiper v1.2<br>iWGS v1.1<br>samtools v1.4<br>R and RStudio - packages: dplyr, ggplot2, ggpubr, pheatmap, reshape2<br>sppIDer v1

For manuscripts utilizing custom algorithms or software that are central to the research but not yet described in published literature, software must be made available to editors/reviewers. We strongly encourage code deposition in a community repository (e.g. GitHub). See the Nature Research guidelines for submitting code & software for further information.

Data

Policy information about availability of data

All manuscripts must include a data availability statement. This statement should provide the following information, where applicable:

- Accession codes, unique identifiers, or web links for publicly available datasets
- A list of figures that have associated raw data
- A description of any restrictions on data availability

Data availability: curated raw reads were submitted to the SRA database as Bioproject PRJNA476226.
Source data are provided at http://bit.ly/2v1rq1T.

Field-specific reporting

Please select the one below that is the best fit for your research. If you are not sure, read the appropriate sections before making your selection.

- [x] Life sciences
- [ ] Behavioural & social sciences
- [ ] Ecological, evolutionary & environmental sciences

For a reference copy of the document with all sections, see nature.com/documents/nr-reporting-summary-flat.pdf

Life sciences study design

All studies must disclose on these points even when the disclosure is negative.

Sample size	Sample size was not determined using statistical methods. We chose to perform three ALE replicates of two independently generated six-species hybrids.
Data exclusions	A six-species hybrid was excluded because it lost S. cerevisiae chromosome IV, which is where Scheffersomyces (Pichia) stipitis xylose utilization genes had been inserted, and the experiment was performed in xylose-containing media.
Replication	We evolved our two new six-species hybrids in three independent cultures of YPX or YPD to confirm our independently generated six-species hybrids improved the fitness under similar conditions.
Randomization	Our six-species hybrids are independent organisms that did not require allocation to particular groups.
Blinding	It was not necessary because strains were not allocated to particular groups.

Reporting for specific materials, systems and methods

We require information from authors about some types of materials, experimental systems and methods used in many studies. Here, indicate whether each material, system or method listed is relevant to your study. If you are not sure if a list item applies to your research, read the appropriate section before selecting a response.

Materials & experimental systems

n/a	Involved in the study	
x		Antibodies
	x	Eukaryotic cell lines
x		Palaeontology
x		Animals and other organisms
x		Human research participants
x		Clinical data

Methods

n/a	Involved in the study	
x		ChIP-seq
	x	Flow cytometry
x		MRI-based neuroimaging

Eukaryotic cell lines

Policy information about cell lines

Cell line source(s)	See Supplementary Table 1.
Authentication	Strains were previously identified using molecular methods, and references to those strains are included in the Supplementary Table 1.
Mycoplasma contamination	*Confirm that all cell lines tested negative for mycoplasma contamination OR describe the results of the testing for mycoplasma contamination OR declare that the cell lines were not tested for mycoplasma contamination.*
Commonly misidentified lines (See ICLAC register)	*Name any commonly misidentified cell lines used in the study and provide a rationale for their use.*

Flow Cytometry

Plots

Confirm that:

- [x] The axis labels state the marker and fluorochrome used (e.g. CD4-FITC).
- [x] The axis scales are clearly visible. Include numbers along axes only for bottom left plot of group (a 'group' is an analysis of identical markers).
- [x] All plots are contour plots with outliers or pseudocolor plots.
- [x] A numerical value for number of cells or percentage (with statistics) is provided.

Methodology

Sample preparation	Both asynchronous and hydroxyurea-arrested (G1/S arrested) mid-log cultures were prepared for each strain. Hydroxyura-arrested strains were prepared to assist in the identification of G1 peaks in samples with broad and undefined cell cycle peaks. Briefly, cultures were grown to saturation and then diluted back 1:200. Back-diluted cultures were then grown to an OD of 0.4-0.6. For each strain, one ml mid-log culture was transferred into 200 µl 1M hydroxyurea and incubated on a room temperature roller drum for approximately half the time the respective strain took to grow from back dilution to an OD of 0.4-0.6. This ranged between 3 and 12 hours. At the same time, one ml of asynchronous mid-log culture was harvested for fixation. All samples were fixed in 70% ethanol overnight, treated with RNase and Proteinase K, and finally stained with Sytox Green (Molecular Probes). Stained cell suspensions were sonicated before flow cytometry.
Instrument	Attune NxT
Software	FlowJo v 10.4.2
Cell population abundance	Sorting was not performed
Gating strategy	Samples were first gated on SSC and FSC to remove debris. Doublets were then removed by gating on BL1-A and FSC-A. A histogram of BL1-A values were then generated for remaining cells. Hydroxyurea peaks were identified and gated manually. Asynchronous G1 and G2 peaks were identified by applying a Watson (Pragmatic) Cell Cycle model and identifying G1 and G2 means. When cell cycle models did not fit the asynchronous sample data automatically, hydroxyura samples were used to identify G1 peaks and these were manually gated to constrain G1 in asynchrounous samples.

- [x] Tick this box to confirm that a figure exemplifying the gating strategy is provided in the Supplementary Information.
