# Supplementary material for: Synthetic hybrids of six yeast species
Source: Nat Commun. 2020 Apr 29;11:2085. doi: 10.1038/s41467-020-15559-4 (PMC7190663; doi:10.1038/s41467-020-15559-4)
Supplement: Supplementary file 14 — Description of Additional Supplementary Files [file 41467_2020_15559_MOESM14_ESM.pdf]

Title: Supplementary Data 1.
Description: Wild, engineered, and synthetic hybrid strain information. Genotypic information of selected *Saccharomyces* species for generating synthetic hybrids.

Title: Supplementary Data 2.
Description: Ploidy and genome size estimation.

Title: Supplementary Data 3.
Description: Frequency of successful crosses.

Title: Supplementary Data 4.
Description: Kinetic parameters for parent strains and synthetic hybrids.

Title: Supplementary Data 5.
Description: Kinetic parameter information for the reference strain, ancestor synthetic hybrids, and evolved synthetic hybrids after colony selection on YPX plates.

Title: Supplementary Data 6.
Description: Kinetic parameter information for the reference strain and synthetic hybrids.

Title: Supplementary Data 7.
Description: Coverage calculations for chromosome IV and the xylose cassette.

Title: Supplementary Data 8.
Description: Plasmid descriptions.

Title: Supplementary Data 9.
Description:PCR primers used to confirm mating-type switching and hybridization.

Title: Supplementary Data 10.
Description:PCR-RFLP of expected *Saccharomyces* bands.

Title: Supplementary Data 11.
Description: Summary of whole genome sequencing statistics.
