# Supplementary material for: Synthetic hybrids of six yeast species
Source: Nat Commun. 2020 Apr 29;11:2085. doi: 10.1038/s41467-020-15559-4 (PMC7190663; doi:10.1038/s41467-020-15559-4)
Supplement: Supplementary file 15 — Source Data [file 41467_2020_15559_MOESM15_ESM.zip › Source Data 2.zip!/yHRWh4_sppIDerDepthPlot-covBins.pdf]

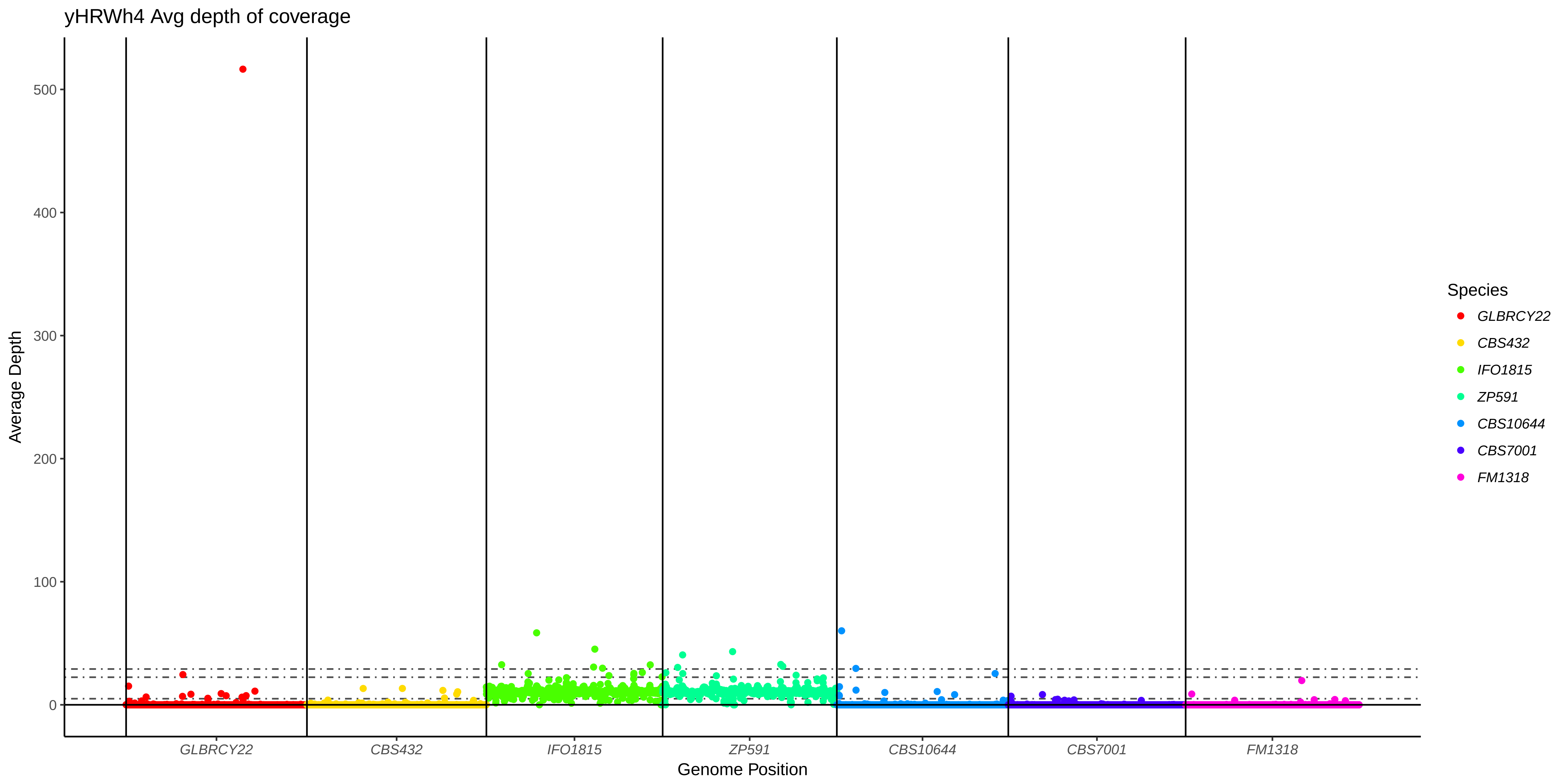
yHRWh4 Avg depth of coverage
Average Depth
500
400
300
200
100
0
GLBRCY22
CBS432
IFO1815
ZP591
CBS10644
CBS7001
FM1318
Genome Position
Species
GLBRCY22
CBS432
IFO1815
ZP591
CBS10644
CBS7001
FM1318

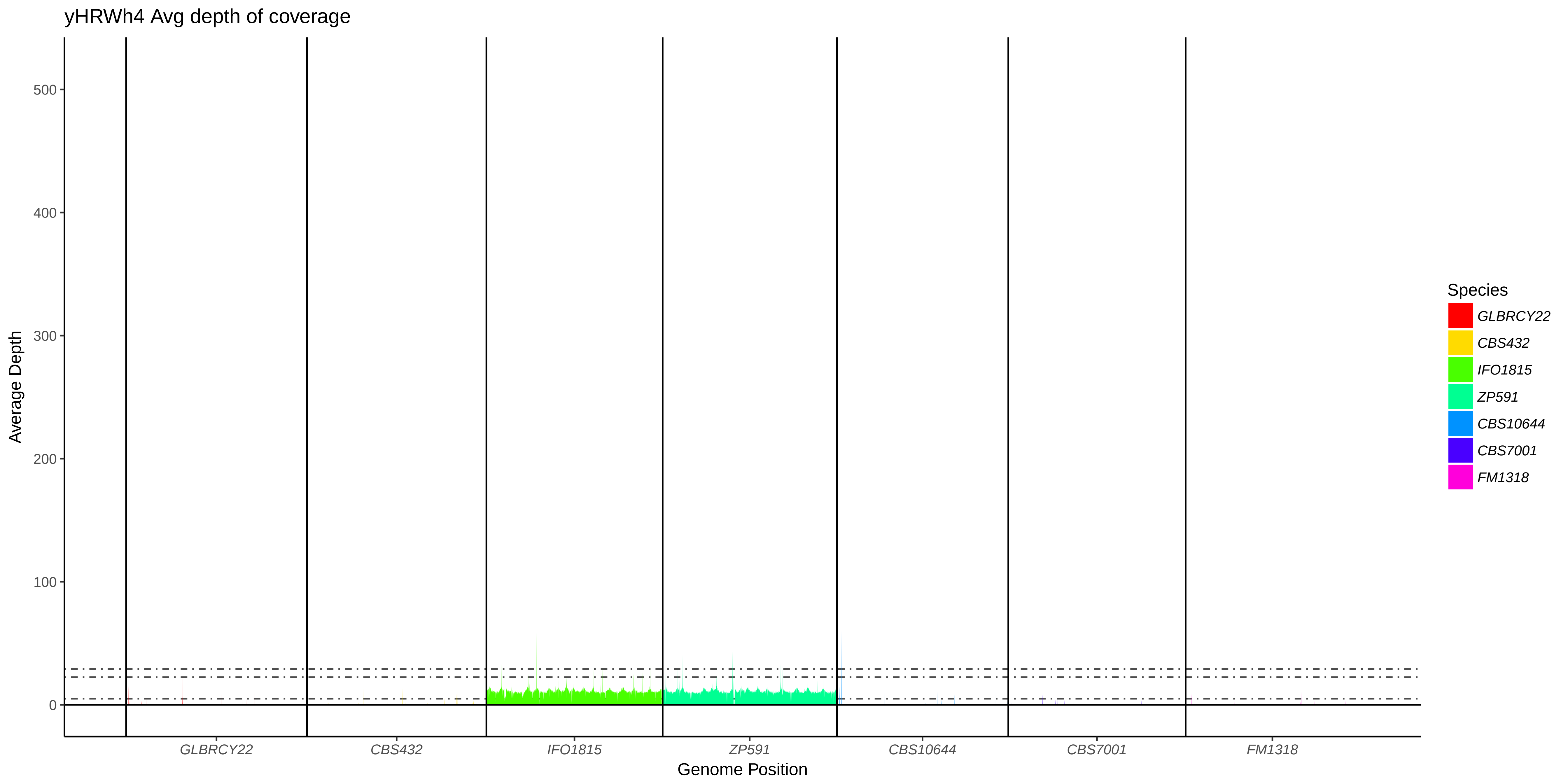

yHRWh4 Avg depth of coverage
Average Depth
500
400
300
200
100
0
GLBRCY22
CBS432
IFO1815
ZP591
CBS10644
CBS7001
FM1318
Genome Position
Species
GLBRCY22
CBS432
IFO1815
ZP591
CBS10644
CBS7001
FM1318

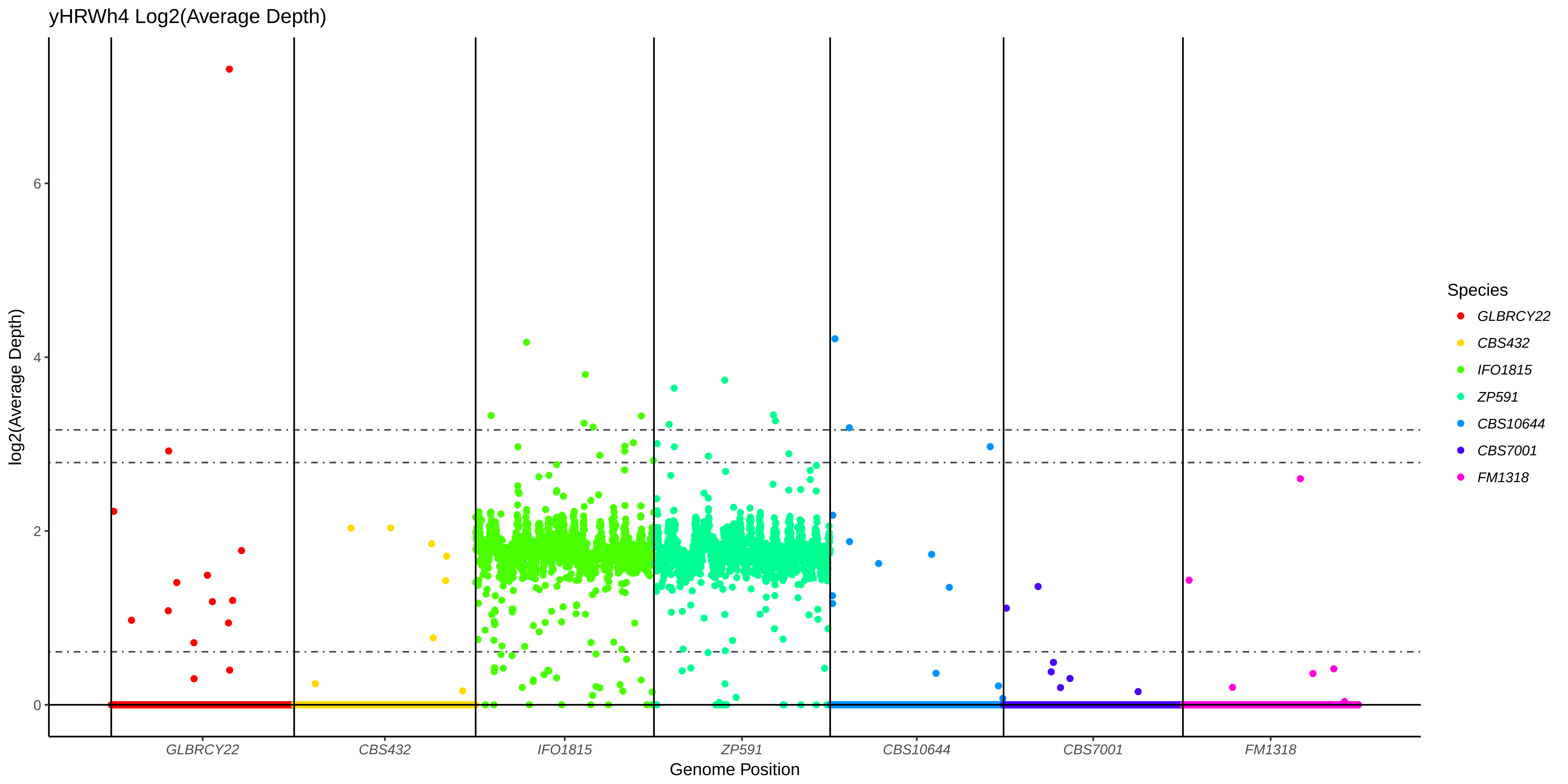

yHRWh4 Log2(Average Depth)
log2(Average Depth)
0
2
4
6
GLBRCY22
CBS432
IFO1815
ZP591
CBS10644
CBS7001
FM1318
Genome Position
Species
GLBRCY22
CBS432
IFO1815
ZP591
CBS10644
CBS7001
FM1318

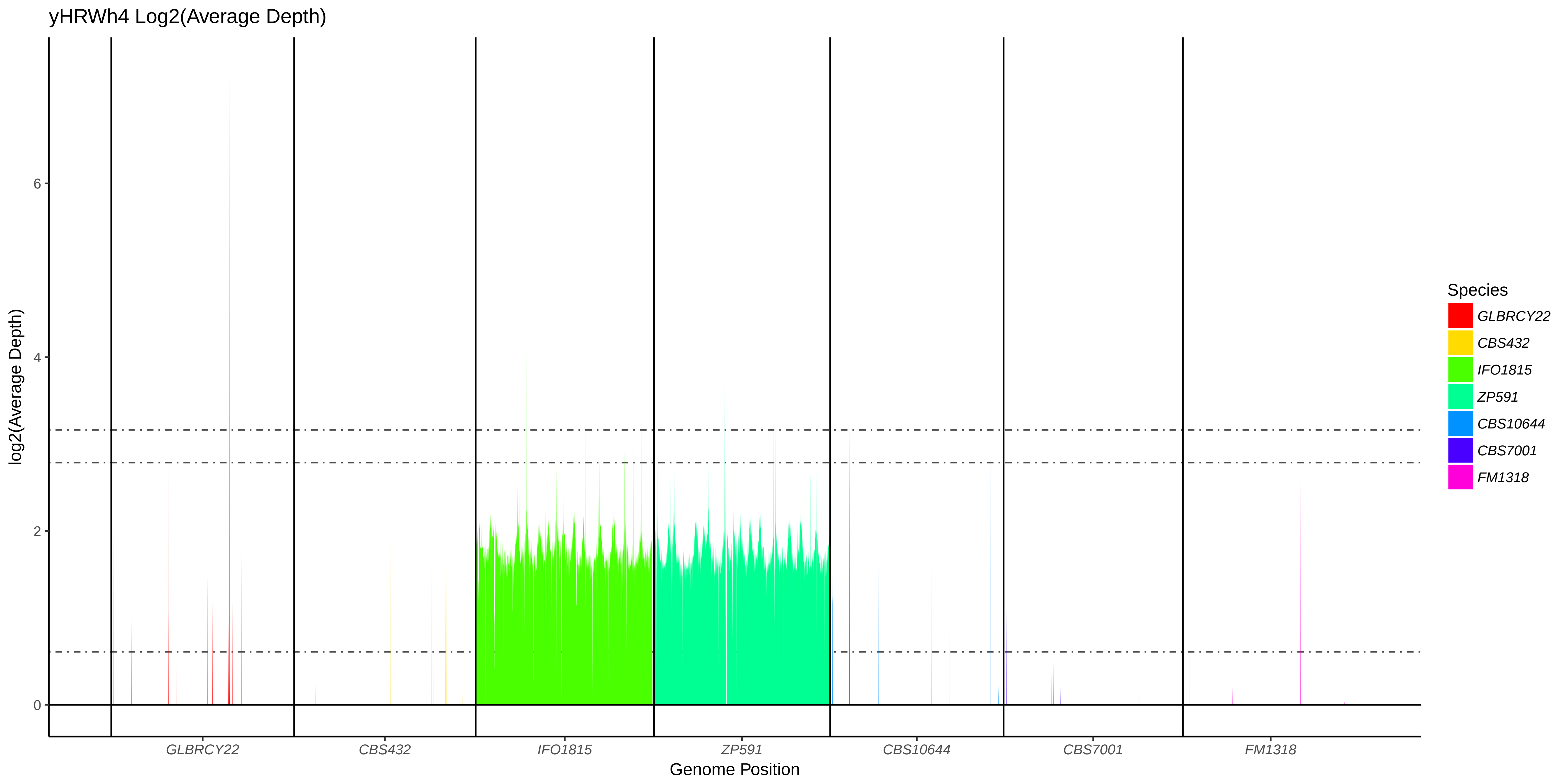

yHRWh4 Log2(Average Depth)
log2(Average Depth)
0
2
4
6
GLBRCY22
CBS432
IFO1815
ZP591
CBS10644
CBS7001
FM1318
Genome Position
Species
GLBRCY22
CBS432
IFO1815
ZP591
CBS10644
CBS7001
FM1318

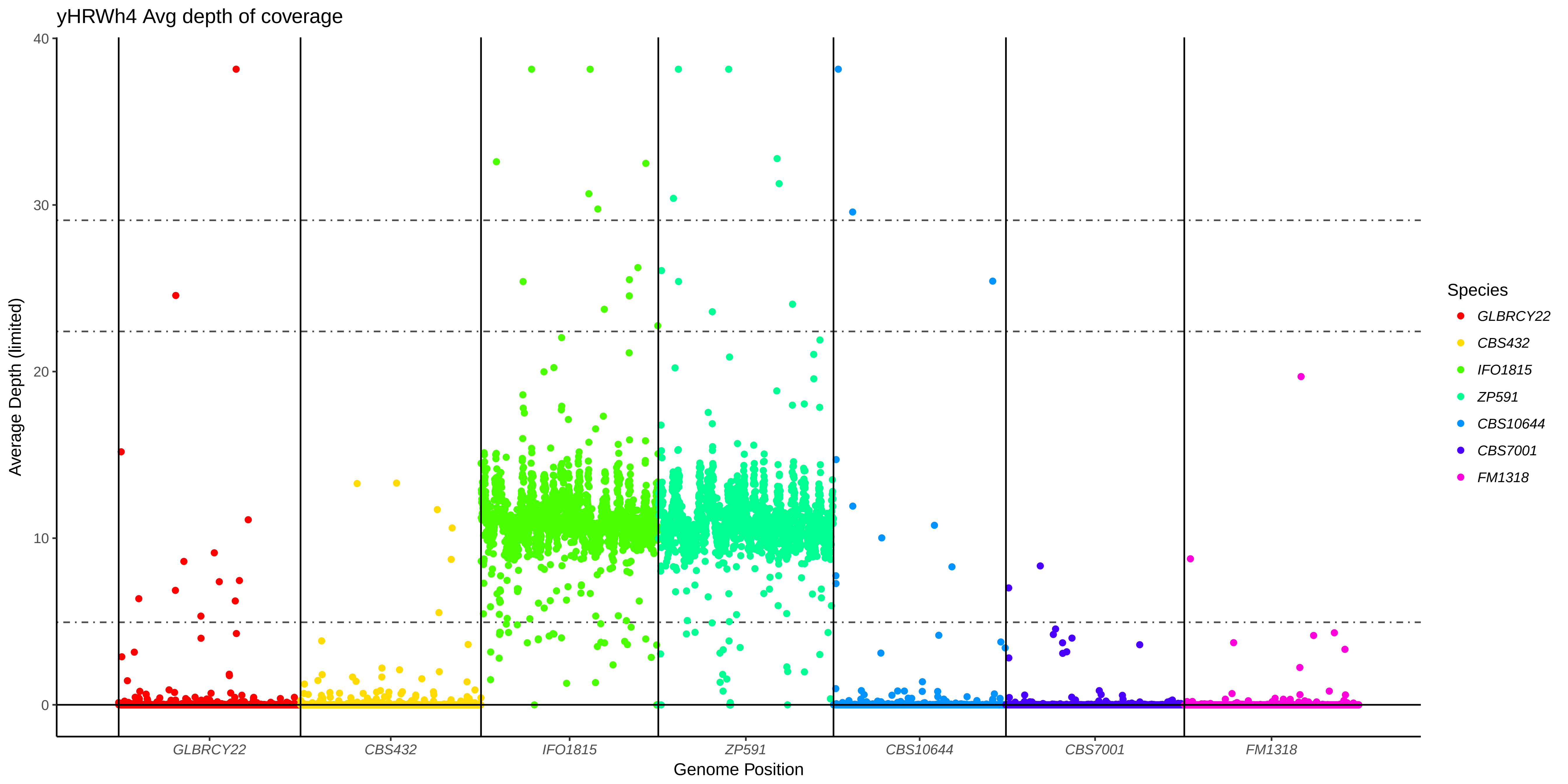

yHRWh4 Avg depth of coverage
Average Depth (limited)
40
30
20
10
0
GLBRCY22
CBS432
IFO1815
ZP591
CBS10644
CBS7001
FM1318
Genome Position
Species
GLBRCY22
CBS432
IFO1815
ZP591
CBS10644
CBS7001
FM1318

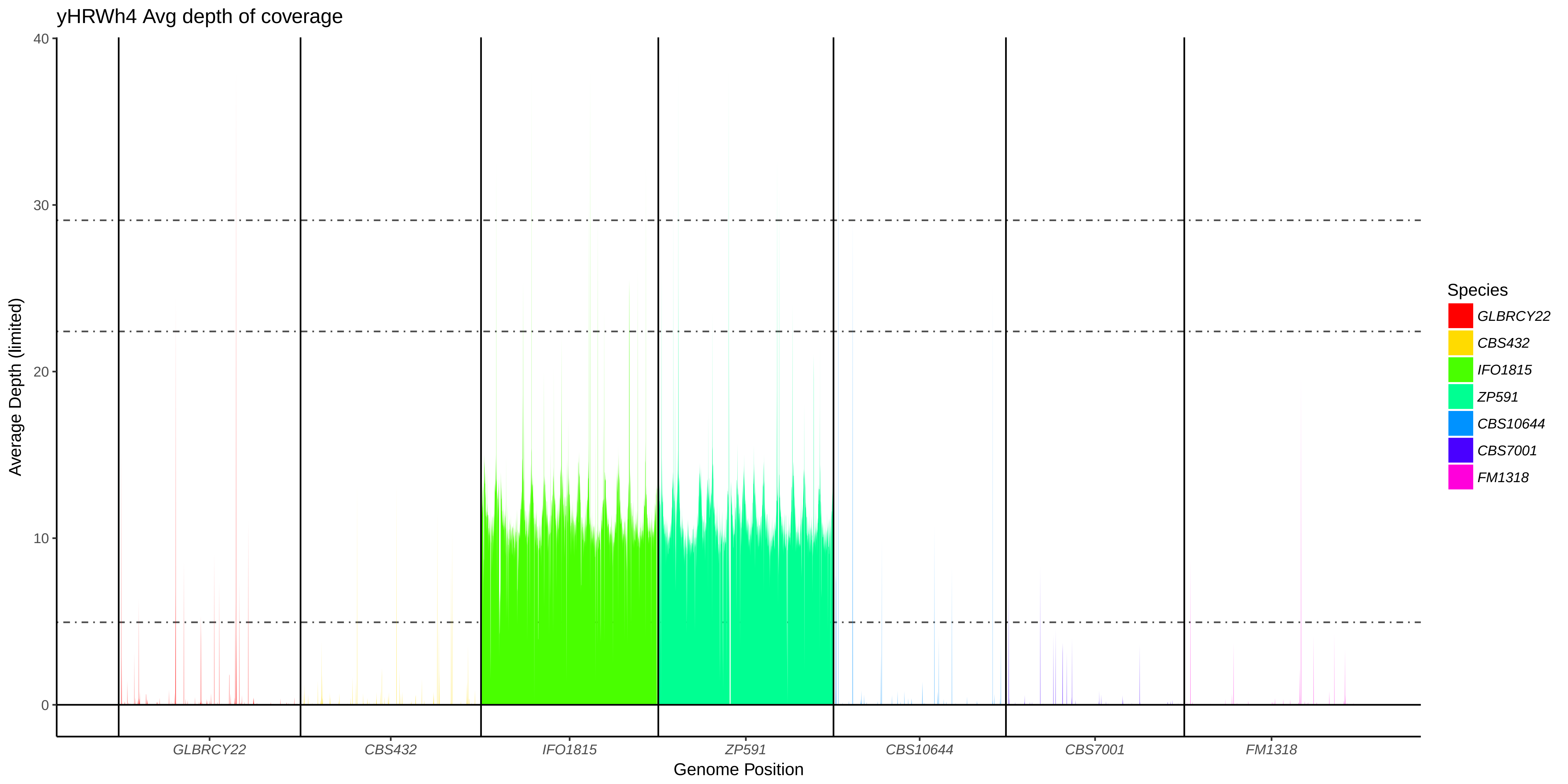

yHRWh4 Avg depth of coverage
Average Depth (limited)
0
10
20
30
40
GLBRCY22
CBS432
IFO1815
ZP591
CBS10644
CBS7001
FM1318
Genome Position
Species
GLBRCY22
CBS432
IFO1815
ZP591
CBS10644
CBS7001
FM1318

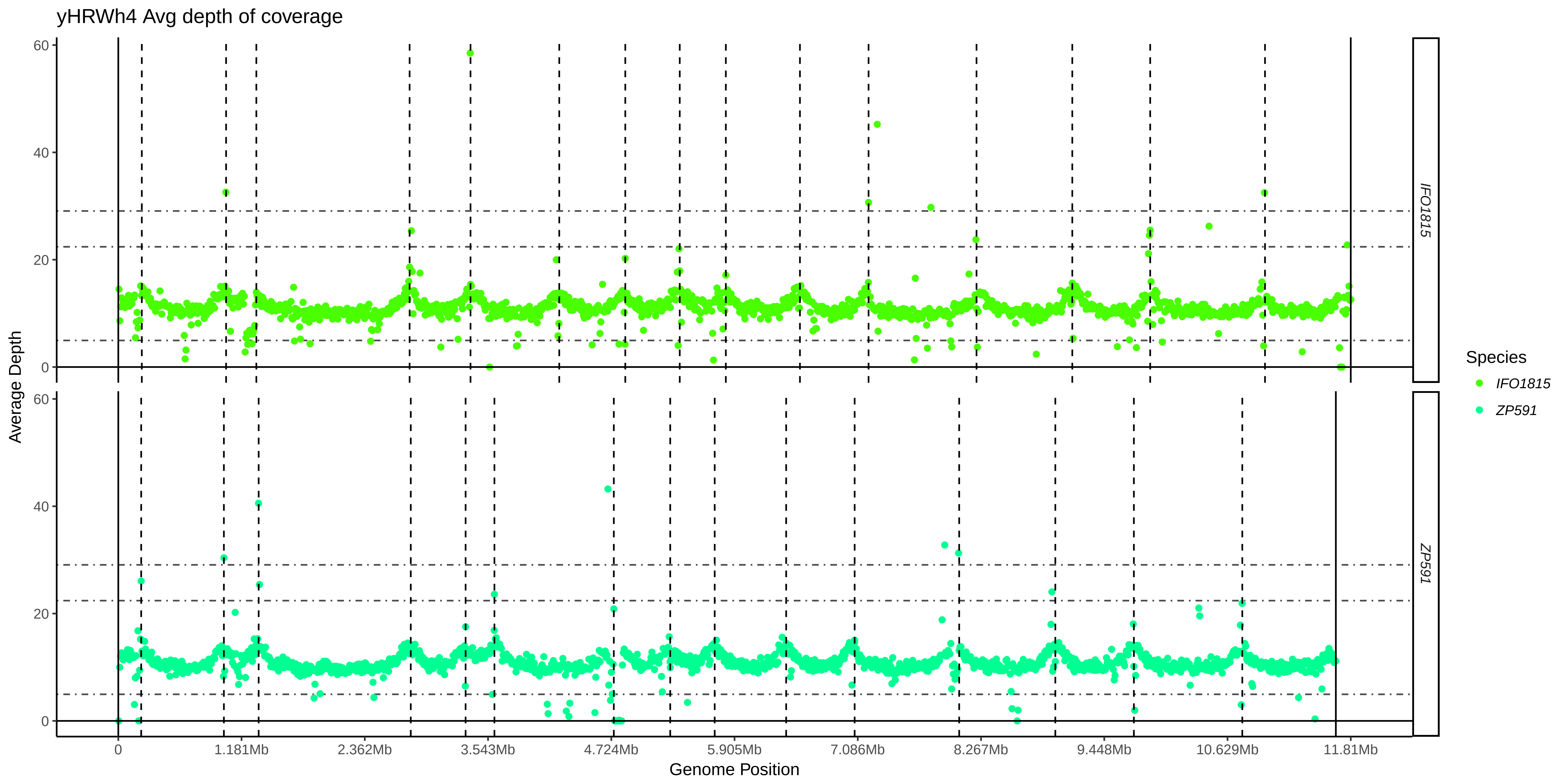
yHRWh4 Avg depth of coverage
Average Depth
60
40
20
0
IFO1815
ZP591
0
1.181Mb
2.362Mb
3.543Mb
4.724Mb
5.905Mb
7.086Mb
8.267Mb
9.448Mb
10.629Mb
11.81Mb
Genome Position
Species
IFO1815
ZP591

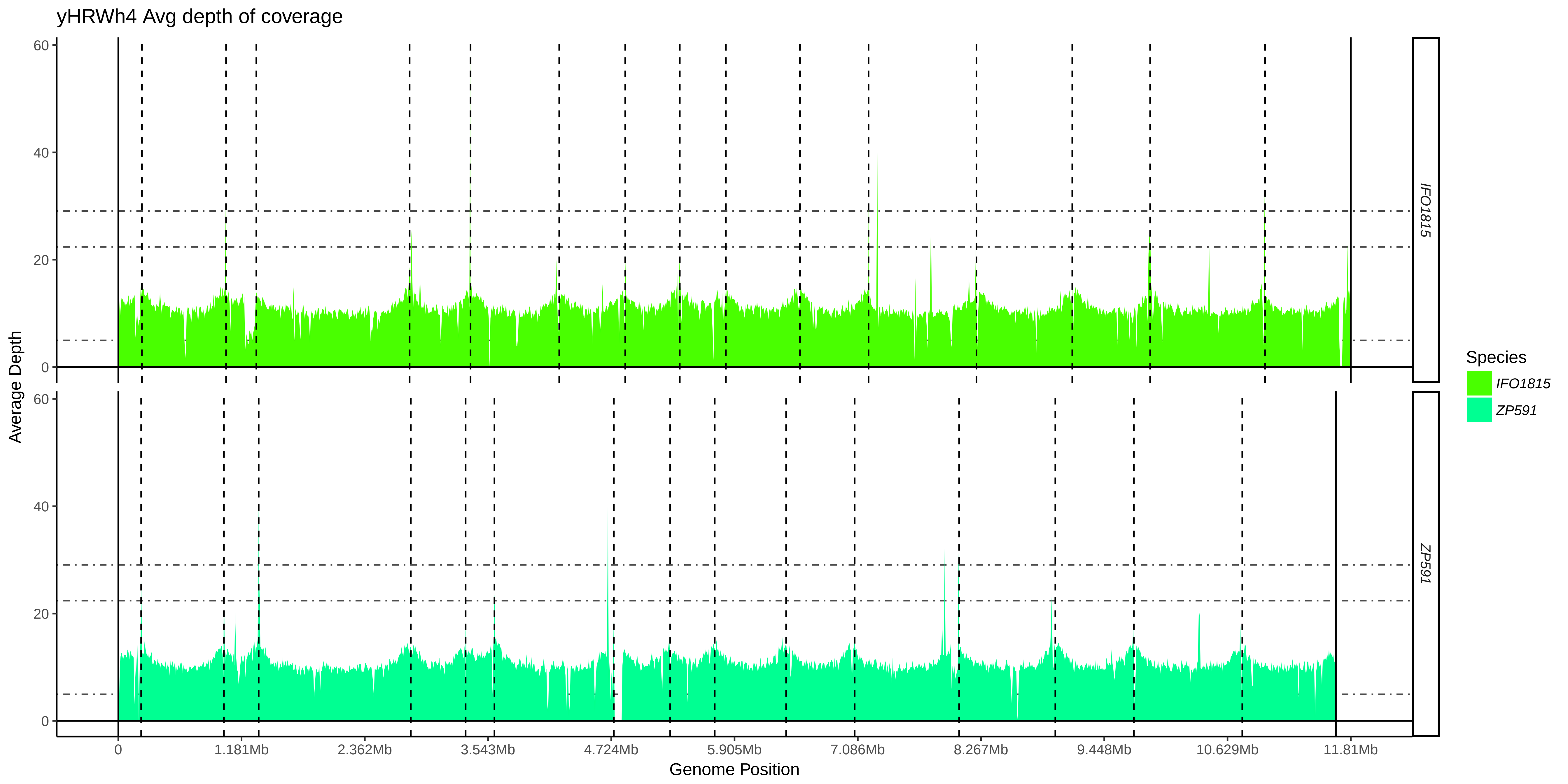
yHRWh4 Avg depth of coverage
Average Depth
60
40
20
0
Genome Position
0
1.181Mb
2.362Mb
3.543Mb
4.724Mb
5.905Mb
7.086Mb
8.267Mb
9.448Mb
10.629Mb
11.81Mb
IFO1815
ZP591
Species
IFO1815
ZP591

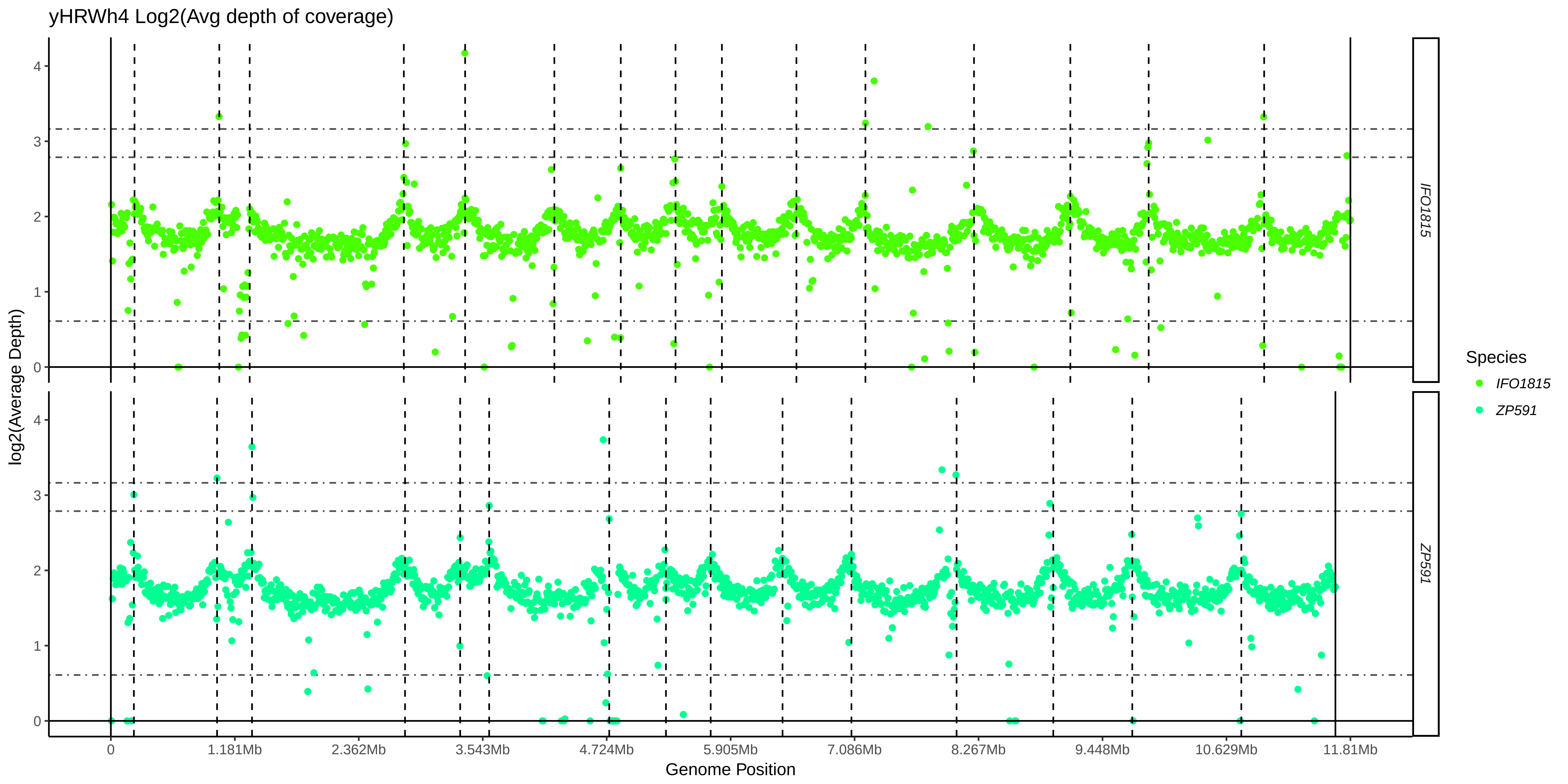

yHRWh4 Log2(Avg depth of coverage)
log2(Average Depth)
Genome Position
0
1.181Mb
2.362Mb
3.543Mb
4.724Mb
5.905Mb
7.086Mb
8.267Mb
9.448Mb
10.629Mb
11.81Mb
IFO1815
ZP591
Species

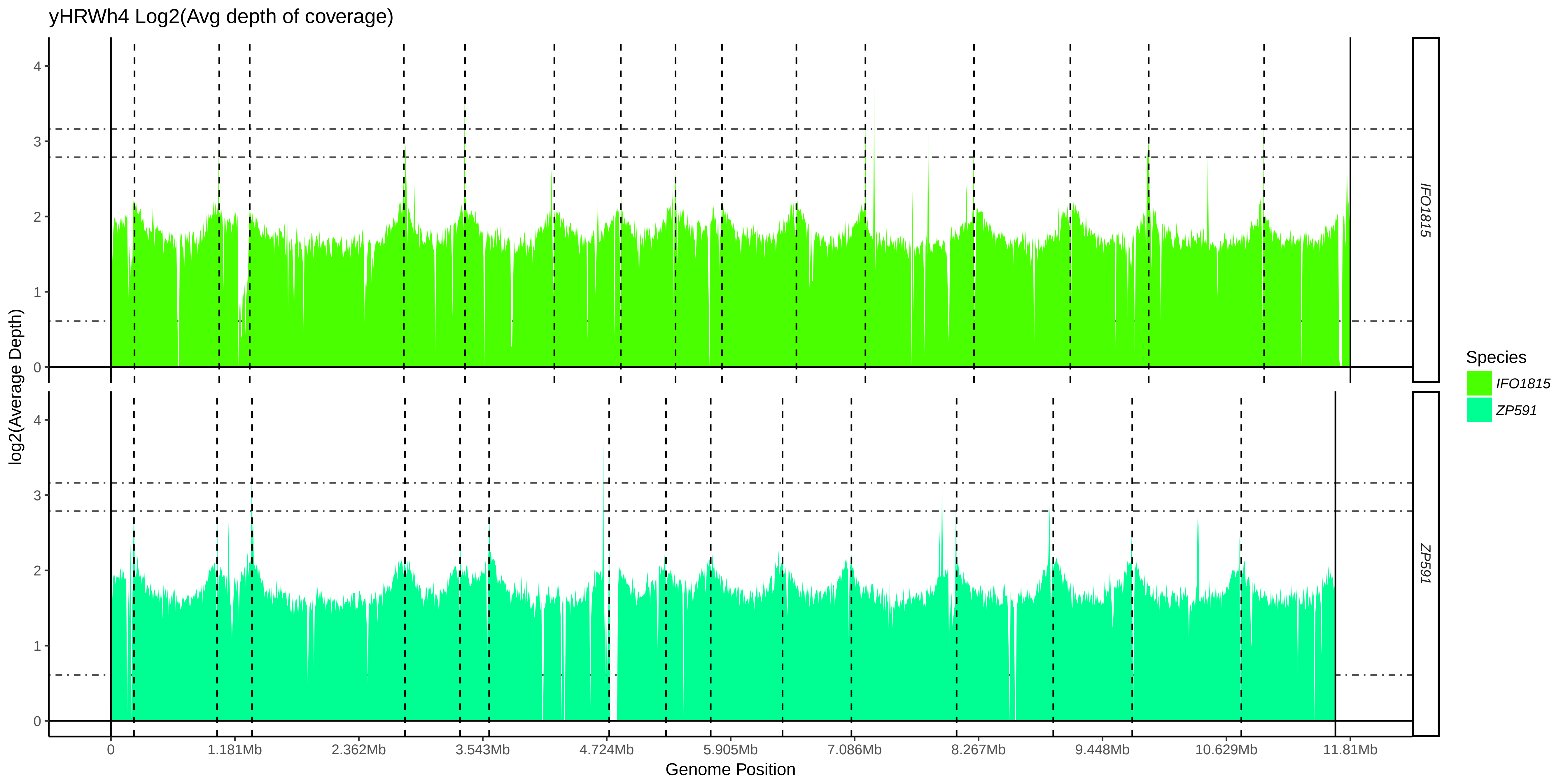

yHRWh4 Log2(Avg depth of coverage)
log2(Average Depth)
Genome Position
0
1.181Mb
2.362Mb
3.543Mb
4.724Mb
5.905Mb
7.086Mb
8.267Mb
9.448Mb
10.629Mb
11.81Mb
IFO1815
ZP591
Species
IFO1815
ZP591

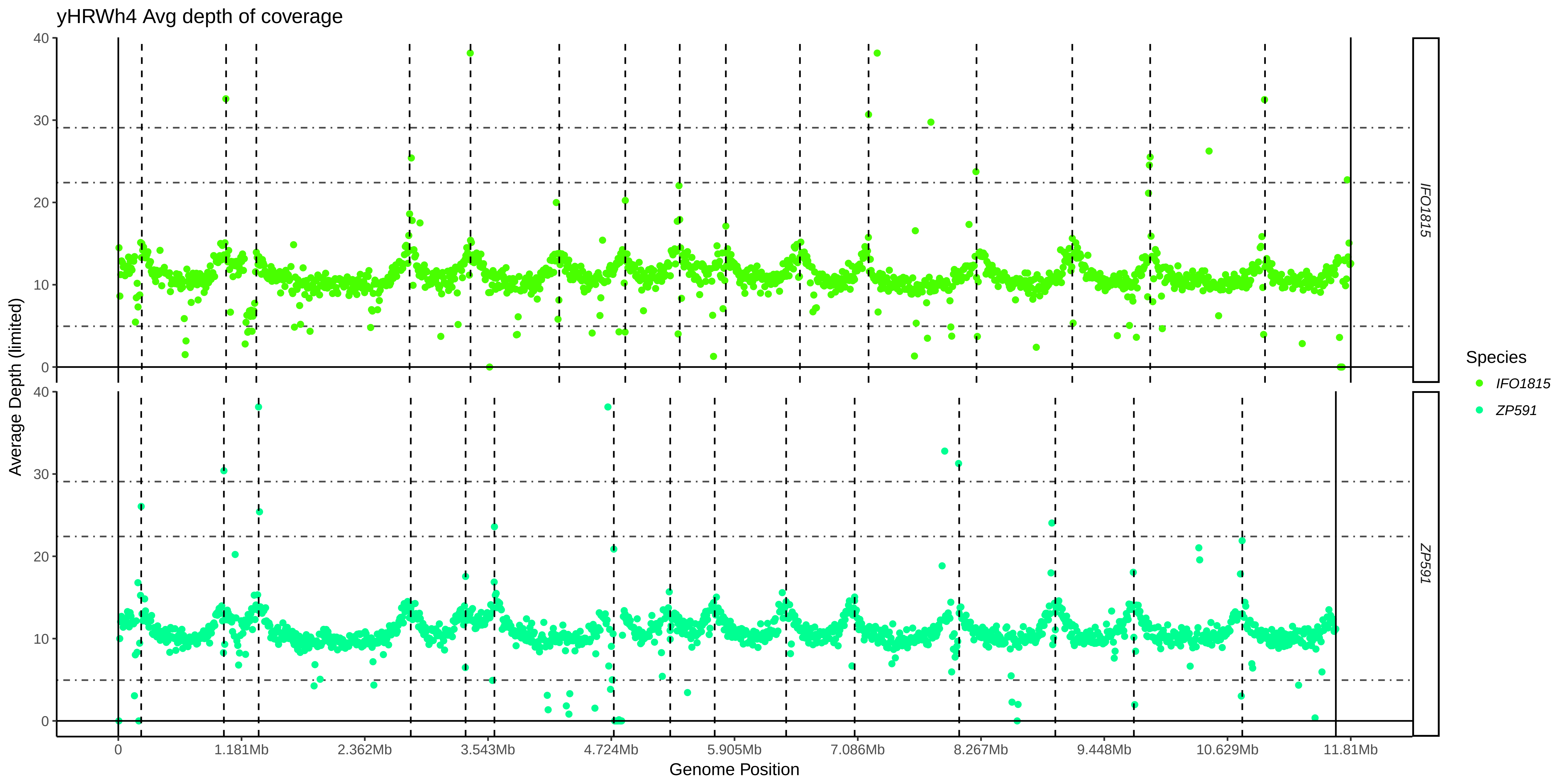

yHRWh4 Avg depth of coverage
Average Depth (limited)
Genome Position
0
1.181Mb
2.362Mb
3.543Mb
4.724Mb
5.905Mb
7.086Mb
8.267Mb
9.448Mb
10.629Mb
11.81Mb
0
10
20
30
40
IFO1815
ZP591
Species
IFO1815
ZP591

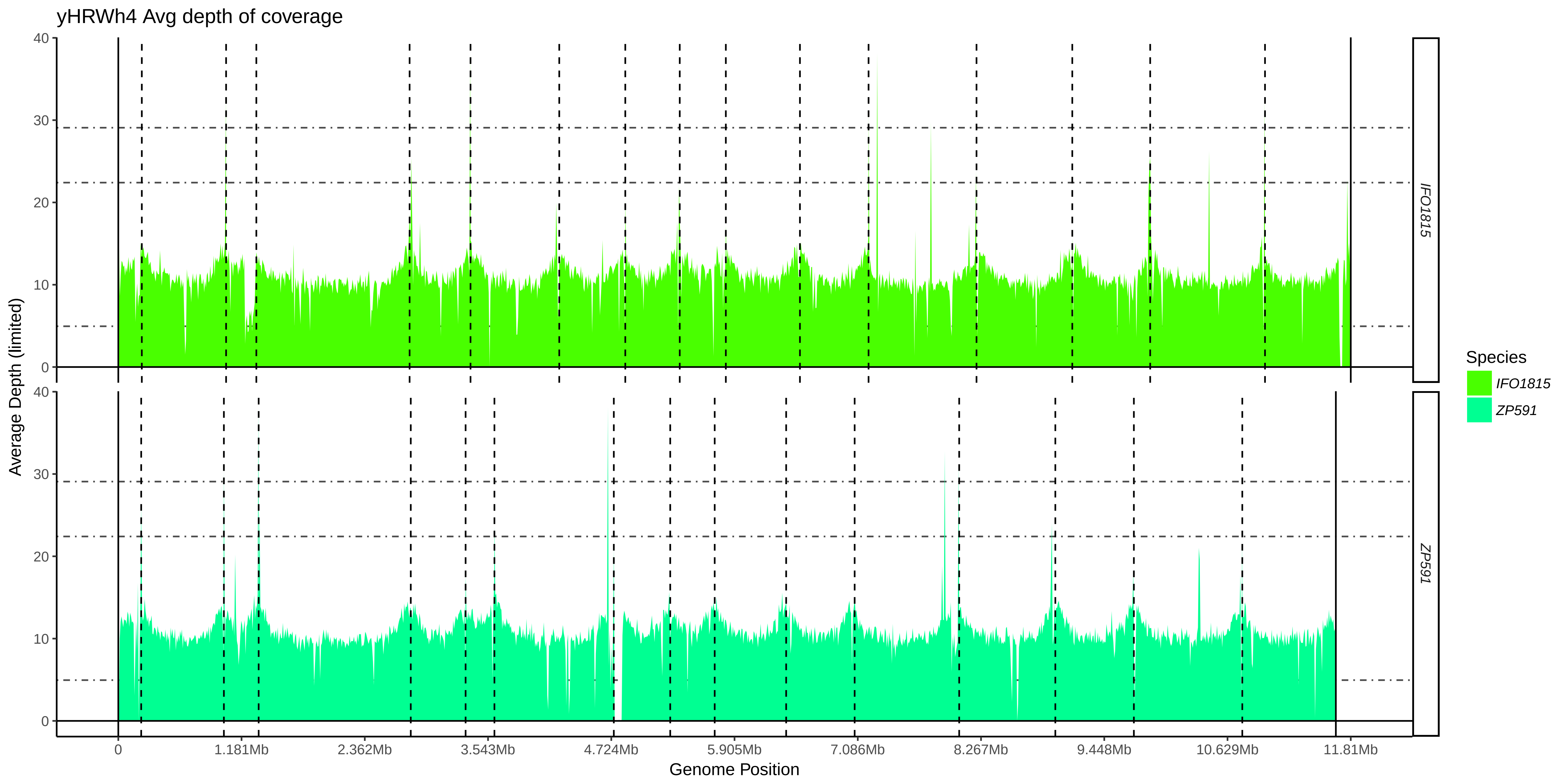
yHRWh4 Avg depth of coverage
Average Depth (limited)
0
10
20
30
40
IFO1815
ZP591
Species
IFO1815
ZP591
0
1.181Mb
2.362Mb
3.543Mb
4.724Mb
5.905Mb
7.086Mb
8.267Mb
9.448Mb
10.629Mb
11.81Mb
Genome Position
