# Supplementary material for: Synthetic hybrids of six yeast species
Source: Nat Commun. 2020 Apr 29;11:2085. doi: 10.1038/s41467-020-15559-4 (PMC7190663; doi:10.1038/s41467-020-15559-4)
Supplement: Supplementary file 15 — Source Data [file 41467_2020_15559_MOESM15_ESM.zip › Source Data 2.zip!/yHRWh8_sppIDerDepthPlot-covBins.pdf]

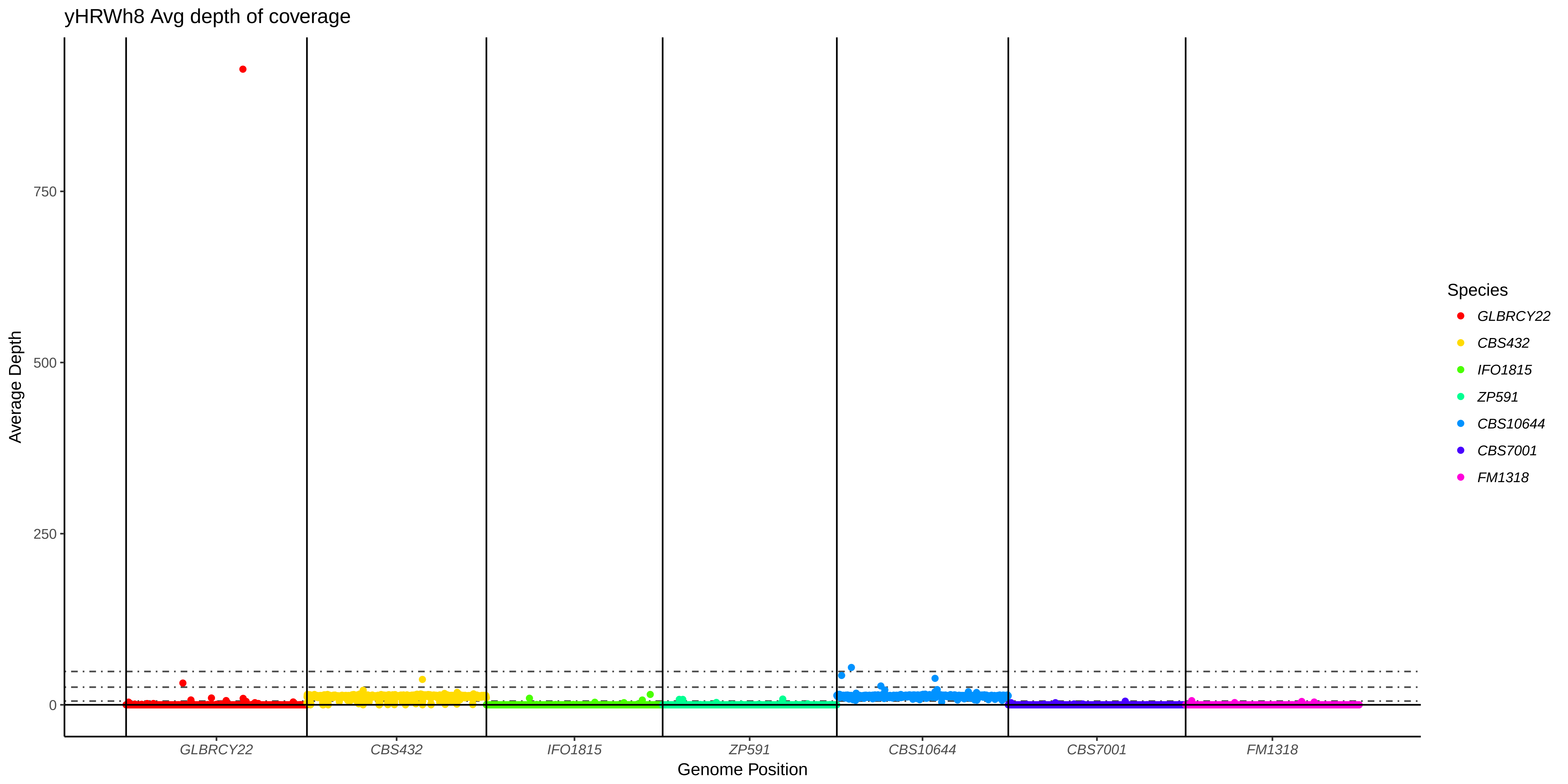

yHRWh8 Avg depth of coverage
Average Depth
750
500
250
0
GLBRCY22
CBS432
IFO1815
ZP591
CBS10644
CBS7001
FM1318
Genome Position
Species
GLBRCY22
CBS432
IFO1815
ZP591
CBS10644
CBS7001
FM1318

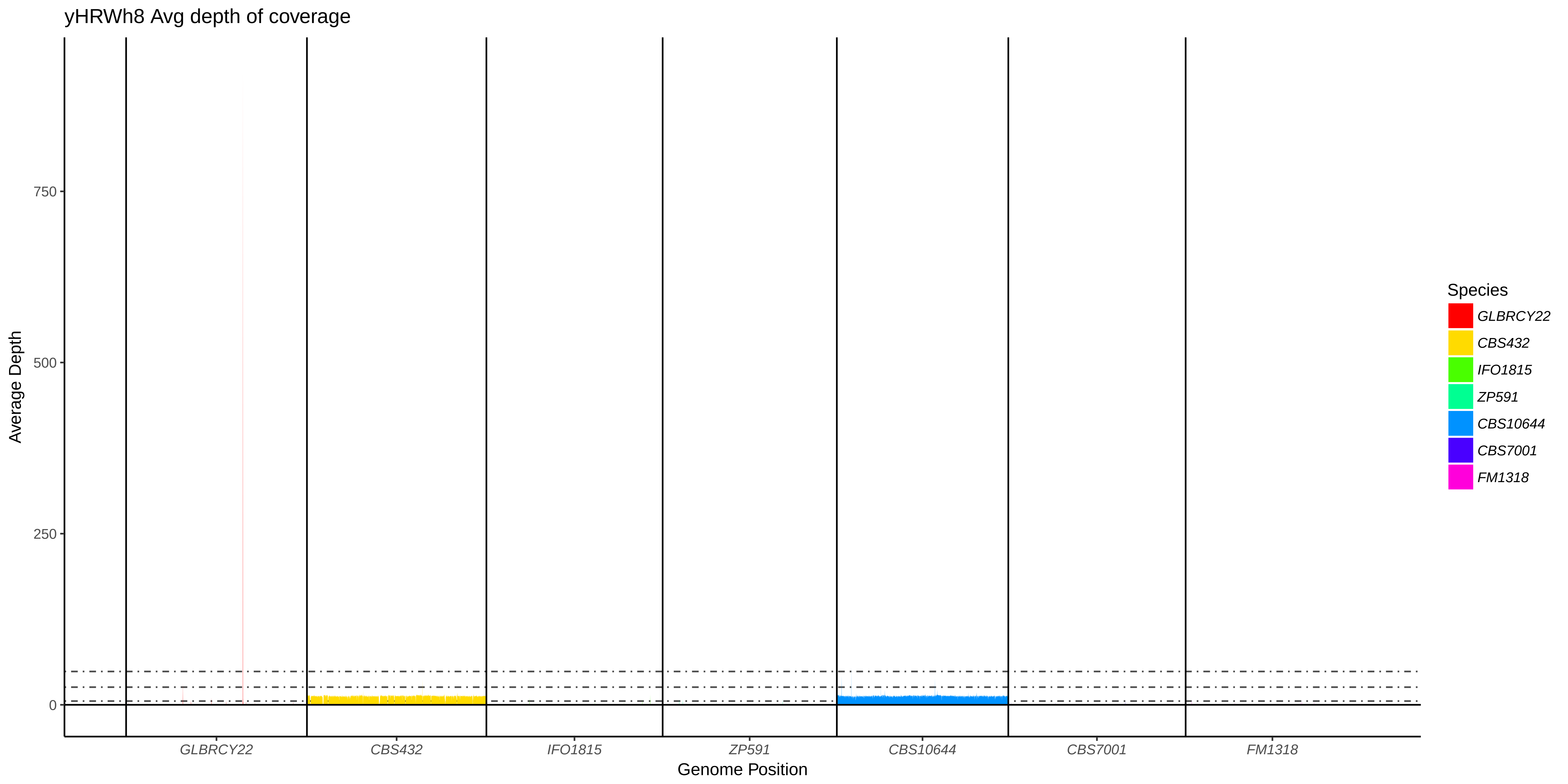

yHRWh8 Avg depth of coverage
Average Depth
750
500
250
0
GLBRCY22
CBS432
IFO1815
ZP591
CBS10644
CBS7001
FM1318
Genome Position
Species
GLBRCY22
CBS432
IFO1815
ZP591
CBS10644
CBS7001
FM1318

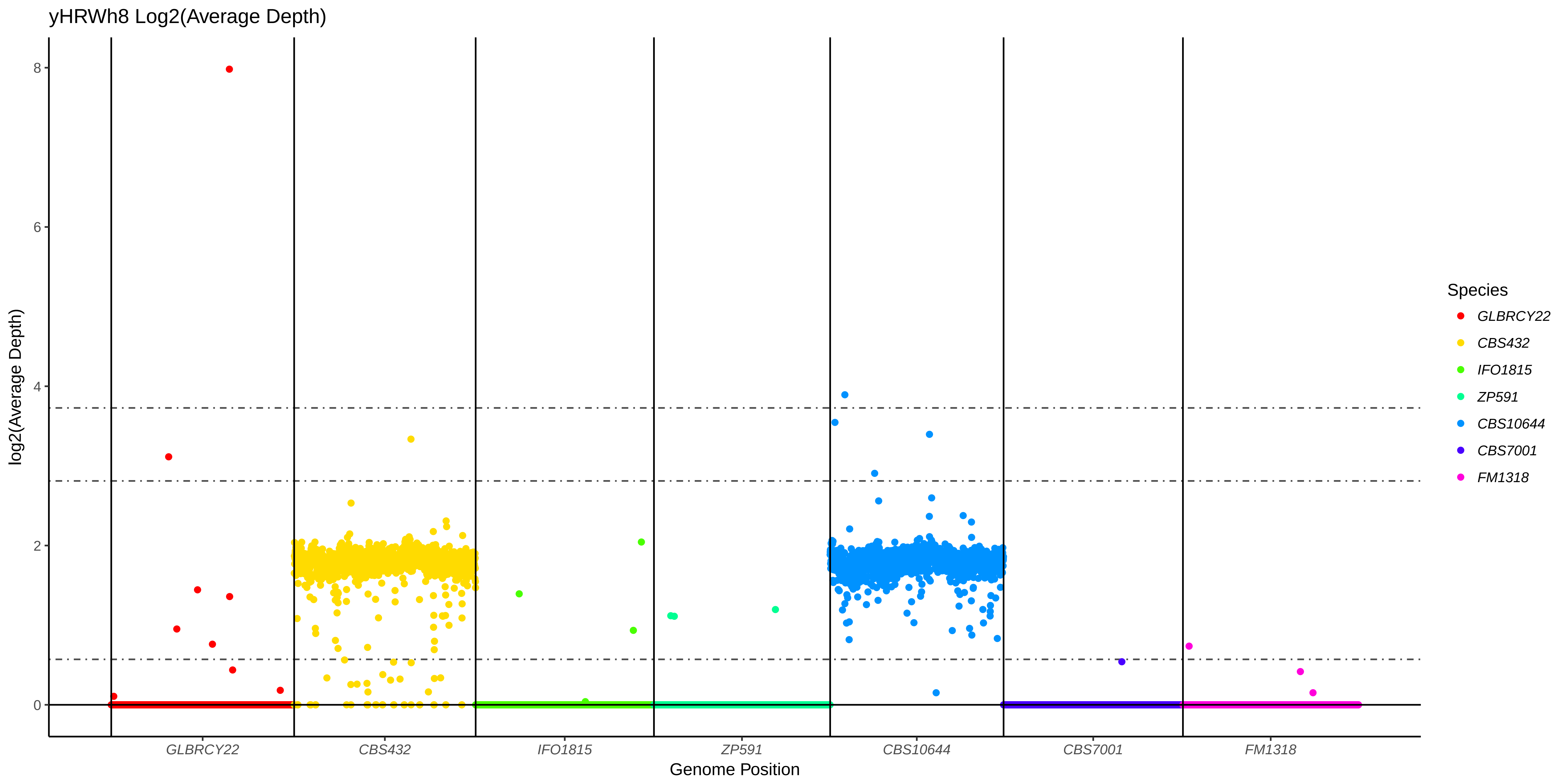

yHRWh8 Log2(Average Depth)
log2(Average Depth)
0
2
4
6
8
GLBRCY22
CBS432
IFO1815
ZP591
CBS10644
CBS7001
FM1318
Genome Position
Species
GLBRCY22
CBS432
IFO1815
ZP591
CBS10644
CBS7001
FM1318

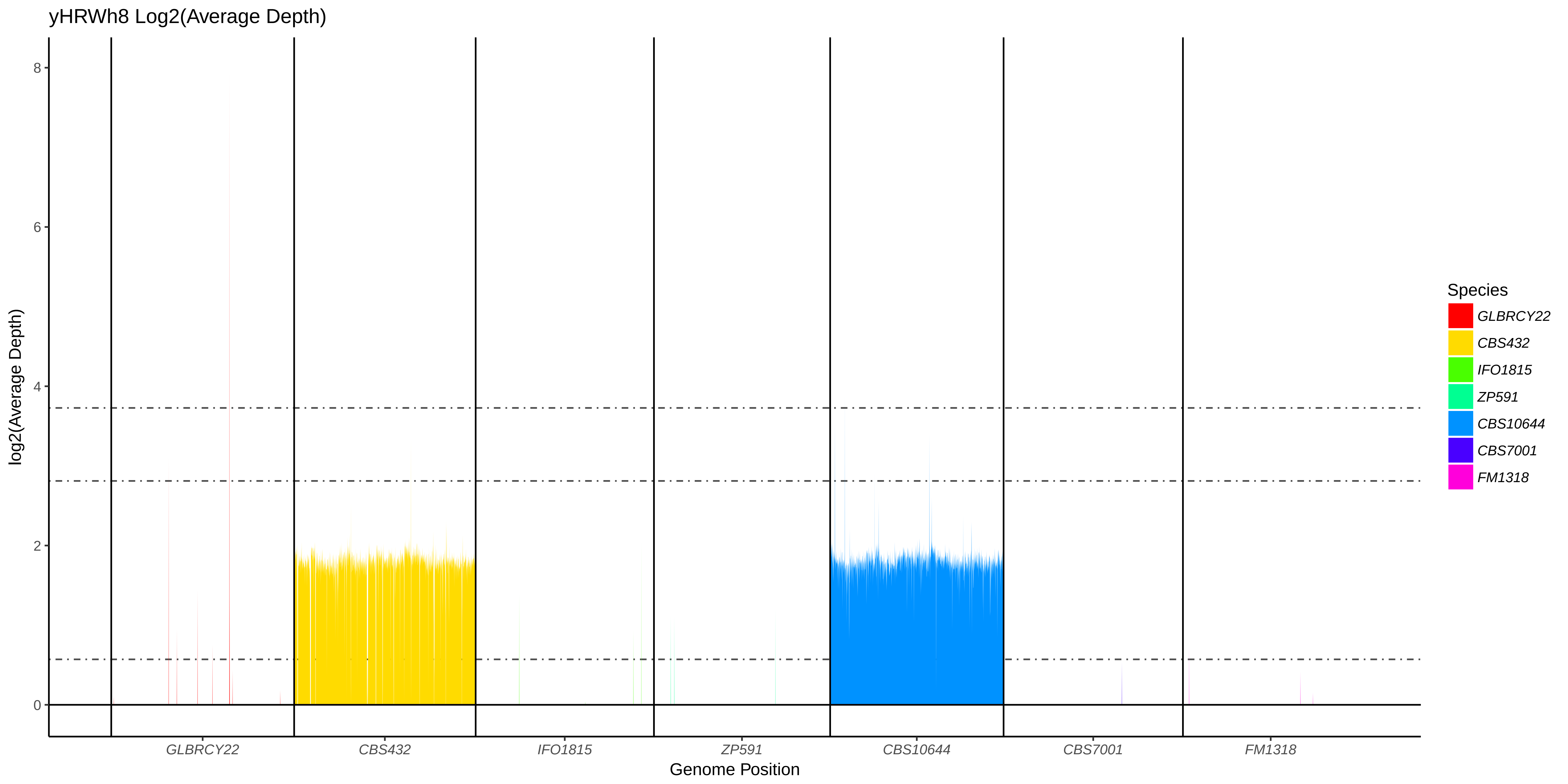

yHRWh8 Log2(Average Depth)
log2(Average Depth)
0
2
4
6
8
GLBRCY22
CBS432
IFO1815
ZP591
CBS10644
CBS7001
FM1318
Genome Position
Species
GLBRCY22
CBS432
IFO1815
ZP591
CBS10644
CBS7001
FM1318

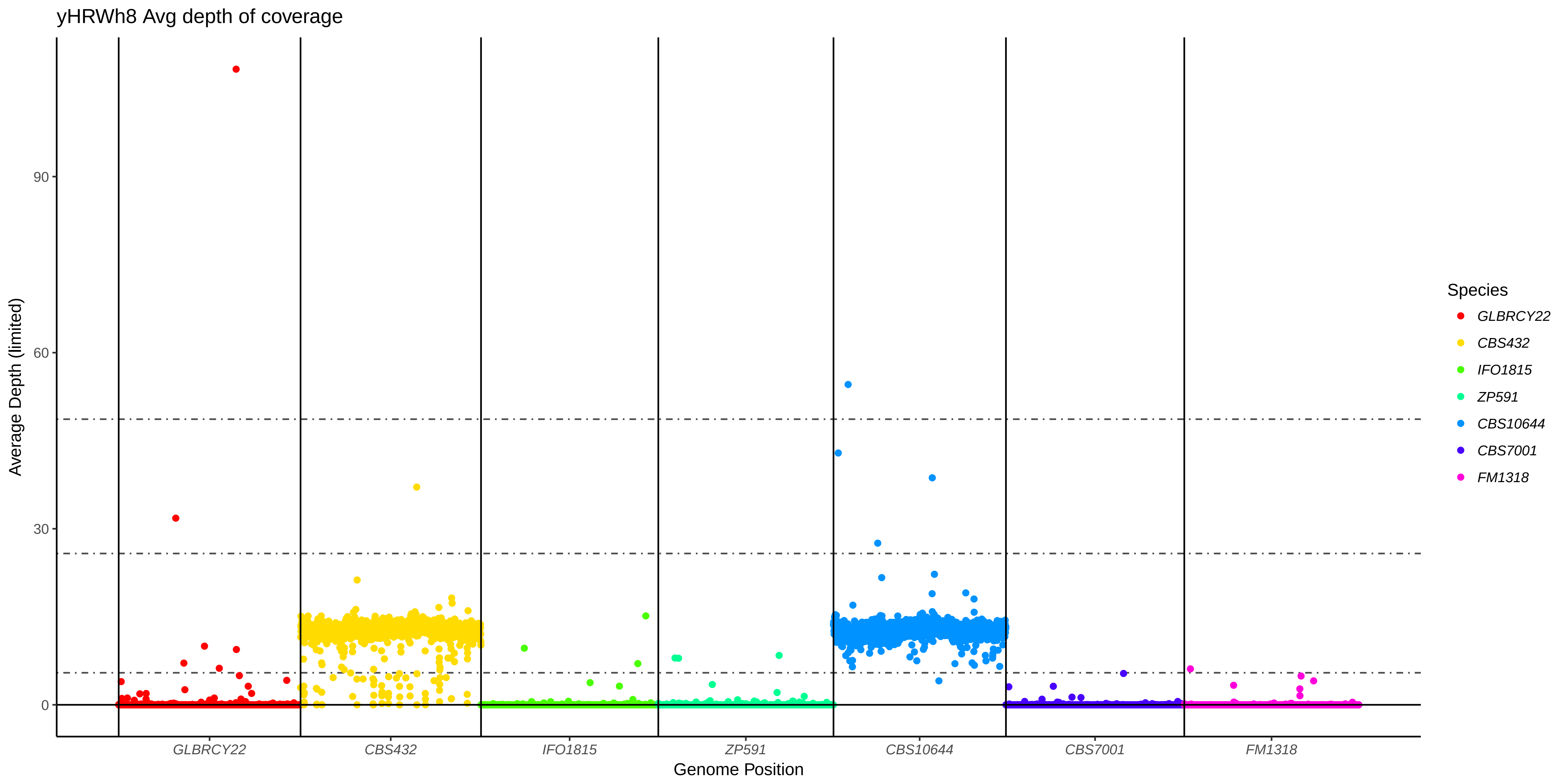

yHRWh8 Avg depth of coverage
Average Depth (limited)
0
30
60
90
GLBRCY22
CBS432
IFO1815
ZP591
CBS10644
CBS7001
FM1318
Genome Position
Species
GLBRCY22
CBS432
IFO1815
ZP591
CBS10644
CBS7001
FM1318

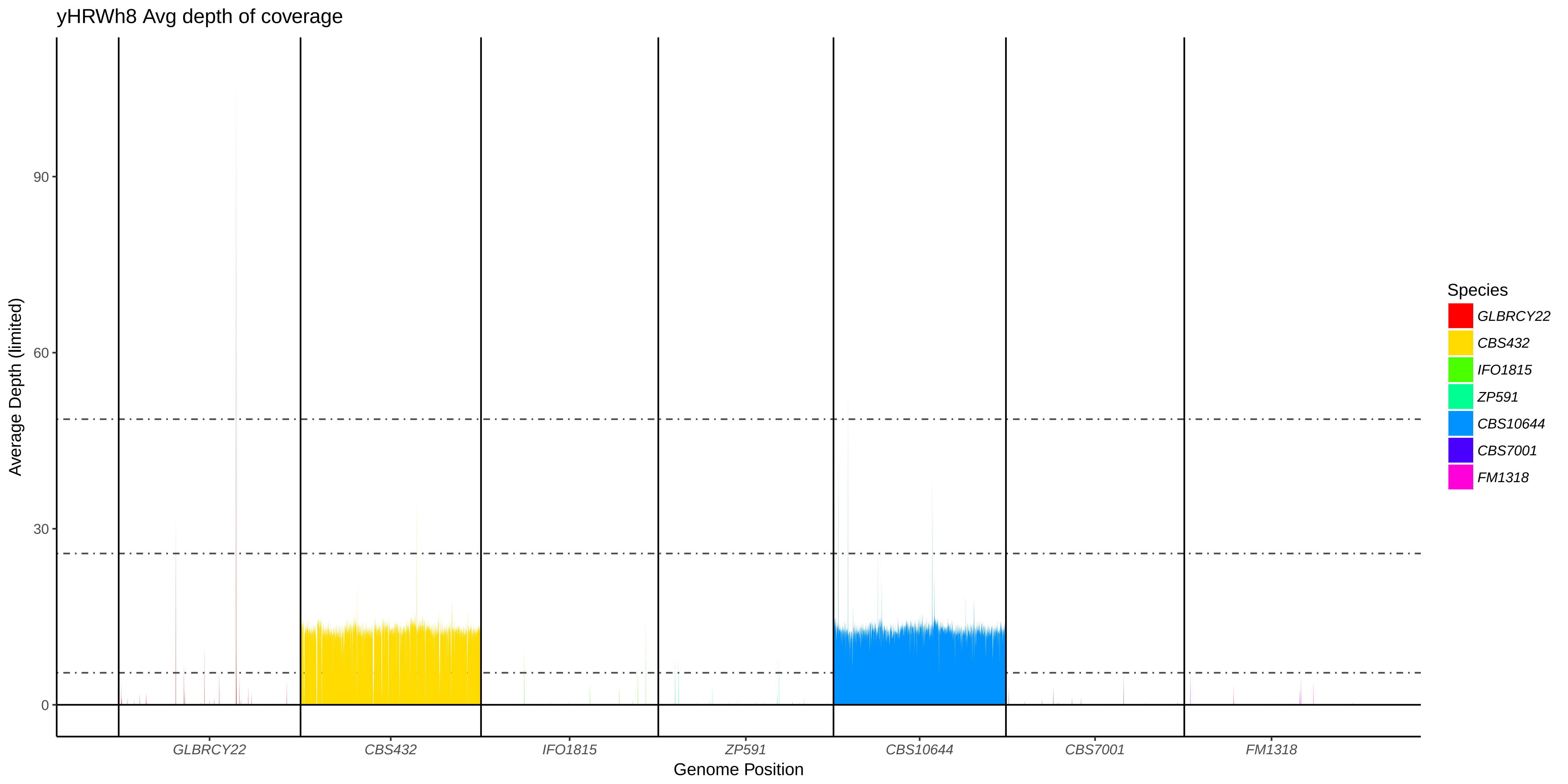

yHRWh8 Avg depth of coverage
Average Depth (limited)
90
60
30
0
GLBRCY22
CBS432
IFO1815
ZP591
CBS10644
CBS7001
FM1318
Genome Position
Species
GLBRCY22
CBS432
IFO1815
ZP591
CBS10644
CBS7001
FM1318

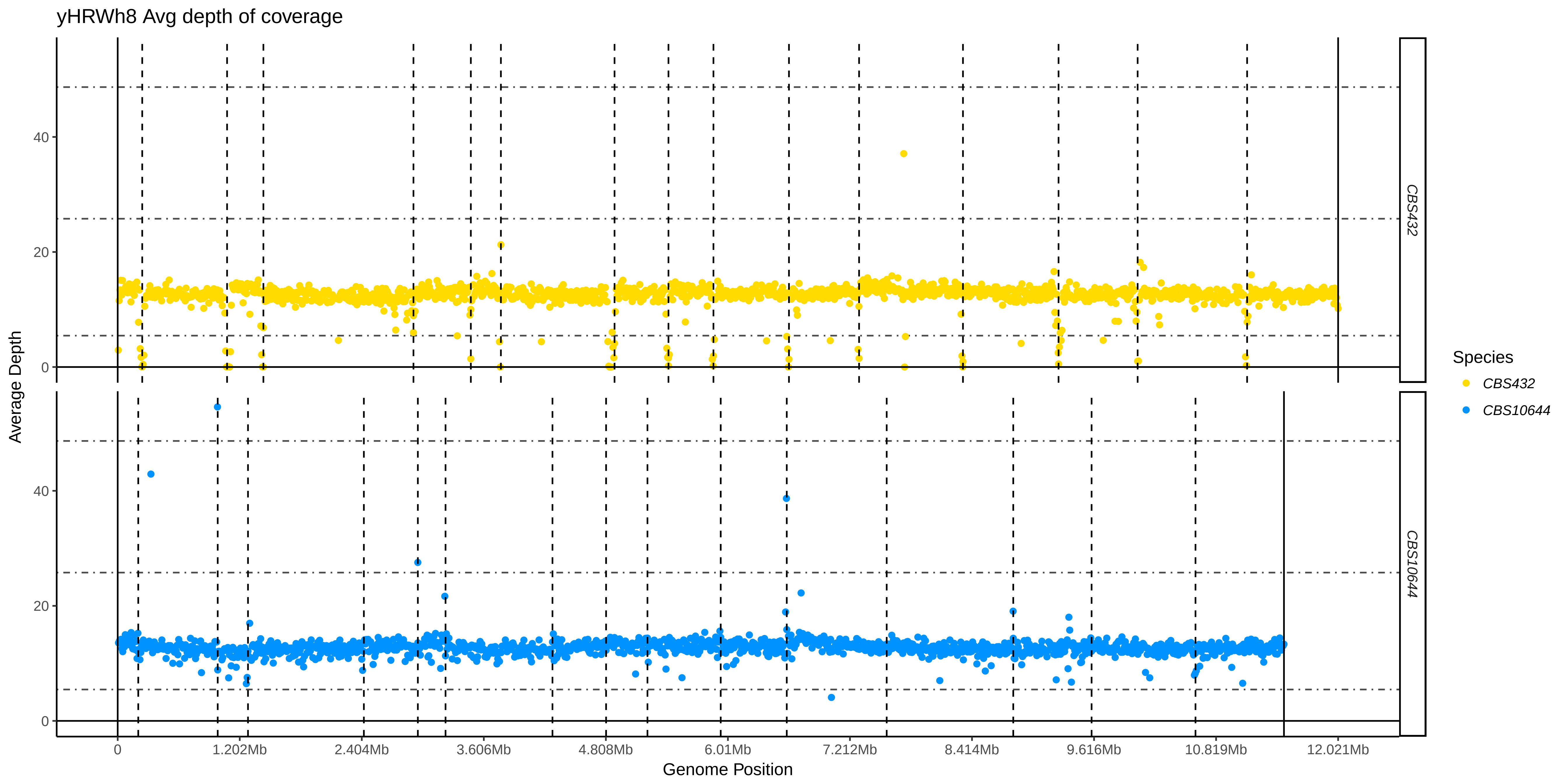

yHRWh8 Avg depth of coverage
Average Depth
0
20
40
CBS432
CBS10644
Genome Position
1.202Mb
2.404Mb
3.606Mb
4.808Mb
6.01Mb
7.212Mb
8.414Mb
9.616Mb
10.819Mb
12.021Mb
Species
CBS432
CBS10644

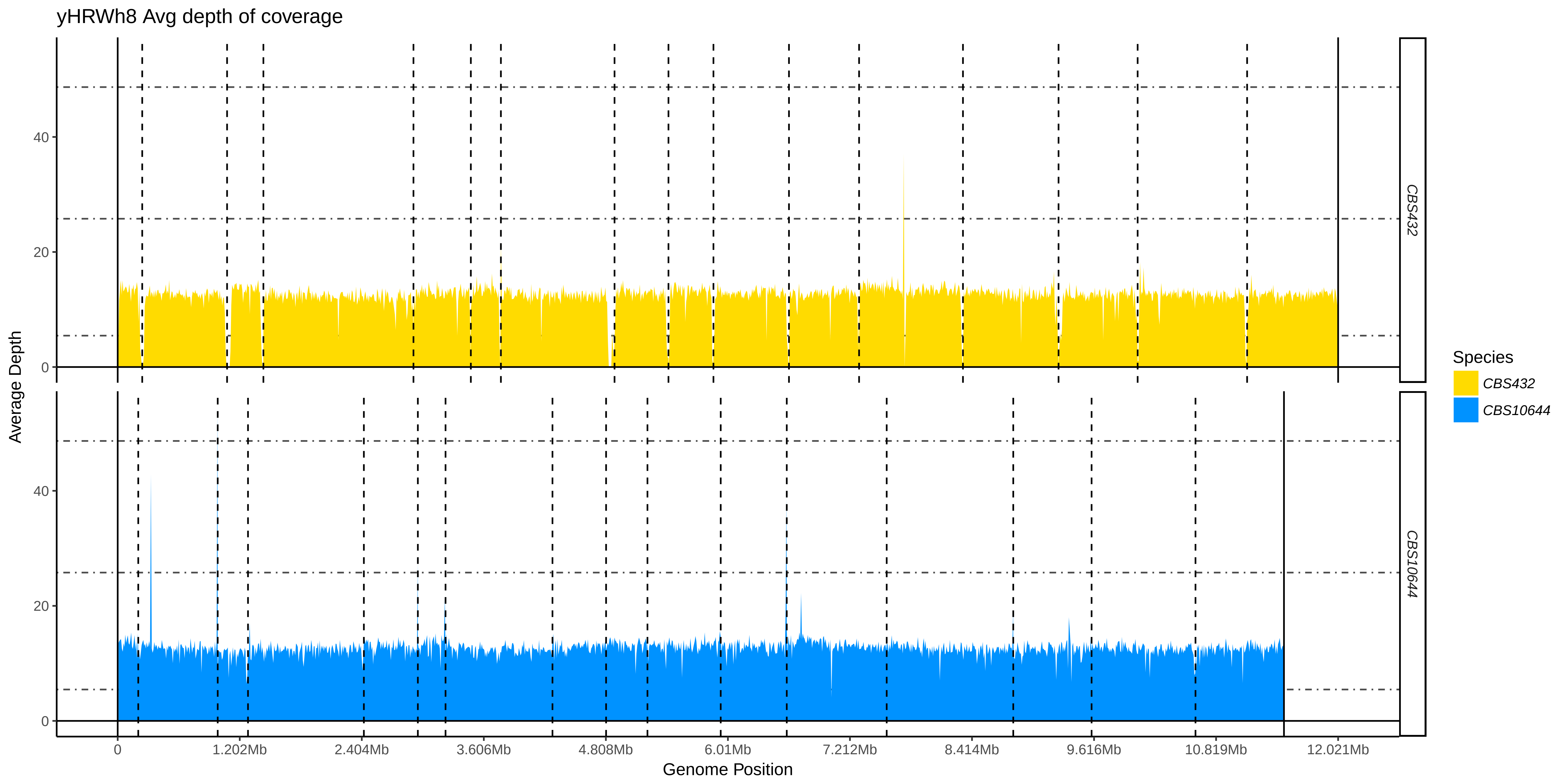

yHRWh8 Avg depth of coverage
Average Depth
40
20
0
CBS432
CBS10644
0
1.202Mb
2.404Mb
3.606Mb
4.808Mb
6.01Mb
7.212Mb
8.414Mb
9.616Mb
10.819Mb
12.021Mb
Genome Position
Species
CBS432
CBS10644

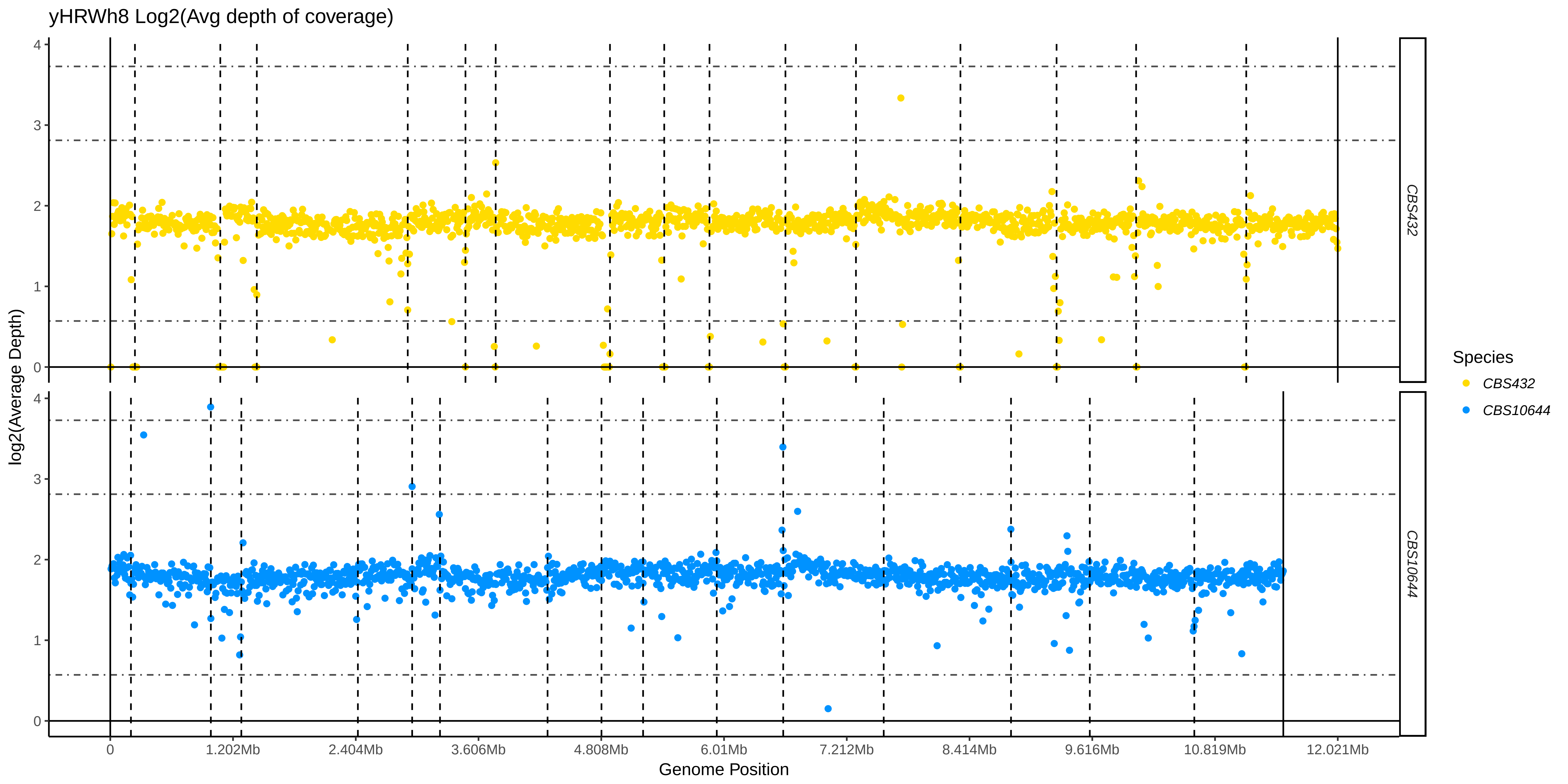
yHRWh8 Log2(Avg depth of coverage)
log2(Average Depth)
CBS432
CBS10644
0
1
2
3
4
0
1.202Mb
2.404Mb
3.606Mb
4.808Mb
6.01Mb
7.212Mb
8.414Mb
9.616Mb
10.819Mb
12.021Mb
Genome Position
Species
CBS432
CBS10644

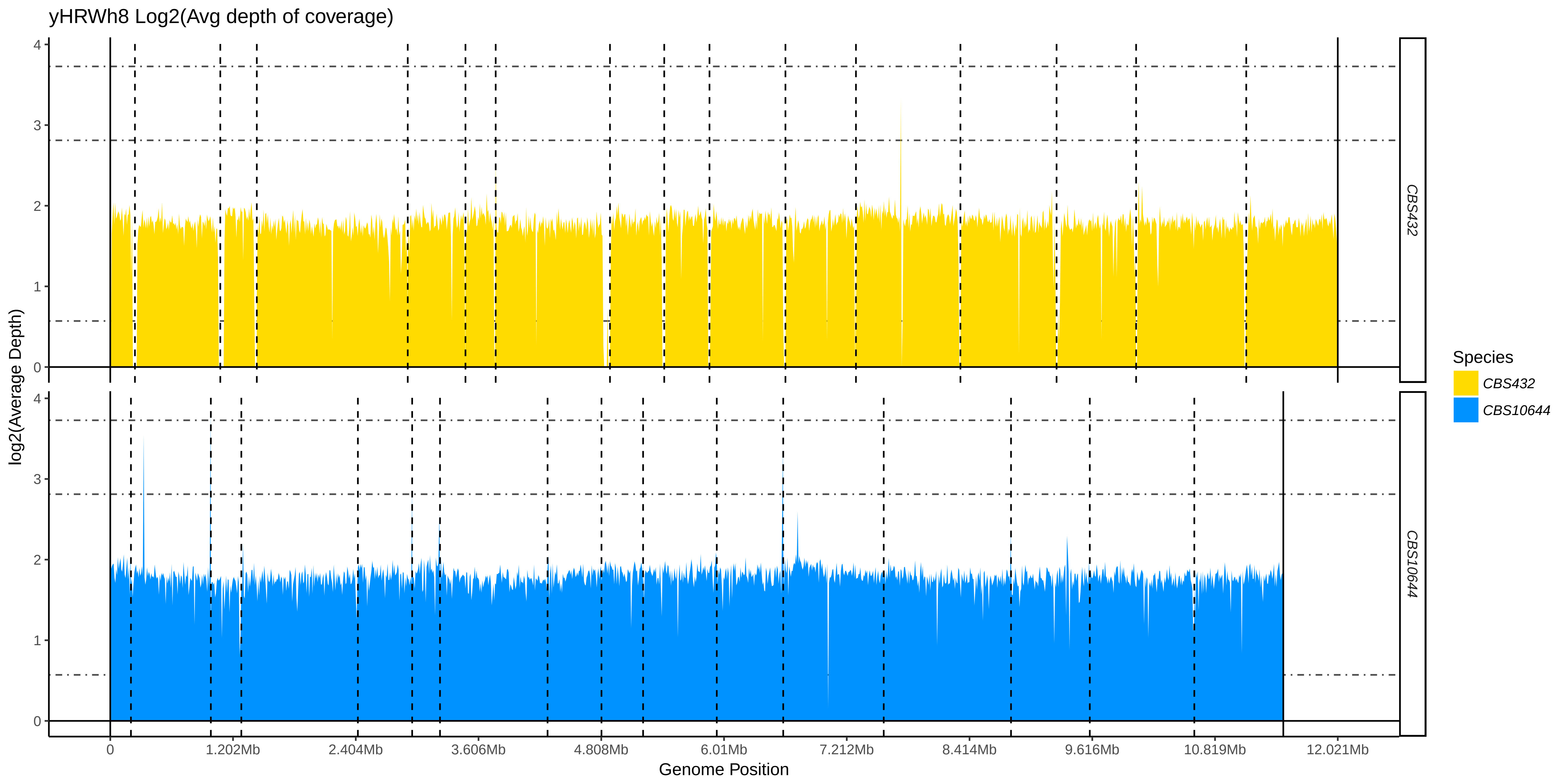
yHRWh8 Log2(Avg depth of coverage)
log2(Average Depth)
0
1
2
3
4
CBS432
CBS10644
Genome Position
0
1.202Mb
2.404Mb
3.606Mb
4.808Mb
6.01Mb
7.212Mb
8.414Mb
9.616Mb
10.819Mb
12.021Mb
Species
CBS432
CBS10644

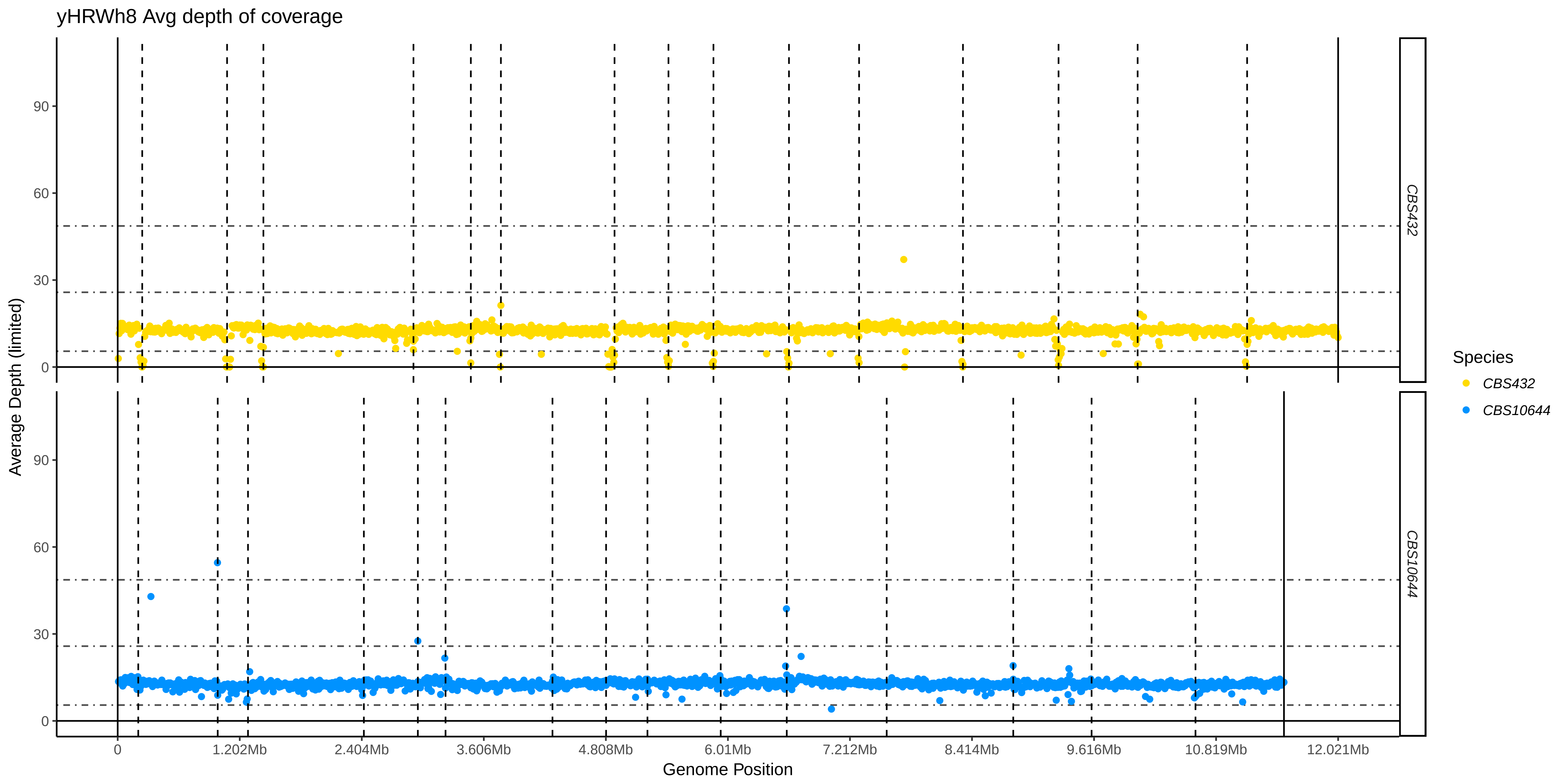

yHRWh8 Avg depth of coverage
Average Depth (limited)
Genome Position
CBS432
CBS10644
Species
CBS432
CBS10644
0
30
60
90
0
1.202Mb
2.404Mb
3.606Mb
4.808Mb
6.01Mb
7.212Mb
8.414Mb
9.616Mb
10.819Mb
12.021Mb

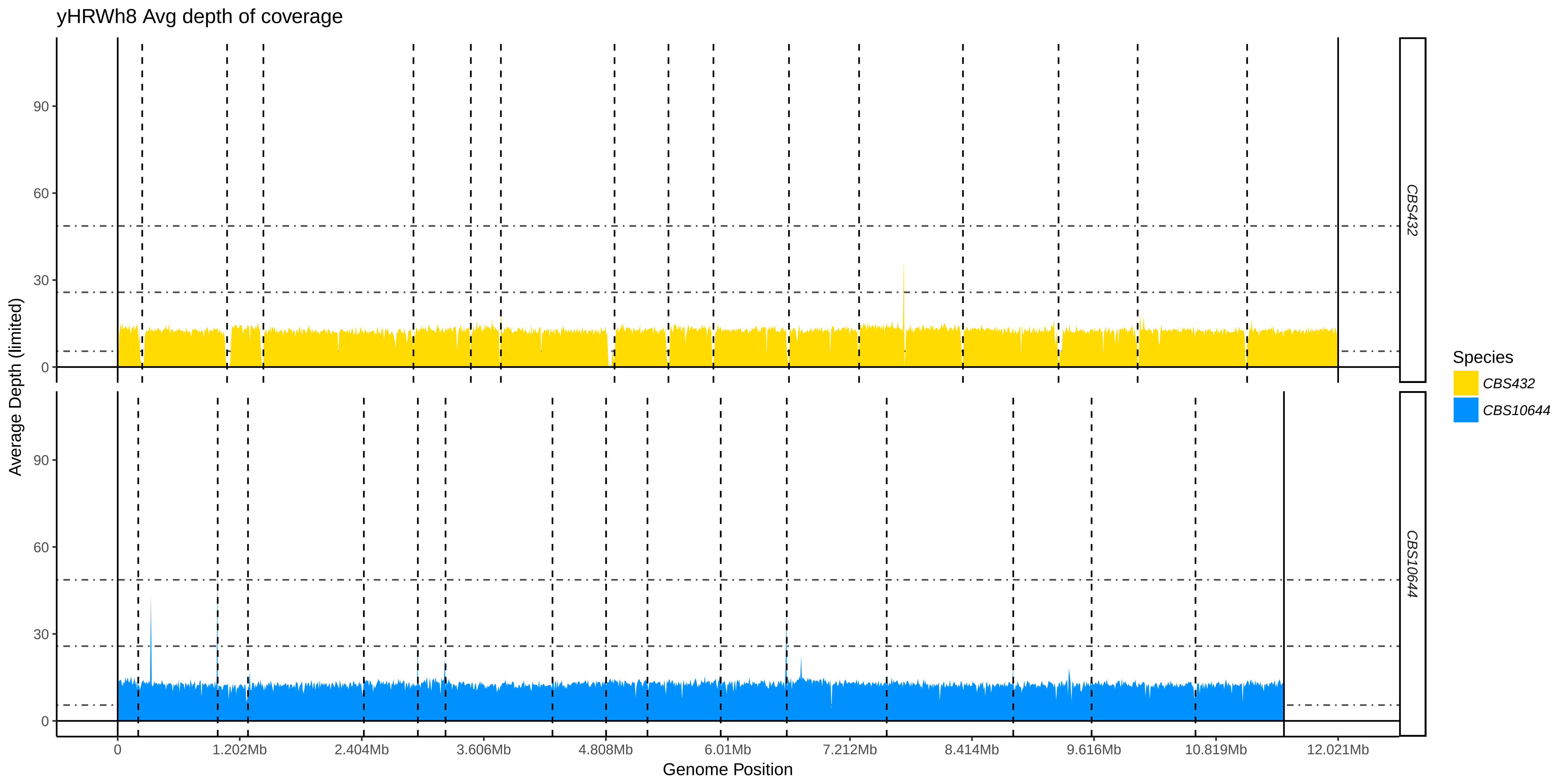

yHRWh8 Avg depth of coverage
Average Depth (limited)
0
30
60
90
0
1.202Mb
2.404Mb
3.606Mb
4.808Mb
6.01Mb
7.212Mb
8.414Mb
9.616Mb
10.819Mb
12.021Mb
Genome Position
CBS432
CBS10644
Species
CBS432
CBS10644
