# Supplementary material for: Synthetic hybrids of six yeast species
Source: Nat Commun. 2020 Apr 29;11:2085. doi: 10.1038/s41467-020-15559-4 (PMC7190663; doi:10.1038/s41467-020-15559-4)
Supplement: Supplementary file 15 — Source Data [file 41467_2020_15559_MOESM15_ESM.zip › Source Data 2.zip!/yHRWh13_sppIDerDepthPlot-covBins.pdf]

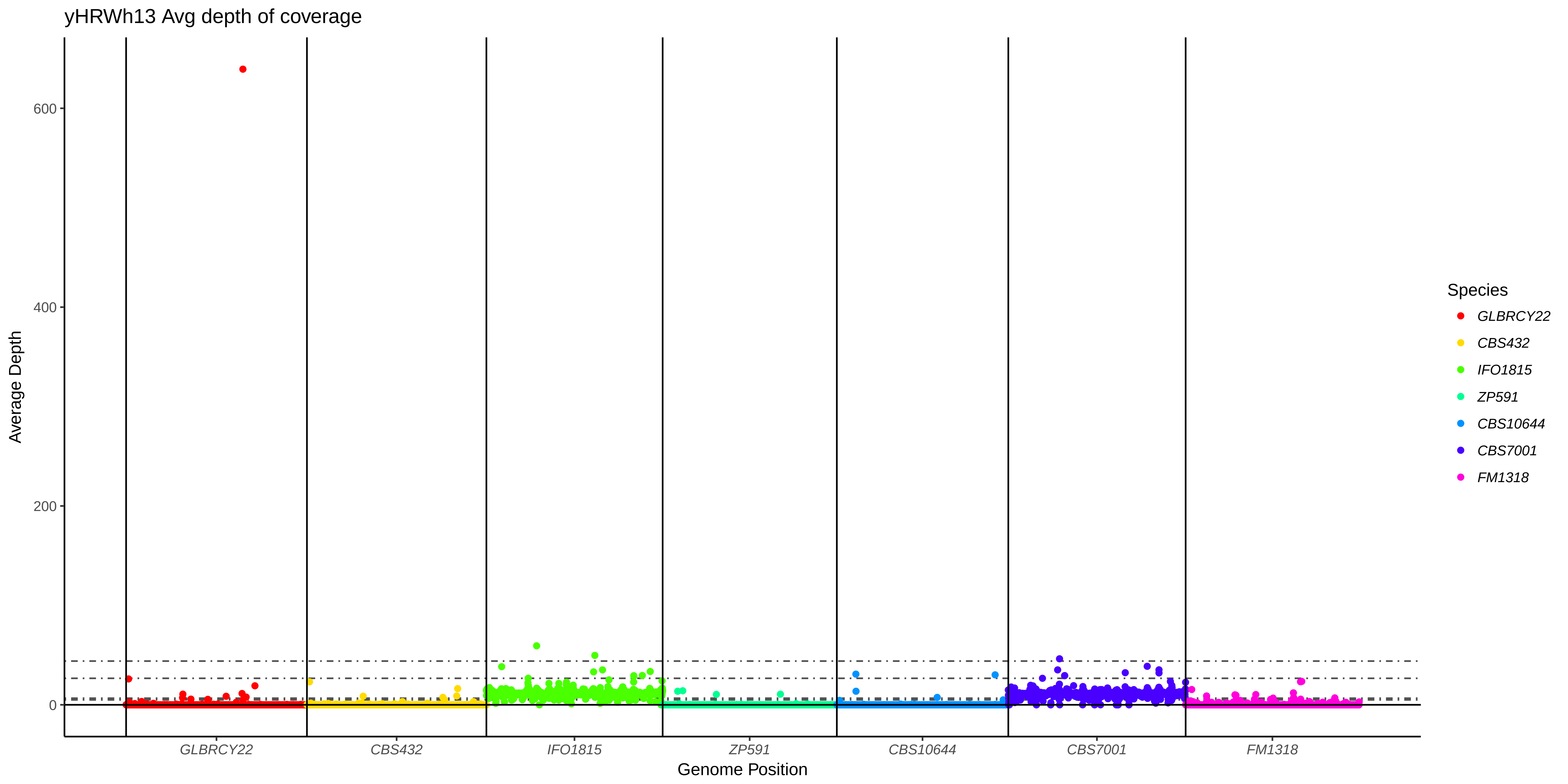
yHRWh13 Avg depth of coverage
Average Depth
600
400
200
0
GLBRCY22
CBS432
IFO1815
ZP591
CBS10644
CBS7001
FM1318
Genome Position
Species
GLBRCY22
CBS432
IFO1815
ZP591
CBS10644
CBS7001
FM1318

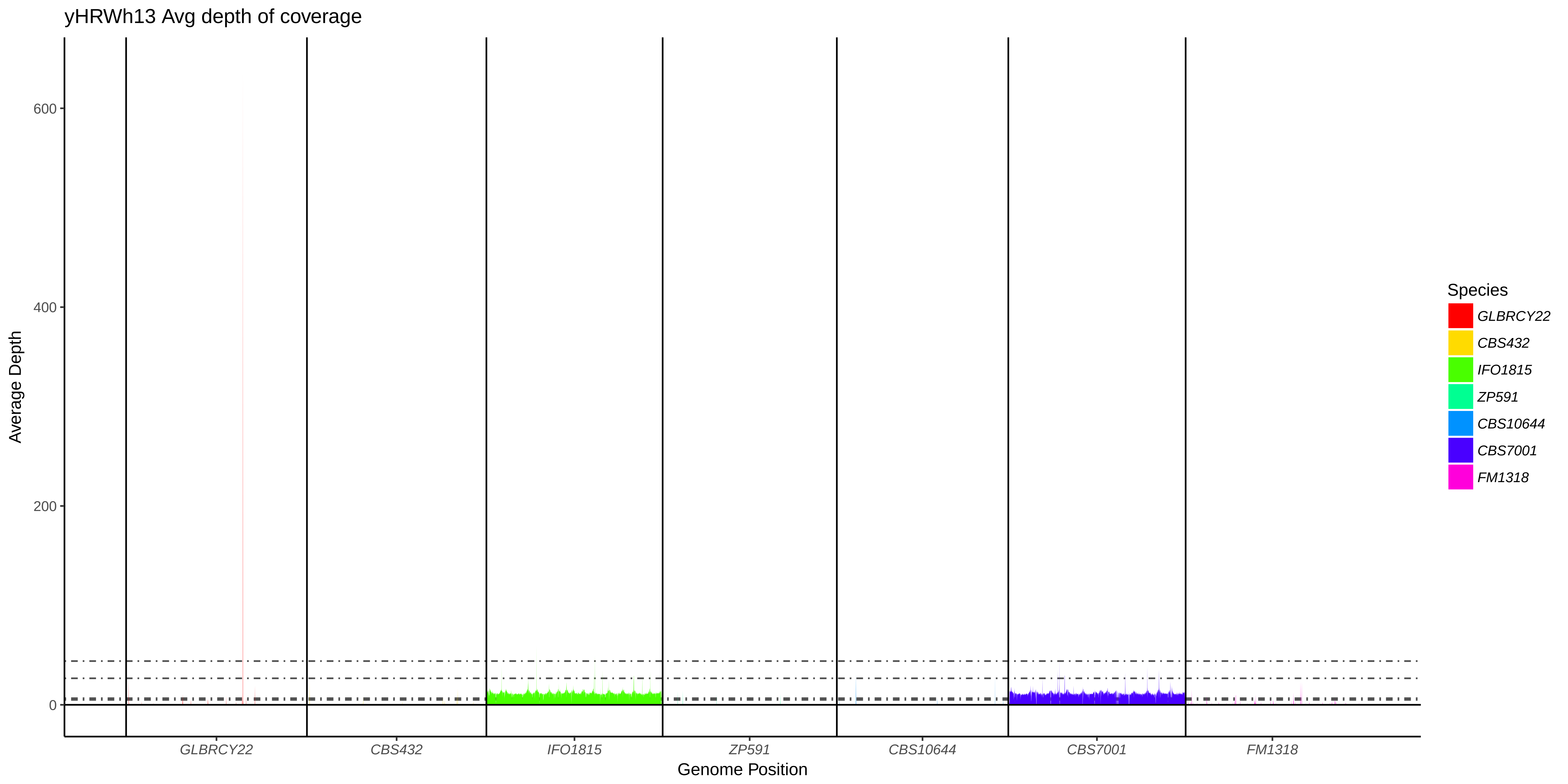
yHRWh13 Avg depth of coverage
600
400
200
0
Average Depth
GLBRCY22
CBS432
IFO1815
ZP591
CBS10644
CBS7001
FM1318
Genome Position
Species
GLBRCY22
CBS432
IFO1815
ZP591
CBS10644
CBS7001
FM1318

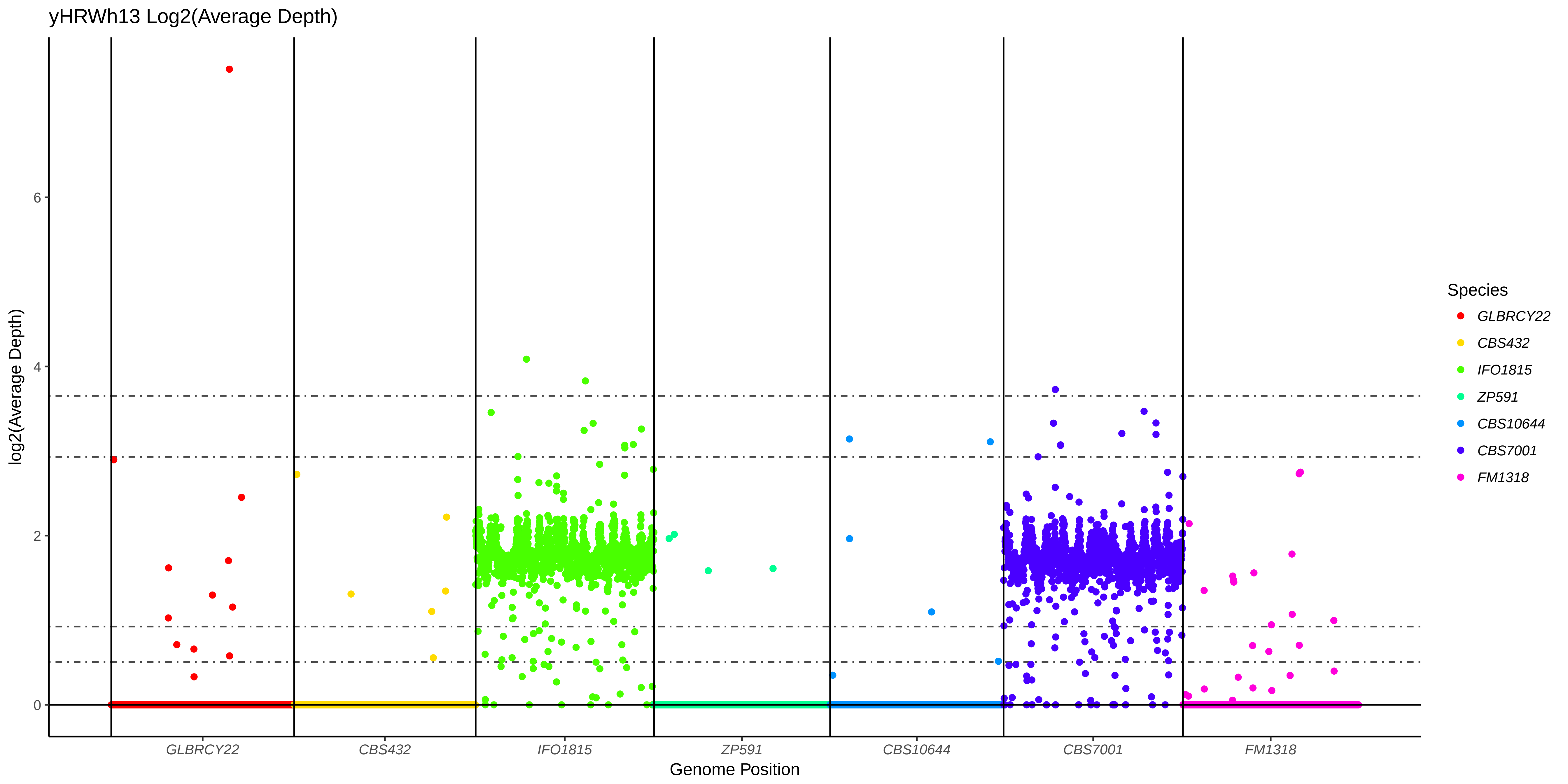
yHRWh13 Log2(Average Depth)
log2(Average Depth)
0
2
4
6
GLBRCY22
CBS432
IFO1815
ZP591
CBS10644
CBS7001
FM1318
Genome Position
Species
GLBRCY22
CBS432
IFO1815
ZP591
CBS10644
CBS7001
FM1318

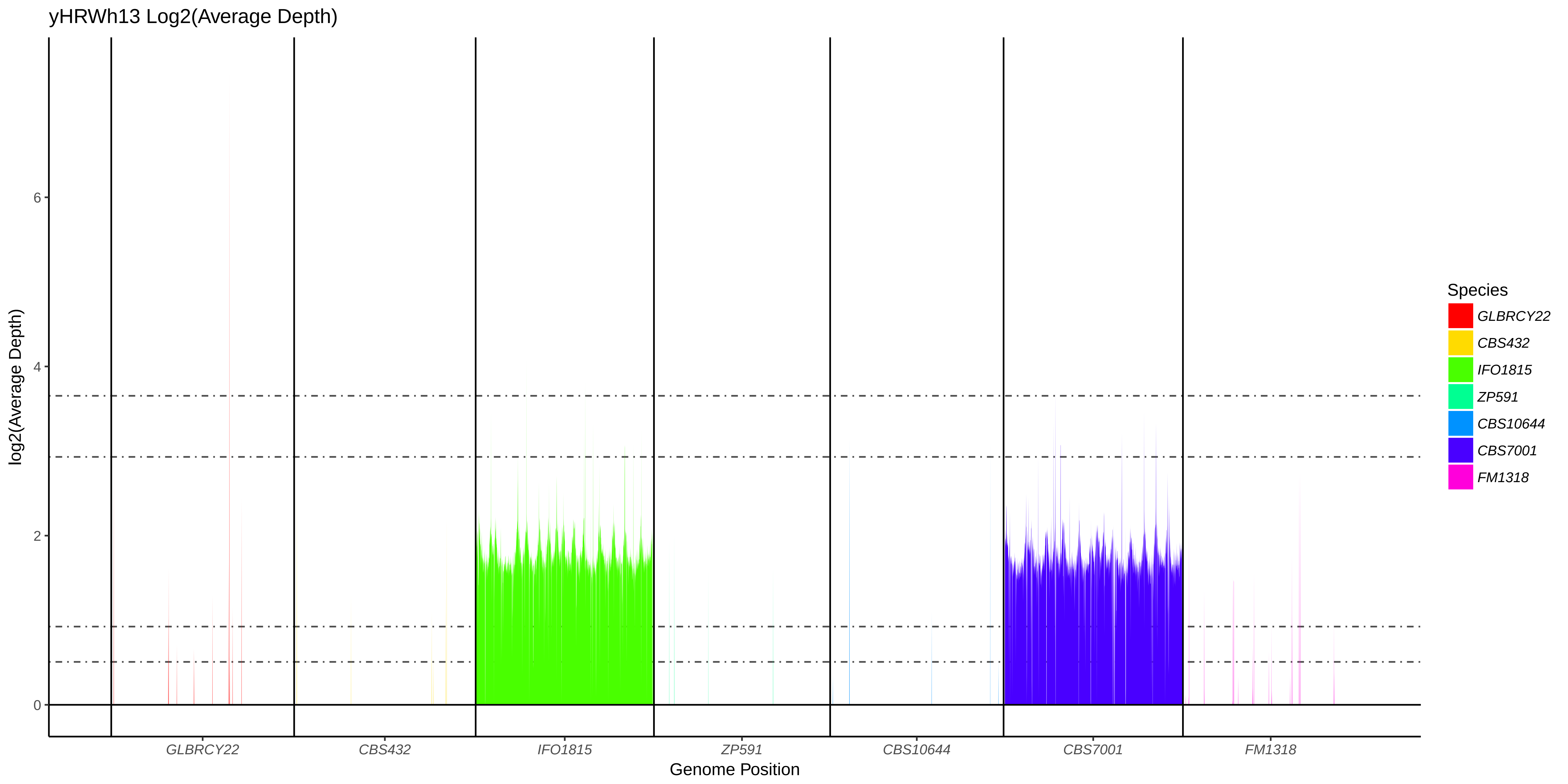

yHRWh13 Log2(Average Depth)
log2(Average Depth)
0
2
4
6
GLBRCY22
CBS432
IFO1815
ZP591
CBS10644
CBS7001
FM1318
Genome Position
Species
GLBRCY22
CBS432
IFO1815
ZP591
CBS10644
CBS7001
FM1318

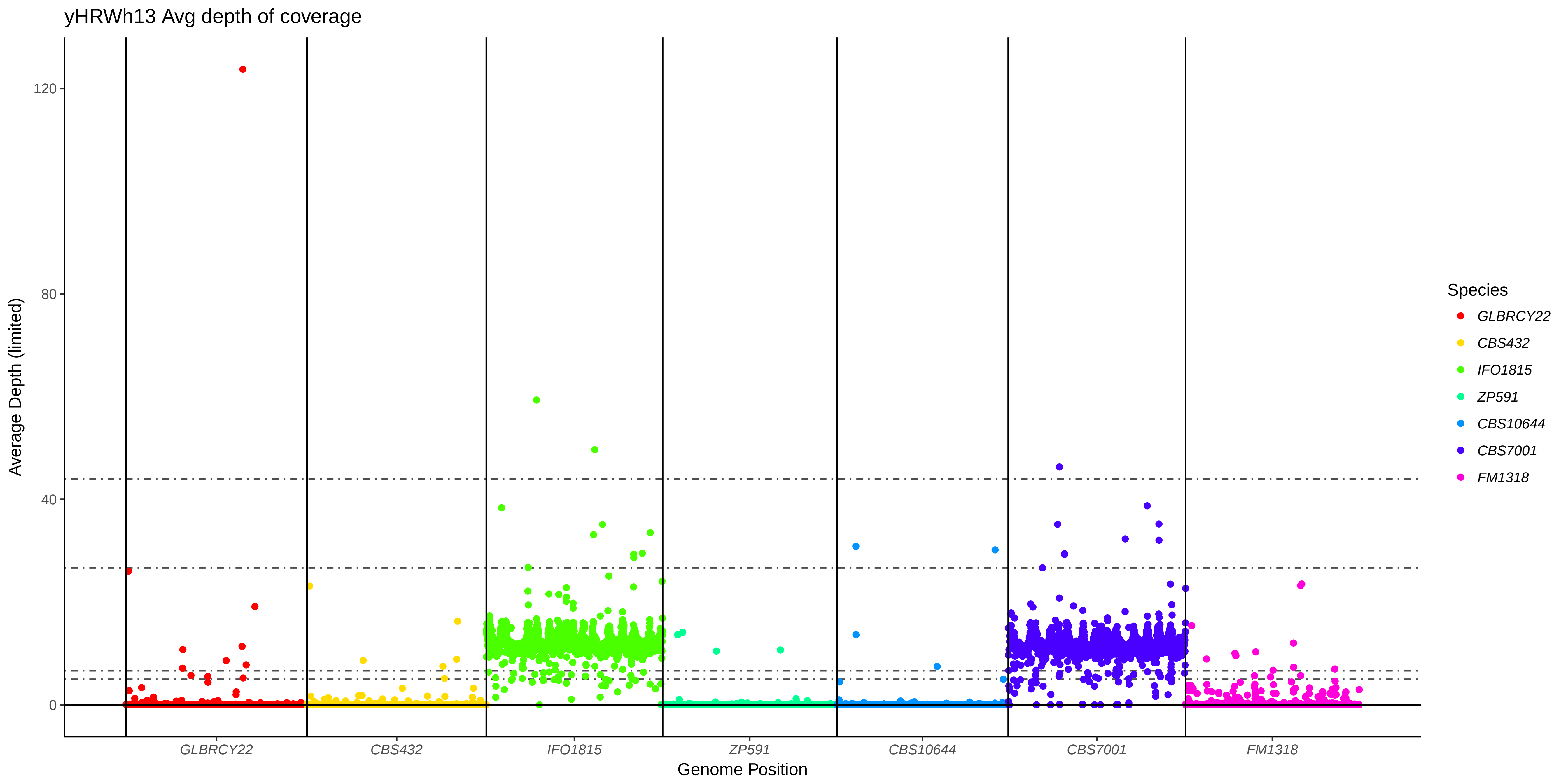

yHRWh13 Avg depth of coverage
Average Depth (limited)
120
80
40
0
GLBRCY22
CBS432
IFO1815
ZP591
CBS10644
CBS7001
FM1318
Genome Position
Species
GLBRCY22
CBS432
IFO1815
ZP591
CBS10644
CBS7001
FM1318

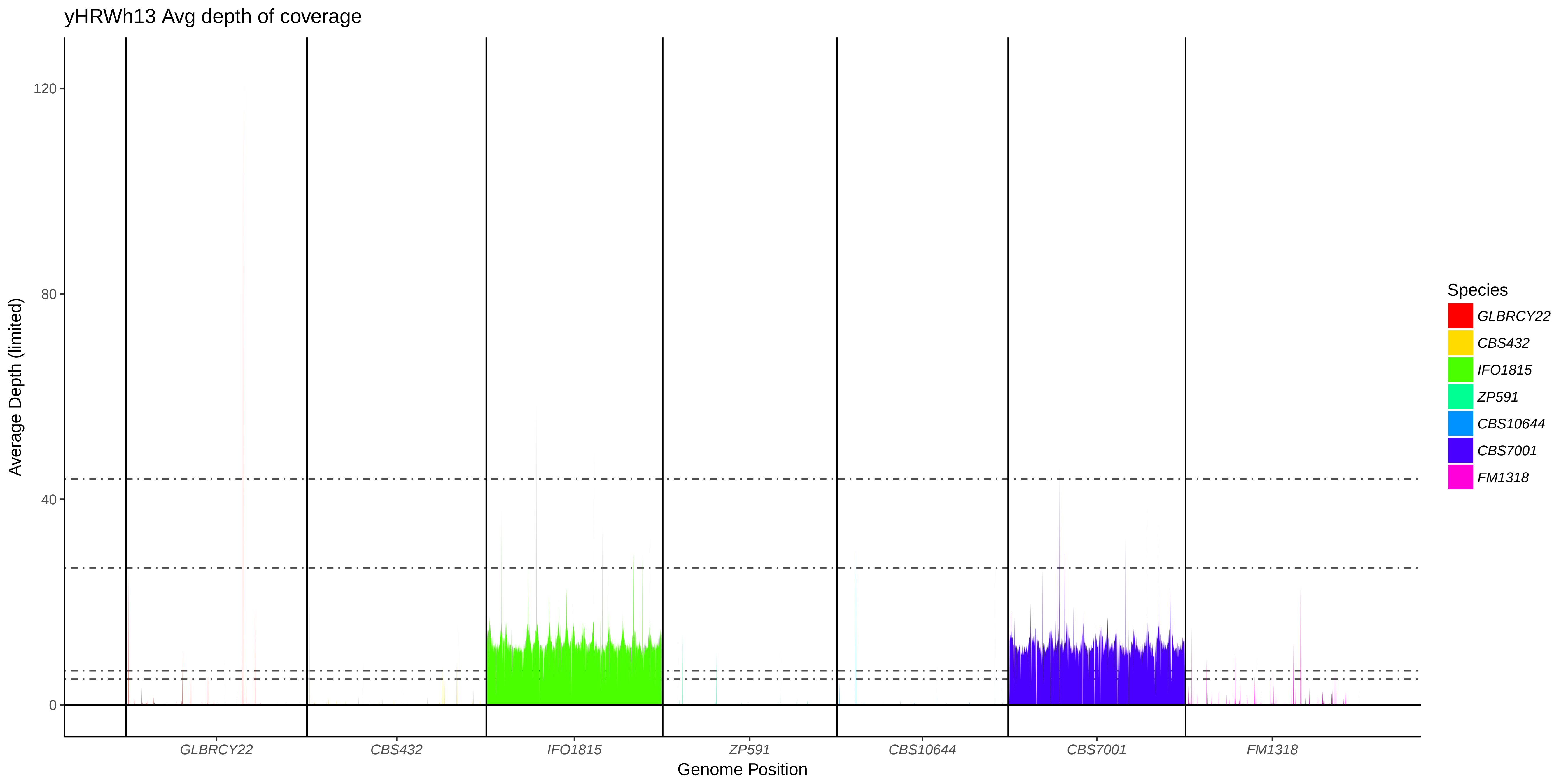

yHRWh13 Avg depth of coverage
Average Depth (limited)
120
80
40
0
GLBRCY22
CBS432
IFO1815
ZP591
CBS10644
CBS7001
FM1318
Genome Position
Species
GLBRCY22
CBS432
IFO1815
ZP591
CBS10644
CBS7001
FM1318

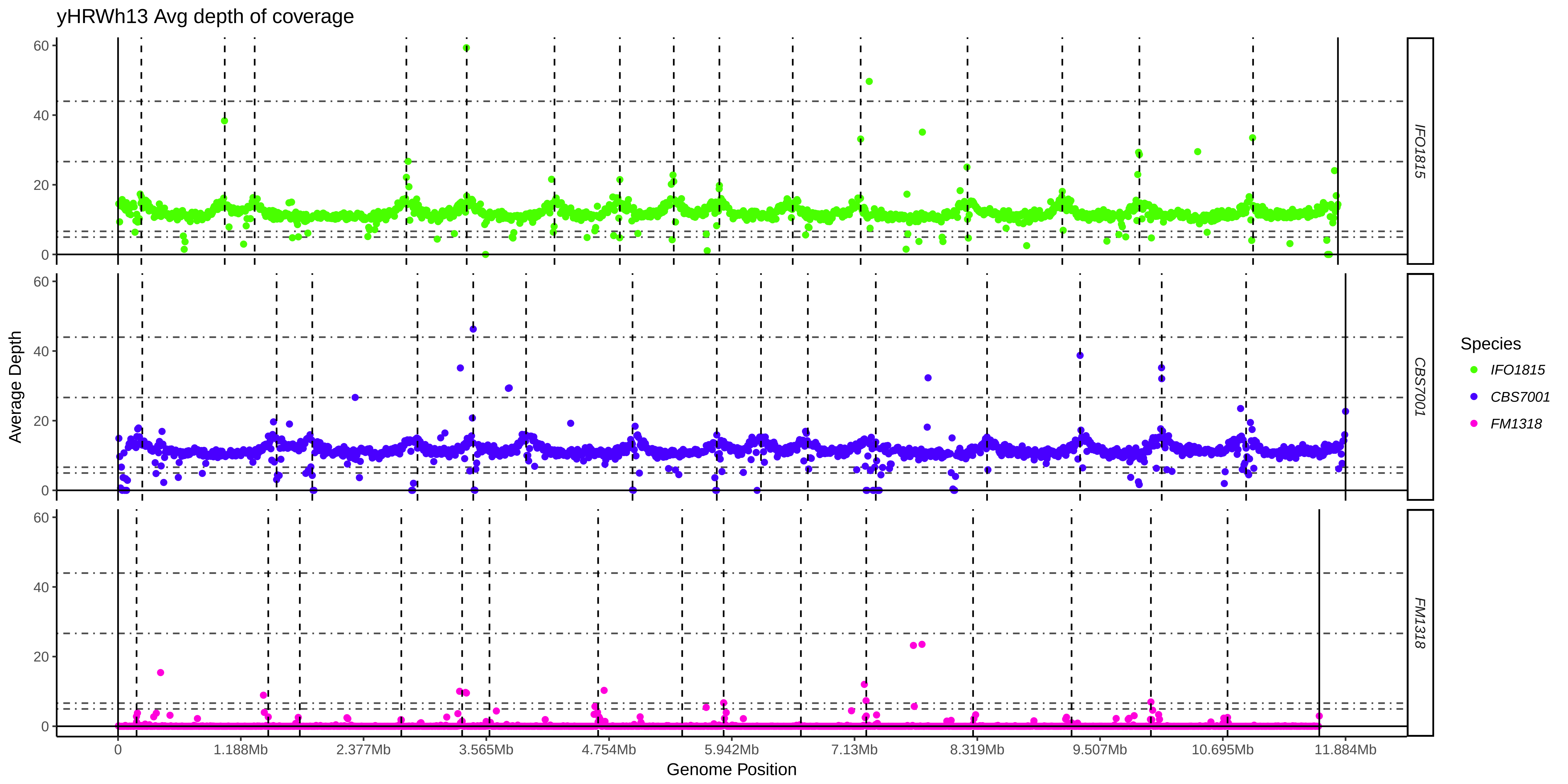

yHRWh13 Avg depth of coverage
Average Depth
Genome Position
0
1.188Mb
2.377Mb
3.565Mb
4.754Mb
5.942Mb
7.13Mb
8.319Mb
9.507Mb
10.695Mb
11.884Mb
IFO1815
CBS7001
FM1318
Species
IFO1815
CBS7001
FM1318

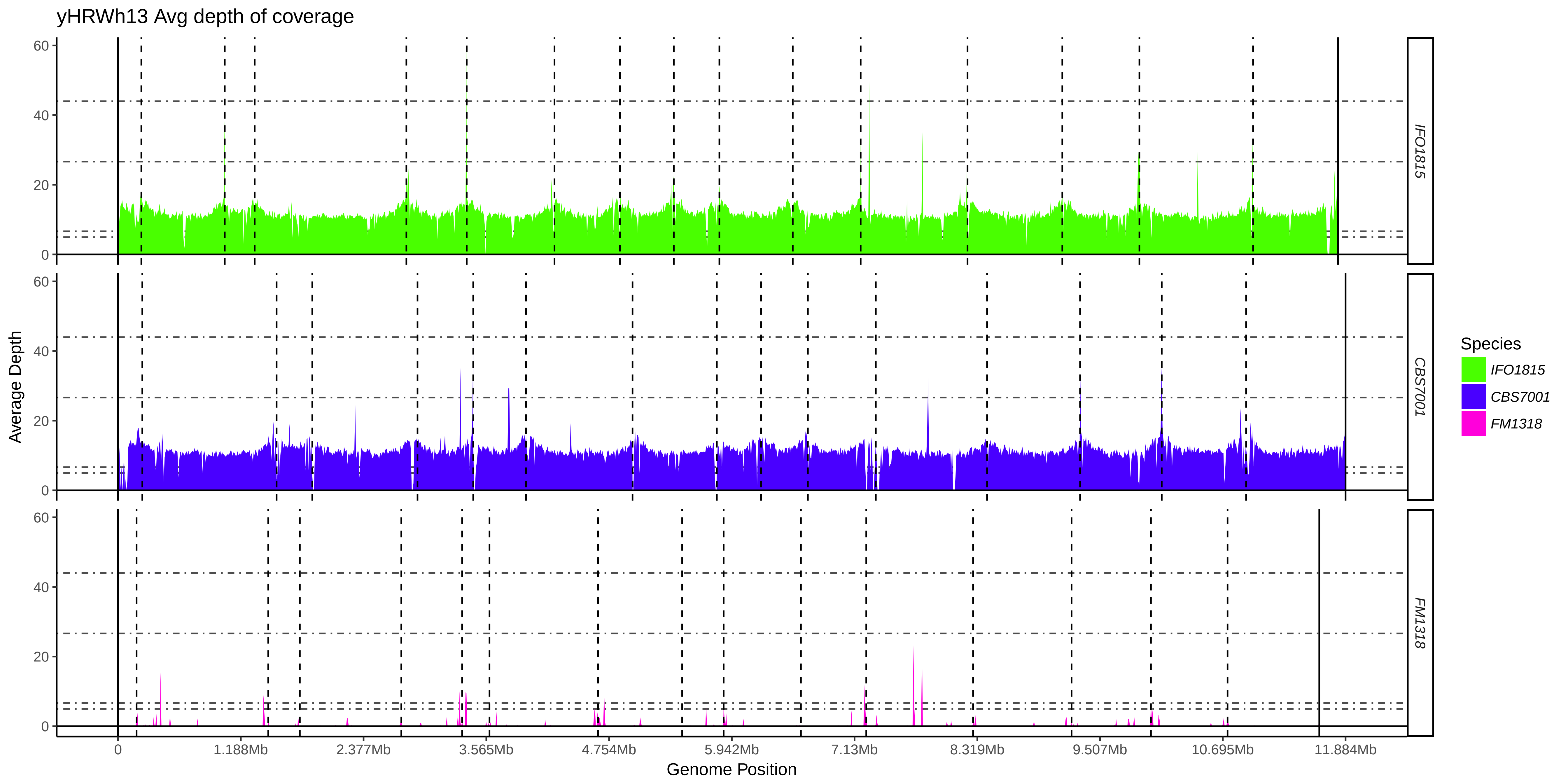

yHRWh13 Avg depth of coverage
Average Depth
60
40
20
0
IFO1815
CBS7001
FM1318
0
1.188Mb
2.377Mb
3.565Mb
4.754Mb
5.942Mb
7.13Mb
8.319Mb
9.507Mb
10.695Mb
11.884Mb
Genome Position
Species
IFO1815
CBS7001
FM1318

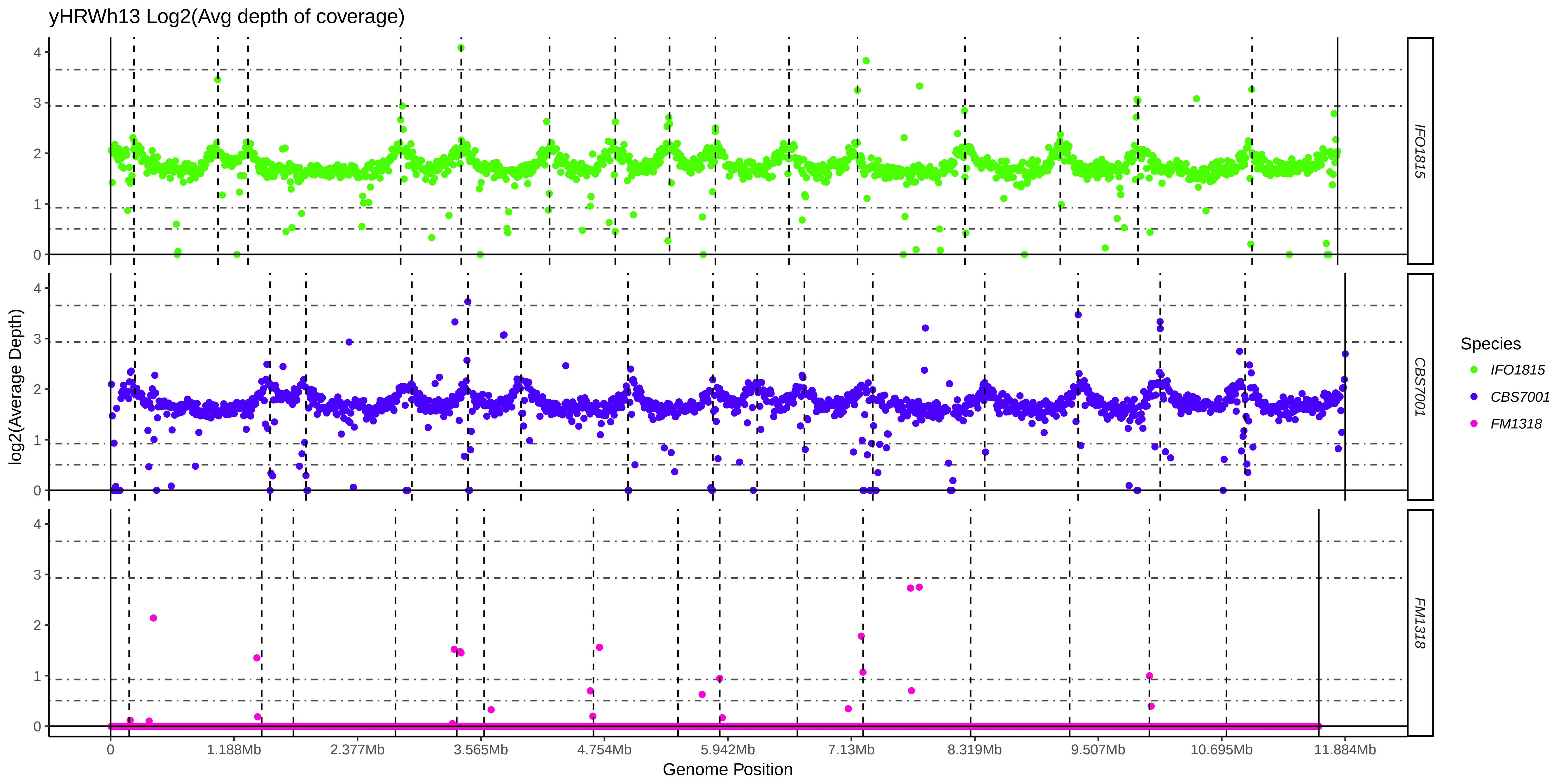
yHRWh13 Log2(Avg depth of coverage)
log2(Average Depth)
Genome Position
0
1.188Mb
2.377Mb
3.565Mb
4.754Mb
5.942Mb
7.13Mb
8.319Mb
9.507Mb
10.695Mb
11.884Mb
IFO1815
CBS7001
FM1318
Species
IFO1815
CBS7001
FM1318

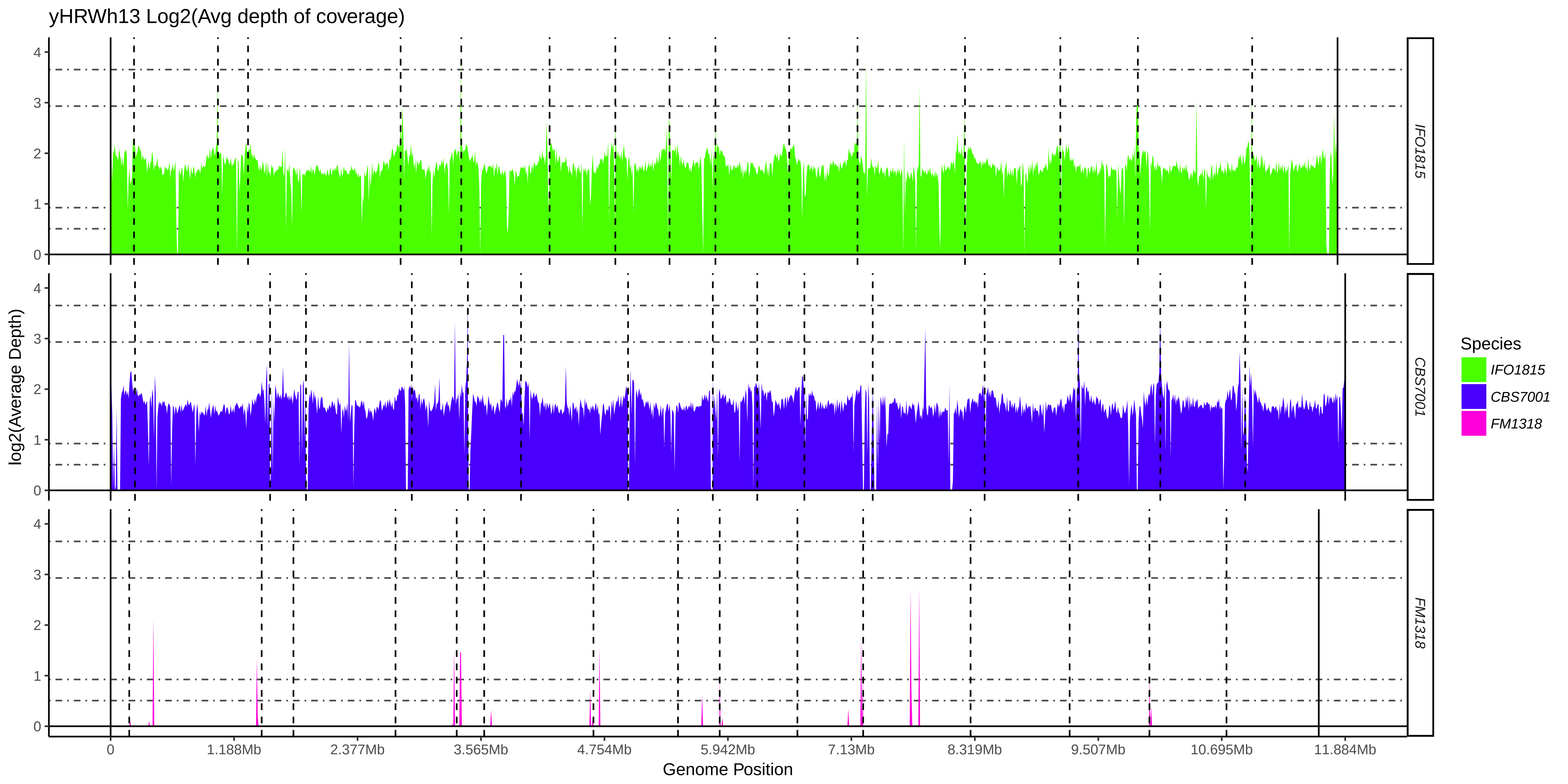

yHRWh13 Log2(Avg depth of coverage)
log2(Average Depth)
Genome Position
0
1.188Mb
2.377Mb
3.565Mb
4.754Mb
5.942Mb
7.13Mb
8.319Mb
9.507Mb
10.695Mb
11.884Mb
IFO1815
CBS7001
FM1318
Species
IFO1815
CBS7001
FM1318

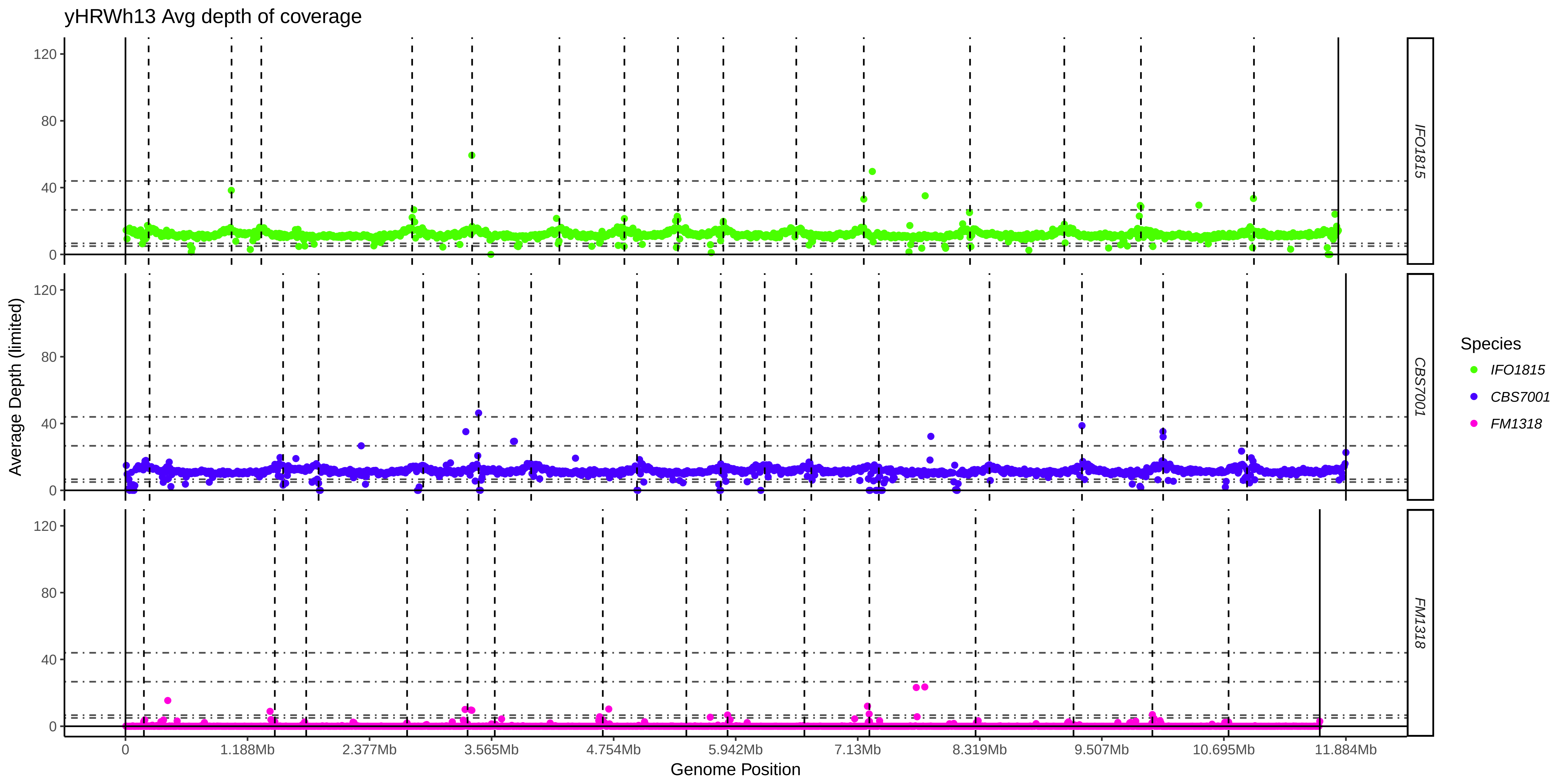
yHRWh13 Avg depth of coverage
Average Depth (limited)
Genome Position
0
40
80
120
0
1.188Mb
2.377Mb
3.565Mb
4.754Mb
5.942Mb
7.13Mb
8.319Mb
9.507Mb
10.695Mb
11.884Mb
IFO1815
CBS7001
FM1318
Species
IFO1815
CBS7001
FM1318

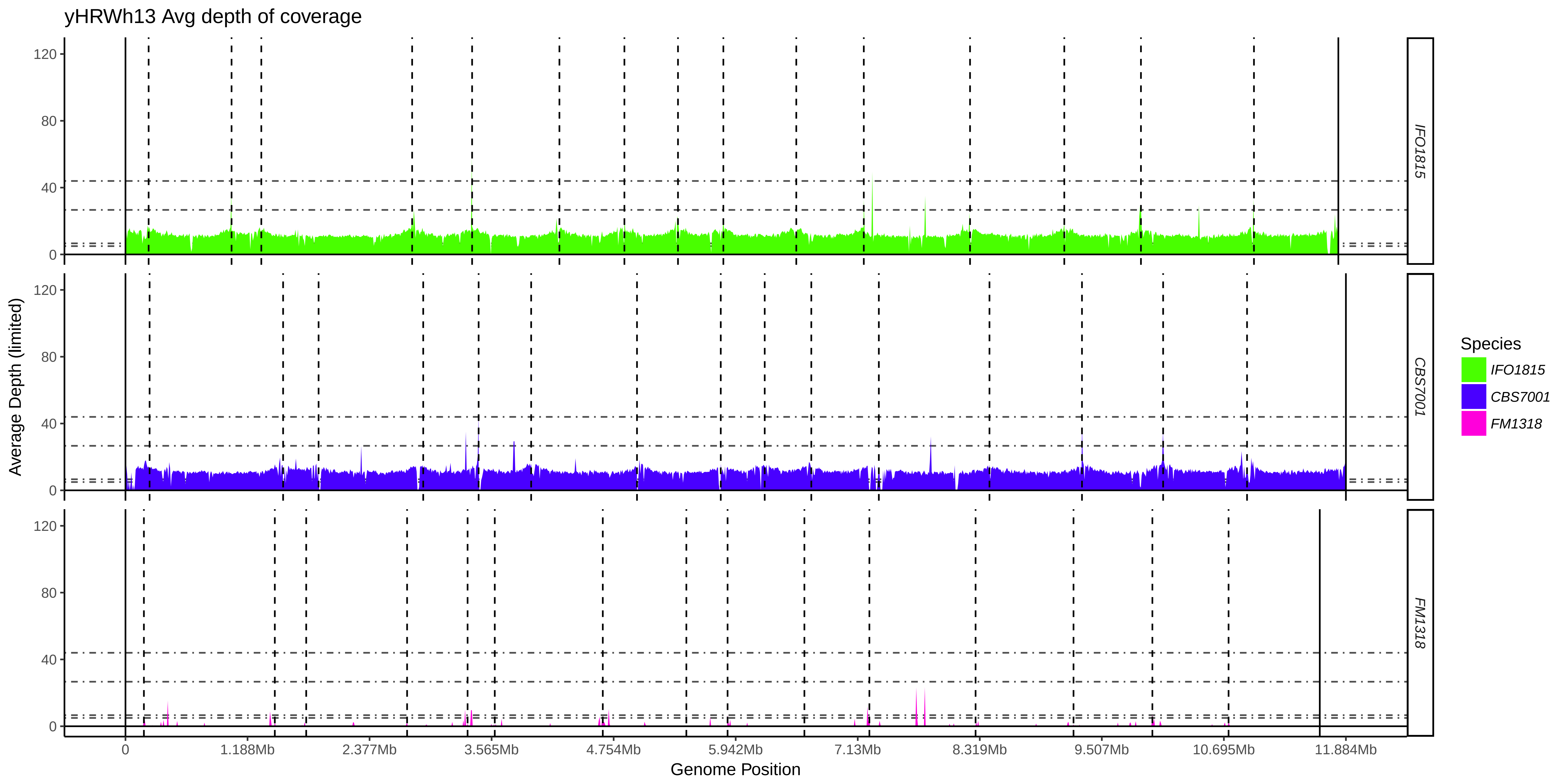

yHRWh13 Avg depth of coverage
Average Depth (limited)
0
40
80
120
IFO1815
CBS7001
FM1318
0
1.188Mb
2.377Mb
3.565Mb
4.754Mb
5.942Mb
7.13Mb
8.319Mb
9.507Mb
10.695Mb
11.884Mb
Genome Position
Species
IFO1815
CBS7001
FM1318
