# Supplementary material for: Synthetic hybrids of six yeast species
Source: Nat Commun. 2020 Apr 29;11:2085. doi: 10.1038/s41467-020-15559-4 (PMC7190663; doi:10.1038/s41467-020-15559-4)
Supplement: Supplementary file 15 — Source Data [file 41467_2020_15559_MOESM15_ESM.zip › Source Data 2.zip!/yHRWh18_sppIDerDepthPlot-covBins.pdf]

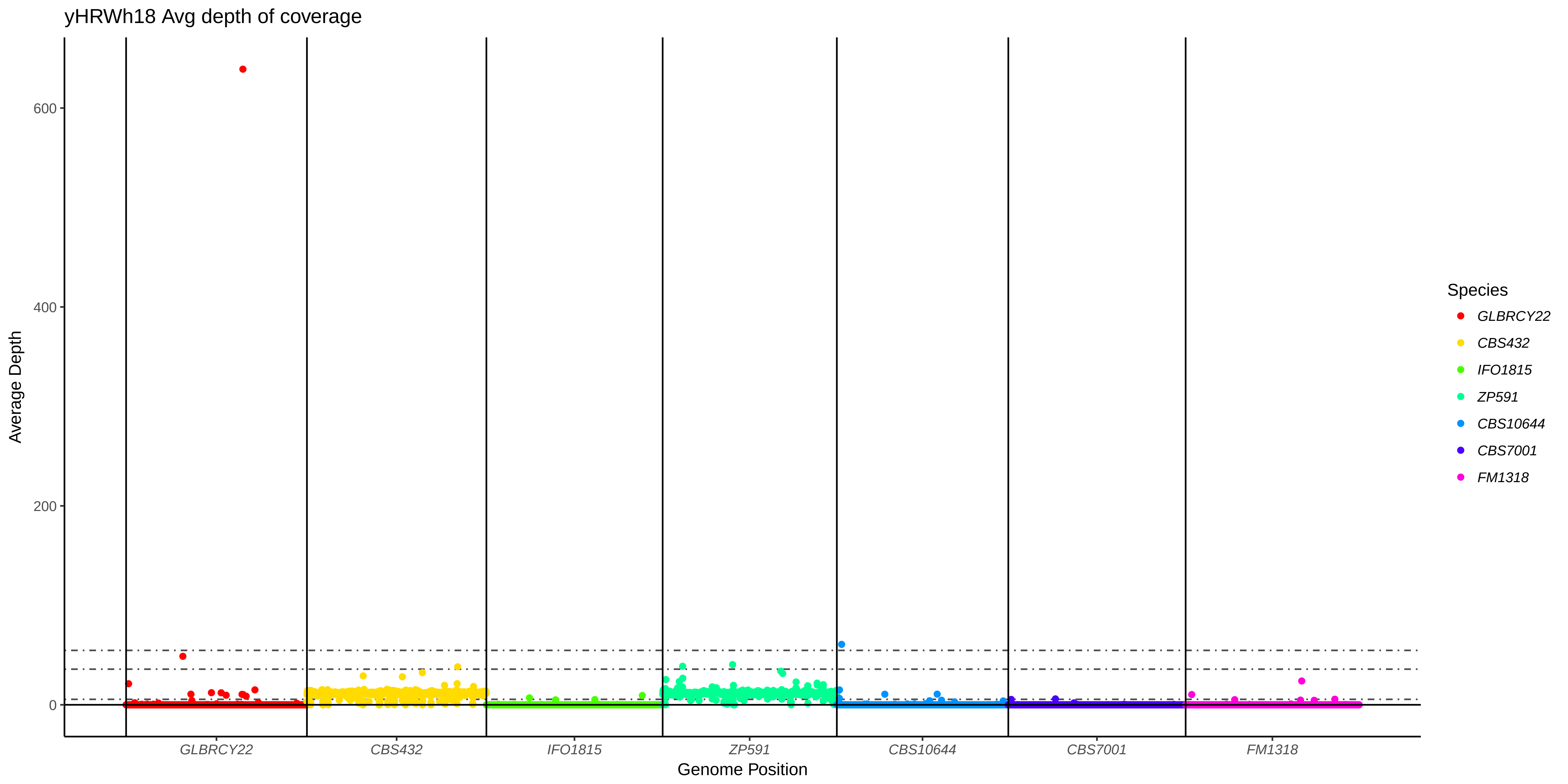
yHRWh18 Avg depth of coverage
Average Depth
600
400
200
0
GLBRCY22
CBS432
IFO1815
ZP591
CBS10644
CBS7001
FM1318
Genome Position
Species
GLBRCY22
CBS432
IFO1815
ZP591
CBS10644
CBS7001
FM1318

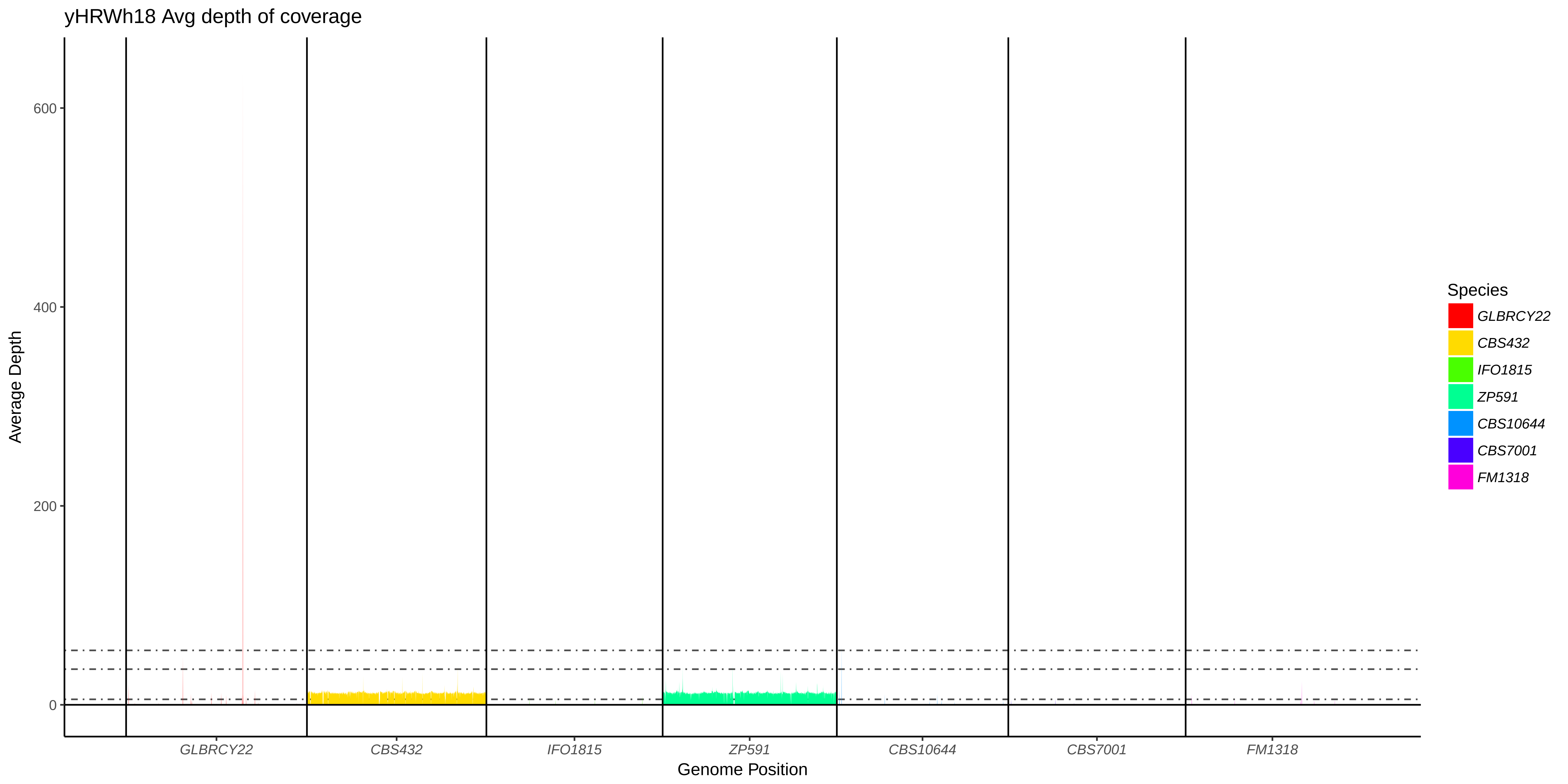
yHRWh18 Avg depth of coverage
Average Depth
600
400
200
0
GLBRCY22
CBS432
IFO1815
ZP591
CBS10644
CBS7001
FM1318
Genome Position
Species
GLBRCY22
CBS432
IFO1815
ZP591
CBS10644
CBS7001
FM1318

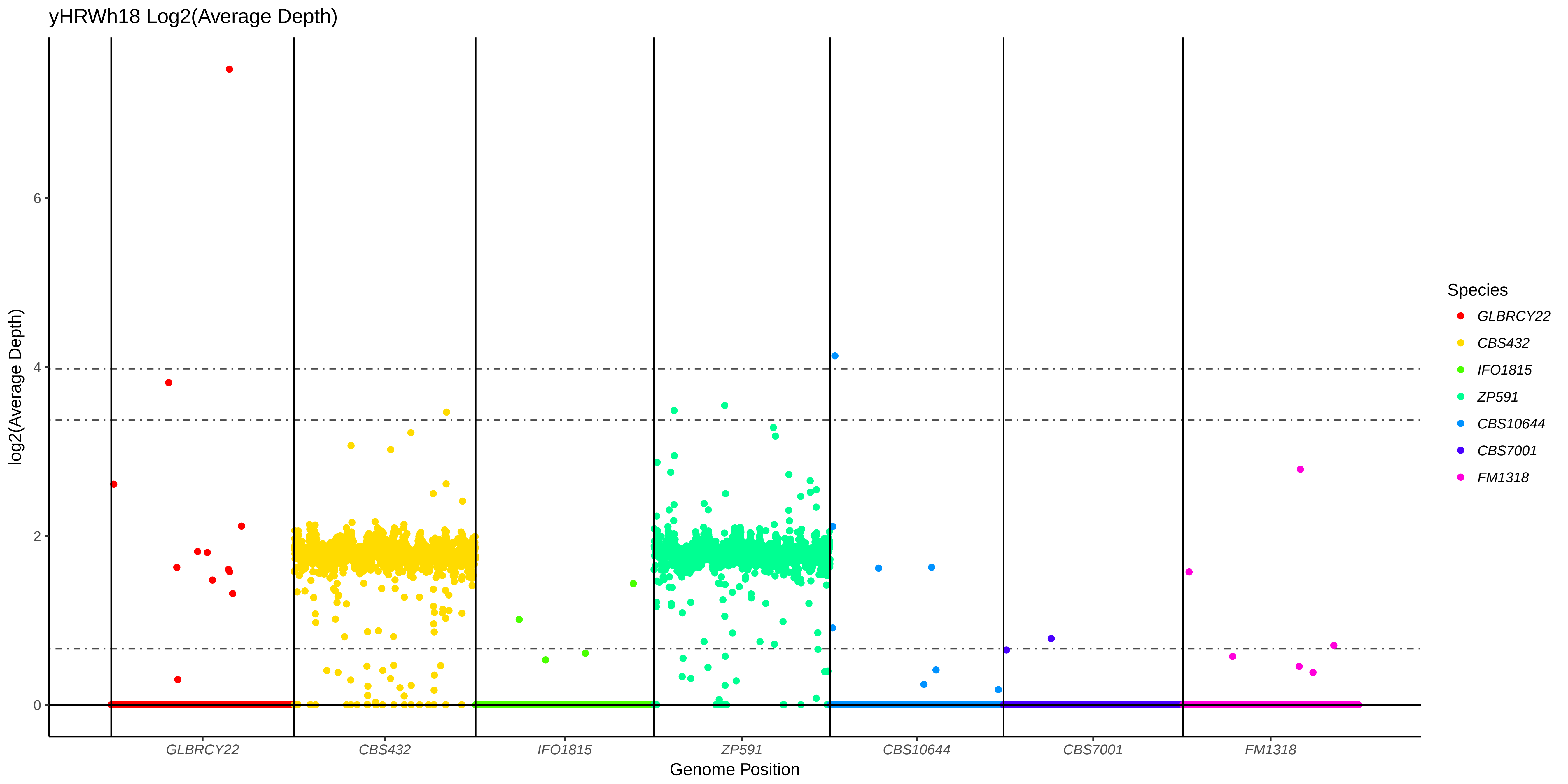

yHRWh18 Log2(Average Depth)
log2(Average Depth)
0
2
4
6
Genome Position
GLBRCY22
CBS432
IFO1815
ZP591
CBS10644
CBS7001
FM1318
Species
GLBRCY22
CBS432
IFO1815
ZP591
CBS10644
CBS7001
FM1318

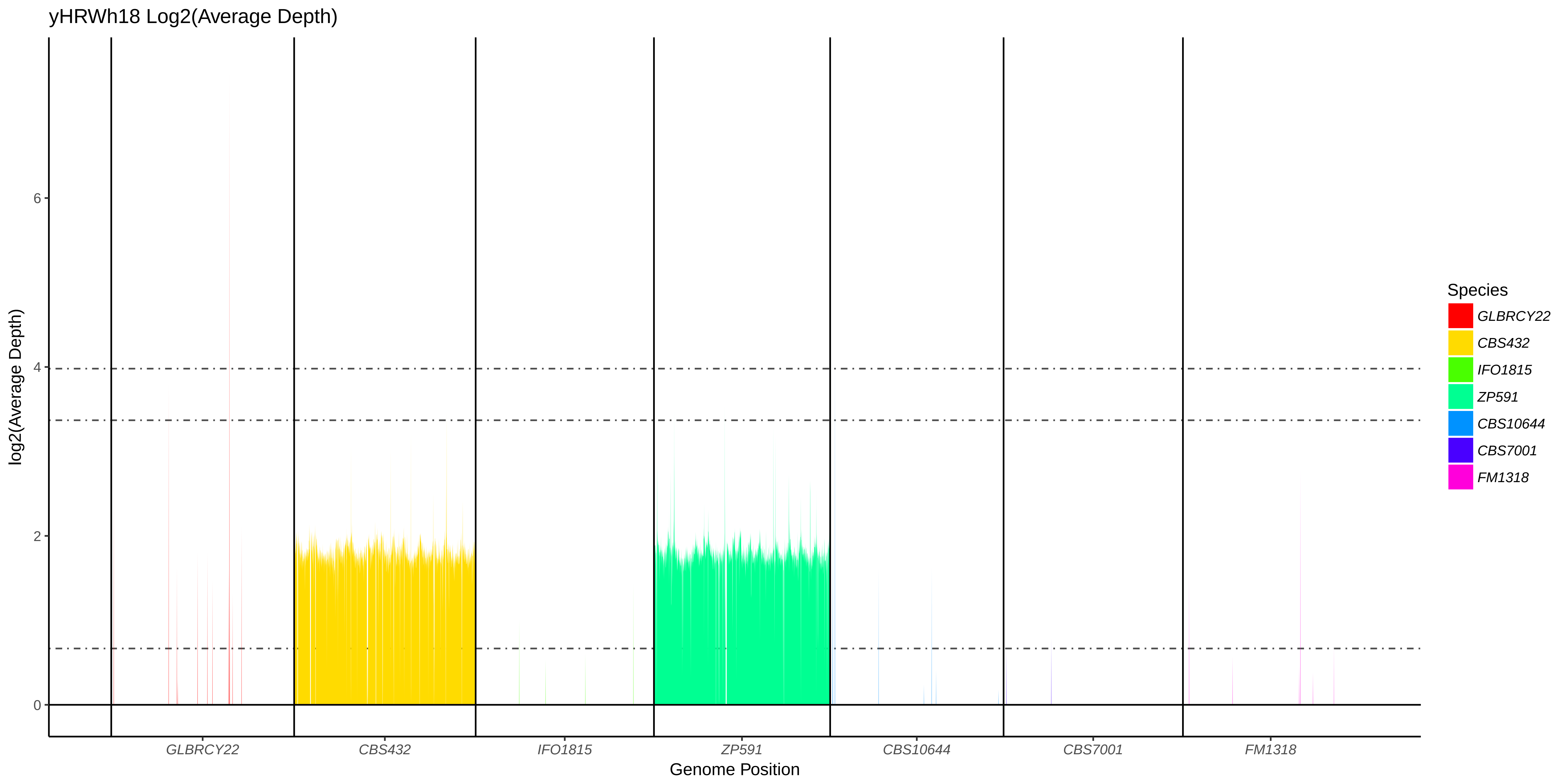

yHRWh18 Log2(Average Depth)
log2(Average Depth)
0
2
4
6
Genome Position
GLBRCY22
CBS432
IFO1815
ZP591
CBS10644
CBS7001
FM1318
Species
GLBRCY22
CBS432
IFO1815
ZP591
CBS10644
CBS7001
FM1318

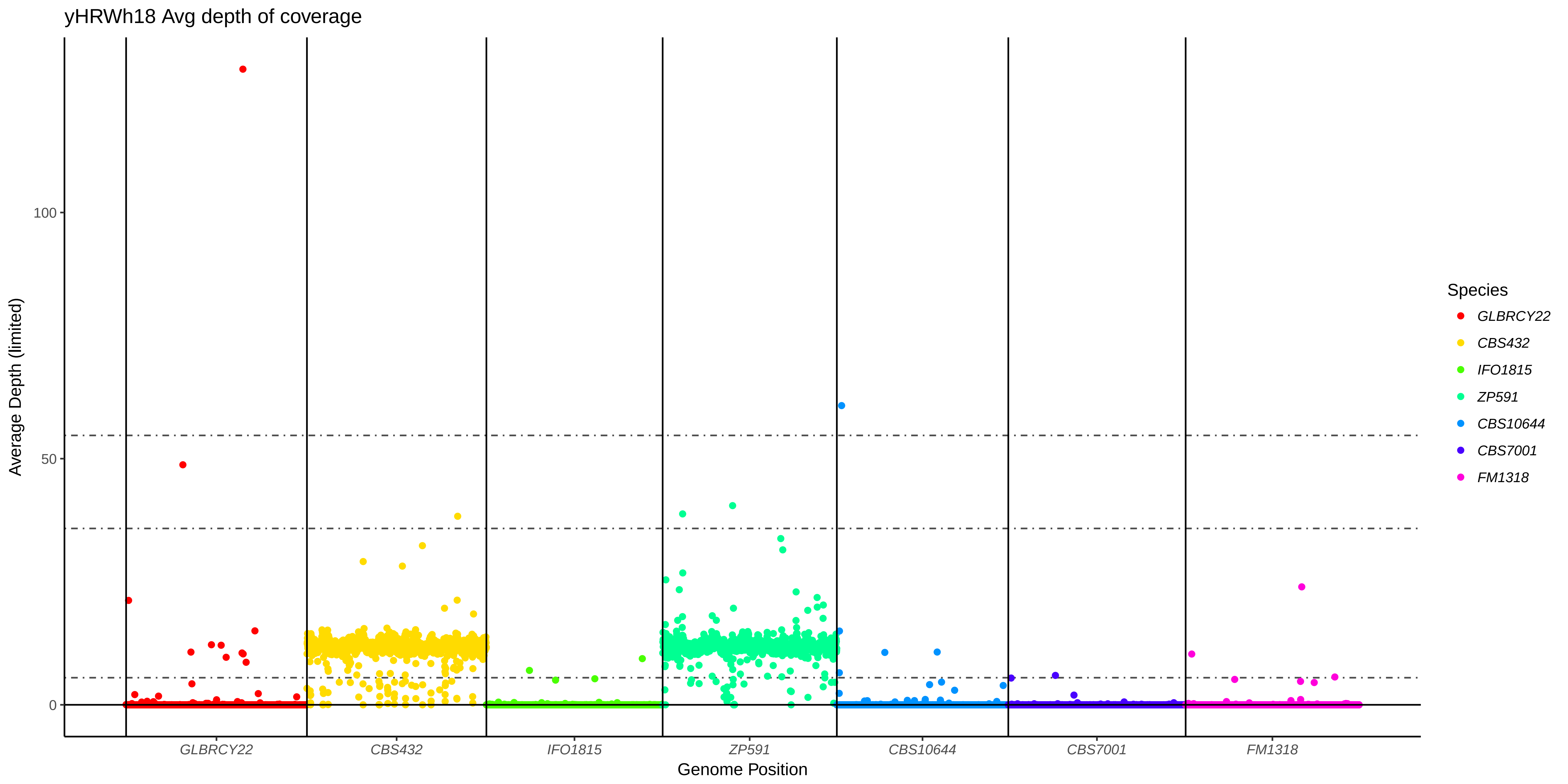
yHRWh18 Avg depth of coverage
Average Depth (limited)
100
50
0
GLBRCY22
CBS432
IFO1815
ZP591
CBS10644
CBS7001
FM1318
Genome Position
Species
GLBRCY22
CBS432
IFO1815
ZP591
CBS10644
CBS7001
FM1318

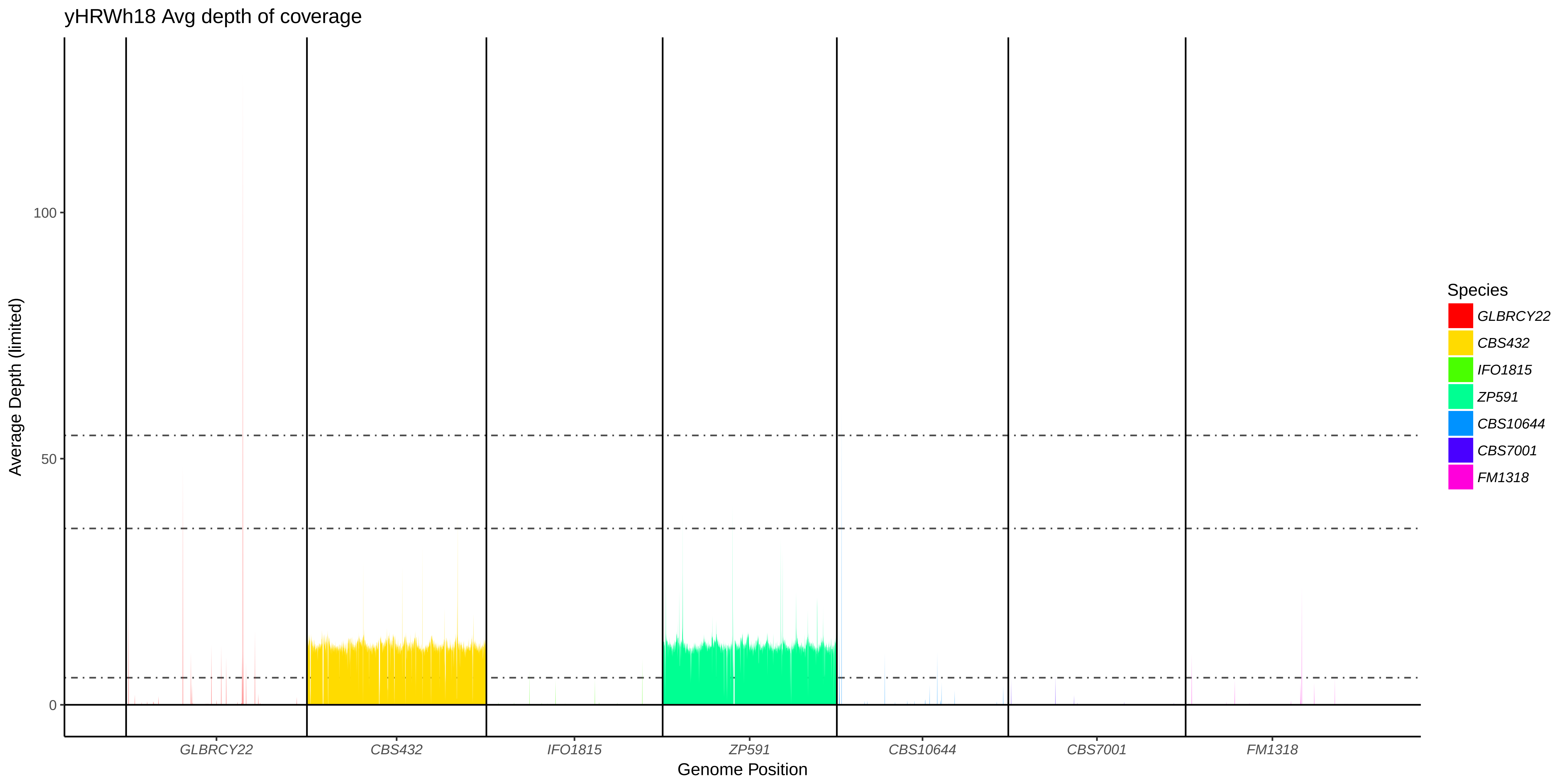

yHRWh18 Avg depth of coverage
Average Depth (limited)
100
50
0
GLBRCY22
CBS432
IFO1815
ZP591
CBS10644
CBS7001
FM1318
Genome Position
Species
GLBRCY22
CBS432
IFO1815
ZP591
CBS10644
CBS7001
FM1318

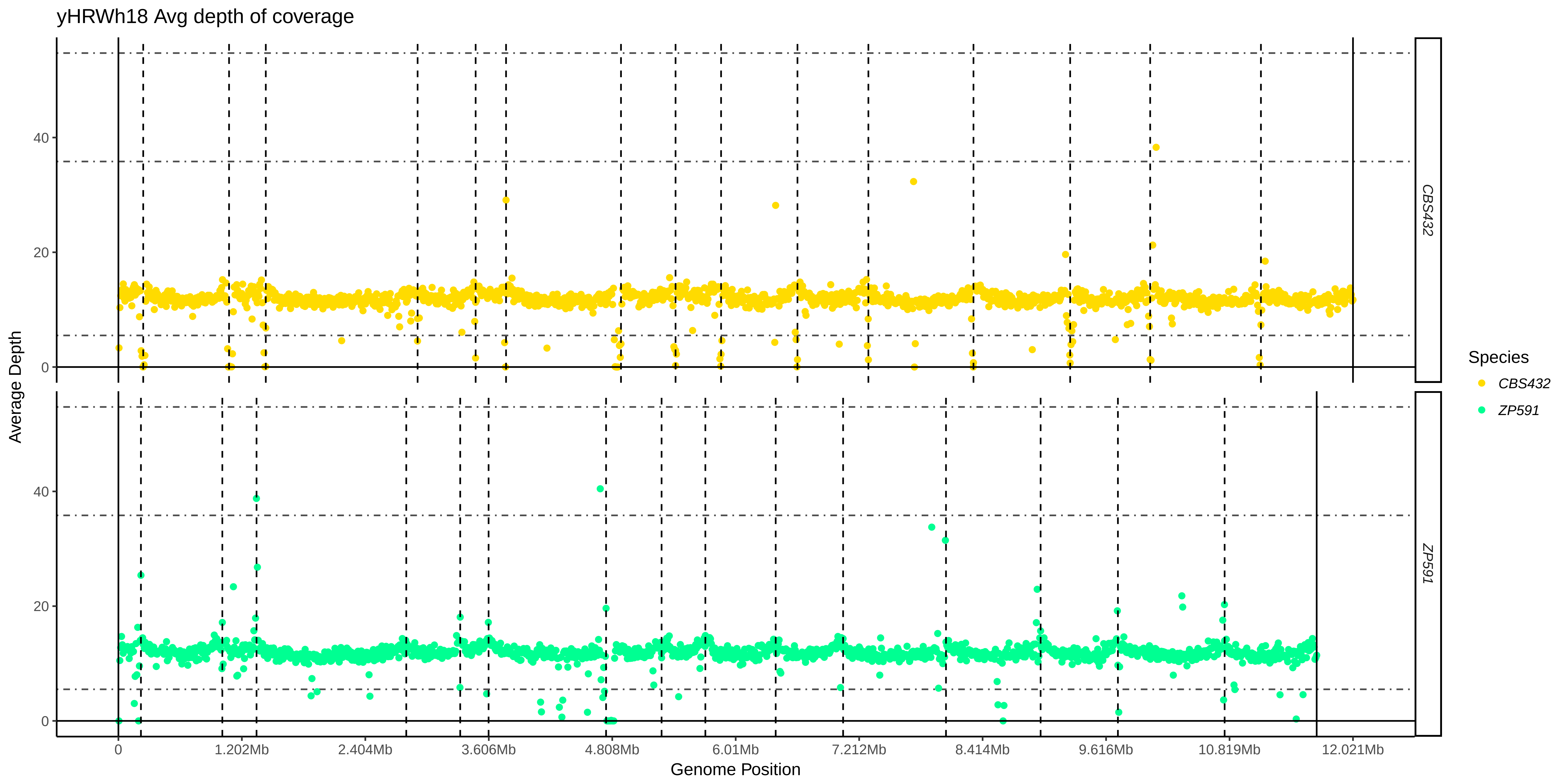

yHRWh18 Avg depth of coverage
Average Depth
0
20
40
CBS432
ZP591
0
1.202Mb
2.404Mb
3.606Mb
4.808Mb
6.01Mb
7.212Mb
8.414Mb
9.616Mb
10.819Mb
12.021Mb
Genome Position
Species
CBS432
ZP591

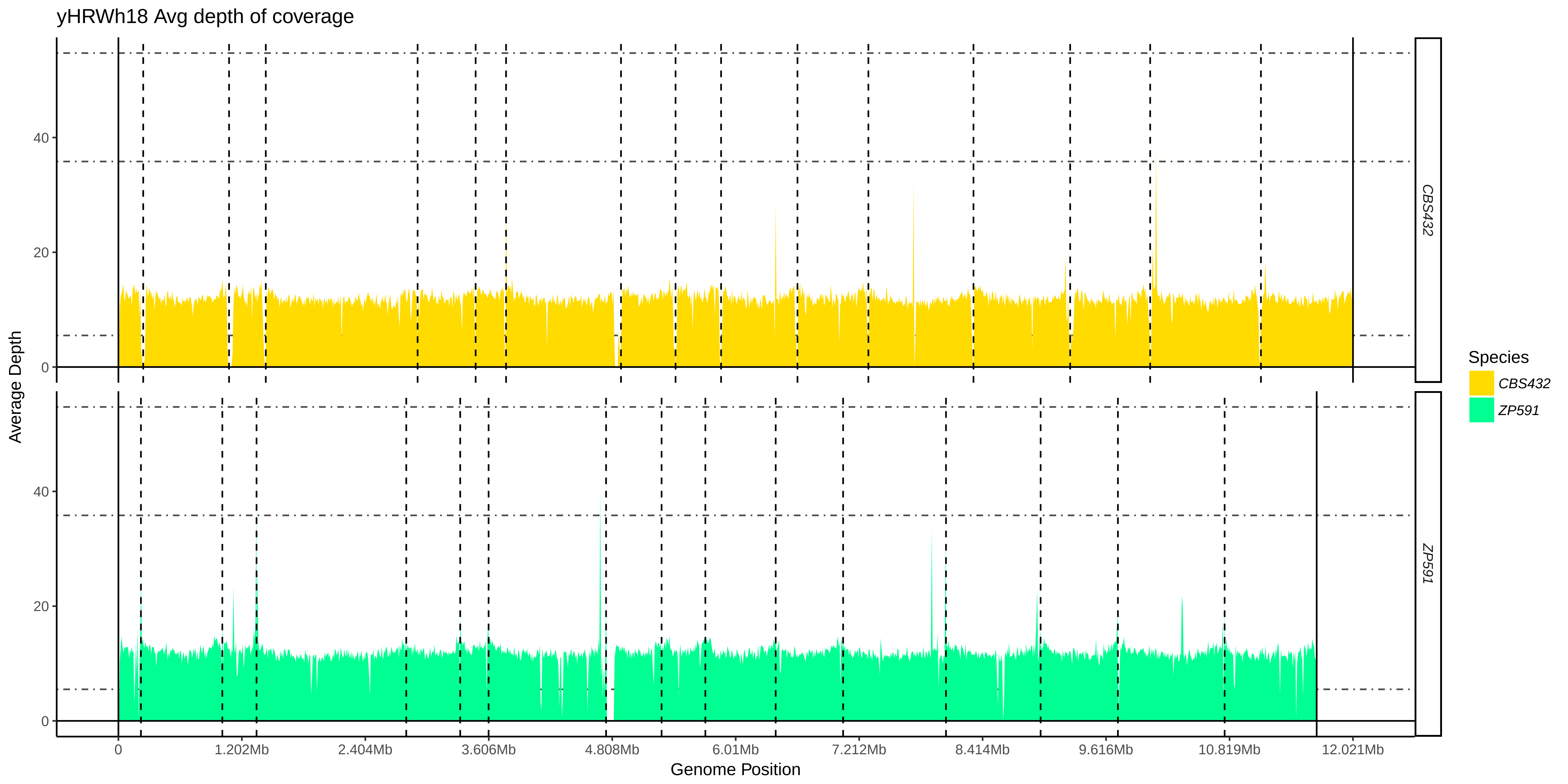

yHRWh18 Avg depth of coverage
Average Depth
0
20
40
CBS432
ZP591
Genome Position
0
1.202Mb
2.404Mb
3.606Mb
4.808Mb
6.01Mb
7.212Mb
8.414Mb
9.616Mb
10.819Mb
12.021Mb
Species
CBS432
ZP591

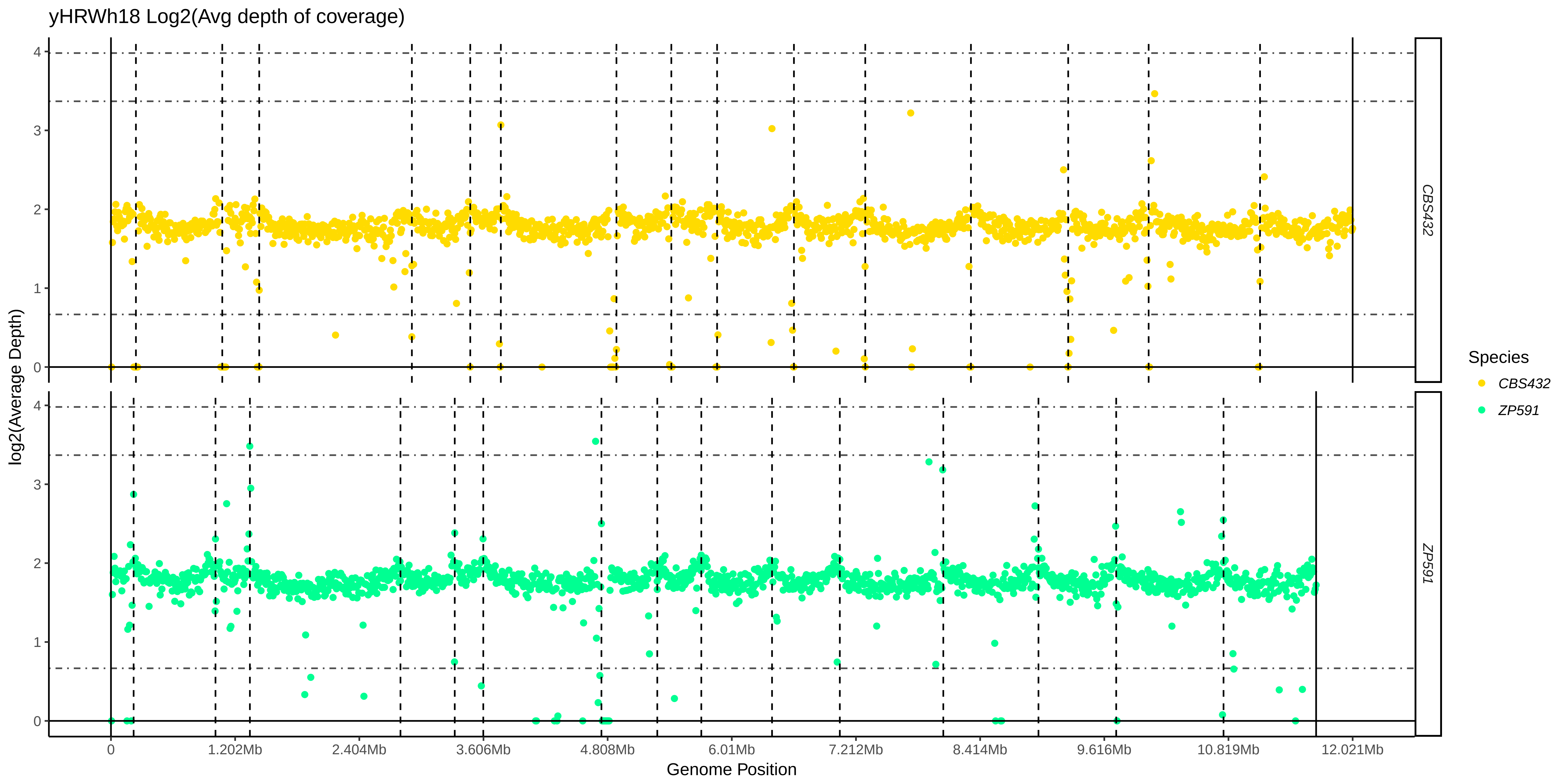

yHRWh18 Log2(Avg depth of coverage)
log2(Average Depth)
0
1
2
3
4
CBS432
ZP591
0
1.202Mb
2.404Mb
3.606Mb
4.808Mb
6.01Mb
7.212Mb
8.414Mb
9.616Mb
10.819Mb
12.021Mb
Genome Position
Species
CBS432
ZP591

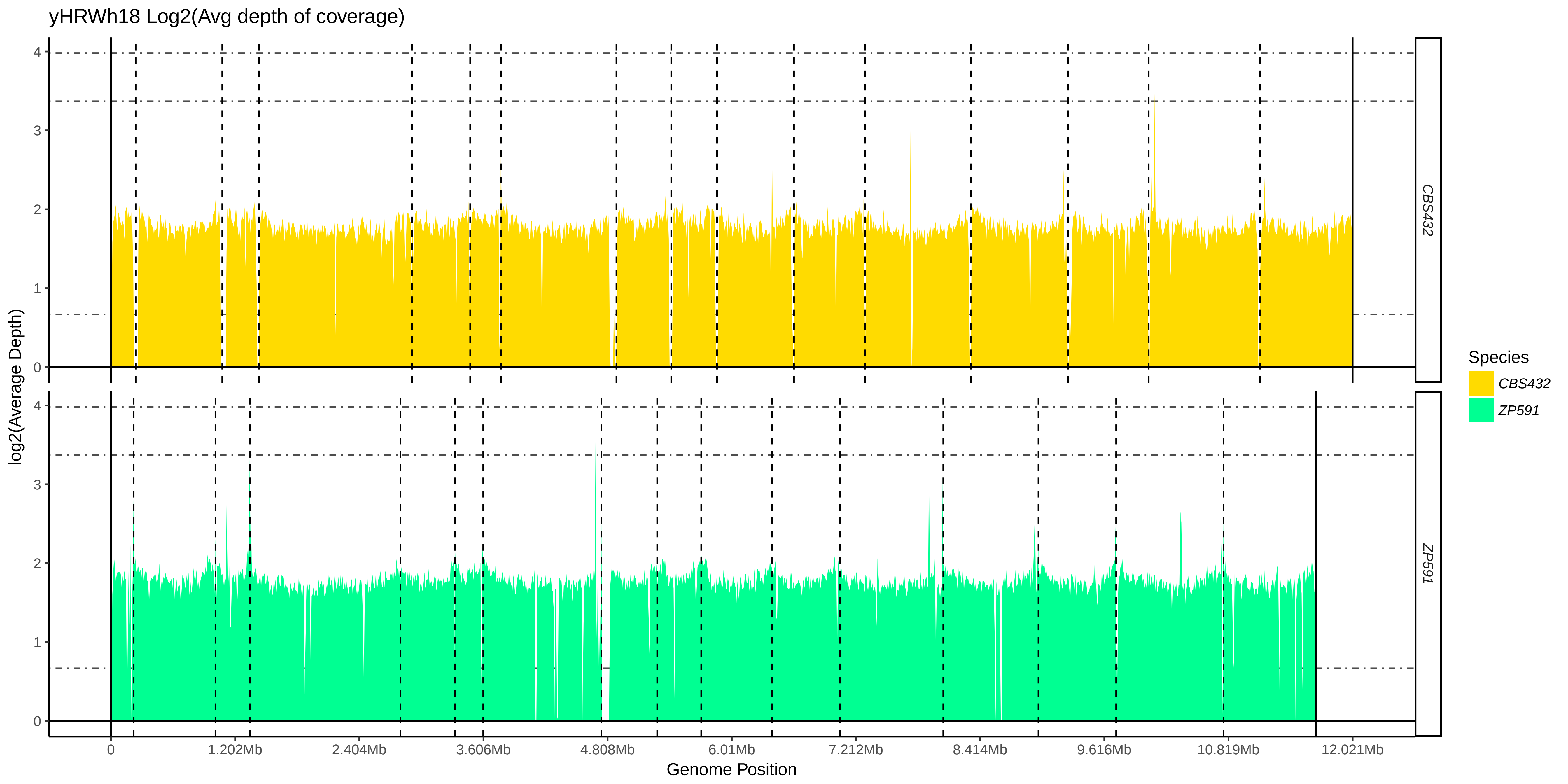
yHRWh18 Log2(Avg depth of coverage)
log2(Average Depth)
Genome Position
0
1.202Mb
2.404Mb
3.606Mb
4.808Mb
6.01Mb
7.212Mb
8.414Mb
9.616Mb
10.819Mb
12.021Mb
CBS432
ZP591
Species

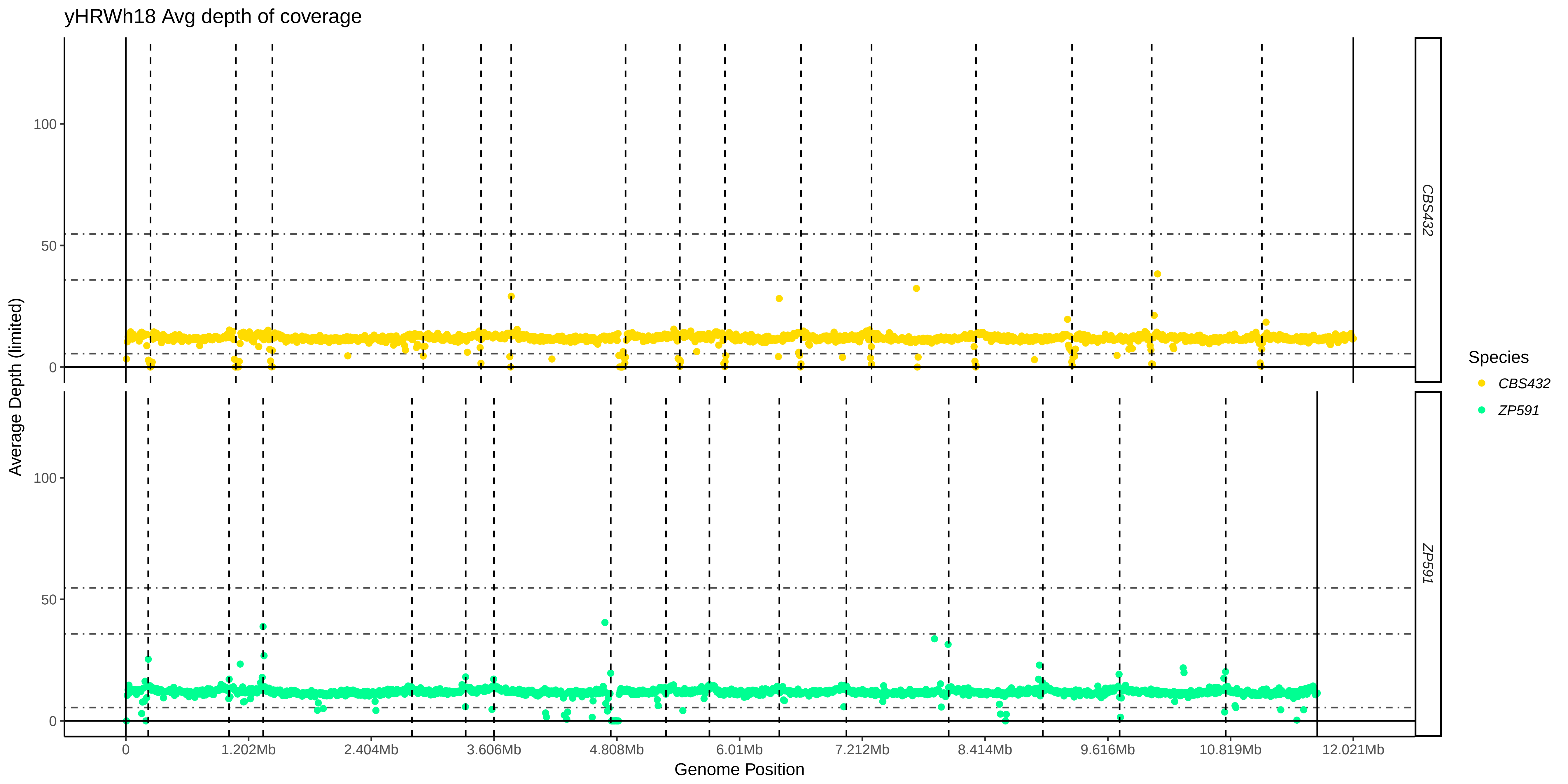
yHRWh18 Avg depth of coverage
Average Depth (limited)
100
50
0
CBS432
ZP591
Species
CBS432
ZP591
0
1.202Mb
2.404Mb
3.606Mb
4.808Mb
6.01Mb
7.212Mb
8.414Mb
9.616Mb
10.819Mb
12.021Mb
Genome Position

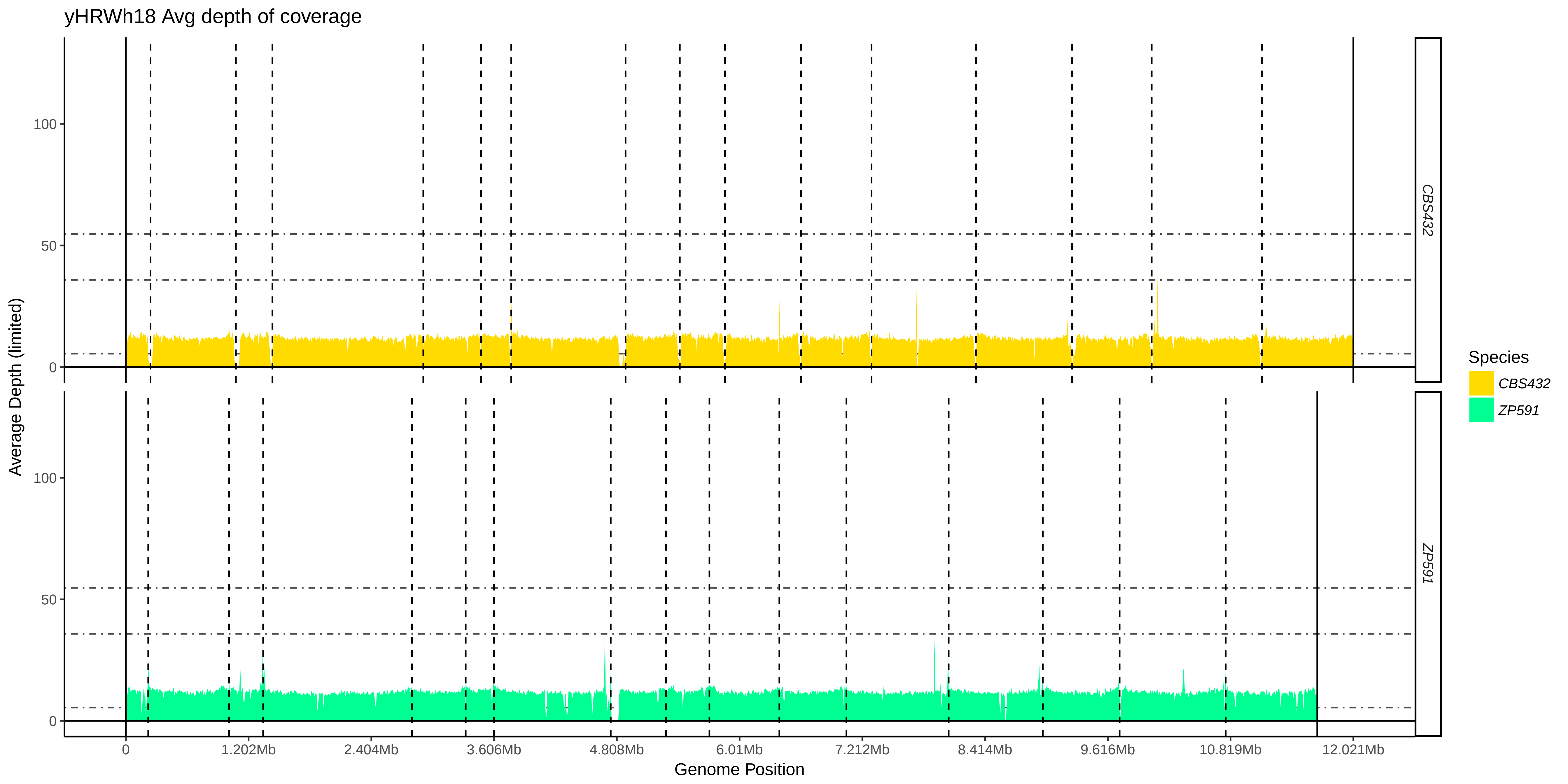

yHRWh18 Avg depth of coverage
Average Depth (limited)
100
50
0
Genome Position
0
1.202Mb
2.404Mb
3.606Mb
4.808Mb
6.01Mb
7.212Mb
8.414Mb
9.616Mb
10.819Mb
12.021Mb
CBS432
ZP591
Species
CBS432
ZP591
