# Supplementary material for: Synthetic hybrids of six yeast species
Source: Nat Commun. 2020 Apr 29;11:2085. doi: 10.1038/s41467-020-15559-4 (PMC7190663; doi:10.1038/s41467-020-15559-4)
Supplement: Supplementary file 15 — Source Data [file 41467_2020_15559_MOESM15_ESM.zip › Source Data 2.zip!/yHRWh24_sppIDerDepthPlot-covBins.pdf]

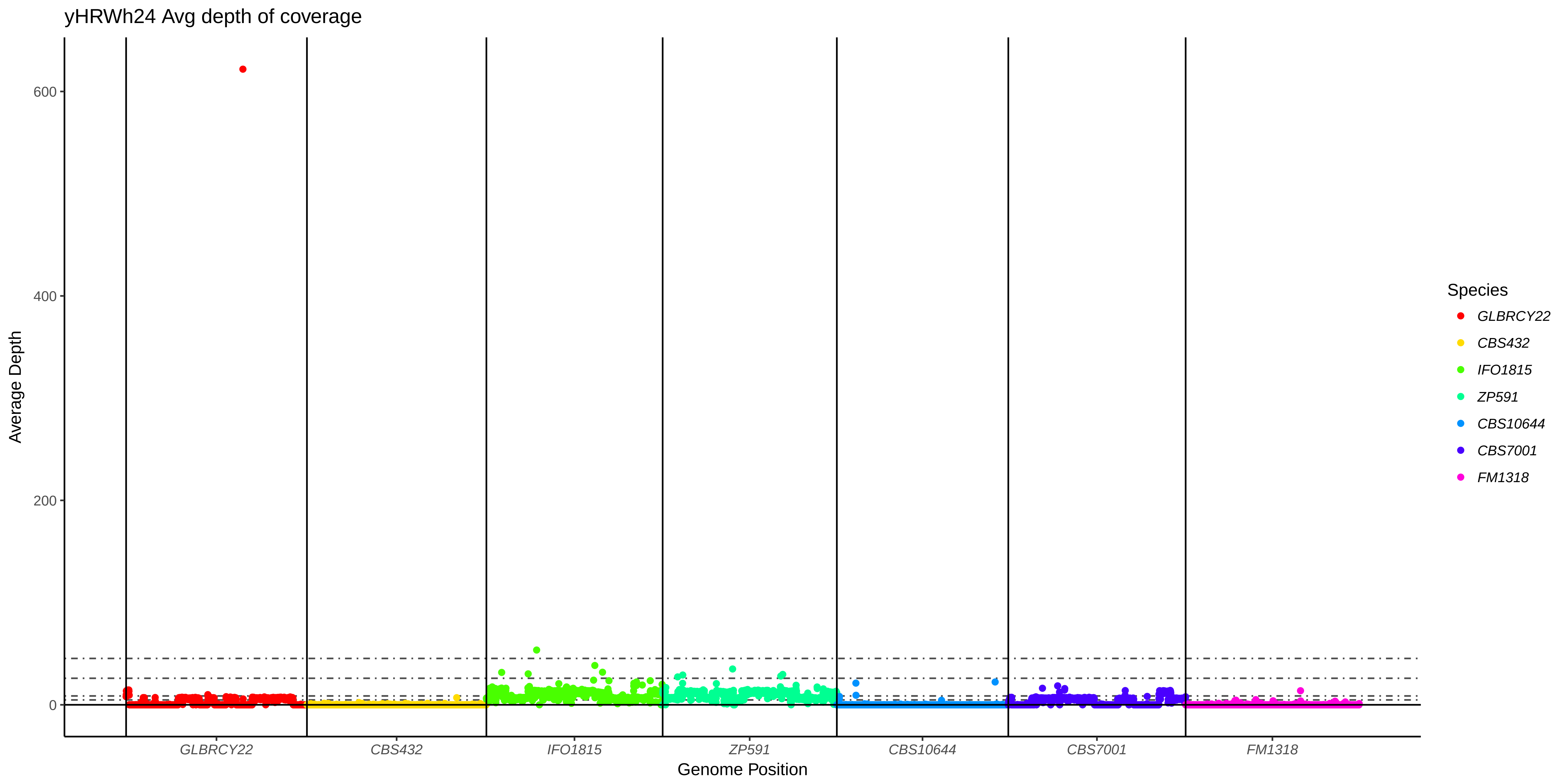

yHRWh24 Avg depth of coverage
600
400
200
0
Average Depth
GLBRCY22
CBS432
IFO1815
ZP591
CBS10644
CBS7001
FM1318
Genome Position
Species
GLBRCY22
CBS432
IFO1815
ZP591
CBS10644
CBS7001
FM1318

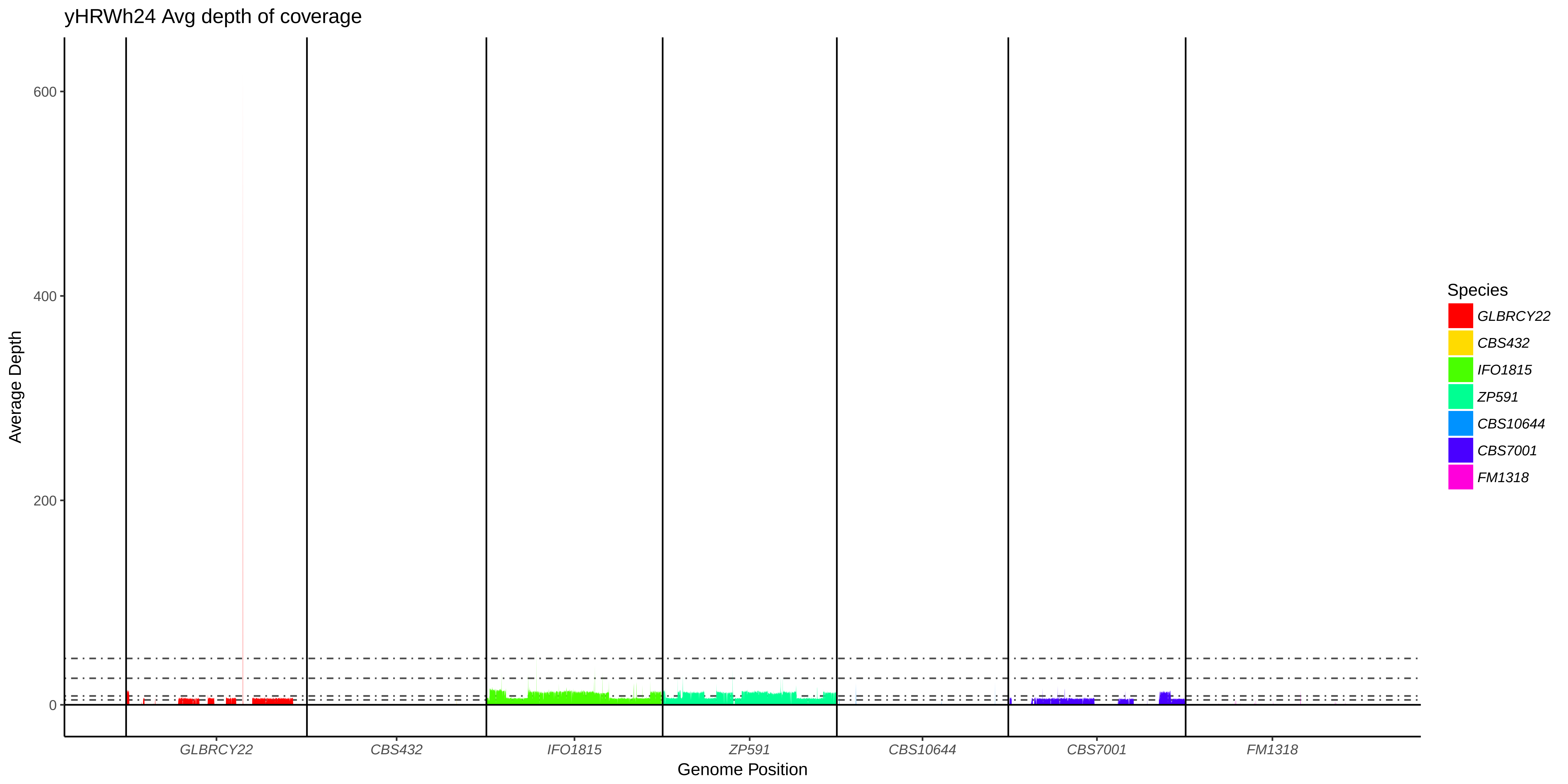

yHRWh24 Avg depth of coverage
Average Depth
600
400
200
0
GLBRCY22
CBS432
IFO1815
ZP591
CBS10644
CBS7001
FM1318
Genome Position
Species
GLBRCY22
CBS432
IFO1815
ZP591
CBS10644
CBS7001
FM1318

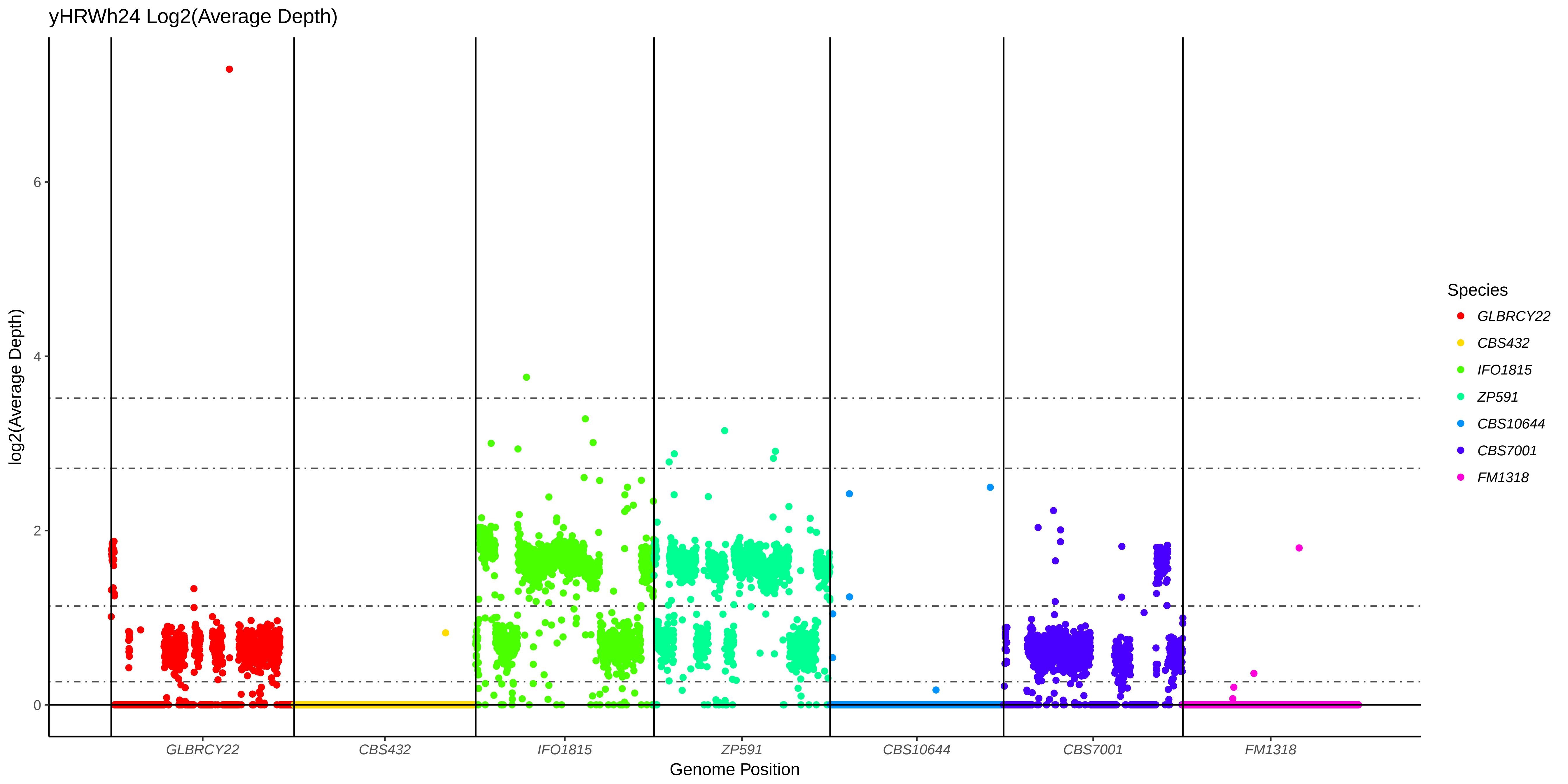

yHRWh24 Log2(Average Depth)
log2(Average Depth)
0
2
4
6
GLBRCY22
CBS432
IFO1815
ZP591
CBS10644
CBS7001
FM1318
Genome Position
Species
GLBRCY22
CBS432
IFO1815
ZP591
CBS10644
CBS7001
FM1318

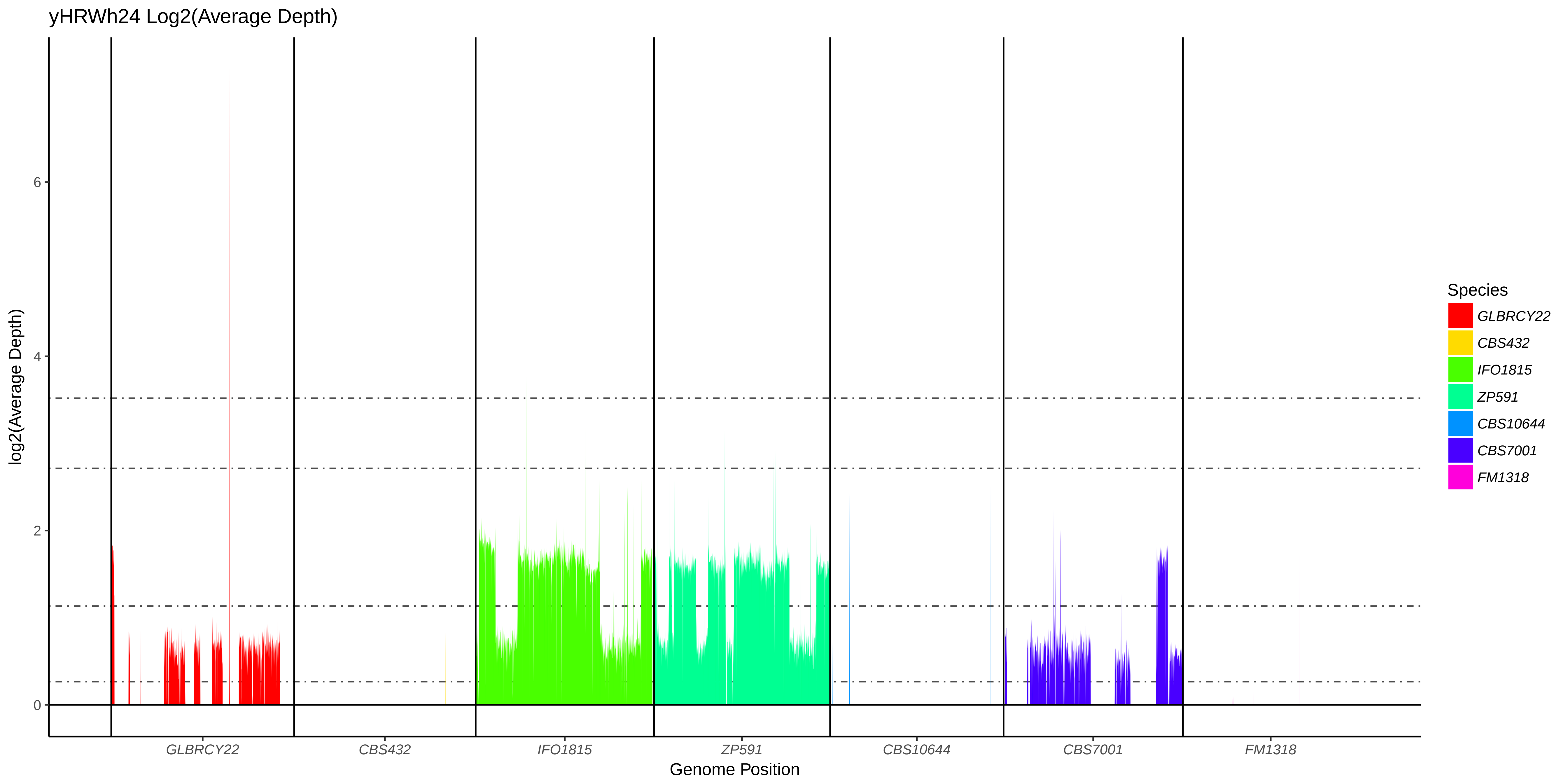

yHRWh24 Log2(Average Depth)
log2(Average Depth)
0
2
4
6
GLBRCY22
CBS432
IFO1815
ZP591
CBS10644
CBS7001
FM1318
Genome Position
Species
GLBRCY22
CBS432
IFO1815
ZP591
CBS10644
CBS7001
FM1318

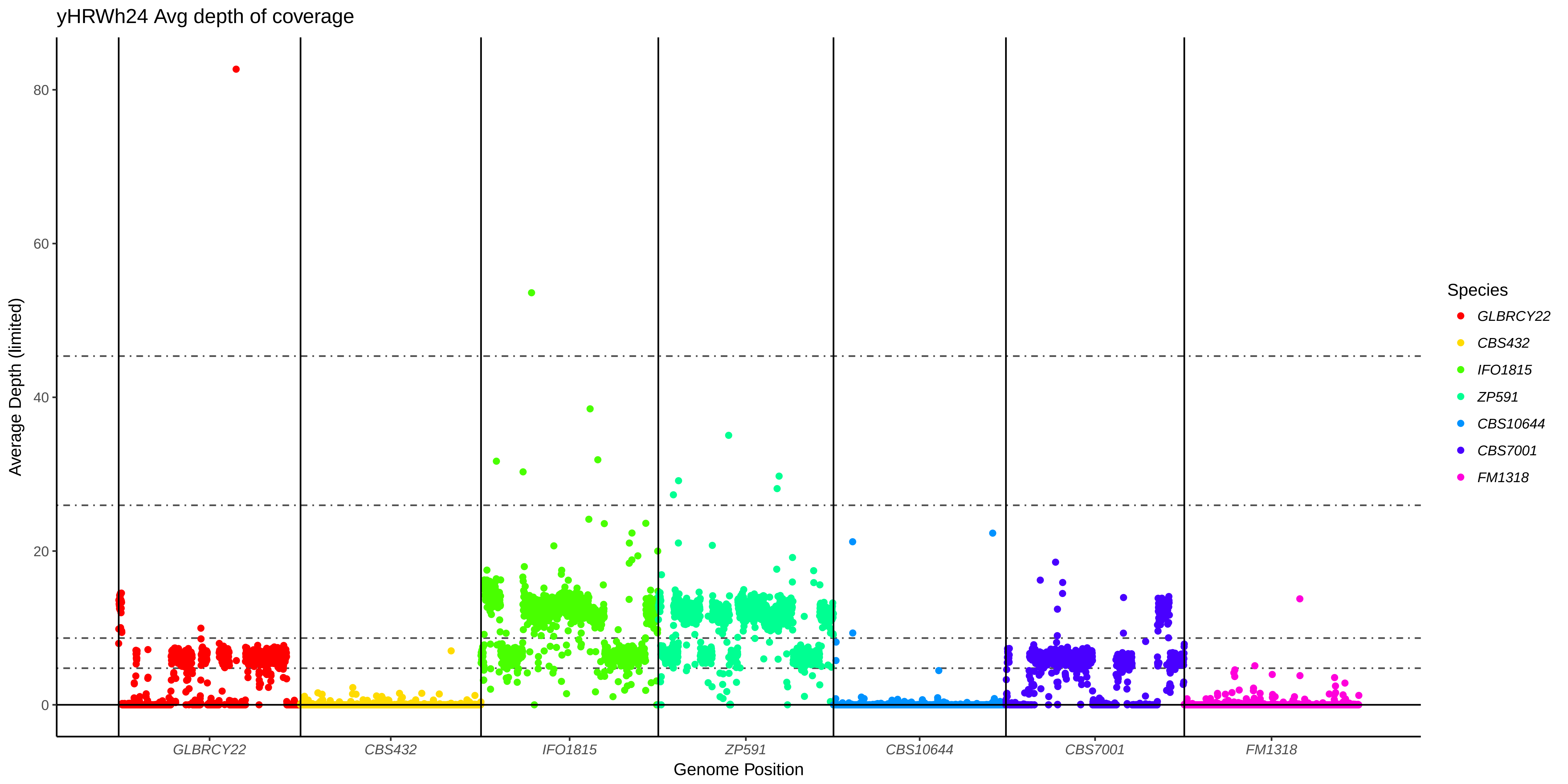

yHRWh24 Avg depth of coverage
Average Depth (limited)
80
60
40
20
0
GLBRCY22
CBS432
IFO1815
ZP591
CBS10644
CBS7001
FM1318
Genome Position
Species
GLBRCY22
CBS432
IFO1815
ZP591
CBS10644
CBS7001
FM1318

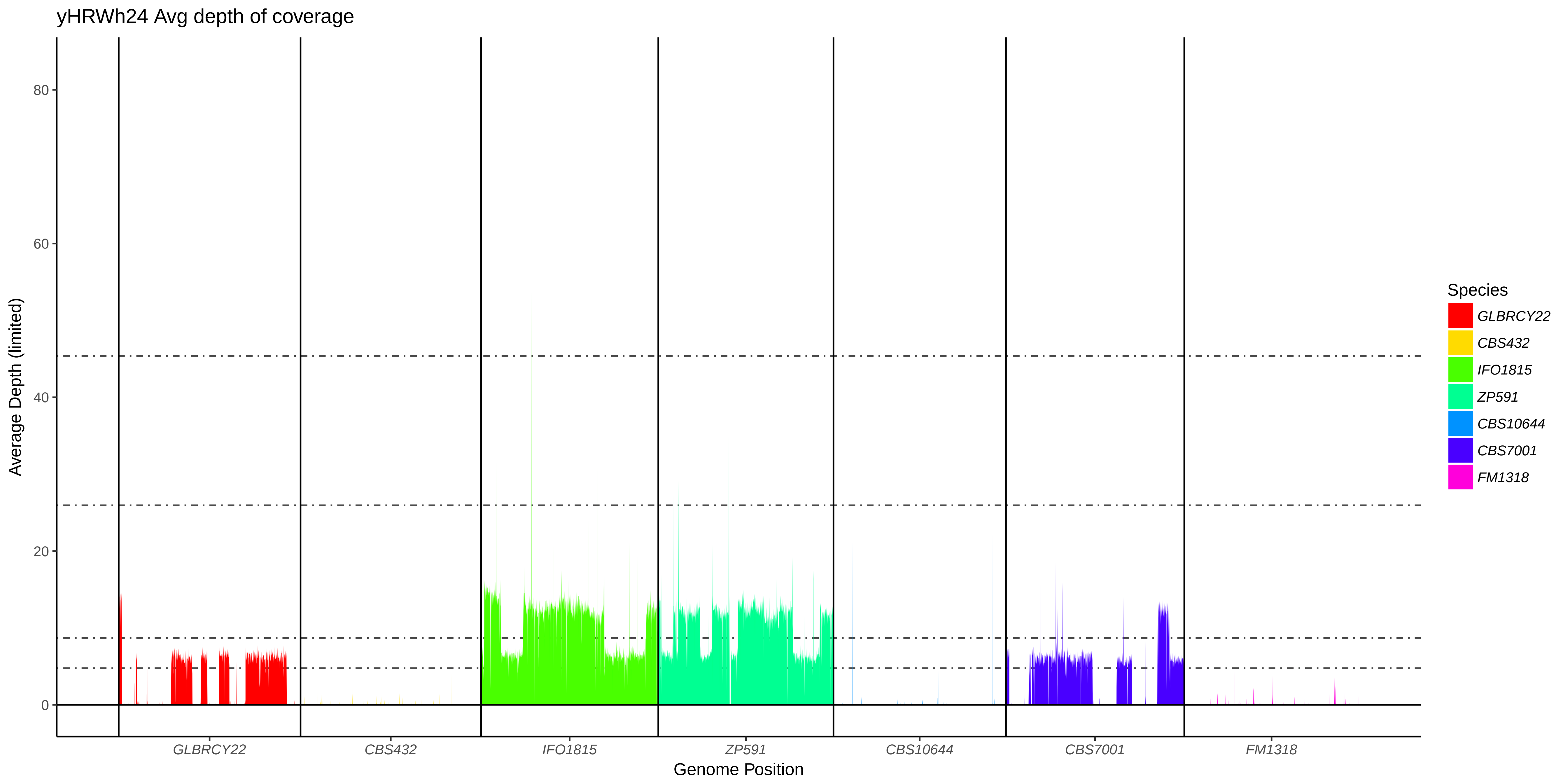
yHRWh24 Avg depth of coverage
Average Depth (limited)
80
60
40
20
0
GLBRCY22
CBS432
IFO1815
ZP591
CBS10644
CBS7001
FM1318
Genome Position
Species
GLBRCY22
CBS432
IFO1815
ZP591
CBS10644
CBS7001
FM1318

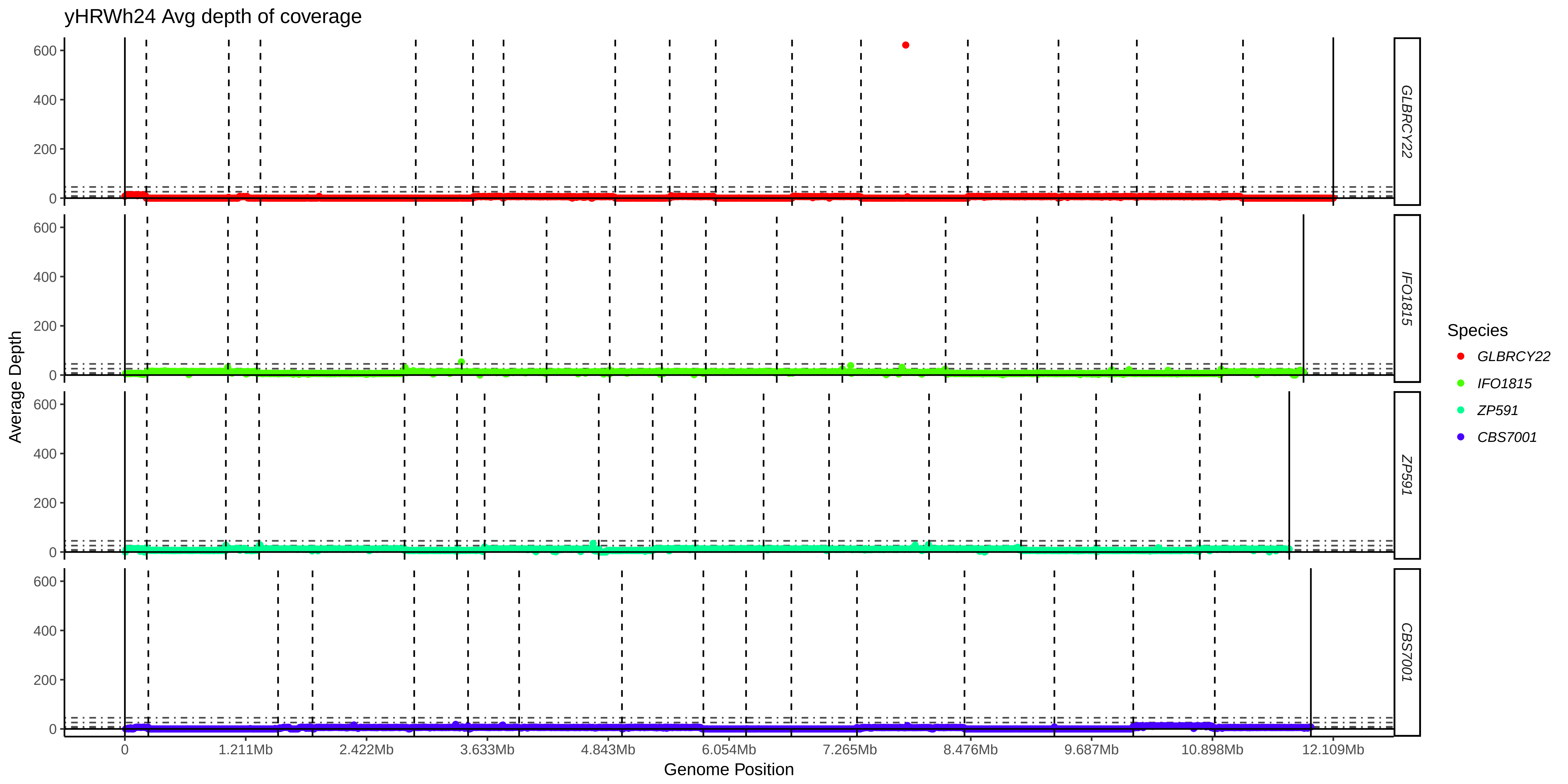

yHRWh24 Avg depth of coverage
Average Depth
Genome Position
0
200
400
600
0
1.211Mb
2.422Mb
3.633Mb
4.843Mb
6.054Mb
7.265Mb
8.476Mb
9.687Mb
10.898Mb
12.109Mb
GLBRCY22
IFO1815
ZP591
CBS7001
Species
GLBRCY22
IFO1815
ZP591
CBS7001

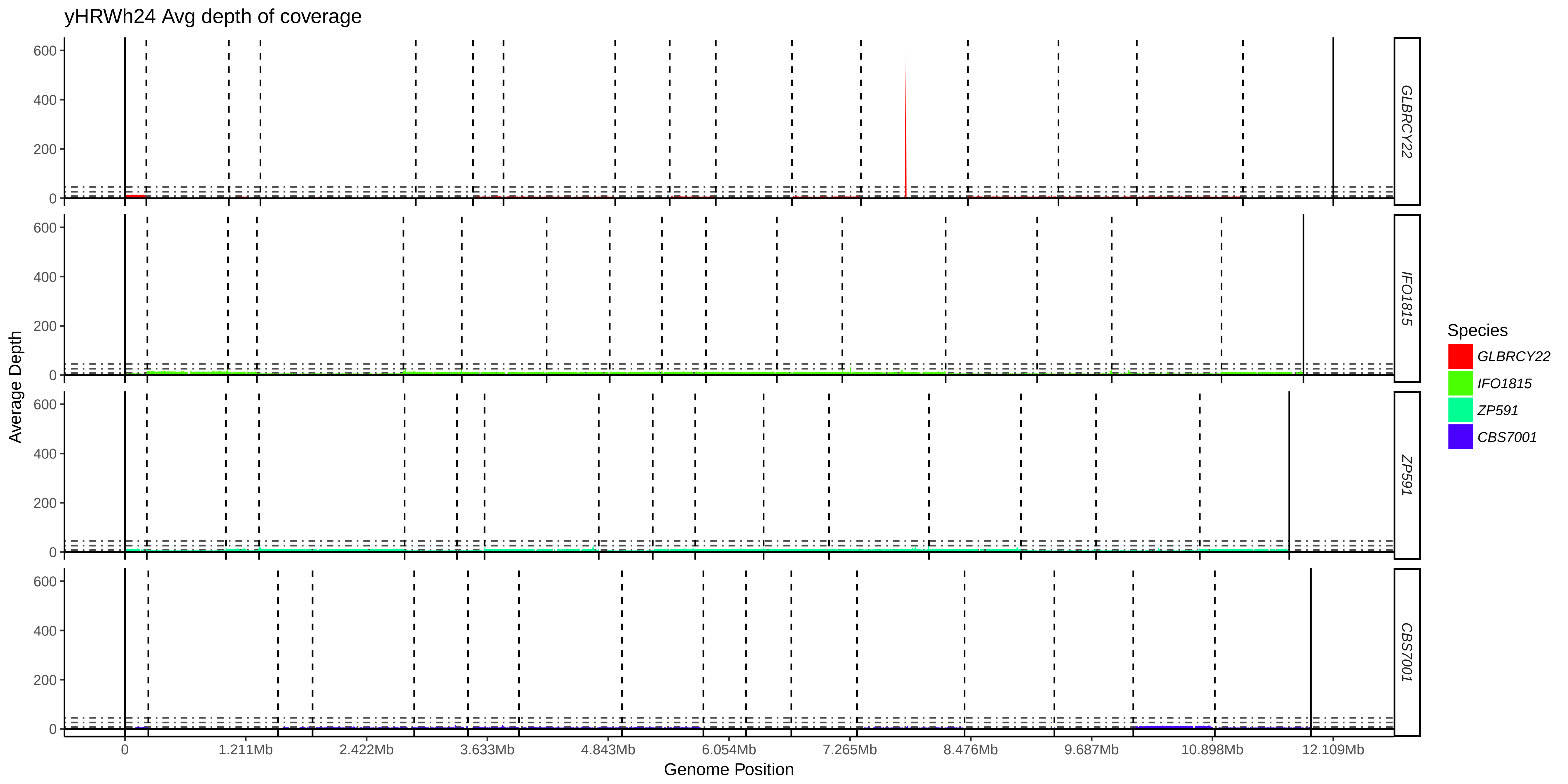

yHRWh24 Avg depth of coverage
Average Depth
Genome Position
0
200
400
600
0
1.211Mb
2.422Mb
3.633Mb
4.843Mb
6.054Mb
7.265Mb
8.476Mb
9.687Mb
10.898Mb
12.109Mb
GLBRCY22
IFO1815
ZP591
CBS7001
Species
GLBRCY22
IFO1815
ZP591
CBS7001

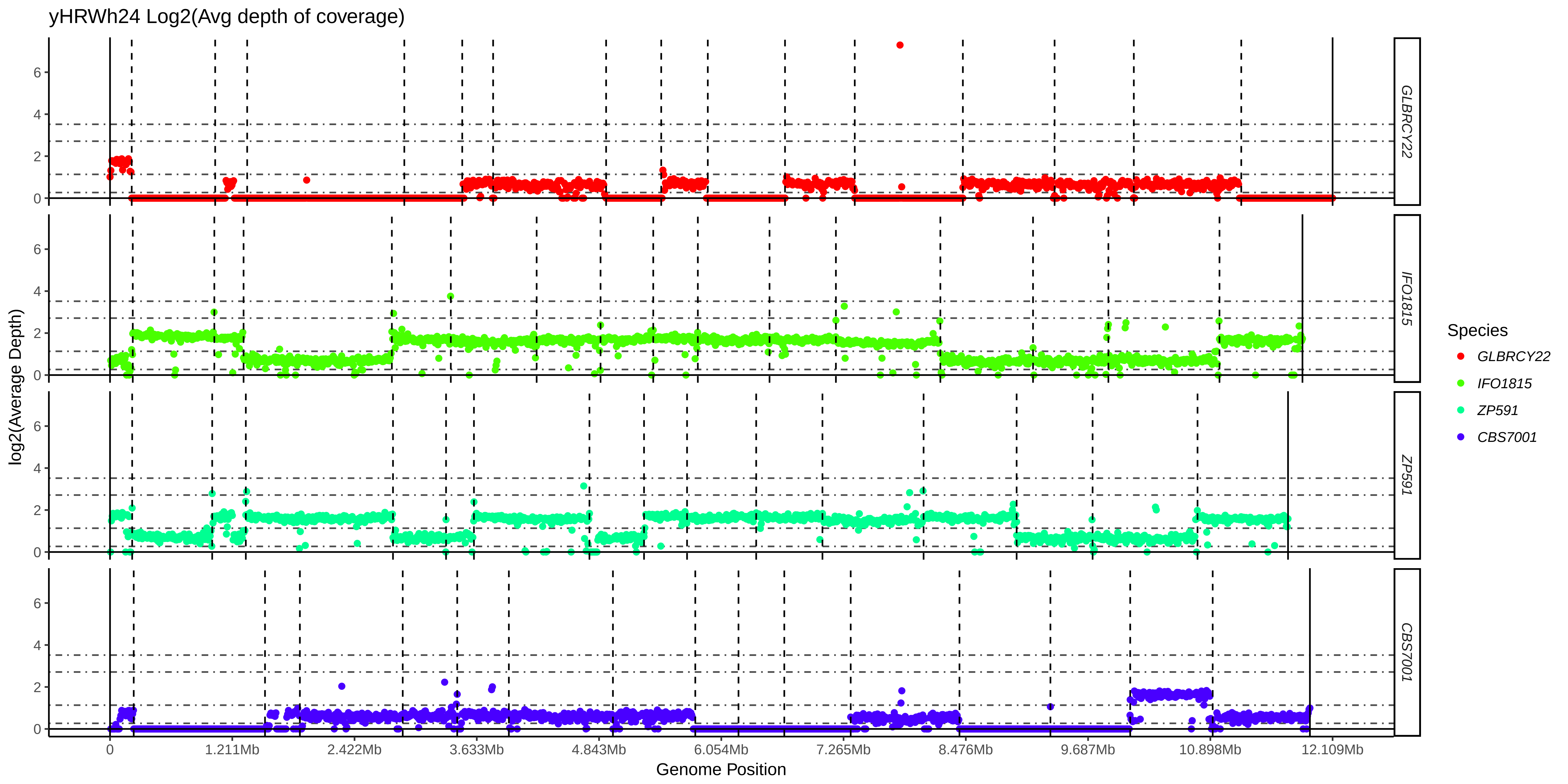

yHRWh24 Log2(Avg depth of coverage)
log2(Average Depth)
Genome Position
0
1.211Mb
2.422Mb
3.633Mb
4.843Mb
6.054Mb
7.265Mb
8.476Mb
9.687Mb
10.898Mb
12.109Mb
0
2
4
6
GLBRCY22
IFO1815
ZP591
CBS7001
Species
GLBRCY22
IFO1815
ZP591
CBS7001

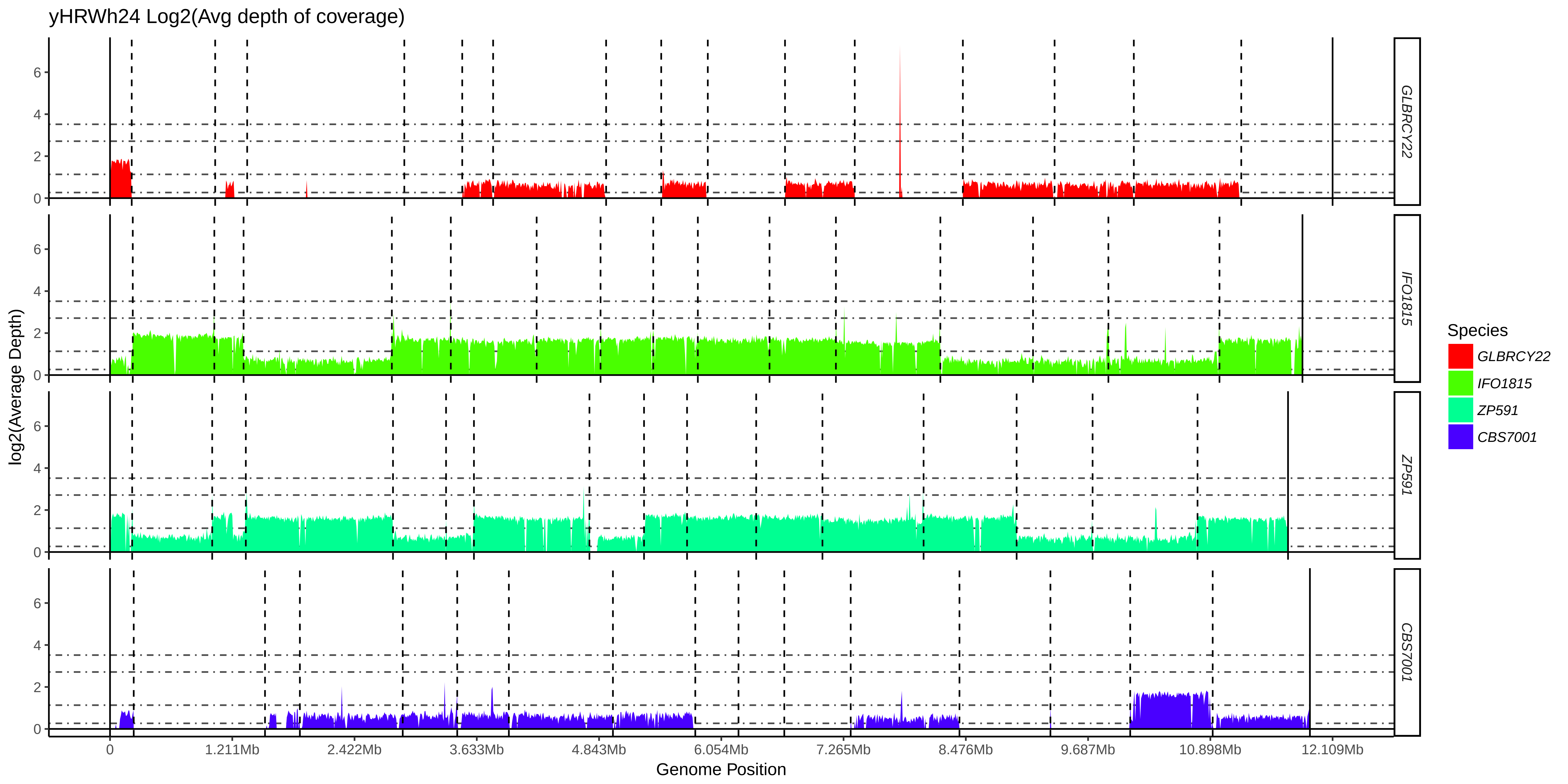

yHRWh24 Log2(Avg depth of coverage)
log2(Average Depth)
Genome Position
0
1.211Mb
2.422Mb
3.633Mb
4.843Mb
6.054Mb
7.265Mb
8.476Mb
9.687Mb
10.898Mb
12.109Mb
GLBRCY22
IFO1815
ZP591
CBS7001
Species

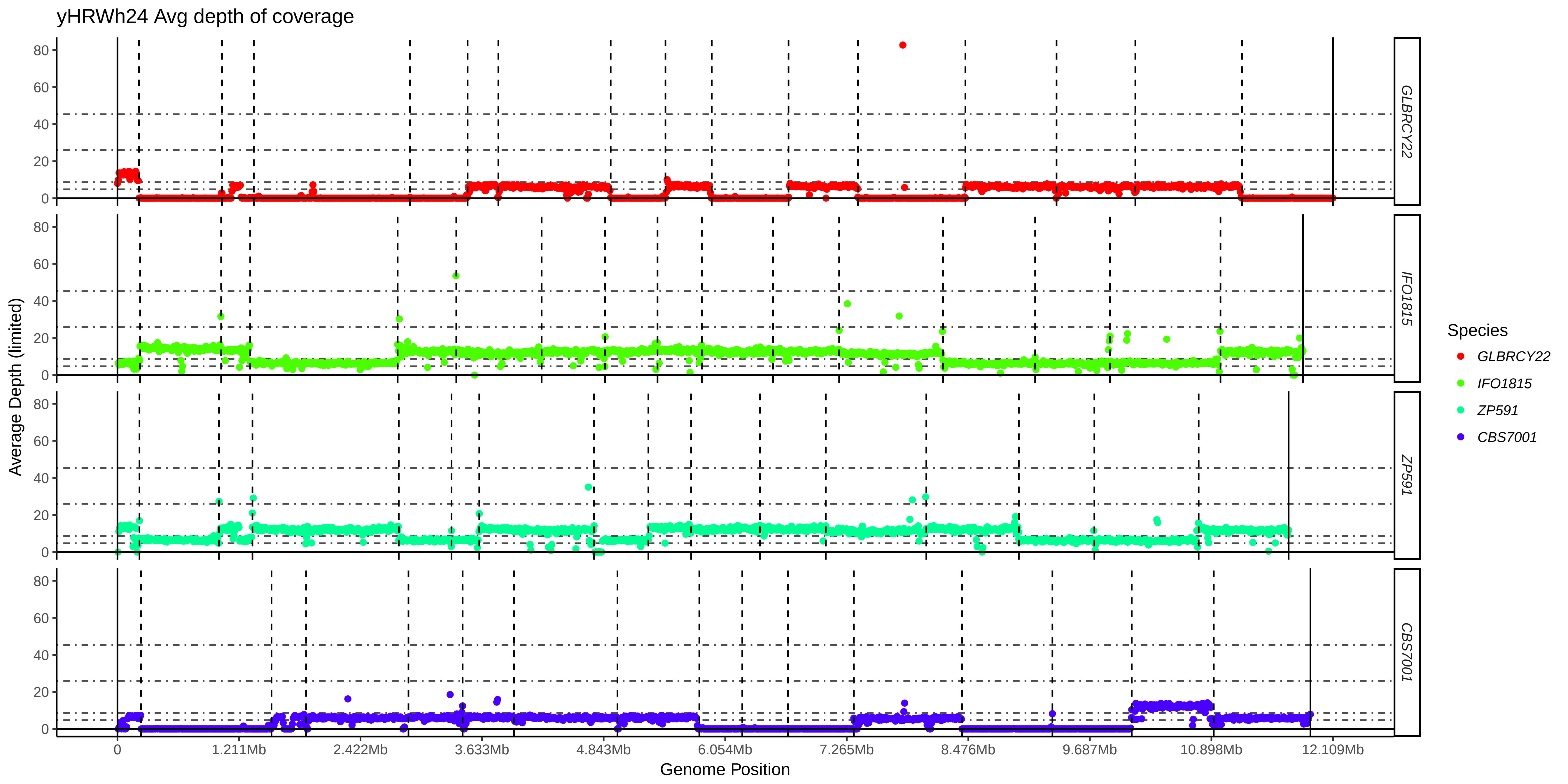

yHRWh24 Avg depth of coverage
Average Depth (limited)
80
60
40
20
0
GLBRCY22
IFO1815
ZP591
CBS7001
0
1.211Mb
2.422Mb
3.633Mb
4.843Mb
6.054Mb
7.265Mb
8.476Mb
9.687Mb
10.898Mb
12.109Mb
Genome Position
Species
GLBRCY22
IFO1815
ZP591
CBS7001

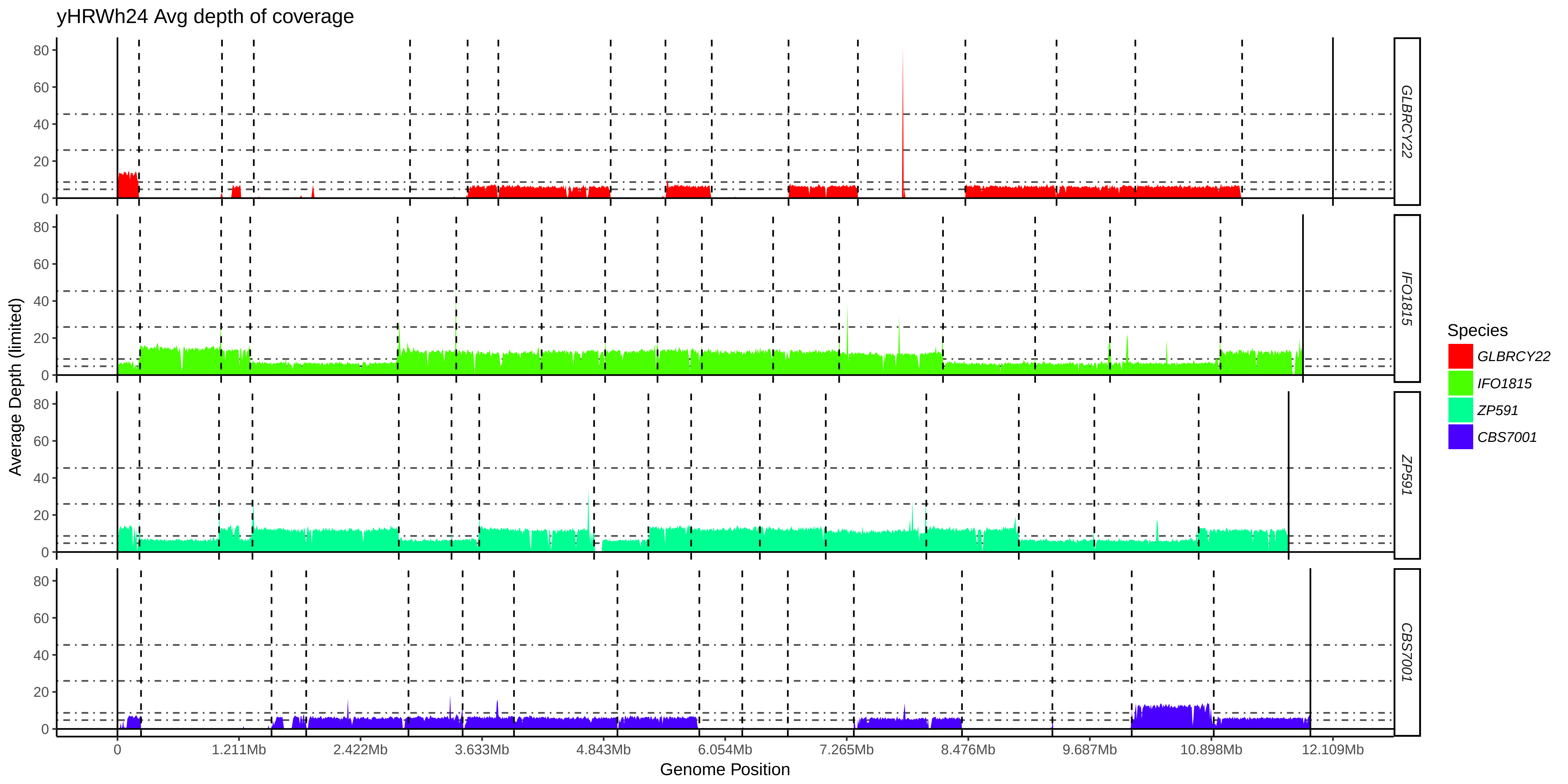

yHRWh24 Avg depth of coverage
Average Depth (limited)
Genome Position
0
1.211Mb
2.422Mb
3.633Mb
4.843Mb
6.054Mb
7.265Mb
8.476Mb
9.687Mb
10.898Mb
12.109Mb
0
20
40
60
80
GLBRCY22
IFO1815
ZP591
CBS7001
Species
GLBRCY22
IFO1815
ZP591
CBS7001
