# Supplementary material for: Synthetic hybrids of six yeast species
Source: Nat Commun. 2020 Apr 29;11:2085. doi: 10.1038/s41467-020-15559-4 (PMC7190663; doi:10.1038/s41467-020-15559-4)
Supplement: Supplementary file 15 — Source Data [file 41467_2020_15559_MOESM15_ESM.zip › Source Data 2.zip!/yHRWh36_sppIDerDepthPlot-covBins.pdf]

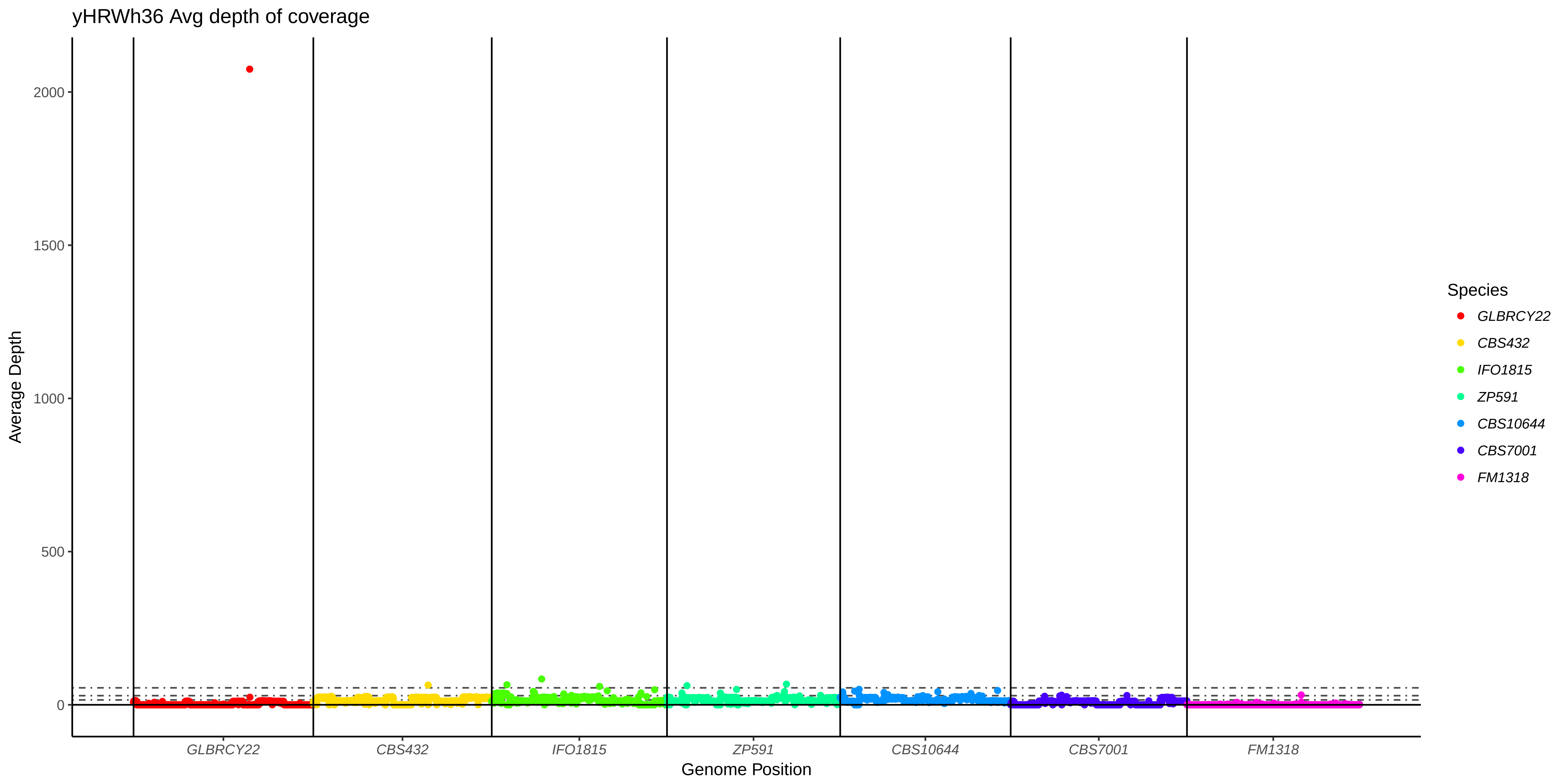

yHRWh36 Avg depth of coverage
Average Depth
2000
1500
1000
500
0
GLBRCY22
CBS432
IFO1815
ZP591
CBS10644
CBS7001
FM1318
Genome Position
Species
GLBRCY22
CBS432
IFO1815
ZP591
CBS10644
CBS7001
FM1318

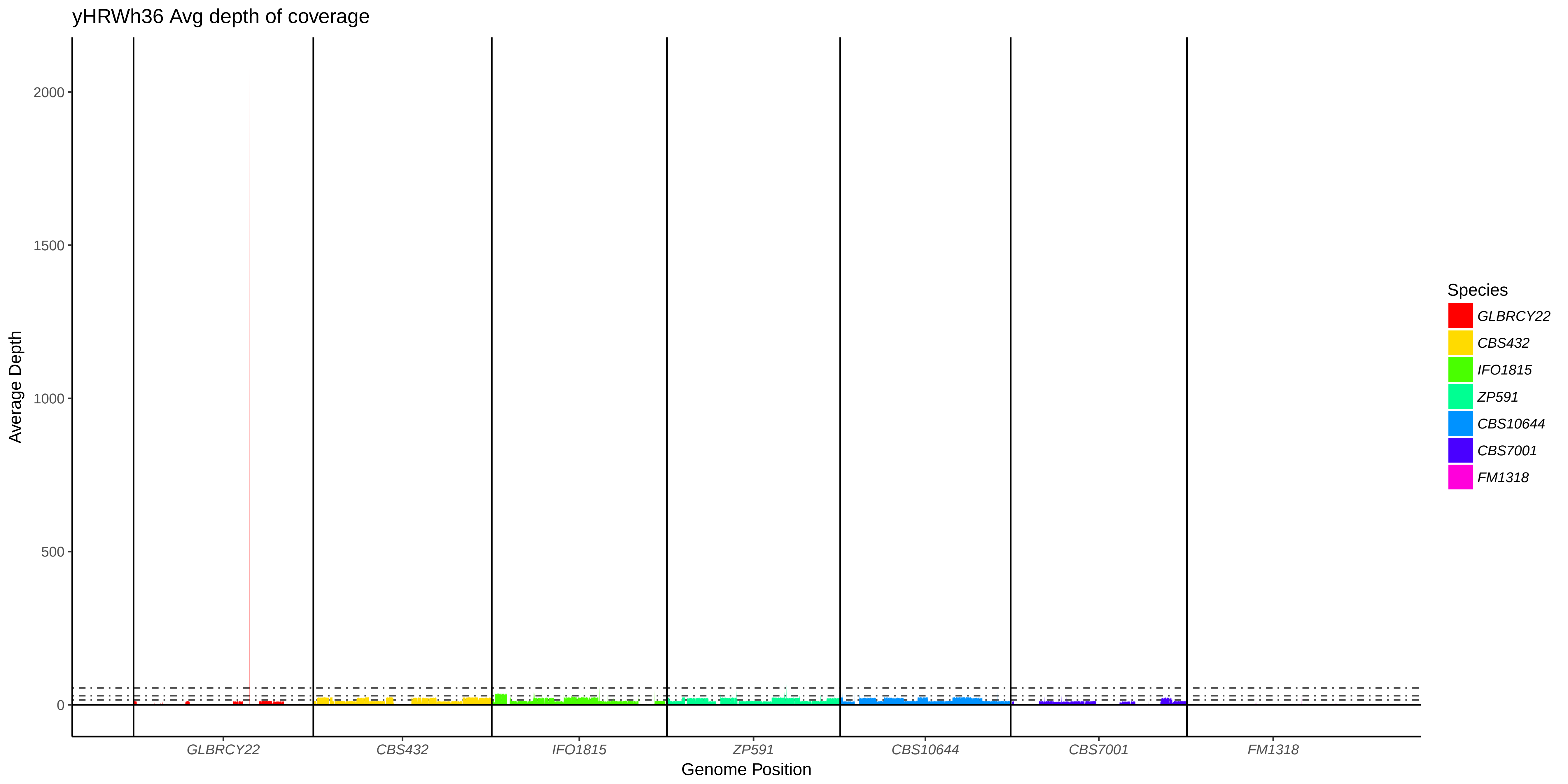

yHRWh36 Avg depth of coverage
Average Depth
2000
1500
1000
500
0
GLBRCY22
CBS432
IFO1815
ZP591
CBS10644
CBS7001
FM1318
Genome Position
Species
GLBRCY22
CBS432
IFO1815
ZP591
CBS10644
CBS7001
FM1318

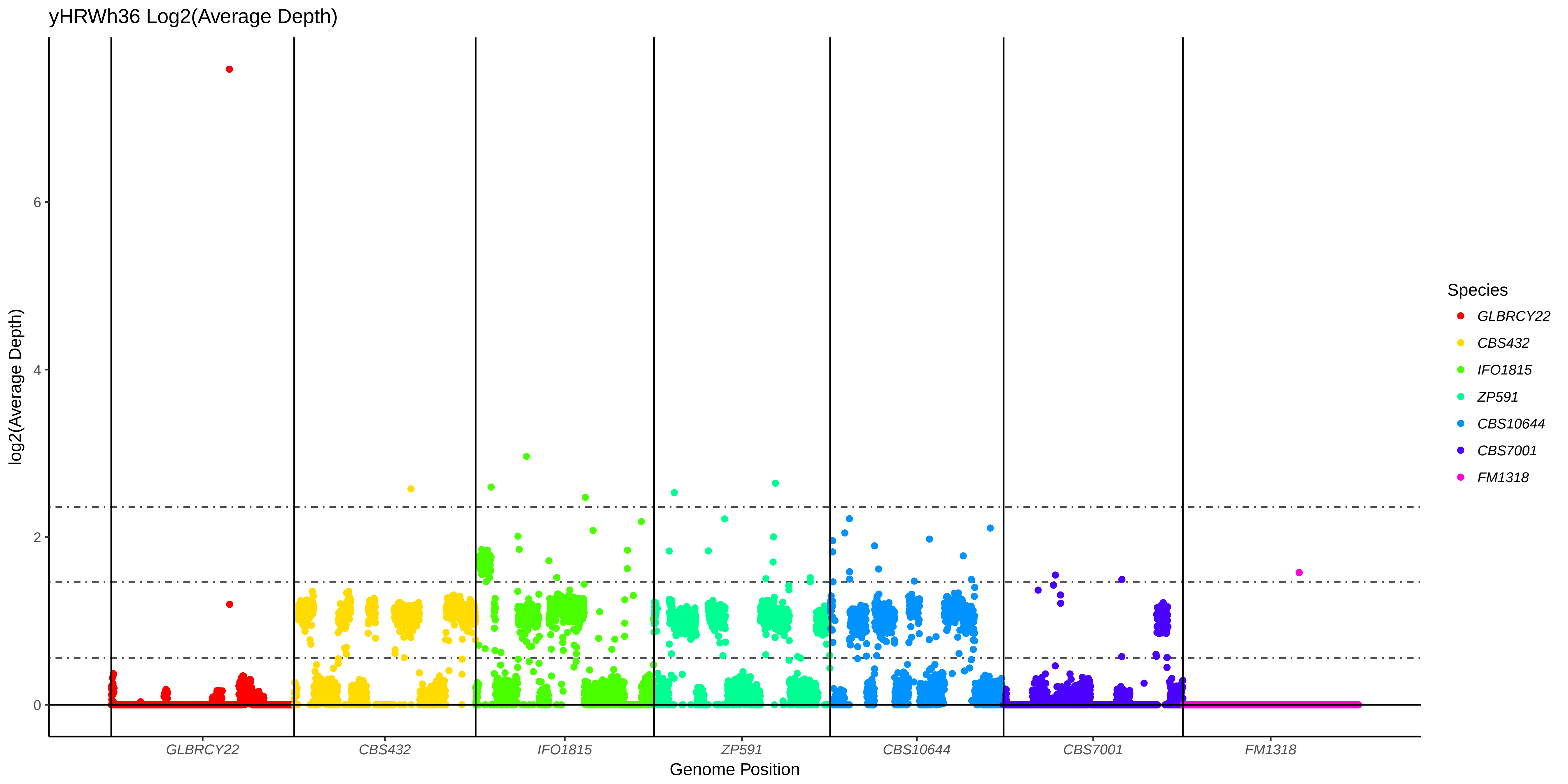
yHRWh36 Log2(Average Depth)
log2(Average Depth)
0
2
4
6
Genome Position
GLBRCY22
CBS432
IFO1815
ZP591
CBS10644
CBS7001
FM1318
Species
GLBRCY22
CBS432
IFO1815
ZP591
CBS10644
CBS7001
FM1318

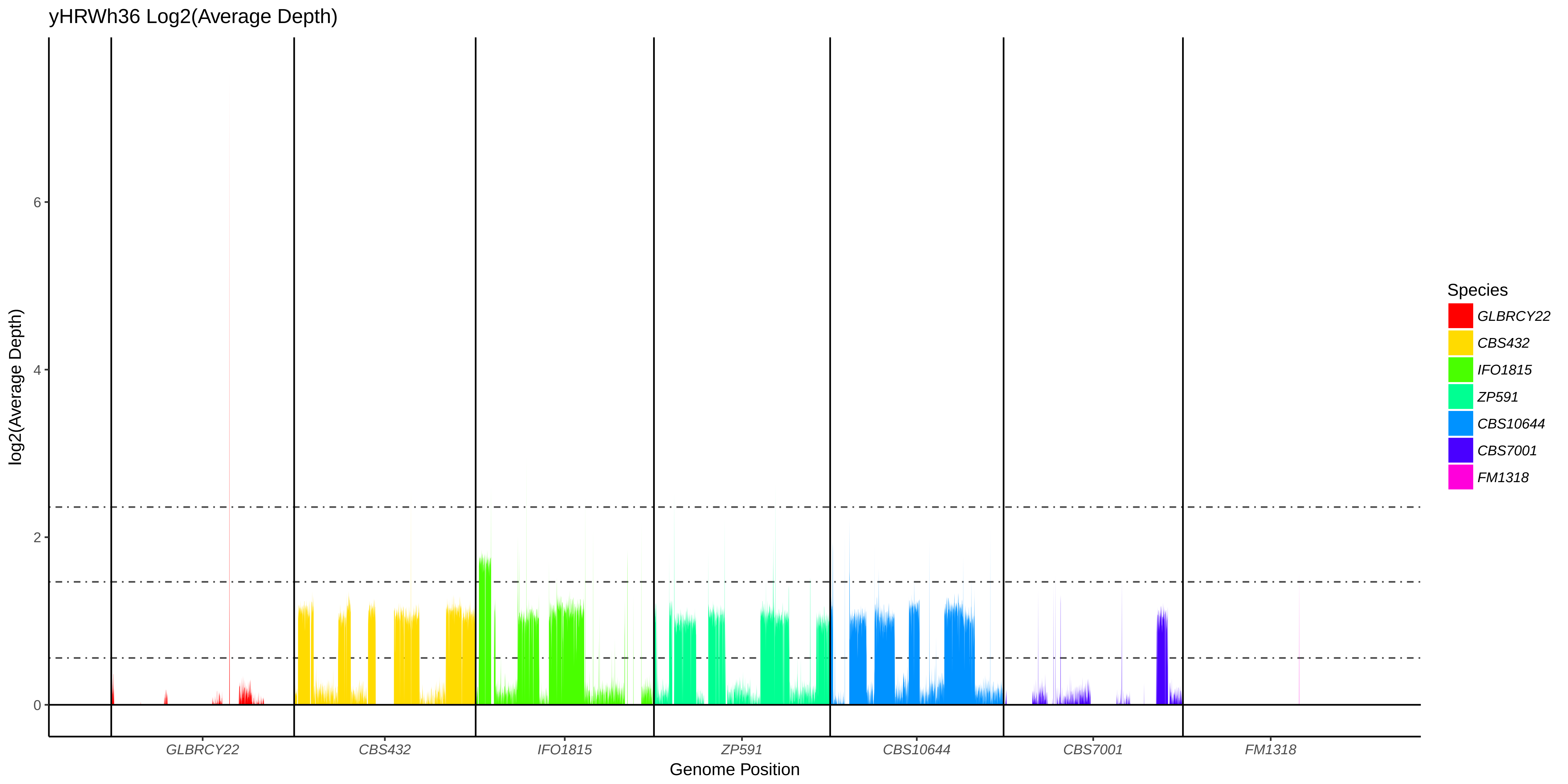

yHRWh36 Log2(Average Depth)
log2(Average Depth)
0
2
4
6
Genome Position
GLBRCY22
CBS432
IFO1815
ZP591
CBS10644
CBS7001
FM1318
Species
GLBRCY22
CBS432
IFO1815
ZP591
CBS10644
CBS7001
FM1318

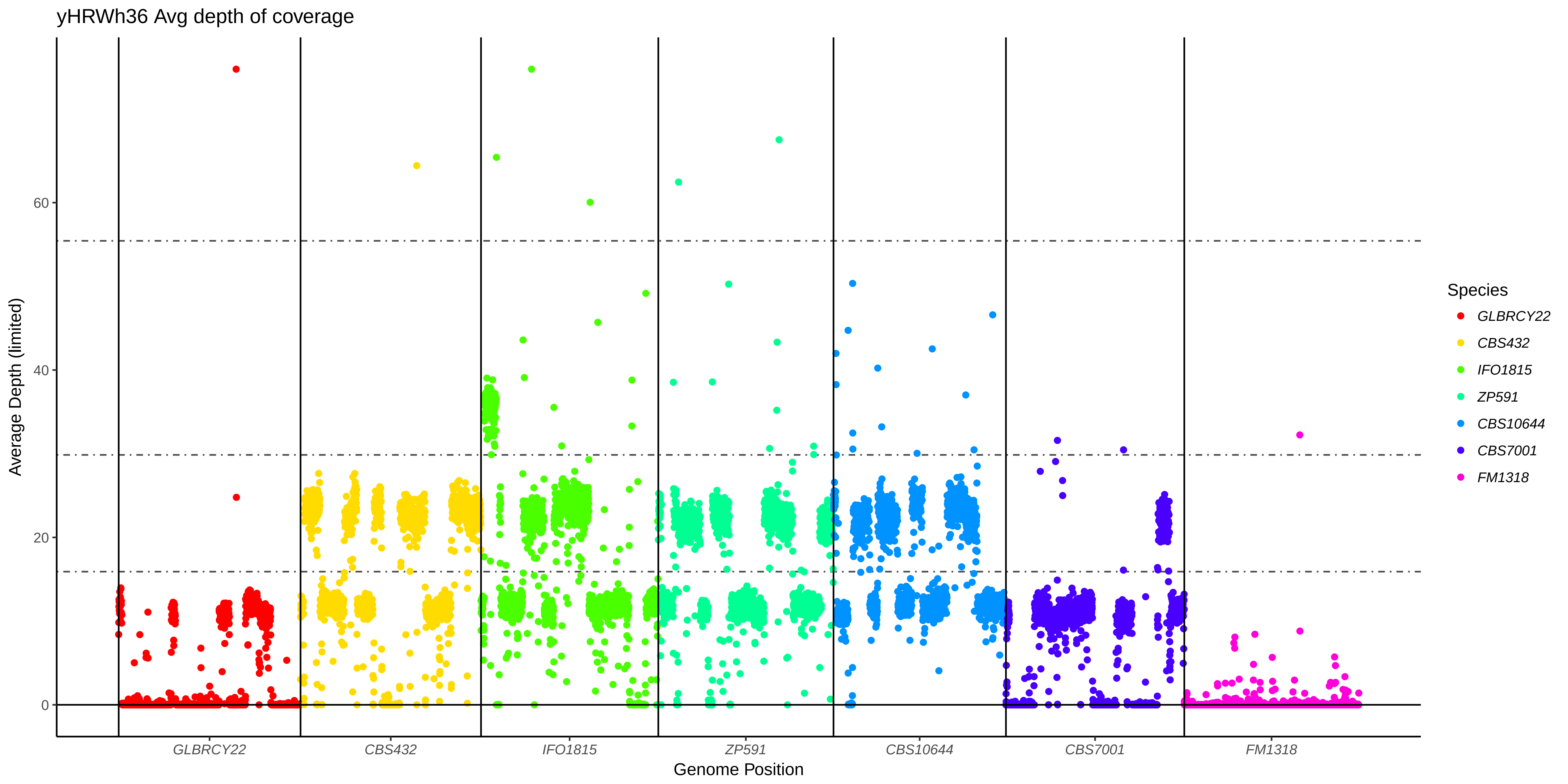

yHRWh36 Avg depth of coverage
Average Depth (limited)
0
20
40
60
GLBRCY22
CBS432
IFO1815
ZP591
CBS10644
CBS7001
FM1318
Genome Position
Species
GLBRCY22
CBS432
IFO1815
ZP591
CBS10644
CBS7001
FM1318

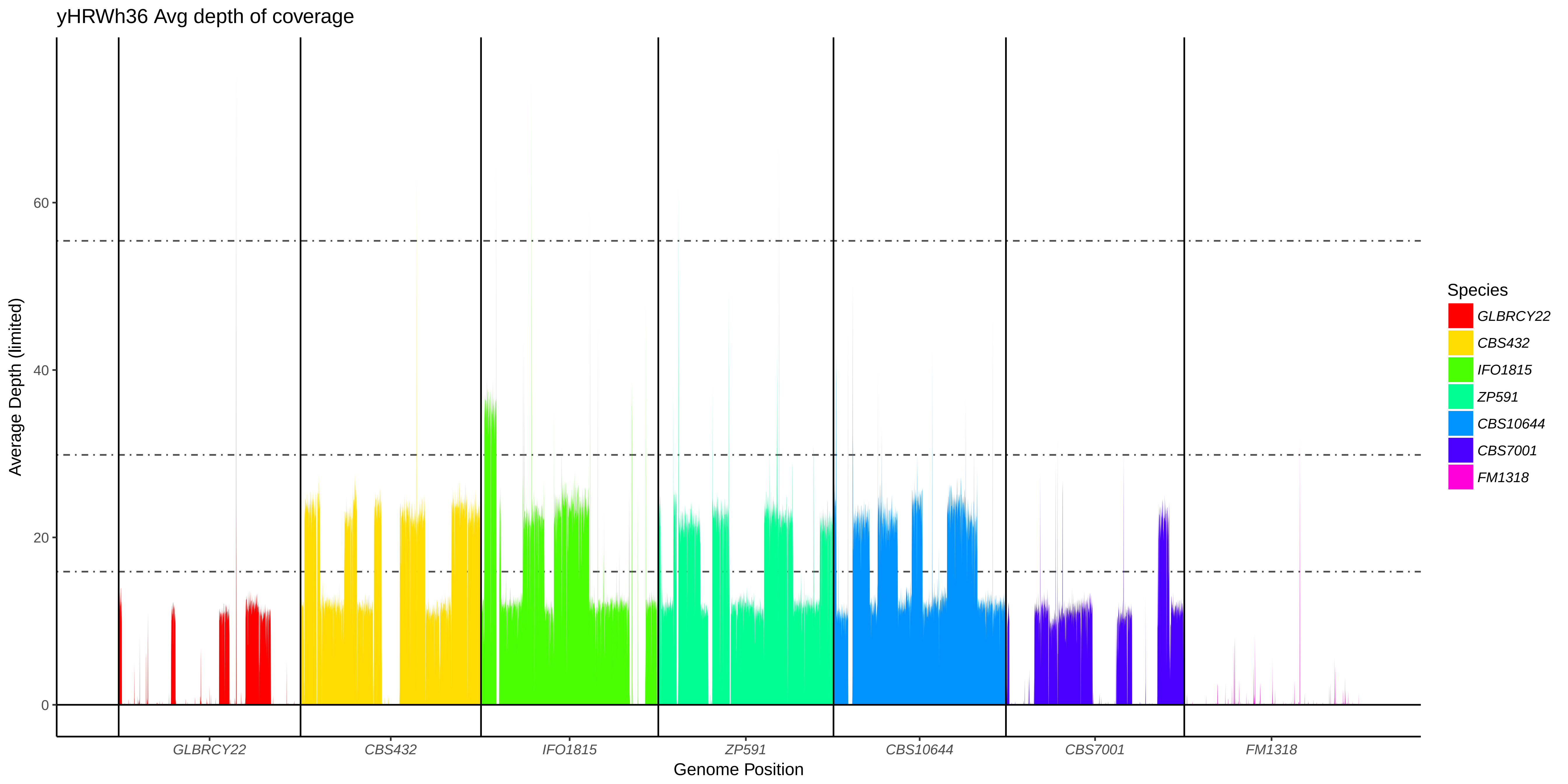
yHRWh36 Avg depth of coverage
Average Depth (limited)
0
20
40
60
GLBRCY22
CBS432
IFO1815
ZP591
CBS10644
CBS7001
FM1318
Genome Position
Species
GLBRCY22
CBS432
IFO1815
ZP591
CBS10644
CBS7001
FM1318

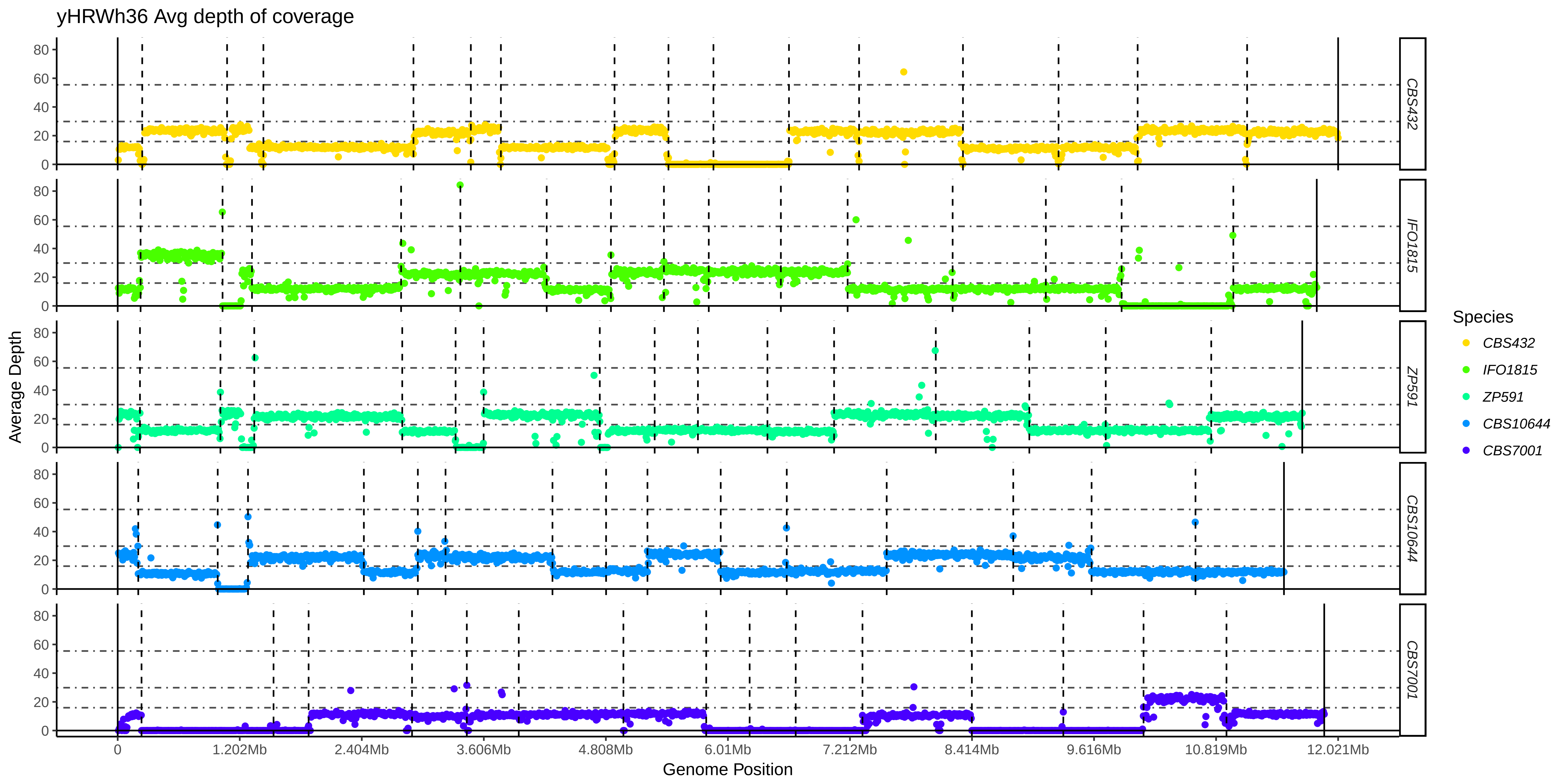
yHRWh36 Avg depth of coverage
Average Depth
Genome Position
0
1.202Mb
2.404Mb
3.606Mb
4.808Mb
6.01Mb
7.212Mb
8.414Mb
9.616Mb
10.819Mb
12.021Mb
0
20
40
60
80
CBS432
IFO1815
ZP591
CBS10644
CBS7001
Species
CBS432
IFO1815
ZP591
CBS10644
CBS7001

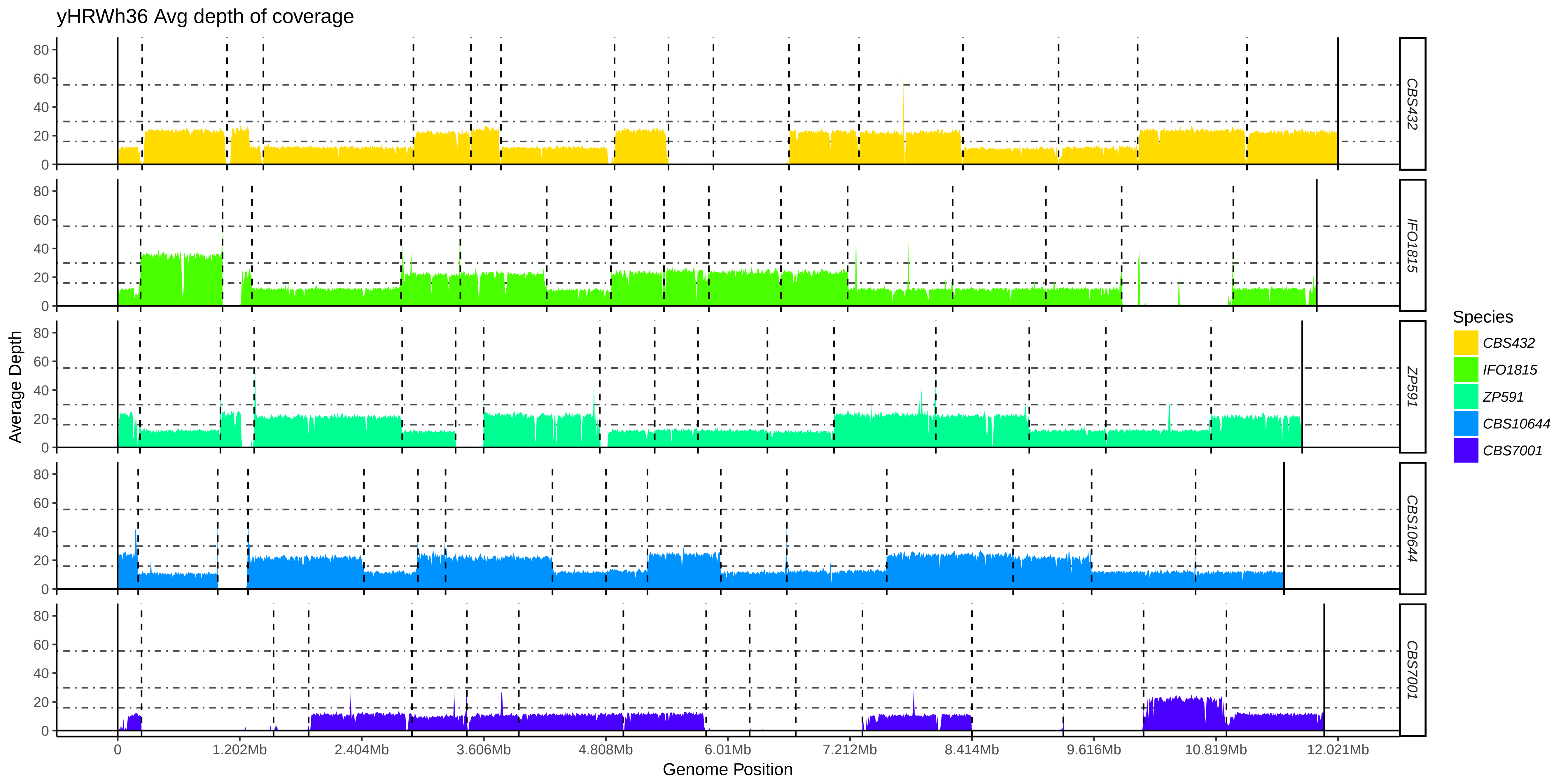
yHRWh36 Avg depth of coverage
Average Depth
Genome Position
0
1.202Mb
2.404Mb
3.606Mb
4.808Mb
6.01Mb
7.212Mb
8.414Mb
9.616Mb
10.819Mb
12.021Mb
0
20
40
60
80
CBS432
IFO1815
ZP591
CBS10644
CBS7001
Species
CBS432
IFO1815
ZP591
CBS10644
CBS7001

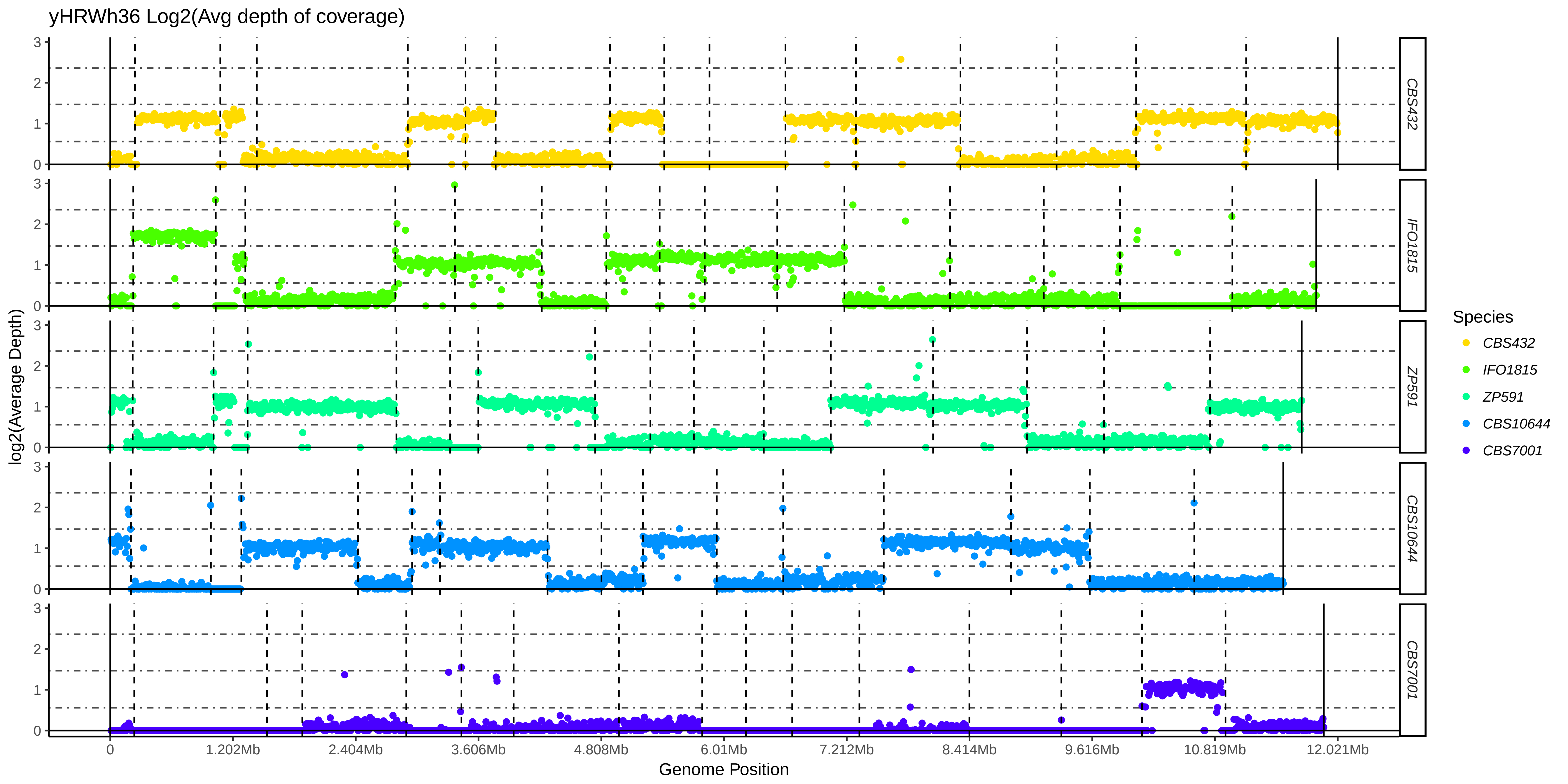
yHRWh36 Log2(Avg depth of coverage)
log2(Average Depth)
Genome Position
0
1.202Mb
2.404Mb
3.606Mb
4.808Mb
6.01Mb
7.212Mb
8.414Mb
9.616Mb
10.819Mb
12.021Mb
CBS432
IFO1815
ZP591
CBS10644
CBS7001
Species
CBS432
IFO1815
ZP591
CBS10644
CBS7001

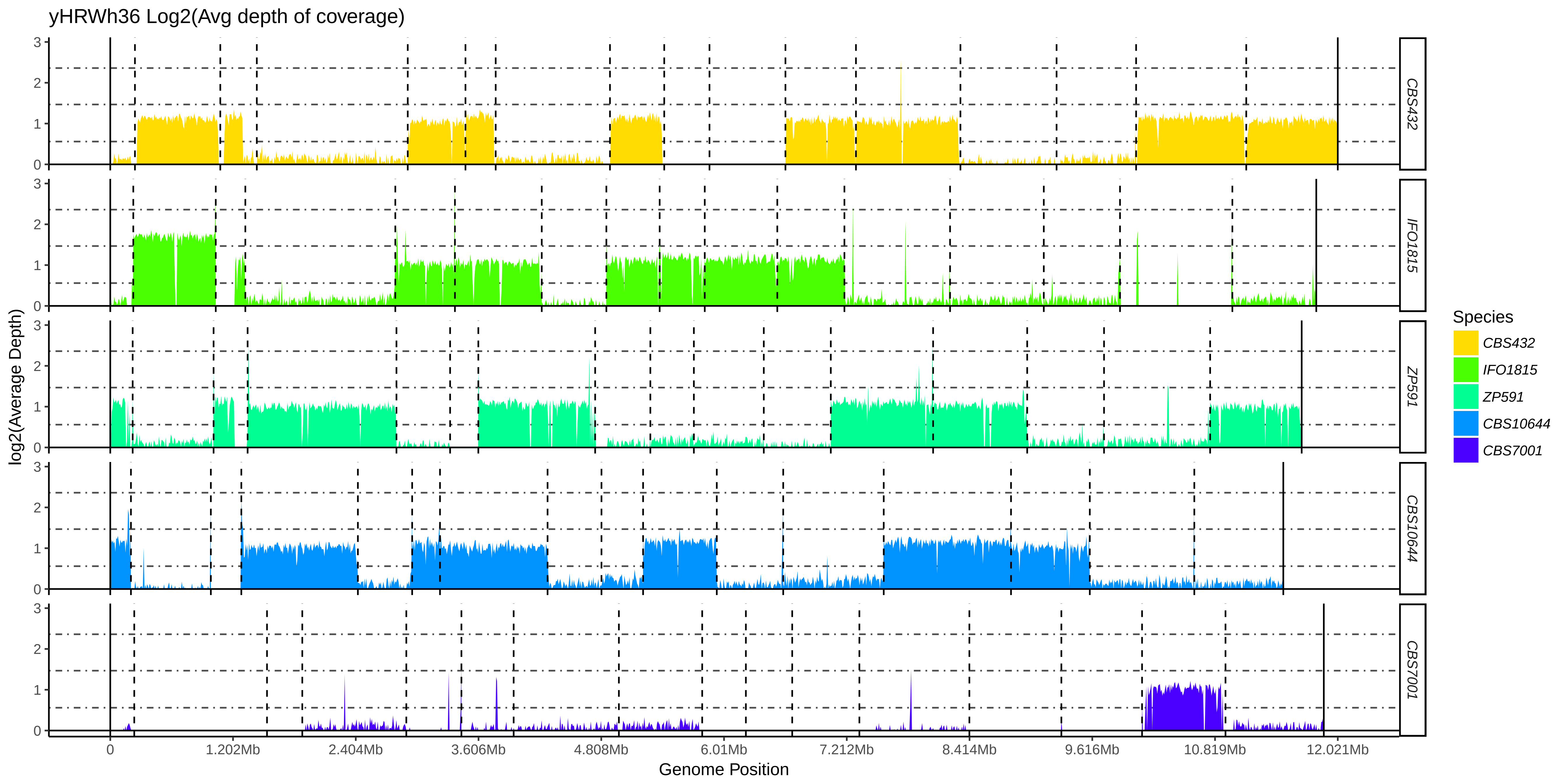

yHRWh36 Log2(Avg depth of coverage)
log2(Average Depth)
Genome Position
0
1.202Mb
2.404Mb
3.606Mb
4.808Mb
6.01Mb
7.212Mb
8.414Mb
9.616Mb
10.819Mb
12.021Mb
CBS432
IFO1815
ZP591
CBS10644
CBS7001
Species

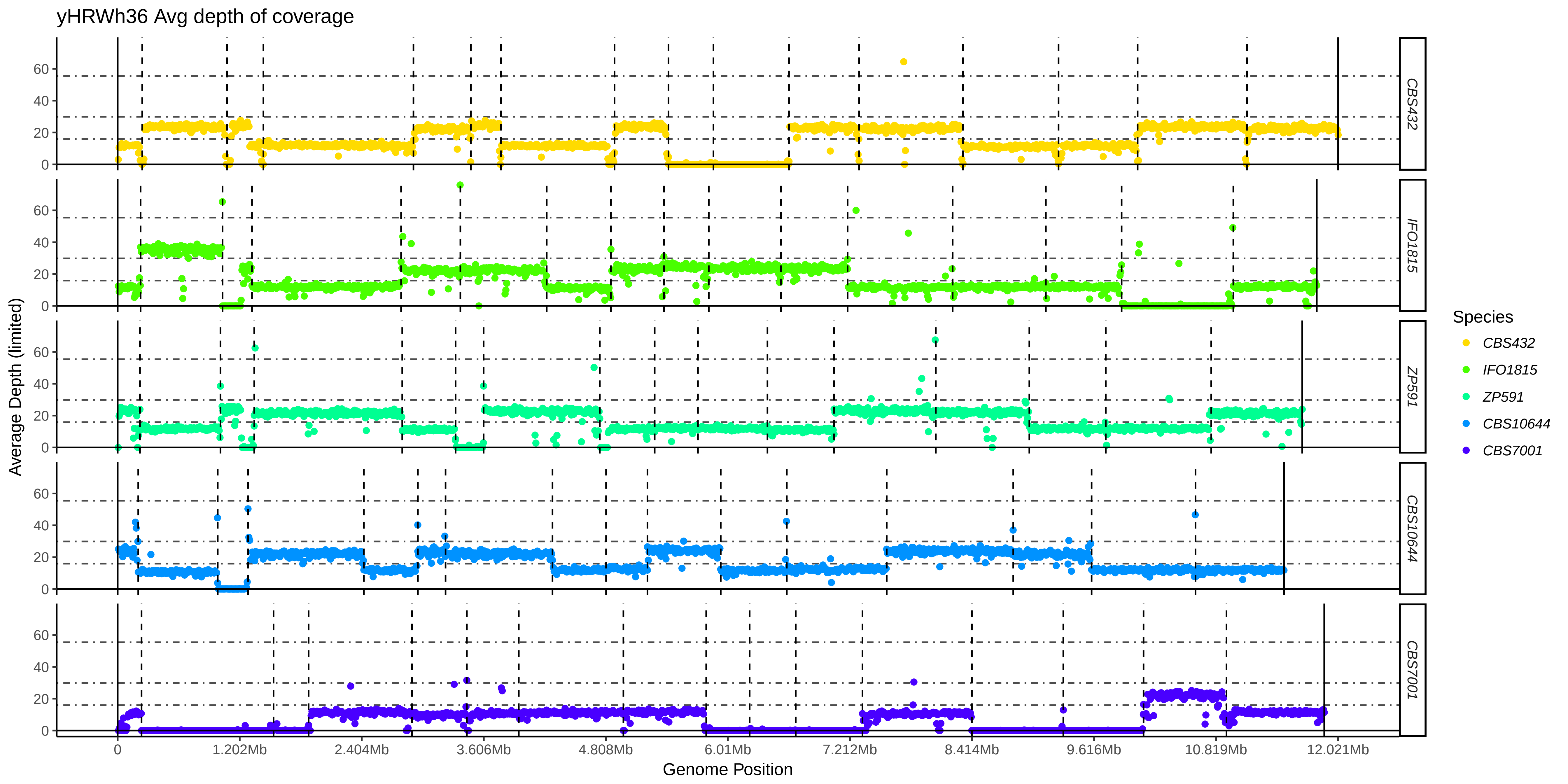

yHRWh36 Avg depth of coverage
Average Depth (limited)
Genome Position
0
1.202Mb
2.404Mb
3.606Mb
4.808Mb
6.01Mb
7.212Mb
8.414Mb
9.616Mb
10.819Mb
12.021Mb
0
20
40
60
CBS432
IFO1815
ZP591
CBS10644
CBS7001
Species
CBS432
IFO1815
ZP591
CBS10644
CBS7001

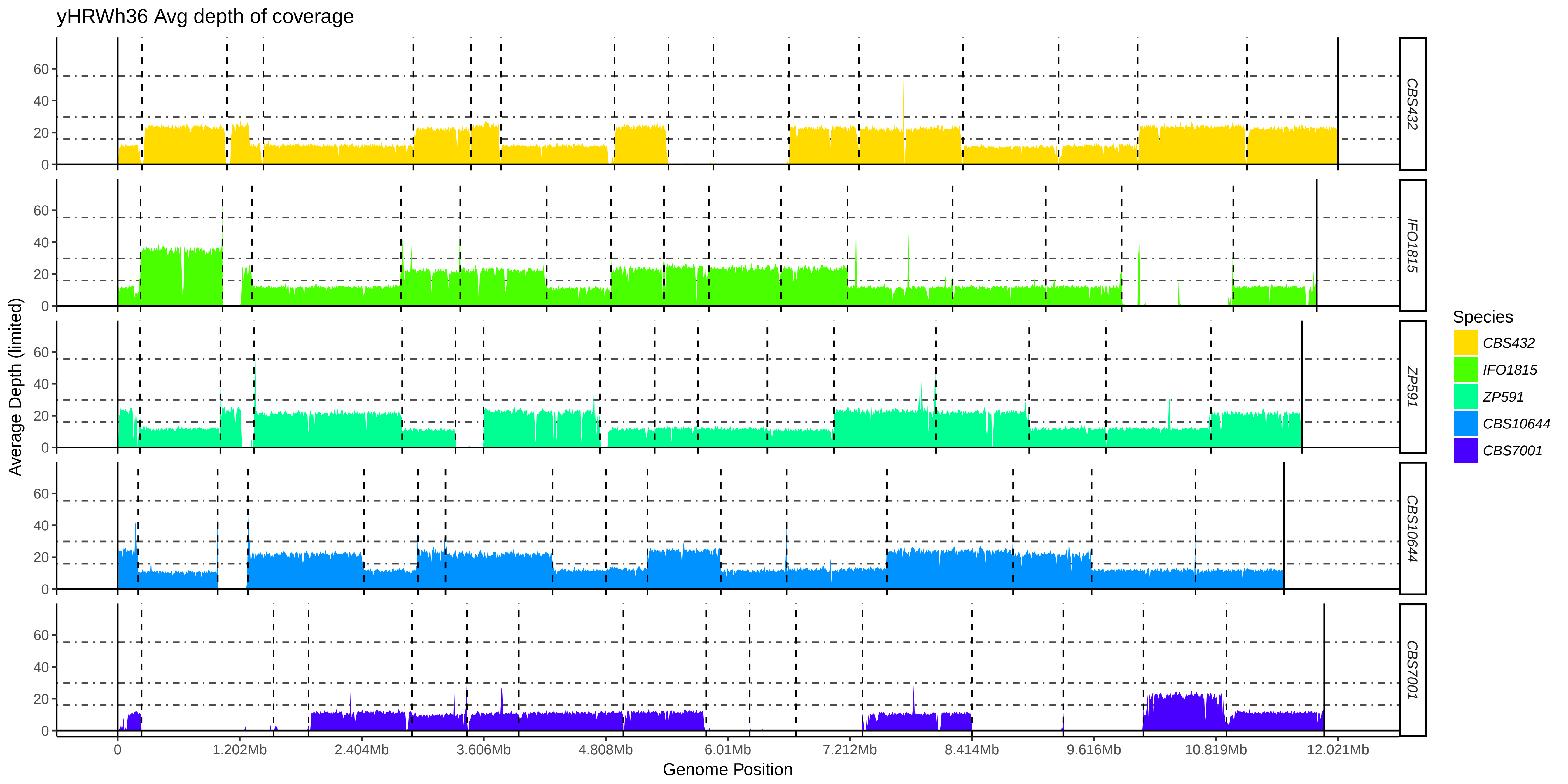

yHRWh36 Avg depth of coverage
Average Depth (limited)
0
20
40
60
CBS432
IFO1815
ZP591
CBS10644
CBS7001
0
1.202Mb
2.404Mb
3.606Mb
4.808Mb
6.01Mb
7.212Mb
8.414Mb
9.616Mb
10.819Mb
12.021Mb
Genome Position
Species
CBS432
IFO1815
ZP591
CBS10644
CBS7001
