# Supplementary material for: Synthetic hybrids of six yeast species
Source: Nat Commun. 2020 Apr 29;11:2085. doi: 10.1038/s41467-020-15559-4 (PMC7190663; doi:10.1038/s41467-020-15559-4)
Supplement: Supplementary file 15 — Source Data [file 41467_2020_15559_MOESM15_ESM.zip › Source Data 2.zip!/yHRWh42_sppIDerDepthPlot-covBins.pdf]

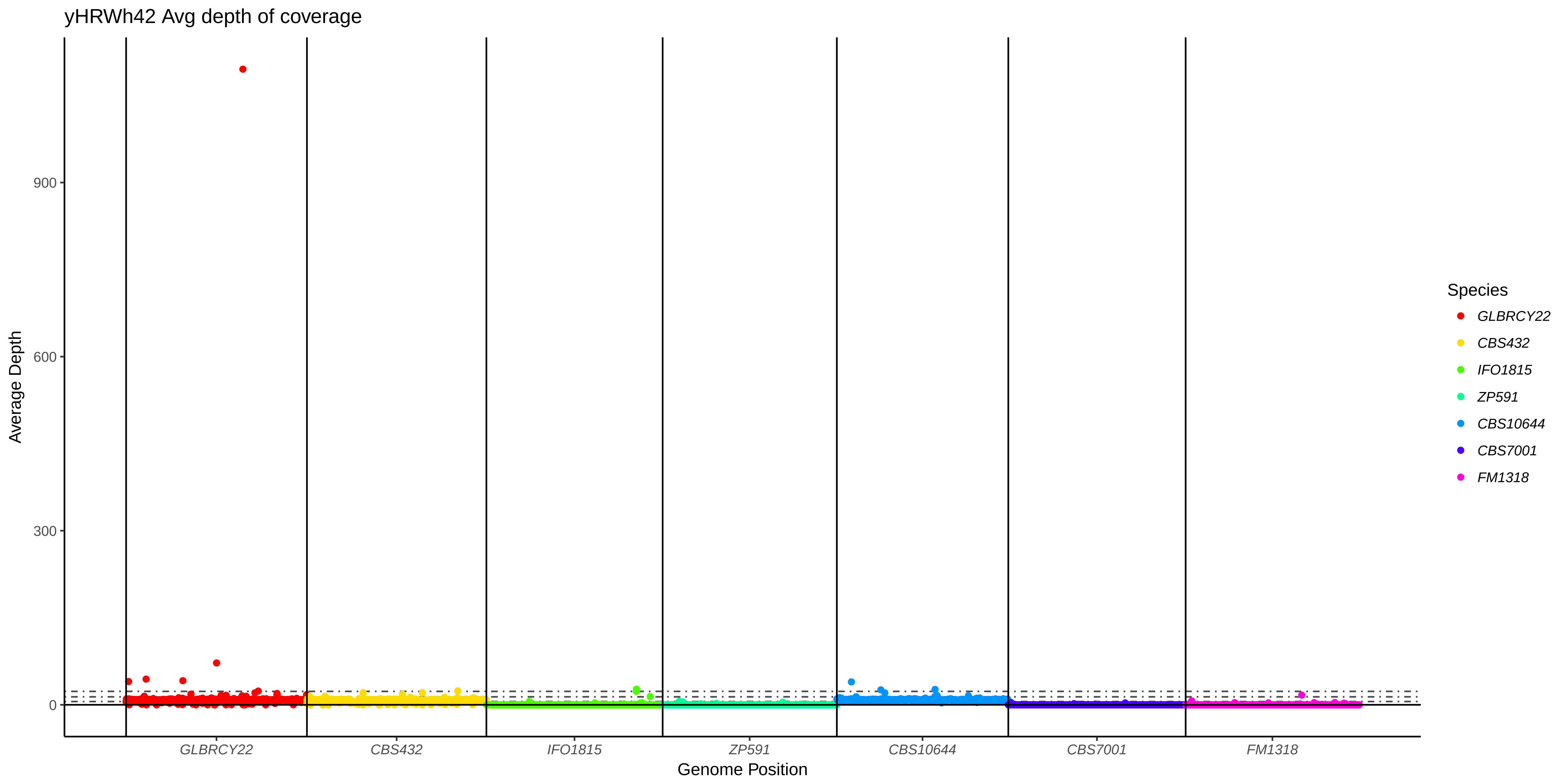
yHRWh42 Avg depth of coverage
Average Depth
900
600
300
0
GLBRCY22
CBS432
IFO1815
ZP591
CBS10644
CBS7001
FM1318
Genome Position
Species
GLBRCY22
CBS432
IFO1815
ZP591
CBS10644
CBS7001
FM1318

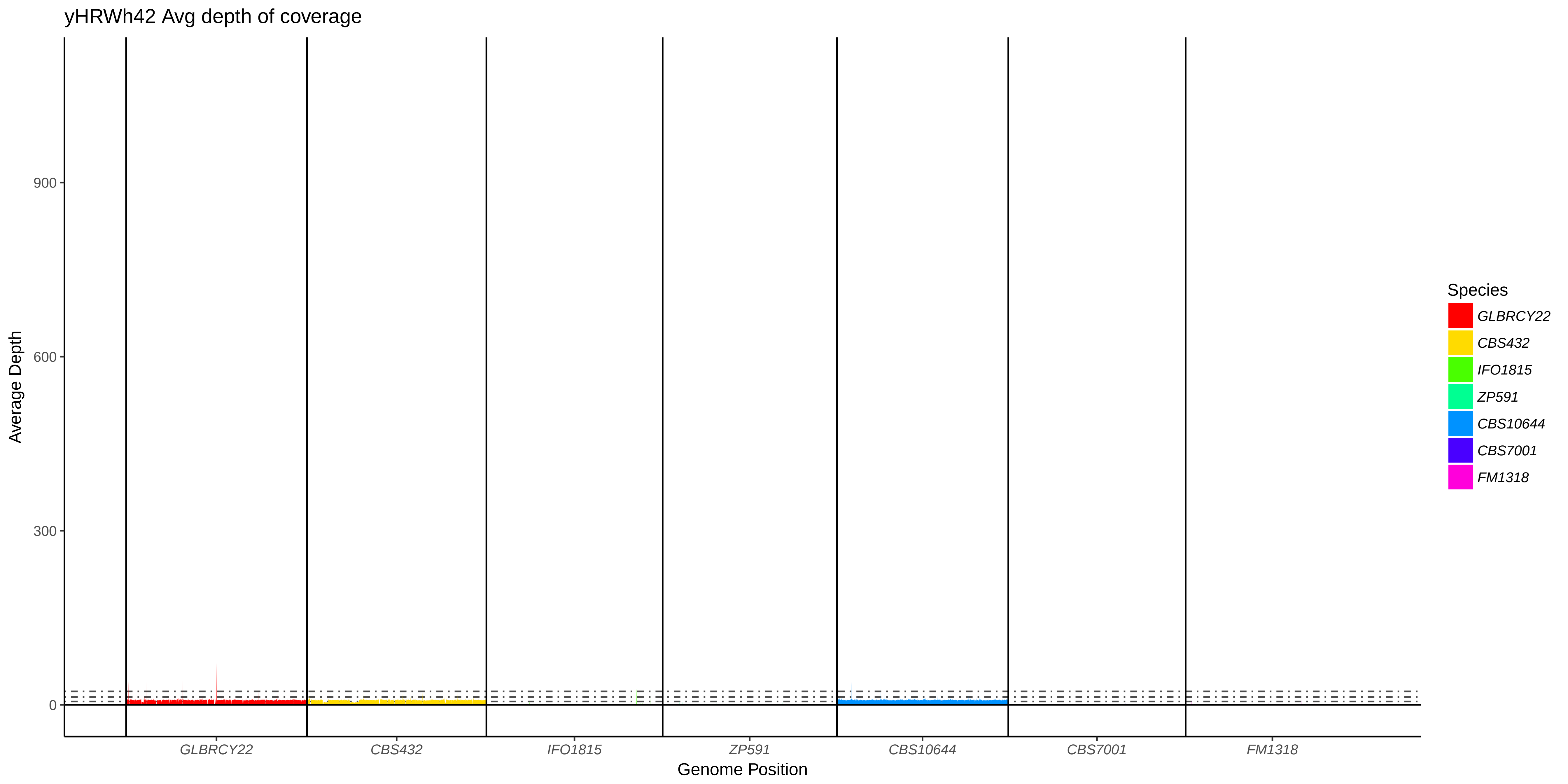
yHRWh42 Avg depth of coverage
Average Depth
900
600
300
0
GLBRCY22
CBS432
IFO1815
ZP591
CBS10644
CBS7001
FM1318
Genome Position
Species
GLBRCY22
CBS432
IFO1815
ZP591
CBS10644
CBS7001
FM1318

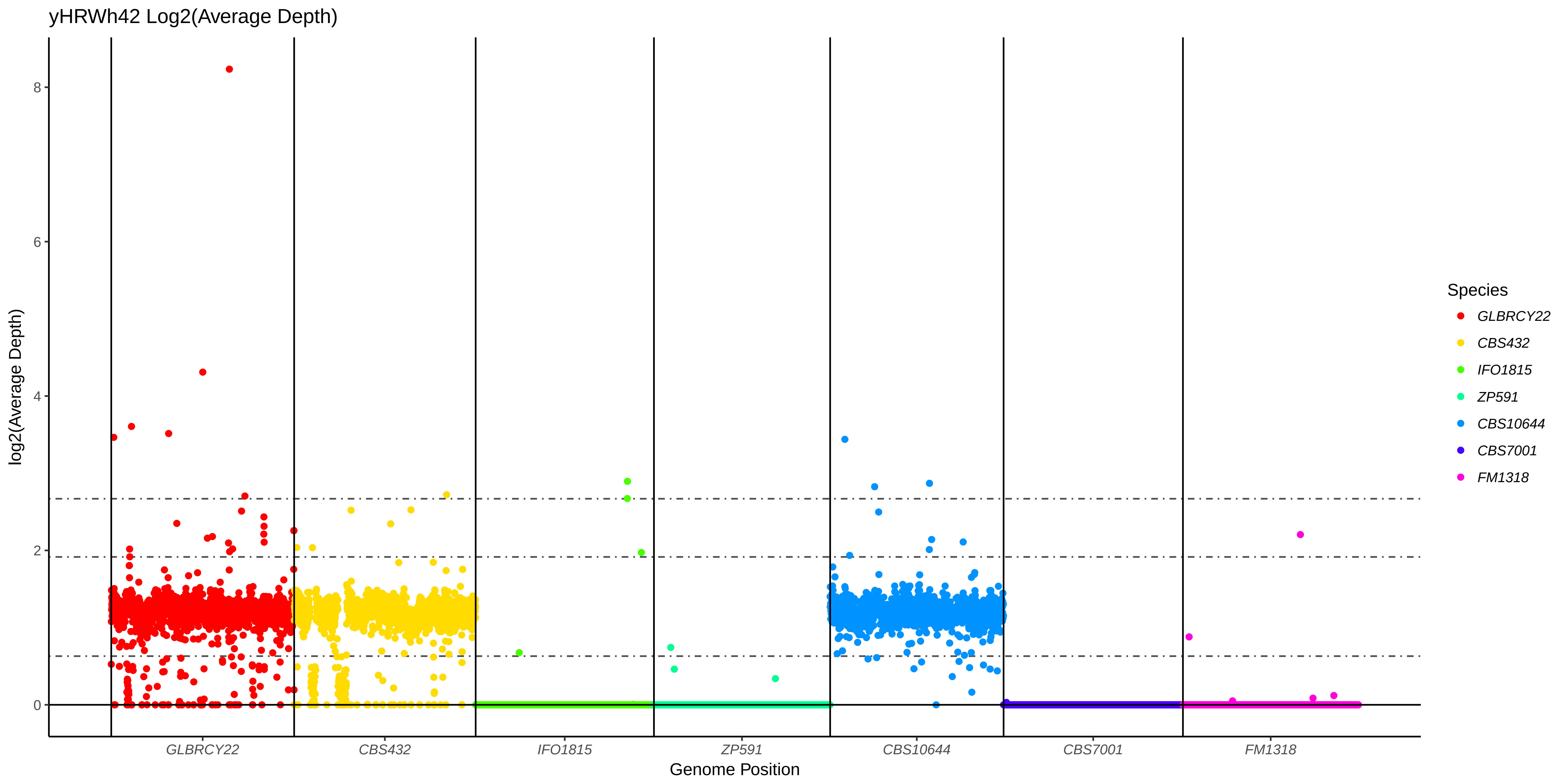

yHRWh42 Log2(Average Depth)
log2(Average Depth)
8
6
4
2
0
GLBRCY22
CBS432
IFO1815
ZP591
CBS10644
CBS7001
FM1318
Genome Position
Species
GLBRCY22
CBS432
IFO1815
ZP591
CBS10644
CBS7001
FM1318

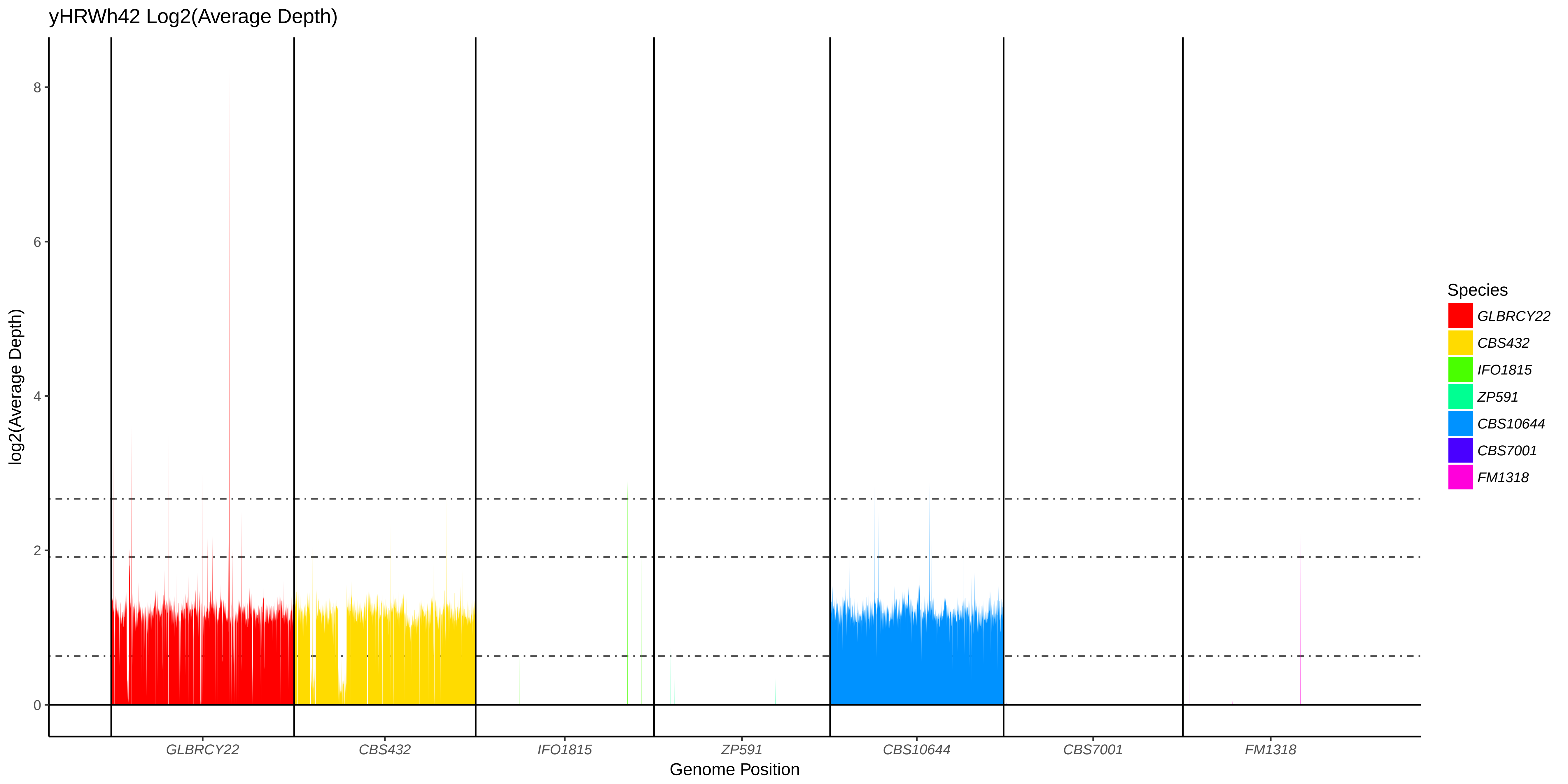

yHRWh42 Log2(Average Depth)
log2(Average Depth)
8
6
4
2
0
GLBRCY22
CBS432
IFO1815
ZP591
CBS10644
CBS7001
FM1318
Genome Position
Species
GLBRCY22
CBS432
IFO1815
ZP591
CBS10644
CBS7001
FM1318

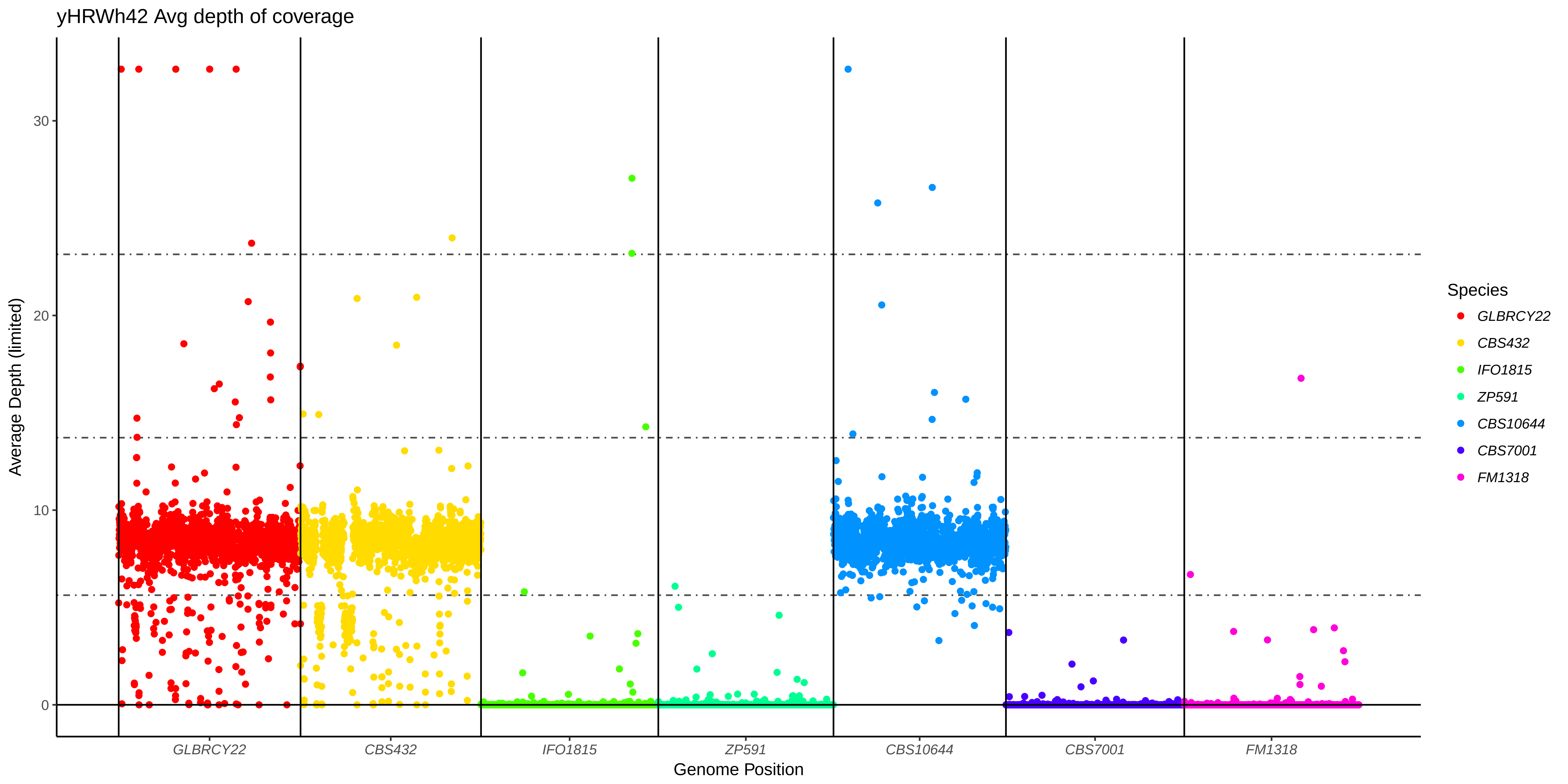

yHRWh42 Avg depth of coverage
Average Depth (limited)
30
20
10
0
GLBRCY22
CBS432
IFO1815
ZP591
CBS10644
CBS7001
FM1318
Genome Position
Species
GLBRCY22
CBS432
IFO1815
ZP591
CBS10644
CBS7001
FM1318

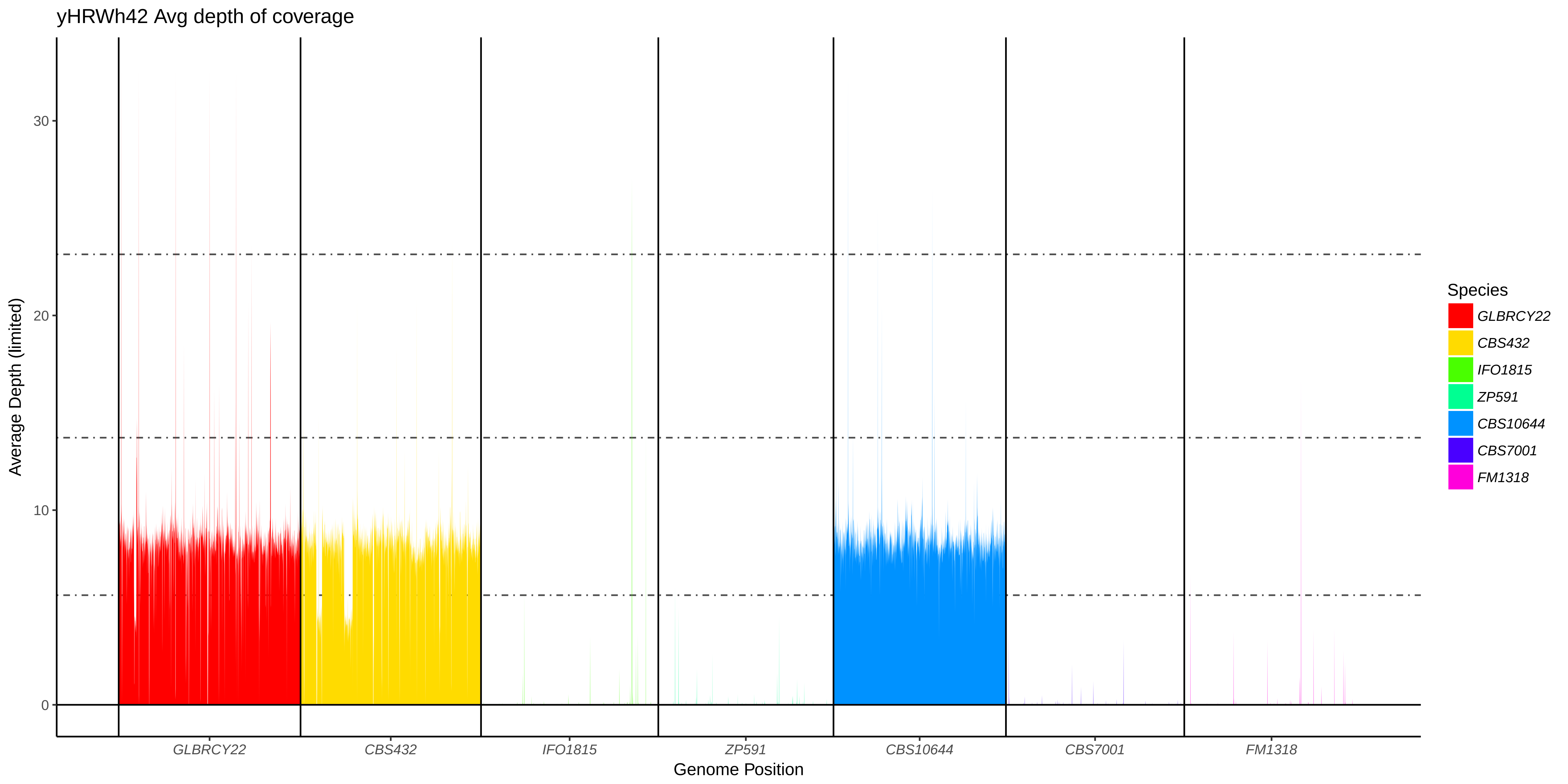

yHRWh42 Avg depth of coverage
Average Depth (limited)
0
10
20
30
GLBRCY22
CBS432
IFO1815
ZP591
CBS10644
CBS7001
FM1318
Genome Position
Species
GLBRCY22
CBS432
IFO1815
ZP591
CBS10644
CBS7001
FM1318

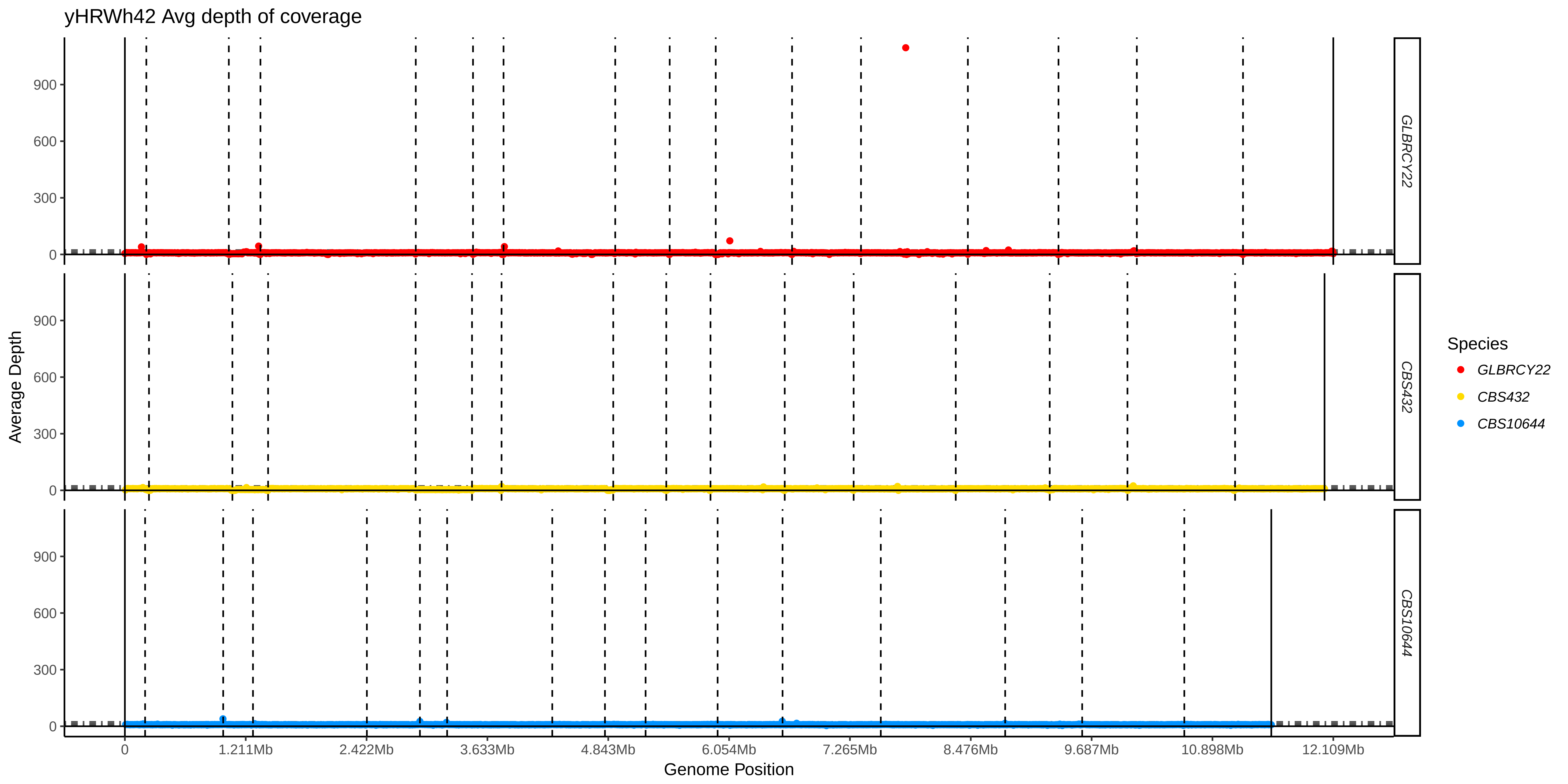

yHRWh42 Avg depth of coverage
Average Depth
Genome Position
0
300
600
900
0
1.211Mb
2.422Mb
3.633Mb
4.843Mb
6.054Mb
7.265Mb
8.476Mb
9.687Mb
10.898Mb
12.109Mb
GLBRCY22
CBS432
CBS10644
Species
GLBRCY22
CBS432
CBS10644

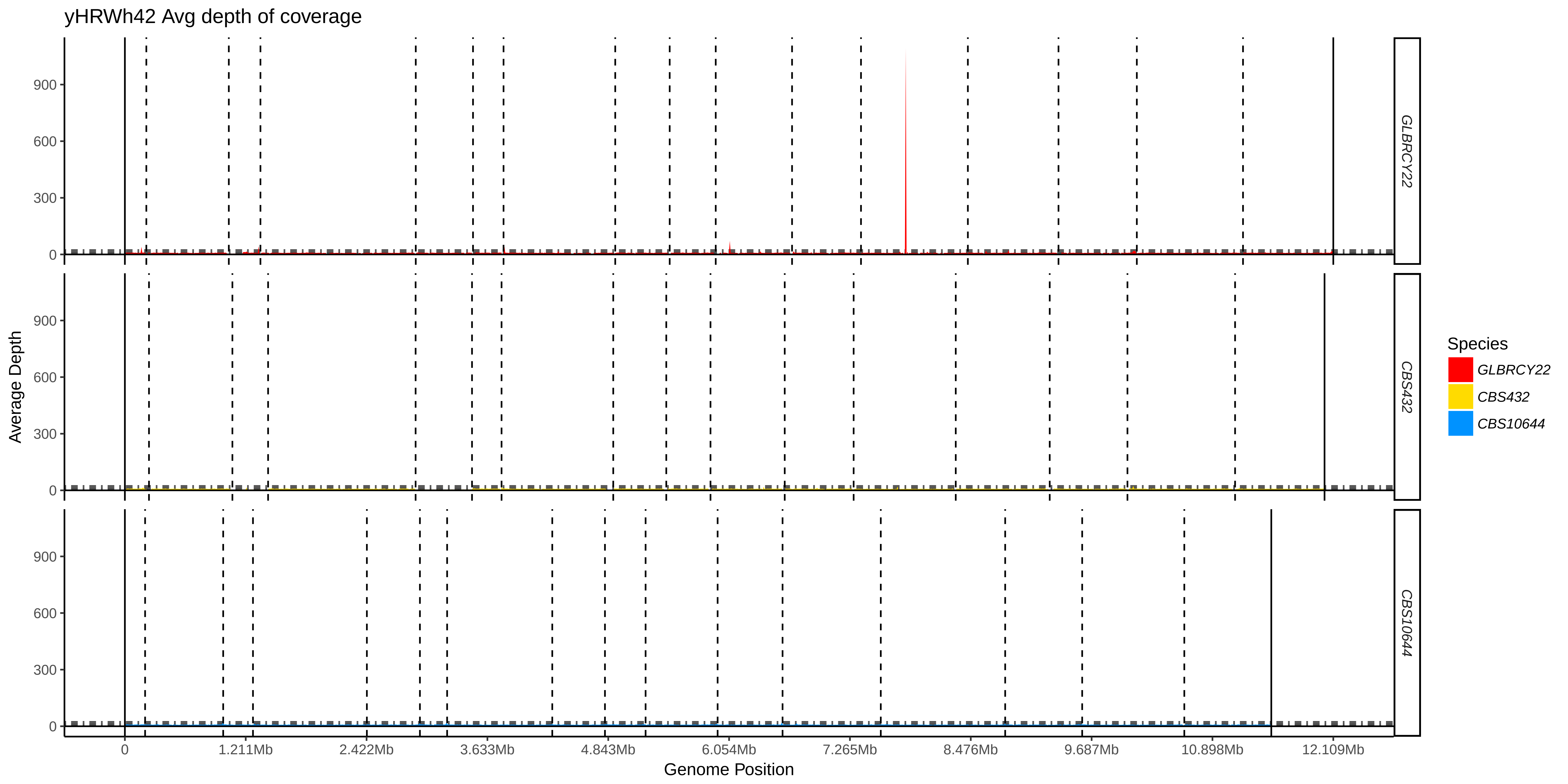
yHRWh42 Avg depth of coverage
Average Depth
900
600
300
0
GLBRCY22
CBS432
CBS10644
0
1.211Mb
2.422Mb
3.633Mb
4.843Mb
6.054Mb
7.265Mb
8.476Mb
9.687Mb
10.898Mb
12.109Mb
Genome Position
Species
GLBRCY22
CBS432
CBS10644

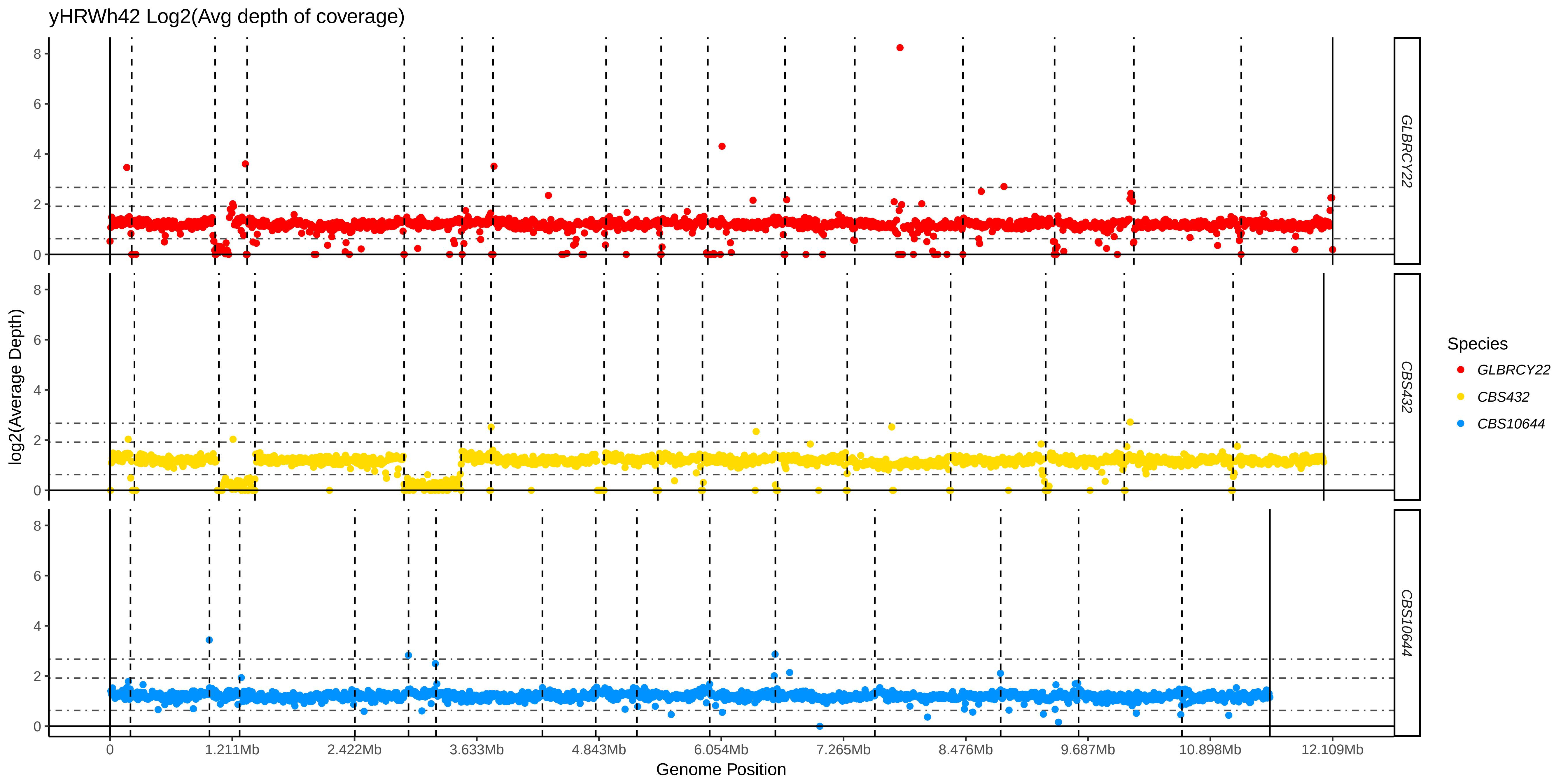
yHRWh42 Log2(Avg depth of coverage)
log2(Average Depth)
Genome Position
0
1.211Mb
2.422Mb
3.633Mb
4.843Mb
6.054Mb
7.265Mb
8.476Mb
9.687Mb
10.898Mb
12.109Mb
GLBRCY22
CBS432
CBS10644
Species

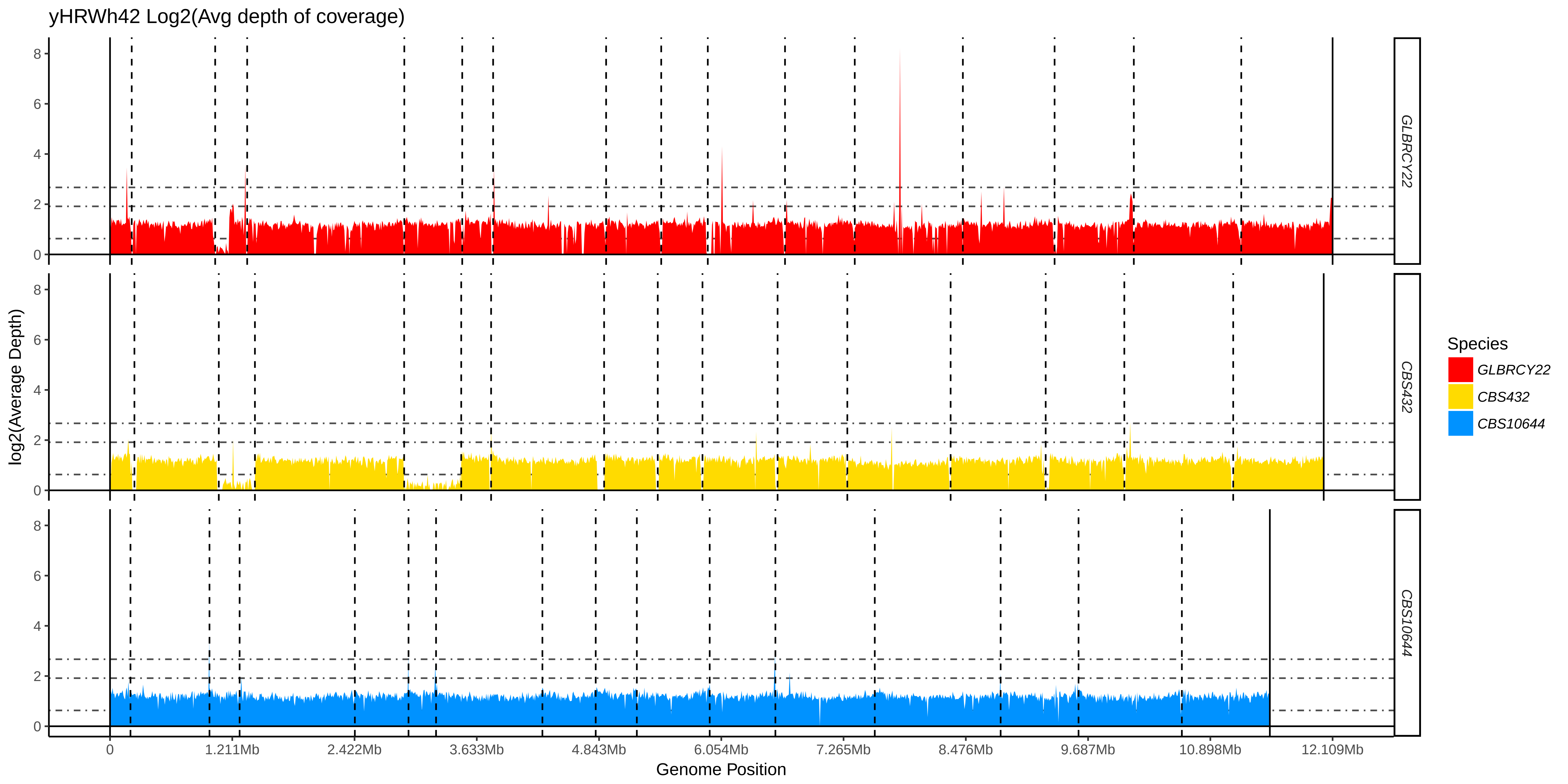

yHRWh42 Log2(Avg depth of coverage)
log2(Average Depth)
Genome Position
0
1.211Mb
2.422Mb
3.633Mb
4.843Mb
6.054Mb
7.265Mb
8.476Mb
9.687Mb
10.898Mb
12.109Mb
GLBRCY22
CBS432
CBS10644
Species

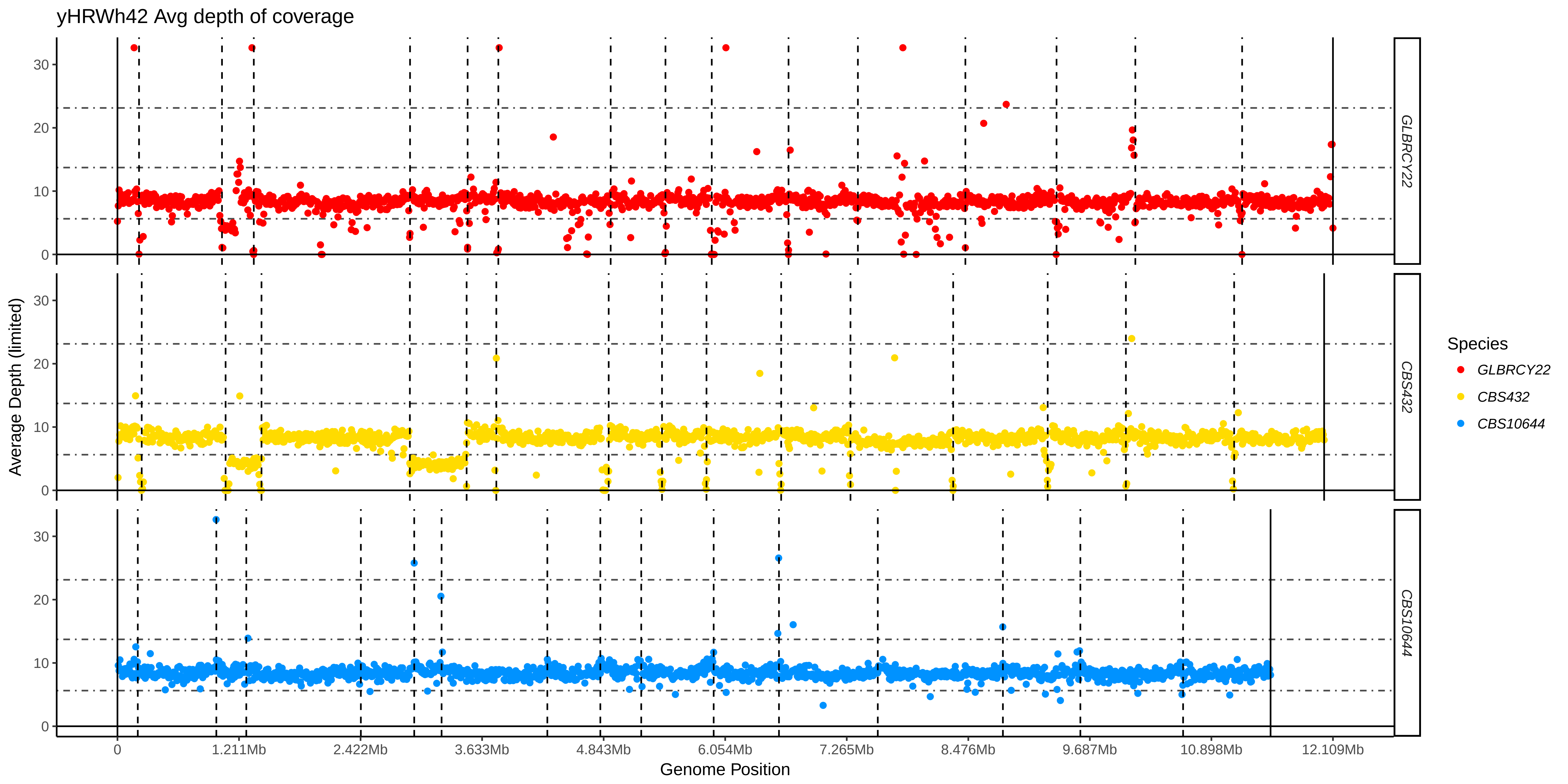

yHRWh42 Avg depth of coverage
Average Depth (limited)
Genome Position
0
1.211Mb
2.422Mb
3.633Mb
4.843Mb
6.054Mb
7.265Mb
8.476Mb
9.687Mb
10.898Mb
12.109Mb
0
10
20
30
GLBRCY22
CBS432
CBS10644
Species
GLBRCY22
CBS432
CBS10644

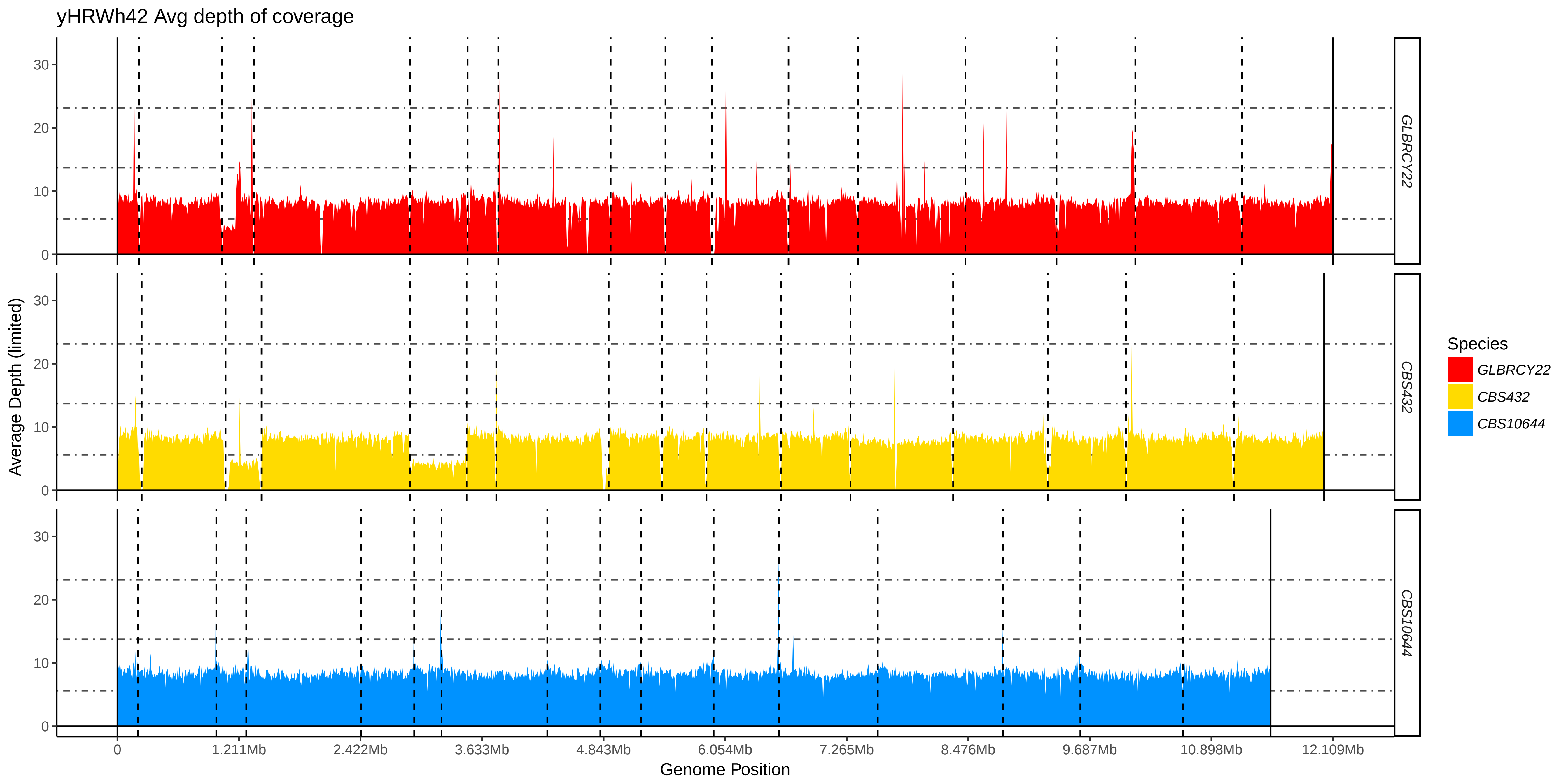
yHRWh42 Avg depth of coverage
Average Depth (limited)
Genome Position
0
10
20
30
0
1.211Mb
2.422Mb
3.633Mb
4.843Mb
6.054Mb
7.265Mb
8.476Mb
9.687Mb
10.898Mb
12.109Mb
GLBRCY22
CBS432
CBS10644
Species
GLBRCY22
CBS432
CBS10644
