# Supplementary material for: Synthetic hybrids of six yeast species
Source: Nat Commun. 2020 Apr 29;11:2085. doi: 10.1038/s41467-020-15559-4 (PMC7190663; doi:10.1038/s41467-020-15559-4)
Supplement: Supplementary file 15 — Source Data [file 41467_2020_15559_MOESM15_ESM.zip › Source Data 2.zip!/yHRWh51_sppIDerDepthPlot-covBins.pdf]

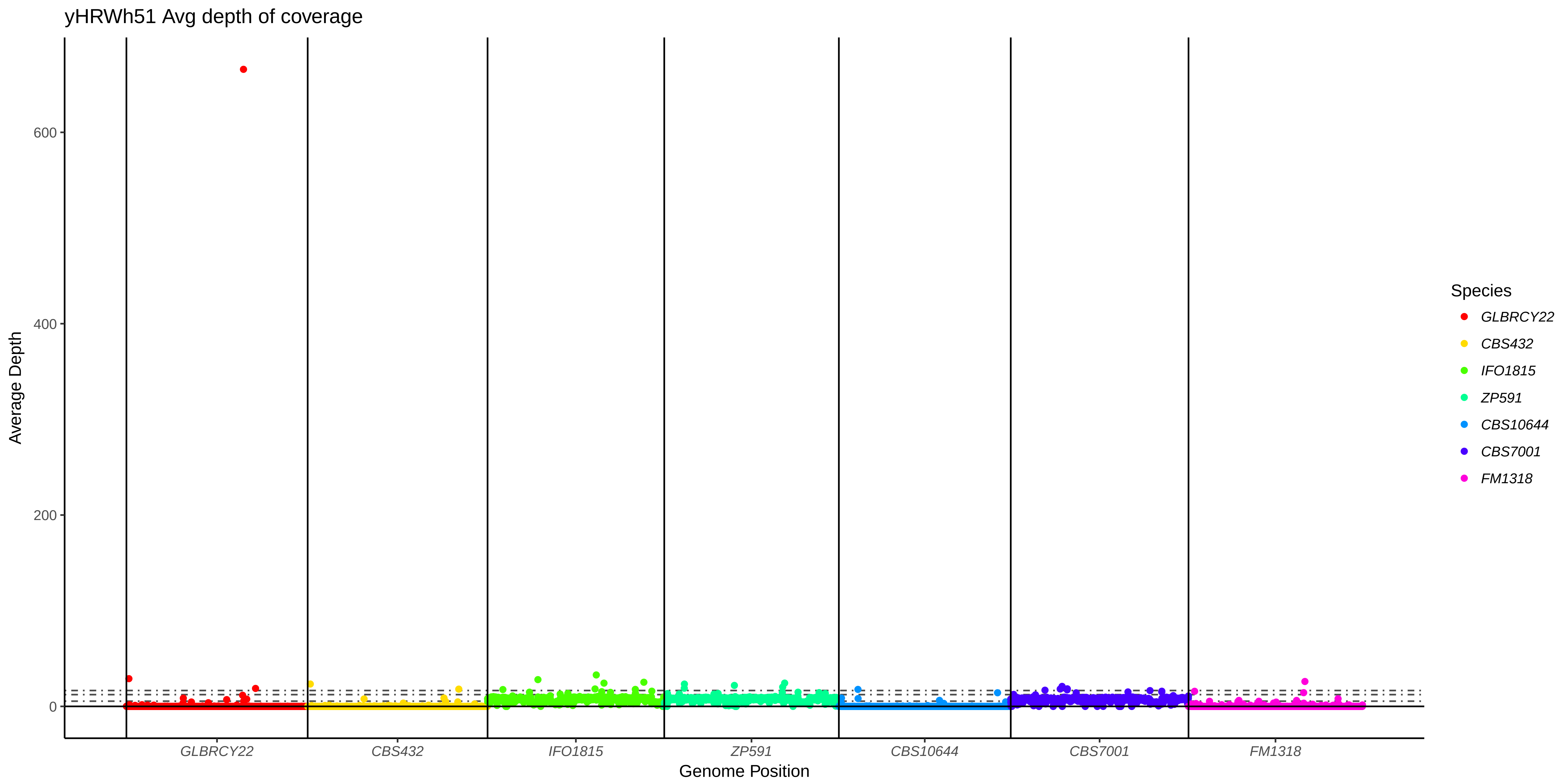

yHRWh51 Avg depth of coverage
Average Depth
600
400
200
0
GLBRCY22
CBS432
IFO1815
ZP591
CBS10644
CBS7001
FM1318
Genome Position
Species
GLBRCY22
CBS432
IFO1815
ZP591
CBS10644
CBS7001
FM1318

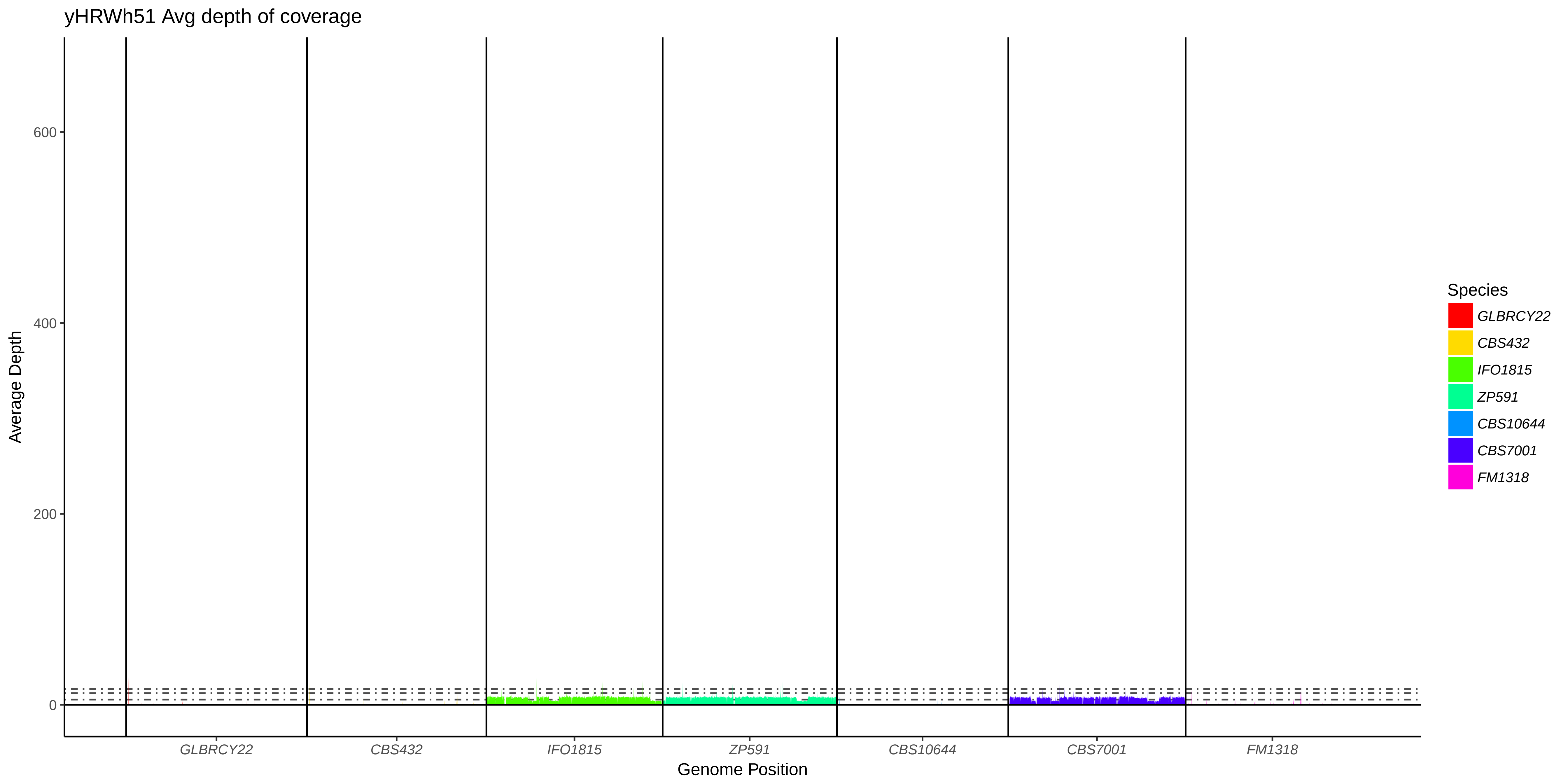
yHRWh51 Avg depth of coverage
Average Depth
600
400
200
0
GLBRCY22
CBS432
IFO1815
ZP591
CBS10644
CBS7001
FM1318
Genome Position
Species
GLBRCY22
CBS432
IFO1815
ZP591
CBS10644
CBS7001
FM1318

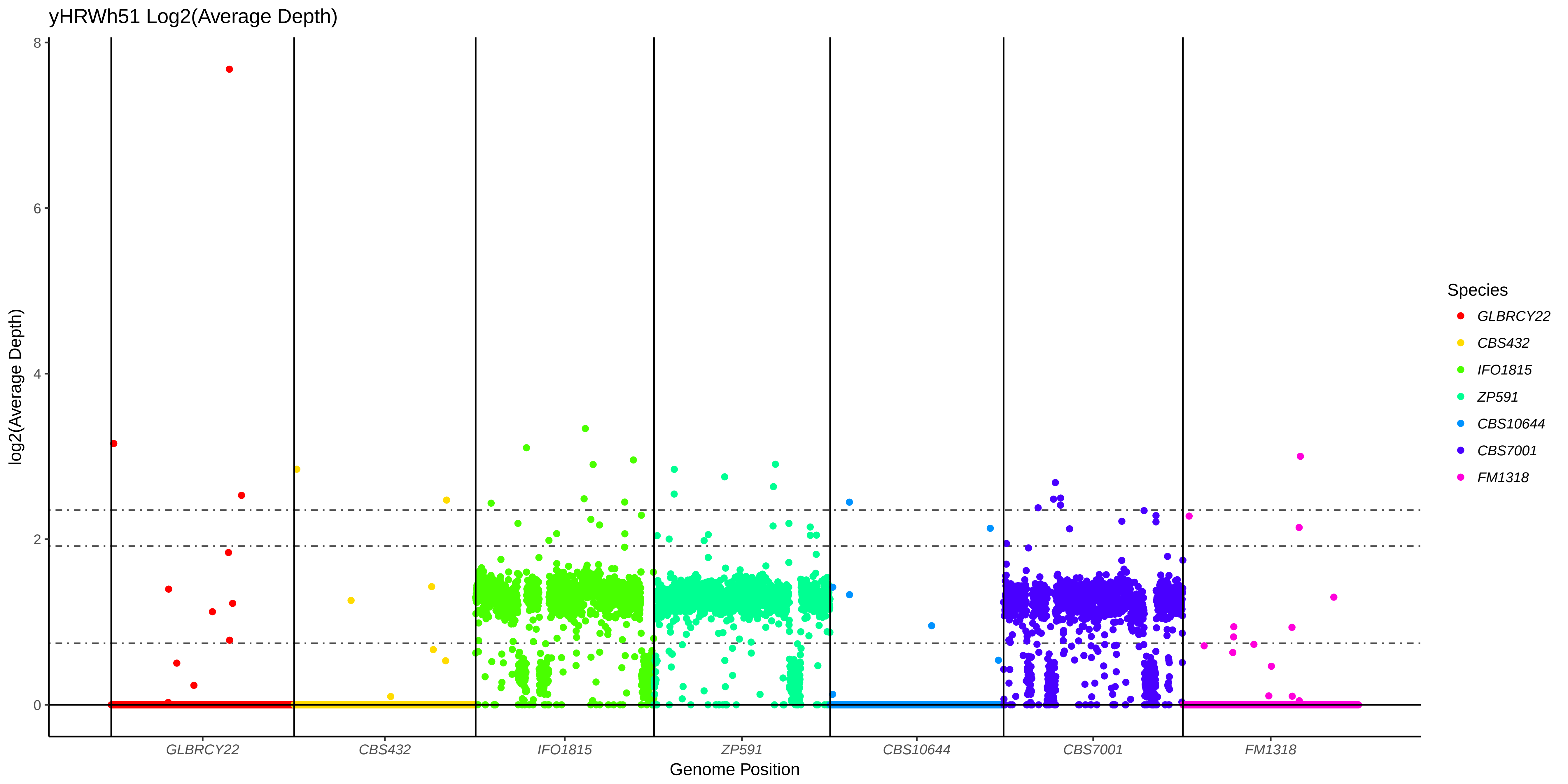

yHRWh51 Log2(Average Depth)
log2(Average Depth)
8
6
4
2
0
Genome Position
GLBRCY22
CBS432
IFO1815
ZP591
CBS10644
CBS7001
FM1318
Species
GLBRCY22
CBS432
IFO1815
ZP591
CBS10644
CBS7001
FM1318

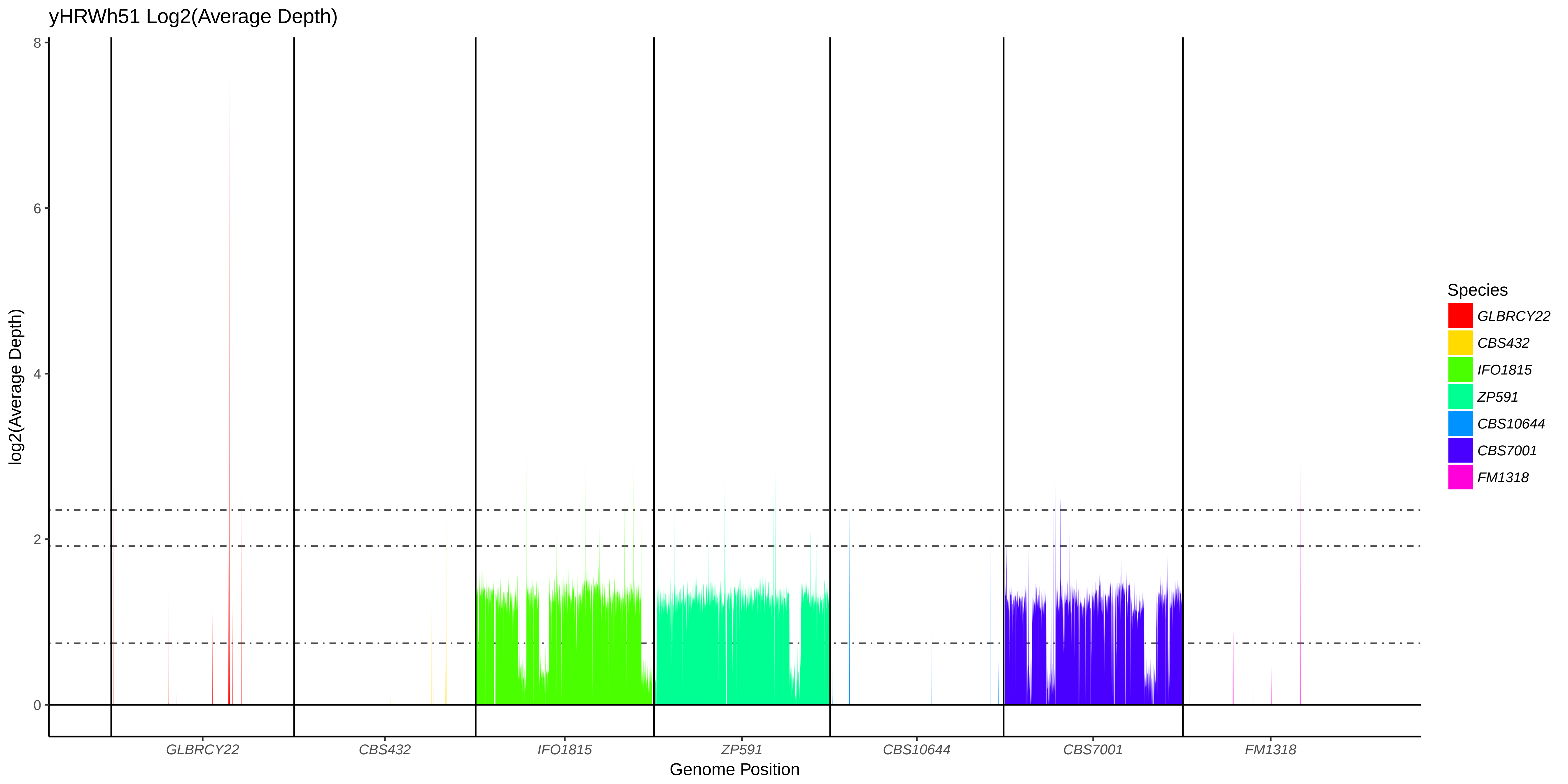

yHRWh51 Log2(Average Depth)
log2(Average Depth)
0
2
4
6
8
GLBRCY22
CBS432
IFO1815
ZP591
CBS10644
CBS7001
FM1318
Genome Position
Species
GLBRCY22
CBS432
IFO1815
ZP591
CBS10644
CBS7001
FM1318

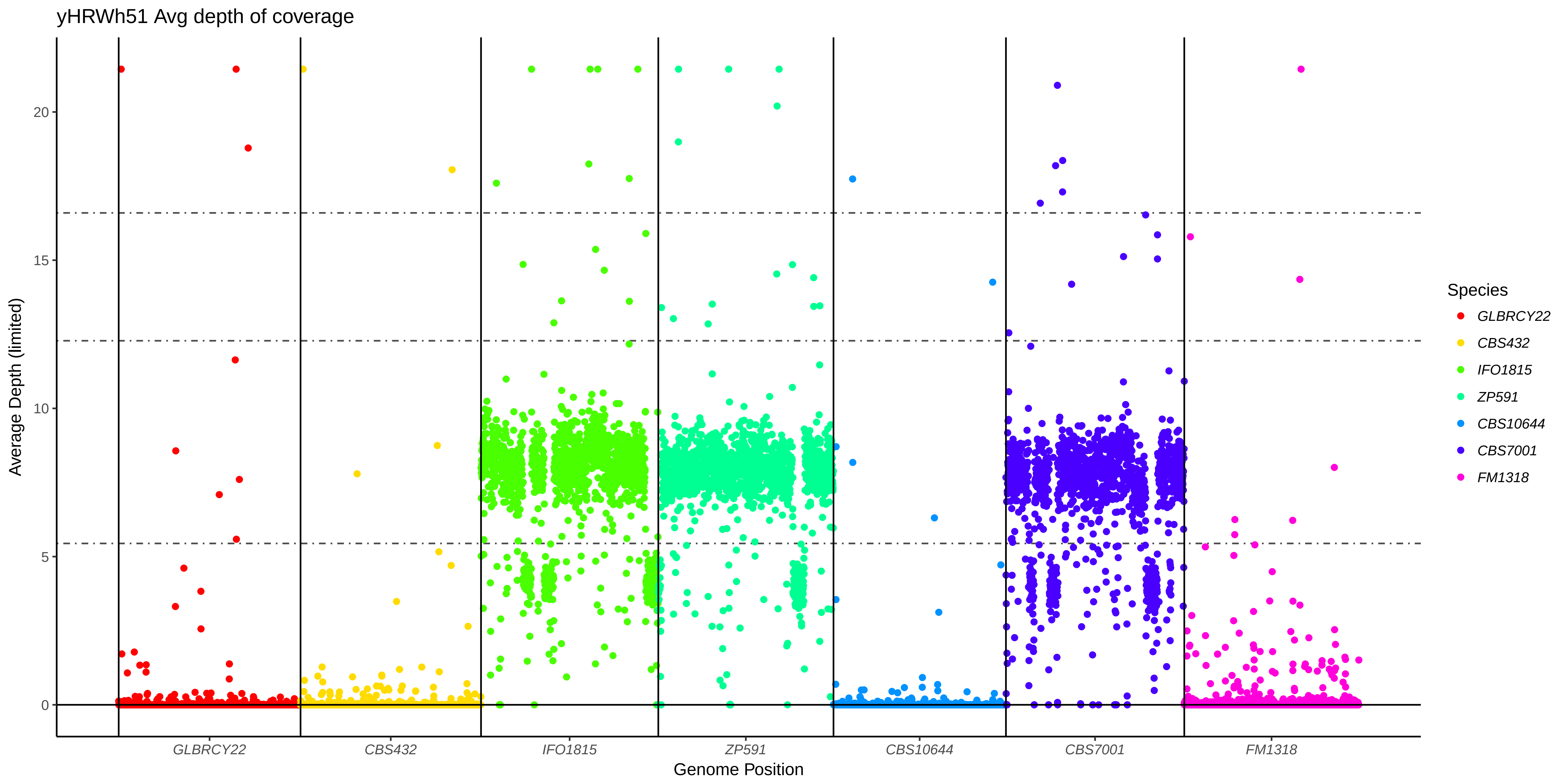

yHRWh51 Avg depth of coverage
Average Depth (limited)
20
15
10
5
0
GLBRCY22
CBS432
IFO1815
ZP591
CBS10644
CBS7001
FM1318
Genome Position
Species
GLBRCY22
CBS432
IFO1815
ZP591
CBS10644
CBS7001
FM1318

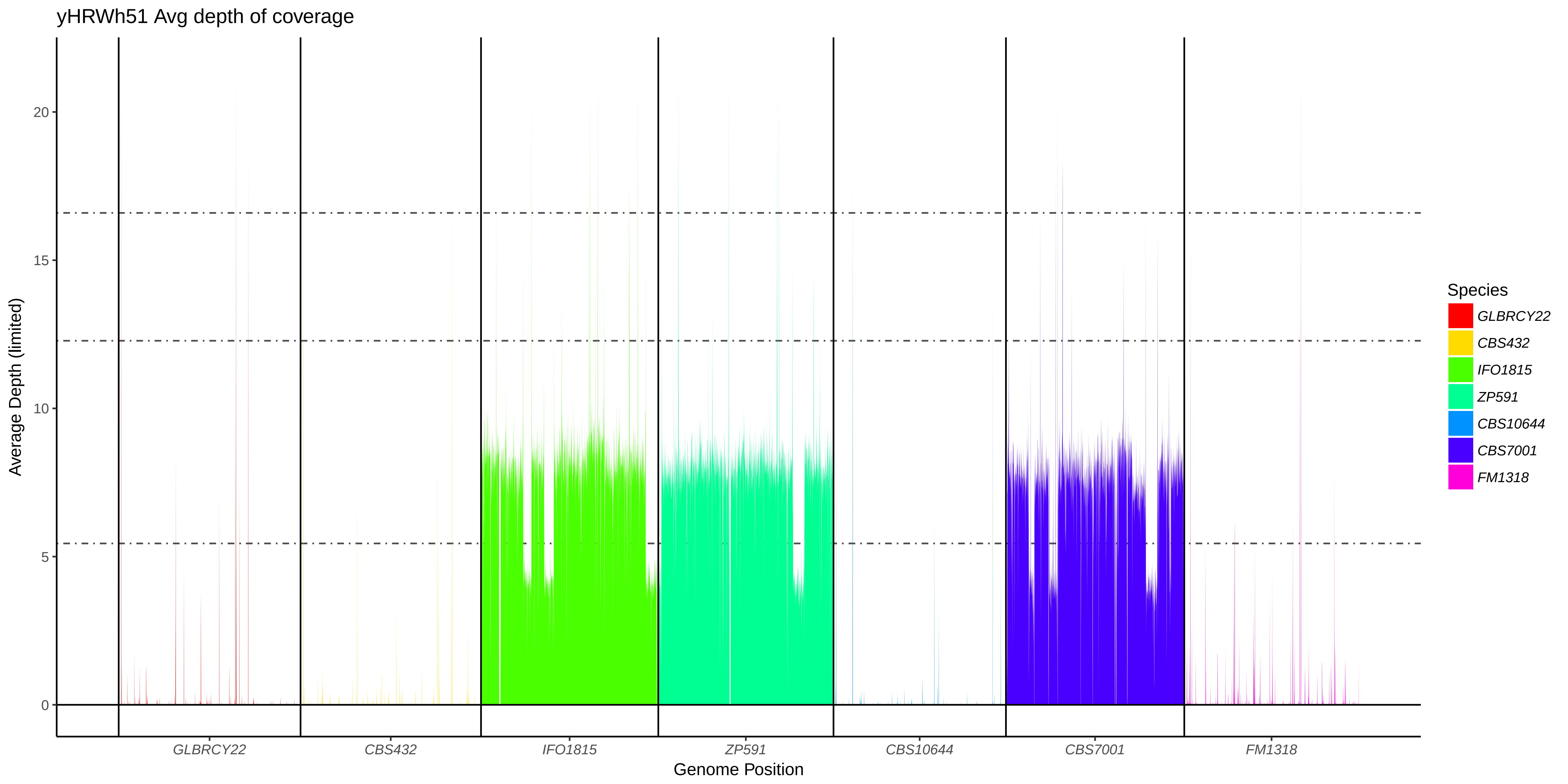
yHRWh51 Avg depth of coverage
Average Depth (limited)
0
5
10
15
20
GLBRCY22
CBS432
IFO1815
ZP591
CBS10644
CBS7001
FM1318
Genome Position
Species
GLBRCY22
CBS432
IFO1815
ZP591
CBS10644
CBS7001
FM1318

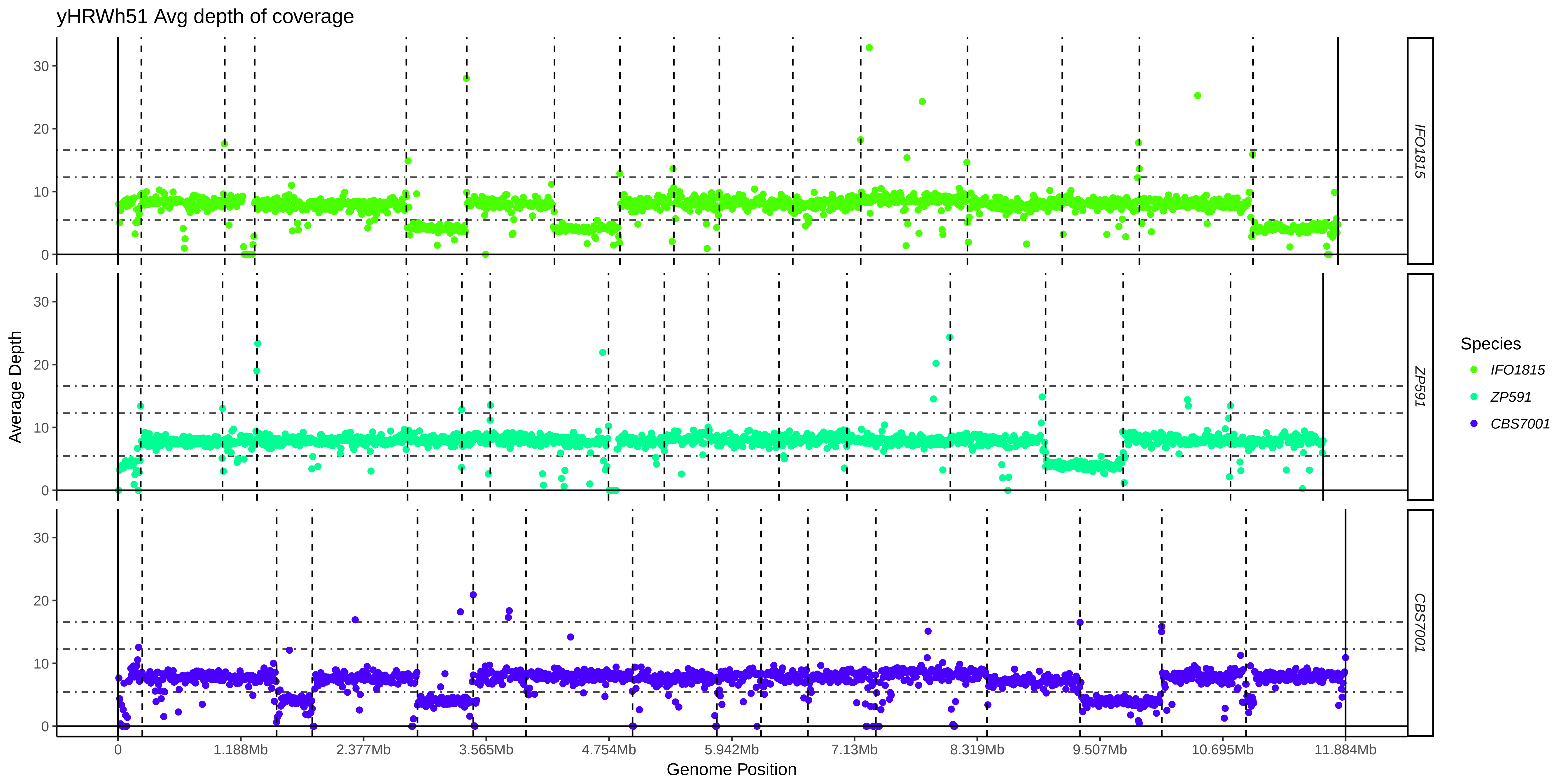

yHRWh51 Avg depth of coverage
Average Depth
Genome Position
0
10
20
30
0
1.188Mb
2.377Mb
3.565Mb
4.754Mb
5.942Mb
7.13Mb
8.319Mb
9.507Mb
10.695Mb
11.884Mb
IFO1815
ZP591
CBS7001
Species
IFO1815
ZP591
CBS7001

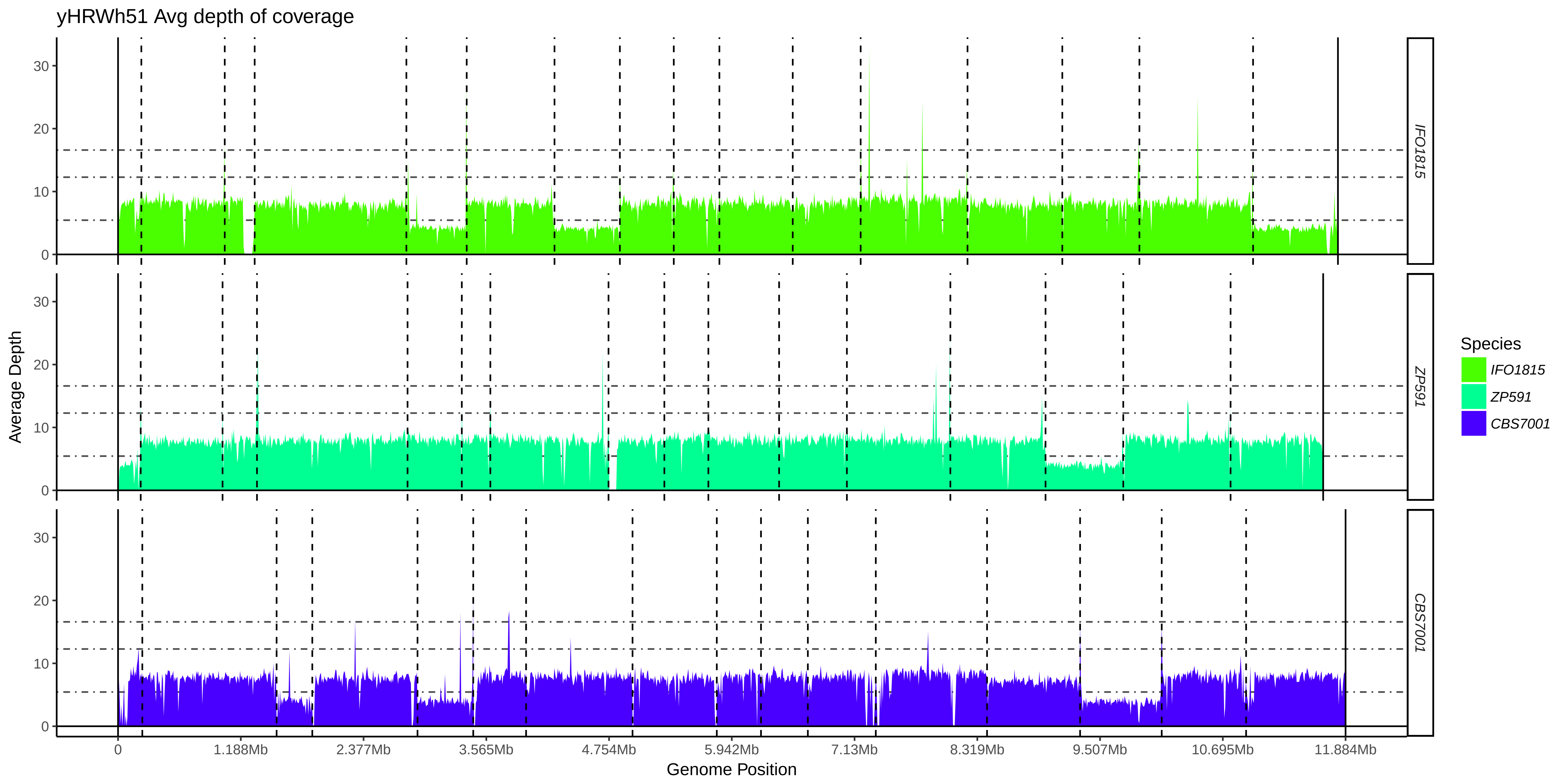

yHRWh51 Avg depth of coverage
Average Depth
30
20
10
0
IFO1815
ZP591
CBS7001
Species
IFO1815
ZP591
CBS7001
0
1.188Mb
2.377Mb
3.565Mb
4.754Mb
5.942Mb
7.13Mb
8.319Mb
9.507Mb
10.695Mb
11.884Mb
Genome Position

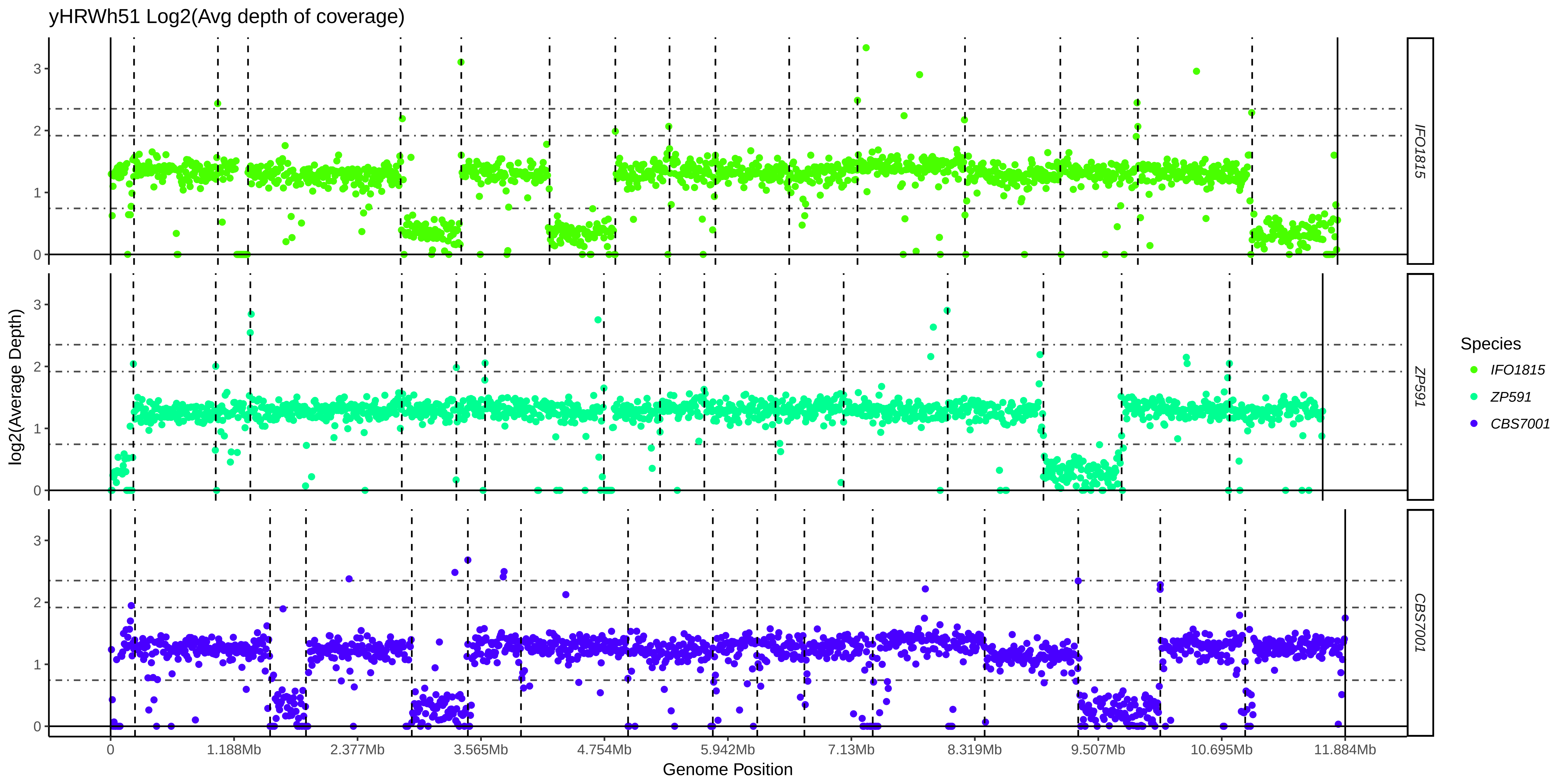

yHRWh51 Log2(Avg depth of coverage)
log2(Average Depth)
Genome Position
0
1.188Mb
2.377Mb
3.565Mb
4.754Mb
5.942Mb
7.13Mb
8.319Mb
9.507Mb
10.695Mb
11.884Mb
IFO1815
ZP591
CBS7001
Species
IFO1815
ZP591
CBS7001

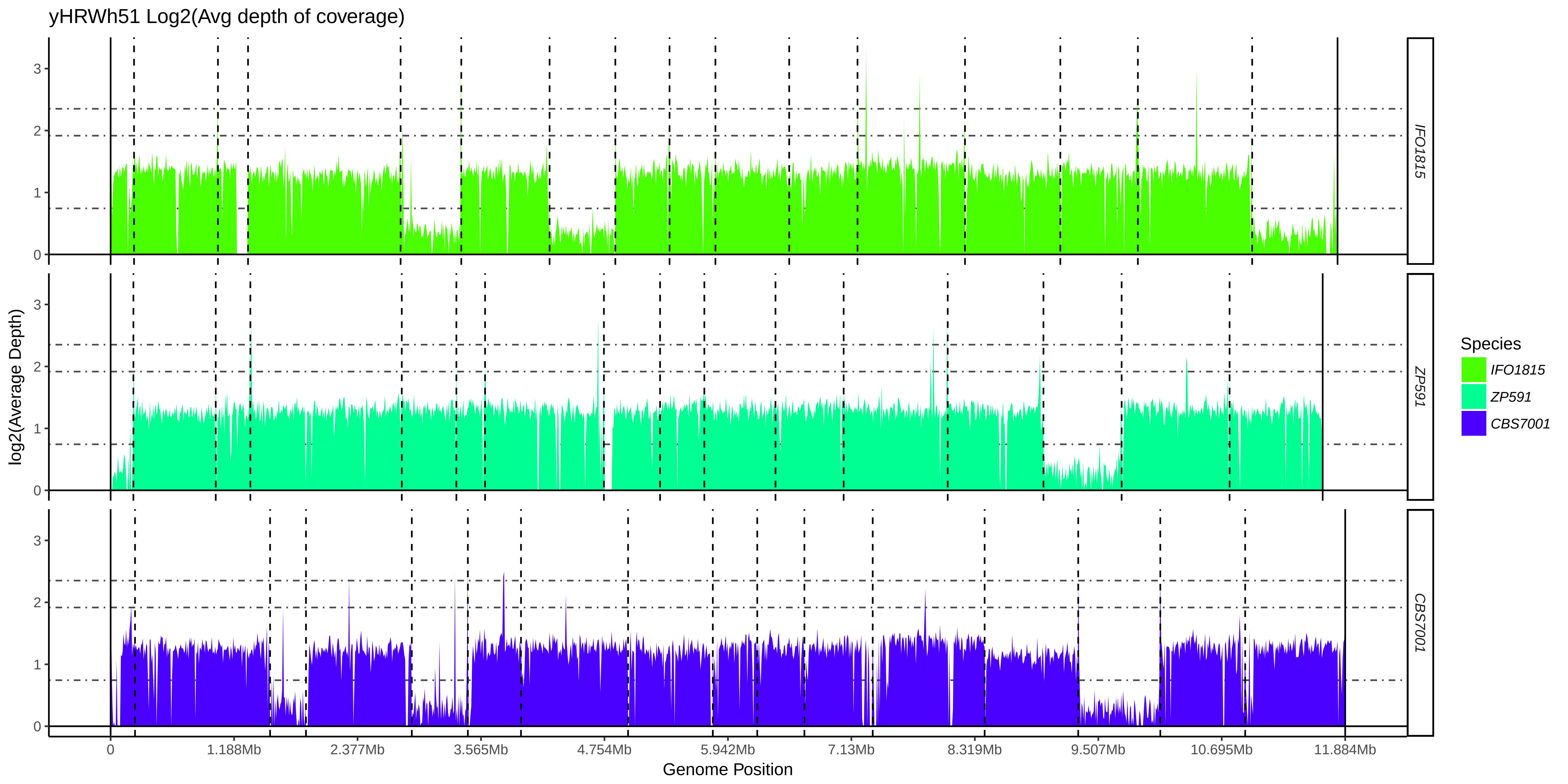
yHRWh51 Log2(Avg depth of coverage)
log2(Average Depth)
Genome Position
0
1.188Mb
2.377Mb
3.565Mb
4.754Mb
5.942Mb
7.13Mb
8.319Mb
9.507Mb
10.695Mb
11.884Mb
IFO1815
ZP591
CBS7001
Species
IFO1815
ZP591
CBS7001

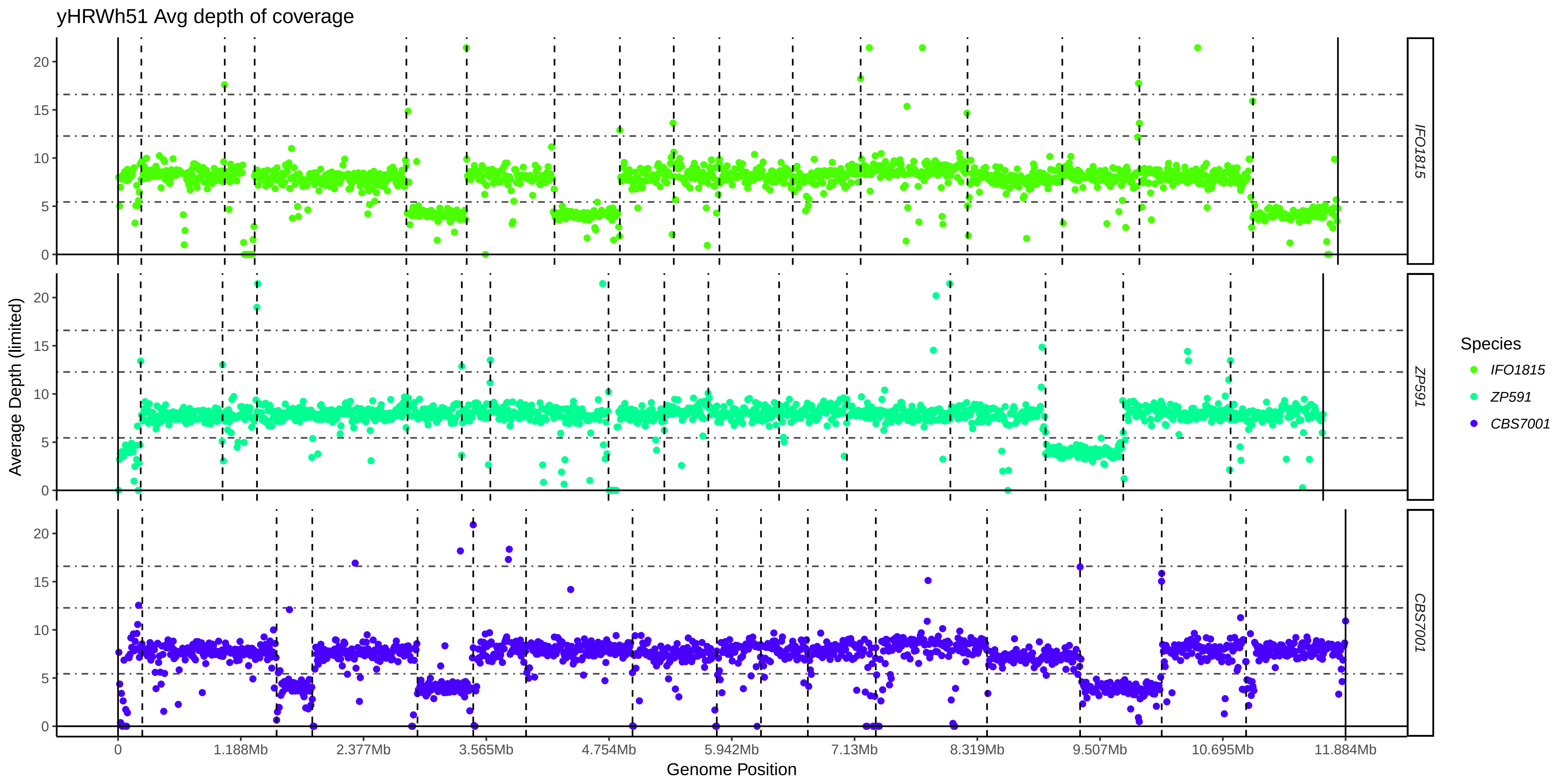
yHRWh51 Avg depth of coverage
Average Depth (limited)
Genome Position
0
1.188Mb
2.377Mb
3.565Mb
4.754Mb
5.942Mb
7.13Mb
8.319Mb
9.507Mb
10.695Mb
11.884Mb
0
5
10
15
20
IFO1815
ZP591
CBS7001
Species
IFO1815
ZP591
CBS7001

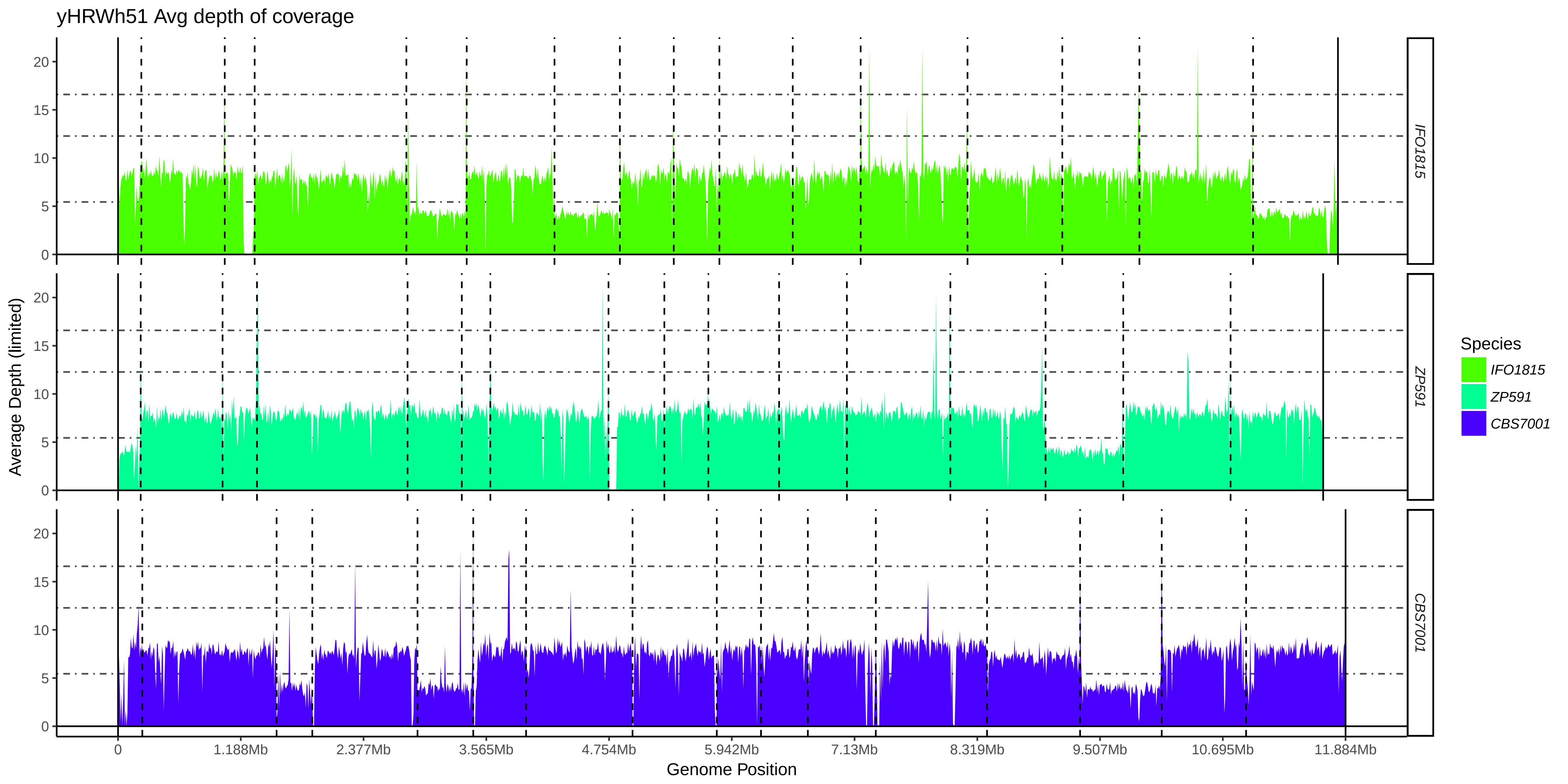

yHRWh51 Avg depth of coverage
Average Depth (limited)
Genome Position
0
1.188Mb
2.377Mb
3.565Mb
4.754Mb
5.942Mb
7.13Mb
8.319Mb
9.507Mb
10.695Mb
11.884Mb
0
5
10
15
20
IFO1815
ZP591
CBS7001
Species
IFO1815
ZP591
CBS7001
