# Supplementary material for: Synthetic hybrids of six yeast species
Source: Nat Commun. 2020 Apr 29;11:2085. doi: 10.1038/s41467-020-15559-4 (PMC7190663; doi:10.1038/s41467-020-15559-4)
Supplement: Supplementary file 15 — Source Data [file 41467_2020_15559_MOESM15_ESM.zip › Source Data 2.zip!/yHRWh85_sppIDerDepthPlot-covBins.pdf]

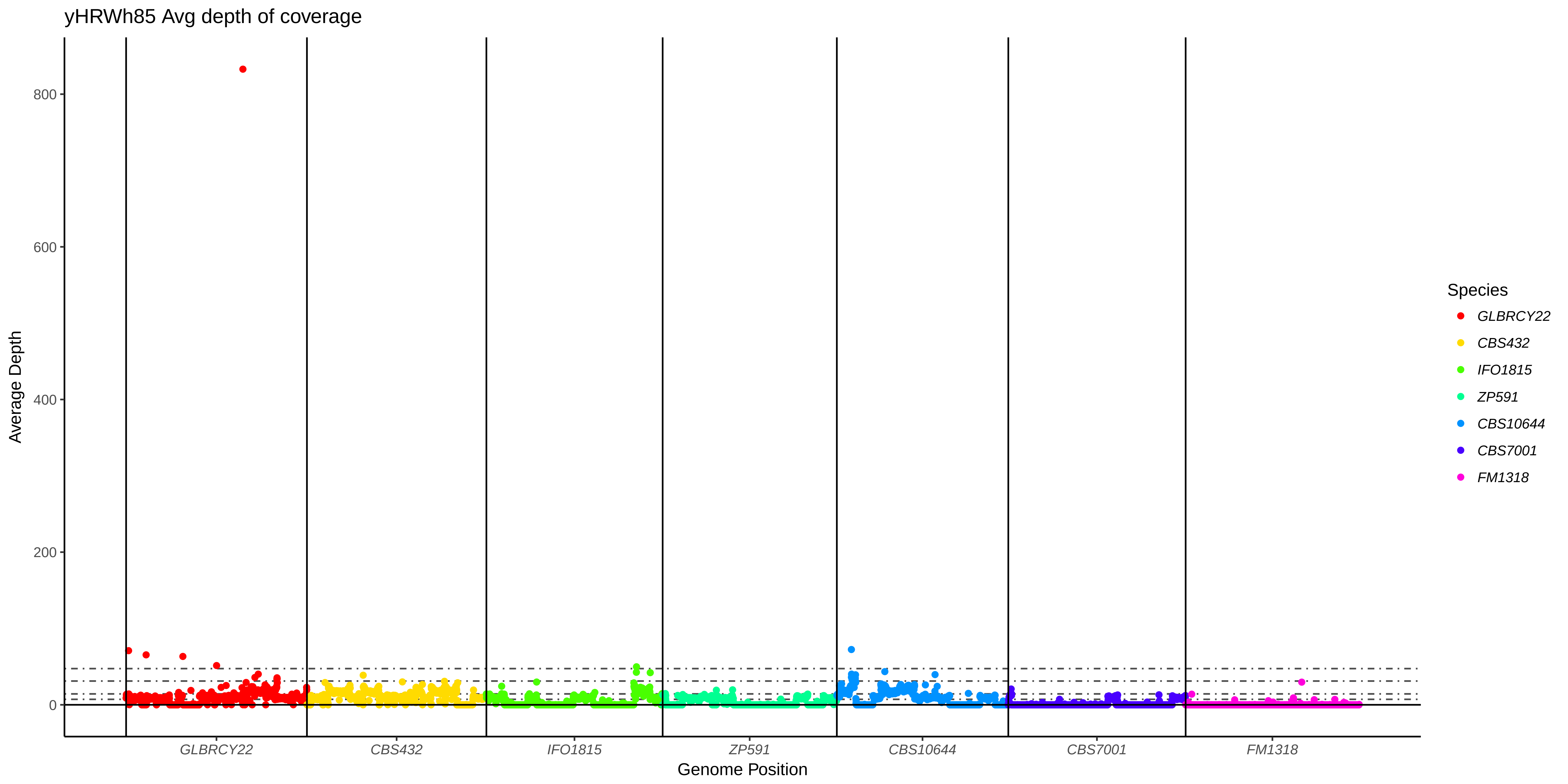
yHRWh85 Avg depth of coverage
Average Depth
800
600
400
200
0
GLBRCY22
CBS432
IFO1815
ZP591
CBS10644
CBS7001
FM1318
Genome Position
Species
GLBRCY22
CBS432
IFO1815
ZP591
CBS10644
CBS7001
FM1318

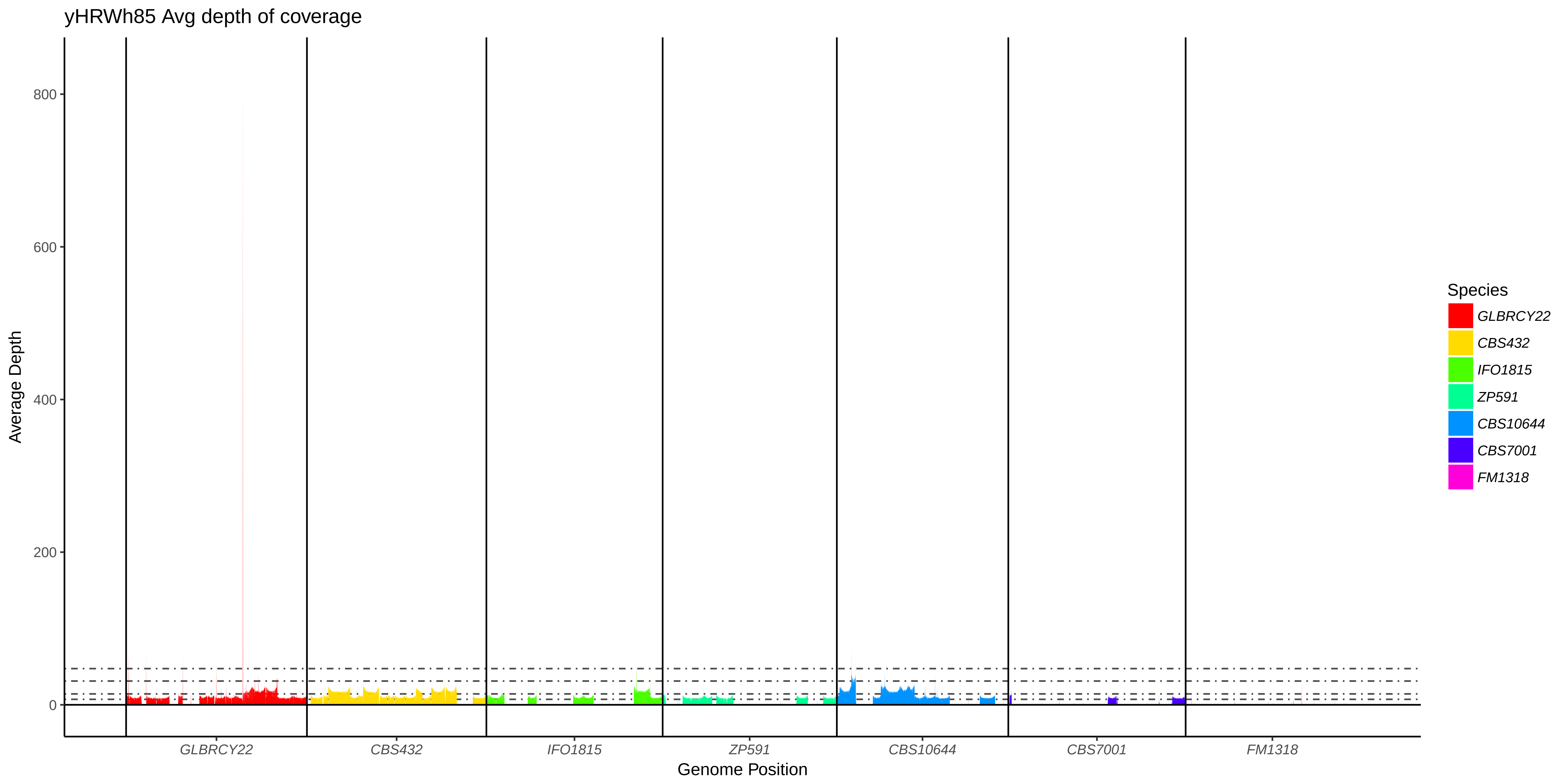

yHRWh85 Avg depth of coverage
Average Depth
800
600
400
200
0
GLBRCY22
CBS432
IFO1815
ZP591
CBS10644
CBS7001
FM1318
Genome Position
Species
GLBRCY22
CBS432
IFO1815
ZP591
CBS10644
CBS7001
FM1318

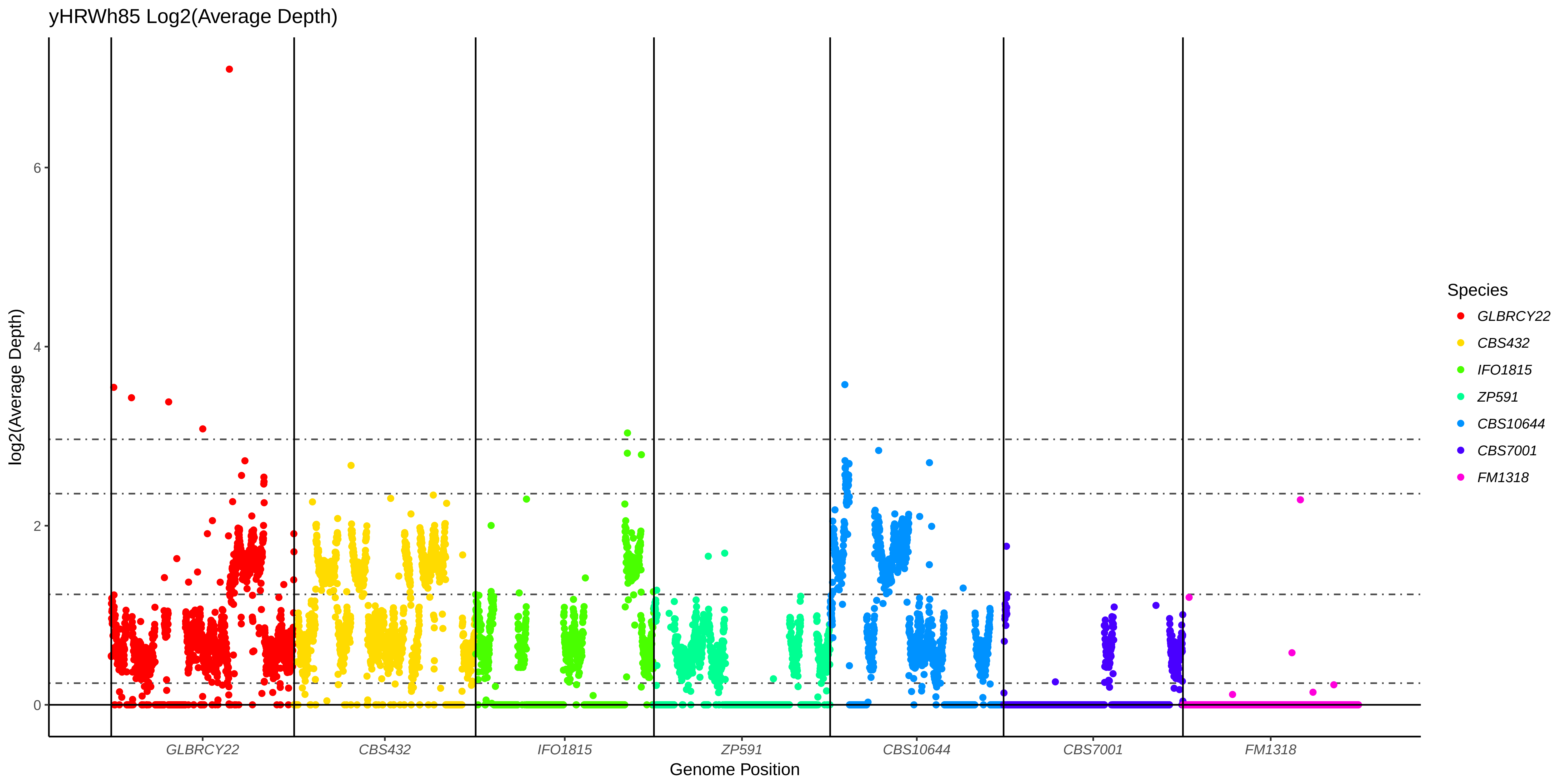

yHRWh85 Log2(Average Depth)
log2(Average Depth)
0
2
4
6
Genome Position
GLBRCY22
CBS432
IFO1815
ZP591
CBS10644
CBS7001
FM1318
Species
GLBRCY22
CBS432
IFO1815
ZP591
CBS10644
CBS7001
FM1318

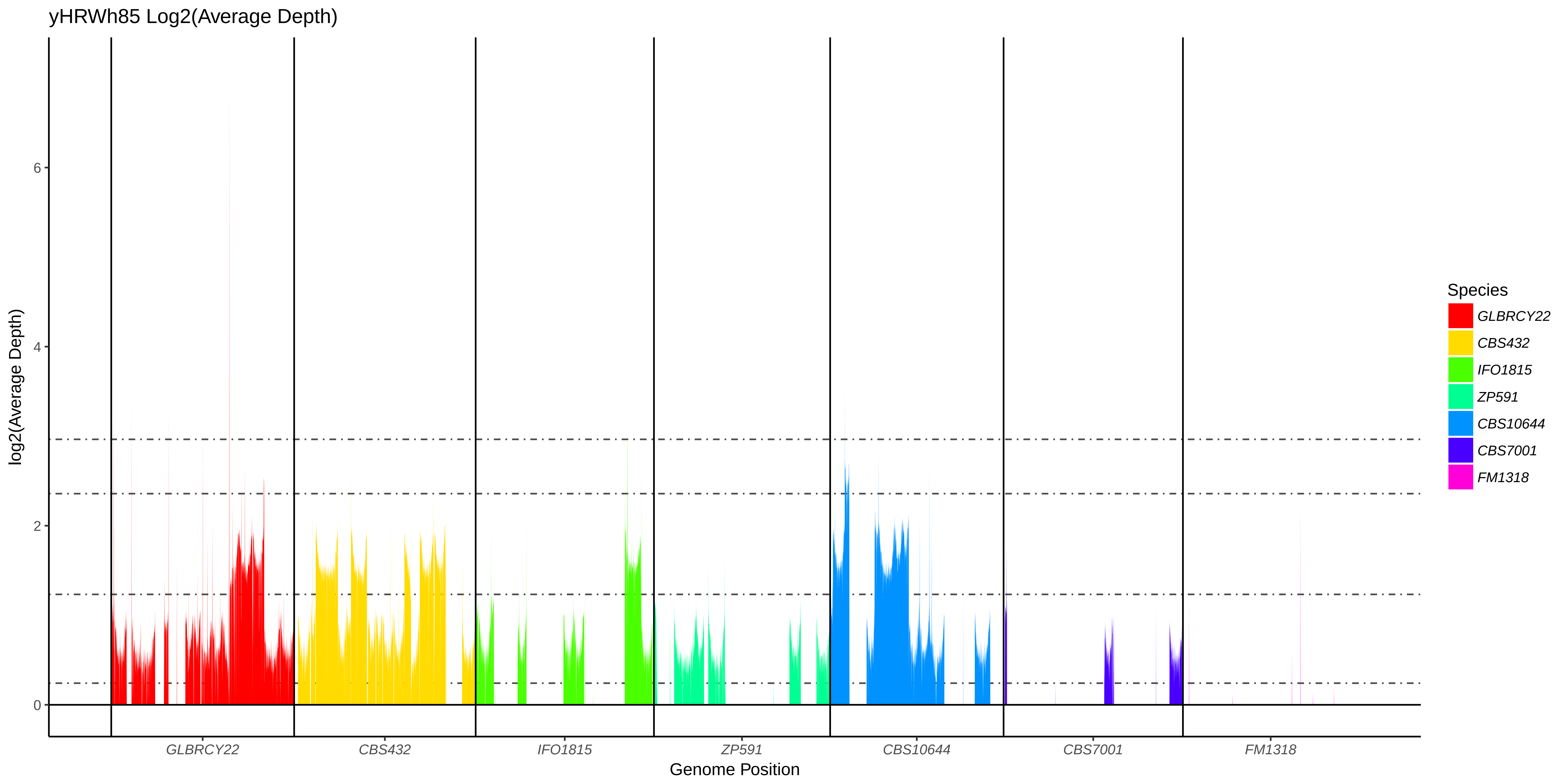
yHRWh85 Log2(Average Depth)
log2(Average Depth)
0
2
4
6
GLBRCY22
CBS432
IFO1815
ZP591
CBS10644
CBS7001
FM1318
Genome Position
Species
GLBRCY22
CBS432
IFO1815
ZP591
CBS10644
CBS7001
FM1318

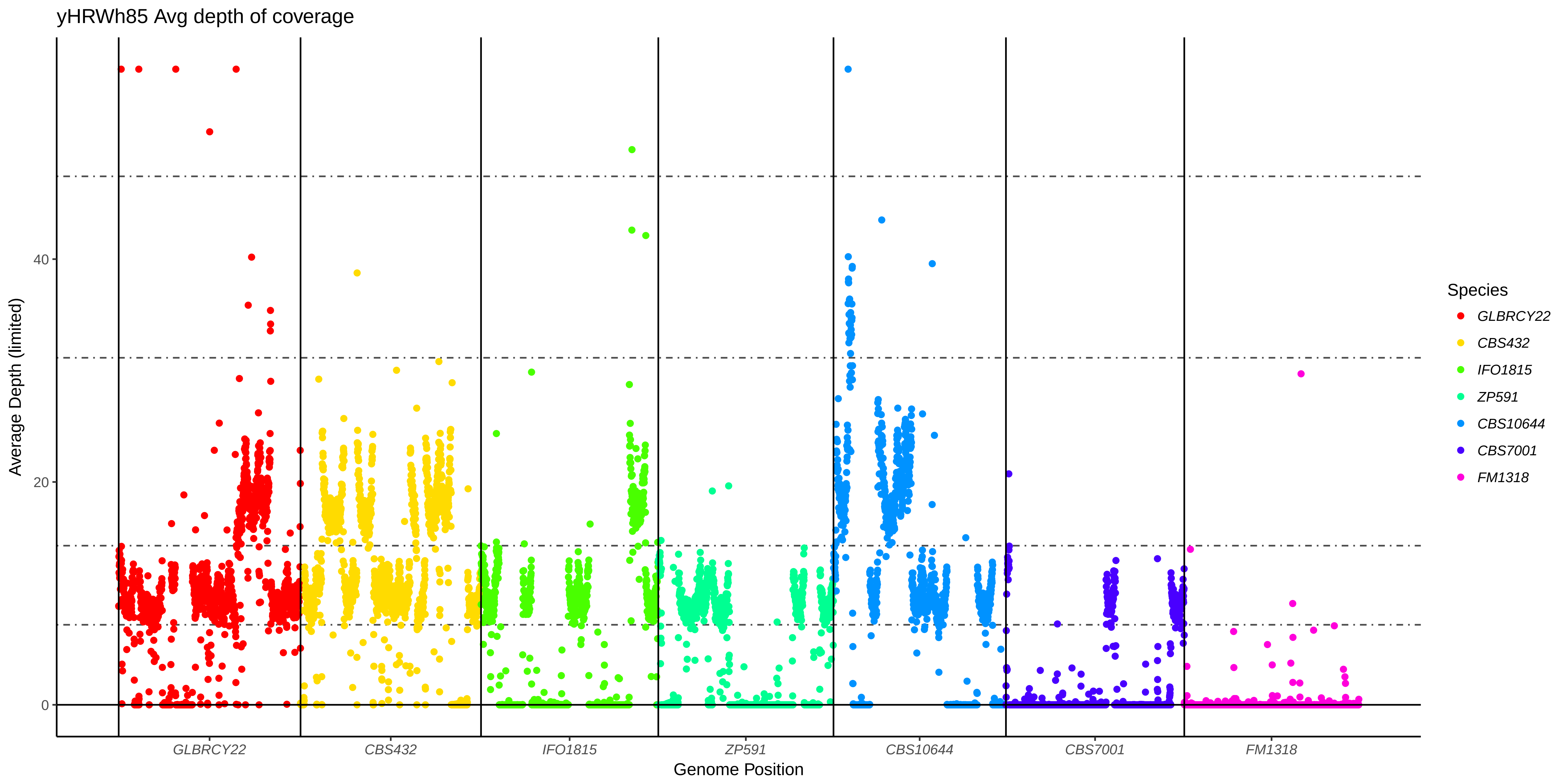

yHRWh85 Avg depth of coverage
Average Depth (limited)
0
20
40
GLBRCY22
CBS432
IFO1815
ZP591
CBS10644
CBS7001
FM1318
Genome Position
Species
GLBRCY22
CBS432
IFO1815
ZP591
CBS10644
CBS7001
FM1318

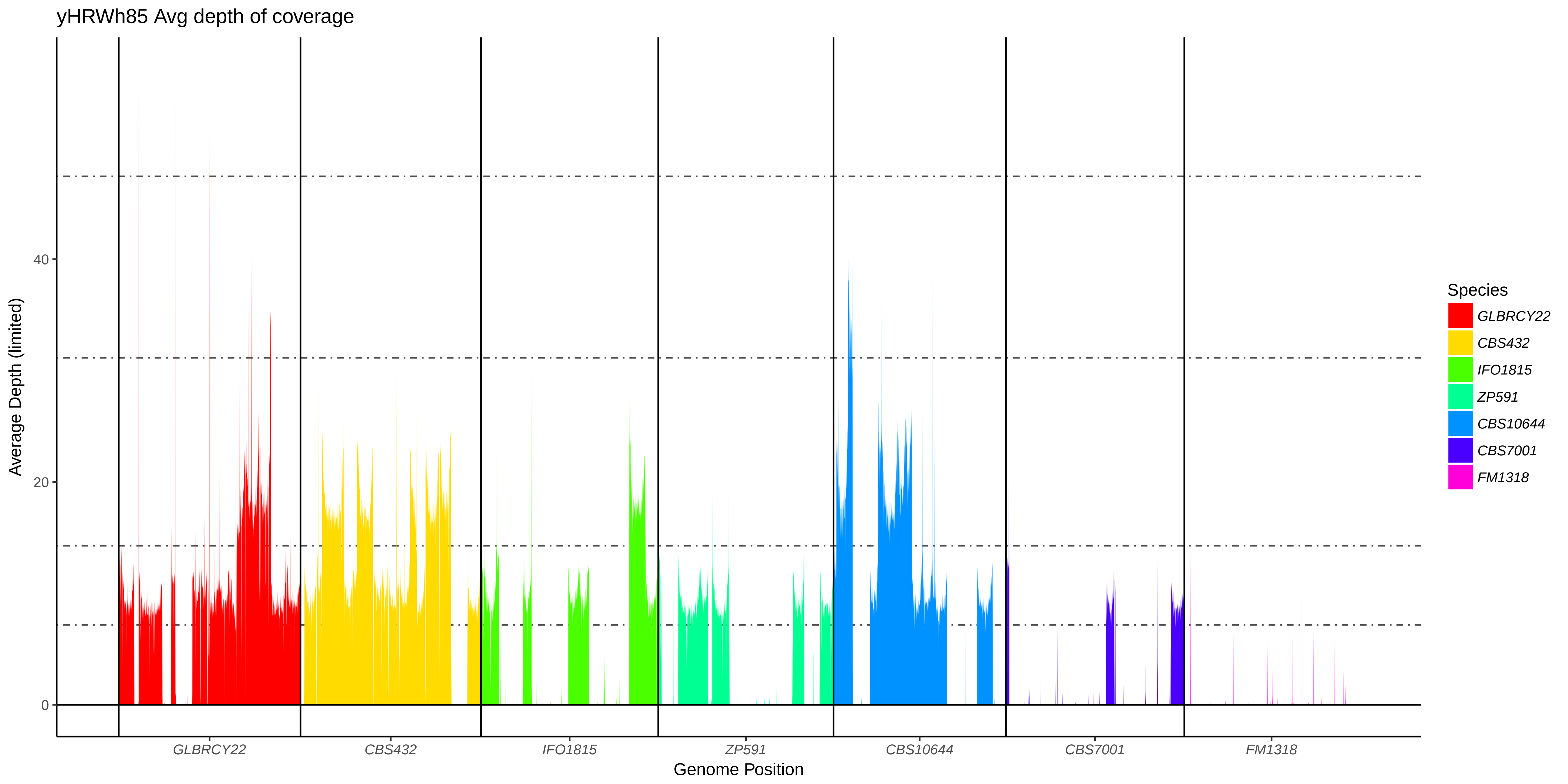

yHRWh85 Avg depth of coverage
Average Depth (limited)
0
20
40
GLBRCY22
CBS432
IFO1815
ZP591
CBS10644
CBS7001
FM1318
Genome Position
Species
GLBRCY22
CBS432
IFO1815
ZP591
CBS10644
CBS7001
FM1318

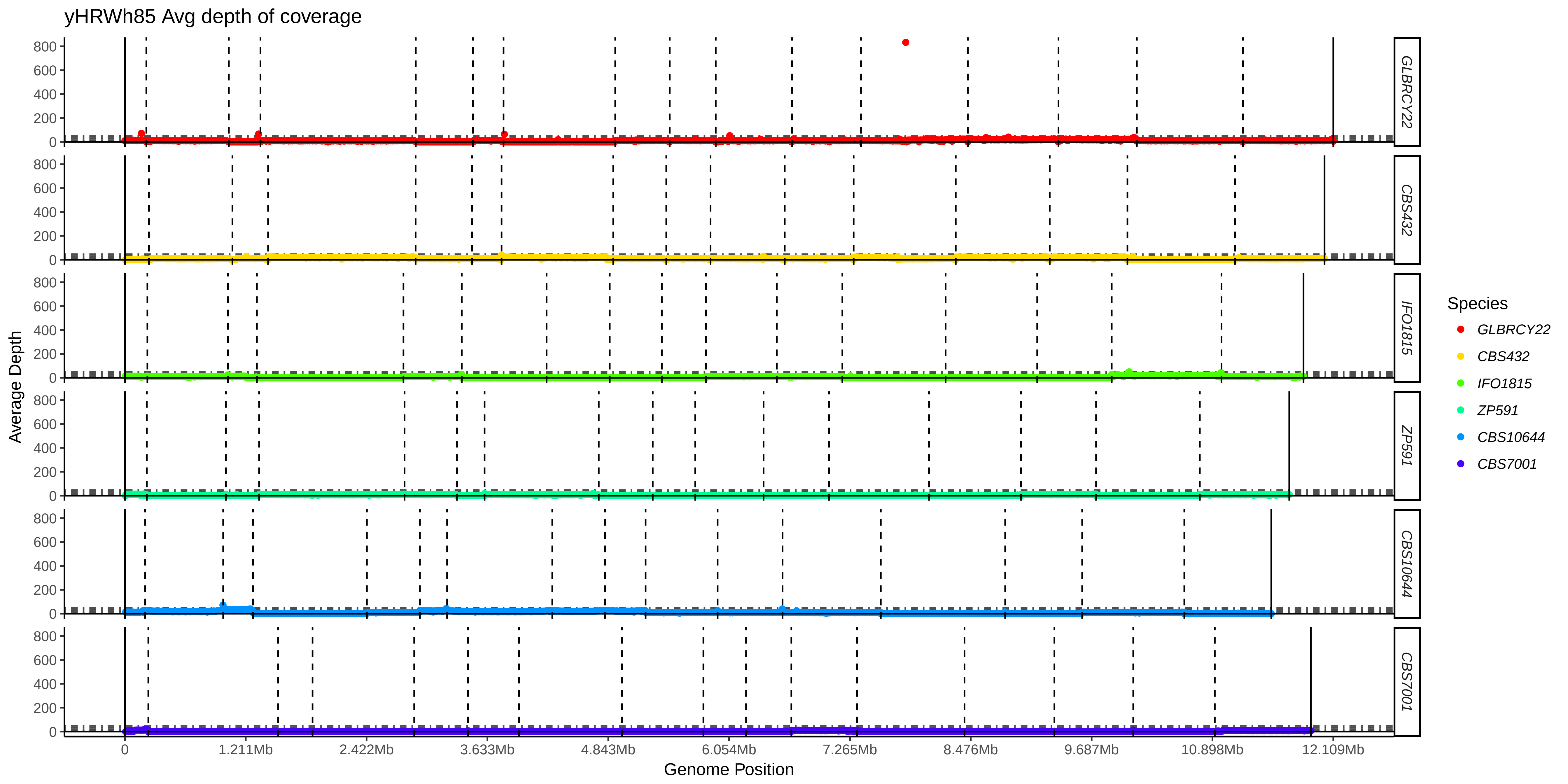

yHRWh85 Avg depth of coverage
Average Depth
800
600
400
200
0
GLBRCY22
CBS432
IFO1815
ZP591
CBS10644
CBS7001
0
1.211Mb
2.422Mb
3.633Mb
4.843Mb
6.054Mb
7.265Mb
8.476Mb
9.687Mb
10.898Mb
12.109Mb
Genome Position
Species
GLBRCY22
CBS432
IFO1815
ZP591
CBS10644
CBS7001

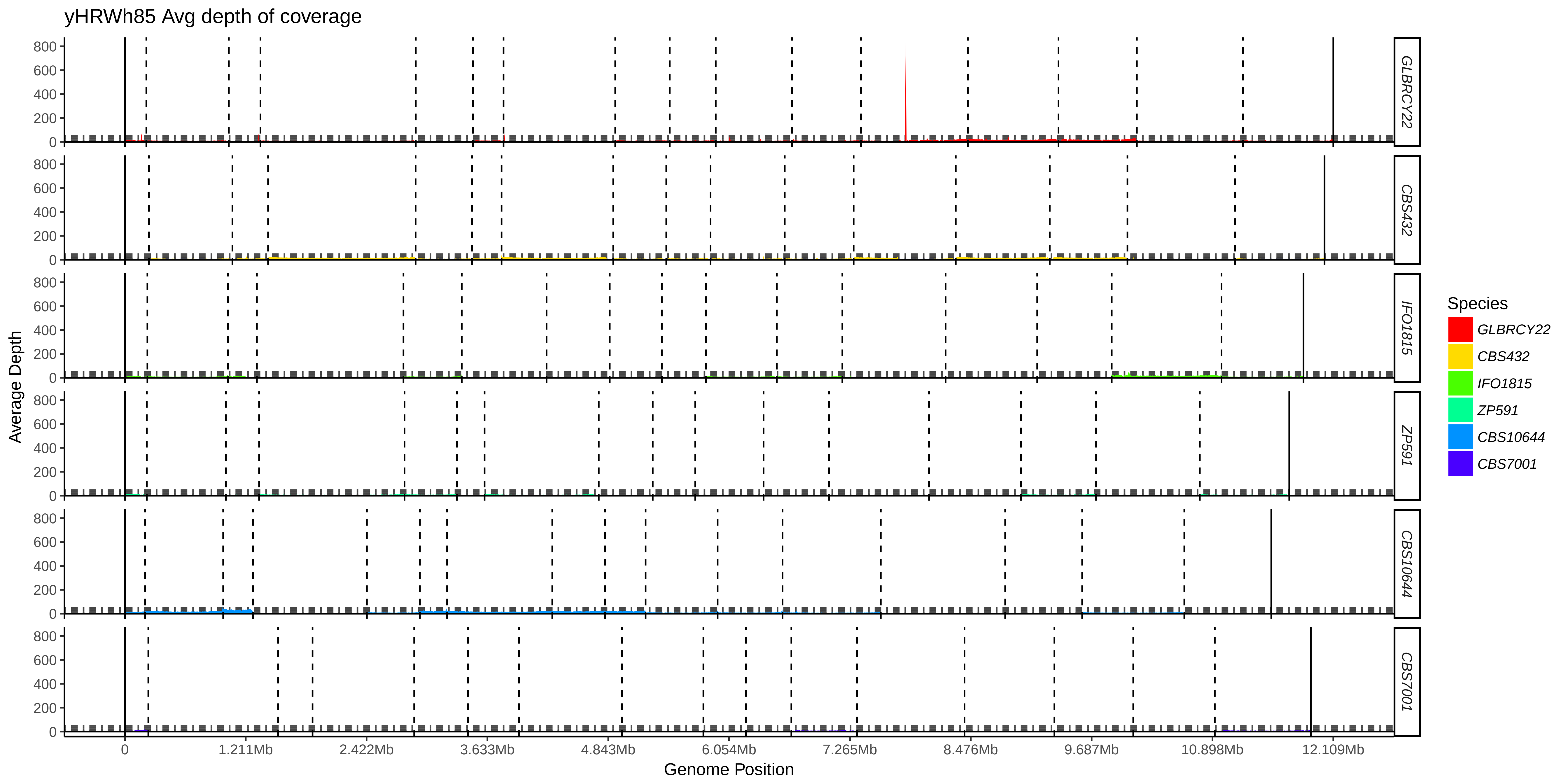
yHRWh85 Avg depth of coverage
Average Depth
Genome Position
0
1.211Mb
2.422Mb
3.633Mb
4.843Mb
6.054Mb
7.265Mb
8.476Mb
9.687Mb
10.898Mb
12.109Mb
GLBRCY22
CBS432
IFO1815
ZP591
CBS10644
CBS7001
Species
GLBRCY22
CBS432
IFO1815
ZP591
CBS10644
CBS7001

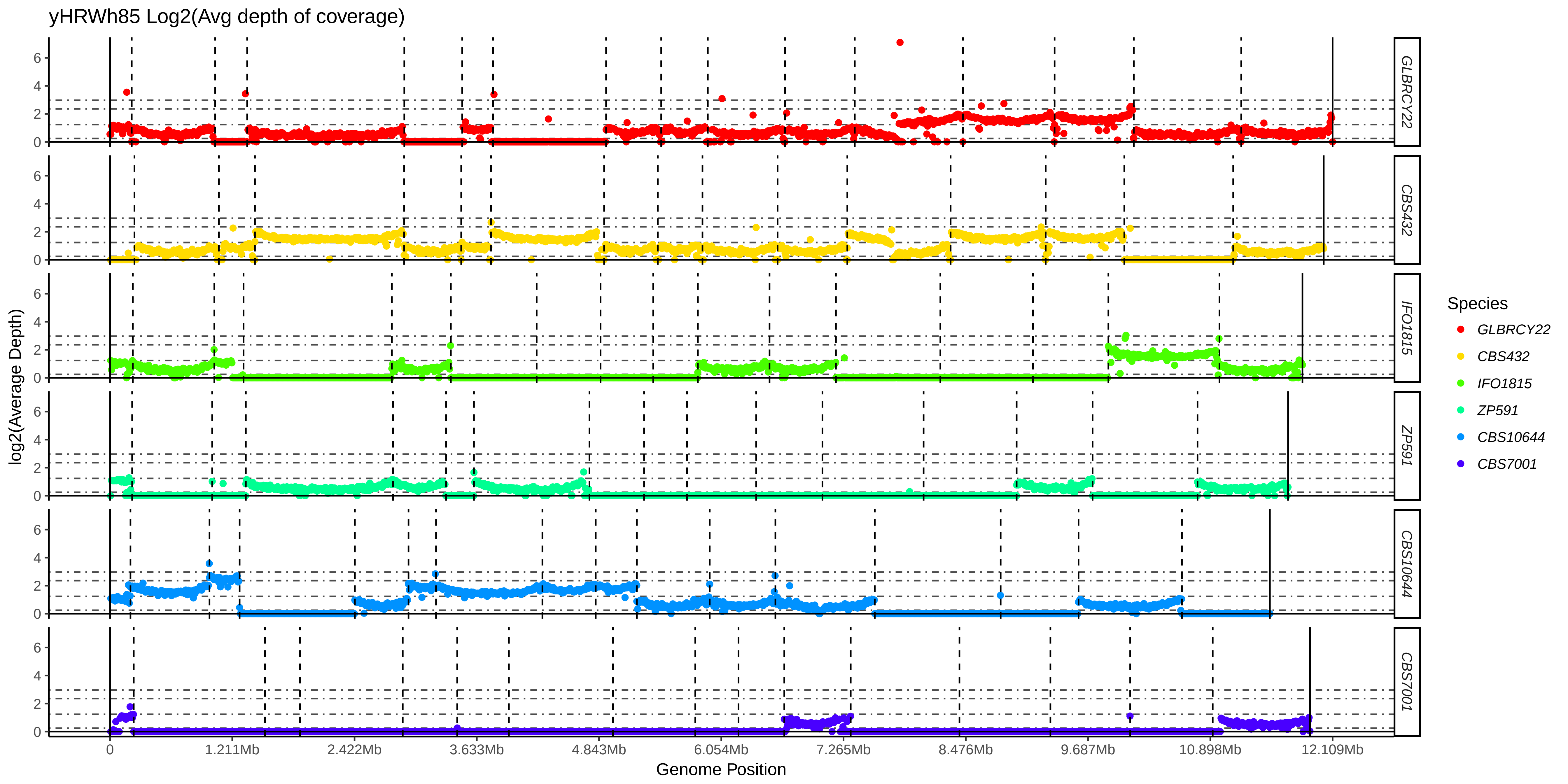

yHRWh85 Log2(Avg depth of coverage)
log2(Average Depth)
Genome Position
0
1.211Mb
2.422Mb
3.633Mb
4.843Mb
6.054Mb
7.265Mb
8.476Mb
9.687Mb
10.898Mb
12.109Mb
GLBRCY22
CBS432
IFO1815
ZP591
CBS10644
CBS7001
Species
GLBRCY22
CBS432
IFO1815
ZP591
CBS10644
CBS7001

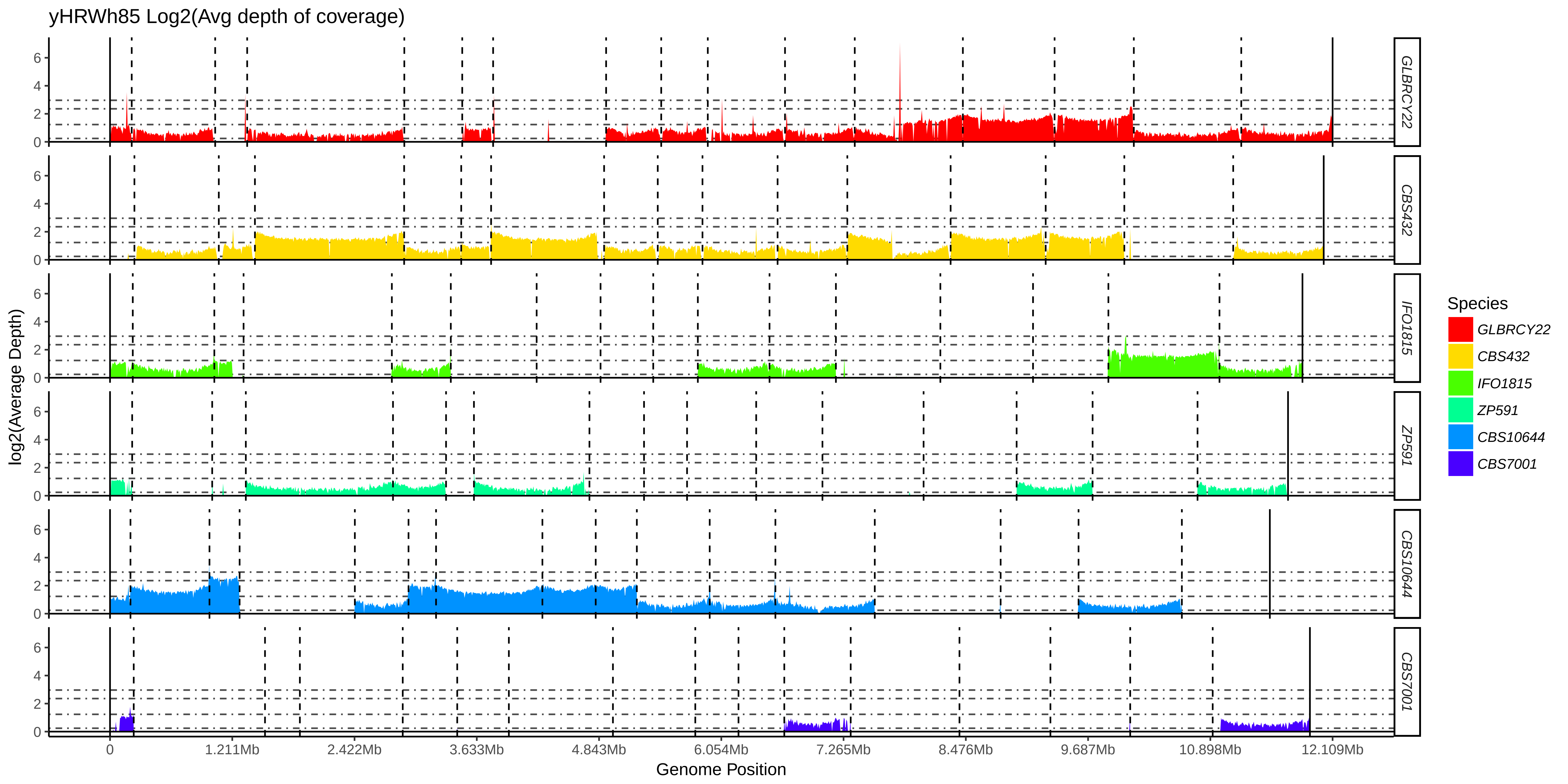

yHRWh85 Log2(Avg depth of coverage)
log2(Average Depth)
Genome Position
0
1.211Mb
2.422Mb
3.633Mb
4.843Mb
6.054Mb
7.265Mb
8.476Mb
9.687Mb
10.898Mb
12.109Mb
GLBRCY22
CBS432
IFO1815
ZP591
CBS10644
CBS7001
Species

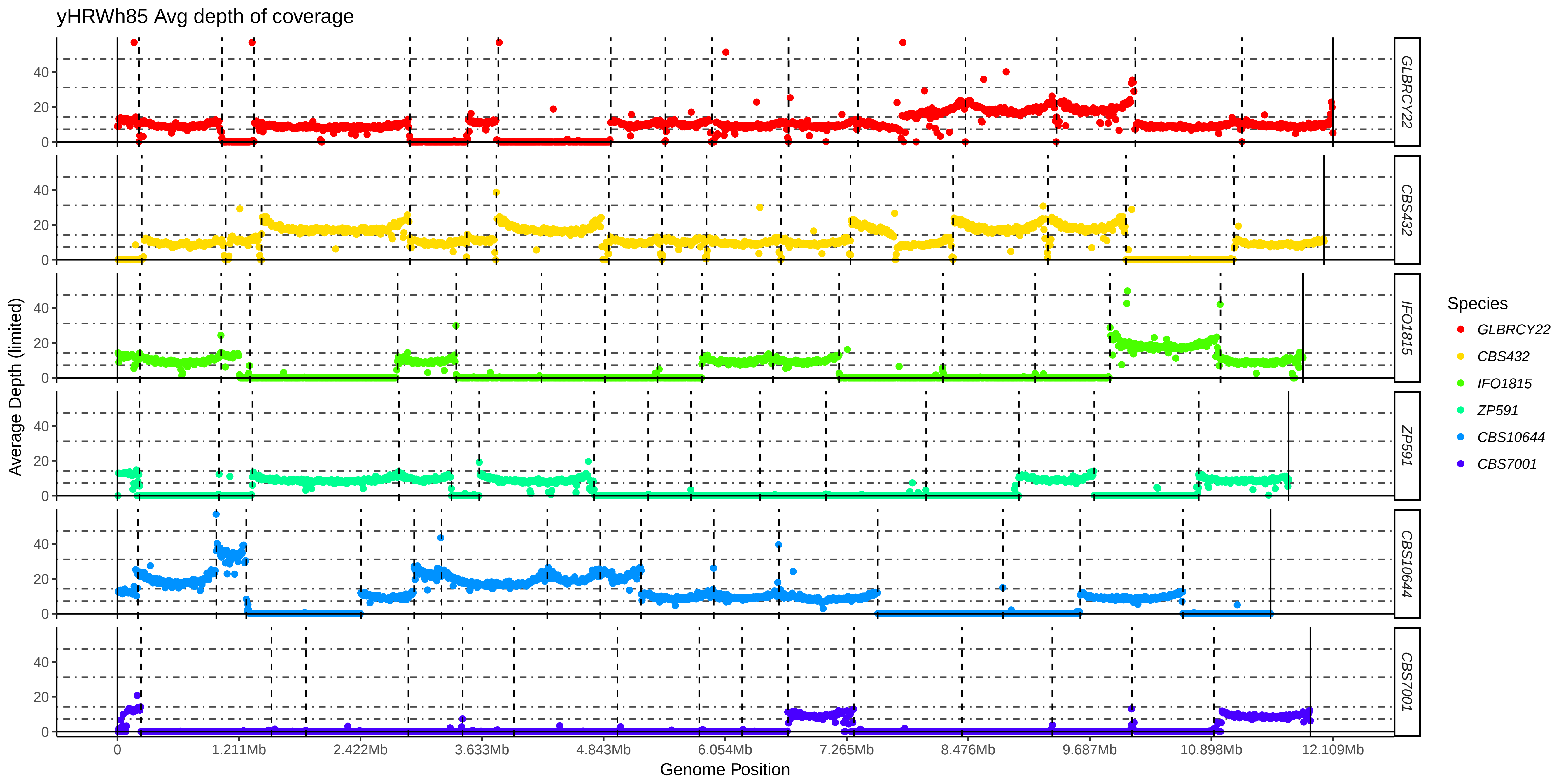

yHRWh85 Avg depth of coverage
Average Depth (limited)
Genome Position
0
1.211Mb
2.422Mb
3.633Mb
4.843Mb
6.054Mb
7.265Mb
8.476Mb
9.687Mb
10.898Mb
12.109Mb
0
20
40
GLBRCY22
CBS432
IFO1815
ZP591
CBS10644
CBS7001
Species

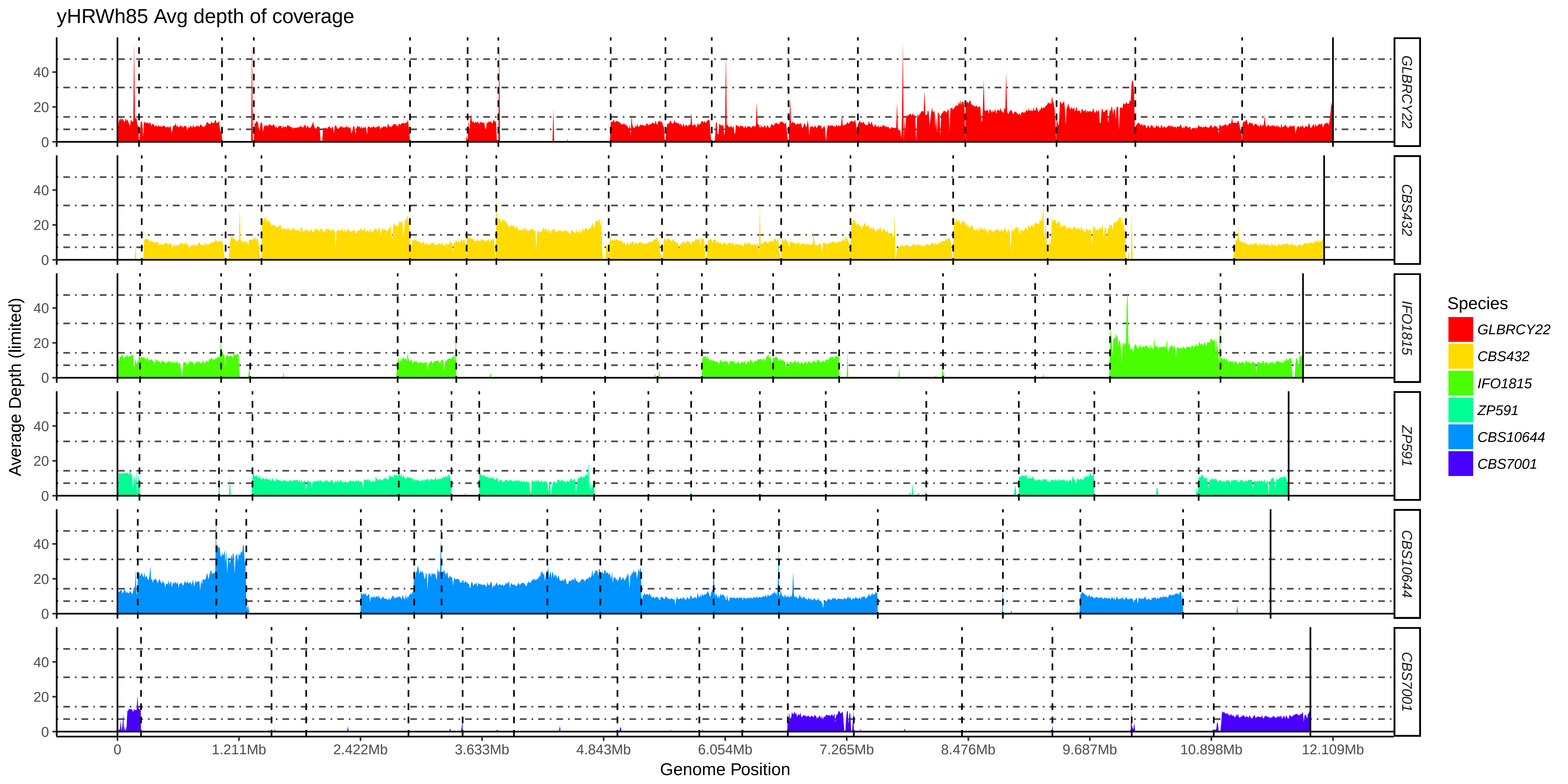

yHRWh85 Avg depth of coverage
Average Depth (limited)
Genome Position
0
1.211Mb
2.422Mb
3.633Mb
4.843Mb
6.054Mb
7.265Mb
8.476Mb
9.687Mb
10.898Mb
12.109Mb
0
20
40
GLBRCY22
CBS432
IFO1815
ZP591
CBS10644
CBS7001
Species
GLBRCY22
CBS432
IFO1815
ZP591
CBS10644
CBS7001
