# Supplementary material for: Synthetic hybrids of six yeast species
Source: Nat Commun. 2020 Apr 29;11:2085. doi: 10.1038/s41467-020-15559-4 (PMC7190663; doi:10.1038/s41467-020-15559-4)
Supplement: Supplementary file 15 — Source Data [file 41467_2020_15559_MOESM15_ESM.zip › Source Data 3.zip!/yHRWh4_mitoSppIDerDepthPlot-d.pdf]

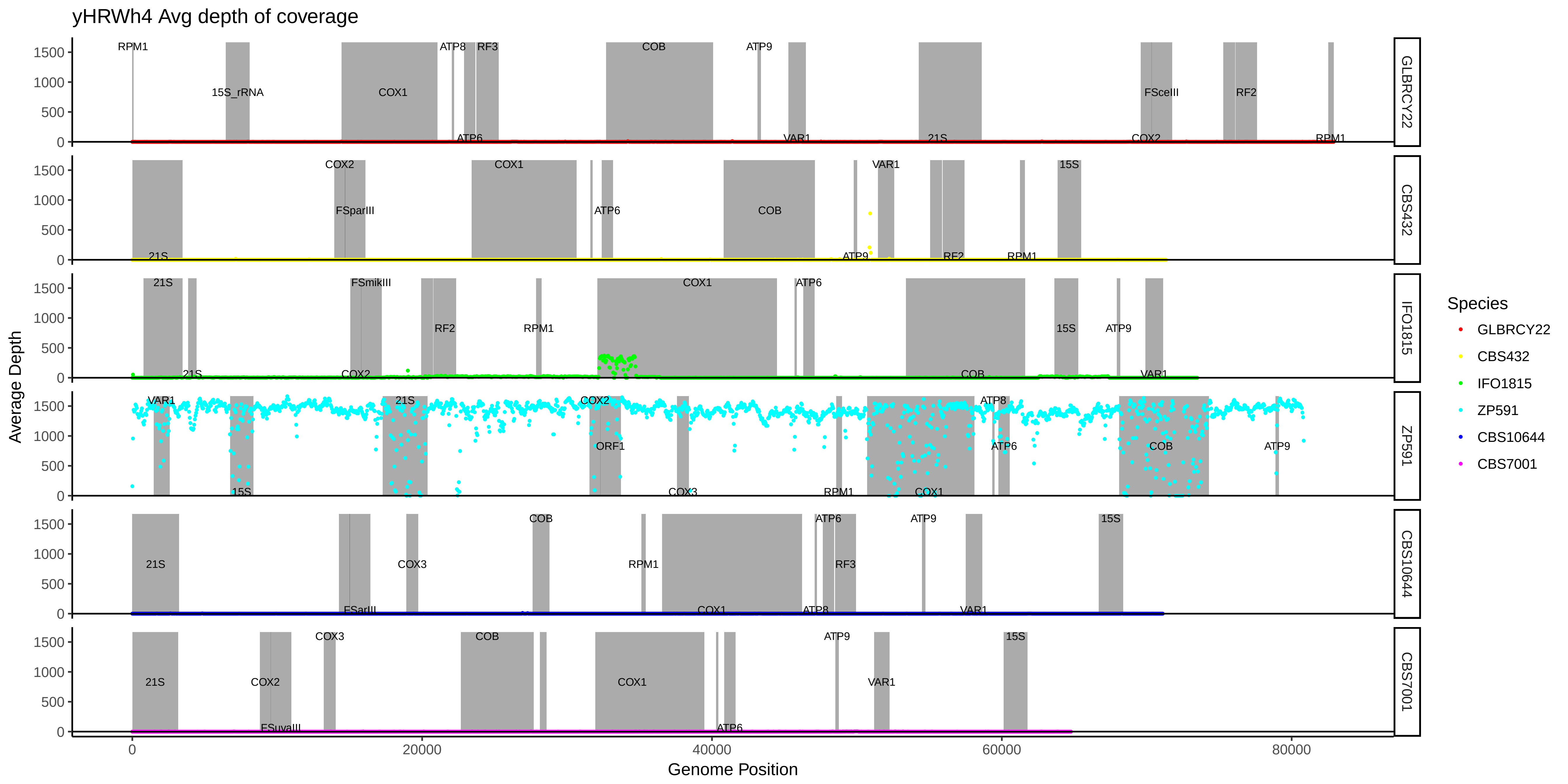
yHRWh4 Avg depth of coverage
Average Depth
Genome Position
0
20000
40000
60000
80000
0
500
1000
1500
GLBRCY22
CBS432
IFO1815
ZP591
CBS10644
CBS7001
Species
GLBRCY22
CBS432
IFO1815
ZP591
CBS10644
CBS7001
RPM1
15S_rRNA
COX1
ATP8
RF3
ATP6
COB
ATP9
VAR1
21S
FSceIII
COX2
RF2
FSparIII
FSmikIII
ORF1
COX3
15S
FSarIII
FSuvaIII

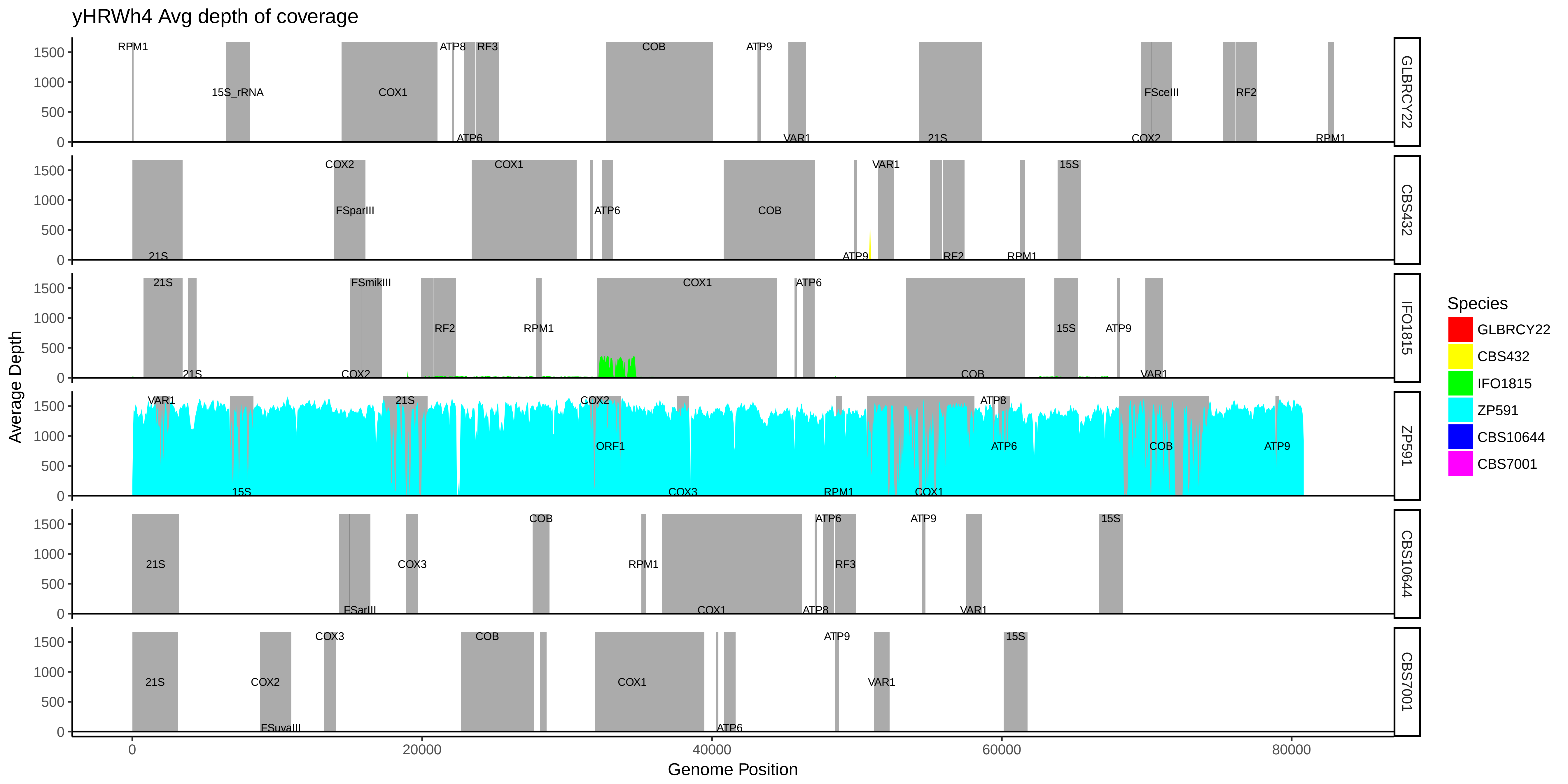
yHRWh4 Avg depth of coverage
Average Depth
Genome Position
GLBRCY22
CBS432
IFO1815
ZP591
CBS10644
CBS7001
Species
GLBRCY22
CBS432
IFO1815
ZP591
CBS10644
CBS7001
RPM1
15S_rRNA
COX1
ATP8
RF3
ATP6
COB
ATP9
VAR1
21S
FSceIII
COX2
RF2
21S
COX2
FSparIII
COX1
ATP6
COB
ATP9
VAR1
RF2
RPM1
15S
21S
FSmikIII
COX2
RF2
RPM1
COX1
ATP6
COB
15S
ATP9
VAR1
VAR1
15S
21S
COX2
ORF1
COX3
RPM1
ATP8
ATP6
COX1
COB
ATP9
21S
FSarIII
COX3
COB
RPM1
COX1
ATP6
ATP8
RF3
ATP9
VAR1
15S
21S
COX2
FSuvaIII
COX3
COB
COX1
ATP6
ATP9
VAR1
15S
0
500
1000
1500
0
20000
40000
60000
80000

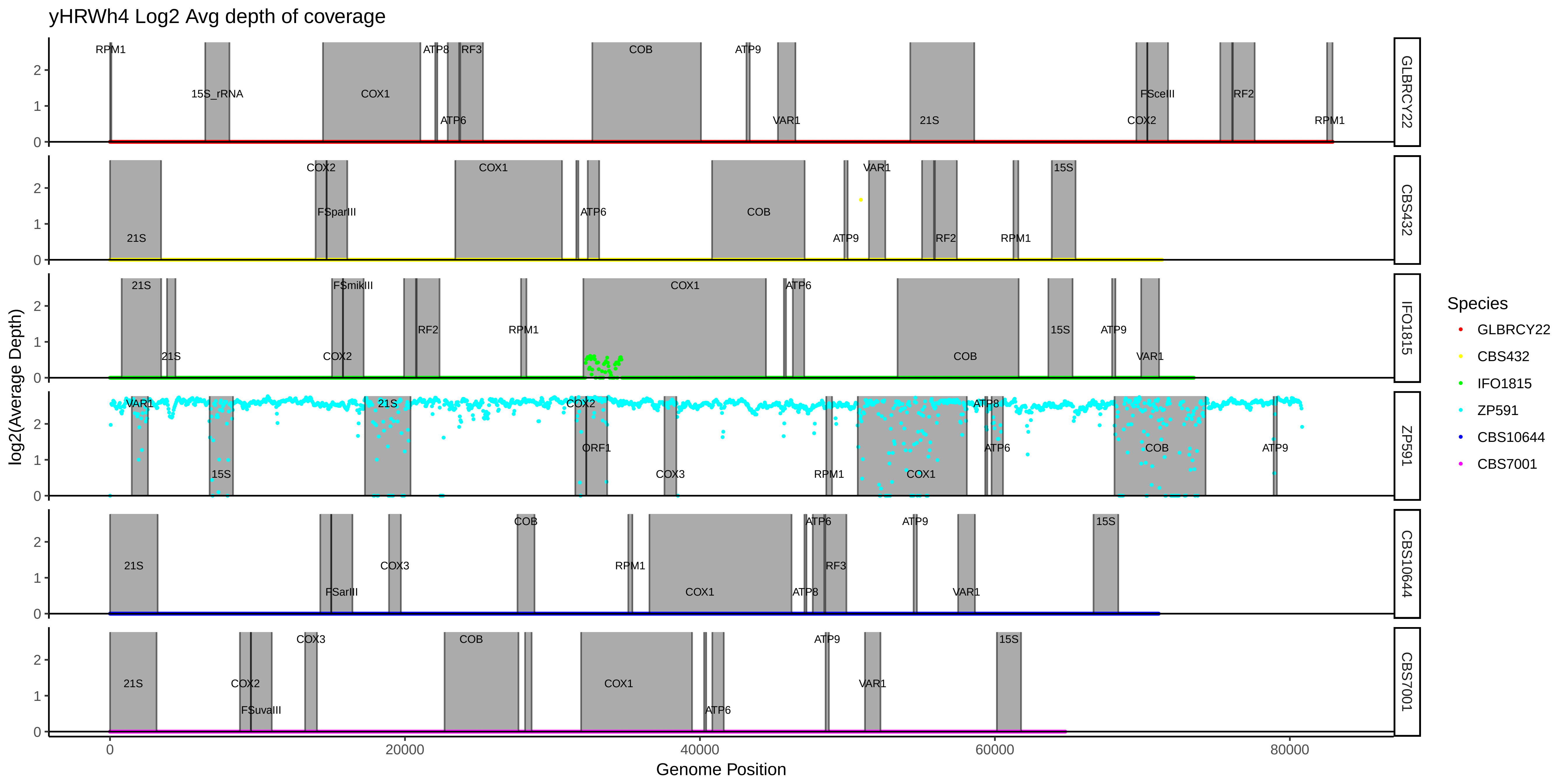

yHRWh4 Log2 Avg depth of coverage
log2(Average Depth)
Genome Position
0
20000
40000
60000
80000
GLBRCY22
CBS432
IFO1815
ZP591
CBS10644
CBS7001
Species
GLBRCY22
CBS432
IFO1815
ZP591
CBS10644
CBS7001
RPM1
15S_rRNA
COX1
ATP8
RF3
ATP6
COB
ATP9
VAR1
21S
FSceIII
COX2
RF2
21S
COX2
FSparIII
ATP6
ATP9
VAR1
RF2
RPM1
15S
FSmikIII
RF2
COX3
ORF1
CBS7001
FSarIII
FSuvaIII

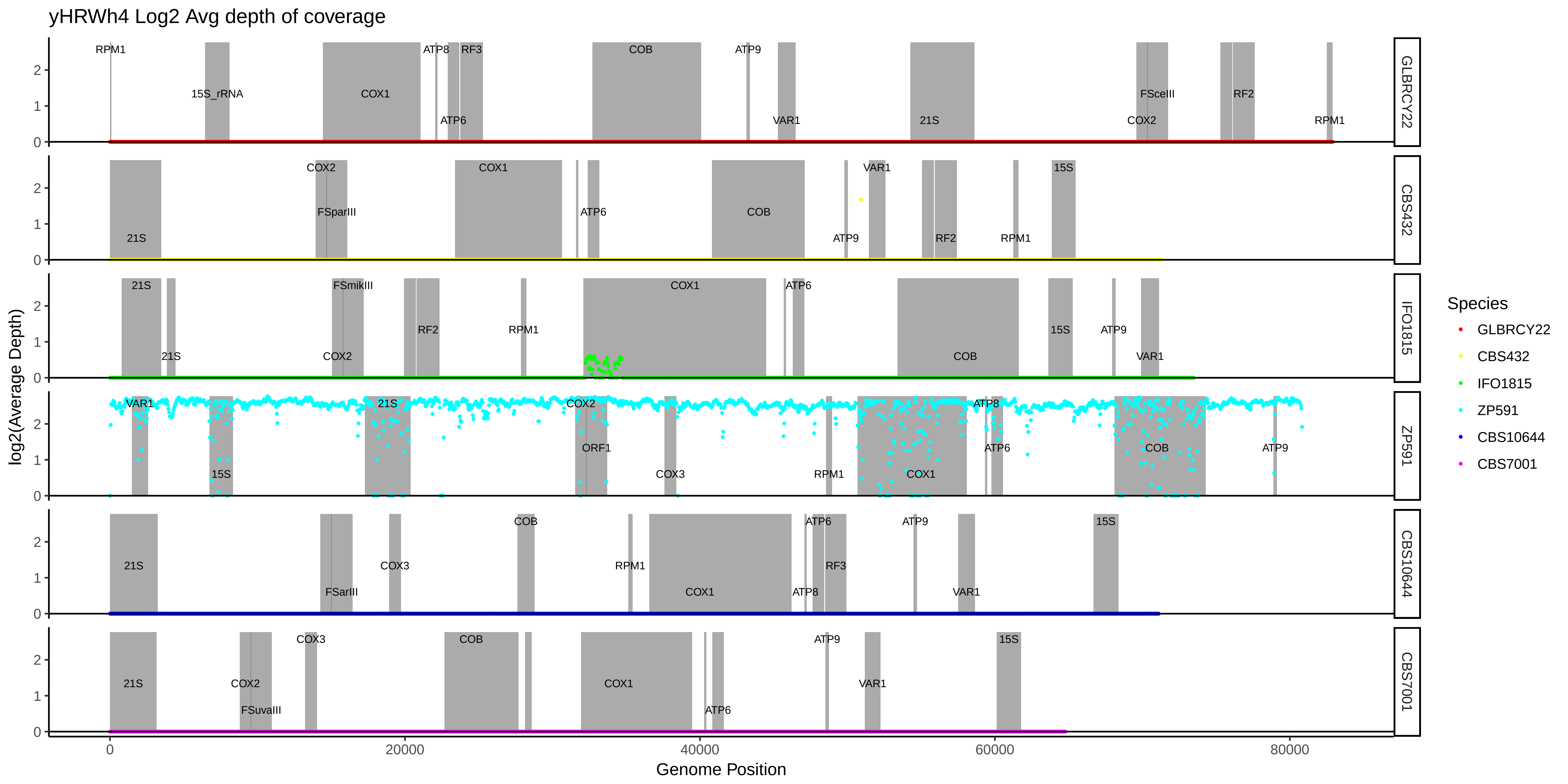

yHRWh4 Log2 Avg depth of coverage
log2(Average Depth)
Genome Position
0
20000
40000
60000
80000
GLBRCY22
CBS432
IFO1815
ZP591
CBS10644
CBS7001
Species
GLBRCY22
CBS432
IFO1815
ZP591
CBS10644
CBS7001
RPM1
15S_rRNA
COX1
ATP8
RF3
ATP6
COB
ATP9
VAR1
21S
FSceIII
COX2
RF2
21S
COX2
FSparIII
ATP6
ATP9
RF2
RPM1
15S
FSmikIII
RPM1
ORF1
COX3
FSarIII
ATP8
FSuvaIII

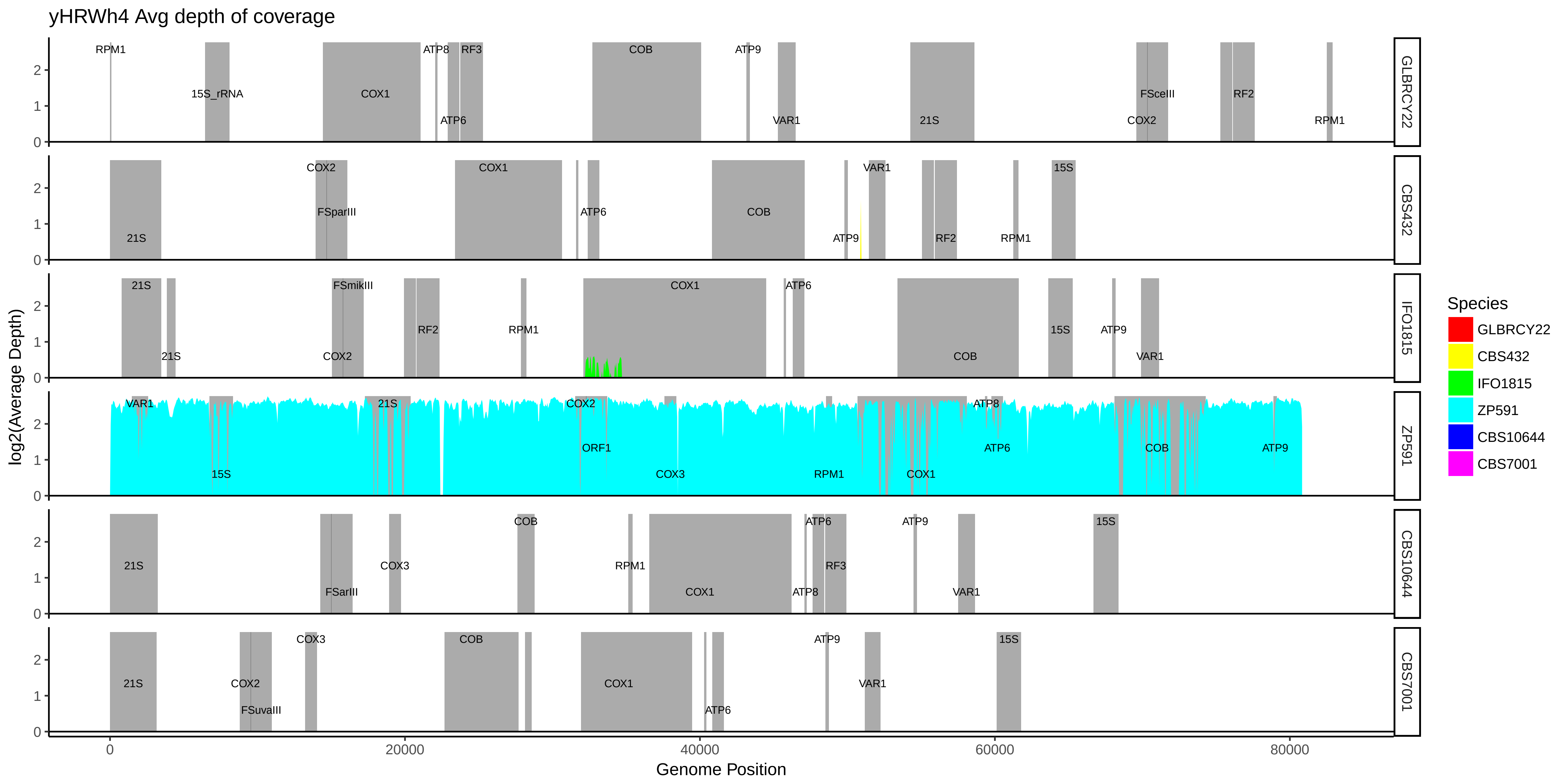

yHRWh4 Avg depth of coverage
log2(Average Depth)
Genome Position
GLBRCY22
CBS432
IFO1815
ZP591
CBS10644
CBS7001
Species
GLBRCY22
CBS432
IFO1815
ZP591
CBS10644
CBS7001
RPM1
15S_rRNA
COX1
ATP8
ATP6
RF3
COB
ATP9
VAR1
21S
FSceIII
COX2
RF2
RPM1
21S
COX2
FSparIII
COX1
ATP6
COB
ATP9
VAR1
RF2
RPM1
15S
21S
21S
FSmikIII
COX2
RF2
RPM1
COX1
ATP6
COB
15S
ATP9
VAR1
VAR1
15S
21S
COX2
ORF1
COX3
RPM1
COX1
ATP8
ATP6
COB
ATP9
21S
FSarIII
COX3
COB
RPM1
COX1
ATP8
ATP6
RF3
ATP9
VAR1
15S
21S
COX2
FSuvaIII
COX3
COB
COX1
ATP6
ATP9
VAR1
15S
0
20000
40000
60000
80000

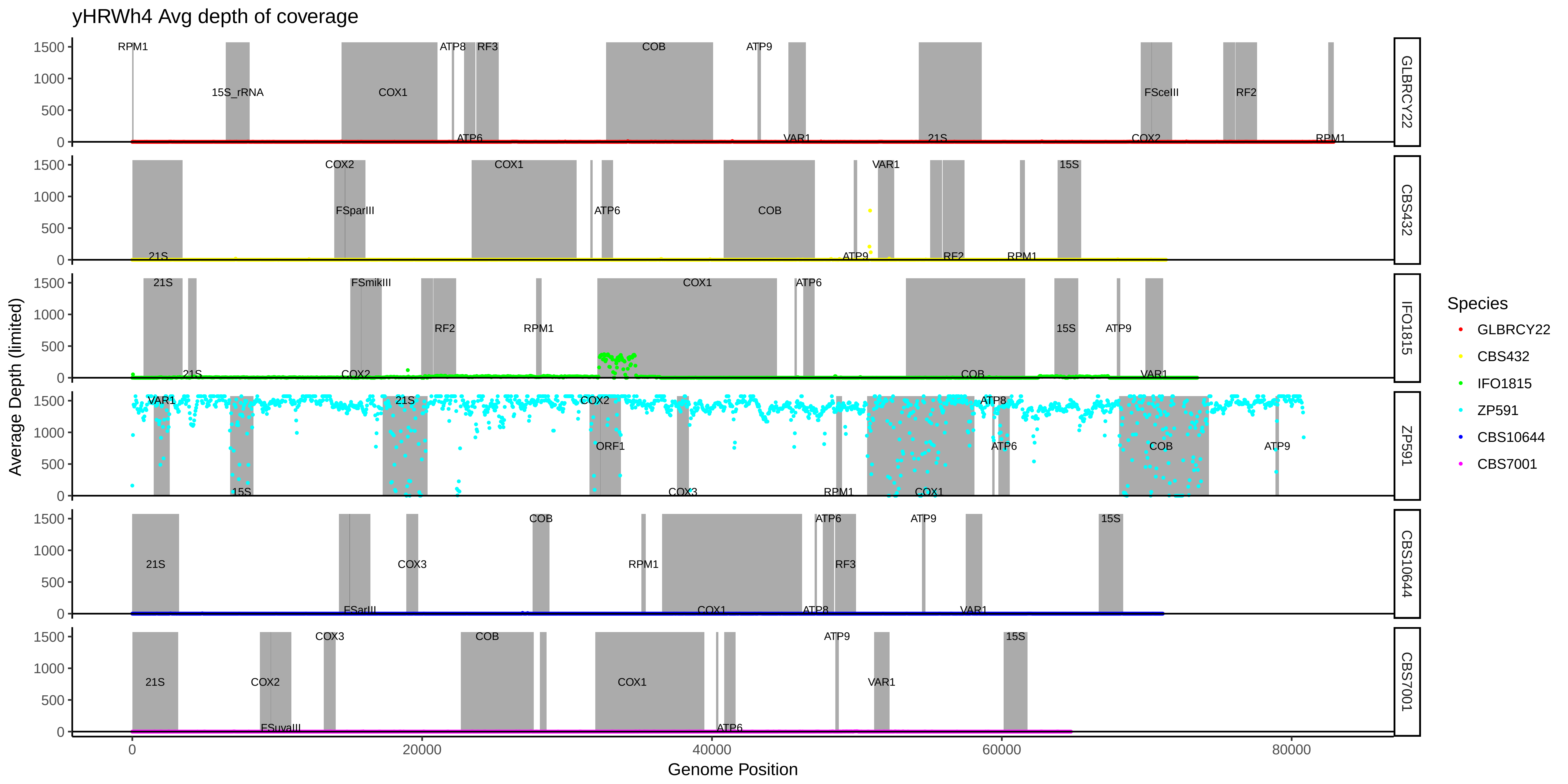

yHRWh4 Avg depth of coverage
Average Depth (limited)
Genome Position
GLBRCY22
CBS432
IFO1815
ZP591
CBS10644
CBS7001
Species
GLBRCY22
CBS432
IFO1815
ZP591
CBS10644
CBS7001
RPM1
15S_rRNA
COX1
ATP8
RF3
ATP6
COB
ATP9
VAR1
21S
FSceIII
COX2
RF2
COX2
FSparIII
ATP6
FSmikIII
ORF1
COX3
FSarIII
FSuvaIII
15S
0
500
1000
1500
0
20000
40000
60000
80000

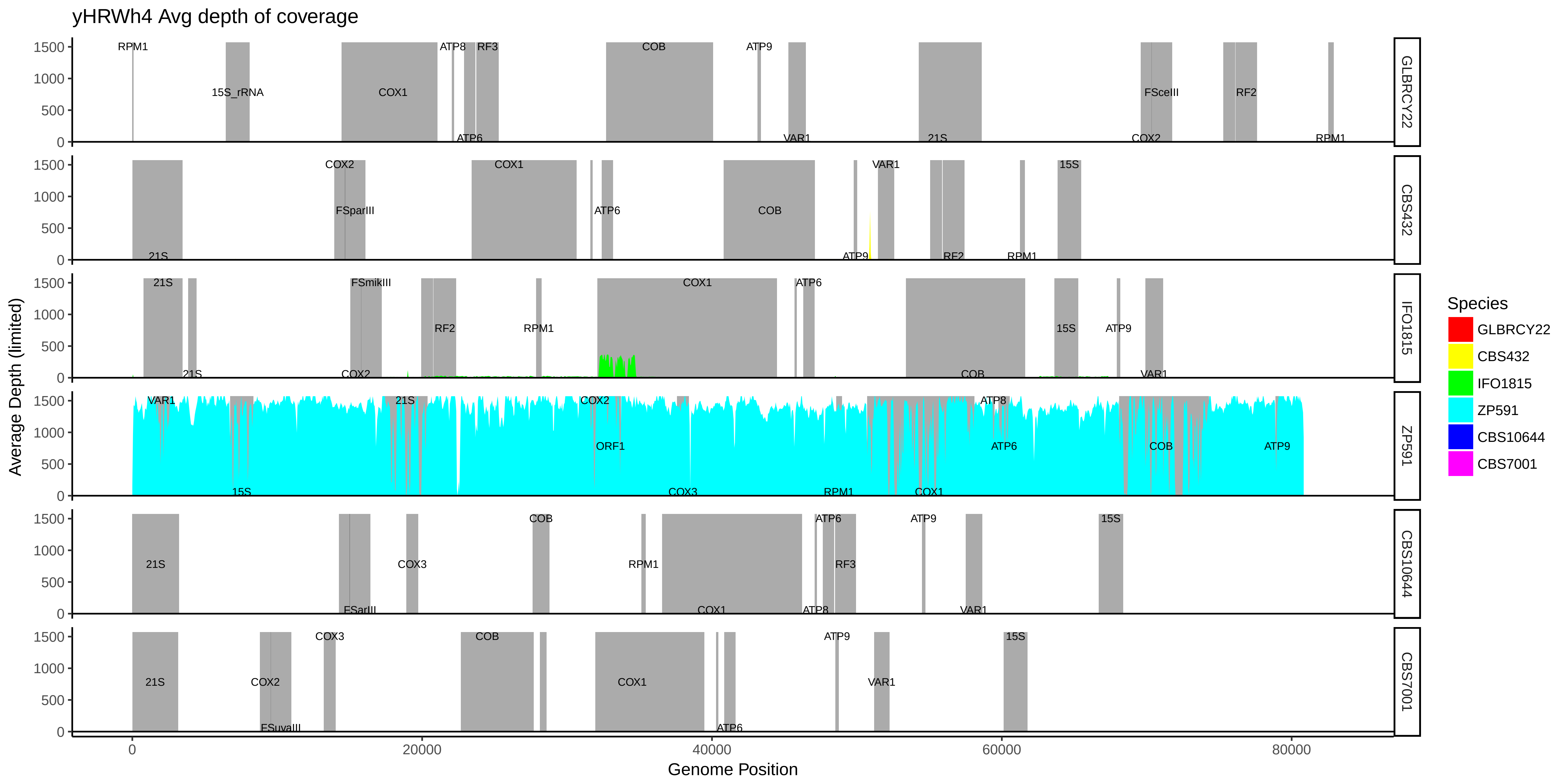

yHRWh4 Avg depth of coverage
Average Depth (limited)
Genome Position
Species
GLBRCY22
CBS432
IFO1815
ZP591
CBS10644
CBS7001

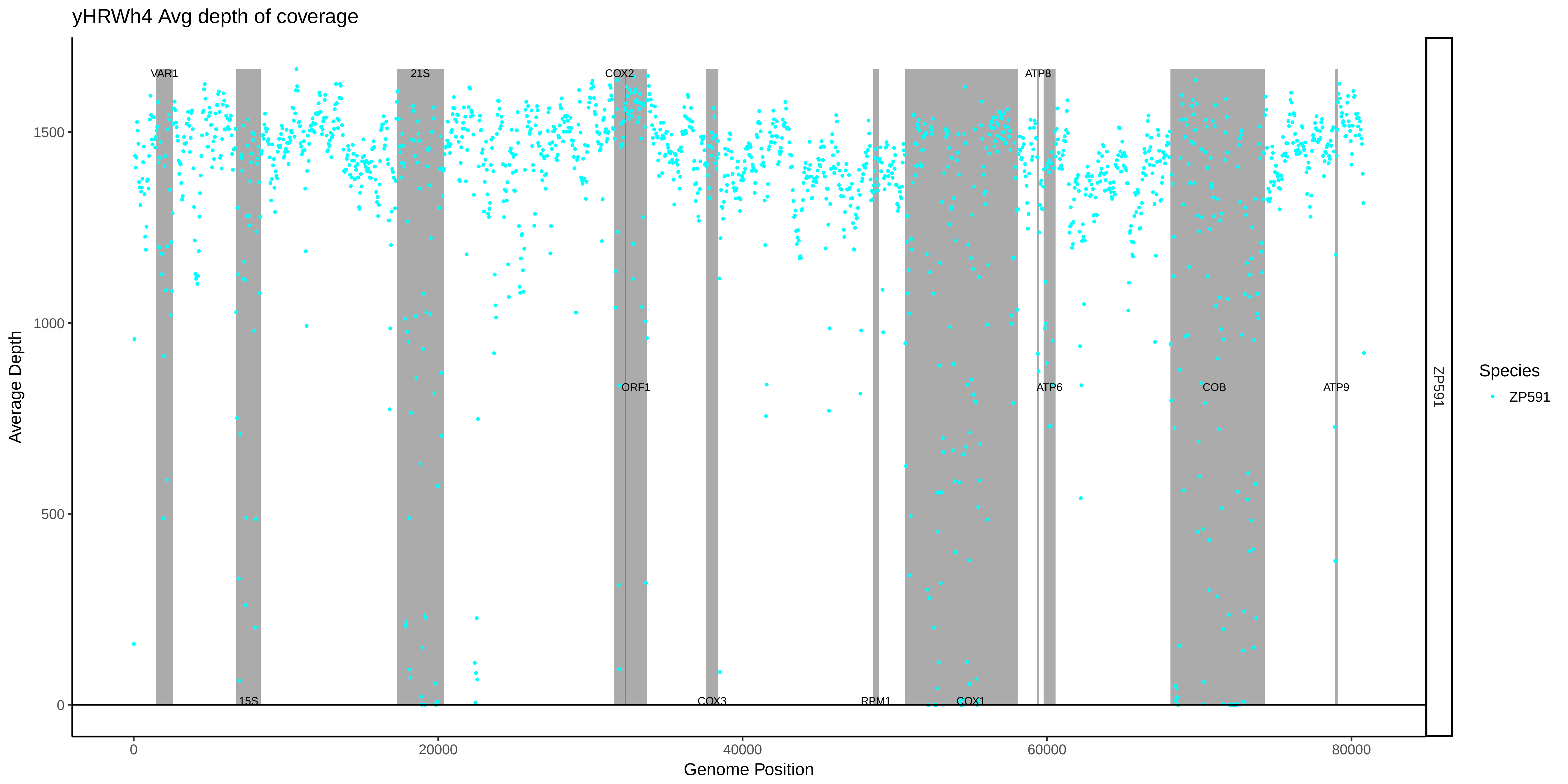

yHRWh4 Avg depth of coverage
Average Depth
Genome Position
VAR1
21S
COX2
ATP8
ORF1
ATP6
COB
ATP9
15S
COX3
RPM1
COX1
ZP591
Species
ZP591
0
500
1000
1500
0
20000
40000
60000
80000

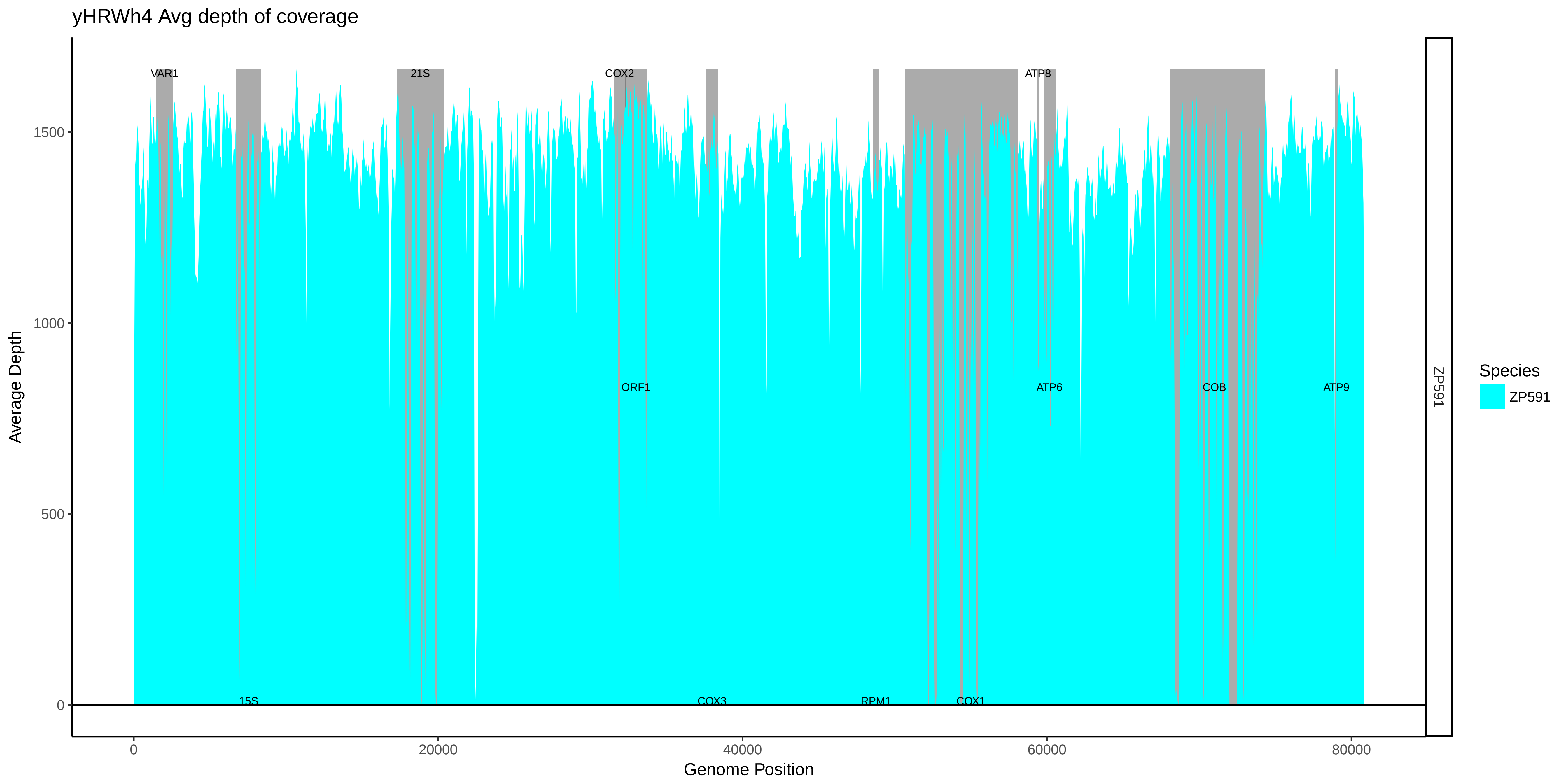

yHRWh4 Avg depth of coverage
Average Depth
Genome Position
0
500
1000
1500
0
20000
40000
60000
80000
VAR1
21S
COX2
ATP8
ORF1
ATP6
COB
ATP9
15S
COX3
RPM1
COX1
ZP591
Species
ZP591

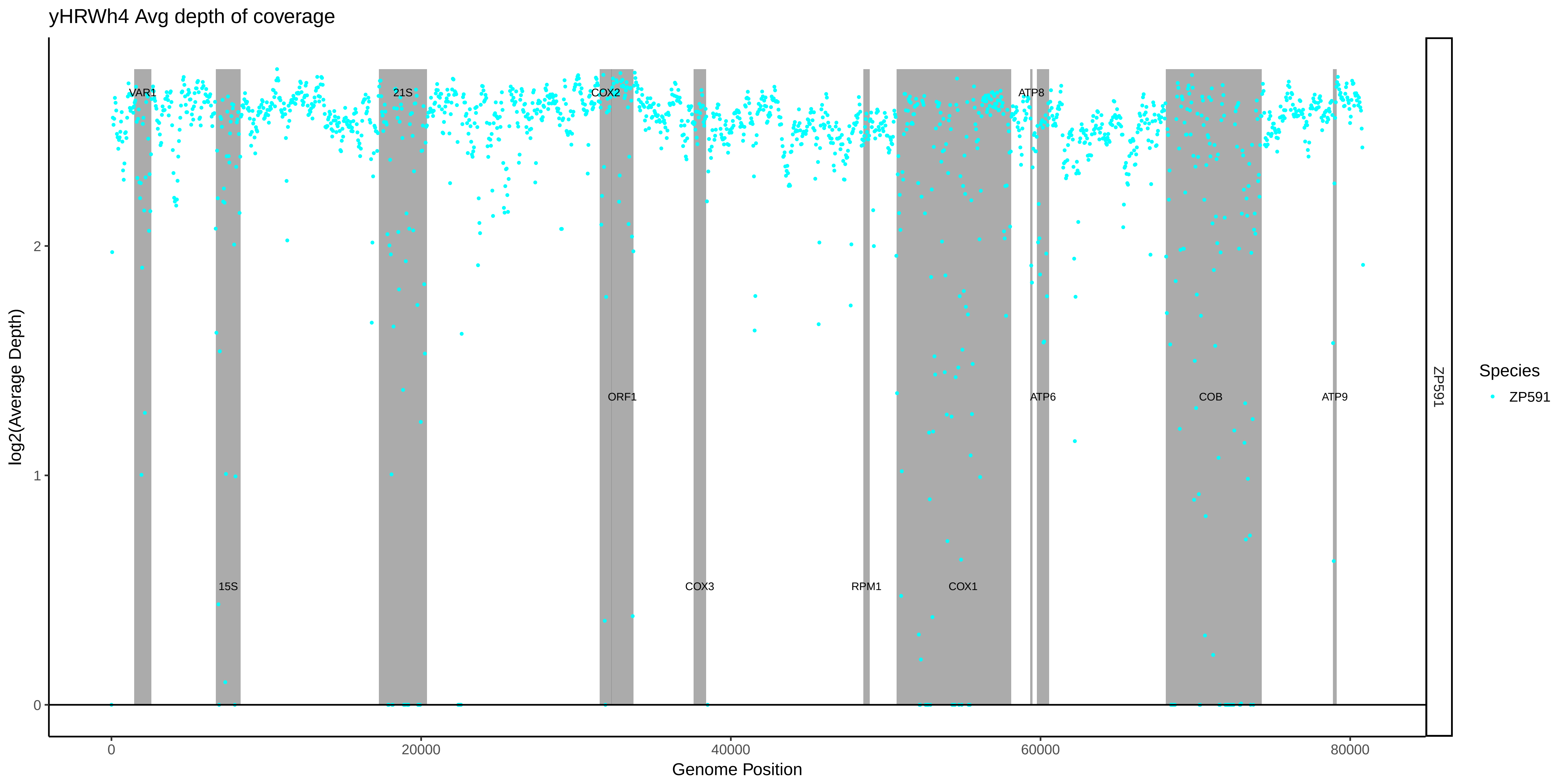

yHRWh4 Avg depth of coverage
log2(Average Depth)
Genome Position
0
1
2
0
20000
40000
60000
80000
VAR1
21S
COX2
ATP8
ORF1
ATP6
COB
ATP9
15S
COX3
RPM1
COX1
ZP591
Species
ZP591

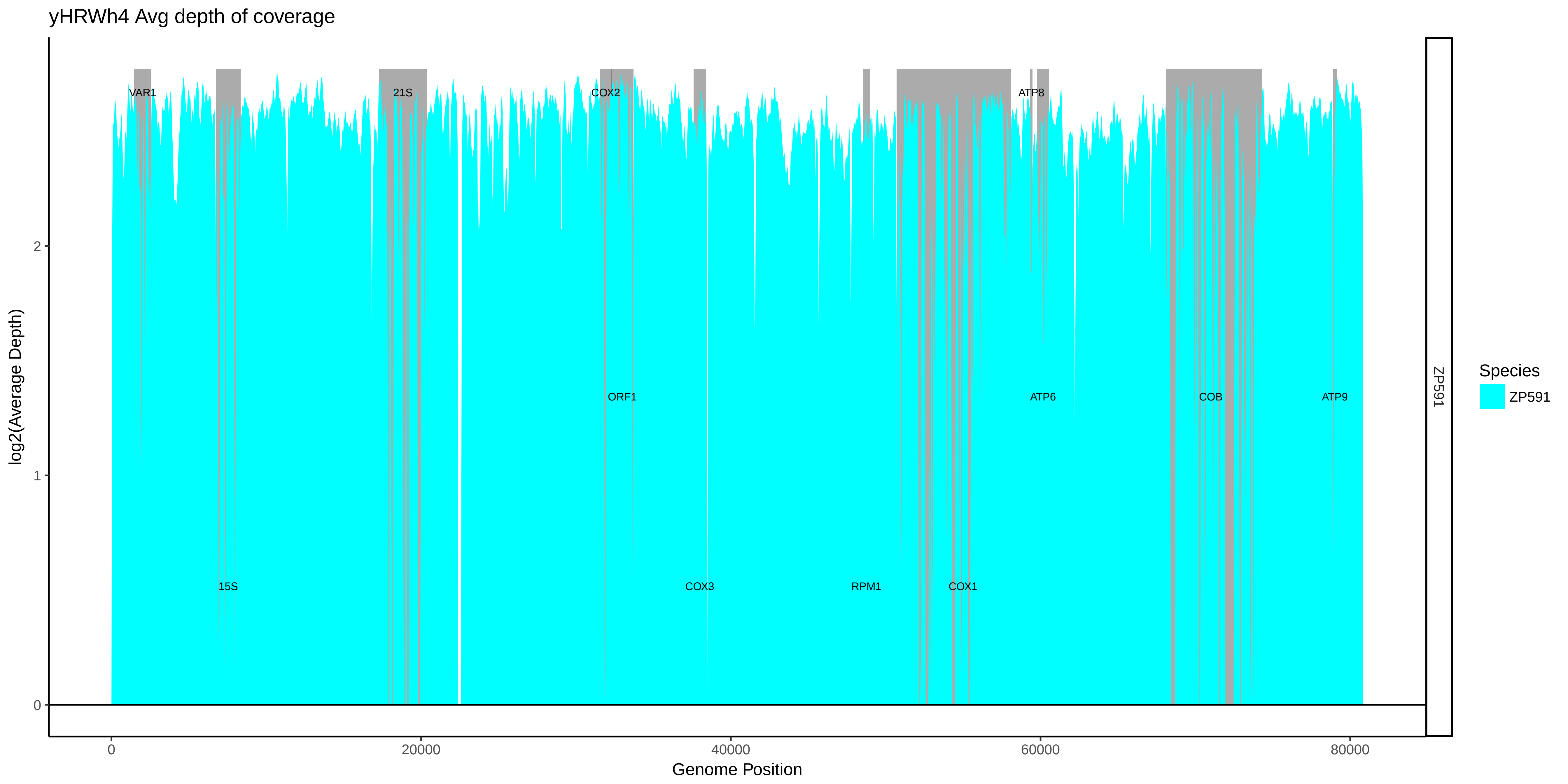
yHRWh4 Avg depth of coverage
log2(Average Depth)
Genome Position
0
1
2
0
20000
40000
60000
80000
VAR1
21S
COX2
ATP8
ORF1
ATP6
COB
ATP9
15S
COX3
RPM1
COX1
ZP591
Species
ZP591

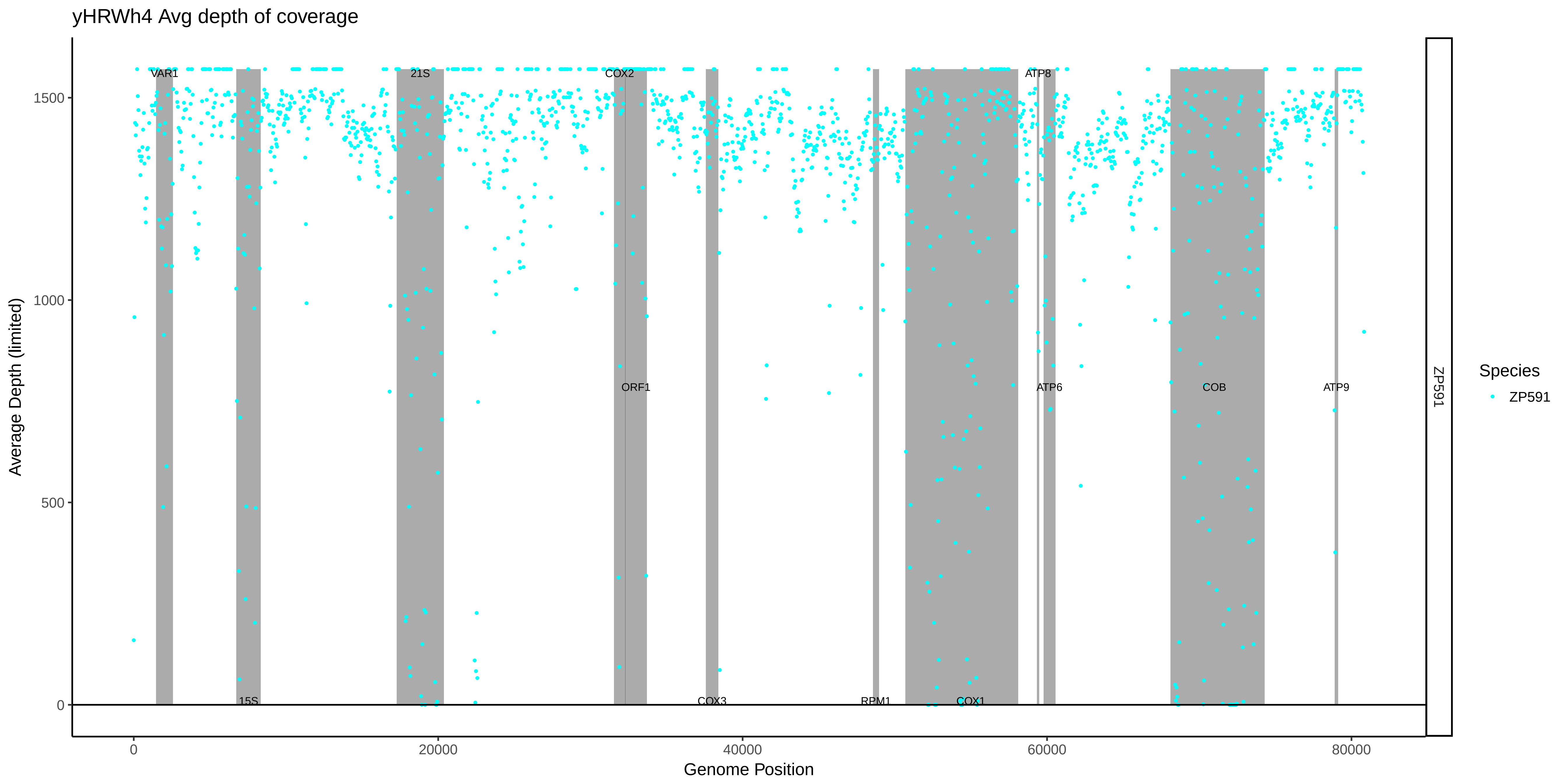

yHRWh4 Avg depth of coverage
Average Depth (limited)
Genome Position
1500
1000
500
0
0
20000
40000
60000
80000
VAR1
15S
21S
COX2
ORF1
COX3
RPM1
COX1
ATP8
ATP6
COB
ATP9
ZP591
Species
ZP591

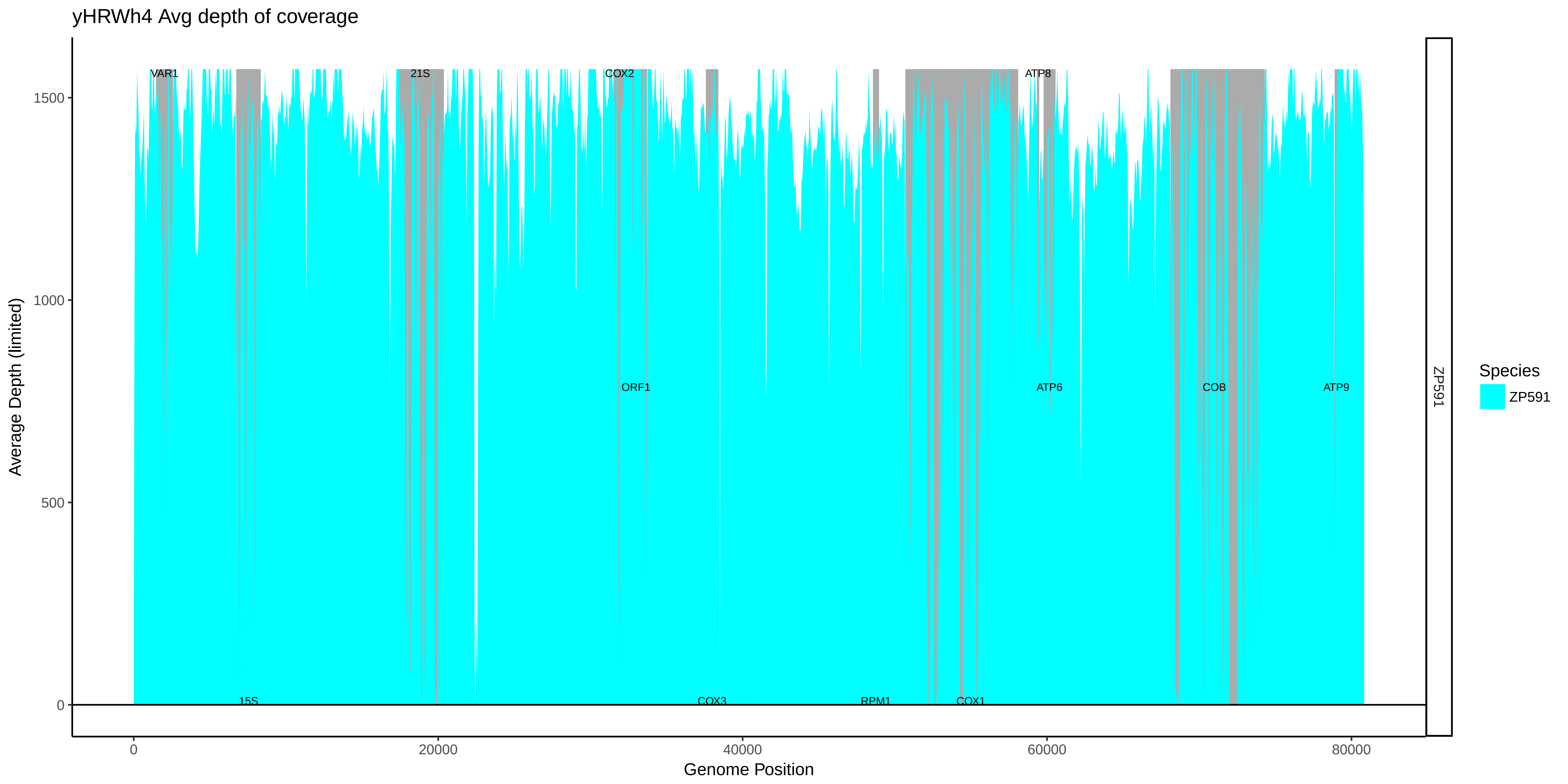

yHRWh4 Avg depth of coverage
VAR1
21S
COX2
ATP8
ORF1
ATP6
COB
ATP9
15S
COX3
RPM1
COX1
Average Depth (limited)
1500
1000
500
0
Genome Position
0
20000
40000
60000
80000
ZP591
Species
ZP591
