# Supplementary material for: Synthetic hybrids of six yeast species
Source: Nat Commun. 2020 Apr 29;11:2085. doi: 10.1038/s41467-020-15559-4 (PMC7190663; doi:10.1038/s41467-020-15559-4)
Supplement: Supplementary file 15 — Source Data [file 41467_2020_15559_MOESM15_ESM.zip › Source Data 3.zip!/yHRWh10_mitoSppIDerDepthPlot-d.pdf]

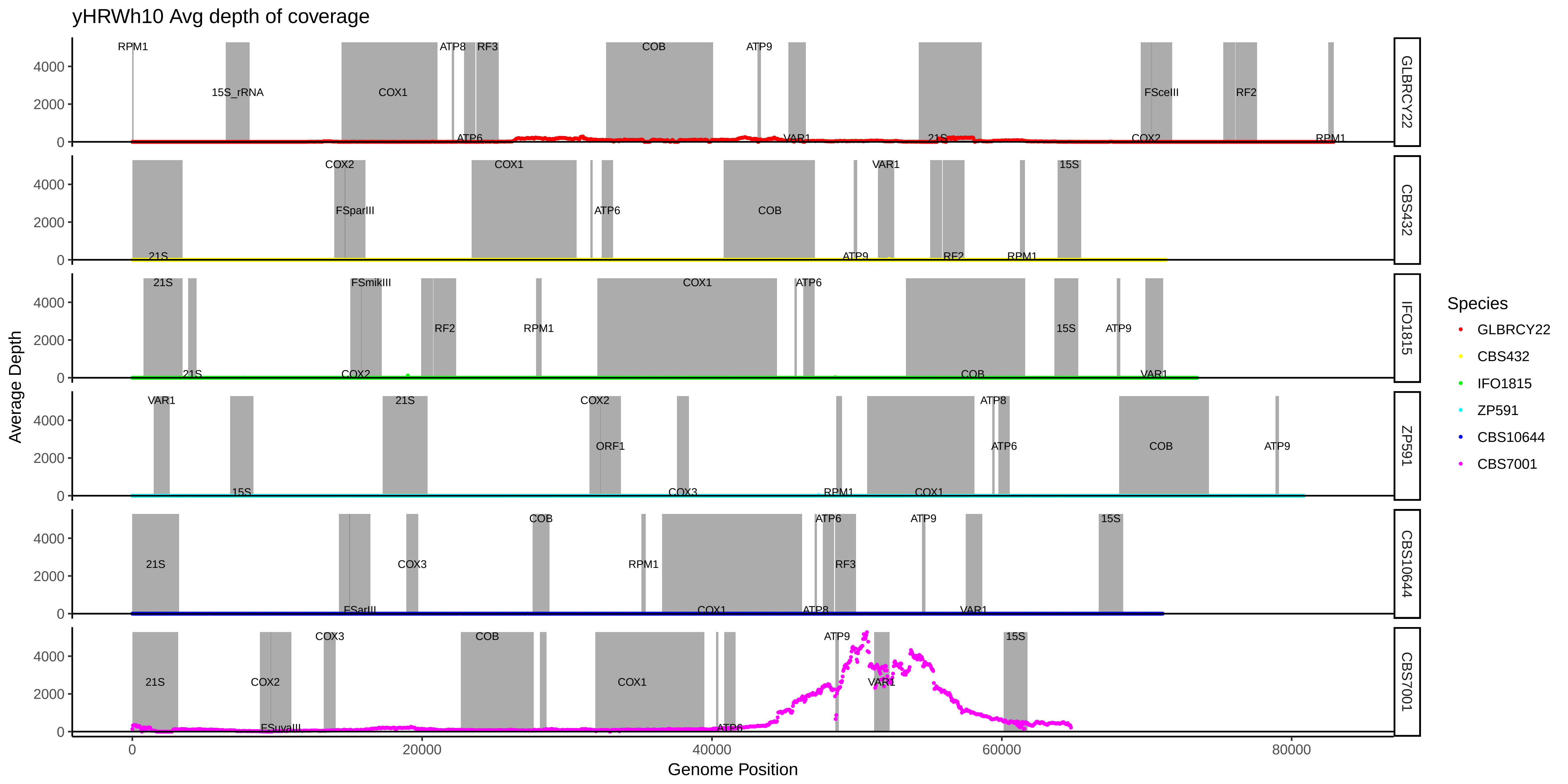

yHRWh10 Avg depth of coverage
Average Depth
Genome Position
0
20000
40000
60000
80000
2000
4000
GLBRCY22
CBS432
IFO1815
ZP591
CBS10644
CBS7001
Species
GLBRCY22
CBS432
IFO1815
ZP591
CBS10644
CBS7001
RPM1
15S_rRNA
COX1
ATP8
RF3
ATP6
COB
ATP9
VAR1
21S
FSceIII
COX2
RF2
COX2
FSparIII
ATP6
15S
COX3
COX1
FSmikIII
RPM1
ORF1
ATP6
FSarIII
FSuvaIII

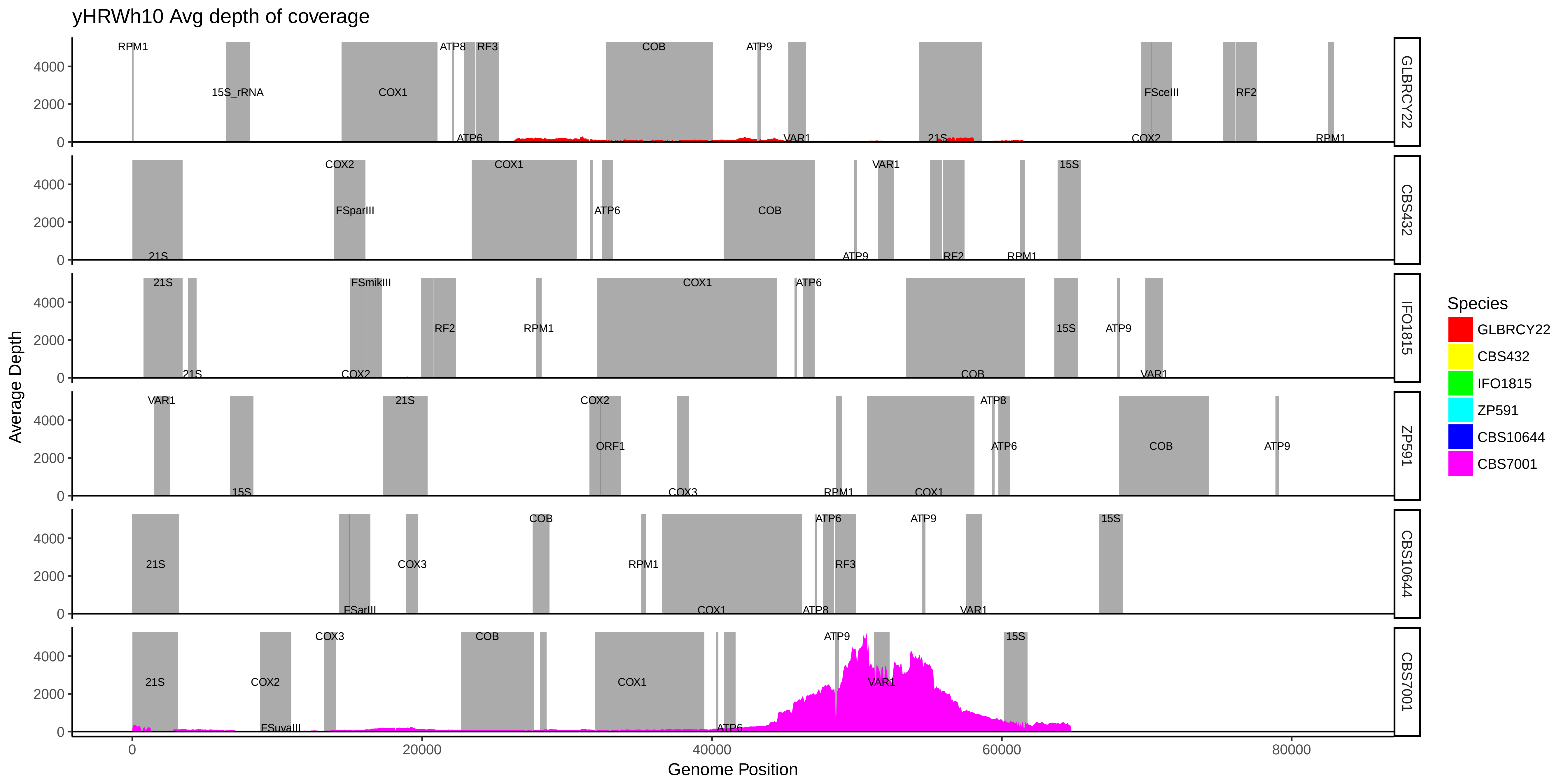
yHRWh10 Avg depth of coverage
Average Depth
Genome Position
0
2000
4000
0
20000
40000
60000
80000
GLBRCY22
CBS432
IFO1815
ZP591
CBS10644
CBS7001
Species
GLBRCY22
CBS432
IFO1815
ZP591
CBS10644
CBS7001
RPM1
15S_rRNA
COX1
ATP8
RF3
ATP6
COB
ATP9
VAR1
21S
FSceIII
COX2
RF2
RPM1
21S
COX2
FSparIII
COX1
ATP6
COB
ATP9
VAR1
RF2
RPM1
15S
21S
21S
FSmikIII
COX2
RF2
RPM1
COX1
ATP6
COB
15S
ATP9
VAR1
VAR1
15S
21S
COX2
ORF1
COX3
RPM1
COX1
ATP8
ATP6
COB
ATP9
21S
FSarIII
COX3
COB
RPM1
COX1
ATP6
ATP8
RF3
ATP9
VAR1
15S
21S
COX2
FSuvaIII
COX3
COB
COX1
ATP6
ATP9
VAR1
15S

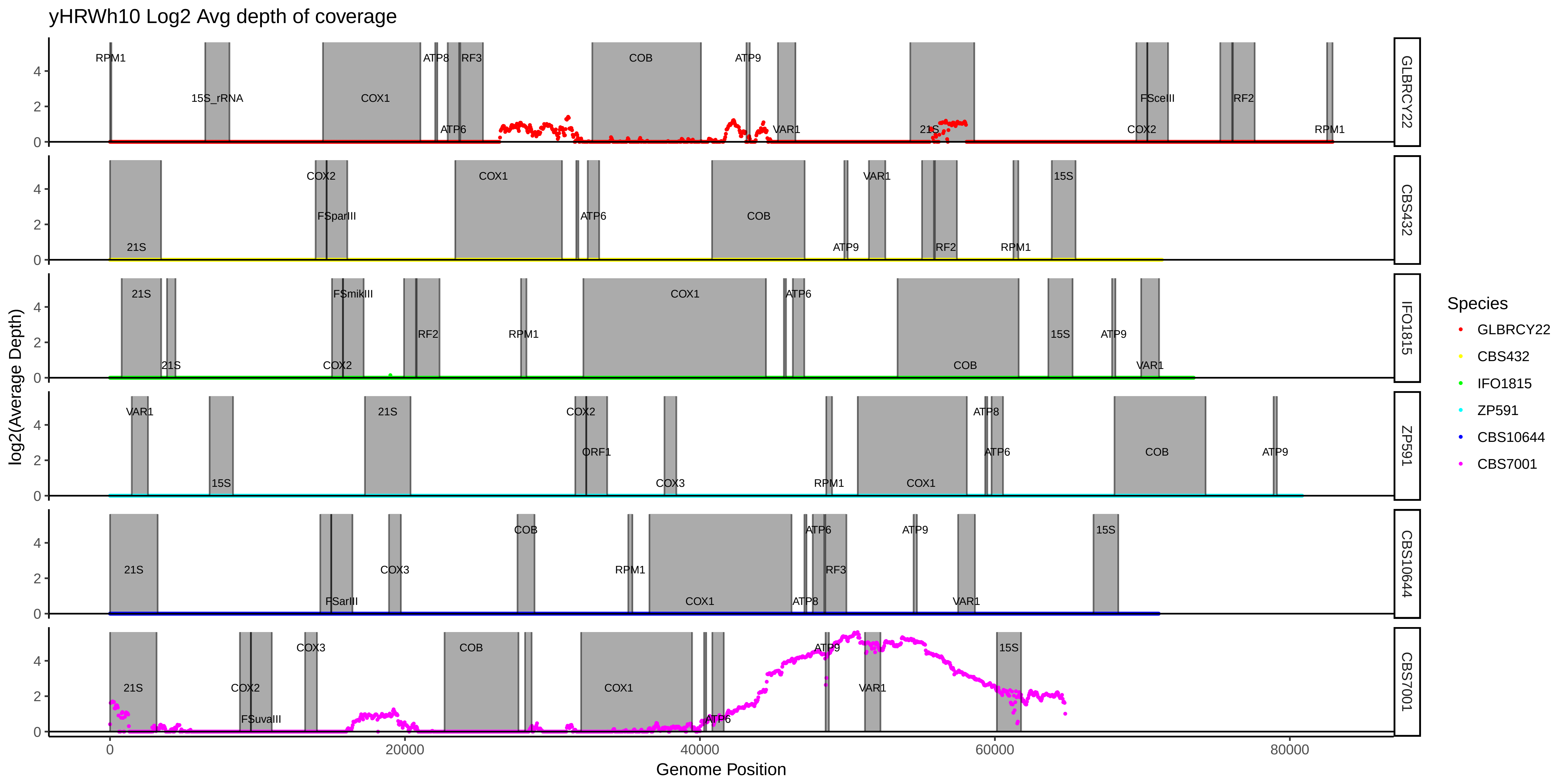

yHRWh10 Log2 Avg depth of coverage
log2(Average Depth)
Genome Position
0
20000
40000
60000
80000
GLBRCY22
CBS432
IFO1815
ZP591
CBS10644
CBS7001
Species
GLBRCY22
CBS432
IFO1815
ZP591
CBS10644
CBS7001
RPM1
15S_rRNA
COX1
ATP8
RF3
ATP6
COB
ATP9
VAR1
21S
FSceIII
COX2
RF2
21S
COX2
FSparIII
ATP6
RPM1
15S
FSmikIII
ORF1
COX3
FSarIII
FSuvaIII

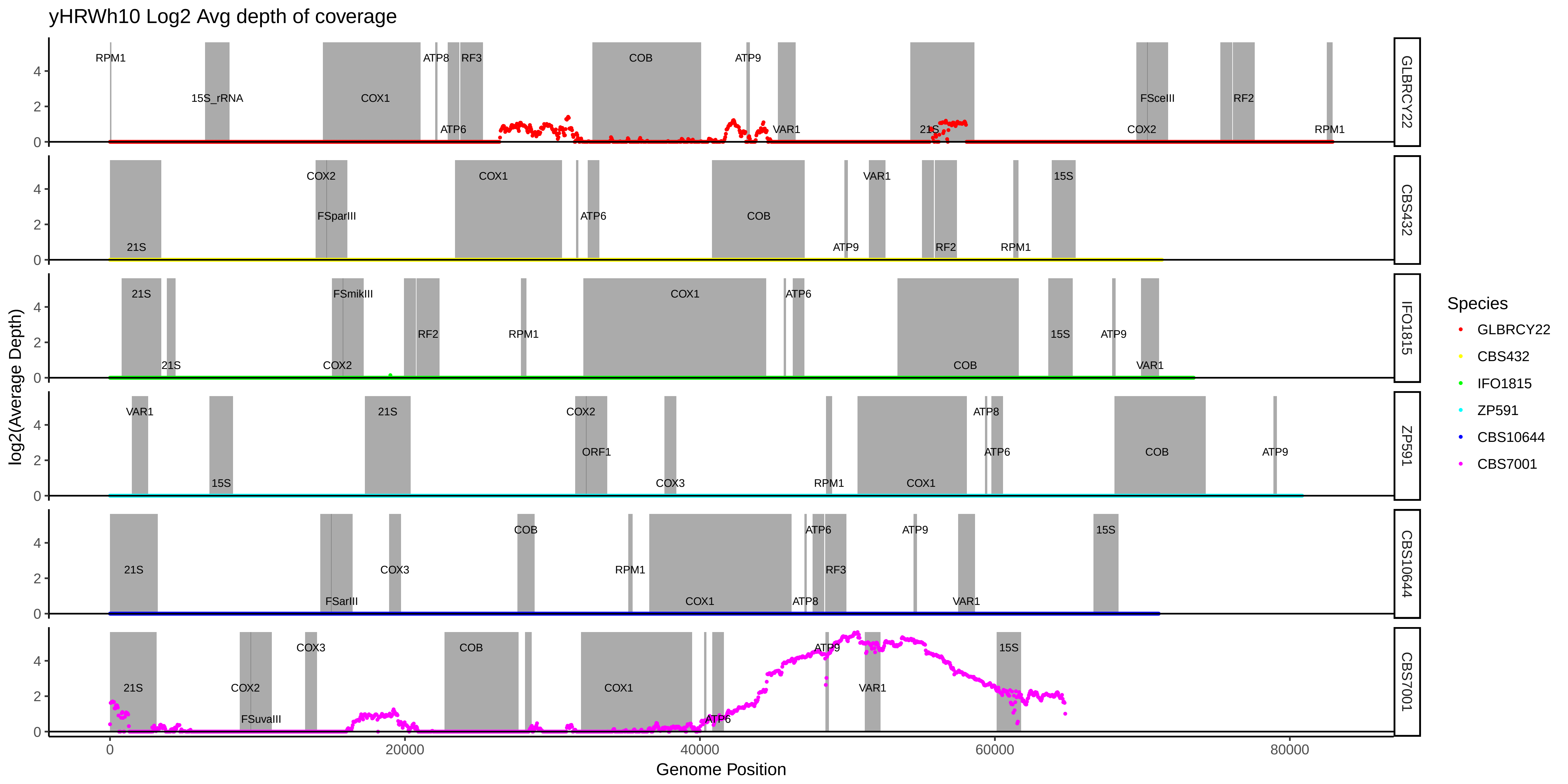

yHRWh10 Log2 Avg depth of coverage
log2(Average Depth)
Genome Position
0
20000
40000
60000
80000
GLBRCY22
RPM1
15S_rRNA
COX1
ATP8
RF3
ATP6
COB
ATP9
VAR1
21S
FSceIII
RF2
COX2
RPM1
CBS432
21S
COX2
FSparIII
COX1
ATP6
COB
ATP9
VAR1
RF2
RPM1
15S
IFO1815
21S
21S
FSmikIII
COX2
RF2
RPM1
COX1
ATP6
COB
15S
ATP9
VAR1
ZP591
VAR1
15S
21S
COX2
ORF1
COX3
RPM1
COX1
ATP8
ATP6
COB
ATP9
CBS10644
21S
FSarIII
COX3
COB
RPM1
COX1
ATP8
ATP6
RF3
ATP9
VAR1
15S
CBS7001
21S
COX2
FSuvaIII
COX3
COB
COX1
ATP6
ATP9
VAR1
15S
Species
GLBRCY22
CBS432
IFO1815
ZP591
CBS10644
CBS7001

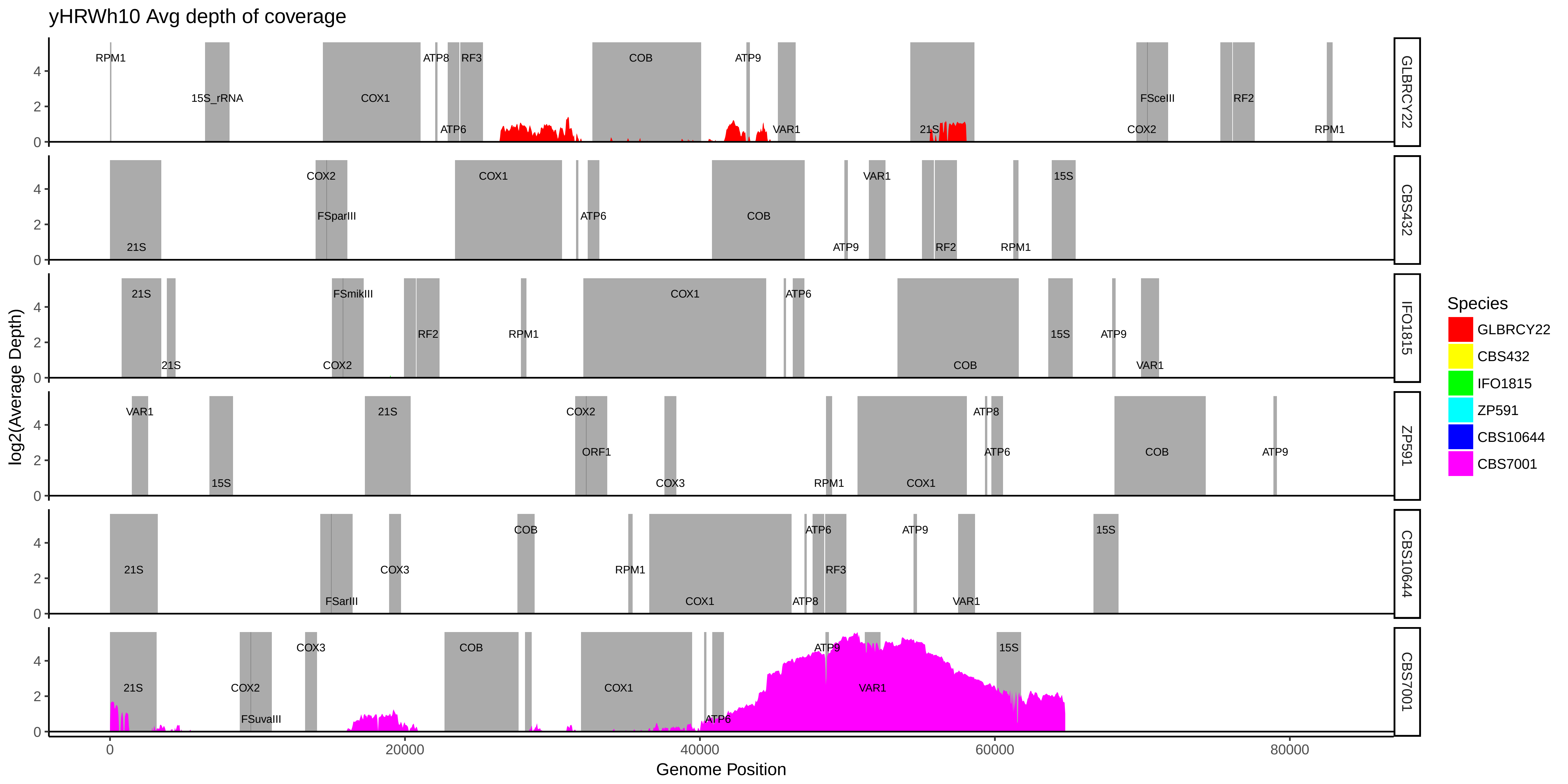

yHRWh10 Avg depth of coverage
log2(Average Depth)
Genome Position
Species
GLBRCY22
CBS432
IFO1815
ZP591
CBS10644
CBS7001
RPM1
15S_rRNA
COX1
ATP8
ATP6
RF3
COB
ATP9
VAR1
21S
COX2
FSceIII
RF2
FSparIII
15S
FSmikIII
ORF1
COX3
FSarIII
FSuvaIII
0
20000
40000
60000
80000

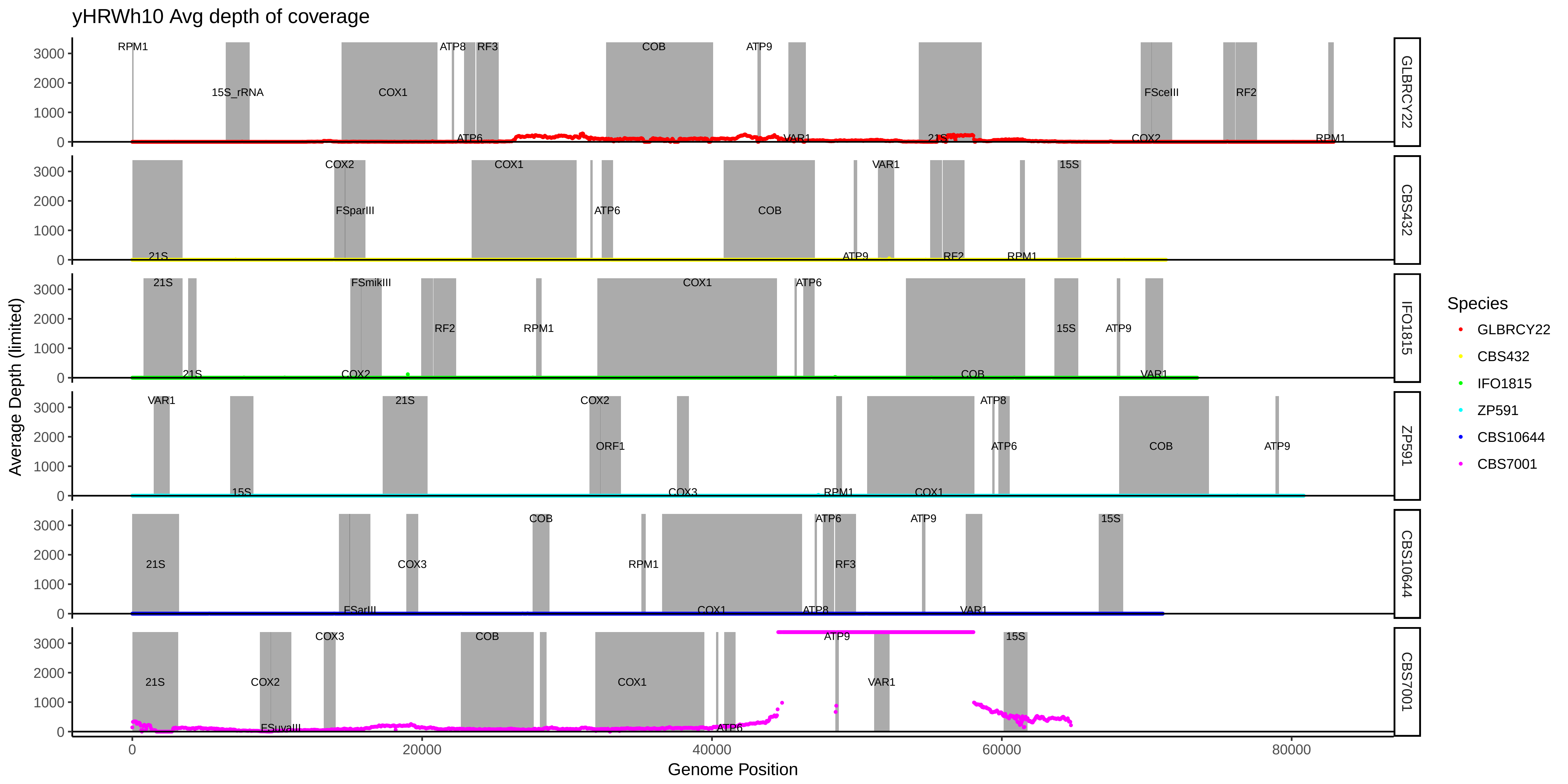
yHRWh10 Avg depth of coverage
Average Depth (limited)
Genome Position
Species
GLBRCY22
CBS432
IFO1815
ZP591
CBS10644
CBS7001

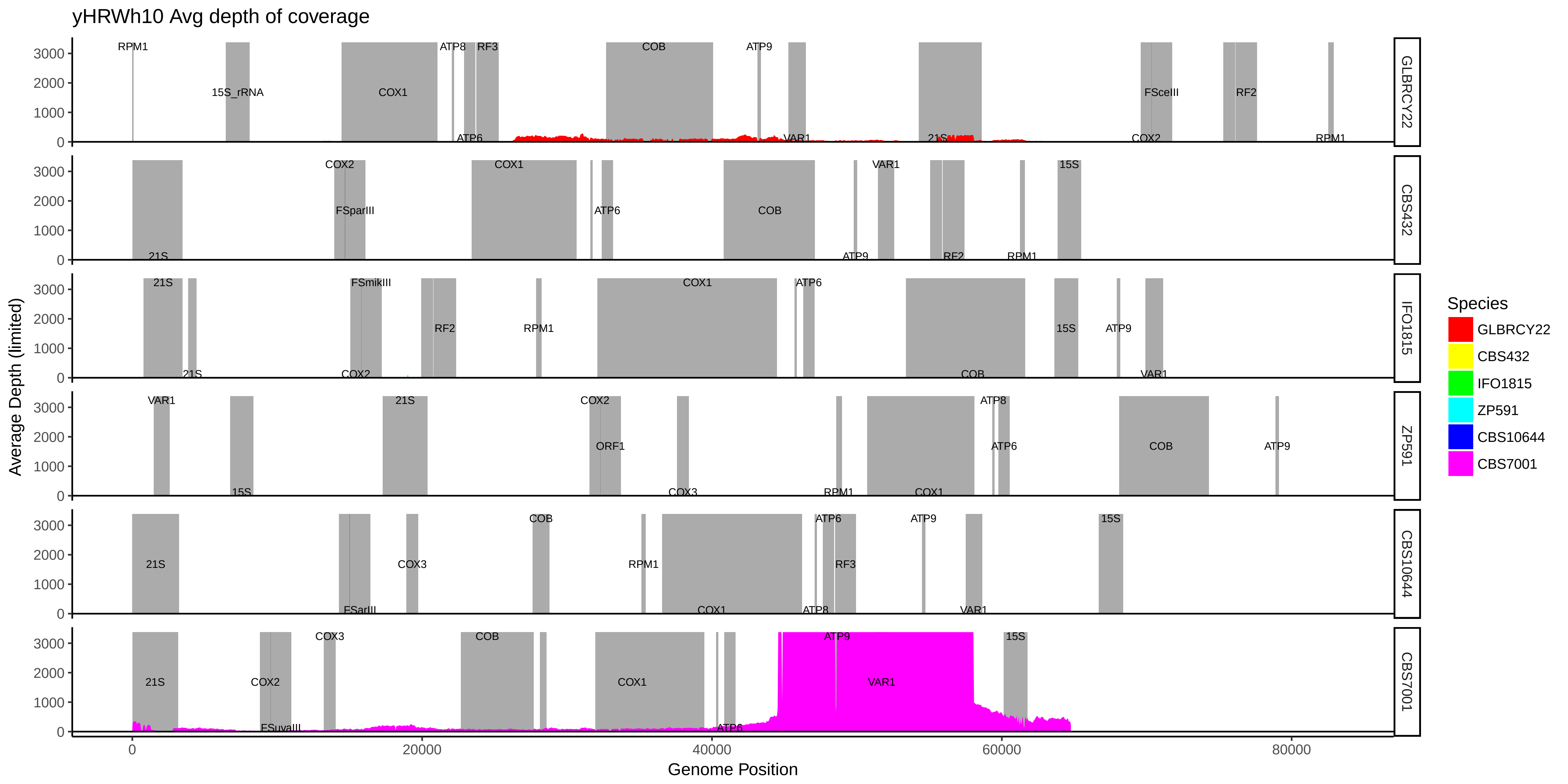
yHRWh10 Avg depth of coverage
Average Depth (limited)
Genome Position
GLBRCY22
CBS432
IFO1815
ZP591
CBS10644
CBS7001
Species
RPM1
15S_rRNA
COX1
ATP8
RF3
ATP6
COB
ATP9
VAR1
21S
FSceIII
COX2
RF2
FSparIII
15S
FSmikIII
ORF1
COX3
FSarIII
FSuvaIII
0
1000
2000
3000
20000
40000
60000
80000

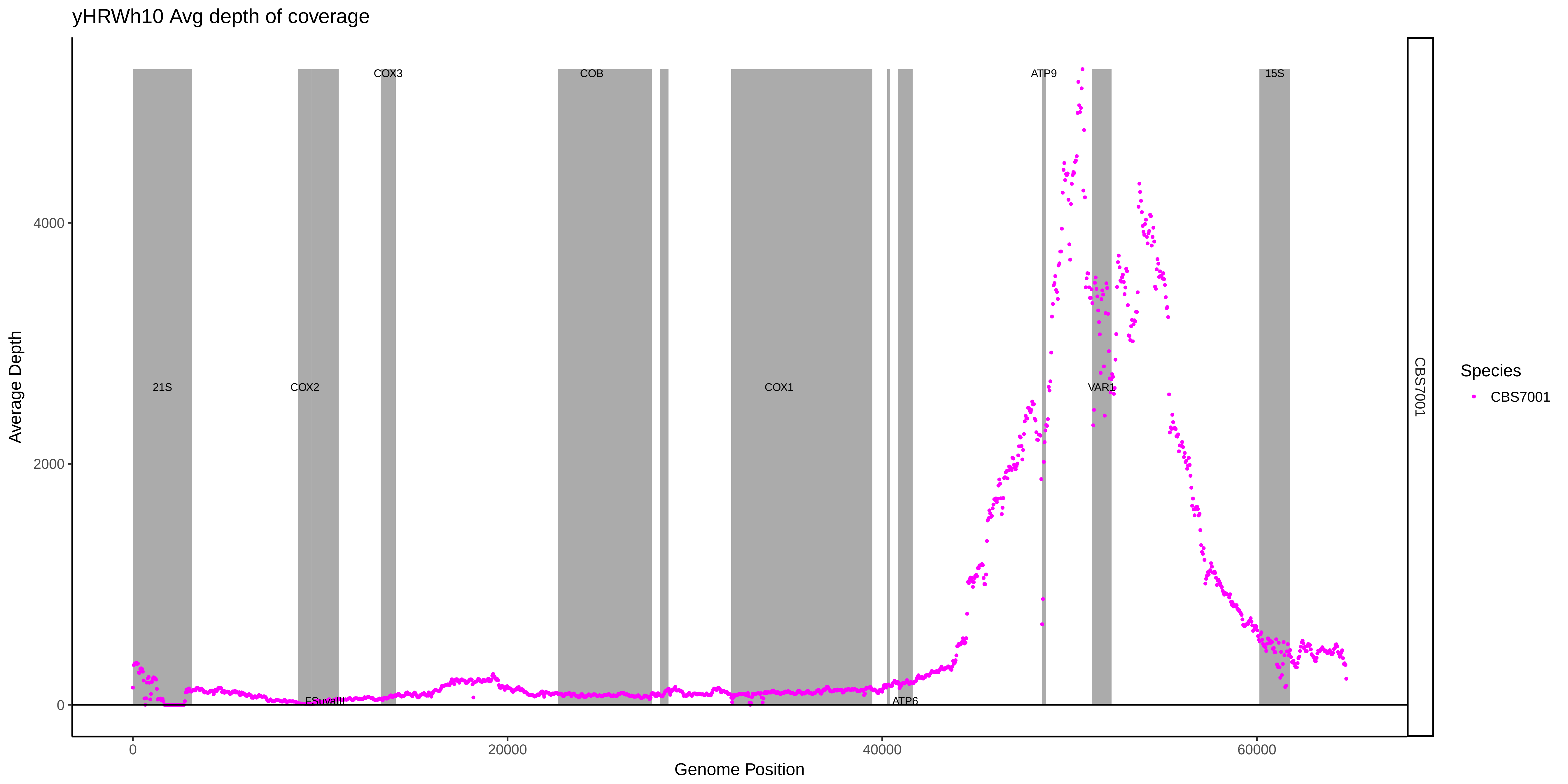
yHRWh10 Avg depth of coverage
Average Depth
Genome Position
4000
2000
0
0
20000
40000
60000
21S
COX2
COX3
COB
COX1
ATP9
VAR1
15S
ESuvalII
ATP6
CBS7001
Species
CBS7001

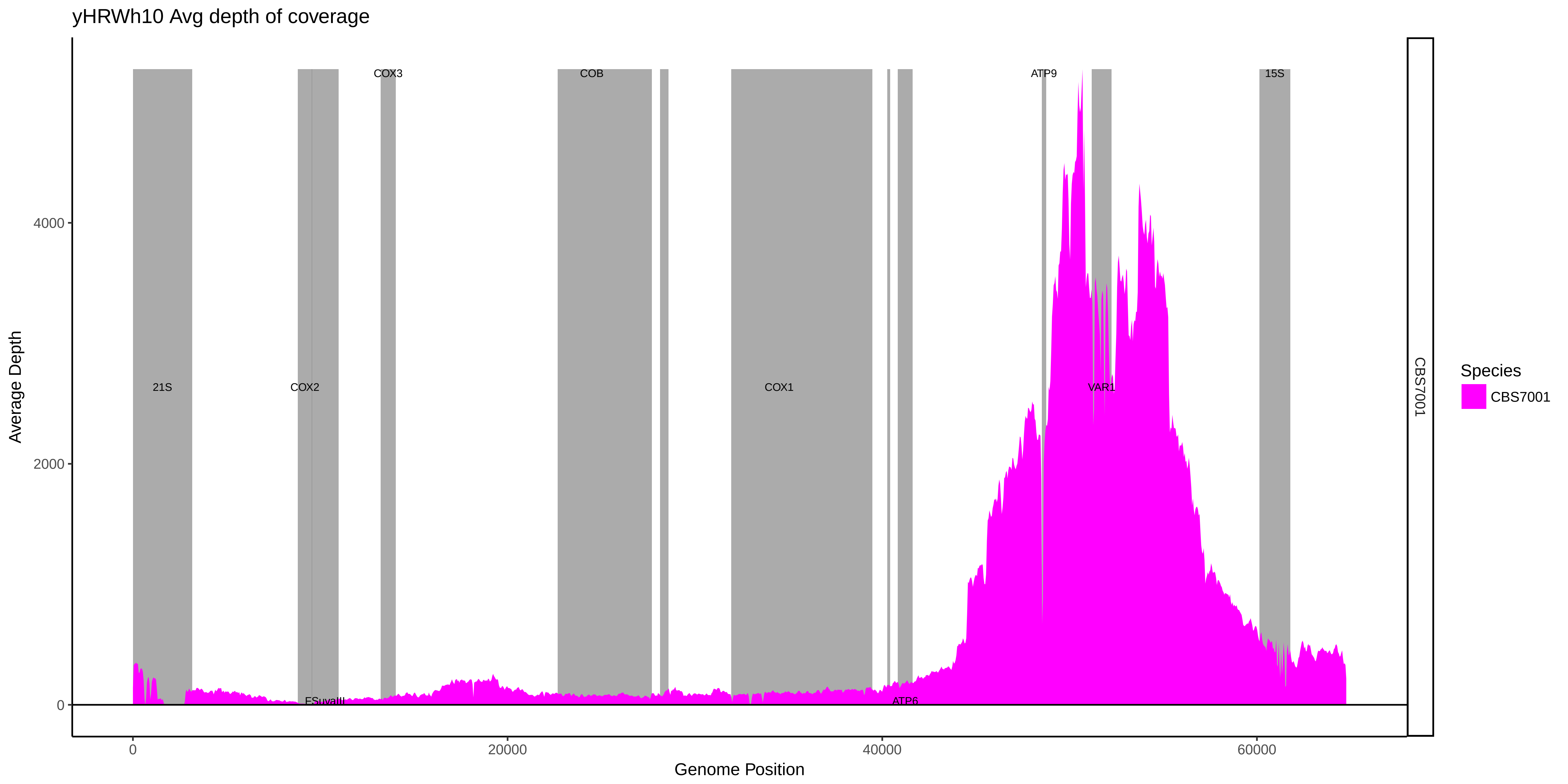

yHRWh10 Avg depth of coverage
21S
COX2
COX3
COB
COX1
ATP9
VAR1
15S
ESuvaIII
ATP6
CBS7001
Species
CBS7001
Average Depth
Genome Position
0
2000
4000
0
20000
40000
60000

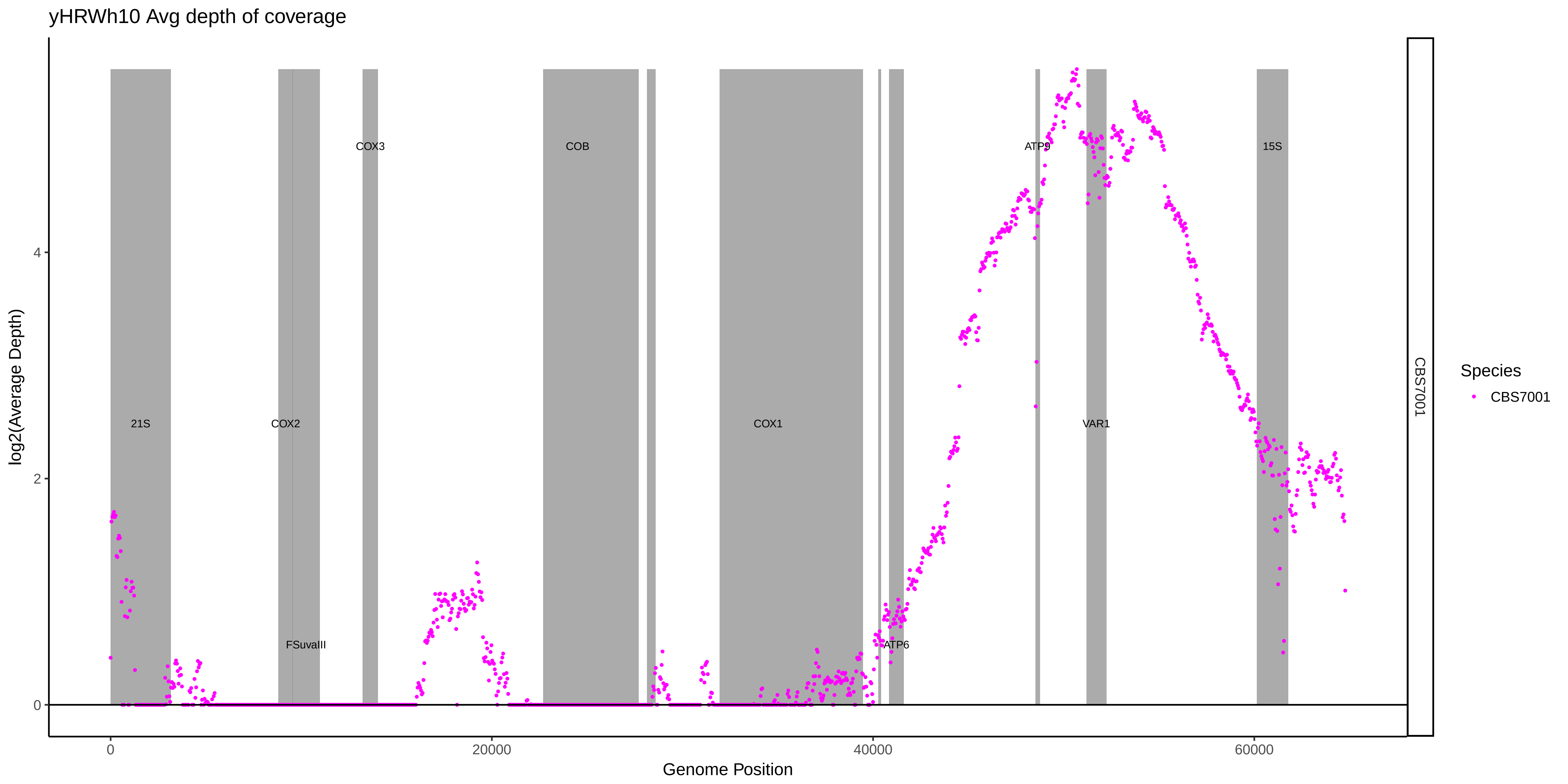

yHRWh10 Avg depth of coverage
21S
COX2
FSuvaIII
COX3
COB
COX1
ATP6
ATP9
VAR1
15S
log2(Average Depth)
0
2
4
Genome Position
0
20000
40000
60000
CBS7001
Species
CBS7001

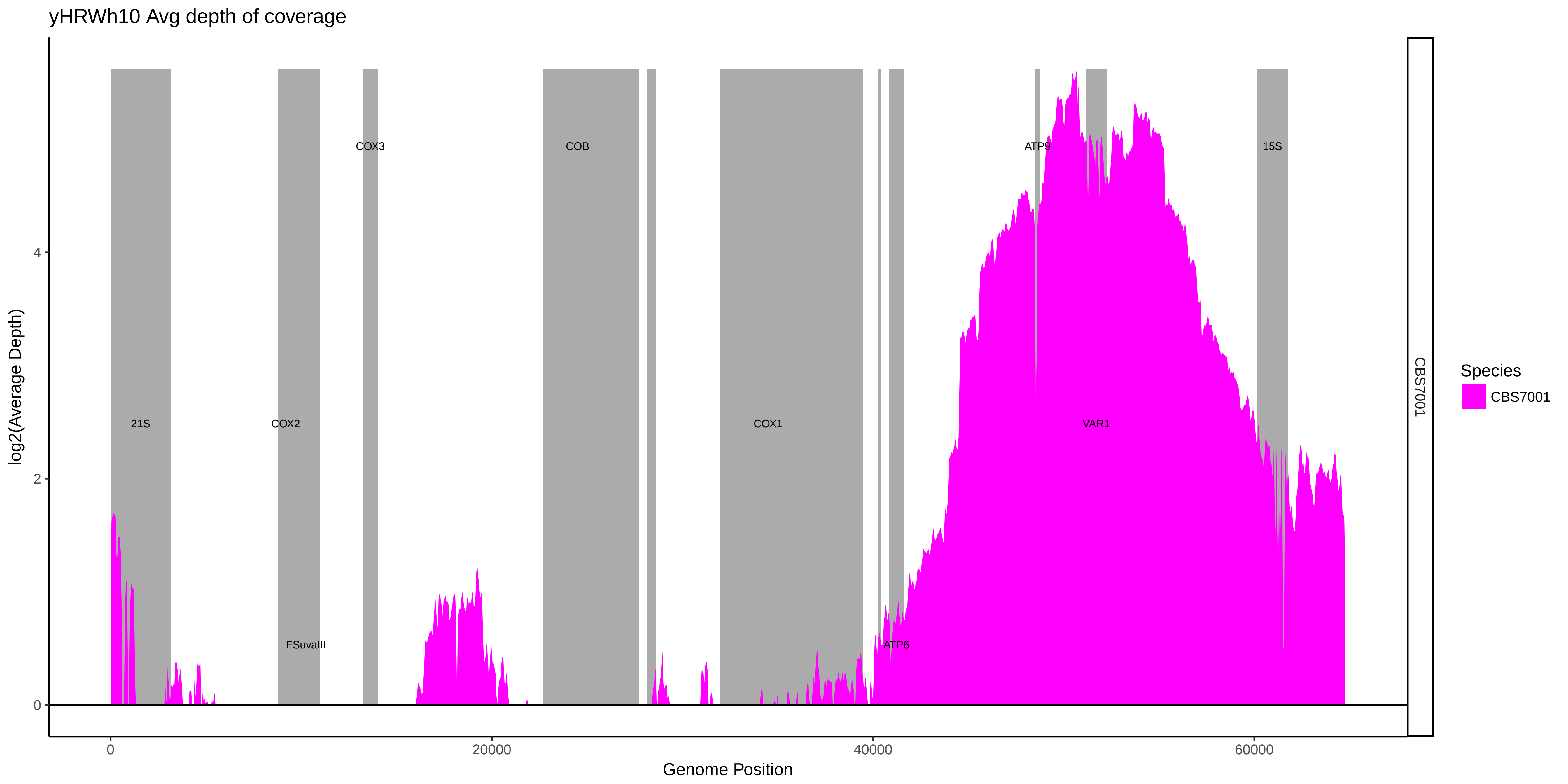
yHRWh10 Avg depth of coverage
log2(Average Depth)
Genome Position
0
2
4
0
20000
40000
60000
21S
COX2
FSuvaIII
COX3
COB
COX1
ATP6
ATP9
VAR1
15S
CBS7001
Species
CBS7001

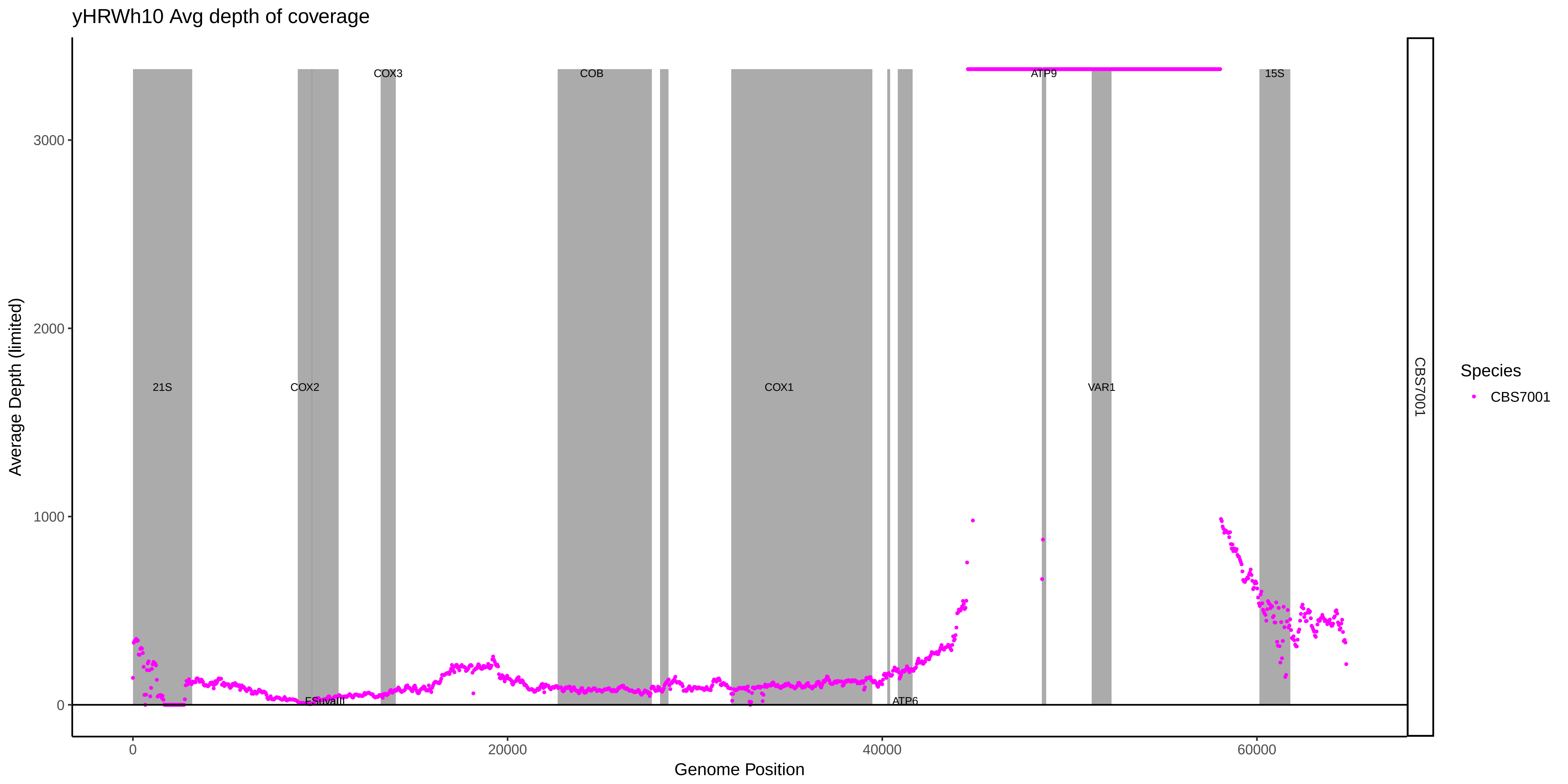

yHRWh10 Avg depth of coverage
21S
COX2
ESuvalII
COX3
COB
COX1
ATP6
ATP9
VAR1
15S
CBS7001
Average Depth (limited)
Genome Position
0
1000
2000
3000
20000
40000
60000
Species
CBS7001

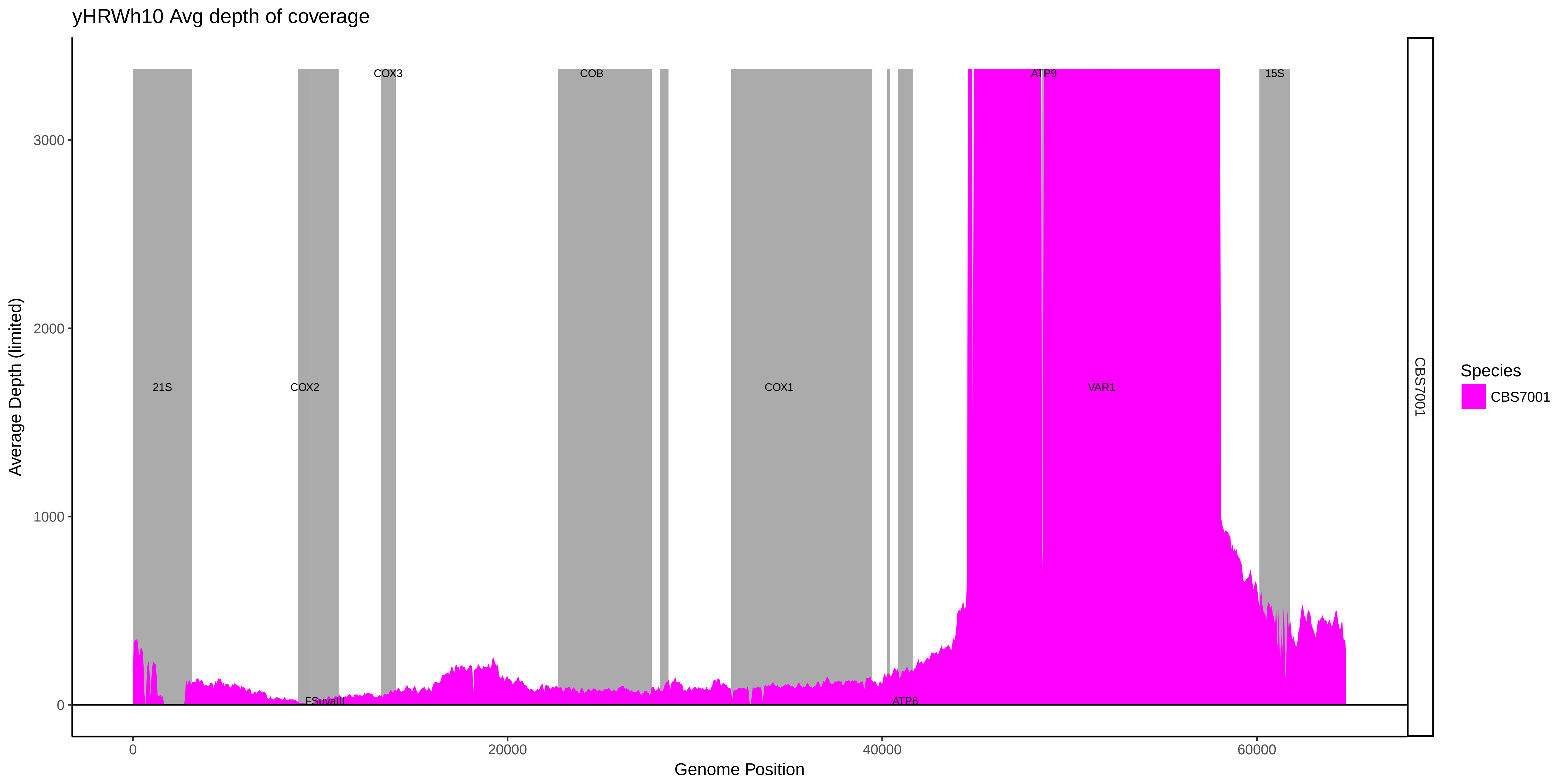

yHRWh10 Avg depth of coverage
Average Depth (limited)
3000
2000
1000
0
0
20000
40000
60000
Genome Position
21S
COX2
COX3
COB
COX1
ATP9
VAR1
15S
ESuvaIII
ATP6
CBS7001
Species
CBS7001
