# Supplementary material for: Synthetic hybrids of six yeast species
Source: Nat Commun. 2020 Apr 29;11:2085. doi: 10.1038/s41467-020-15559-4 (PMC7190663; doi:10.1038/s41467-020-15559-4)
Supplement: Supplementary file 15 — Source Data [file 41467_2020_15559_MOESM15_ESM.zip › Source Data 3.zip!/yHRWh24_mitoSppIDerDepthPlot-d.pdf]

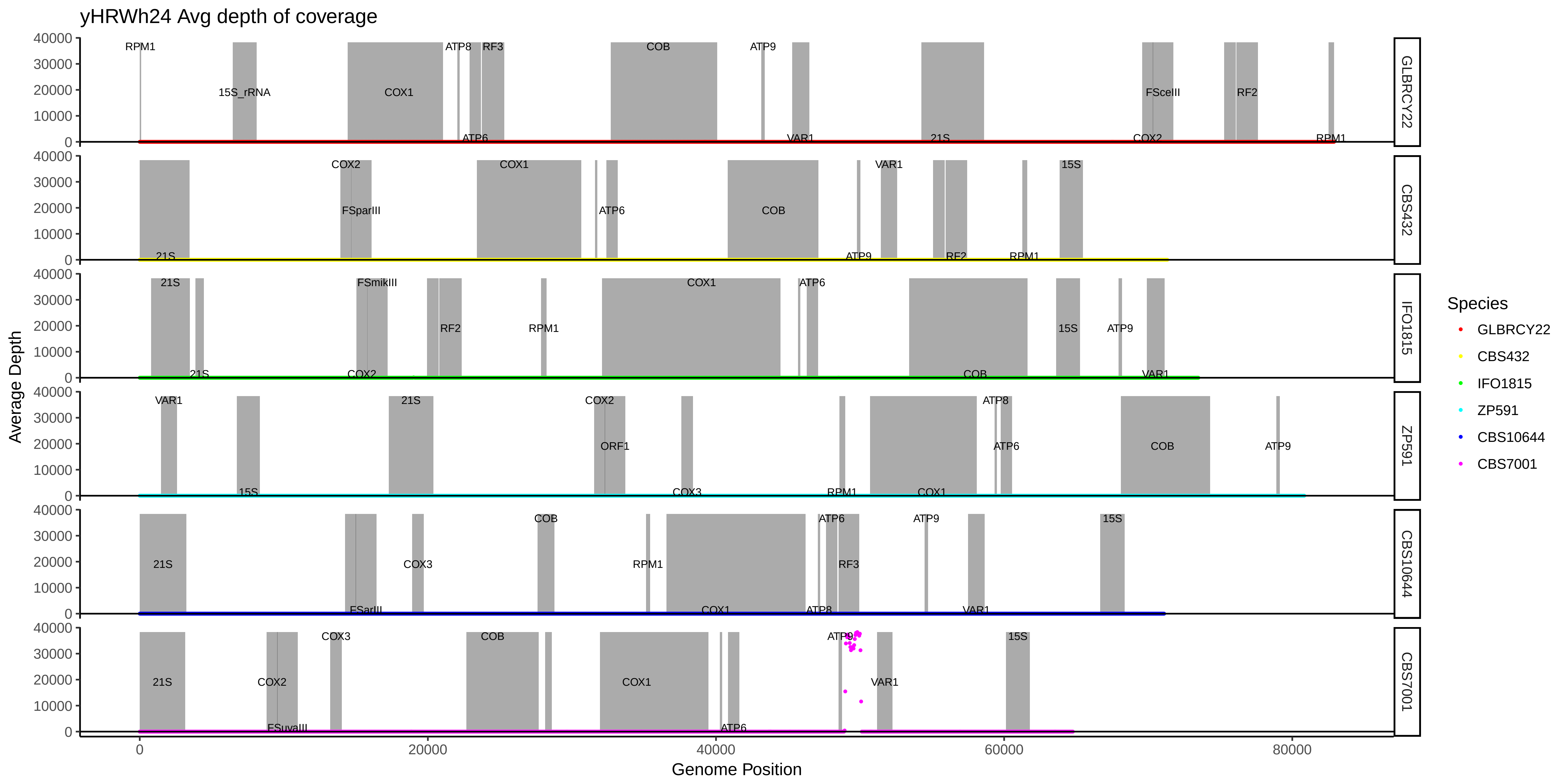
yHRWh24 Avg depth of coverage
Average Depth
Genome Position
0
20000
40000
60000
80000
10000
30000
GLBRCY22
CBS432
IFO1815
ZP591
CBS10644
CBS7001
Species
GLBRCY22
CBS432
IFO1815
ZP591
CBS10644
CBS7001
RPM1
15S_rRNA
COX1
ATP8
RF3
ATP6
COB
ATP9
VAR1
21S
FSceIII
COX2
RF2
COX2
FSparIII
COX1
ATP6
COB
ATP9
VAR1
RF2
RPM1
15S
21S
FSmikIII
COX2
RF2
RPM1
COX1
ATP6
COB
15S
ATP9
VAR1
VAR1
15S
21S
COX2
ORF1
COX3
RPM1
COX1
ATP8
ATP6
COB
ATP9
21S
FSarIII
COX3
COB
RPM1
COX1
ATP6
ATP8
RF3
ATP9
VAR1
15S
21S
COX2
FSuvaIII
COX3
COB
COX1
ATP6
ATP9
VAR1
15S

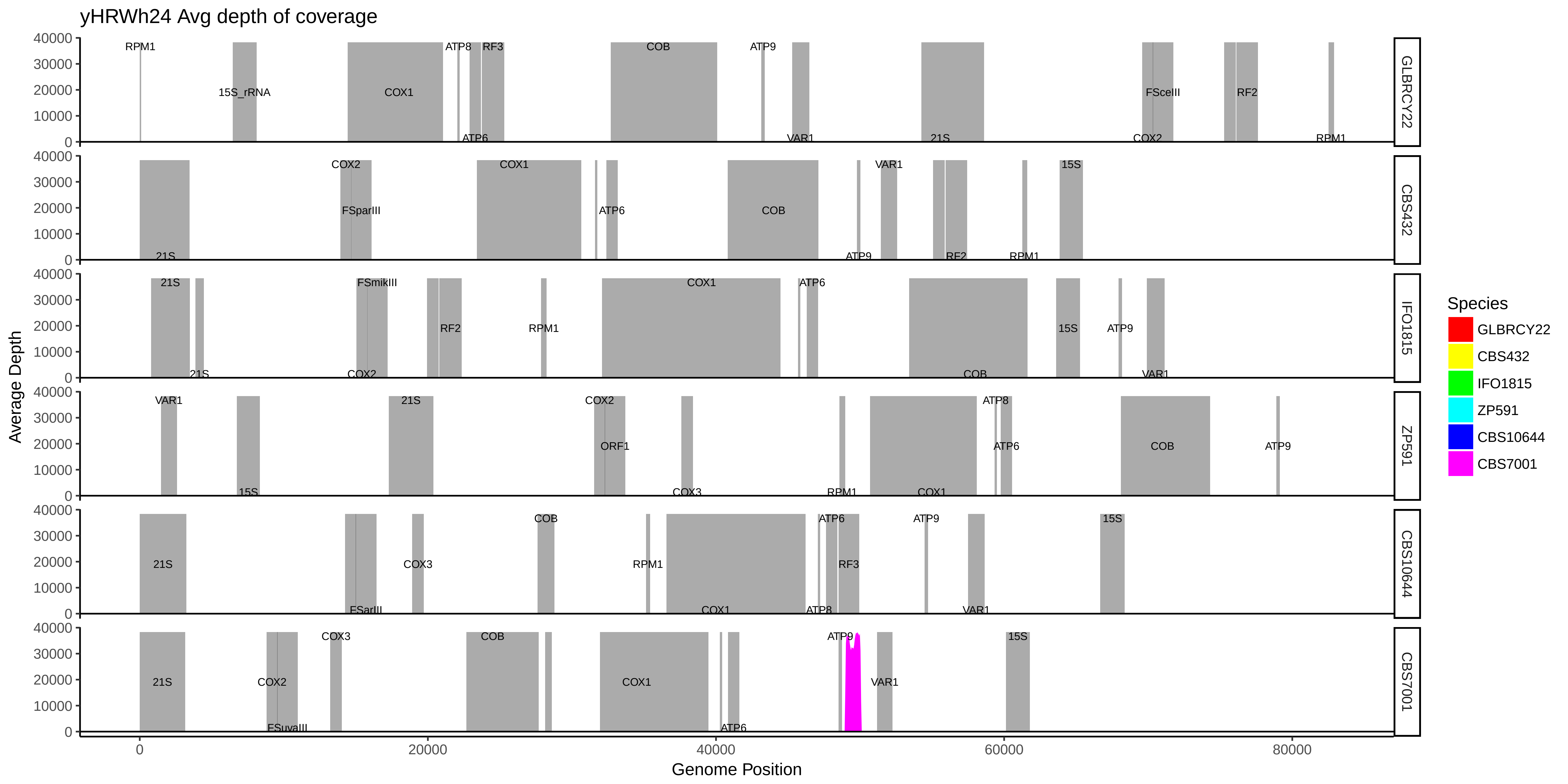

yHRWh24 Avg depth of coverage
Average Depth
Genome Position
GLBRCY22
CBS432
IFO1815
ZP591
CBS10644
CBS7001
Species
RPM1
15S_rRNA
COX1
ATP8
RF3
ATP6
COB
ATP9
VAR1
21S
FSceIII
COX2
RF2
FSparIII
15S
FSmikIII
ORF1
COX3
FSarIII
FSuvaIII
0
10000
20000
30000
40000
60000
80000

yHRWh24 Log2 Avg depth of coverage

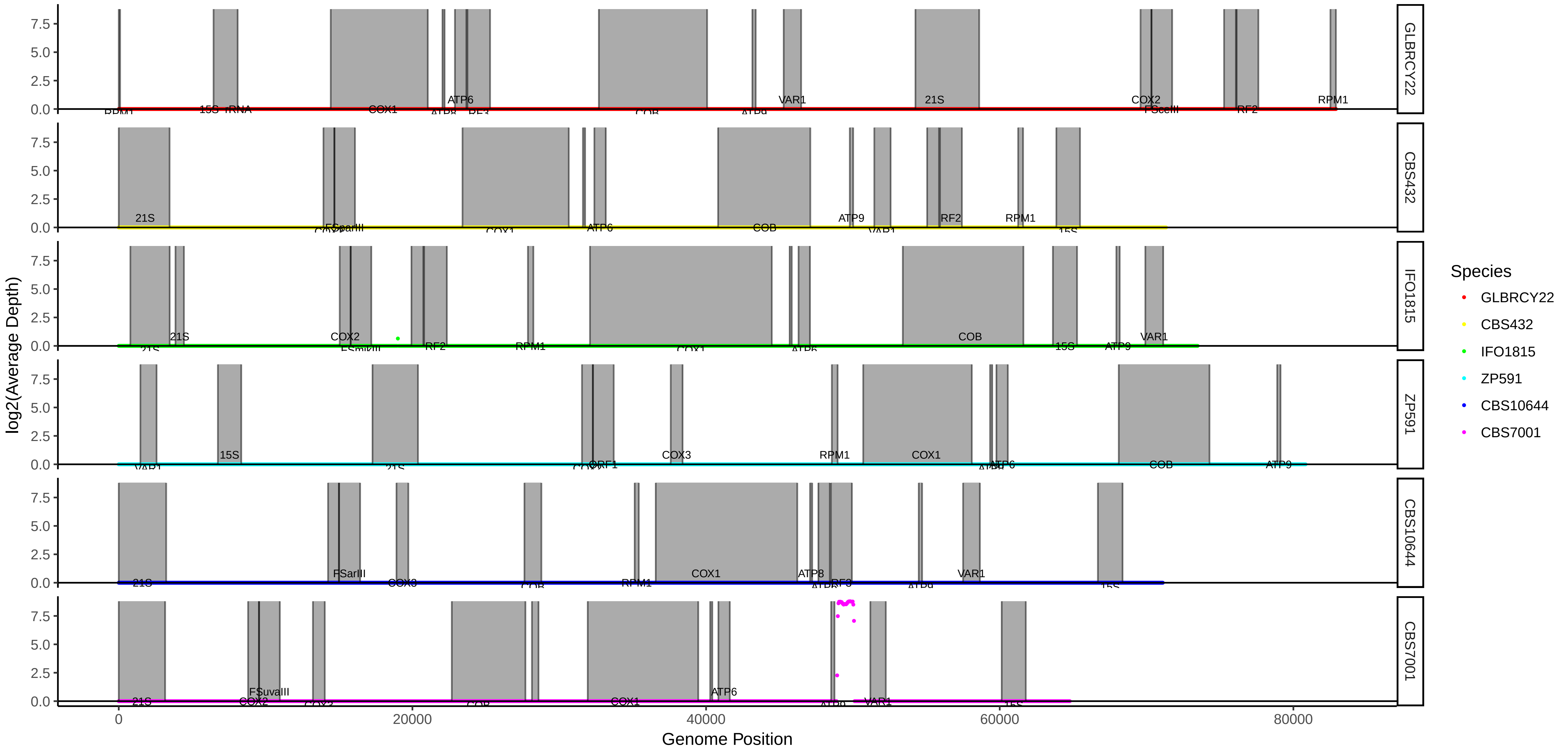

yHRWh24 Log2 Avg depth of coverage

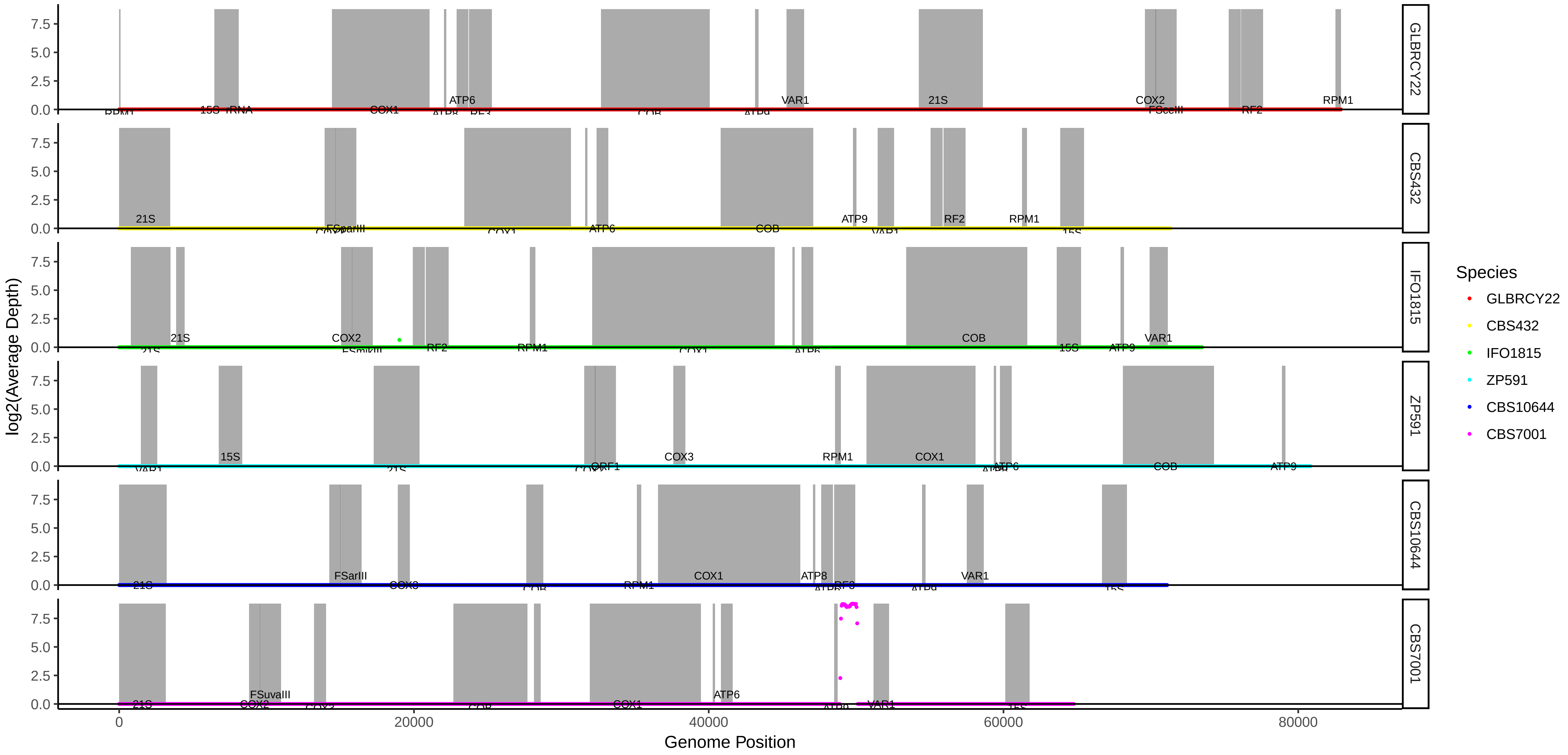

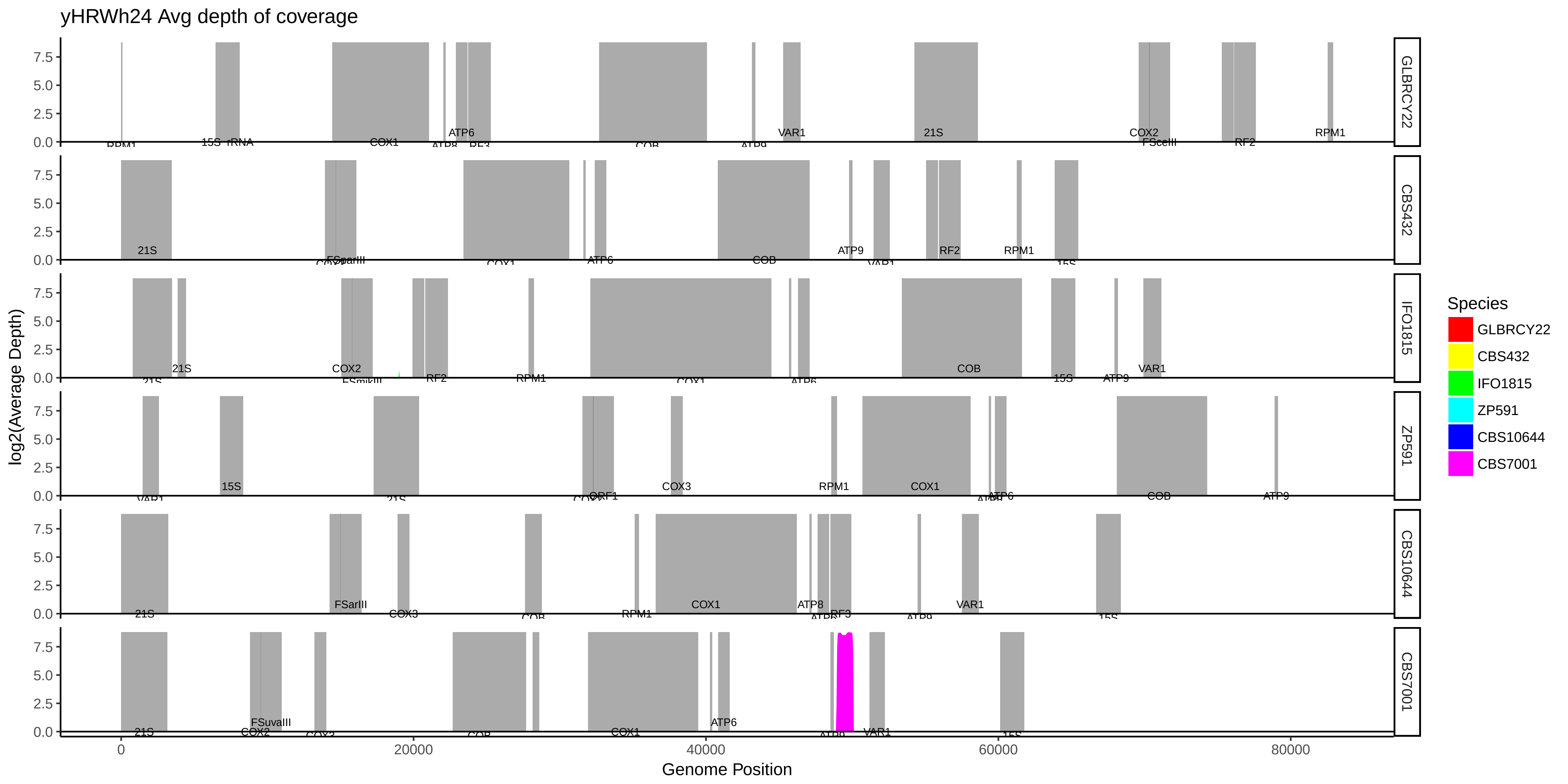

yHRWh24 Avg depth of coverage
log2(Average Depth)
Genome Position
0
20000
40000
60000
80000
0.0
2.5
5.0
7.5
GLBRCY22
CBS432
IFO1815
ZP591
CBS10644
CBS7001
Species
GLBRCY22
CBS432
IFO1815
ZP591
CBS10644
CBS7001
15S
rRNA
COX1
ATP6
VAR1
21S
COX2
RF2
RPM1
21S
FSparIII
COX1
ATP6
COB
ATP9
RF2
RPM1
21S
COX2
RF2
RPM1
COX1
COB
15S
ATP9
VAR1
15S
ORF1
COX3
RPM1
COX1
ATP6
COB
ATP9
21S
FSarIII
COX3
RPM1
COX1
ATP8
RF3
VAR1
21S
COX2
FSuvaIII
COX1
ATP6
VAR1

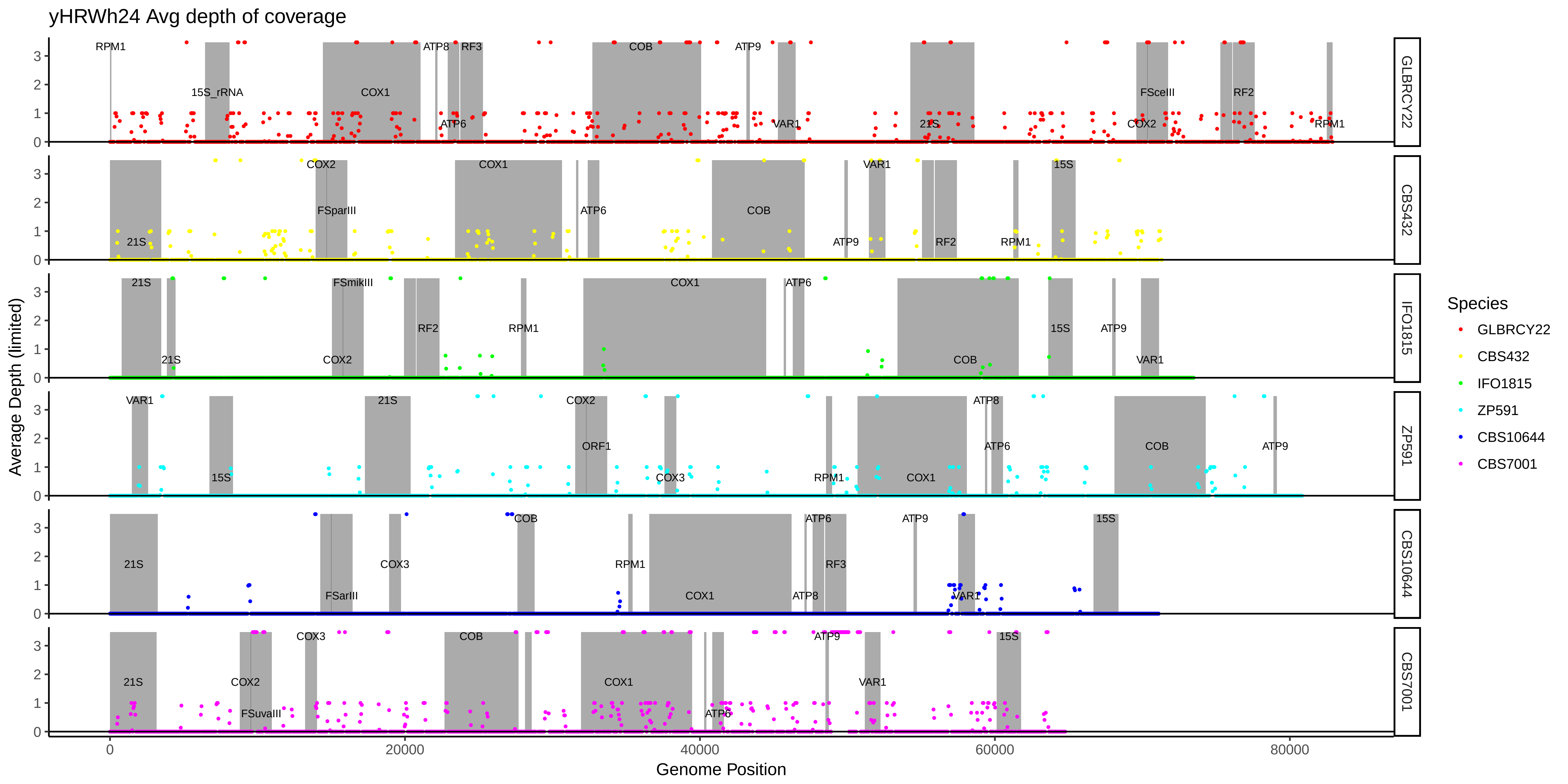
yHRWh24 Avg depth of coverage
Average Depth (limited)
Genome Position
0
20000
40000
60000
80000
GLBRCY22
CBS432
IFO1815
ZP591
CBS10644
CBS7001
Species
GLBRCY22
CBS432
IFO1815
ZP591
CBS10644
CBS7001
RPM1
15S_rRNA
COX1
ATP8
RF3
ATP6
COB
ATP9
VAR1
21S
FSceIII
COX2
RF2
COX2
FSparIII
COX1
ATP6
COB
ATP9
VAR1
RF2
RPM1
15S
21S
FSmikIII
RF2
RPM1
COX1
ATP6
COB
15S
ATP9
VAR1
VAR1
15S
21S
COX2
ORF1
COX3
RPM1
COX1
ATP8
ATP6
COB
ATP9
21S
FSarIII
COX3
COB
RPM1
COX1
ATP6
ATP8
RF3
ATP9
VAR1
15S
21S
COX2
FSuvaIII
COX3
COB
COX1
ATP6
ATP9
VAR1
15S

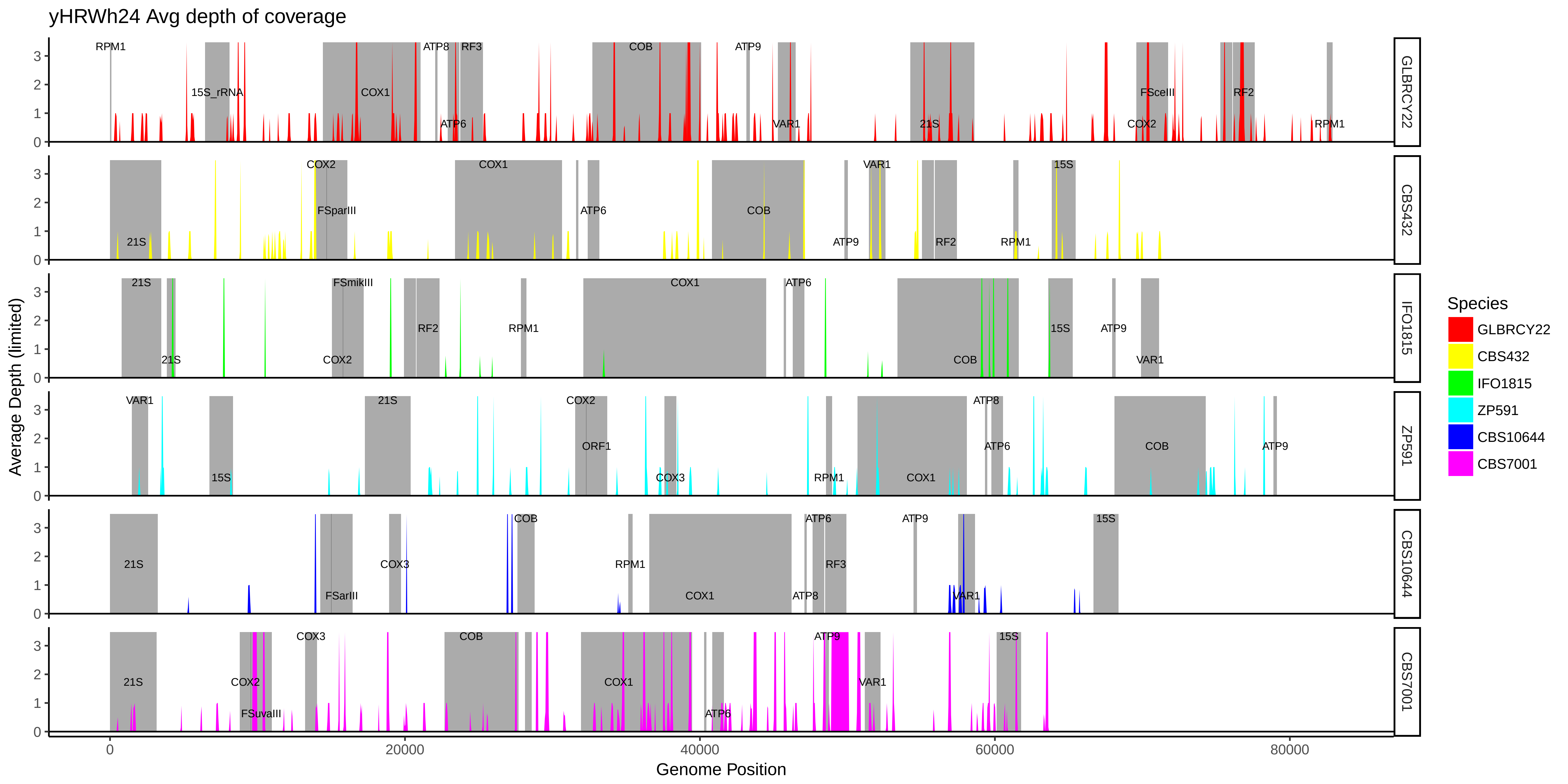

yHRWh24 Avg depth of coverage
Average Depth (limited)
Genome Position
Species
GLBRCY22
CBS432
IFO1815
ZP591
CBS10644
CBS7001
RPM1
15S_rRNA
COX1
ATP8
RF3
ATP6
COB
ATP9
VAR1
21S
FSceIII
COX2
RF2
FSparIII
15S
FSmikIII
RPM1
ORF1
COX3
FSarIII
FSuvaIII
0
20000
40000
60000
80000

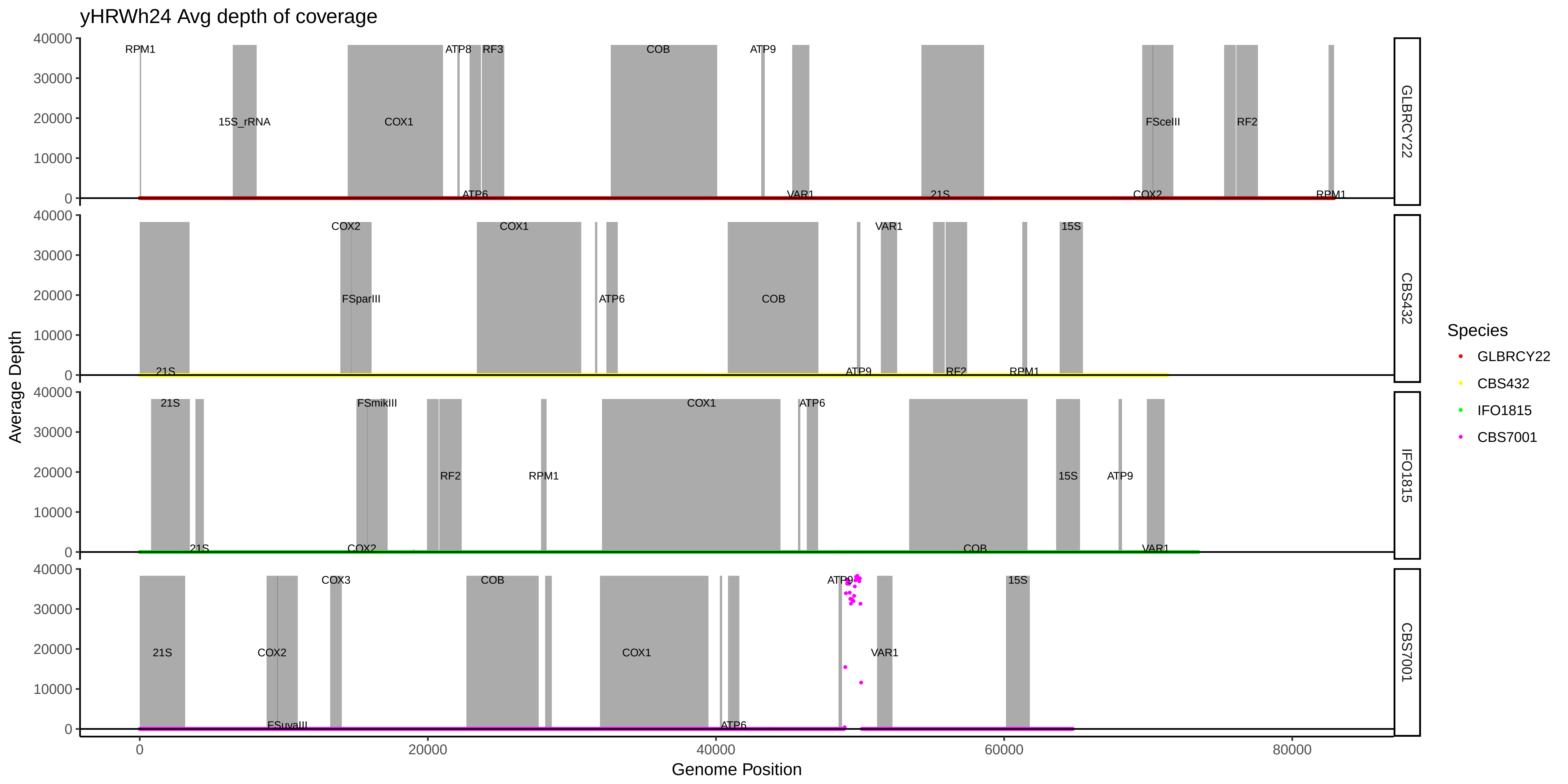

yHRWh24 Avg depth of coverage
Average Depth
Genome Position
GLBRCY22
CBS432
IFO1815
CBS7001
Species
GLBRCY22
CBS432
IFO1815
CBS7001
RPM1
15S_rRNA
COX1
ATP8
RF3
ATP6
COB
ATP9
VAR1
21S
FSceIII
COX2
RF2
COX2
FSparIII
ATP6
COB
VAR1
15S
21S
ATP9
RF2
RPM1
21S
FSmikIII
RF2
RPM1
COX1
ATP6
15S
ATP9
COX2
COB
VAR1
21S
COX2
FSuvaIII
COX3
COB
COX1
ATP6
ATP9
VAR1
15S
0
10000
20000
30000
40000
0
20000
40000
60000
80000

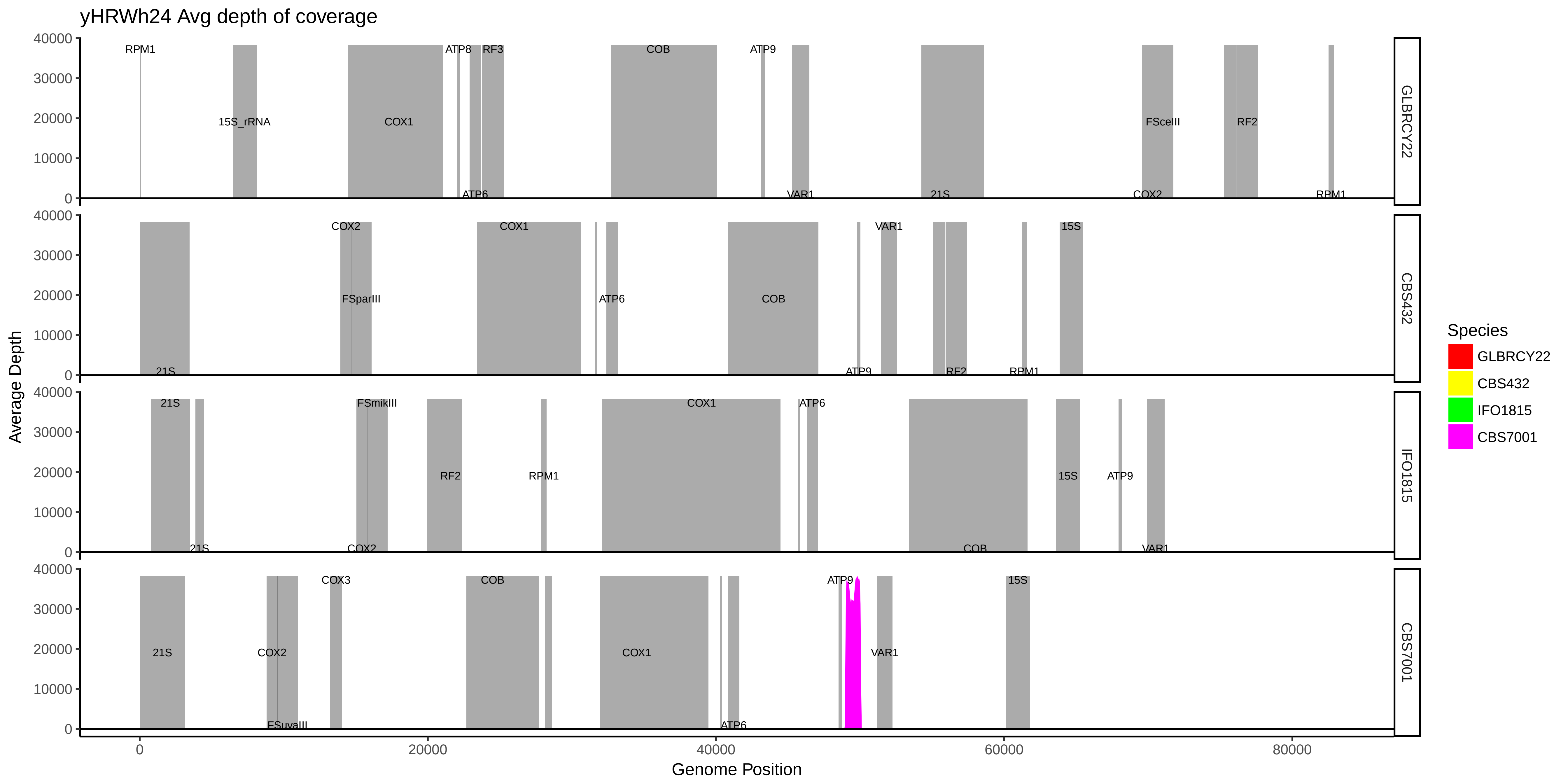

yHRWh24 Avg depth of coverage
Average Depth
Genome Position
GLBRCY22
CBS432
IFO1815
CBS7001
Species
RPM1
15S_rRNA
COX1
ATP8
RF3
ATP6
COB
ATP9
VAR1
21S
FSceIII
COX2
RF2
FSparIII
15S
FSmikIII
RPM1
COX3
FSuvaIII

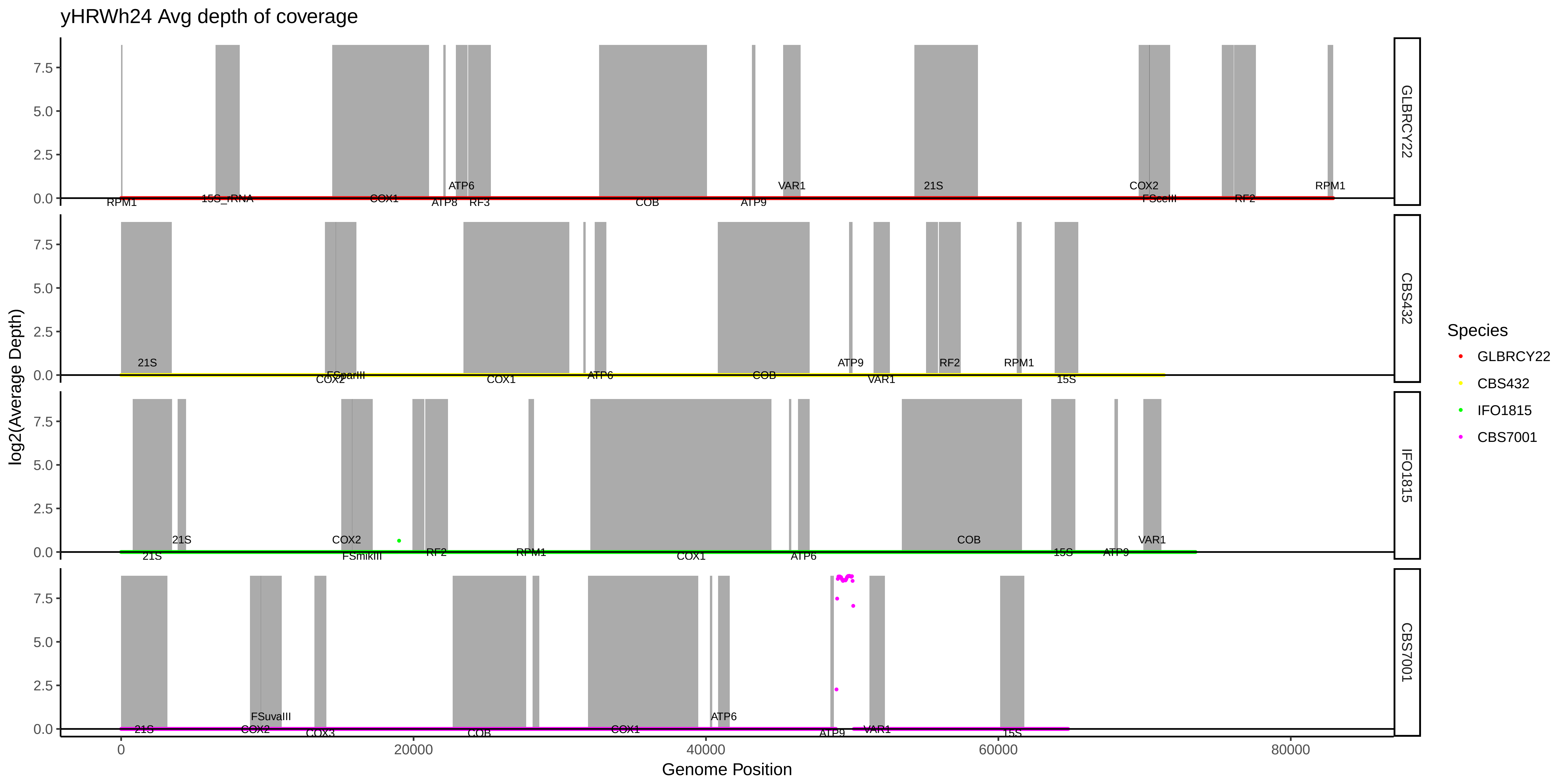
yHRWh24 Avg depth of coverage
log2(Average Depth)
Genome Position
GLBRCY22
CBS432
IFO1815
CBS7001
Species
GLBRCY22
CBS432
IFO1815
CBS7001
RPM1
15S_rRNA
COX1
ATP6
ATP8
RF3
COB
ATP9
VAR1
21S
COX2
FScerIII
RF2
FSparIII
RPM1
15S
FSmikIII
FSuvaIII
COX3

yHRWh24 Avg depth of coverage

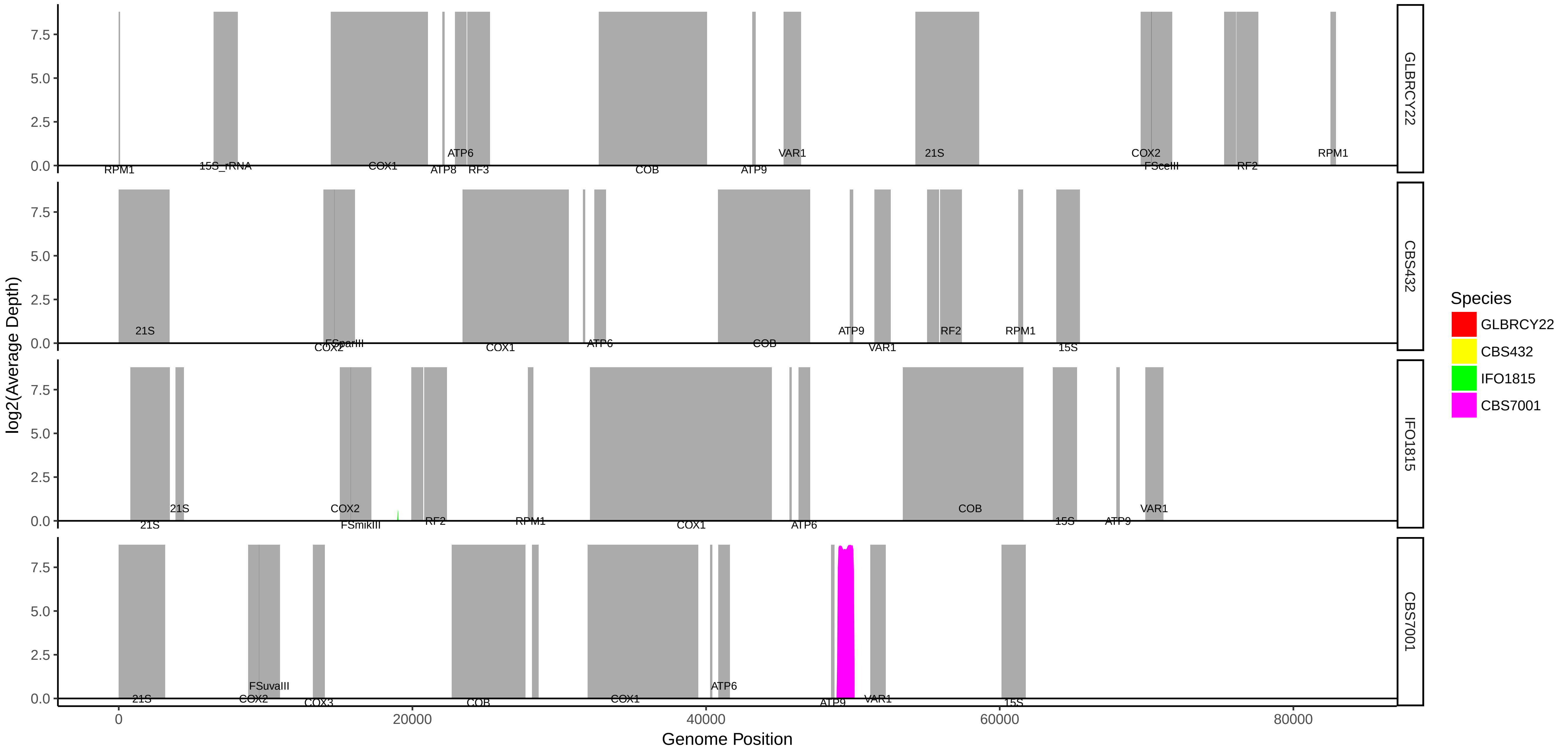

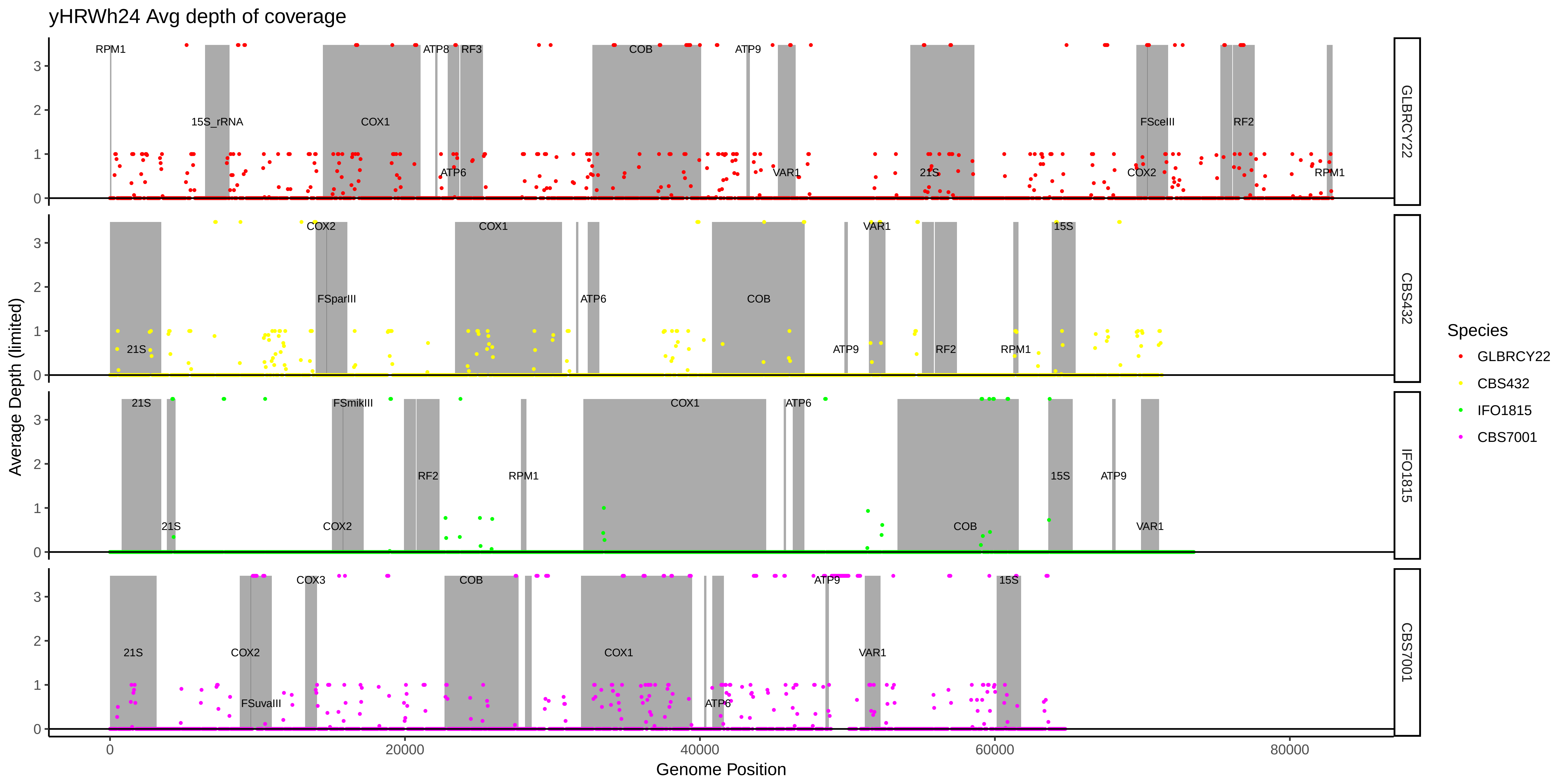

yHRWh24 Avg depth of coverage
Average Depth (limited)
Genome Position
GLBRCY22
CBS432
IFO1815
CBS7001
Species
GLBRCY22
CBS432
IFO1815
CBS7001
RPM1
15S_rRNA
COX1
ATP8
RF3
ATP6
COB
ATP9
VAR1
21S
FSceIII
COX2
RF2
COX2
FSparIII
ATP6
COB
ATP9
VAR1
RF2
RPM1
15S
21S
FSmikIII
RF2
RPM1
COX1
ATP6
COB
15S
ATP9
VAR1
COX3
COB
COX1
ATP9
15S
COX2
FSuvaIII
ATP6
0
20000
40000
60000
80000

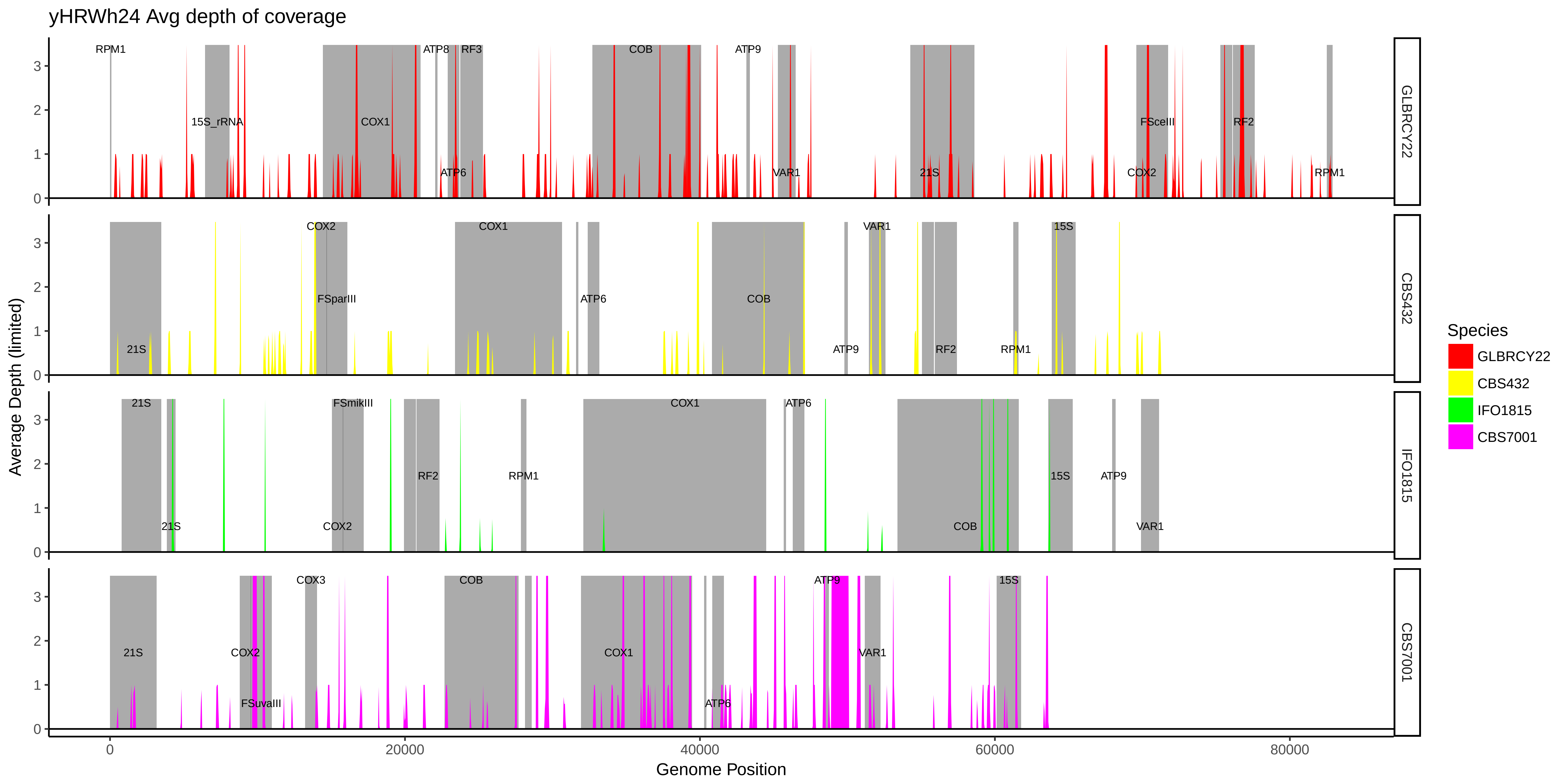

yHRWh24 Avg depth of coverage
Average Depth (limited)
Genome Position
GLBRCY22
CBS432
IFO1815
CBS7001
Species
GLBRCY22
CBS432
IFO1815
CBS7001
RPM1
15S_rRNA
COX1
ATP8
RF3
ATP6
COB
ATP9
VAR1
21S
FSceIII
COX2
RF2
21S
COX2
FSparIII
ATP6
ATP9
RF2
RPM1
15S
FSmikIII
RF2
COX3
COX2
FSuvaIII
0
20000
40000
60000
80000
