# Supplementary material for: Synthetic hybrids of six yeast species
Source: Nat Commun. 2020 Apr 29;11:2085. doi: 10.1038/s41467-020-15559-4 (PMC7190663; doi:10.1038/s41467-020-15559-4)
Supplement: Supplementary file 15 — Source Data [file 41467_2020_15559_MOESM15_ESM.zip › Source Data 3.zip!/yHRWh42_mitoSppIDerDepthPlot-d.pdf]

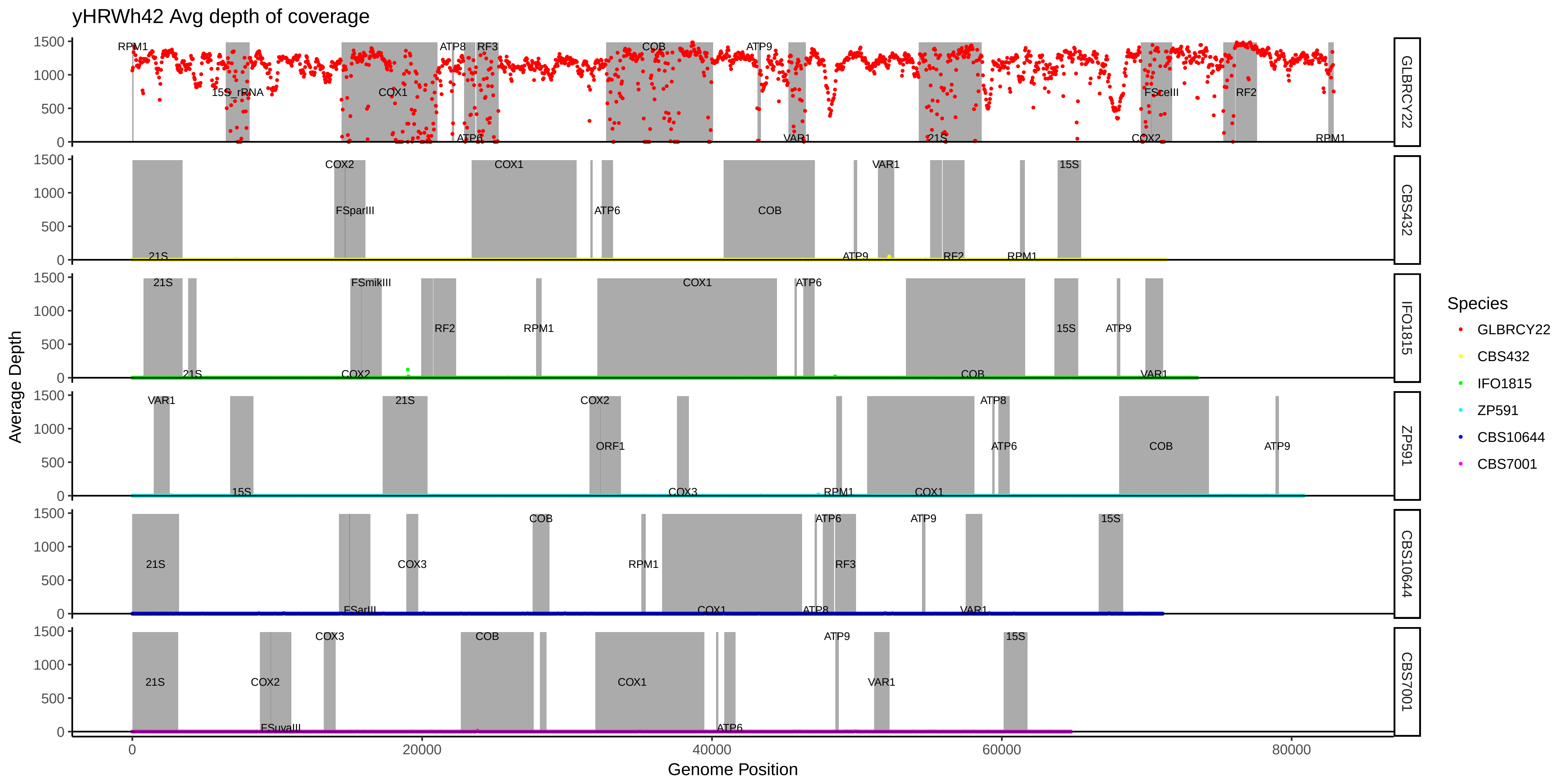

yHRWh42 Avg depth of coverage
Average Depth
Genome Position
Species
GLBRCY22
CBS432
IFO1815
ZP591
CBS10644
CBS7001

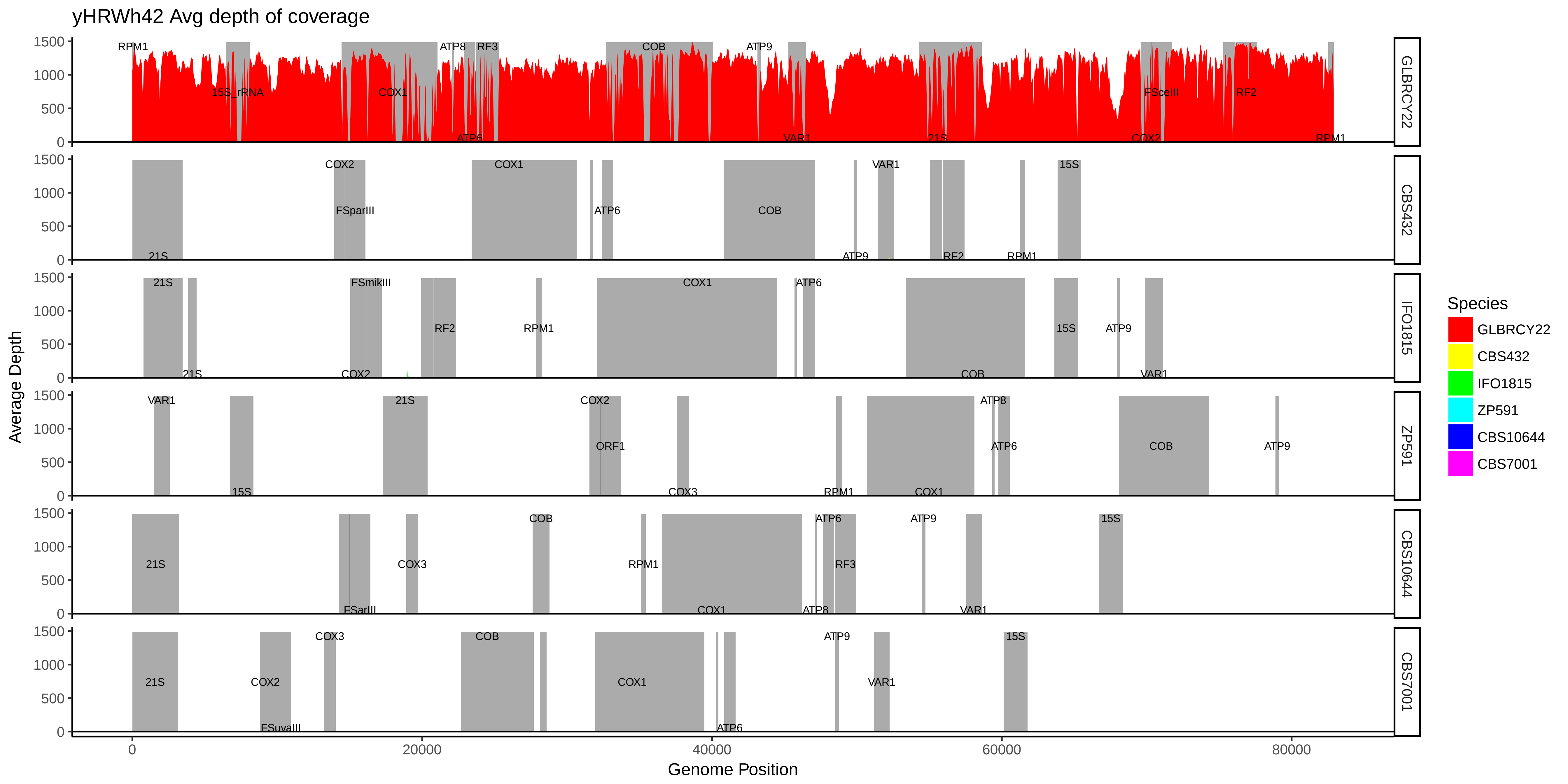

yHRWh42 Avg depth of coverage
Average Depth
Genome Position
Species
GLBRCY22
CBS432
IFO1815
ZP591
CBS10644
CBS7001

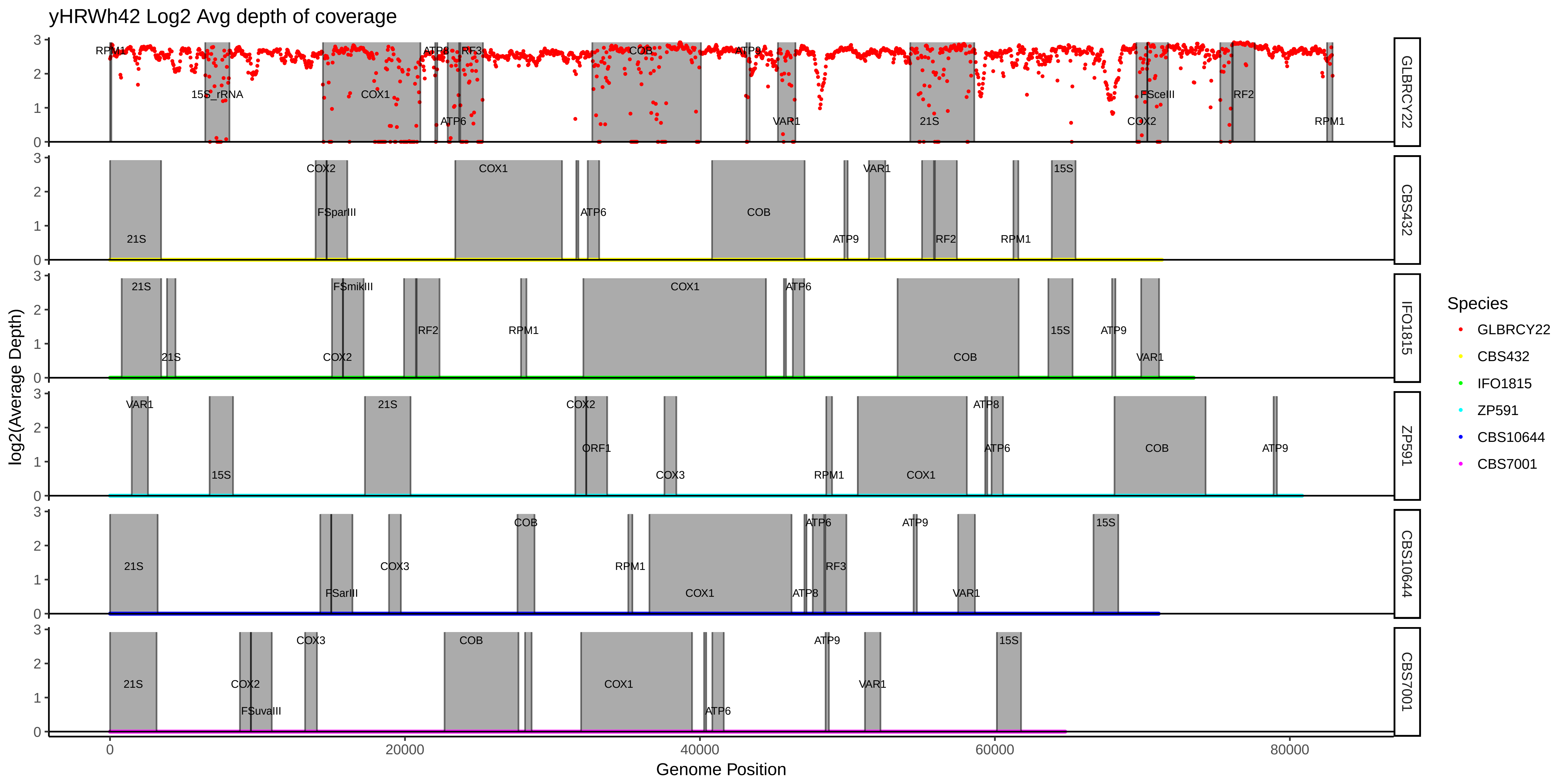

yHRWh42 Log2 Avg depth of coverage
log2(Average Depth)
Genome Position
GLBRCY22
CBS432
IFO1815
ZP591
CBS10644
CBS7001
Species
GLBRCY22
CBS432
IFO1815
ZP591
CBS10644
CBS7001
0
20000
40000
60000
80000
RPM1
15S_rRNA
COX1
ATP8
RF3
ATP6
COB
ATP9
VAR1
21S
FSceIII
COX2
RF2
21S
COX2
FSparIII
COX1
ATP6
COB
ATP9
VAR1
RF2
RPM1
15S
FSmikIII
RPM1
COX3
ORF1
FSarIII
FSuvaIII

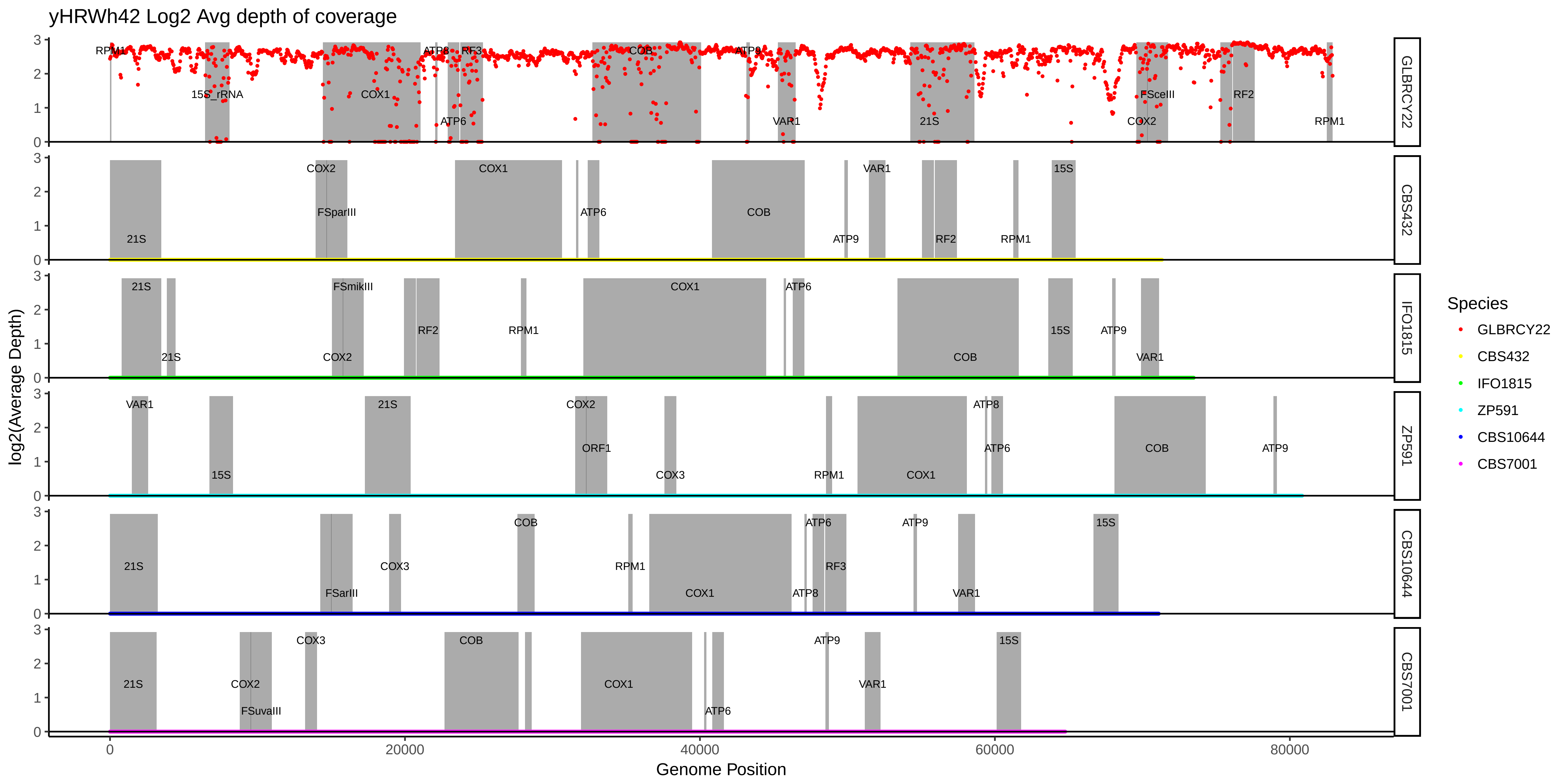

yHRWh42 Log2 Avg depth of coverage
log2(Average Depth)
Genome Position
Species
GLBRCY22
CBS432
IFO1815
ZP591
CBS10644
CBS7001
RPM1
15S_rRNA
COX1
ATP8
RF3
ATP6
COB
ATP9
VAR1
21S
FSceIII
COX2
RF2
COX2
FSparIII
COX1
ATP6
COB
ATP9
VAR1
RF2
RPM1
15S
21S
FSmikIII
RF2
RPM1
COX1
ATP6
COB
15S
ATP9
VAR1
COX2
VAR1
15S
21S
COX2
ORF1
COX3
RPM1
COX1
ATP8
ATP6
COB
ATP9
21S
FSarIII
COX3
COB
RPM1
COX1
ATP8
ATP6
RF3
ATP9
VAR1
15S
21S
COX2
FSuvaIII
COX3
COB
COX1
ATP6
ATP9
VAR1
15S
0
20000
40000
60000
80000

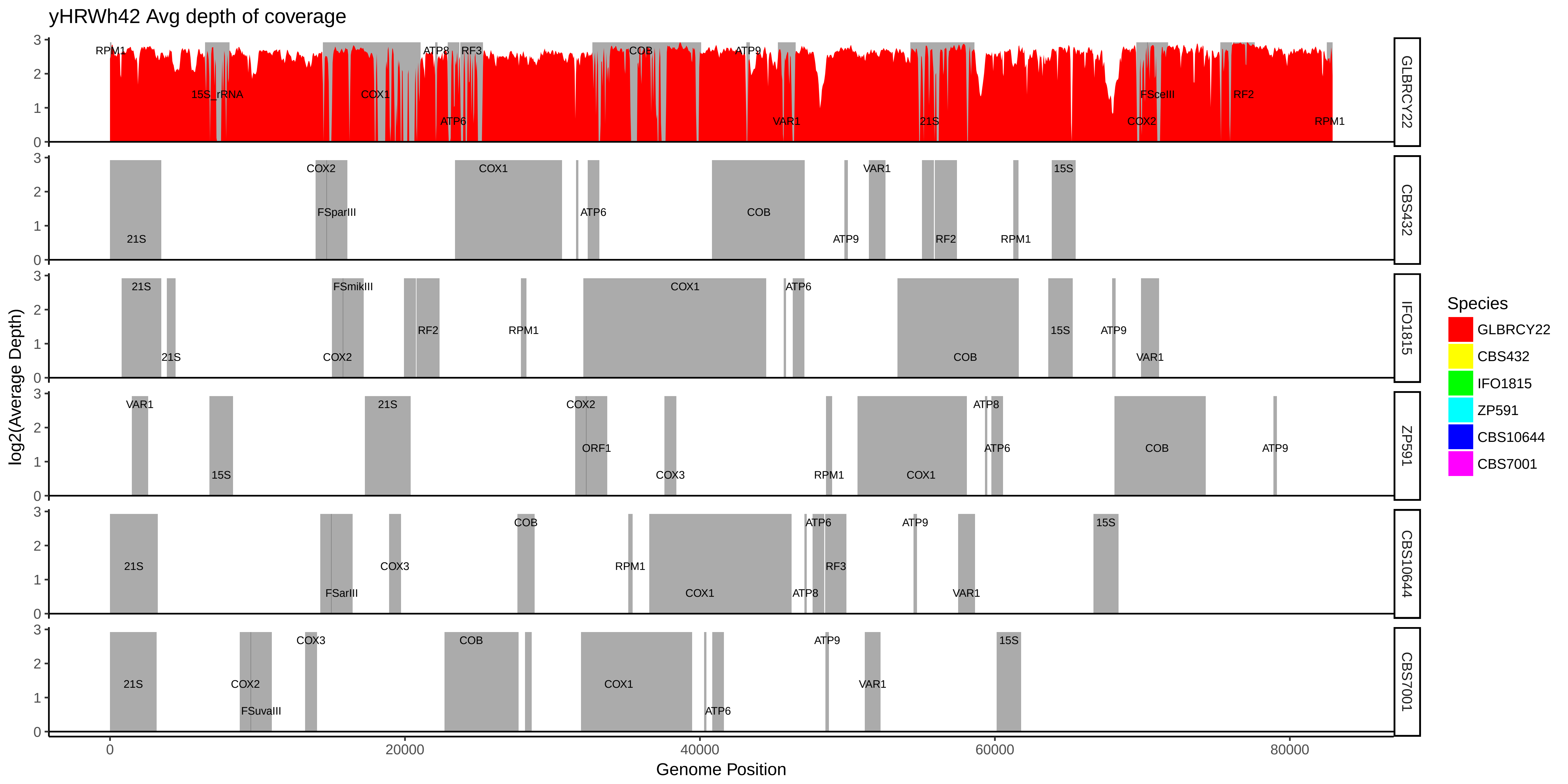

yHRWh42 Avg depth of coverage
log2(Average Depth)
Genome Position
0
20000
40000
60000
80000
GLBRCY22
CBS432
IFO1815
ZP591
CBS10644
CBS7001
Species
GLBRCY22
CBS432
IFO1815
ZP591
CBS10644
CBS7001
RPM1
15S_rRNA
COX1
ATP8
RF3
ATP6
COB
ATP9
VAR1
21S
FScelII
COX2
RF2
21S
COX2
FSparIII
COX1
ATP6
COB
ATP9
VAR1
RF2
RPM1
15S
FSmikIII
RF2
RPM1
ATP6
15S
ATP9
VAR1
ORF1
COX3
ATP8
FSarIII
FSuvaIII

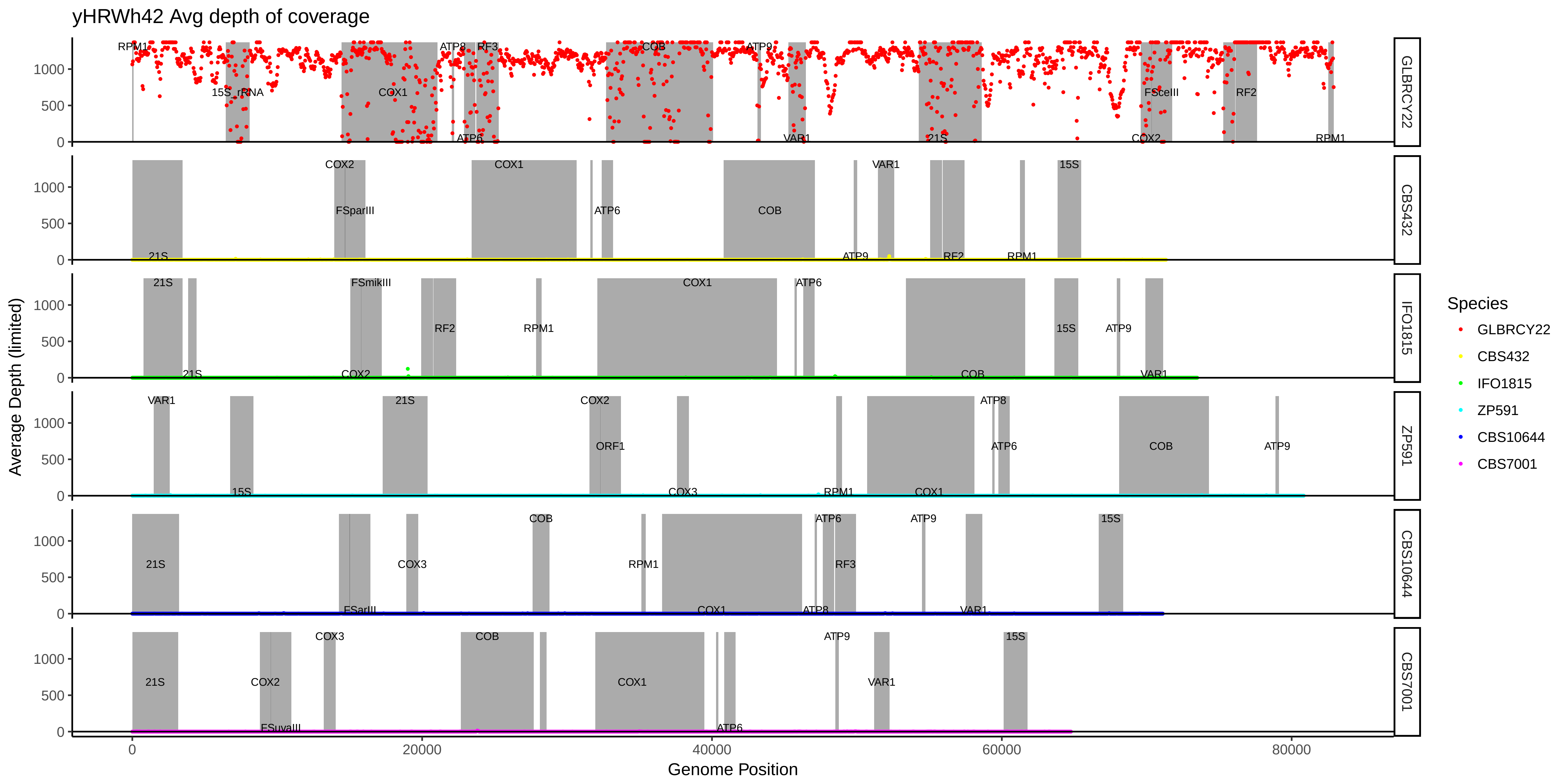
yHRWh42 Avg depth of coverage
Average Depth (limited)
Genome Position
GLBRCY22
CBS432
IFO1815
ZP591
CBS10644
CBS7001
Species
GLBRCY22
CBS432
IFO1815
ZP591
CBS10644
CBS7001
0
500
1000
0
20000
40000
60000
80000
RPM1
15S_rRNA
COX1
ATP8
ATP6
RF3
COB
ATP9
VAR1
21S
COX2
FSceIII
RF2
21S
COX2
FSparIII
COX1
ATP6
COB
ATP9
VAR1
RF2
RPM1
15S
21S
FSmikIII
COX2
RF2
RPM1
COX1
ATP6
COB
15S
ATP9
VAR1
VAR1
15S
21S
COX2
ORF1
COX3
RPM1
COX1
ATP8
ATP6
COB
ATP9
21S
FSarIII
COX3
COB
RPM1
COX1
ATP6
ATP8
RF3
ATP9
VAR1
15S
21S
COX2
FSuvaIII
COX3
COB
COX1
ATP6
ATP9
VAR1
15S

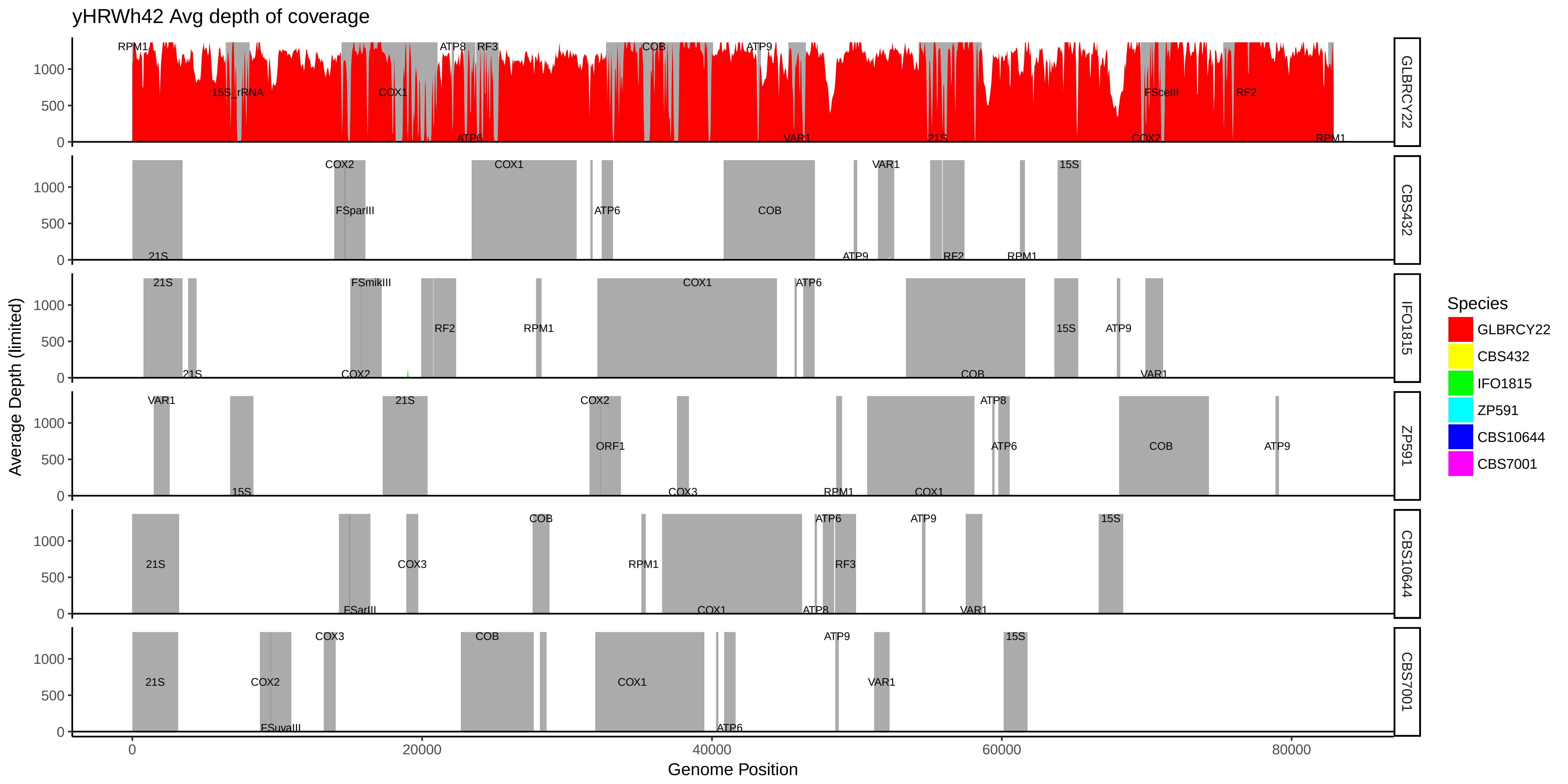

yHRWh42 Avg depth of coverage
Average Depth (limited)
Genome Position
Species
GLBRCY22
CBS432
IFO1815
ZP591
CBS10644
CBS7001
RPM1
15S_rRNA
COX1
ATP8
RF3
ATP6
COB
ATP9
VAR1
21S
FSceIII
COX2
RF2
COX2
FSparIII
ATP6
COB
15S
21S
FSmikIII
RF2
RPM1
ORF1
COX3
FSarIII
FSuvaIII
0
500
1000
20000
40000
60000
80000

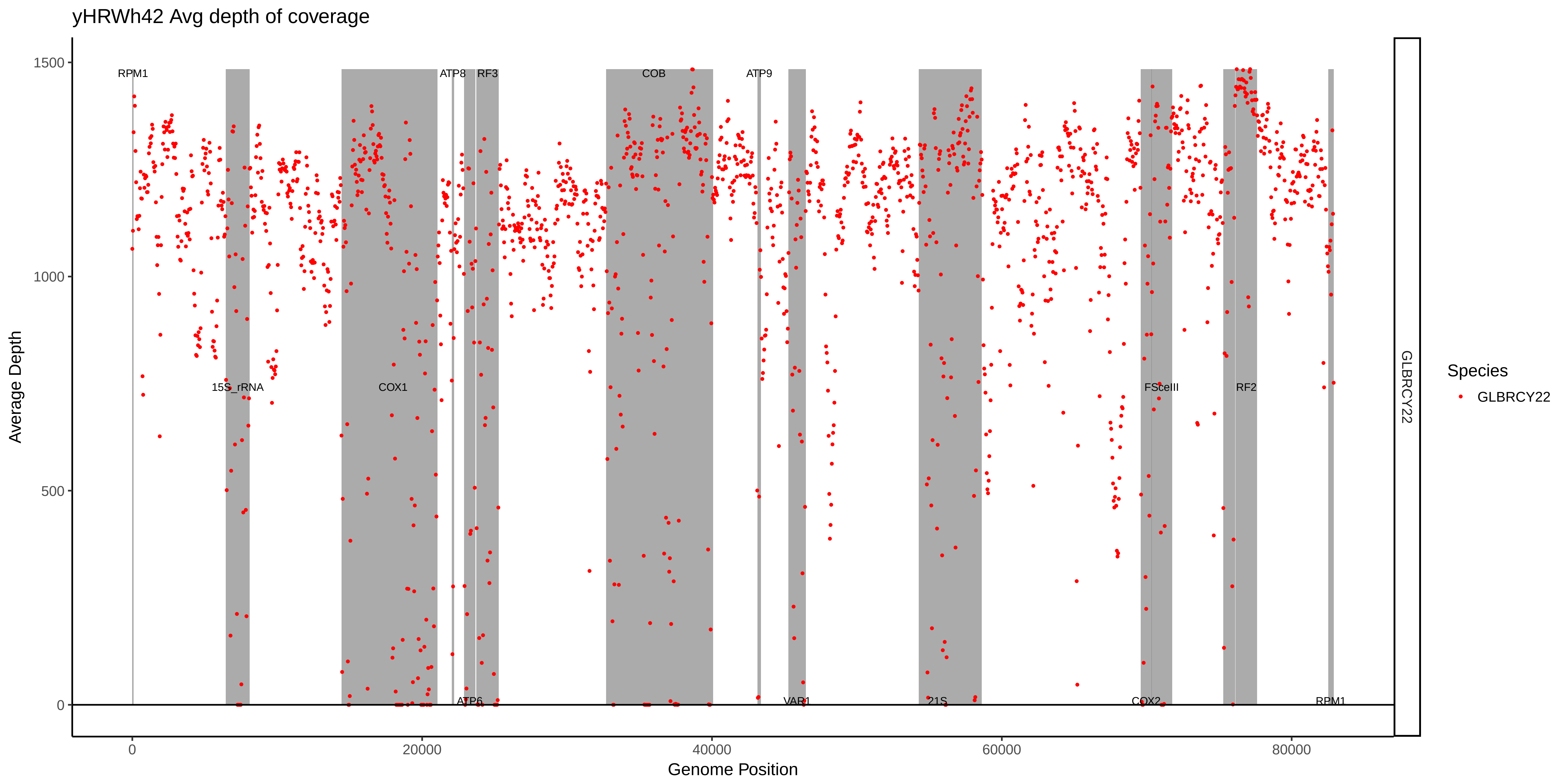

yHRWh42 Avg depth of coverage
Average Depth
1500
1000
500
0
Genome Position
0
20000
40000
60000
80000
RPM1
15S_rRNA
COX1
ATP8
ATP6
RF3
COB
ATP9
VAR1
21S
FScelll
COX2
RF2
RPM1
GLBRCY22
Species
GLBRCY22

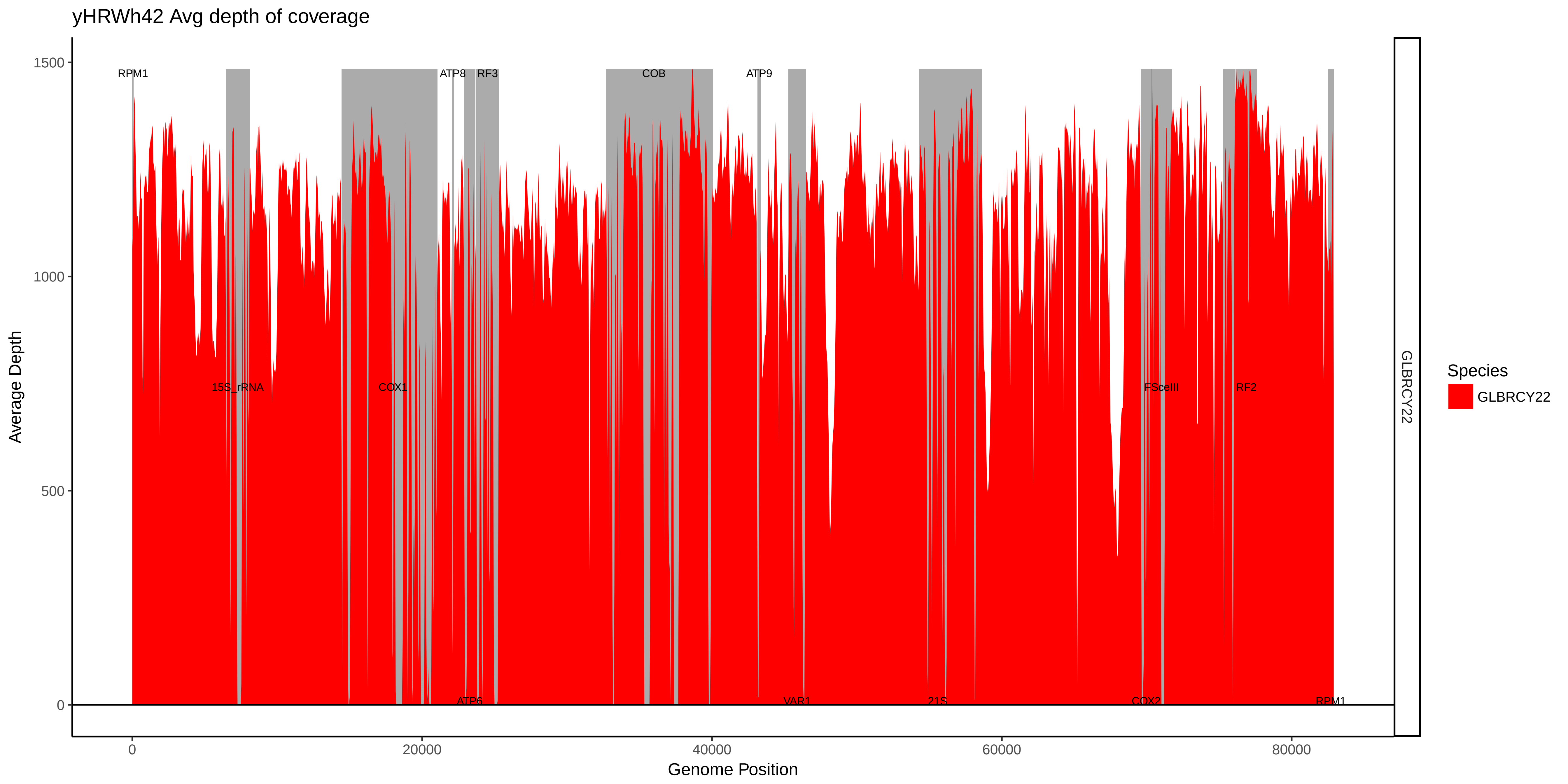
yHRWh42 Avg depth of coverage
Average Depth
1500
1000
500
0
RPM1
ATP8
RF3
COB
ATP9
15S_rRNA
COX1
FScelII
RF2
ATP6
VAR1
21S
COX2
RPM1
0
20000
40000
60000
80000
Genome Position
GLBRCY22
Species
GLBRCY22

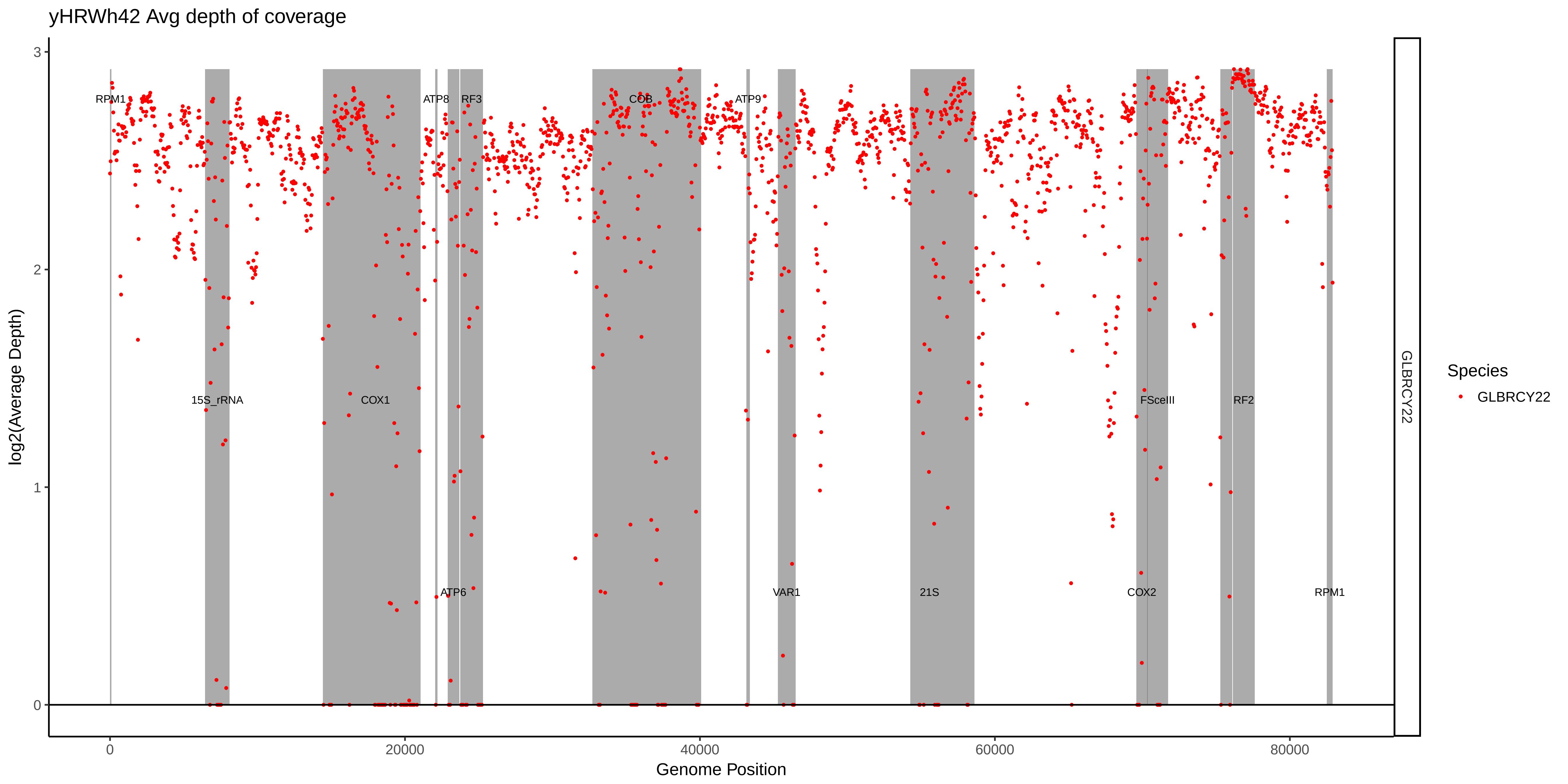

yHRWh42 Avg depth of coverage
log2(Average Depth)
Genome Position
0
1
2
3
0
20000
40000
60000
80000
RPM1
15S_rRNA
COX1
ATP8
ATP6
RF3
COB
ATP9
VAR1
21S
FSceIII
COX2
RF2
RPM1
GLBRCY22
Species
GLBRCY22

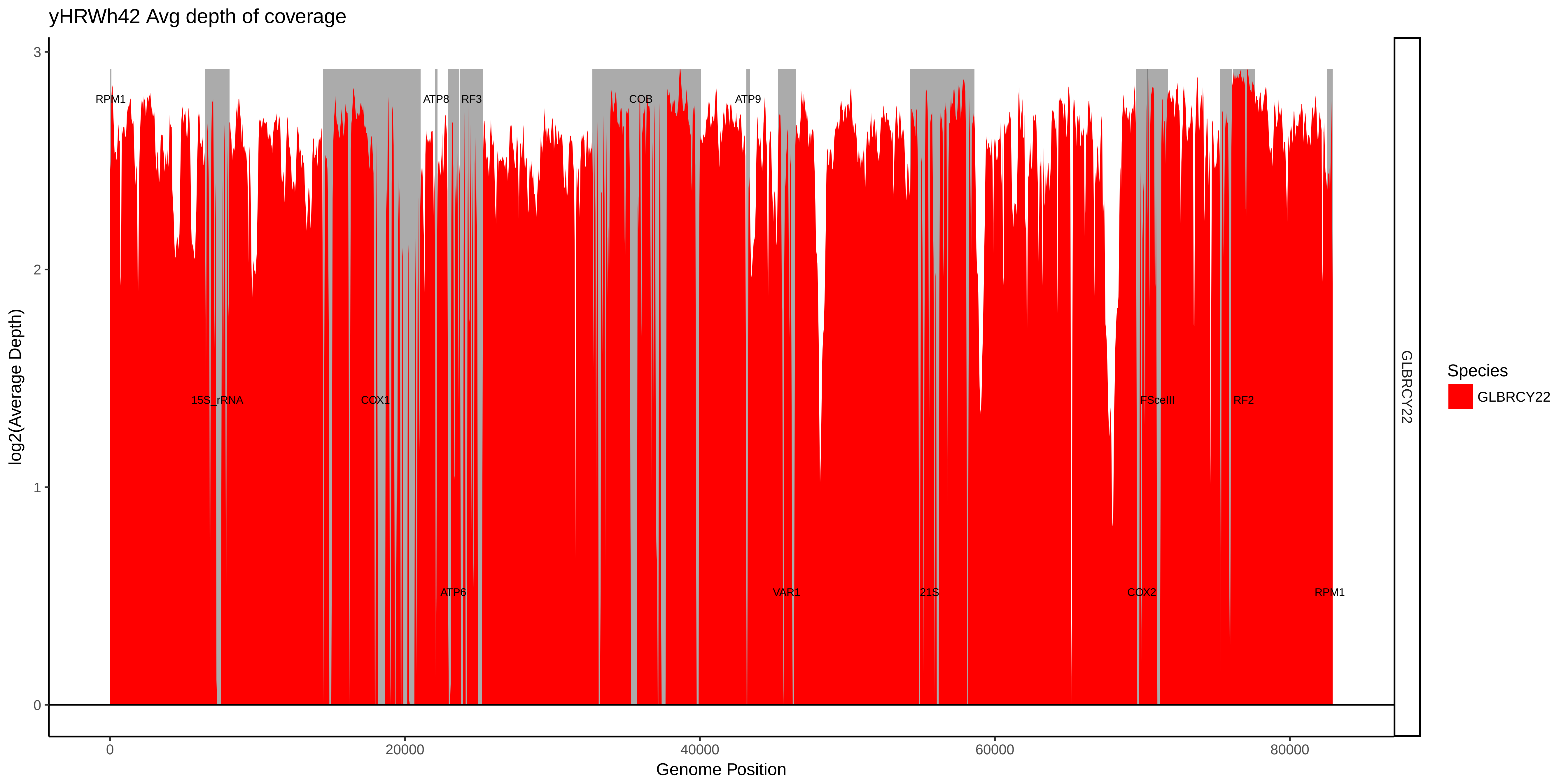

yHRWh42 Avg depth of coverage
log2(Average Depth)
Genome Position
RPM1
ATP8
RF3
COB
ATP9
15S_rRNA
COX1
FScеIII
RF2
ATP6
VAR1
21S
COX2
RPM1
GLBRCY22
Species
GLBRCY22
0
1
2
3
0
20000
40000
60000
80000

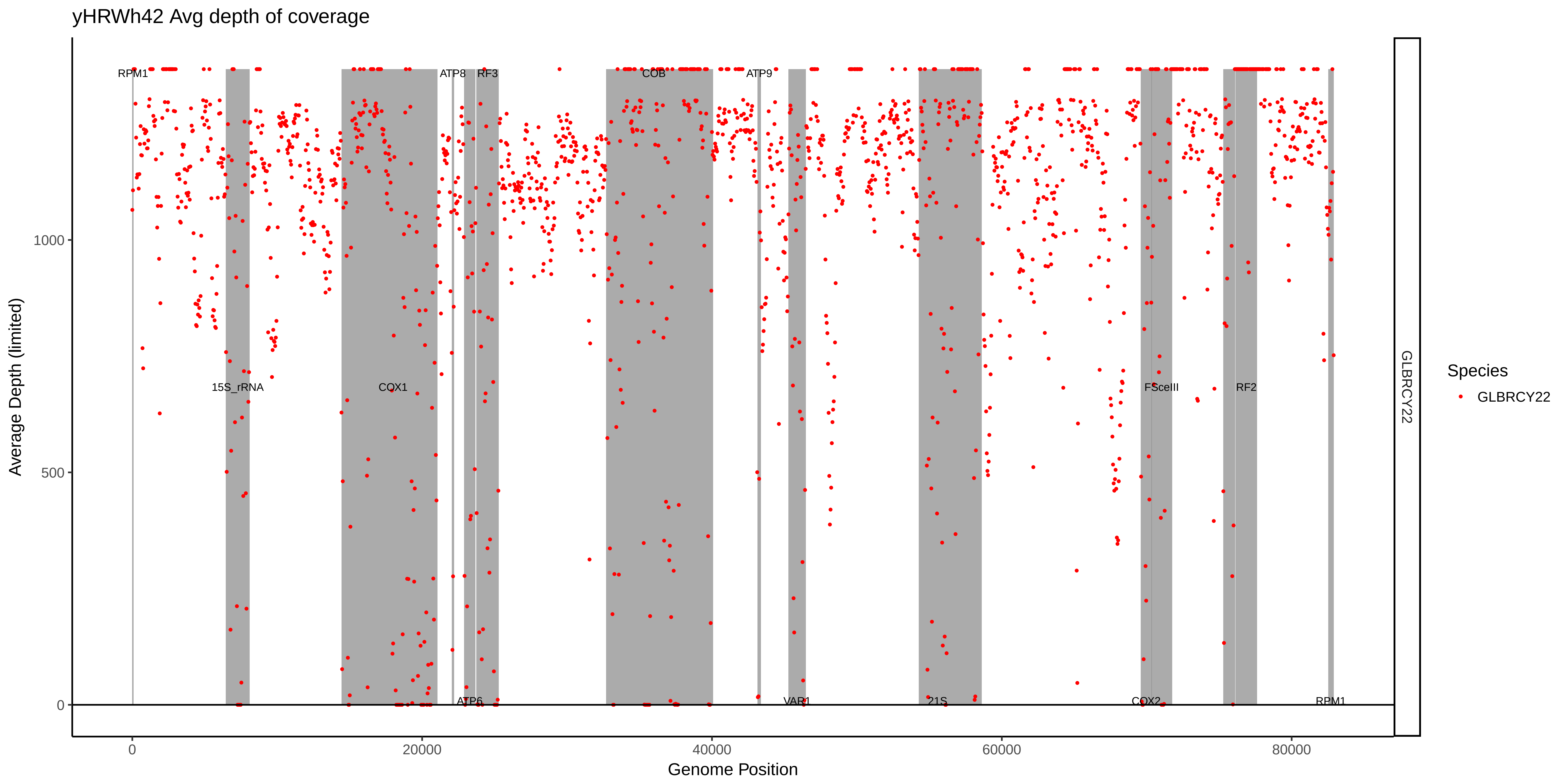

yHRWh42 Avg depth of coverage
RPM1
ATP8
RF3
COB
ATP9
15S_rRNA
COX1
FScelII
RF2
ATP6
VAR1
21S
COX2
RPM1
1000
500
0
Average Depth (limited)
0
20000
40000
60000
80000
Genome Position
GLBRCY22
Species
GLBRCY22

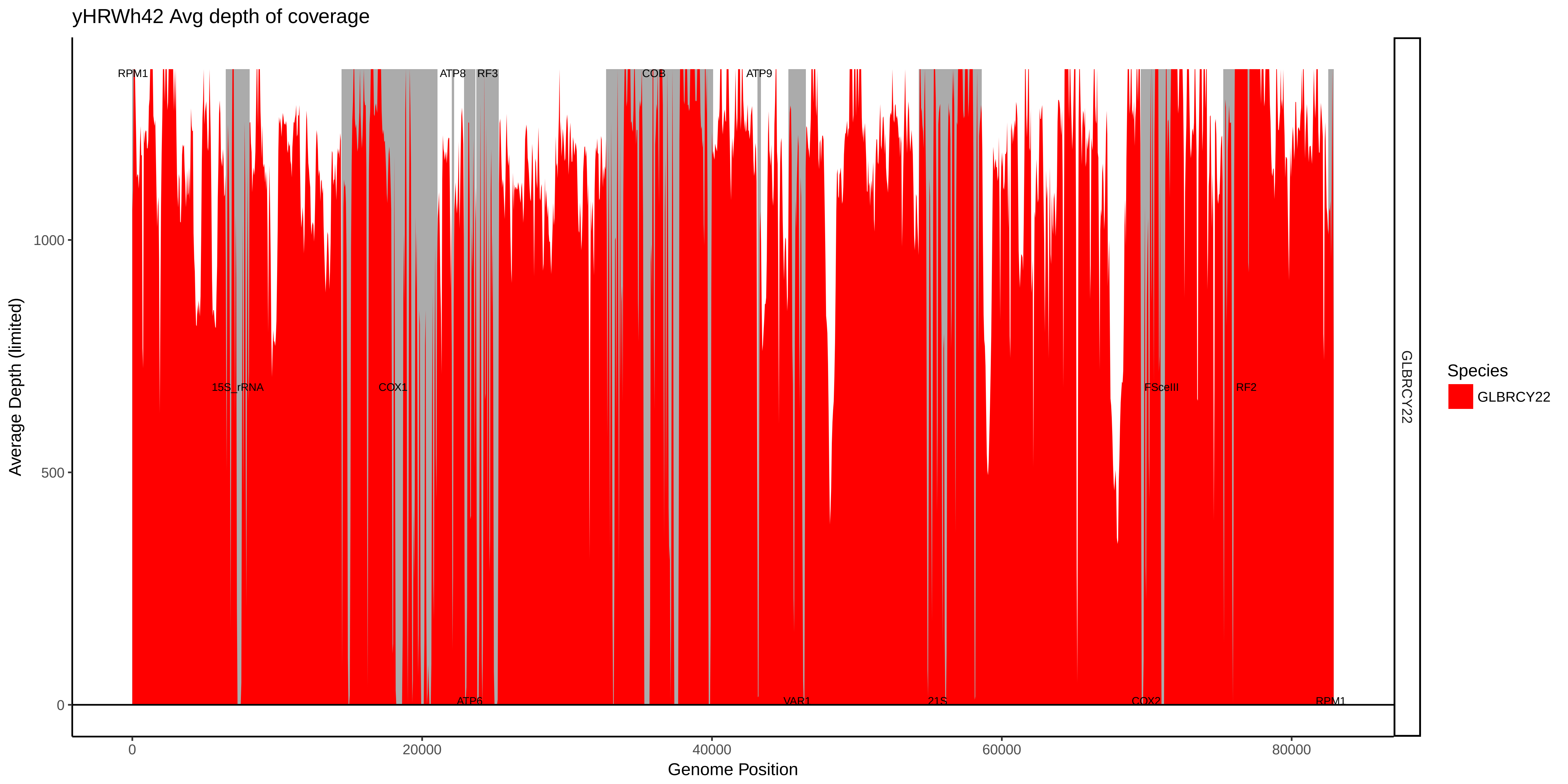

yHRWh42 Avg depth of coverage
RPM1
15S_rRNA
COX1
ATP8
RF3
ATP6
COB
ATP9
VAR1
21S
FScelII
COX2
RF2
RPM1
GLBRCY22
Average Depth (limited)
1000
500
0
0
20000
40000
60000
80000
Genome Position
Species
GLBRCY22
