# Supplementary material for: Synthetic hybrids of six yeast species
Source: Nat Commun. 2020 Apr 29;11:2085. doi: 10.1038/s41467-020-15559-4 (PMC7190663; doi:10.1038/s41467-020-15559-4)
Supplement: Supplementary file 15 — Source Data [file 41467_2020_15559_MOESM15_ESM.zip › Source Data 3.zip!/yHRWh85_mitoSppIDerDepthPlot-d.pdf]

yHRWh85 Avg depth of coverage

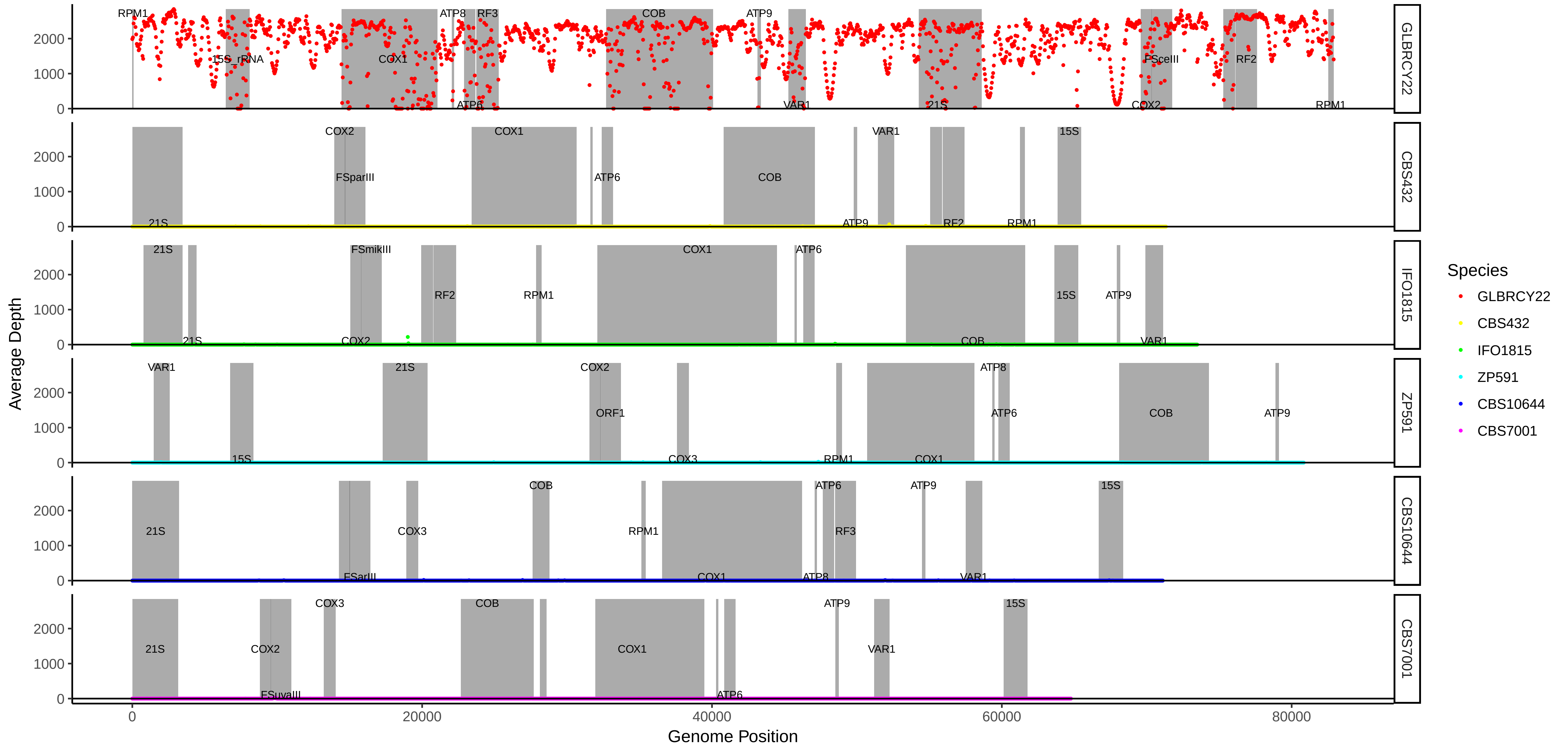

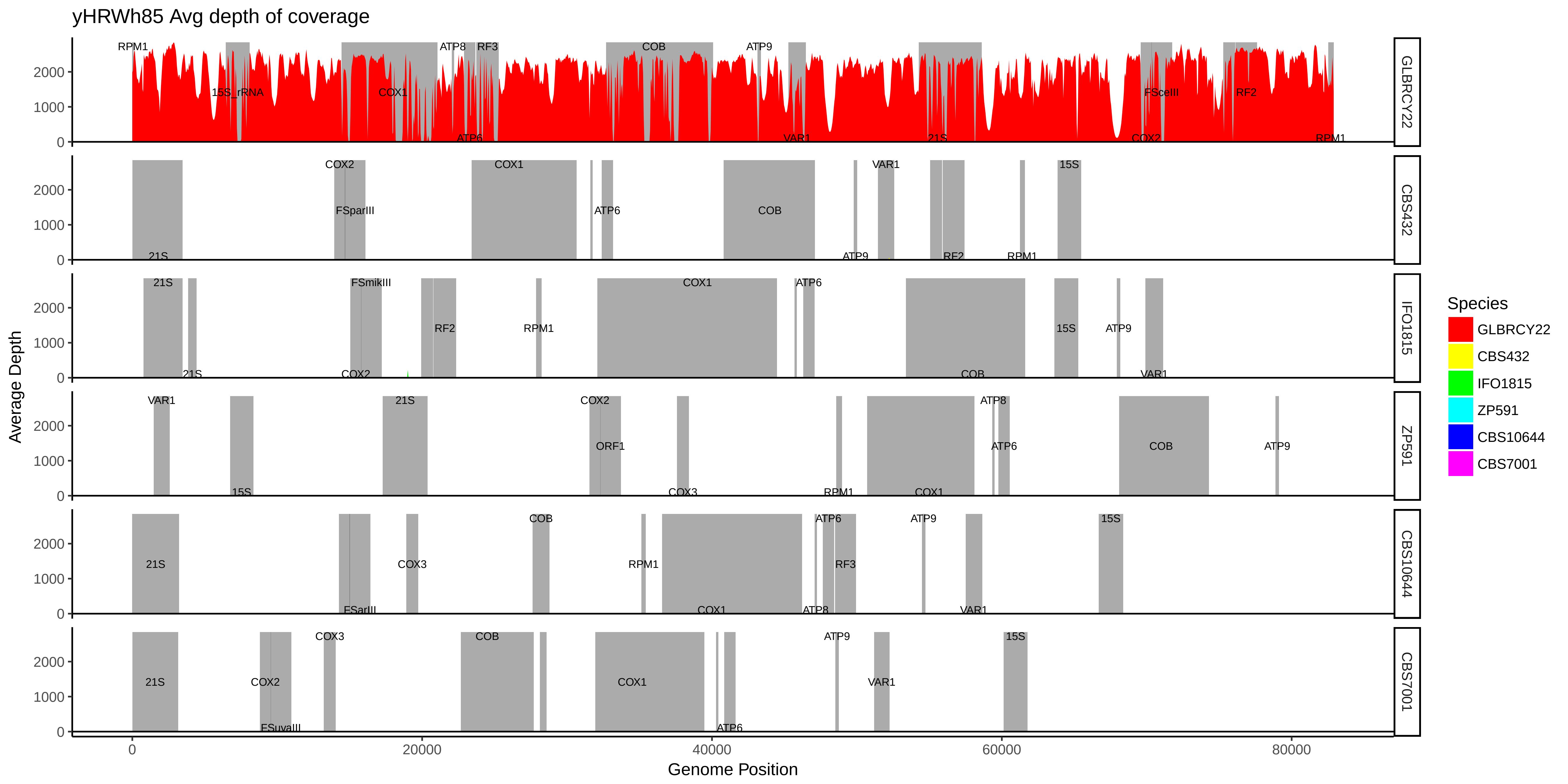

yHRWh85 Avg depth of coverage
Average Depth
Genome Position
Species
GLBRCY22
CBS432
IFO1815
ZP591
CBS10644
CBS7001
0
20000
40000
60000
80000
1000
2000
RPM1
15S_rRNA
COX1
ATP8
RF3
ATP6
COB
ATP9
VAR1
21S
FSceIII
COX2
RF2
COX2
FSparIII
21S
ATP6
COB
ATP9
VAR1
RF2
RPM1
15S
21S
FSmikIII
COX2
RF2
RPM1
COX1
ATP6
COB
15S
ATP9
VAR1
VAR1
15S
21S
COX2
ORF1
COX3
RPM1
COX1
ATP8
ATP6
COB
ATP9
21S
FSarIII
COX3
COB
RPM1
COX1
ATP8
ATP6
RF3
ATP9
VAR1
15S
21S
COX2
FSuvaIII
COX3
COB
COX1
ATP6
ATP9
VAR1
15S

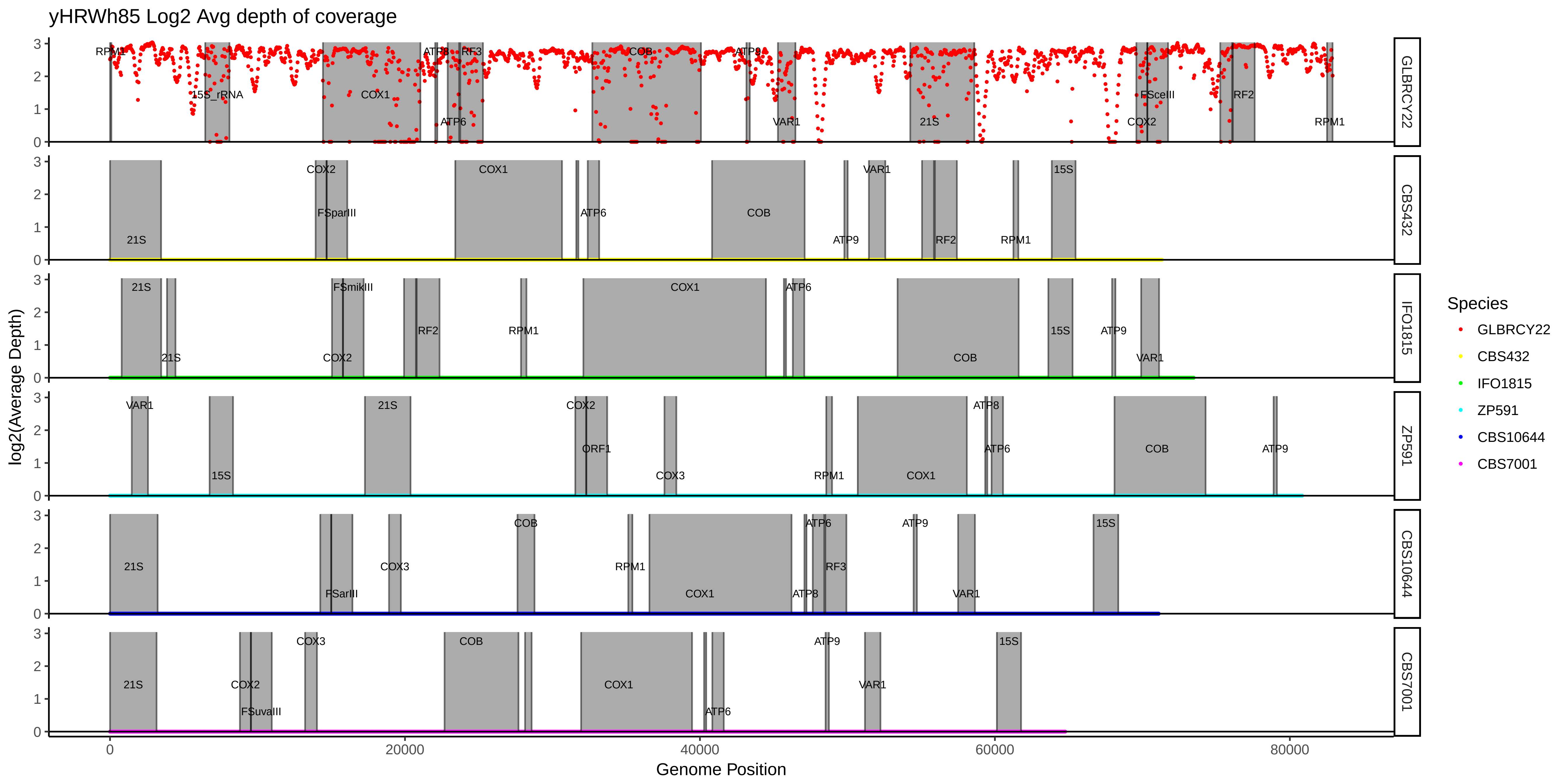
yHRWh85 Log2 Avg depth of coverage
log2(Average Depth)
Genome Position
Species
GLBRCY22
CBS432
IFO1815
ZP591
CBS10644
CBS7001
RPM1
15S_rRNA
COX1
ATP8
RF3
ATP6
COB
ATP9
VAR1
21S
FSceIII
COX2
RF2
21S
COX2
FSparIII
COX1
ATP6
COB
ATP9
VAR1
RF2
RPM1
15S
21S
FSmikIII
COX2
RF2
RPM1
COX1
ATP6
COB
15S
ATP9
VAR1
VAR1
15S
21S
COX2
ORF1
COX3
RPM1
COX1
ATP8
ATP6
COB
ATP9
21S
FSarIII
COX3
COB
RPM1
COX1
ATP6
ATP8
RF3
ATP9
VAR1
15S
21S
COX2
FSuvaIII
COX3
COB
COX1
ATP6
ATP9
VAR1
15S
0
20000
40000
60000
80000

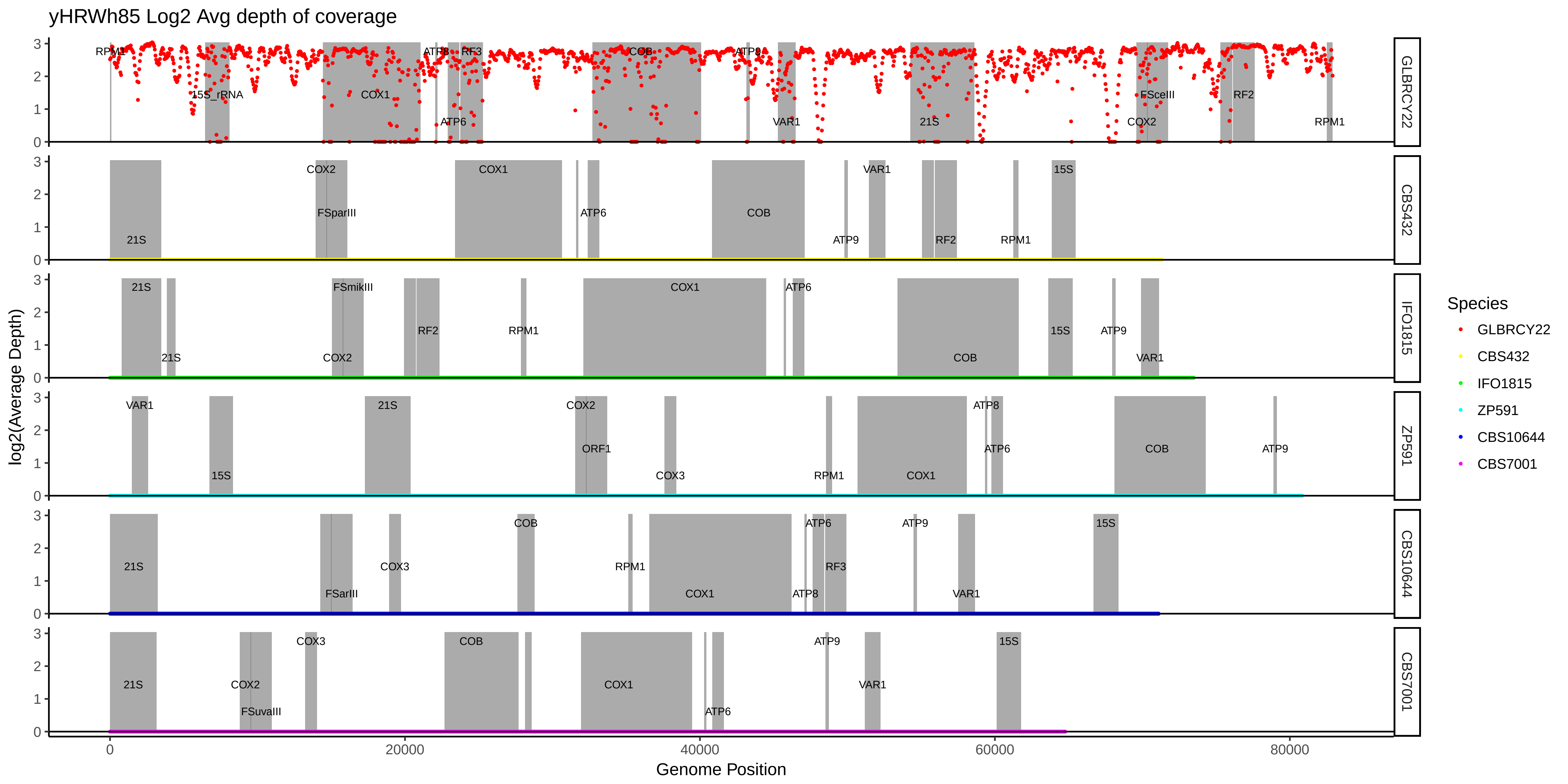
yHRWh85 Log2 Avg depth of coverage
log2(Average Depth)
Genome Position
0
20000
40000
60000
80000
GLBRCY22
CBS432
IFO1815
ZP591
CBS10644
CBS7001
Species
GLBRCY22
CBS432
IFO1815
ZP591
CBS10644
CBS7001
RPM1
15S_rRNA
COX1
ATP8
RF3
ATP6
COB
ATP9
VAR1
21S
FSceIII
COX2
RF2
21S
COX2
FSparIII
COX1
ATP6
COB
ATP9
VAR1
RF2
RPM1
15S
21S
FSmikIII
COX2
RF2
RPM1
COX1
ATP6
COB
15S
ATP9
VAR1
VAR1
15S
21S
COX2
ORF1
COX3
RPM1
COX1
ATP8
ATP6
COB
ATP9
21S
FSarIII
COX3
COB
RPM1
COX1
ATP6
ATP8
RF3
ATP9
VAR1
15S
21S
COX2
FSuvaIII
COX3
COB
COX1
ATP6
ATP9
VAR1
15S

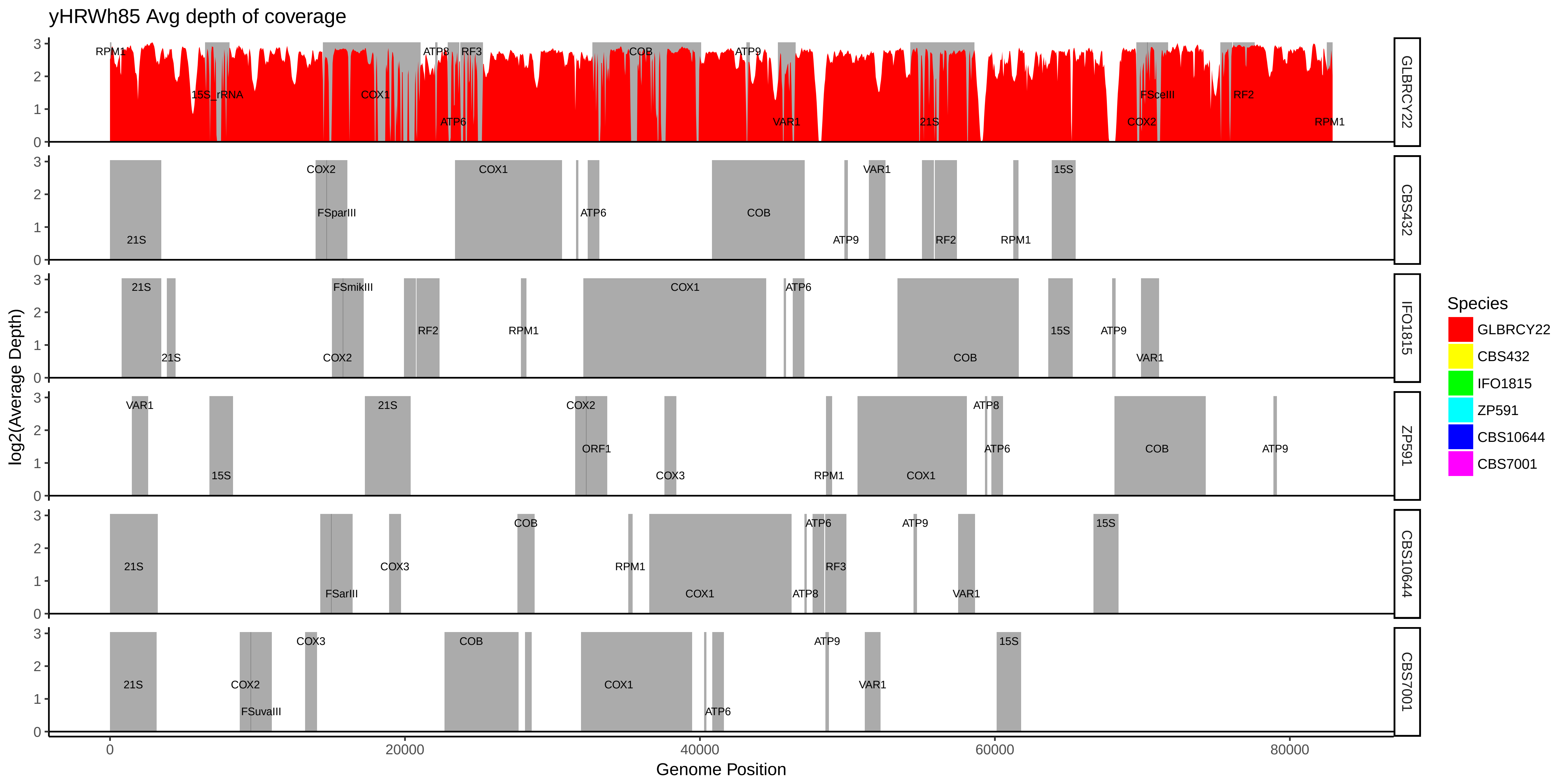

yHRWh85 Avg depth of coverage
log2(Average Depth)
Genome Position
GLBRCY22
CBS432
IFO1815
ZP591
CBS10644
CBS7001
Species
GLBRCY22
CBS432
IFO1815
ZP591
CBS10644
CBS7001
RPM1
15S_rRNA
COX1
ATP8
RF3
ATP6
COB
ATP9
VAR1
21S
FSceIII
COX2
RF2
21S
COX2
FSparIII
COX1
ATP6
COB
ATP9
VAR1
RF2
RPM1
15S
FSmikIII
RPM1
15S
ORF1
COX3
FSarIII
FSuvaIII
0
20000
40000
60000
80000

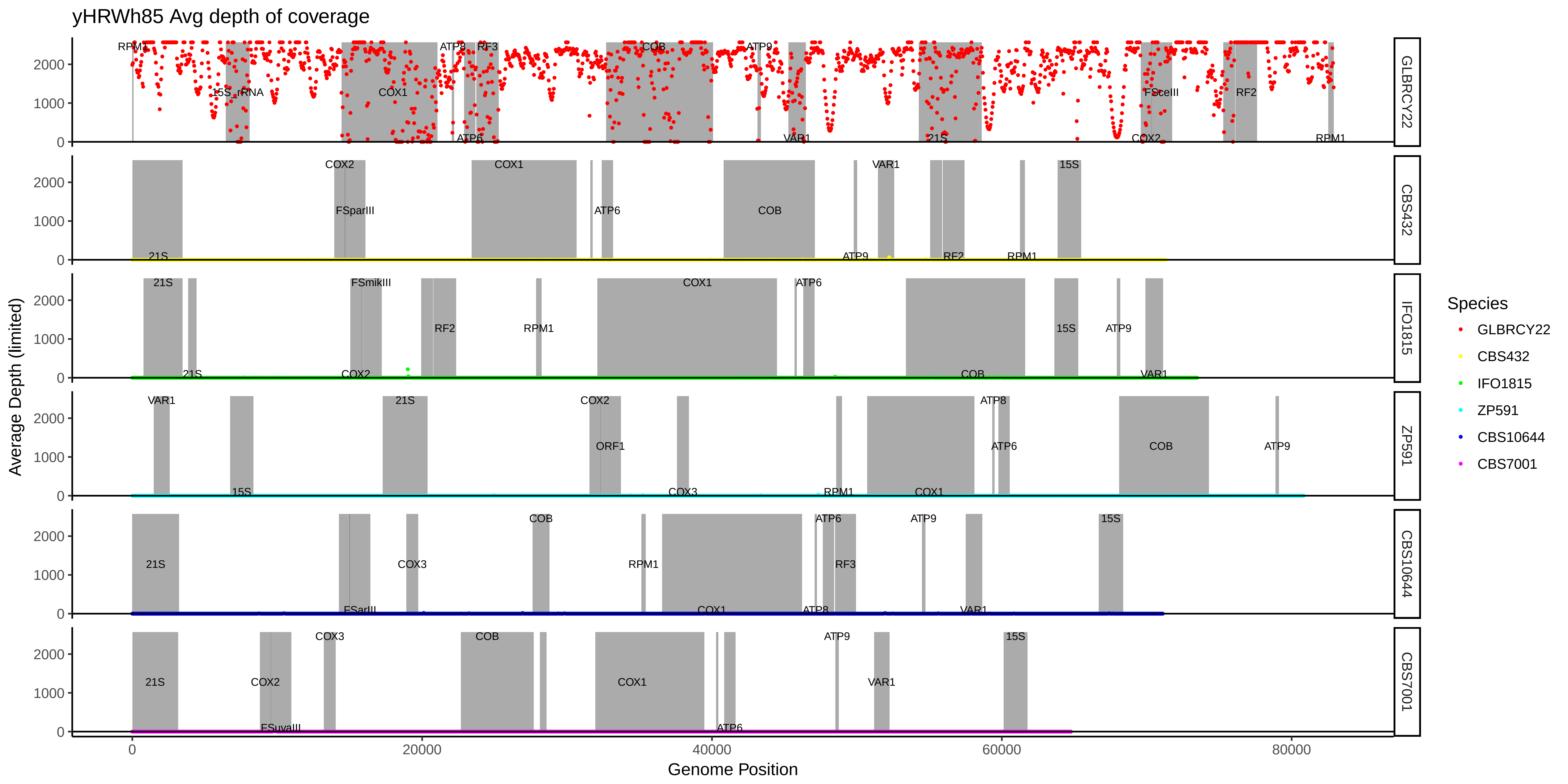

yHRWh85 Avg depth of coverage
Average Depth (limited)
Genome Position
Species
GLBRCY22
CBS432
IFO1815
ZP591
CBS10644
CBS7001
RPM1
15S_rRNA
COX1
ATP8
RF3
ATP6
COB
ATP9
VAR1
21S
FSceIII
COX2
RF2
FSparIII
21S
FSmikIII
ORF1
15S
COX3
FSarIII
FSuvaIII
0
1000
2000
20000
40000
60000
80000

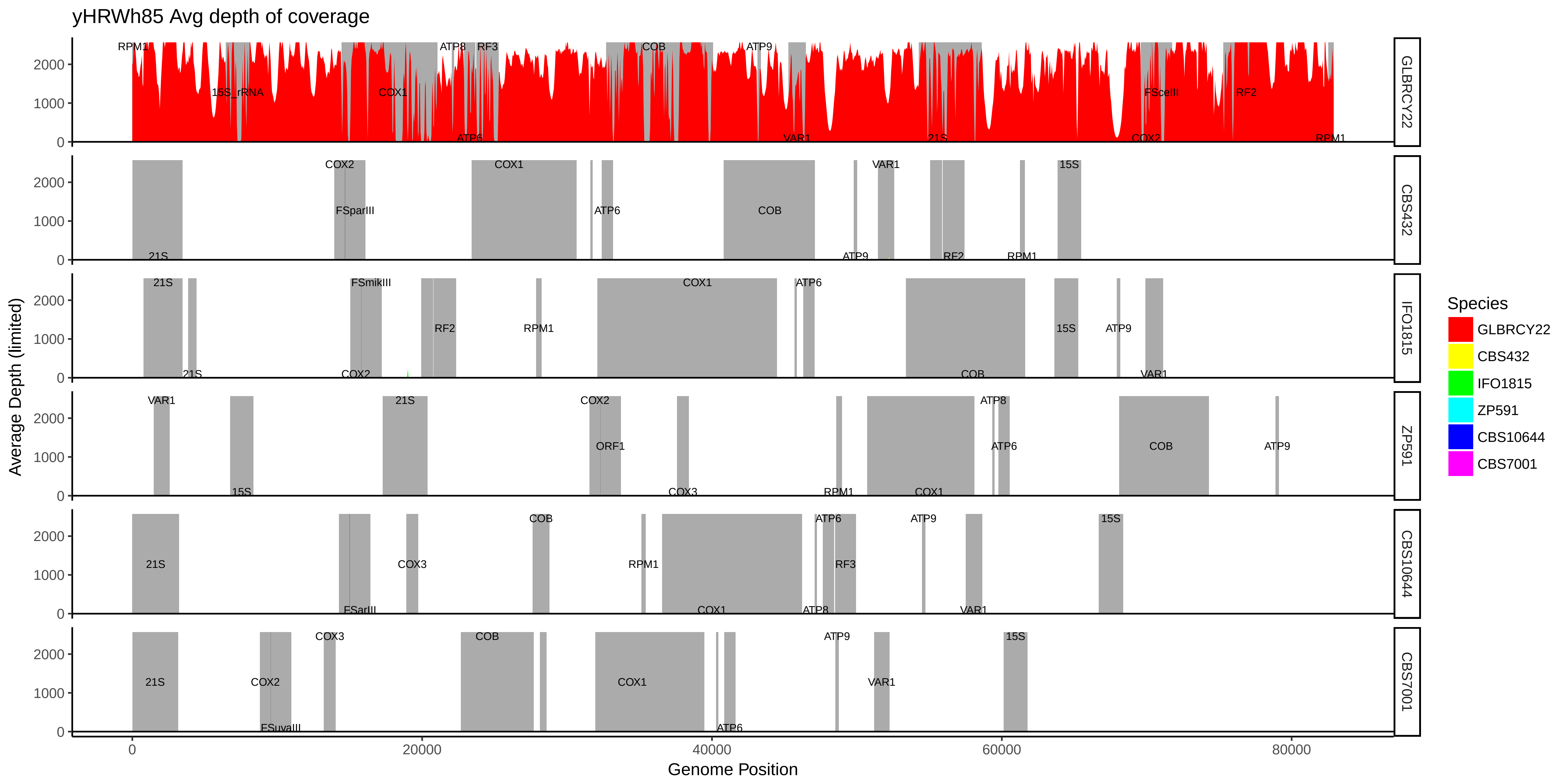
yHRWh85 Avg depth of coverage
Average Depth (limited)
Genome Position
0
20000
40000
60000
80000
1000
2000
GLBRCY22
CBS432
IFO1815
ZP591
CBS10644
CBS7001
Species
GLBRCY22
CBS432
IFO1815
ZP591
CBS10644
CBS7001
RPM1
15S_rRNA
COX1
ATP8
RF3
ATP6
COB
ATP9
VAR1
21S
FSceIII
COX2
RF2
COX2
FSparIII
ATP6
15S
FSmikIII
RF2
ORF1
COX3
FSarIII
FSuvaIII

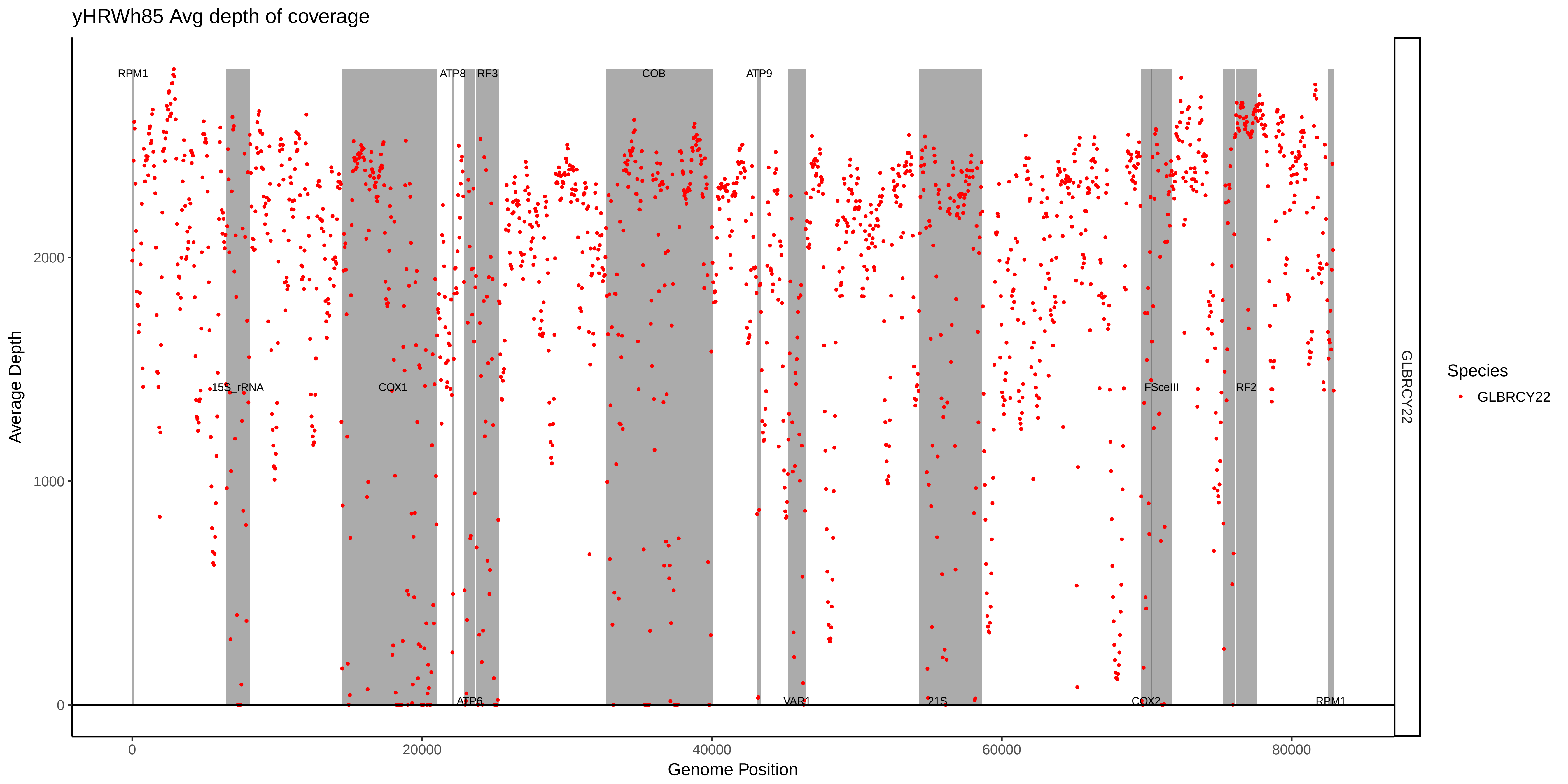
yHRWh85 Avg depth of coverage
RPM1
ATP8
RF3
COB
ATP9
15S_rRNA
COX1
FSceIII
RF2
ATP6
VAR1
21S
COX2
RPM1
GLBRCY22
Average Depth
2000
1000
0
0
20000
40000
60000
80000
Genome Position
Species
GLBRCY22

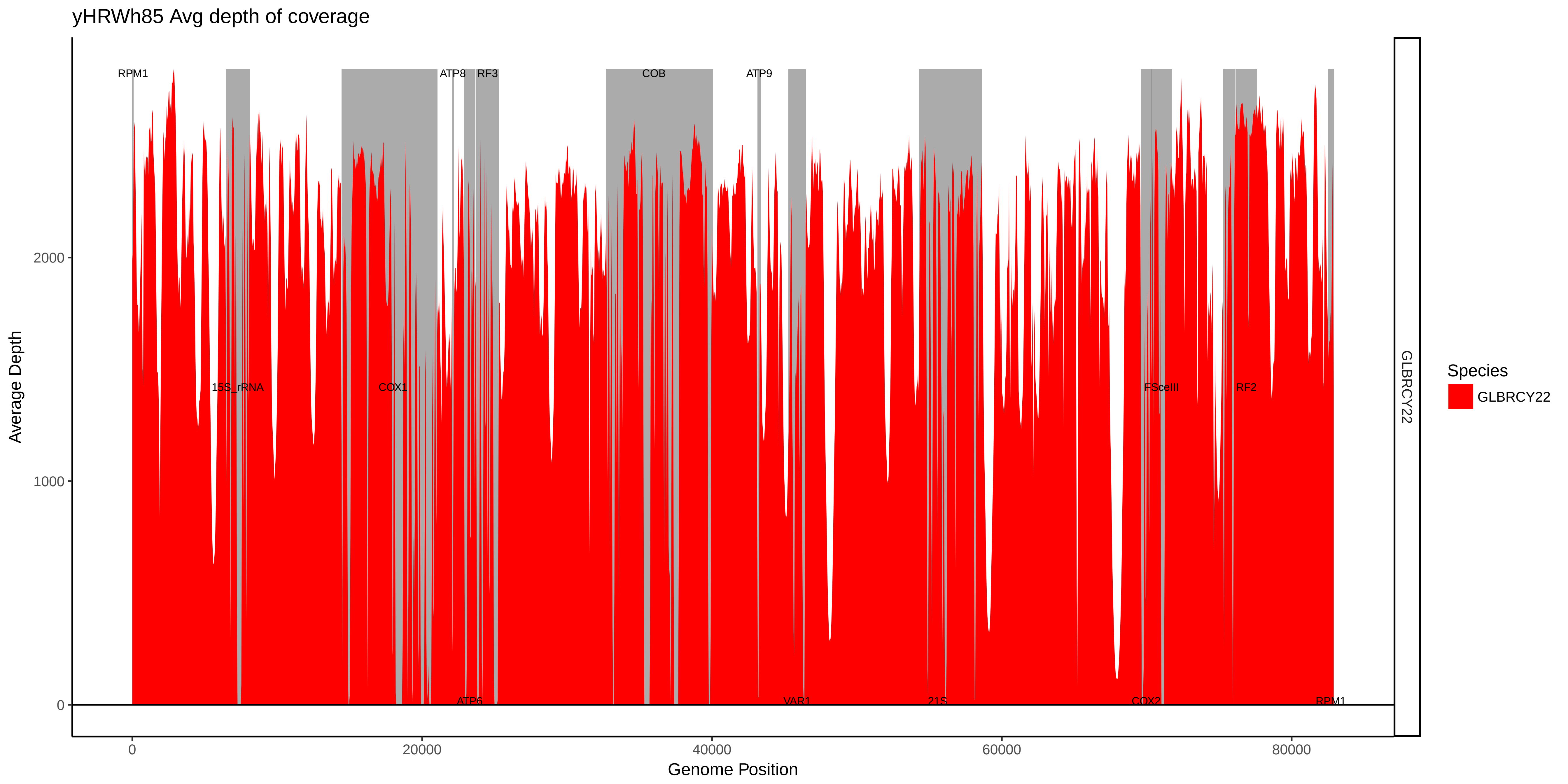

yHRWh85 Avg depth of coverage
Average Depth
Genome Position
0
1000
2000
0
20000
40000
60000
80000
RPM1
ATP8
RF3
COB
ATP9
15S_rRNA
COX1
FScelII
RF2
ATP6
VAR1
21S
COX2
RPM1
GLBRCY22
Species
GLBRCY22

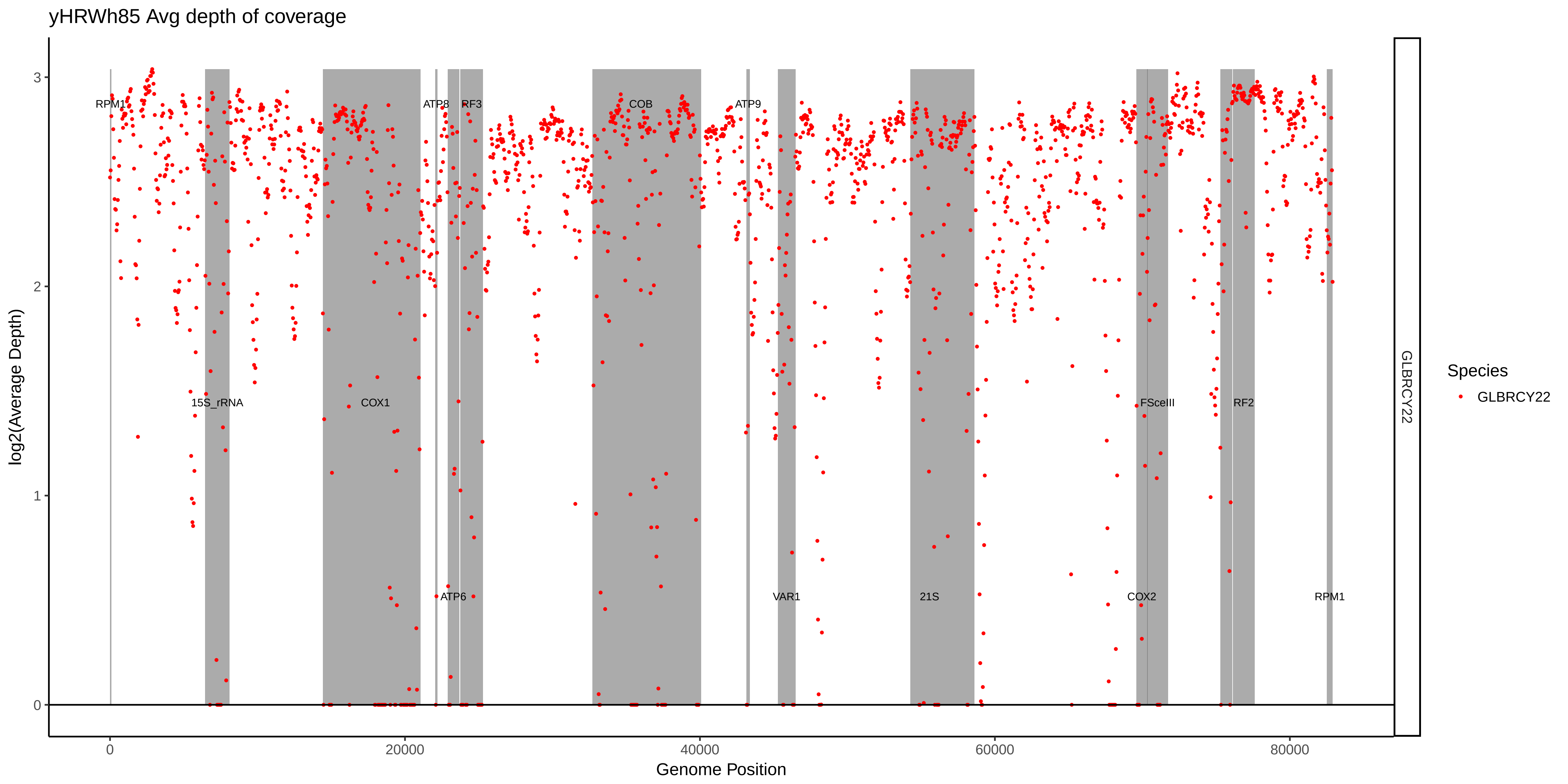

yHRWh85 Avg depth of coverage
RPM1
15S_rRNA
COX1
ATP8
RF3
ATP6
COB
ATP9
VAR1
21S
FSceIII
COX2
RF2
RPM1
log2(Average Depth)
0
1
2
3
0
20000
40000
60000
80000
Genome Position
GLBRCY22
Species
GLBRCY22

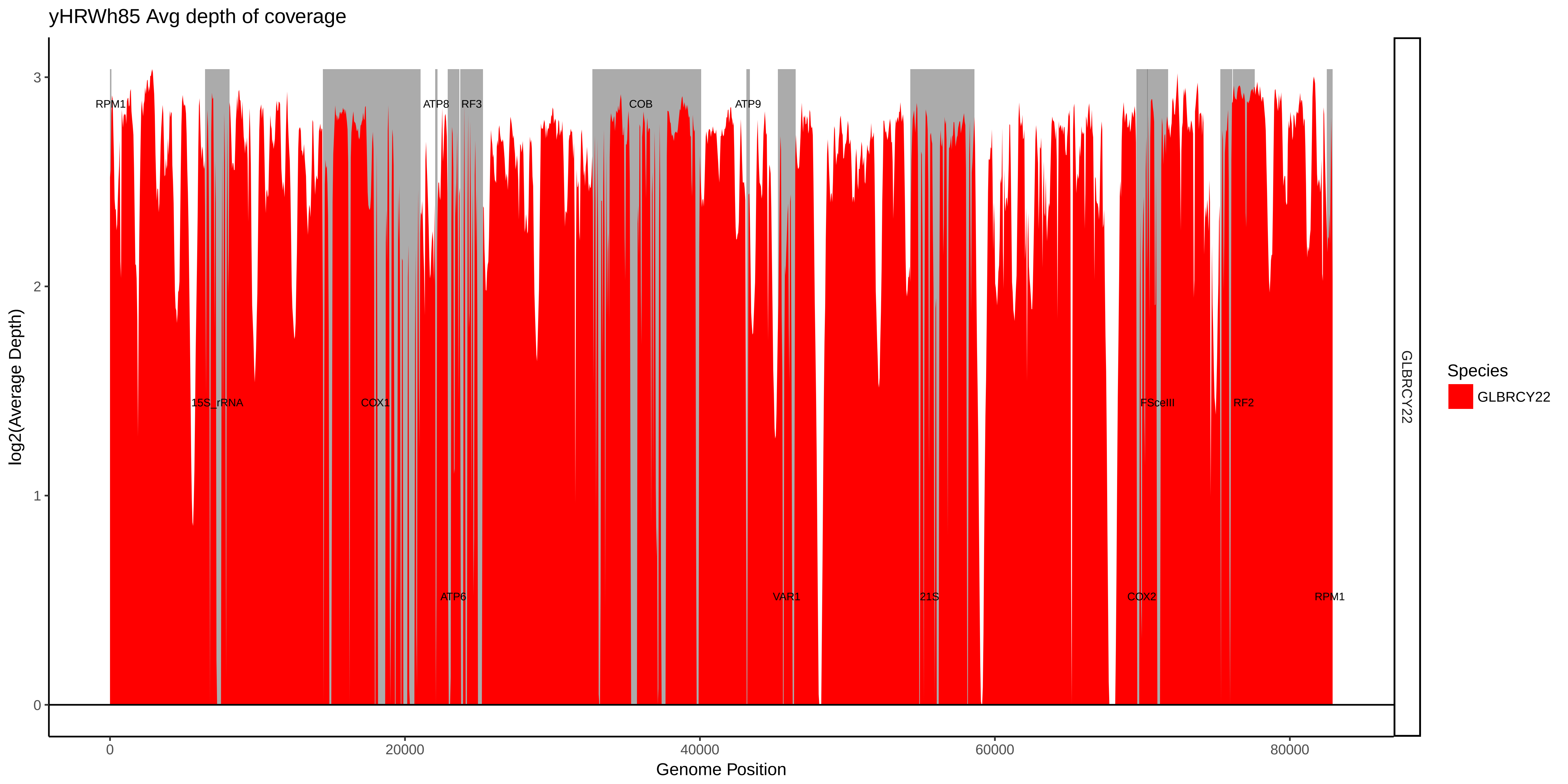
yHRWh85 Avg depth of coverage
log2(Average Depth)
Genome Position
0
1
2
3
0
20000
40000
60000
80000
RPM1
15S_rRNA
COX1
ATP8
ATP6
RF3
COB
ATP9
VAR1
21S
FScelII
COX2
RF2
RPM1
GLBRCY22
Species
GLBRCY22

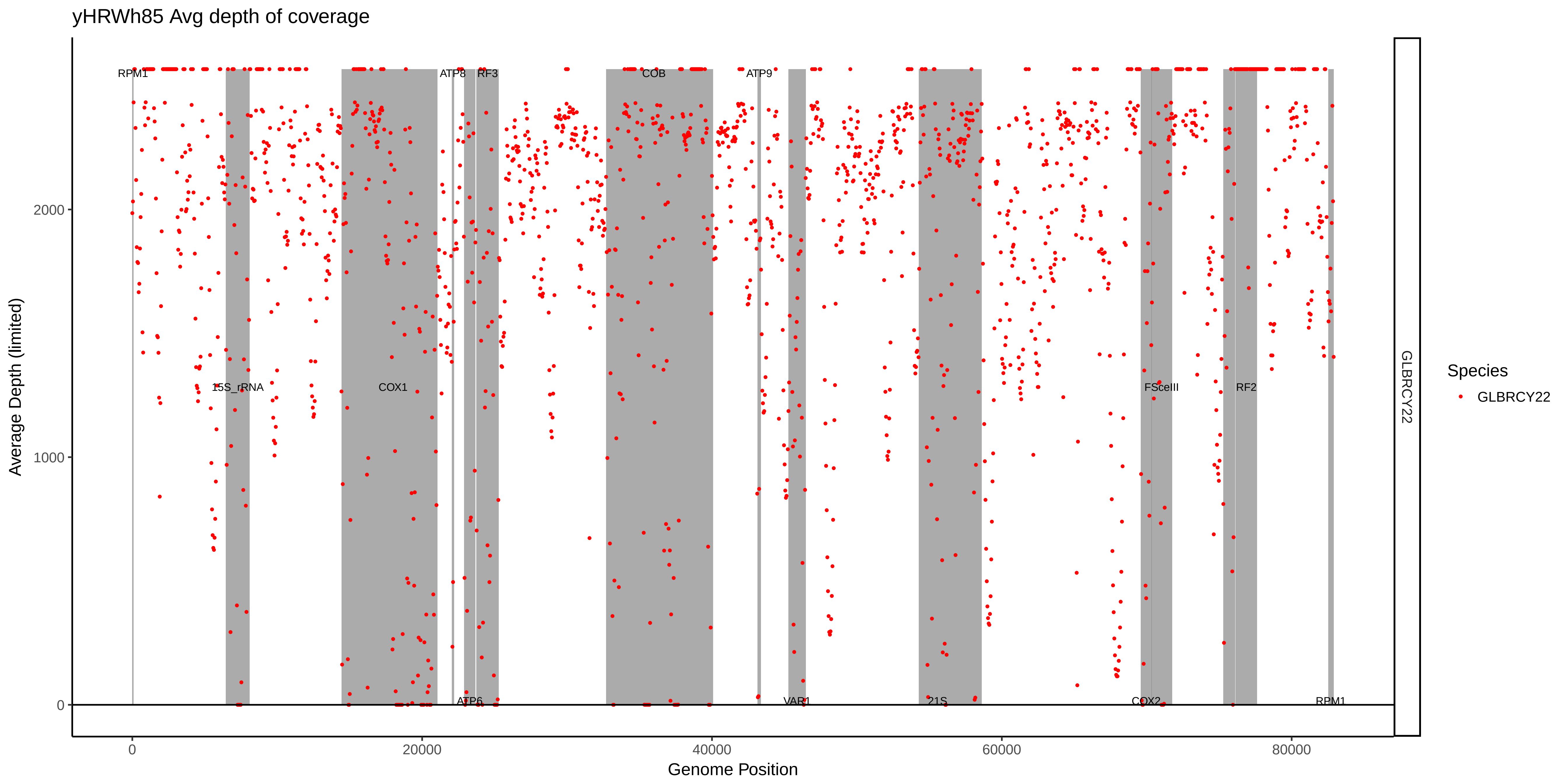

yHRWh85 Avg depth of coverage
RPM1
ATP8
RF3
COB
ATP9
15S_rRNA
COX1
FSceIII
RF2
ATP6
VAR1
21S
COX2
RPM1
GLBRCY22
Species
GLBRCY22
Average Depth (limited)
2000
1000
0
0
20000
40000
60000
80000
Genome Position

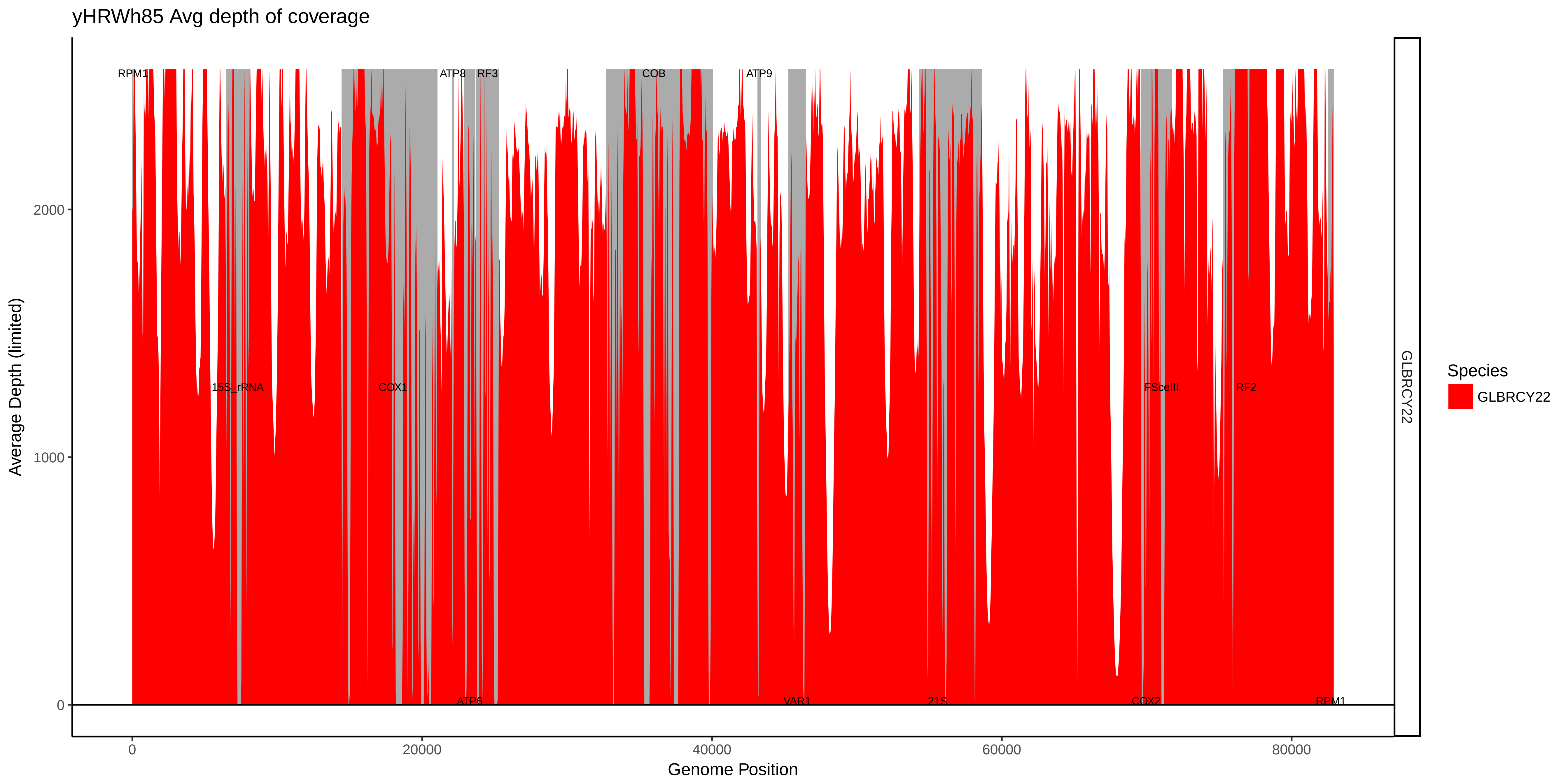

yHRWh85 Avg depth of coverage
RPM1
ATP8
RF3
COB
ATP9
15S_rRNA
COX1
FScelII
RF2
ATP6
VAR1
21S
COX2
RPM1
2000
1000
0
0
20000
40000
60000
80000
Average Depth (limited)
Genome Position
GLBRCY22
Species
GLBRCY22
